# Addendum: Expedient production of site specifically nucleobase-labelled or hypermodified RNA with engineered thermophilic DNA polymerases

**DOI:** 10.1038/s41467-024-48572-y

**Published:** 2024-05-17

**Authors:** Mária Brunderová, Vojtěch Havlíček, Ján Matyašovský, Radek Pohl, Lenka Poštová Slavětínská, Matouš Krömer, Michal Hocek

**Affiliations:** 1 Institute of Organic Chemistry and Biochemistry, Czech Academy of Sciences, Flemingovo nam. 2, CZ-16000, Prague, 6 Czech Republic; 2 Department of Organic Chemistry, Faculty of Science, Charles University, Hlavova 8, CZ-12843, Prague, 2 Czech Republic; 3 Present Address: MRC Laboratory of Molecular Biology, Francis Crick Avenue, Cambridge Biomedical Campus, Cambridge, UK; 4 Present Address: The Rosalind Franklin Institute, Harwell Campus, Didcot, Oxfordshire UK

**Keywords:** RNA, Nucleic acids

Addendum to: *Nature Communications* 10.1038/s41467-024-47444-9, published online 09 April 2024

During the peer review of this Article, a related work reporting a polymerase-driven method for site-specific and segmental labeling of RNA was published in Haslecker, R., Pham, V.V., Glänzer, D. et al. Extending the toolbox for RNA biology with SegModTeX: a polymerase-driven method for site-specific and segmental labeling of RNA. *Nat. Commun.* **14**, 8422 (2023). 10.1038/s41467-023-44254-3

