# Supplementary material for: Expedient production of site specifically nucleobase-labelled or hypermodified RNA with engineered thermophilic DNA polymerases
Source: Nat Commun. 2024 Apr 9;15:3054. doi: 10.1038/s41467-024-47444-9 (PMC11004144; doi:10.1038/s41467-024-47444-9)

Supplementary Data

Expedient production of site specifically nucleobase-labelled or hypermodified RNA with engineered thermophilic DNA polymerases

Mária Brunderová[1,2,3], Vojtěch Havlíček[1,2], Ján Matyašovský[1], Radek Pohl[1], Lenka Poštová Slavětínská[1], Matouš Krömer[1,4]* and Michal Hocek[1,2]*

1) Institute of Organic Chemistry and Biochemistry, Czech Academy of Sciences, Flemingovo nam. 2, CZ-16000 Prague 6, Czech Republic

2) Department of Organic Chemistry, Faculty of Science, Charles University, Hlavova 8, CZ-12843 Prague 2, Czech Republic

3) present address: MRC Laboratory of Molecular Biology, Francis Crick Avenue, Cambridge Biomedical Campus, Cambridge, UK

4) present address: The Rosalind Franklin Institute, Harwell Campus, Didcot, Oxfordshire, UK

correspondence: matous.kromer@uochb.cas.cz or hocek@uochb.cas.cz

Table of contents

1 Synthetic part 12

1.1 General remarks 12

1.2 General procedure for Sonogashira cross-coupling reaction on nucleosides 12

1.3 General procedure for triphosphorylation reaction of pentyne-modified nucleosides (rN^{Pent}) 13

1.4 Chemical synthesis of previously published nucleosides and nucleotides 13

1.4.1 Chemical synthesis of rU^{Pent} 13

1.4.2 Chemical synthesis of rA^{CA}TP 13

1.4.3 Chemical synthesis of rU^{FT}TP 13

1.4.4 Chemical synthesis of rA^{E}TP, A^{Ph}TP, rU^{E}TP, rU^{Ph}TP, rC^{E}TP, rC^{Ph}TP, rG^{E}TP, rG^{Ph}TP 13

1.5 Chemical synthesis of novel nucleosides 14

1.5.1 Chemical synthesis of 7-(pent-1-yn-1-yl)-7-deazaadenosine (rA^{Pent}) 14

1.5.2 Chemical synthesis of 5-{3-[4-(5,5-difluoro-1,3,7,9-tetramethyl-5*H*-4λ^4,5λ^4-dipyrrolo[1,2-c:2',1'-f][1,3,2]diazaborinin-10-yl)-3,5-dimethylphenoxy]prop-1-yn-1-yl}-cytidine (rC^{mBdp}) 14

1.5.3 Chemical synthesis of 5-(pent-1-yn-1-yl)-cytidine (rC^{Pent}) 15

1.5.4 Chemical synthesis of 7-(pent-1-yn-1-yl)-7-deazaguanosine (rG^{Pent}) 16

1.6 Chemical synthesis of novel nucleotides 16

1.6.1 Chemical synthesis of 7-(5-formylthien-2-yl)-7-deazaadenosine-5'-*O*-triphosphate (rA^{FT}TP) 16

1.6.2 Chemical synthesis of 7-(pent-1-yn-1-yl)-7-deazaadenosine-5´-*O*-triphosphate (rA^{Pent}TP) 17

1.6.3 Chemical synthesis of 5-{3-[N-(2-chloroacetamido)]-prop-2-yn-1-yl}-uridine-5'-*O*-triphosphate (rU^{CA}TP) 18

1.6.4 Chemical synthesis of 5-(pent-1-yn-1-yl)-uridine-5´-*O*-triphosphate (rU^{Pent}TP) 19

1.6.5 Chemical synthesis of 5-{3-[N-(2-chloroacetamido)]-prop-2-yn-1-yl}-cytidine-5'-*O*-triphosphate (rC^{CA}TP) 20

1.6.6 Chemical synthesis of 5-(5-formylthien-2-yl)-cytidine-5'-*O*-triphosphate (rC^{FT}TP) 21

1.6.7 Chemical synthesis of 5-{3-[4-(5,5-difluoro-1,3,7,9-tetramethyl-5*H*-4λ^4,5λ^4-dipyrrolo[1,2-c:2',1'-f][1,3,2]diazaborinin-10-yl)-3,5-dimethylphenoxy]prop-1-yn-1-yl}-cytidine-5´-*O*-triphosphate (rC^{mBdp}TP) 22

1.6.8 Chemical synthesis of 5-(pent-1-yn-1-yl)-cytidine-5´-*O*-triphosphate (rC^{Pent}TP) 23

1.6.9 Chemical synthesis of 7-(pent-1-yn-1-yl)-7-deazaguanosine-5'-*O*-triphosphate (rG^{Pent}TP) ... 23

2 Biochemical part ... 25

2.1 General remarks ... 25

2.2 List of oligonucleotide sequences ... 26

2.2.1 RNA oligonucleotides used in this study ... 26

2.2.2 DNA oligonucleotides used in this study ... 27

2.2.3 Oligonucleotide modifications ... 31

2.3 Chemical structures of base-modified rN^{X}TPs used in this study ... 32

2.3.1 Chemical structures of hydrophobic and/or clickable base-modified rN^{X}TPs ... 32

2.3.2 Chemical structures of fluorescent base-modified rN^{X}TPs ... 33

2.3.3 Chemical structures of hapten base-modified rN^{X}TPs ... 34

2.3.4 Chemical structures of reactive base-modified rN^{X}TPs ... 35

2.4 Engineered DNA polymerases – expression and purification ... 35

2.4.1 Protocol for TGK polymerase[12,13] ... 35

2.4.2 Protocol for SFM4-3 polymerase[14] ... 36

2.5 General procedures ... 36

2.5.1 Sample preparation protocol for dPAGE analysis ... 36

2.5.2 Urea agarose gel analysis ... 36

2.5.3 Preparation of dsDNA templates for IVT reactions ... 37

2.5.4 Standard conditions for analytical scale PEX reaction using templ_19nt_X (X = A, U, C, G) ... 37

2.5.5 Modified conditions for analytical scale PEX reaction using templ_19nt_X (X = A, U, C) ... 37

2.5.6 Procedure for analytical scale PEX reaction at mild conditions using templ_19nt_X (X = A, C) ... 37

2.5.7 Standard conditions for analytical scale PEX reaction using 5´-(TINA)-templ_31nt 37

2.5.8 Standard conditions for analytical scale PEX reaction using four different base-modified rN^{X}TPs and variously long templates ... 38

2.5.9 Standard conditions for semi-preparative scale PEX reaction using templ_19nt_X (X = A, U, C, G) followed by spin column purification ... 38

2.5.10 Modified conditions I for semi-preparative scale PEX reaction using templ_19nt_X (X = A, C) followed by spin column purification ... 38

2.5.11 Modified conditions II for semi-preparative scale PEX reaction using templ_19nt_X (X = A, U, C) followed by spin column purification ... 38

2.5.12 Procedure for semi-preparative scale PEX reaction at mild conditions using templ_19nt_X (X = A, C) followed by spin column purification 39

2.5.13 Conditions for semi-preparative scale PEX reaction using 5´-(TINA)-templ_31nt followed by spin column purification 39

2.5.14 Conditions for semi-preparative scale PEX reaction using 5´-(dual-Bio)-templ_31nt followed by magnetoseparation 39

2.5.15 Conditions for semi-preparative scale PEX reaction using four different base-modified rN^{X}TPs and variously long templates 39

2.5.16 Standard protocol for RNA digestion and mass spectrometry analysis 40

2.5.17 Standard magnetoseparation procedure for generation of ssDNA templates after PCR 40

2.5.18 Standard conditions for PCR reaction 40

2.5.19 Standard work up protocol for either IRES-RNA or IRES-RNA_prolonged 41

2.5.20 Standard work up protocol for mRNA 41

2.5.21 Standard reaction conditions for RT-PCR 41

2.6 Analytical scale PEX reaction with templ_19nt_X (X = A, U, C, G) and TGK polymerase (incorporation of 1 modification) 41

2.6.1 Incorporation of rA^{E}TP, rA^{Pent}TP, rA^{Ph}TP 41

2.6.2 Incorporation of rA^{CA}TP, rA^{FT}TP 42

2.6.3 Incorporation of rA^{CA}TP at mild conditions 43

2.6.4 Incorporation of rU^{E}TP, rU^{Pent}TP, rU^{Ph}TP 43

2.6.5 Incorporation of rU^{Bio}TP, rU^{Dig}TP 44

2.6.6 Incorporation of rU^{CA}TP, rU^{FT}TP 45

2.6.7 Incorporation of rC^{Me}TP 45

2.6.8 Incorporation of rC^{E}TP, rC^{Pent}TP, rC^{Ph}TP 46

2.6.9 Incorporation of rC^{mBdp}TP 47

2.6.10 Incorporation of rC^{Cy5}TP 47

2.6.11 Incorporation of rC^{CA}TP, rC^{FT}TP 48

2.6.12 Incorporation of rC^{CA}TP at mild conditions 49

2.6.13 Incorporation of rG^{E}TP, rG^{Pent}TP, rG^{Ph}TP 49

2.7 Analytical scale PEX reaction with templ_19nt_X (X = A, U, C, G) and SFM4-3 polymerase (incorporation of 1 modification) 50

2.7.1 Incorporation of rA^{E}TP, rA^{Pent}TP, rA^{Ph}TP 50

2.7.2 Incorporation of rA^{CA}TP, rA^{FT}TP 51

2.7.3 Incorporation of rA^{CA}TP at mild conditions 52

2.7.4 Incorporation of $rU^{E}TP$, $rU^{Pent}TP$, $rU^{Ph}TP$ 52
2.7.5 Incorporation of $rU^{Bio}TP$, $rU^{Dig}TP$ 53
2.7.6 Incorporation of $rU^{CA}TP$, $rU^{FT}TP$ 54
2.7.7 Incorporation of $rC^{E}TP$, $rC^{Pent}TP$, $rC^{Ph}TP$ 54
2.7.8 Incorporation of $rC^{mBdp}TP$ 55
2.7.9 Incorporation of $rC^{Cy5}TP$ 56
2.7.10 Incorporation of $rC^{CA}TP$, $rC^{FT}TP$ 56
2.7.11 Incorporation of $rC^{CA}TP$ at mild conditions 57
2.7.12 Incorporation of $rG^{E}TP$, $rG^{Pent}TP$, $rG^{Ph}TP$ 57
2.8 Semi-preparative scale PEX reaction with templ_19nt_X (X = A, U, C, G) and TGK polymerase (incorporation of 1 modification) 58
2.8.1 Incorporation of $rA^{E}TP$, $rA^{Pent}TP$, $rA^{Ph}TP$ 58
2.8.2 Incorporation of $rA^{CA}TP$, $rA^{FT}TP$ 58
2.8.3 Incorporation of $rA^{CA}TP$ at reduced time 59
2.8.4 Incorporation of $rA^{CA}TP$ at mild conditions 59
2.8.5 Incorporation of $rU^{E}TP$, $rU^{Pent}TP$, $rU^{Ph}TP$ 59
2.8.6 Incorporation of $rU^{Bio}TP$, $rU^{Dig}TP$ 59
2.8.7 Incorporation of $rU^{CA}TP$, $rU^{FT}TP$ 59
2.8.8 Incorporation of $rC^{Me}TP$ 60
2.8.9 Incorporation of $rC^{E}TP$, $rC^{Pent}TP$, $rC^{Ph}TP$ 60
2.8.10 Incorporation of $rC^{mBdp}TP$ 60
2.8.11 Incorporation of $rC^{Cy5}TP$ 60
2.8.12 Incorporation of $rC^{CA}TP$, $rC^{FT}TP$ 60
2.8.13 Incorporation of $rC^{CA}TP$ at reduced time 61
2.8.14 Incorporation of $rC^{CA}TP$ at mild conditions 61
2.8.15 Incorporation of $rG^{E}TP$, $rG^{Pent}TP$, $rG^{Ph}TP$ 61
2.9 Semi-preparative scale PEX reaction with templ_19nt_X (X = A, U, C, G) and SFM4-3 polymerase (incorporation of 1 modification) 61
2.9.1 Incorporation of $rA^{E}TP$, $rA^{Pent}TP$, $rA^{Ph}TP$ using SFM4-3 polymerase 61
2.9.2 Incorporation of $rA^{CA}TP$, $rA^{FT}TP$ 61
2.9.3 Incorporation of $rA^{CA}TP$ at mild conditions 62
2.9.4 Incorporation of $rU^{E}TP$, $rU^{Pent}TP$, $rU^{Ph}TP$ 62
2.9.5 Incorporation of $rU^{Bio}TP$, $rU^{Dig}TP$ 62
2.9.6 Incorporation of $rU^{CA}TP$, $rU^{FT}TP$ 62

2.9.7 Incorporation of $rC^{E}TP$, $rC^{Pent}TP$, $rC^{Ph}TP$ 62

2.9.8 Incorporation of $rC^{mBdp}TP$ 63

2.9.9 Incorporation of $rC^{Cy5}TP$ 63

2.9.10 Incorporation of $rC^{CA}TP$, $rC^{FT}TP$ 63

2.9.11 Incorporation of $rC^{CA}TP$ at mild conditions 63

2.9.12 Incorporation of $rG^{E}TP$, $rG^{Pent}TP$, $rG^{Ph}TP$ 63

2.10 Analytical scale PEX reaction with 5´-(TINA)-templ_31nt and TGK polymerase (incorporation of 4 modifications) 64

2.10.1 Incorporation of $rA^{E}TP$, $rA^{Pent}TP$, $rA^{Ph}TP$ 64

2.10.2 Incorporation of $rU^{E}TP$, $rU^{Pent}TP$, $rU^{Ph}TP$ 65

2.10.3 Incorporation of $rU^{Bio}TP$, $rU^{Dig}TP$ 65

2.10.4 Incorporation of $rC^{Me}TP$ 66

2.10.5 Incorporation of $rC^{E}TP$, $rC^{Pent}TP$, $rC^{Ph}TP$ 67

2.10.6 Incorporation of $rC^{mBdp}TP$ 68

2.10.7 Incorporation of $rG^{E}TP$, $rG^{Pent}TP$, $rG^{Ph}TP$ 69

2.11 Analytical scale PEX reaction with 5´-(TINA)-templ_31nt and SFM4-3 polymerase (incorporation of 4 modifications) 70

2.11.1 Incorporation of $rA^{E}TP$, $rA^{Pent}TP$, $rA^{Ph}TP$ 70

2.11.2 Incorporation of $rU^{E}TP$, $rU^{Pent}TP$, $rU^{Ph}TP$ 71

2.11.3 Incorporation of $rU^{Bio}TP$, $rU^{Dig}TP$ 72

2.11.4 Incorporation of $rC^{E}TP$, $rC^{Pent}TP$, $rC^{Ph}TP$ 73

2.11.5 Incorporation of $rC^{mBdp}TP$ 74

2.11.6 Incorporation of $rG^{E}TP$, $rG^{Pent}TP$, $rG^{Ph}TP$ 75

2.12 Semi-preparative scale PEX reaction with 5´-(TINA)-templ_31nt or 5´-(dual-Bio)-templ_31nt and preparative scale PEX with templ_31nt and TGK polymerase (incorporation of 4 modifications) 76

2.12.1 Incorporation of $rA^{E}TP$, $rA^{Pent}TP$, $rA^{Ph}TP$ 76

2.12.2 Incorporation of $rU^{E}TP$, $rU^{Pent}TP$, $rU^{Ph}TP$ 76

2.12.3 Incorporation of $rU^{Bio}TP$, $rU^{Dig}TP$ 77

2.12.4 Incorporation of $rC^{Me}TP$ 77

2.12.5 Incorporation of $rC^{E}TP$, $rC^{Pent}TP$, $rC^{Ph}TP$ 77

2.12.6 Incorporation of $rC^{mBdp}TP$ 77

2.12.7 Incorporation of $rG^{E}TP$, $rG^{Pent}TP$, $rG^{Ph}TP$ 77

2.12.8 Preparative (1 nmol scale) PEX reaction with natural rNTPs 78

2.12.9 Preparative (1 nmol scale) PEX reaction with a mixture of rA^{E}TP, rUTP, rCTP, rGTP ... 78

2.12.10 Preparative (1 nmol scale) PEX reaction with a mixture of rATP, rU^{Bio}TP, rCTP, rGTP ... 78

2.12.11 Preparative (1 nmol scale) PEX reaction with a mixture of rATP, rUTP, rC^{Me}TP, rGTP ... 79

2.12.12 Preparative (1 nmol scale) PEX reaction with a mixture of rATP, rUTP, rCTP, rG^{Pent}TP ... 79

2.13 Semi-preparative scale PEX reaction with with 5´-(TINA)-templ_31nt or 5´-(dual-Bio)-templ_31nt and SFM4-3 polymerase (incorporation of 4 modifications) ... 81

2.13.1 Incorporation of rA^{E}TP, rA^{Pent}TP, rA^{Ph}TP ... 81

2.13.2 Incorporation of rU^{E}TP, rU^{Pent}TP, rU^{Ph}TP ... 81

2.13.3 Incorporation of rU^{Bio}TP, rU^{Dig}TP ... 81

2.13.4 Incorporation of rC^{E}TP, rC^{Pent}TP, rC^{Ph}TP ... 82

2.13.5 Incorporation of rC^{mBdp}TP ... 82

2.13.6 Incorporation of rG^{E}TP, rG^{Pent}TP, rG^{Ph}TP ... 82

2.14 Analytical scale PEX reaction with combination of four different base-modified rN^{X}TPs and variously long templates ... 82

2.14.1 PEX with rA^{E}TP, rU^{Bio}TP, rC^{Ph}TP, rG^{Pent}TP and templ_19nt_mix using TGK polymerase ... 82

2.14.2 PEX with rA^{E}TP, rU^{Bio}TP, rC^{Ph}TP, rG^{Pent}TP and 5´-(TINA)-templ_31nt using TGK polymerase ... 83

2.14.3 PEX with rA^{E}TP, rU^{Bio}TP, rC^{Ph}TP, rG^{Pent}TP and templ_65nt using TGK polymerase. ... 84

2.14.4 PEX with rA^{E}TP, rU^{Bio}TP, rC^{Ph}TP, rG^{Pent}TP and templ_98nt using TGK polymerase. ... 86

2.14.5 PEX with rA^{E}TP, rU^{Bio}TP, rC^{Ph}TP, rG^{Pent}TP and templ_19nt_mix using SFM4-3 polymerase ... 88

2.14.6 PEX with rA^{E}TP, rU^{Bio}TP, rC^{Ph}TP, rG^{Pent}TP and 5´-(TINA)-templ_31nt using SFM4-3 polymerase ... 89

2.14.7 PEX with rA^{E}TP, rU^{Bio}TP, rC^{Ph}TP, rG^{Pent}TP and templ_65nt using SFM4-3 polymerase ... 90

2.14.8 PEX with rA^{E}TP, rU^{Bio}TP, rC^{Ph}TP, rG^{Pent}TP and templ_98nt using SFM4-3 polymerase ... 92

2.15 Semi-preparative scale PEX reaction with combination of four different base-modified rN^{X}TPs and variously long templates ... 94

2.15.1 PEX with rA^{E}TP, rU^{Bio}TP, rC^{Ph}TP, rG^{Pent}TP and templ_19nt_mix using TGK polymerase ... 94

2.15.2 PEX with $rA^{E}TP$, $rU^{Bio}TP$, $rC^{Ph}TP$, $rG^{Pent}TP$ and 5´-(TINA)-templ_31nt using TGK polymerase ... 94

2.15.3 PEX with $rA^{E}TP$, $rU^{Bio}TP$, $rC^{Ph}TP$, $rG^{Pent}TP$ and templ_65nt using TGK polymerase. ... 94

2.15.4 PEX with $rA^{E}TP$, $rU^{Bio}TP$, $rC^{Ph}TP$, $rG^{Pent}TP$ and templ_98nt using TGK polymerase. ... 94

2.15.5 PEX with $rA^{E}TP$, $rU^{Bio}TP$, $rC^{Ph}TP$, $rG^{Pent}TP$ and templ_98nt for RT analysis ... 94

2.15.6 RT analysis of natural or base-modified RNA ... 95

2.16 Comparison of TGK polymerase, SFM4-3 polymerase and T7 RNAP in enzymatic synthesis of RNA ... 97

2.16.1 Analysis of PEX reaction with RNA primer and various amounts of TGK or SFM4-3 polymerase ... 97

2.16.2 Kinetic studies of PEX reaction with RNA primer and TGK or SFM4-3 polymerase ... 98

2.16.3 Kinetic studies of PEX reaction with DNA primer and TGK or SFM4-3 polymerase ... 98

2.16.4 PEX with templ_poly-U and TGK or SFM4-3 polymerase ... 99

2.16.5 PEX reaction with templ_50nt and TGK or SFM4-3 polymerase ... 100

2.16.6 IVT reaction with ds-templ_poly-U and T7 RNAP ... 102

2.16.7 IVT reaction with ds-templ_52bp and T7 RNAP ... 103

2.16.8 Analysis of TGK or SFM4-3 polymerase fidelity with imbalanced nucleotide mixture. ... 106

2.17 Synthesis of base-modified RNA with cleavable DNA primer ... 107

2.17.1 Analytical PEX reaction with $rC^{Cy5}TP$ and fluorescently labelled DNA primer ... 107

2.17.2 Analytical PEX reaction with $rC^{Cy5}TP$ and fluorescently labelled DNA primer with internal dU modification ... 108

2.17.3 Analytical PEX reaction with $rA^{E}TP$, $rU^{Bio}TP$, $rC^{Cy5}TP$, $rG^{Pent}TP$ and fluorescently labelled DNA primer with internal dU modification ... 110

2.17.4 Analytical PEX reaction with $rA^{E}TP$, $rU^{Bio}TP$, $rC^{Ph}TP$, $rG^{Pent}TP$ and dual fluorescently labelled DNA primer with internal dU modification ... 113

2.17.5 Semi-preparative PEX reaction with $rC^{Cy5}TP$ and fluorescently labelled DNA primer with internal dU modification ... 114

2.17.6 Semi-preparative PEX reaction with $rA^{E}TP$, $rU^{Bio}TP$, $rC^{Cy5}TP$, $rG^{Pent}TP$ and fluorescently labelled DNA primer with internal dU modification ... 115

2.17.7 Semi-preparative PEX reaction with $rA^{E}TP$, $rU^{Bio}TP$, $rC^{Ph}TP$, $rG^{Pent}TP$ and dual fluorescently labelled DNA primer with internal dU modification ... 115

2.18 Selective fluorescent RNA labelling at specific position for structural studies ... 115

2.18.1 Analytical scale PEX reaction for preparation of FAM-Cy5-Cy3-riboswitch 115

2.18.2 Semi-preparative scale PEX reaction for preparation of FAM-Cy5-Cy3-riboswitch 117

2.18.3 Preparative scale PEX reaction for preparation of Cy5-Cy3-riboswitch 118

2.18.4 FRET measurements of the Cy5-Cy3-riboswitch 118

2.19 Enzymatic synthesis of mRNA 119

2.19.1 Preparation of ssDNA template – templ_IRES 119

2.19.2 Preparation of ssDNA template – templ_IRES-prolonged 120

2.19.3 Preparation of ssDNA template – templ_IRES-nLuc 121

2.19.4 Preparation of templ_(dual-Bio)-IRES-nLuc 122

2.19.5 Preparation of IRES-RNA 123

2.19.6 Synthesis of IRES-RNA_prolonged 124

2.19.7 Synthesis of natural mRNA (mRNA-nat) 124

2.19.8 Synthesis of fully modified mRNA (mRNA-full) 124

2.19.9 Synthesis of gene modified mRNA (mRNA-gene) 124

2.19.10 Synthesis of single-site modified mRNA (mRNA-init) 125

2.19.11 Synthesis of single-site modified mRNA (mRNA-mid) 125

2.19.12 Bisulfite conversion of mRNA with single modification 126

2.19.13 One step RT-PCR reaction of bisulfite-treated mRNA 126

2.19.14 Sample preparation for Sanger sequencing 127

2.19.15 PEX with natural rNTPs or a mixture of rA^{E}TP, rU^{E}TP, rC^{Me}TP, rG^{Pent}TP and templ_IRES, templ_IRES-prolonged, templ_IRES-nLuc using TGK polymerase 127

2.19.16 Reverse transcription of natural IRES-RNA and fully modified IRES-RNA_$A^{E}U^{E}C^{Me}G^{Pent}$ 128

2.19.17 Reverse transcription of fully modified IRES-RNA_prolonged_$A^{E}U^{E}C^{Me}G^{Pent}$ and mRNA_$A^{E}U^{E}C^{Me}G^{Pent}$ 128

2.19.18 qPCR amplification of cDNA from IRES-RNA or IRES-RNA_$A^{E}U^{E}C^{Me}G^{Pent}$ and gel analysis 129

2.19.19 qPCR amplification of cDNA from IRES-RNA_prolonged_$A^{E}U^{E}C^{Me}G^{Pent}$ or mRNA_$A^{E}U^{E}C^{Me}G^{Pent}$ and gel analysis 131

2.19.20 PEX reaction with natural rNTPs and templ_(dual-Bio)-IRES-nLuc immobilised on streptavidin magnetic beads (SMB) 134

2.19.21 PEX reaction with a mixture of rATP, rUTP, rC^{Me}TP, rGTP and templ_(dual-Bio)-IRES-nLuc immobilised on streptavidin magnetic beads (SMB) 135

2.20 mRNA translation studies 136

2.20.1 Protocol for *in vitro* translation in rabbit reticulocyte lysate system 136

2.20.2 Protocol for *in cellulo* translation in HEK293T cell system 136

2.21 Amplification PEX reaction with either all four natural rNTPs or a mixture rATP, rUTP, rC^{Me}TP, rGTP using a 98-nt long ssDNA template 136

3 Copies of NMR spectra 138

3.1 NMR spectra of nucleosides 138

3.1.1 Spectra of 7-(pent-1-yn-1-yl)-7-deazaadenosine (rA^{Pent}) 138

3.1.2 Spectra of 5-{3-[4-(5,5-difluoro-1,3,7,9-tetramethyl-5*H*-4λ^4,5λ^4-dipyrrolo[1,2-c:2',1'-f][1,3,2]diazaborinin-10-yl)-3,5-dimethylphenoxy]prop-1-yn-1-yl}-cytidine (rC^{mBdp}) 139

3.1.3 Spectra of 5-(pent-1-yn-1-yl)-5-cytidine (rC^{Pent}) 141

3.1.4 Spectra of 7-(pent-1-yn-1-yl)-7-deazaguanosine (rG^{Pent}) 142

3.2 NMR spectra of nucleotides 143

3.2.1 Spectra of 7-(5-formylthien-2-yl)-7-deazaadenosine-5'-*O*-triphosphate (rA^{FT}TP) ... 143

3.2.2 Spectra of 7-(pent-1-yn-1-yl)-7-deazaadenosine-5'-*O*-triphosphate (rA^{Pent}TP) 144

3.2.3 Spectra of 5-{3-[N-(2-chloroacetamido)]-prop-2-yn-1-yl}-uridine-5'-*O*-triphosphate (rU^{CA}TP) 146

3.2.4 Spectra of 5-(pent-1-yn-1-yl)-5-uridine-5'-*O*-triphosphate (rU^{Pent}TP) 147

3.2.5 5-{3-[N-(2-chloroacetamido)]-prop-2-yn-1-yl}-cytidine-5'-*O*-triphosphate (rC^{CA}TP) 149

3.2.6 5-(5-formylthien-2-yl)-cytidine-5'-*O*-triphosphate (rC^{FT}TP) 150

3.2.7 Spectra of 5-{3-[4-(5,5-difluoro-1,3,7,9-tetramethyl-5*H*-4λ^4,5λ^4-dipyrrolo[1,2-c:2',1'-f][1,3,2]diazaborinin-10-yl)-3,5-dimethylphenoxy]prop-1-yn-1-yl}-cytidine-5´-*O*-triphosphate (rC^{mBdp}TP) 152

3.2.8 Spectra of 5-(pent-1-yn-1-yl)-5-cytidine-5'-*O*-triphosphate (rC^{Pent}TP) 153

3.2.9 Spectra of 7-(pent-1-yn-1-yl)-7-deazaguanosine-5'-*O*-triphosphate (rG^{Pent}TP) 155

4 MS analysis 157

4.1 MS-MALDI-TOF spectra 157

4.2 LC-ESI-MS spectra 200

4.3 LC-MS chromatograms 225

5 Sanger sequencing 227

5.1 Raw Sanger sequencing data 227

6 Additional information 228

6.1 Plasmid sequence (5´→ 3´ direction) 228

6.2 mRNA coding plasmid construct 230

6.3 Overview of enzymatic syntheses with engineered DNA polymerases and T7 RNAP 231

7 Abbreviations and symbols used in this study 233
8 References 235
9 Source data files: uncropped scans of gels in Supplementary Figures 236

1 Synthetic part

1.1 General remarks

All solvents and reagents were purchased from commercial suppliers (Fluorochem, Sigma Aldrich, Lach-Ner) and used as received without further purification, unless otherwise specified. $POCl_3$ and $PO(OMe)_3$ were distilled prior to use. Reactions were performed in heat gun-dried glassware under inert argon atmosphere. Reactions were monitored by TLC Silica gel 60 F254 and detected by UV (254 nm) and by Advion Expression Compact Mass Spectrometer connected with Plate Express® TLC Plate Reader using electrospray ionization. NMR spectra were measured on a Bruker AVANCE III HD 500 spectrometer (^{1}H at 500.0 MHz, ^{11}B at 160.4 MHz, ^{13}C at 125.7 MHz, ^{19}F at 470.4 MHz and ^{31}P at 202.4 MHz) in D_2O (referenced to either to *tert*-butanol or dioxane) or DMSO-d_6 (referenced to $\delta(CHD_2SO_2CD_3)$ = 2.50 ppm and $\delta(CD_3SO_2CD_3)$ = 39.7 ppm) at 25°C. Chemical shifts are given in ppm (δ-scale) and coupling constants (J) are in Hz. Complete assignment of all NMR signals was achieved by using a combination of H,H-COSY, H,C-HSQC and H,C-HMBC experiments. Labelling of NMR signals assignments corresponds to a numbering depicted in compound formulas. High-resolution mass spectra – HR MS (ESI^{-}) were measured on LTQ Orbitrap XL (Thermo Fisher Scientific) and acquired by the MS service at IOCB. Column chromatography was performed using silica gel (40-63 µm, Fluorochem) by FLC Teledyne ISCO Combi Flash Rf 200 or 300. Purification of triphosphates was performed using HPLC (Waters modular HPLC system) on a Phenomenex Kinetex 5 µm EVO C18 100 Å, AXIA Packed LC column (250×21.2 mm) and POROS HQ 50 column (packed in-house, 26×120 mm). Purity of all final compounds was determined by NMR spectroscopy. Milli-Q water was used in the synthetic part. 5-Iodocytidine was purchased from Biosynth. 5-Formyl-2-thienylboronic acid was obtained from Sigma Aldrich.

1.2 General procedure for Sonogashira cross-coupling reaction on nucleosides

Anhydrous acetonitrile (2 mL) was added through septum to an argon-purged flask containing the iodinated nucleoside (**rN^{I}**, 1 equiv), $Pd(PPh_3)_4$ [10 mol.% or 30 mol% for (**rG^{I}**)[1]], CuI (10 mol.%) followed by addition of pent-1-yn (25 equiv) and TEA (10 equiv). Reaction mixture was stirred under argon atmosphere at 70 °C for 48 h. Resulting suspension was evaporated under reduced pressure, resuspended in methanol, co-evaporated with silica gel and purified by high performance reverse phase flash chromatography (0 to 100% MeOH in water) using C18RediSep column on ISCO.

Table S1. Yields (%) of Sonogashira cross-coupling reactions on iodinated nucleosides.

**rN^{I}**	**rN^{Pent}**	**Yield (%)**
(**rA^{I}**)[2]	**rA^{Pent}**	57
**rC^{I}**	**rC^{Pent}**	65
(**rG^{I}**)[1]	**rG^{Pent}**	61

1.3 General procedure for triphosphorylation reaction of pentyne-modified nucleosides (rN^{Pent})

The nucleoside (**rN^{Pent}**, 1 equiv) was dissolved in dry PO(OMe)$_3$ (1.0 mL) under argon atmosphere. Solution was cooled down to 0 ºC and afterwards POCl$_3$ (1.2 equiv) was added dropwise. After 2.5 h of stirring a solution of (n-Bu$_3$NH)$_2$H$_2$P$_2$O$_7$ (5.0 equiv) and n-Bu$_3$N (4.0 equiv) in dry acetonitrile (1.0 ml) was added. The mixture was stirred for another 2 h and then quenched by addition of TEAB (2 mL, 2 M). Crude mixture was concentrated under reduced pressure, co-evaporated with distilled water and purified by semi-preparative HPLC using C18 reversed phase column with a linear gradient from 0.1 M TEAB (aq.) to 0.1 M TEAB in 50% MeOH. Desired compound was isolated as a triethylammonium salt and converted to sodium salt using Dowex 50WX8 in Na$^+$ cycle. Solvent was evaporated, pure product was again dissolved in small amount of H$_2$O and freeze-dried overnight.

Table S2. Yields (%) of triphosphorylation reaction of modified nucleosides.

rN^{Pent}	rN^{Pent}TP	Yield (%)
**rA^{Pent}**	**rA^{Pent}TP**	41
(**rU^{Pent}**)[3]	**rU^{Pent}TP**	12
**rC^{Pent}**	**rC^{Pent}TP**	15
**rG^{Pent}**	**rG^{Pent}TP**	18

1.4 Chemical synthesis of previously published nucleosides and nucleotides

1.4.1 Chemical synthesis of rU^{Pent}

Synthesis was performed according to published literature[3].

1.4.2 Chemical synthesis of rA^{CA}TP

Synthesis was performed according to published literature[4].

1.4.3 Chemical synthesis of rU^{FT}TP

Synthesis was performed according to published literature[5].

1.4.4 Chemical synthesis of rA^{E}TP, A^{Ph}TP, rU^{E}TP, rU^{Ph}TP, rC^{E}TP, rC^{Ph}TP, rG^{E}TP, rG^{Ph}TP

Synthesis of these compounds was performed according to published literature[6].

1.5 Chemical synthesis of novel nucleosides

1.5.1 Chemical synthesis of 7-(pent-1-yn-1-yl)-7-deazaadenosine (rA^{Pent})

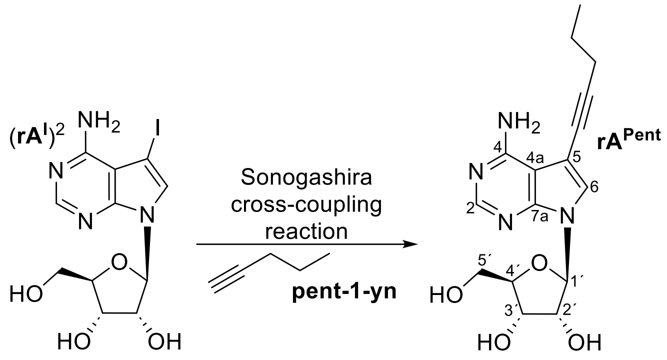

Compound **rA^{Pent}** was prepared from (**rA^{I}**)[2] according to standard procedure in section 1.2. Final product was isolated as a white solid. For yield of the reaction see Table S1. For NMR spectra see section 3.1.1.

^{1}H NMR (500.0 MHz, DMSO-*d*$_6$): 1.00 (t, 3H, J_{vic} = 7.4, C**H$_3$**CH$_2$CH$_2$); 1.57 (qt, 2H, J_{vic} = 7.4, 7.0, CH$_3$C**H$_2$**CH$_2$); 2.45 (t, 2H, J_{vic} = 7.0, CH$_3$CH$_2$C**H$_2$**); 3.53 (ddd, 1H, J_{gem} = 11.9, $J_{5'b,OH}$ = 6.2, $J_{5'b,4'}$ = 3.8, H-5′b); 3.62 (ddd, 1H, J_{gem} = 11.9, $J_{5'a,4'}$ = 5.0, $J_{5'a,4'}$ = 3.8, H-5′a); 3.88 (td, 1H, $J_{4',5'}$ = 3.8, $J_{4',3'}$ = 3.2, H-4′); 4.07 (ddd, 1H, $J_{3',2'}$ = 5.0, $J_{3',OH}$ = 4.7, $J_{3',4'}$ = 3.2, H-3′); 4.36 (td, 1H, $J_{2',1'}$ = $J_{2',OH}$ = 6.2, $J_{2',3'}$ = 5.0, H-2′); 5.12 (d, 1H, $J_{OH,3'}$ = 4.7, OH-3′); 5.20 (dd, 1H, $J_{OH,5'}$ = 6.2, 5.0, OH-5′); 5.32 (d, 1H, $J_{OH,2'}$ = 6.2, OH-2′); 6.00 (d, 1H, $J_{1',2'}$ = 6.2, H-1′); 7.66 (s, 1H, H-6); 8.10 (s, 1H, H-2).

^{13}C NMR (125.7 MHz, DMSO-*d*$_6$): 13.63 (**C**H$_3$CH$_2$CH$_2$); 21.05 (CH$_3$CH$_2$**C**H$_2$); 21.90 (CH$_3$**C**H$_2$CH$_2$); 61.74 (CH$_2$-5′); 70.73 (CH-3′); 73.94 (deazaA-**C**≡C-*n*Pr); 74.12 (CH-2′); 85.39 (CH-4′); 87.26 (CH-1′); 92.62 (deazaA-C≡**C**-*n*Pr); 95.65 (C-5); 102.60 (C-4a); 126.06 (CH-6); 149.60 (C-7a); 152.75 (CH-2); 157.79 (C-4).

HR MS (ESI^{-}): $C_{16}H_{21}O_4N_4$ calculated: 333.15573, found: 333.15569; **$C_{16}H_{20}O_4N_4Na$** calculated: 355.13768, found: 355.13763.

1.5.2 Chemical synthesis of 5-{3-[4-(5,5-difluoro-1,3,7,9-tetramethyl-5*H*-4λ^4,5λ^4-dipyrrolo[1,2-c:2',1'-f][1,3,2]diazaborinin-10-yl)-3,5-dimethylphenoxy]prop-1-yn-1-yl}-cytidine (rC^{mBdp})

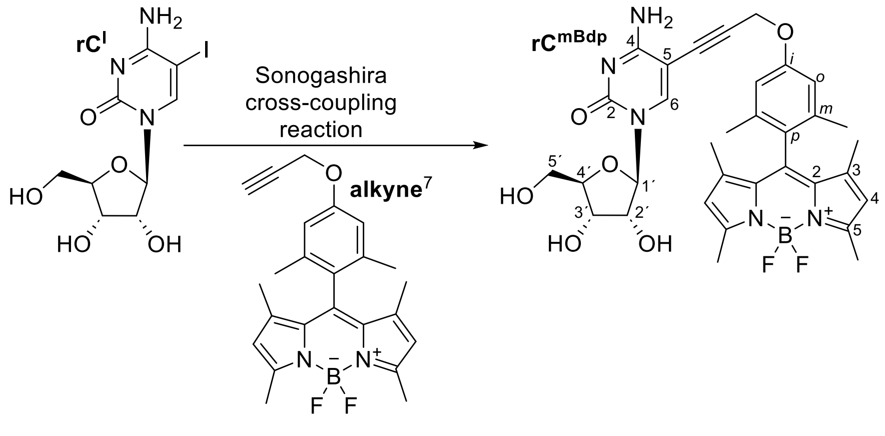

Nucleoside (**rC^{I}**, 1 equiv) was dissolved in dry DMF (1.2 mL) with the corresponding alkyne[7] (1.2 equiv), $PdCl_2(PPh_3)_2$ (5 mol%), CuI (5 mol%) under argon atmosphere. After addition of Et_3N (45 µl) the mixture was stirred at room temperature for 3 h. Crude reaction mixture was concentrated under reduced pressure and purified by silica gel column chromatography with a gradient of MeOH in CH_2Cl_2 (5% - 20%). The final nucleoside (**rC^{mBdp}**) was isolated as an orange solid in 67% yield. For NMR spectra see section 3.1.2.

^{1}**H NMR (500.0 MHz, DMSO-*d*$_6$):** 1.35 (s, 6H, CH_3-3-pyrr); 2.03 (s, 6H, CH_3-*m*-Ph); 2.45 (s, 6H, CH_3-5-pyrr); 3.55, 3.68 (2 × ddd, 2 × 1H, J_{gem} = 12.0, $J_{5',OH}$ = 5.0, $J_{5',4'}$ = 2.9, H-5'); 3.83 (dt, 1H, $J_{4',3'}$ = 5.4, $J_{4',5'}$ = 2.9, H-4'); 3.91 – 3.96 (m, 2H, H-2′,3'); 5.01 (d, 1H, $J_{OH,3'}$ = 4.9, OH-3′); 5.02 (s, 2H, CH_2O); 5.19 (t, 1H, $J_{OH,5'}$ = 5.0, OH-5′); 5.39 (d, 1H, $J_{OH,2'}$ = 4.4, OH-2′); 5.75 (d, 1H, $J_{1',2'}$ = 3.4, H-1'); 6.16 (s, 2H, H-4-pyrr); 6.94 (bs, 1H, $NH_a\mathbf{H_b}$); 6.97 (m, 2H, H-*o*-Ph); 7.84 (bs, 1H, $N\mathbf{H_a}H_b$); 8.32 (s, 1H, H-6).
^{13}**C NMR (125.7 MHz, DMSO-*d*$_6$):** 13.17 (CH_3-3-pyrr); 14.50 (CH_3-5-pyrr); 19.47 (CH_3-*m*-Ph); 56.72 (CH_2O); 60.25 (CH_2-5'); 69.14 (CH-3'); 74.55 (CH-2'); 79.40 (-**C**≡C-CH_2-); 84.35 (CH-4'); 88.90 (C-5); 89.59 (CH-1'); 90.52 (-C≡**C**-CH_2-); 114.86 (CH-*o*-Ph); 121.32 (CH-4-pyrr); 126.33 (C-*p*-Ph); 130.40 (C-2-pyrr); 136.21 (C-*m*-Ph); 141.09 (C-pyrr); 142.15 (C-3-pyrr); 145.84 (CH-6); 153.82 (C-2); 154.91 (C-5-pyrr); 157.76 (C-*i*-Ph); 164.59 (C-4).
^{19}**F NMR (470.4 MHz, DMSO-*d*$_6$):** -139.62 (q, $J_{F,B}$ = 32.2).
^{11}**B NMR (160.4 MHz, DMSO-*d*$_6$):** 0.60 (t, $J_{B,F}$ = 32.2).
HR MS (ESI$^-$): $C_{33}H_{37}O_6N_5BF_2$ calculated: 648.27995, found: 648.28045; **$C_{33}H_{36}O_6N_5BF_2Na$** calculated: 670.26189, found: 670.26241.

1.5.3 Chemical synthesis of 5-(pent-1-yn-1-yl)-cytidine (rC^{Pent})

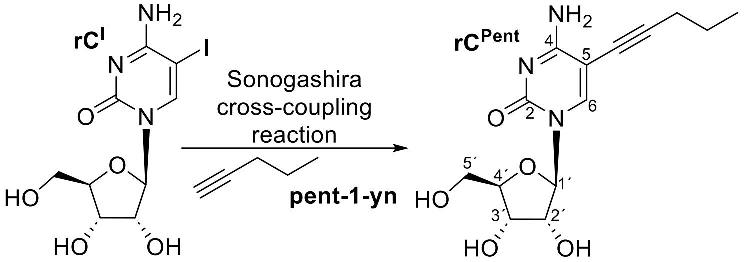

Compound **rC^{Pent}** was prepared from **rC^{I}** according to standard procedure in section 1.2. Final product was isolated as a white solid. For yield of the reaction see Table S1. For NMR spectra see section 3.1.3.

^{1}**H NMR (500.0 MHz, DMSO-*d*$_6$):** 0.96 (t, 3H, J_{vic} = 7.4, $C\mathbf{H_3}CH_2CH_2$); 1.54 (qt, 2H, J_{vic} = 7.4, 7.1, $CH_3C\mathbf{H_2}CH_2$); 2.37 (t, 2H, J_{vic} = 7.1, $CH_3CH_2C\mathbf{H_2}$); 3.56, 3.69 (2 × dd, 2 × 1H, J_{gem} = 12.1, $J_{5',4'}$ = 2.8, H-5′); 3.84 (dt, 1H, $J_{4',3'}$ = 5.4, $J_{4',5'}$ = 2.8, H-4′); 3.91 – 3.98 (m, 2H, H-2′,3′); 5.03, 5.20, 5.36 (3 × bs, 3 × 1H, OH-2′,3′,5′); 5.74 (d, 1H, $J_{1',2'}$ = 3.3, H-1′); 6.99, 7.81 (2 × bs, 2 × 1H, NH_2); 8.23 (s, 1H, H-6).
^{13}**C NMR (125.7 MHz, DMSO-*d*$_6$):** 13.71 ($\mathbf{C}H_3CH_2CH_2$); 21.24 ($CH_3CH_2\mathbf{C}H_2$); 21.72 ($CH_3\mathbf{C}H_2CH_2$); 60.16 (CH_2-5′); 69.07 (CH-3′); 71.91 (U-**C**≡C-*n*Pr); 74.41 (CH-2′); 84.30 (CH-4′); 89.55 (CH-1′); 90.76 (C-5); 96.07 (U-C≡**C**-*n*Pr); 144.55 (CH-6); 153.00 (C-2); 163.85 (C-4).
HR MS (ESI$^-$): $C_{14}H_{20}O_5N_3$ calculated: 310.13975, found: 310.13965; **$C_{14}H_{19}O_5N_3Na$** calculated: 332.12169, found: 332.12147.

1.5.4 Chemical synthesis of 7-(pent-1-yn-1-yl)-7-deazaguanosine (rG^{Pent})

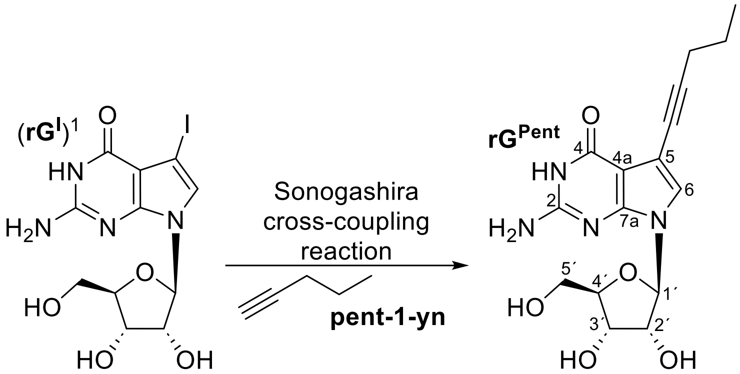

Compound **rG^{Pent}** was prepared from (**rG^{I}**)[1] according to standard procedure in section 1.2. Final product was isolated as a white solid. For yield of the reaction see Table S1. For NMR spectra see section 3.1.4.

^{1}H NMR (500.0 MHz, DMSO-*d*$_6$): 0.99 (t, 3H, J_{vic} = 7.4, C**H$_3$**CH$_2$CH$_2$); 1.52 (qt, 2H, J_{vic} = 7.4, 7.0, CH$_3$C**H$_2$**CH$_2$); 2.34 (t, 2H, J_{vic} = 7.0, CH$_3$CH$_2$C**H$_2$**); 3.49, 3.56 (2 × bdd, 2 × 1H, J_{gem} = 11.9, $J_{5',OH}$ = 5.4, $J_{5',4'}$ = 4.2, H-5′); 3.79 (td, 1H, $J_{4',5'}$ = 4.2, $J_{4',3'}$ = 3.4, H-4′); 4.01 (bdd, 1H, $J_{3',2'}$ = 5.2, $J_{3',4'}$ = 3.4, H-3′); 4.21 (bdd, 1H, $J_{2',1'}$ = 6.3, $J_{2',3'}$ = 5.2, H-2′); 4.97 (bt, 1H, $J_{OH,5'}$ = 5.4, OH-5′); 5.03 (bs, 1H, OH-3′); 5.24 (bs, 1H, OH-2′); 5.84 (d, 1H, $J_{1',2'}$ = 6.3, H-1′); 6.29 (bs, 2H, NH$_2$); 7.15 (s, 1H, H-6); 10.40 (bs, 1H, NH).

^{13}C NMR (125.7 MHz, DMSO-*d*$_6$): 13.61 (**C**H$_3$CH$_2$CH$_2$); 21.18 (CH$_3$CH$_2$**C**H$_2$); 22.10 (CH$_3$**C**H$_2$CH$_2$); 61.71 (CH$_2$-5′); 70.69 (CH-3′); 73.89 (CH-2′); 74.89 (deazaG-**C**≡C-*n*Pr); 84.88 (CH-4′); 85.95 (CH-1′); 89.89 (deazaG-C≡**C**-*n*Pr); 99.64, 99.71 (C-4a,5); 121.55 (CH-6); 150.95 (C-7a); 153.20 (CH-2); 158.09 (C-4).

HR MS (ESI$^-$): C$_{16}$H$_{21}$O$_5$N$_4$ calculated: 349.15065, found: 349.15069; **C$_{16}$H$_{20}$O$_5$N$_4$Na** calculated: 371.13259, found: 371.13280.

1.6 Chemical synthesis of novel nucleotides

1.6.1 Chemical synthesis of 7-(5-formylthien-2-yl)-7-deazaadenosine-5'-*O*-triphosphate (rA^{FT}TP)

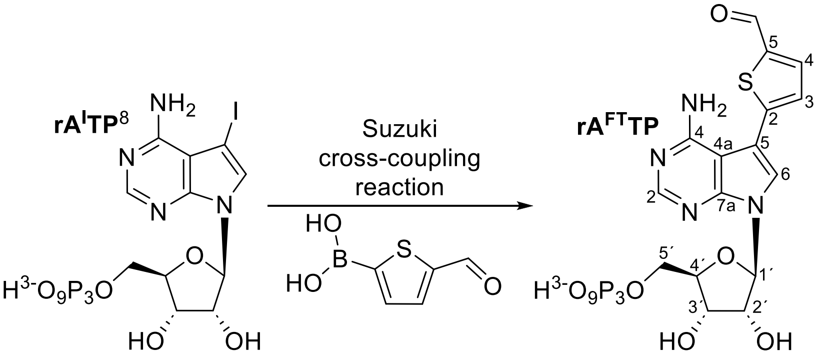

Triphosphate (**rA^{I}TP**[8], 1 equiv), 5-formylthiophene-2-boronic acid (1.8 equiv), Cs_2CO_3 (3 equiv), $Pd(OAc)_2$ (5 mol%) and TPPTS (25 mol%) were dissolved in a mixture of H_2O/acetonitrile (2:1, 1.5 mL) under argon atmosphere. The mixture was stirred at 100 °C for 1 h. Afterwards, solvents were evaporated under reduced pressure and the residue was again dissolved in H_2O prior to purification. The HPLC separation was performed on C18 reversed phase column using linear gradient from 0.1 M TEAB (aq.) to 0.1 M TEAB in 50% MeOH followed by several co-evaporations with H_2O. The pure product was isolated as a triethylammonium salt and converted to sodium salt using Dowex 50WX8 in Na$^+$ cycle. The solvent was evaporated, pure product was again dissolved in small amount of H_2O and freeze-

dried overnight. The desired product **rA^{FT}TP** (as sodium salt) was obtained as a yellow solid powder in 59% yield. For NMR spectra see section 3.2.1.

^{1}H NMR (500 MHz, D$_2$O, ref(*t*BuOH) = 1.25 ppm): 4.16 (ddd, 1H, J_{gem} = 11.7, $J_{5'a,P}$ = 4.9, $J_{5'a,4'}$ = 3.5, H-5´a); 4.26 (ddd, 1H, J_{gem} = 11.7, $J_{5'b,P}$ = 6.7, $J_{5'b,4'}$ = 3.4, H-5´b); 4.35 (qd, 1H, $J_{4',3'}$ = $J_{4',5'a}$ = $J_{4',5'b}$ = 3.2, $J_{4',P}$ = 2.0, H-4´); 4.58 (dd, 1H, $J_{3',2'}$ = 5.4, $J_{3',4'}$ = 2.9, H-3´); 4.72 (dd, 1H, $J_{2',1'}$ = 6.8, $J_{2',3'}$ = 5.4, H-2´); 6.25 (d, 1H, $J_{1',2'}$ = 6.8, H-1´); 7.28 (d, 1H, $J_{3,4}$ = 3.9, H-3-thienyl); 7.77 (s, 1H, H-6); 7.96 (d, 1H, $J_{4,3}$ = 3.9, H-4-thienyl); 8.13 (s, 1H, H-2); 9.74 (s, 1H, CHO).
^{13}C NMR (125.7 MHz, D$_2$O, ref(*t*BuOH) = 31.60 ppm): 67.44 (d, $J_{C,P}$ = 5.7, CH$_2$-5´); 72.45 (CH-3´); 75.65 (CH-2´); 85.71 (d, $J_{C,P}$ = 8.9, CH-4´); 87.79 (CH-1´); 102.46 (C-4a); 112.20 (C-5); 124.66 (CH-6); 130.26 (CH-3-thienyl); 142.56 (CH-4-thienyl); 143.56 (C-5-thienyl); 148.69 (C-2-thienyl); 152.71 (C-7a); 153.80 (CH-2); 158.84 (C-4); 188.49 (CHO).
^{31}P NMR (202.4 MHz, D$_2$O): -21.49 (t, *J* =19.6, P$_\beta$); -10.46 (d, *J* = 19.6, P$_\alpha$); -7.10 (d, *J* = 19.6, P$_\gamma$).
HR MS (ESI): $C_{16}H_{18}O_{14}N_4P_3S$ calculated 614.97531, found 614.97546; **$C_{16}H_{17}O_{14}N_4P_3SNa$** calculated 636.95780, found 636.95772; **$C_{16}H_{16}O_{14}N_4P_3SNa_2$** calculated 658.93974, found 658.93890.

1.6.2 Chemical synthesis of 7-(pent-1-yn-1-yl)-7-deazaadenosine-5´-*O*-triphosphate (rA^{Pent}TP)

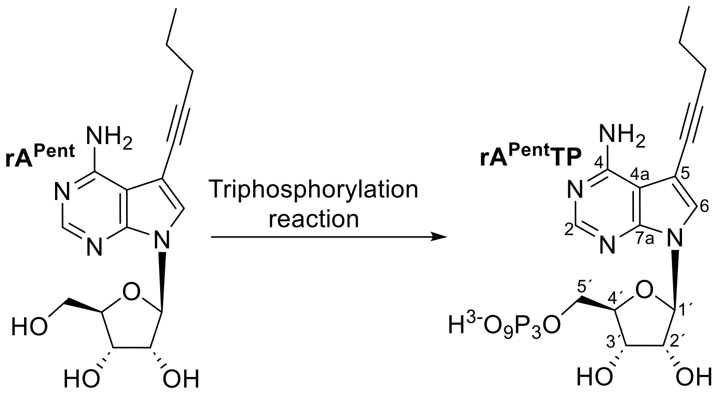

Compound **rA^{Pent}TP** was prepared from **rA^{Pent}** according to standard procedure in section 1.3. Final product was isolated as a white solid powder. For yield of the reaction see Table S2. For NMR spectra see section 3.2.2.

^{1}H NMR (500.0 MHz, D$_2$O, ref(*t*BuOH) = 1.24 ppm): 1.03 (t, 3H, J_{vic} = 7.4, C**H$_3$**CH$_2$CH$_2$); 1.64 (qt, 2H, J_{vic} = 7.4, 7.1, CH$_3$C**H$_2$**CH$_2$); 2.46 (t, 2H, J_{vic} = 7.1, CH$_3$CH$_2$C**H$_2$**); 4.12 (ddd, 1H, J_{gem} = 11.7, $J_{H,P}$ = 4.7, $J_{5'b,4'}$ = 3.2, H-5′b); 4.27 (ddd, 1H, J_{gem} = 11.7, $J_{H,P}$ = 6.8, $J_{5'a,4'}$ = 3.0, H-5′a); 4.34 (dddd, 1H, $J_{4',5'}$ = 3.2, 3.0, $J_{4',3'}$ = 2.7, $J_{H,P}$ = 1.7, H-4′); 4.58 (dd, 1H, $J_{3',2'}$ = 5.4, $J_{3',4'}$ = 2.7, H-3′); 4.68 (dd, 1H, $J_{2',1'}$ = 7.0, $J_{2',3'}$ = 5.4, H-2′); 6.22 (d, 1H, $J_{1',2'}$ = 7.0, H-1′); 7.68 (s, 1H, H-6); 8.16 (s, 1H, H-2).
^{13}C NMR (125.7 MHz, D$_2$O, ref(*t*BuOH) = 32.43 ppm): 15.78 (**C**H$_3$CH$_2$CH$_2$); 23.67 (CH$_3$CH$_2$**C**H$_2$); 24.38 (CH$_3$**C**H$_2$CH$_2$); 68.21 (d, $J_{C,P}$ = 5.6, CH$_2$-5′); 73.29 (CH-3′); 75.48 (deazaA-**C**≡C-*n*Pr); 76.43 (CH-2′); 86.70 (d, $J_{C,P}$ = 8.8, CH-4′); 88.36 (CH-1′); 97.65 (deazaA-C≡**C**-*n*Pr); 100.57 (C-5); 106.08 (C-4a); 128.00 (CH-6); 152.21 (C-7a); 155.35 (CH-2); 160.65 (C-4).

^{31}**P NMR (202.4 MHz, D$_2$O):** -20.45 (dd, J = 19.9, 19.4, Pβ); -9.68 (d, J = 19.4, Pα); -4.56 (d, J = 19.9, Pγ).
HR MS (ESI$^-$): $C_{16}H_{22}O_{13}N_4P_3$ calculated: 571.04017, found: 571.03961; **$C_{16}H_{21}O_{13}N_4P_3Na$** calculated: 593.02211, found: 593.02148; **$C_{16}H_{20}O_{13}N_4P_3Na_2$** calculated: 615.00406, found: 615.00330.

1.6.3 Chemical synthesis of 5-{3-[N-(2-chloroacetamido)]-prop-2-yn-1-yl}-uridine-5'-*O*-triphosphate (rU^{CA}TP)

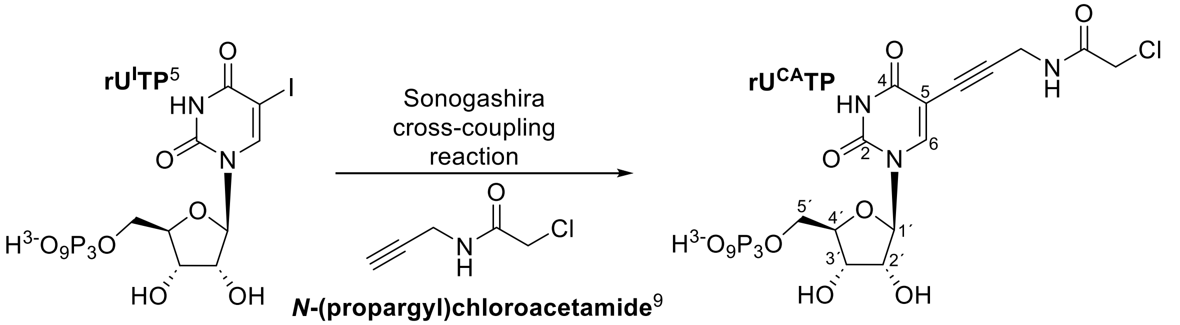

Triphosphate (**rU^{I}TP**[5], 1 equiv), *N*-(propargyl)chloroacetamide[9] (3 equiv), CuI (10 mol%), Pd(OAc)$_2$ (5 mol%) and TPPTS (25 mol%) were dissolved in a mixture of water/acetonitrile (2:1, 1 mL) under argon atmosphere. After addition of DIPEA (10 eq.) the mixture was stirred at 65 °C for 1 h. Additional amount of Pd(OAc)$_2$ (5 mol%) was added and the reaction was stirred for another 2 h at 65 °C. Afterwards, solvents were removed under reduced pressure and the residue was dissolved in H$_2$O prior to purification. HPLC separation was performed using reverse phase column with gradient starting from 0.1 M TEAB (aq.) up to 0.1 M TEAB in 50% MeOH. The second purification was performed on POROS HQ 50 column using linear gradient from H$_2$O to 400 mM TEAB (aq.) followed by several co-evaporations with H$_2$O. The pure product was isolated as a triethylammonium salt and converted to sodium salt using Dowex 50WX8 in Na$^+$ cycle. The solvent was evaporated, pure product was again dissolved in small amount of H$_2$O and freeze-dried overnight. The desired product **rU^{CA}TP** (as sodium salt) was obtained as a white solid powder in 23% yield. For NMR spectra see section 3.2.3.

^{1}**H NMR (500 MHz, D$_2$O, ref(*t*BuOH) = 1.25 ppm):** 4.20 (s, 2H, CH$_2$Cl); 4.23 – 4.34 (m, 5H, H-4´,5´,C≡CC**H$_2$**); 4.39 (dd, 1H, $J_{2',3'}$ = 5.1, $J_{2',1'}$ = 4.7, H-2´); 4.47 (bt, 1H, $J_{3',4'}$ = $J_{3',2'}$ = 5.0, H-3´); 5.96 (d, 1H, $J_{1',2'}$ = 4.7, H-1´); 8.24 (s, 1H, H-6).
^{13}**C NMR (125.7 MHz, D$_2$O, ref(*t*BuOH) = 31.60 ppm):** 31.13 (C≡C**C**H$_2$); 44.26 (CH$_2$Cl); 66.63 (d, $J_{C,P}$ = 5.3, CH$_2$-5´); 71.04 (CH-3´); 75.95 (**C**≡CCH$_2$); 76.01 (CH-2´); 85.32 (d, $J_{C,P}$ = 9.2, CH-4´); 90.81 (CH-1´); 91.23 (C≡**C**CH$_2$); 101.20 (C-5); 146.83 (CH-6); 152.9 (C-2); 166.8 (C-4); 171.59 (NHCO).
^{31}**P NMR (202.4 MHz, D$_2$O):** -21.72 (t, J = 19.6, P$_\beta$); -10.71 (d, J = 20.0, P$_\alpha$); -6.58 (bs, 1P, P$_\gamma$).

HR MS (ESI⁻): $C_{14}H_{18}O_{16}N_3P_3(^{35}Cl)$ calculated: 611.95939, found: 611.95880; **$C_{14}H_{17}O_{16}N_3P_3(^{35}Cl)Na$** calculated: 633.94134, found: 633.94089; **$C_{14}H_{16}O_{16}N_3P_3(^{35}Cl)Na_2$** calculated: 655.92328, found: 655.92267.

1.6.4 Chemical synthesis of 5-(pent-1-yn-1-yl)-uridine-5´-*O*-triphosphate (rU^{Pent}TP)

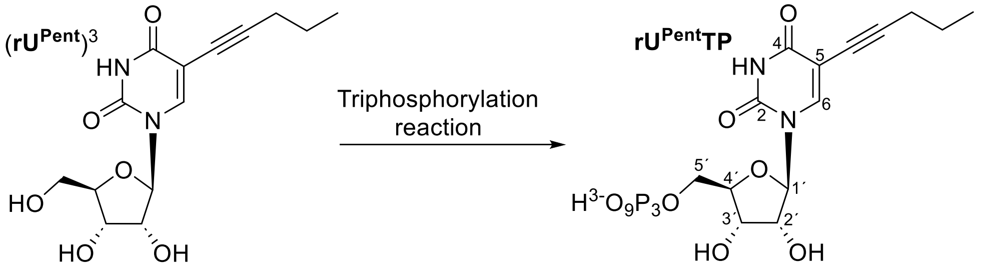

Compound **rU^{Pent}TP** was prepared from (**rU^{Pent}**)^3 according to standard procedure in section 1.3. Final product was isolated as a white solid powder. For yield of the reaction see Table S2. For NMR spectra see section 3.2.4.

^{1}H NMR (500.0 MHz, D_2O, ref(*t*BuOH) = 1.24 ppm): 0.99 (t, 3H, J_{vic} = 7.4, C**H$_3$**CH$_2$CH$_2$); 1.60 (qt, 2H, J_{vic} = 7.4, 7.1, CH$_3$C**H$_2$**CH$_2$); 2.42 (t, 2H, J_{vic} = 7.1, CH$_3$CH$_2$C**H$_2$**); 4.24 – 4.29 (m, 3H, H-4′,5′); 4.32 (dd, 1H, $J_{2',3'}$ = 5.2, $J_{2',1'}$ = 4.6, H-2′); 4.39 (t, 1H, $J_{3',2'}$ = $J_{3',4'}$ = 5.2, H-3′); 5.97 (d, 1H, $J_{1',2'}$ = 4.6, H-1′); 8.04 (s, 1H, H-6).

^{13}C NMR (125.7 MHz, D_2O, ref(*t*BuOH) = 32.43 ppm): 15.82 (**C**H$_3$CH$_2$CH$_2$); 23.68 (CH$_3$CH$_2$**C**H$_2$); 24.28 (CH$_3$**C**H$_2$CH$_2$); 67.66 (d, $J_{C,P}$ = 5.3, CH$_2$-5′); 71.96 (CH-3′); 73.09 (U-**C**≡C-*n*Pr); 76.88 (CH-2′); 85.64 (d, $J_{C,P}$ = 9.0, CH-4′); 92.18 (CH-1′); 96.63 (C-5); 101.42 (U-C≡**C**-*n*Pr); 146.24 (CH-6); 159.19 (C-2); 168.14 (C-4).

^{31}P NMR (202.4 MHz, D_2O): -21.39 (t, J = 19.9, P$_\beta$); -9.92 (d, J = 19.9, P$_\alpha$); -7.73 (bs, P$_\gamma$).

HR MS (ESI⁻): $C_{14}H_{20}O_{15}N_2P_3$ calculated: 549.00820, found: 549.00794; **$C_{14}H_{19}O_{15}N_2P_3Na$** calculated: 570.99015, found: 570.98982.

1.6.5 Chemical synthesis of 5-{3-[N-(2-chloroacetamido)]-prop-2-yn-1-yl}-cytidine-5'-*O*-triphosphate (rC^{CA}TP)

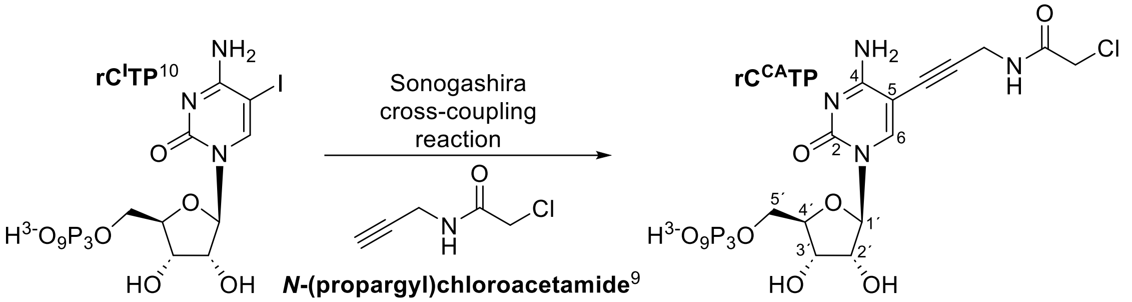

Triphosphate (**rC^{I}TP**[10], 1 equiv), *N*-(propargyl)chloroacetamide[9] (2.5 equiv), CuI (10 mol%), $Pd(OAc)_2$ (5 mol%) and TPPTS (20 mol%) were dissolved in a mixture of water/acetonitrile (2:1, 1 mL) under argon atmosphere. After addition of DIPEA (10 equiv) the mixture was stirred at 65 °C for 3 h. Afterwards, solvents were removed under reduced pressure and the residue was dissolved in H_2O prior to purification. HPLC separation was performed using reverse phase column with gradient starting from 0.1 M TEAB (aq.) up to 0.1 M TEAB in 50% MeOH. The second purification was performed on POROS HQ 50 column using linear gradient from H_2O to 400 mM TEAB (aq.) followed by several co-evaporations with H_2O. The pure product was isolated as a triethylammonium salt and converted to sodium salt using Dowex 50WX8 in Na^+ cycle. The solvent was evaporated, pure product was again dissolved in small amount of H_2O and freeze-dried overnight. The desired product **rC^{CA}TP** (as sodium salt) was obtained as a white solid powder in 34% yield. For NMR spectra see section 3.2.5.

^{1}H NMR (500 MHz, D_2O, ref(*t*BuOH) = 1.25 ppm): 4.20 (s, 2H, CH_2Cl); 4.25 – 4.35 (m, 5H, H-4′,5′,C≡CC**H$_2$**); 4.32 (dd, 1H, $J_{2',3'}$ = 5.2, $J_{2',1'}$ = 3.7, H-2′); 4.42 (bdd, 1H, $J_{3',4'}$ = 5.9, $J_{3',2'}$ = 5.2, H-3′); 5.95 (d, 1H, $J_{1',2'}$ = 3.7, H-1′); 8.24 (s, 1H, H-6).

^{13}C NMR (125.7 MHz, D_2O, ref(*t*BuOH) = 31.6 ppm): 32.17 (C≡C**C**H$_2$); 44.23 (CH_2Cl); 66.33 (d, $J_{C,P}$ = 5.4, CH_2-5′); 70.54 (CH-3′); 75.55 (**C**≡CCH$_2$); 76.42 (CH-2′); 84.66 (d, $J_{C,P}$ = 9.1, CH-4′); 91.70 (CH-1′); 93.11 (C≡**C**CH$_2$); 94.14 (C-5); 147.06 (CH-6); 158.21 (C-2); 167.07 (C-4); 171.73 (NHCO).

^{31}P NMR (202.4 MHz, D_2O): -21.35 (t, *J* = 19.7, P_β); -10.52 (d, *J* = 19.7, P_α); -6.04 (bd, *J* = 19.7, P_γ).

HR MS (ESI$^-$): $C_{14}H_{17}O_9N_4P(^{35}Cl)$ calculated: 451.04272, found: 451.04224; **$C_{14}H_{18}O_{12}N_4P_2(^{35}Cl)$** calculated: 531.00850, found: 531.00835; **$C_{14}H_{17}O_{12}N_4P_2(^{35}Cl)Na$** calculated: 552.99044, found: 552.99013.

1.6.6 Chemical synthesis of 5-(5-formylthien-2-yl)-cytidine-5'-*O*-triphosphate (rC^{FT}TP)

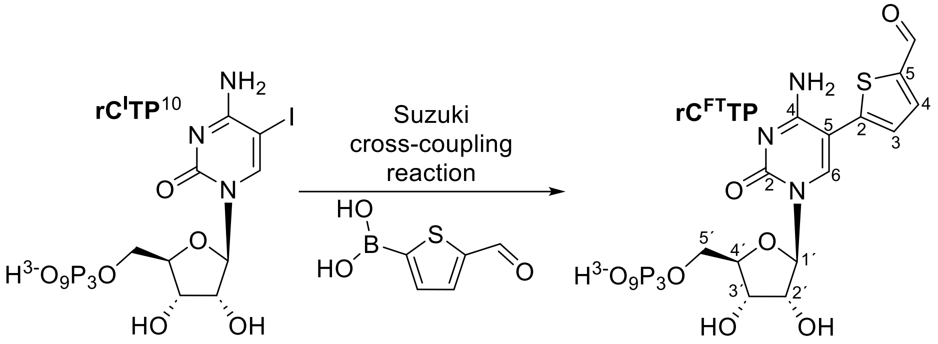

Triphosphate (rC^{I}TP[10], 1 equiv), 5-formylthiophene-2-boronic acid (1.8 equiv), Cs_2CO_3 (3 equiv), $Pd(OAc)_2$ (5 mol%) and TPPTS (25 mol%) were dissolved in a mixture of H_2O/acetonitrile (2:1, 1.5 mL) under argon atmosphere. The mixture was stirred at 100 °C for 1 h. Afterwards, solvents were evaporated under reduced pressure and the residue was again dissolved in H_2O prior to purification. The HPLC separation was performed on C18 reversed phase column using linear gradient from 0.1 M TEAB (aq.) to 0.1 M TEAB in 50% MeOH followed by several co-evaporations with H_2O. The pure product was isolated as a triethylammonium salt and converted to sodium salt using Dowex 50WX8 in Na^+ cycle. The solvent was evaporated, pure product was again dissolved in small amount of H_2O and freeze-dried overnight. The desired product **rC^{FT}TP** (as sodium salt) was obtained as a white solid powder in 28% yield. For NMR spectra see section 3.2.6.

^{1}H NMR (500 MHz, D_2O, ref(*t*BuOH) = 1.25 ppm): 4.20 – 4.32 (m, 3H, H-4´,5´); 4.39 – 4.46 (m, 2H, H-2´,3´); 6.01 (d, 1H, $J_{1',2'}$ = 4.3, H-1´); 7.43 (d, 1H, $J_{3,4}$ = 3.9, H-3-thienyl); 8.03 (d, 1H, $J_{4,3}$ = 3.9, H-4-thienyl); 8.06 (s, 1H, H-6); 9.84 (s, 1H, CHO).
^{13}C NMR (125.7 MHz, D_2O, ref(*t*BuOH) = 31.60 ppm): 66.71 (d, $J_{C,P}$ = 5.5, CH_2-5´); 71.12 (CH-3´); 76.18 (CH-2´); 85.05 (d, $J_{C,P}$ = 9.1, CH-4´); 91.59 (CH-1´); 104.88 (C-5); 132.52 (CH-3-thienyl); 141.95 (CH-4-thienyl); 143.74 (CH-6); 145.14 (C-5-thienyl); 146.01 (C-2-thienyl); 158.91 (C-2); 165.97 (C-4); 188.89 (CHO).
^{31}P NMR (202.4 MHz, D_2O): -21.68 (t, *J* = 19.9, P_β); -10.84 (d, *J* = 19.9, P_α); -7.26 (m, P_γ).
HR MS (ESI): $C_{14}H_{17}O_{15}N_3P_3S$ calculated: 591.95987, found: 591.95897; **$C_{14}H_{16}O_{15}N_3P_3SNa$** calculated: 613.94181, found: 613.94141; **$C_{14}H_{15}O_{15}N_3P_3SNa_2$** calculated: 635.92376, found: 635.92274.

1.6.7 Chemical synthesis of 5-{3-[4-(5,5-difluoro-1,3,7,9-tetramethyl-5*H*-4λ[4],5λ[4]-dipyrrolo[1,2-c:2',1'-f][1,3,2]diazaborinin-10-yl)-3,5-dimethylphenoxy]prop-1-yn-1-yl}-cytidine-5´-*O*-triphosphate (rC^{mBdp}TP)

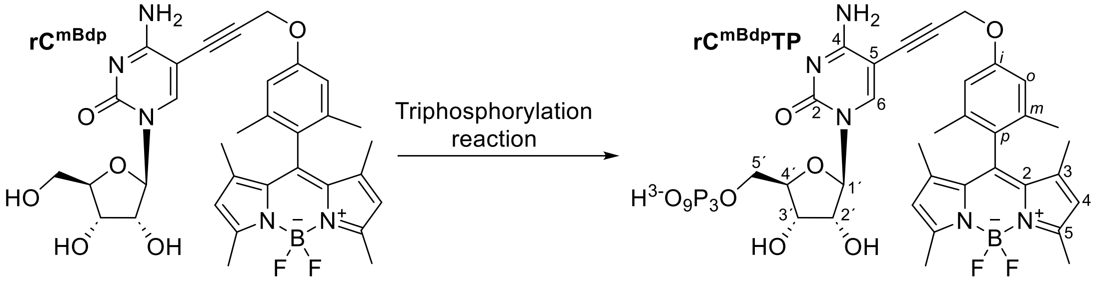

Nucleoside (**rC^{mBdp}**, 1 equiv) was dissolved in dry $PO(OMe)_3$ (0.55 mL) under argon atmosphere. The solution was cooled down to 0 °C and afterwards $POCl_3$ (2.4 equiv) was added dropwise. After 4 h of stirring a solution of (n-$Bu_3NH)_2H_2P_2O_7$ (4.2 equiv) and n-Bu_3N (4.2 equiv) in dry DMF (0.55 ml) was added. The mixture was stirred for another 1 h and then quenched by addition of TEAB (2 mL, 2 M). Crude mixture was concentrated under reduced pressure, co-evaporated with distilled water and purified by semi-preparative HPLC using C18 reversed phase column with a linear gradient from 0.1 M TEAB (aq.) to 0.1 M TEAB in 50% MeOH. Desired compound was isolated as a triethylammonium salt and converted to sodium salt using Dowex 50WX8 in Na^+ cycle. Solvent was evaporated, pure product was again dissolved in small amount of H_2O and freeze-dried overnight. Final product was isolated as an orange solid in 41% yield. For NMR spectra see section 3.2.7.

^{1}H NMR (500.0 MHz, D_2O, ref(dioxane) = 3.75 ppm): 1.36 (s, 6H, CH_3-3-pyrr); 2.05 (s, 6H, CH_3-*m*-Ph); 2.48 (s, 6H, CH_3-5-pyrr); 4.19 – 4.31 (bm, 4H, H-3′,4′,5'); 4.36 (bm, 1H, H-2′); 5.08 (bs, 2H, CH_2O); 5.90 (bd, 1H, $J_{1',2'}$ = 3.4, H-1'); 6.15 (bs, 2H, H-4-pyrr); 6.97 (bs, 2H, H-*o*-Ph); 8.18 (bs, 1H, H-6).

^{13}C NMR (125.7 MHz, D_2O, ref(dioxane) = 69.30 ppm): 15.35 (CH_3-3-pyrr); 16.48 (CH_3-5-pyrr); 21.37 (CH_3-*m*-Ph); 59.34 (CH_2O); 67.30 (d, $J_{C,P}$ = 4.5, CH_2-5'); 71.60 (CH-3'); 77.05 (CH-2'); 80.92 (-**C**≡C-CH_2-); 85.42 (d, $J_{C,P}$ = 8.4, CH-4'); 92.51 (CH-1'); 93.75 (-C≡**C**-CH_2-); 117.91 (CH-*o*-Ph); 123.97 (CH-4-pyrr); 129.85 (C-*p*-Ph); 133.38 (C-2-pyrr); 139.70 (C-*m*-Ph); 144.38 (C-pyrr); 146.89 (C-3-pyrr); 147.90 (CH-6); 158.47 (C-2); 158.77 (C-5-pyrr); 159.76 (C-*i*-Ph); 167.72 (C-4); (C-5 not detected).

^{31}P NMR (202.4 MHz, D_2O): -22.23 (bs, P_β); -10.80 (bs, P_α); -9.40 (bs, P_γ).

HR MS (ESI$^-$): $C_{33}H_{37}O_{15}N_5P_3BF_2$, z = 2 calculated: 442.57855, found: 442.57835; **$C_{33}H_{38}O_{15}N_5P_3BF_2$** calculated: 886.16438, found: 886.16328; **$C_{33}H_{37}O_{15}N_5P_3BF_2Na$** calculated: 908.14633, found: 908.14508.

1.6.8 Chemical synthesis of 5-(pent-1-yn-1-yl)-cytidine-5´-*O*-triphosphate (rC^{Pent}TP)

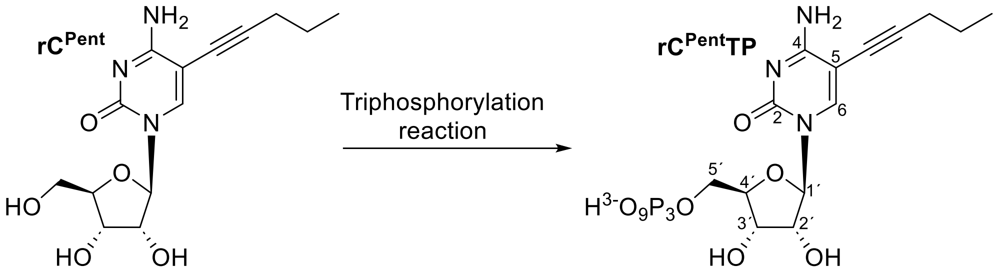

Compound **rC^{Pent}TP** was prepared from **rC^{Pent}** according to standard procedure in section 1.3. Final product was isolated as a white solid powder. For yield of the reaction see Table S2. For NMR spectra see section 3.2.8.

^{1}H NMR (500.0 MHz, D$_2$O, ref(*t*BuOH) = 1.24 ppm): 0.99 (t, 3H, J_{vic} = 7.4, C**H$_3$**CH$_2$CH$_2$); 1.60 (qt, 2H, J_{vic} = 7.4, 7.1, CH$_3$C**H$_2$**CH$_2$); 2.42 (t, 2H, J_{vic} = 7.1, CH$_3$CH$_2$C**H$_2$**); 4.24 – 4.29 (m, 3H, H-4′,5′); 4.32 (dd, 1H, $J_{2′,3′}$ = 5.2, $J_{2′,1′}$ = 4.6, H-2′); 4.39 (t, 1H, $J_{3′,2′}$ = $J_{3′,4′}$ = 5.2, H-3′); 5.97 (d, 1H, $J_{1′,2′}$ = 4.6, H-1′); 8.04 (s, 1H, H-6).
^{13}C NMR (125.7 MHz, D$_2$O, ref(*t*BuOH) = 32.43 ppm): 15.82 (**C**H$_3$CH$_2$CH$_2$); 23.68 (CH$_3$CH$_2$**C**H$_2$); 24.28 (CH$_3$**C**H$_2$CH$_2$); 67.66 (d, $J_{C,P}$ = 5.3, CH$_2$-5′); 71.96 (CH-3′); 73.09 (U-**C**≡C-*n*Pr); 76.88 (CH-2′); 85.64 (d, $J_{C,P}$ = 9.0, CH-4′); 92.18 (CH-1′); 96.63 (C-5); 101.42 (U-C≡**C**-*n*Pr); 146.24 (CH-6); 159.19 (C-2); 168.14 (C-4).
^{31}P NMR (202.4 MHz, D$_2$O): -21.39 (t, *J* = 19.9, P$_\beta$); -9.92 (d, *J* = 19.9, P$_\alpha$); -7.73 (bs, P$_\gamma$).
HR MS (ESI$^-$): C$_{14}$H$_{21}$O$_{14}$N$_3$P$_3$ calculated: 548.02418, found: 548.02368; **C$_{14}$H$_{20}$O$_{14}$N$_3$P$_3$Na** calculated: 570.00613, found: 570.00568; **C$_{14}$H$_{19}$O$_{14}$N$_3$P$_3$Na$_2$** calculated: 591.98807, found: 591.98743.

1.6.9 Chemical synthesis of 7-(pent-1-yn-1-yl)-7-deazaguanosine-5’-*O*-triphosphate (rG^{Pent}TP)

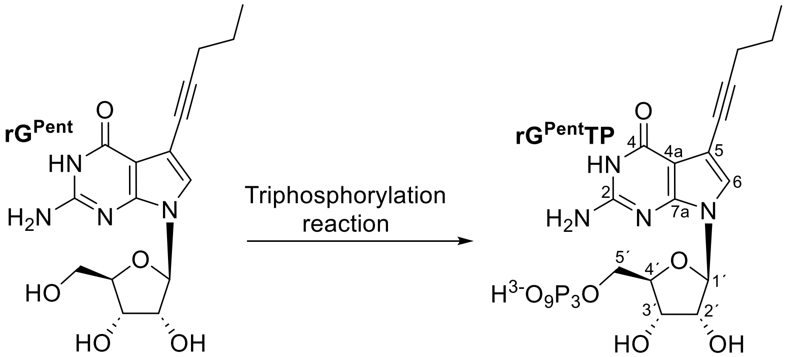

Compound **rG^{Pent}TP** was prepared from **rG^{Pent}** according to standard procedure in section 1.3. Final product was isolated as a white solid powder. For yield of the reaction see Table S2. For NMR spectra see section 3.2.9.

^{1}H NMR (500.0 MHz, D$_2$O, ref(*t*BuOH) = 1.24 ppm): 1.02 (t, 3H, J_{vic} = 7.4, C**H$_3$**CH$_2$CH$_2$); 1.60 (qt, 2H, J_{vic} = 7.4, 7.1, CH$_3$C**H$_2$**CH$_2$); 2.41 (t, 2H, J_{vic} = 7.0, CH$_3$CH$_2$C**H$_2$**); 4.13 (ddd, 1H, J_{gem} = 11.7, $J_{H,P}$ = 4.8, $J_{5′b,4′}$ = 3.4, H-5′b); 4.27 (ddd, 1H, J_{gem} = 11.7, $J_{H,P}$ = 6.8, $J_{5′a,4′}$ = 3.3, H-5′a); 4.29

(m, 1H, H-4′); 4.56 (dd, 1H, $J_{3',2'}$ = 5.3, $J_{3',4'}$ = 2.6, H-3′); 4.65 (dd, 1H, $J_{2',1'}$ = 7.1, $J_{2',3'}$ = 5.3, H-2′); 6.00 (d, 1H, $J_{1',2'}$ = 7.1, H-1′); 7.29 (s, 1H, H-6).

^{13}C NMR (125.7 MHz, D$_2$O, ref(*t*BuOH) = 32.43 ppm): 15.77 (**C**H$_3$CH$_2$CH$_2$); 23.72 (CH$_3$CH$_2$**C**H$_2$); 24.42 (CH$_3$**C**H$_2$CH$_2$); 68.19 (d, $J_{C,P}$ = 5.4, CH$_2$-5′); 73.22 (CH-3′); 75.81 (deazaG-**C**≡C-*n*Pr); 76.00 (CH-2′); 86.55 (d, $J_{C,P}$ = 8.8, CH-4′); 88.53 (CH-1′); 96.33 (deazaG-C≡**C**-*n*Pr); 102.85, 103.15 (C-4a,5); 125.39 (CH-6); 154.30 (C-7a); 156.24 (C-2); 163.99 (C-4).

^{31}P NMR (202.4 MHz, D$_2$O): -20.32 (bt, J = 19.4, P_β); -10.38 (d, J = 19.4, P_α); -5.43 (bd, J = 19.4, P_γ).

HR MS (ESI$^-$): $C_{16}H_{22}O_{14}N_4P_3$ calculated: 587.03508, found: 587.03557.

2 Biochemical part

2.1 General remarks

All PAGE and agarose gels were analysed by fluorescence and/or phosphor imaging using Typhoon FLA 9500 (GE Healthcare Life Sciences). Quantification from dPAGE gels was performed by ImageJ. Mass spectra of oligonucleotides were measured on UltrafleXtreme MALDI-TOF/TOF (Bruker) mass spectrometer with 1 kHz smartbeam II laser technology by MS service at IOCB. LC-ESI-MS spectra of oligonucleotides were acquired on Agilent 1290 Infinity II Bio system with DAD detector and mass spectrometer MSD XT. LC-ESI-MS analysis of oligonucleotides was carried out according to standard procedures using mobile phases A (12.2 mM Et_3N, 300 mM HFIP in H_2O) and B (12.2 mM Et_3N, 300 mM HFIP in H_2O in 100% MeOH) by 10 min gradient from 5% B to 100% B in A using ACQUITY Premier CSH C18 column 1.7 µm 2.1×150 mm (Waters) on Agilent UHPLC Bio system. Deconvolutions of LC-ESI-MS spectra were carried out using UniDec program[11]. Fluorescence spectra were measured in a 100 µL quartz cuvette at room temperature on a Fluoromax 4 spectrofluorometer (HORIBA Scientific). RNase/DNase free solutions for biochemical reactions were prepared using Milli-Q water, that was treated with DEPC and sterilized by autoclaving. Concentrations of the prepared RNA solutions were calculated using extinction coefficients obtained from on-line tool at https://www.atdbio.com/tools/oligo-calculator and A_{260} values measured on Nanodrop 1000 (Thermo Fischer Scientific), except of Cy5-labelled mRNA samples, that were quantified by fluorescence measurements against standard curve from Cy5-labelled oligonucleotides on Tecan Spark Multimode Reader. DNA oligonucleotides were purchased from Eurofins, Generi Biotech and Biomers. RNA oligonucleotides were obtained from Biomers. HiTrap Heparin HP, HisTrap FF and HisTrap HP affinity columns were purchased from GE Healthcare. Dynabeads MyOne streptavidin C1, Ribolock RNase inhibitor, TurboDNase, T7 RNA polymerase with the corresponding transcription buffer, RiboRuler High Range RNA ladder, RiboRuler Low Range RNA ladder, TriTrack DNA loading dye, GeneRuler 1 kb Plus DNA ladder, GeneRuler 1 kb DNA ladder, GeneRuler 100 bp DNA ladder ready-to-use, SybrGreen, Uracil-DNA Glycosylase, SuperScript™ IV reverse transcriptase, Lipofectamine MessengerMAX, OptiMEM medium were purchased from Thermo Fisher Scientific. Q5 High-Fidelity DNA polymerase, deoxyribonucleotide solution mix, ribonucleotide solution mix, ThermoPol buffer, nucleoside digestion mix, Proteinase K, Shrimp Alkaline Phosphatase, Monarch RNA cleanup kit, LunaScript Multiplex one-step RT-PCR kit, magnetic mRNA isolation kit, NEB 10-beta competent E. coli (high efficiency), Gel loading dye purple (6X) were bought from New England Biolabs. Microspin G-25 columns and GelRed were obtained from Sigma Aldrich. Protector RNase inhibitor was purchased from Roche. mRNA template encoding plasmid was designed and purchased from VectorBuilder. QIAquick nucleotide removal kit, QIAquick gel extraction kit, QIAquick PCR purification kit were bought from QIAGEN. EZ RNA methylation kit and RNA Clean & Concentrator-25 kit were purchased from Zymo Research. BL21(DE3) pLysS cells, rabbit reticulocyte lysate, amino acid mixture, Nano-Glo luciferase assay system were obtained from Promega. Aminoallyl-UTP-Cy3 (**rU^{Cy3}TP**), Aminoallyl-UTP-Cy5 (**rU^{Cy5}TP**), 5-

Propargylamino-CTP-Cy5 (**rC^{Cy5}TP**), Biotin-16-UTP (**rU^{Bio}TP**), Digoxigenin-11-UTP (**rU^{Dig}TP**), 5-Methyl-CTP (**rC^{Me}TP**) were purchased from Jena Bioscience. NucleoSpin gel and PCR clean-up midi kit was purchased from Macherey-Nagel.

2.2 List of oligonucleotide sequences

2.2.1 RNA oligonucleotides used in this study

Table S3. RNA oligonucleotides used as primers.

Name	Size (nt)	Modification	Sequence (5´→3´ direction)
**FAM-RNA-prim_15nt**	15	5´-FAM	CAUGGGCGGCAUGGG
**Cy5-RNA-prim_15nt**	15	5´-Cy5	CAUGGGCGGCAUGGG
**RNA-prim_15nt**	15	–	CAUGGGCGGCAUGGG
**Cy5-mRNA-prim**	23	5´-Cy5	GACUCACUAUAGGGCCCCUCUCC
**FAM-RNA-prim_23nt**	23	5´-FAM	GGGAAGAUAUAAUCCUAAUGAUA
**RNA-prim_23nt**	23	–	GGGAAGAUAUAAUCCUAAUGAUA
**Cy5-IRES-RNA**	606	5´-Cy5	GACUCACUAUAGGGCCCCUCUCCCUCCCCCCCCCCUAA<br>CGUUACUGGCCGAAGCCGCUUGGAAUAAGGCCGGUGUG<br>CGUUUGUCUAUAUGUUAUUUUCCACCAUAUUGCCGUCU<br>UUUGGCAAUGUGAGGGCCCGGAAACCUGGCCCUGUCUU<br>CUUGACGAGCAUUCCUAGGGGUCUUUCCCCUCUCGCCA<br>AAGGAAUGCAAGGUCUGUUGAAUGUCGUGAAGGAAGCA<br>GUUCCUCUGGAAGCUUCUUGAAGACAAACAACGUCUGU<br>AGCGACCCUUUGCAGGCAGCGGAACCCCCCACCUGGCG<br>ACAGGUGCCUCUGCGGCCAAAAGCCACGUGUAUAAGAU<br>ACACCUGCAAAGGCGGCACAACCCCAGUGCCACGUUGU<br>GAGUUGGAUAGUUGUGGAAAGAGUCAAAUGGCUCUCCU<br>CAAGCGUAUUCAACAAGGGGCUGAAGGAUGCCCAGAAG<br>GUACCCCAUUGUAUGGGAUCUGAUCUGGGGCCUCGGUG<br>CACAUGCUUUACAUGUGUUUAGUCGAGGUUAAAAAAAC<br>GUCUAGGCCCCCCGAACCACGGGGACGUGGUUUUCCUU<br>UGAAAAACACGAUGAUAAUAUGGCCACAACCAUGGU

**Cy5-IRES-RNA_prolonged**	804	Cy5	GACUCACUAUAGGGCCCCUCUCCCUCCCCCCCCCCUAA CGUUACUGGCCGAAGCCGCUUGGAAUAAGGCCGGUGUG CGUUUGUCUAUAUGUUAUUUUCCACCAUAUUGCCGUCU UUUGGCAAUGUGAGGGCCCGGAAACCUGGCCCUGUCUU CUUGACGAGCAUUCCUAGGGGUCUUUCCCCUCUCGCCA AAGGAAUGCAAGGUCUGUUGAAUGUCGUGAAGGAAGCA GUUCCUCUGGAAGCUUCUUGAAGACAAACAACGUCUGU AGCGACCCUUUGCAGGCAGCGGAACCCCCCACCUGGCG ACAGGUGCCUCUGCGGCCAAAAGCCACGUGUAUAAGAU ACACCUGCAAAGGCGGCACAACCCCAGUGCCACGUUGU GAGUUGGAUAGUUGUGGAAAGAGUCAAAUGGCUCUCCU CAAGCGUAUUCAACAAGGGGCUGAAGGAUGCCCAGAAG GUACCCCAUUGUAUGGGAUCUGAUCUGGGGCCUCGGUG CACAUGCUUUACAUGUGUUUAGUCGAGGUUAAAAAAAC GUCUAGGCCCCCCGAACCACGGGGACGUGGUUUUCCUU UGAAAAACACGAUGAUAAUAUGGCCACAACCAUGGUCU UCACACUCGAAGAUUUCGUUGGGGACUGGCGACAGACA GCCGGCUACAACCUGGACCAAGUCCUUGAACAGGGAGG UGUGUCCAGUUUGUUUCAGAAUCUCGGGGUGUCCGUAA CUCCGAUCCAAAGGAUUGUCCUGAGCGGUGAAAAUGGG CUGAAGAUCGACAUCCAUGUCAUCAUCCCGUAUGAAGG UCUGAG

2.2.2 DNA oligonucleotides used in this study

Table S4. DNA oligonucleotides used as primers.

Name	Size (nt)	Modification	Sequence (5´→3´ direction)
**FAM-DNA-prim_15nt**	15	5´-FAM	CATGGGCGGCATGGG
**Cy5-DNA-prim_15nt**	15	5´-Cy5	CATGGGCGGCATGGG
**FAM-DNA-prim_dU**	17	internal [dU]	CATGGGCGGCATGGGA[dU]
**Cy5-DNA-prim_dU_dT-FAM**	17	5´-Cy5; internal [dU][dT-FAM]	CATGGGCGGCATGGG[dU][dT-FAM]
**Cy5-DNA-prim_20nt**	20	5´-Cy5	GACATCATGAGAGACATCGC
**DNA-REV-prim_21nt**	21	–	ACACCAAAATACATTCACACA
**DNA-REV-prim_24nt**	24	–	CTCAGACCTTCATACGGGATGATG
**DNA-FOR-prim_26nt**	26	–	AATATGATGATAATATGGTTATAATT
**DNA-REV-prim_26nt**	26	–	ACCATGGTTGTGGCCATATTATCATC
**5´-(dual-Bio)-DNA-FOR-prim_29nt**	29	5´-dual-Bio	CCCTTTCGTCTAATACGACTCACTATAGG
**5´-(P)-DNA-FOR-prim_29nt**	29	5´-P	CCCTTTCGTCTAATACGACTCACTATAGG
**DNA-REV-prim_48nt**	48	–	TTTTTTTTTTTTTTTTTTTTTTTTTTTTTT TACGCCAGAATGCGTTC
**5´-(dual-Bio)-DNA-REV-prim_48nt**	48	5´-dual-Bio	CCCTTTCGTCTAATACGACTCACTATAGG

Table S5. DNA oligonucleotides used as templates.

Name	Size (nt)	Modification	Sequence (5´→3´ direction)
**templ_16nt**	16	–	GCCCATGCCGCCCATG
**templ_19nt_A**	19	–	CCCTCCCATGCCGCCCATG

**templ_19nt_U**	19	–	CCCACCCATGCCGCCCATG
**templ_19nt_C**	19	–	CCCGCCCATGCCGCCCATG
**templ_19nt_G**	19	–	AAACCCCATGCCGCCCATG
**templ_19nt_mix**	19	–	CAGTCCCATGCCGCCCATG
**5´-(TINA)-templ_31nt**	31	5´-*ortho*-TINA	CTAGCATGAGCTCAGTCCCATGCCGCCCATG
**5´-(dual-Bio)-templ_31nt**	31	5´-dual-Bio	CTAGCATGAGCTCAGTCCCATGCCGCCCATG
**templ_31nt**	31	–	CTAGCATGAGCTCAGTCCCATGCCGCCCATG
**templ_35nt**	35	–	AGCTAGCATGAGCTCAGTAACCCATGCCGCCCATG
**templ_36nt**	36	–	ATCTCACATGCTATACACTATCCCATGCCGCCCATG
**templ_poly-U**	45	–	GTCTCGGCTGGGCGTTTCCCTAAAAAAAAACCCATGC<br>CGCCCATG
**templ_50nt**	50	–	TCCCTATCCCAGCATACCTCTGTACTGATCCTCCCCC<br>CATGCCGCCCATG
**templ_ribosw71_A**	50	–	AGGCTCTTGGTAGAAACTCCCAAACCATATCATTAGG<br>ATTATATCTTCCC
**templ_65nt**	65	–	GGACTACTTCTAATCTGTAAGAGCAGATCCCTGGCTA<br>GCATGAGCTCAGTCCCATGCCGCCCATG
**templ_ribosw71_B**	71	–	GGGAAGATAATCAAGAGTTTAAGGCTCTTGGTAGAAA<br>CTCCCAAACCATATCATTAGGATTATATCTTCCC
**templ_98nt**	98	–	GACATCATGAGAGACATCGCCTCTGGGCTAATAGGAC<br>TACTTCTAATCTGTAAGAGCAGATCCCTGGCTAGCAT<br>GAGCTCAGTCCCATGCCGCCCATG
**templ_IRES**	622	–	ACCATGGTTGTGGCCATATTATCATCGTGTTTTTCAA<br>AGGAAAACCACGTCCCCGTGGTTCGGGGGGCCTAGAC<br>GTTTTTTTAACCTCGACTAAACACATGTAAAGCATGT<br>GCACCGAGGCCCCAGATCAGATCCCATACAATGGGGT<br>ACCTTCTGGGCATCCTTCAGCCCCTTGTTGAATACGC<br>TTGAGGAGAGCCATTTGACTCTTTCCACAACTATCCA<br>ACTCACAACGTGGCACTGGGGTTGTGCCGCCTTTGCA<br>GGTGTATCTTATACACGTGGCTTTTGGCCGCAGAGGC<br>ACCTGTCGCCAGGTGGGGGGTTCCGCTGCCTGCAAAG<br>GGTCGCTACAGACGTTGTTTGTCTTCAAGAAGCTTCC<br>AGAGGAACTGCTTCCTTCACGACATTCAACAGACCTT<br>GCATTCCTTTGGCGAGAGGGGAAAGACCCCTAGGAAT<br>GCTCGTCAAGAAGACAGGGCCAGGTTTCCGGGCCCTC<br>ACATTGCCAAAAGACGGCAATATGGTGGAAAATAACA<br>TATAGACAAACGCACACCGGCCTTATTCCAAGCGGCT<br>TCGGCCAGTAACGTTAGGGGGGGGGGGAGGGAGAGGGG<br>CCCTATAGTGAGTCGTATTAGACGAAAGGG

**templ_IRES-prolonged**	820	–	CTCAGACCTTCATACGGGATGATGACATGGATGTCGA<br>TCTTCAGCCCATTTTCACCGCTCAGGACAATCCTTTG<br>GATCGGAGTTACGGACACCCCGAGATTCTGAAACAAA<br>CTGGACACACCTCCCTGTTCAAGGACTTGGTCCAGGT<br>TGTAGCCGGCTGTCTGTCGCCAGTCCCCAACGAAATC<br>TTCGAGTGTGAAGACCATGGTTGTGGCCATATTATCA<br>TCGTGTTTTTCAAAGGAAAACCACGTCCCCGTGGTTC<br>GGGGGGCCTAGACGTTTTTTTAACCTCGACTAAACAC<br>ATGTAAAGCATGTGCACCGAGGCCCCAGATCAGATCC<br>CATACAATGGGGTACCTTCTGGGCATCCTTCAGCCCC<br>TTGTTGAATACGCTTGAGGAGAGCCATTTGACTCTTT<br>CCACAACTATCCAACTCACAACGTGGCACTGGGGTTG<br>TGCCGCCTTTGCAGGTGTATCTTATACACGTGGCTTT<br>TGGCCGCAGAGGCACCTGTCGCCAGGTGGGGGGTTCC<br>GCTGCCTGCAAAGGGTCGCTACAGACGTTGTTTGTCT<br>TCAAGAAGCTTCCAGAGGAACTGCTTCCTTCACGACA<br>TTCAACAGACCTTGCATTCCTTTGGCGAGAGGGGAAA<br>GACCCCTAGGAATGCTCGTCAAGAAGACAGGGCCAGG<br>TTTCCGGGCCCTCACATTGCCAAAAGACGGCAATATG<br>GTGGAAAATAACATATAGACAAACGCACACCGGCCTT<br>ATTCCAAGCGGCTTCGGCCAGTAACGTTAGGGGGGGG<br>GGAGGGAGAGGGGCCCTATAGTGAGTCGTATTAGACG<br>AAAGGG
**templ_IRES-nLuc**	1163	–	TTTTTTTTTTTTTTTTTTTTTTTTTTTTTTTTACGCC<br>AGAATGCGTTCGCACAGCCGCCAGCCGGTCACTCCGT<br>TGATGGTTACTCGGAACAGCAGGGAGCCGTCGGGGTT<br>GATCAGGCGCTCGTCGATAATTTTGTTGCCGTTCCAC<br>AGGGTCCCTGTTACAGTGATCTTTTTGCCGTCGAACA<br>CGGCGATGCCTTCATACGGCCGTCCGAAATAGTCGAT<br>CATGTTCGGCGTAACCCCGTCGATTACCAGTGTGCCA<br>TAGTGCAGGATCACCTTAAAGTGATGATCATCCACAG<br>GGTACACCACCTTAAAAATTTTTTCGATCTGGCCCAT<br>TTGGTCGCCGCTCAGACCTTCATACGGGATGATGACA<br>TGGATGTCGATCTTCAGCCCATTTTCACCGCTCAGGA<br>CAATCCTTTGGATCGGAGTTACGGACACCCCGAGATT<br>CTGAAACAAACTGGACACACCTCCCTGTTCAAGGACT<br>TGGTCCAGGTTGTAGCCGGCTGTCTGTCGCCAGTCCC<br>CAACGAAATCTTCGAGTGTGAAGACCATGGTTGTGGC<br>CATATTATCATCGTGTTTTTCAAAGGAAAACCACGTC<br>CCCGTGGTTCGGGGGGCCTAGACGTTTTTTTAACCTC<br>GACTAAACACATGTAAAGCATGTGCACCGAGGCCCCA<br>GATCAGATCCCATACAATGGGGTACCTTCTGGGCATC<br>CTTCAGCCCCTTGTTGAATACGCTTGAGGAGAGCCAT<br>TTGACTCTTTCCACAACTATCCAACTCACAACGTGGC<br>ACTGGGGTTGTGCCGCCTTTGCAGGTGTATCTTATAC<br>ACGTGGCTTTTGGCCGCAGAGGCACCTGTCGCCAGGT<br>GGGGGGTTCCGCTGCCTGCAAAGGGTCGCTACAGACG<br>TTGTTTGTCTTCAAGAAGCTTCCAGAGGAACTGCTTC<br>CTTCACGACATTCAACAGACCTTGCATTCCTTTGGCG<br>AGAGGGGAAAGACCCCTAGGAATGCTCGTCAAGAAGA<br>CAGGGCCAGGTTTCCGGGCCCTCACATTGCCAAAAGA<br>CGGCAATATGGTGGAAAATAACATATAGACAAACGCA<br>CACCGGCCTTATTCCAAGCGGCTTCGGCCAGTAACGT<br>TAGGGGGGGGGGAGGGAGAGGGGCCCTATAGTGAGTC<br>GTATTAGACGAAAGGG

**templ_(dual-Bio)-IRES-nLuc**	1163	5´-dual-Bio	TTTTTTTTTTTTTTTTTTTTTTTTTTTTTTTTACGCC AGAATGCGTTCGCACAGCCGCCAGCCGGTCACTCCGT TGATGGTTACTCGGAACAGCAGGGAGCCGTCGGGGTT GATCAGGCGCTCGTCGATAATTTTGTTGCCGTTCCAC AGGGTCCCTGTTACAGTGATCTTTTTGCCGTCGAACA CGGCGATGCCTTCATACGGCCGTCCGAAATAGTCGAT CATGTTCGGCGTAACCCCGTCGATTACCAGTGTGCCA TAGTGCAGGATCACCTTAAAGTGATGATCATCCACAG GGTACACCACCTTAAAAATTTTTTCGATCTGGCCCAT TTGGTCGCCGCTCAGACCTTCATACGGGATGATGACA TGGATGTCGATCTTCAGCCCATTTTCACCGCTCAGGA CAATCCTTTGGATCGGAGTTACGGACACCCCGAGATT CTGAAACAAACTGGACACACCTCCCTGTTCAAGGACT TGGTCCAGGTTGTAGCCGGCTGTCTGTCGCCAGTCCC CAACGAAATCTTCGAGTGTGAAGACCATGGTTGTGGC CATATTATCATCGTGTTTTTCAAAGGAAAACCACGTC CCCGTGGTTCGGGGGGCCTAGACGTTTTTTTAACCTC GACTAAACACATGTAAAGCATGTGCACCGAGGCCCCA GATCAGATCCCATACAATGGGGTACCTTCTGGGCATC CTTCAGCCCCTTGTTGAATACGCTTGAGGAGAGCCAT TTGACTCTTTCCACAACTATCCAACTCACAACGTGGC ACTGGGGTTGTGCCGCCTTTGCAGGTGTATCTTATAC ACGTGGCTTTTGGCCGCAGAGGCACCTGTCGCCAGGT GGGGGGTTCCGCTGCCTGCAAAGGGTCGCTACAGACG TTGTTTGTCTTCAAGAAGCTTCCAGAGGAACTGCTTC CTTCACGACATTCAACAGACCTTGCATTCCTTTGGCG AGAGGGGAAAGACCCCTAGGAATGCTCGTCAAGAAGA CAGGGCCAGGTTTCCGGGCCCTCACATTGCCAAAAGA CGGCAATATGGTGGAAAATAACATATAGACAAACGCA CACCGGCCTTATTCCAAGCGGCTTCGGCCAGTAACGT TAGGGGGGGGGGAGGGAGAGGGGCCCTATAGTGAGTC GTATTAGACGAAAGGG

Table S6. DNA oligonucleotides for dsDNA template construction.

Name		Size (nt)	Modification	Sequence (5´→3´ direction)
**ds-templ_poly-U**	sense strand	50	–	TAATACGACTCACTATAGGGTTTTTTT TTAGGGAAACGCCCAGCCGAGAC
	anti-sense strand	50	5´-[2´-*O*-Me-G]; [2´-*O*-Me-U]	[mG][mU]CTCGGCTGGGCGTTTCCCT AAAAAAAAACCCTATAGTGAGTCGTAT TA
**ds-templ_52bp**	sense strand	52	–	TAATACGACTCACTATAGGGAGGATCA GTACAGAGGTATGCTGGGATAGGGA
	anti-sense strand	52	5´-[2´-*O*-Me-U]; [2´-*O*-Me-C]	[mU][mC]CCTATCCCAGCATACCTCT GTACTGATCCTCCCTATAGTGAGTCGT ATTA

Table S7. DNA oligonucleotides used in fluorescence measurements.

Name	Size (nt)	Modification	Sequence (5´→3´ direction)
**Cy5-DNA-oligo**	15	5´-Cy5	CATGGGCGGCATGGG
**Cy3-DNA-oligo**	20	5´-Cy3	TGGCGCGAAGGCTGTCATTG

2.2.3 Oligonucleotide modifications

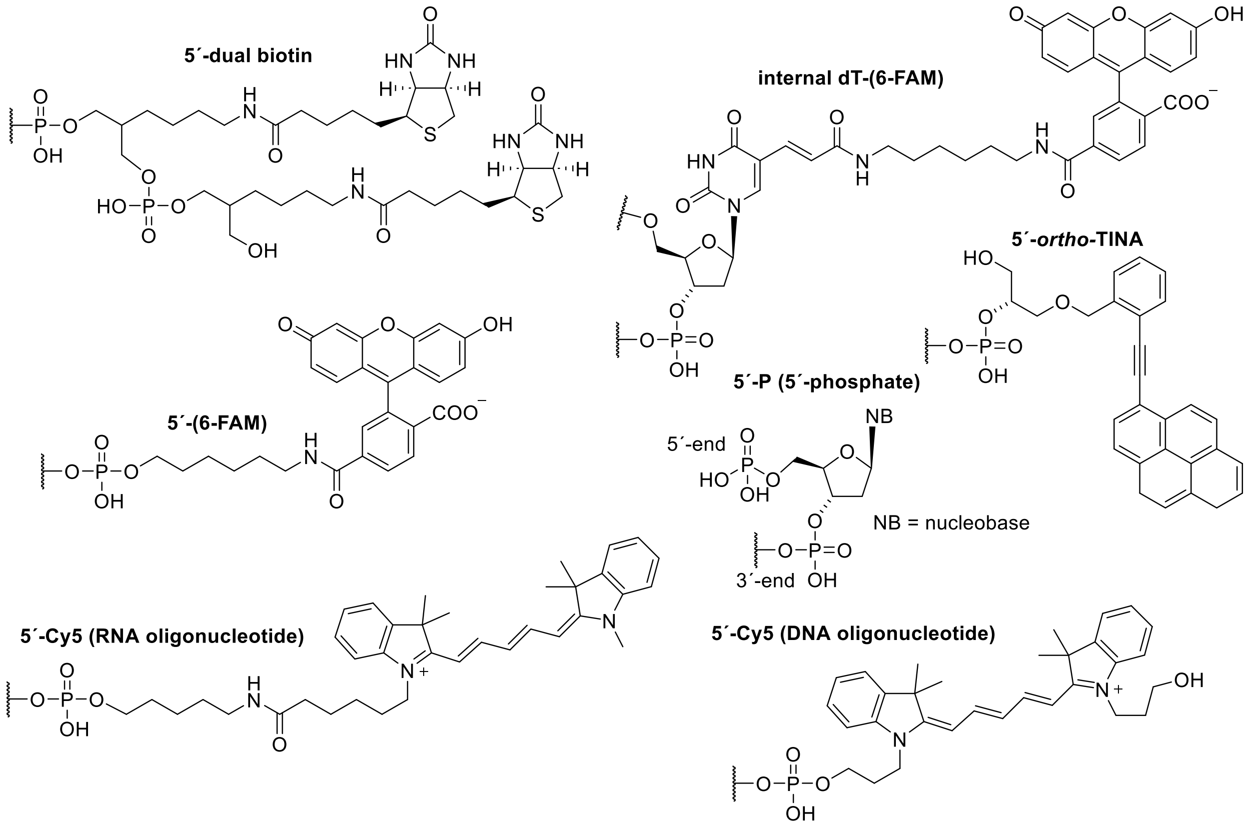

Figure S1. Structures of modifications of synthetic oligonucleotides.

2.3 Chemical structures of base-modified rN^X^TPs used in this study

2.3.1 Chemical structures of hydrophobic and/or clickable base-modified rN^X^TPs

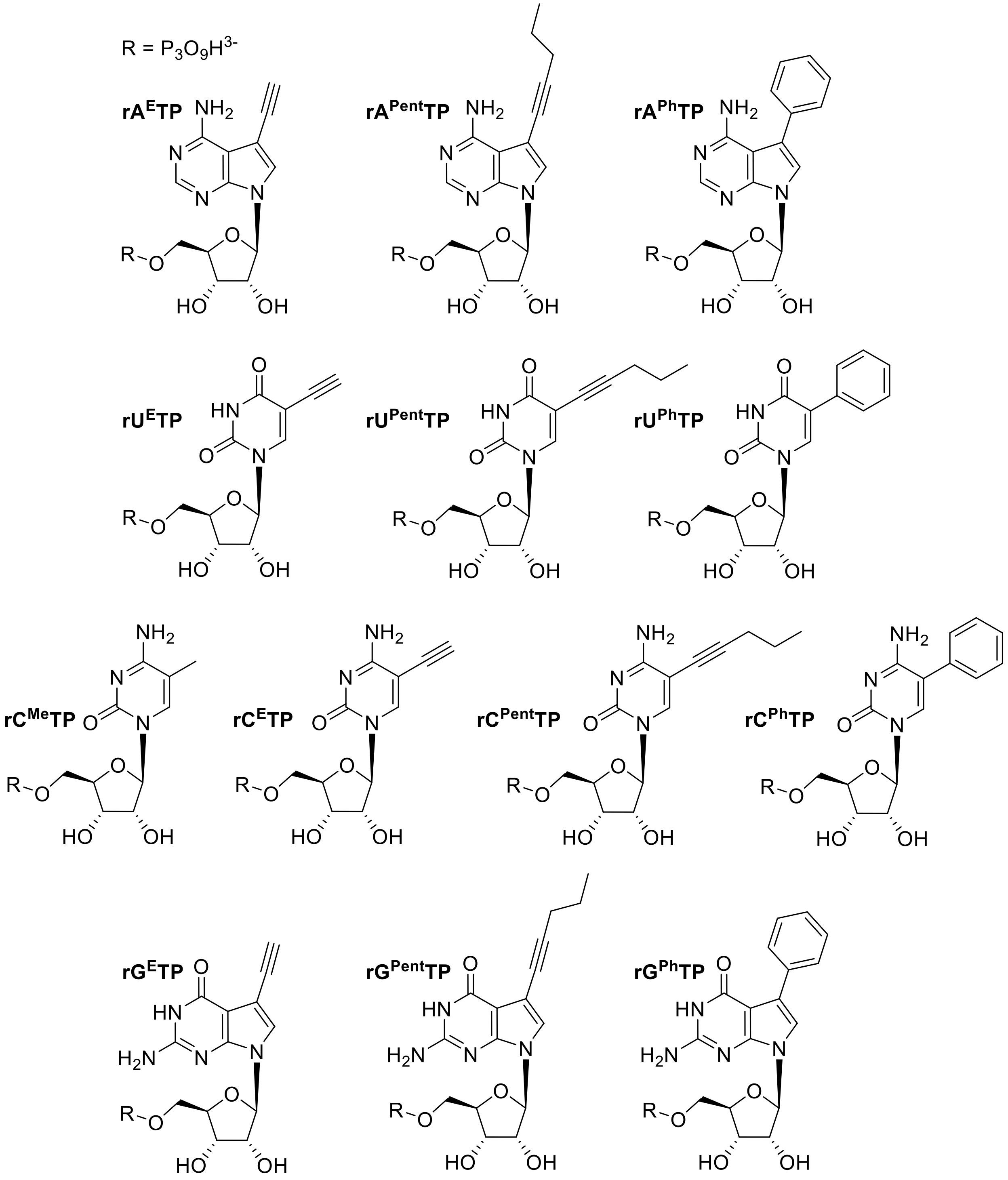

Figure S2. Chemical structures of Me-, E-, Pent-, Ph-modified **rN^X^TPs**.

2.3.2 Chemical structures of fluorescent base-modified rN^{X}TPs

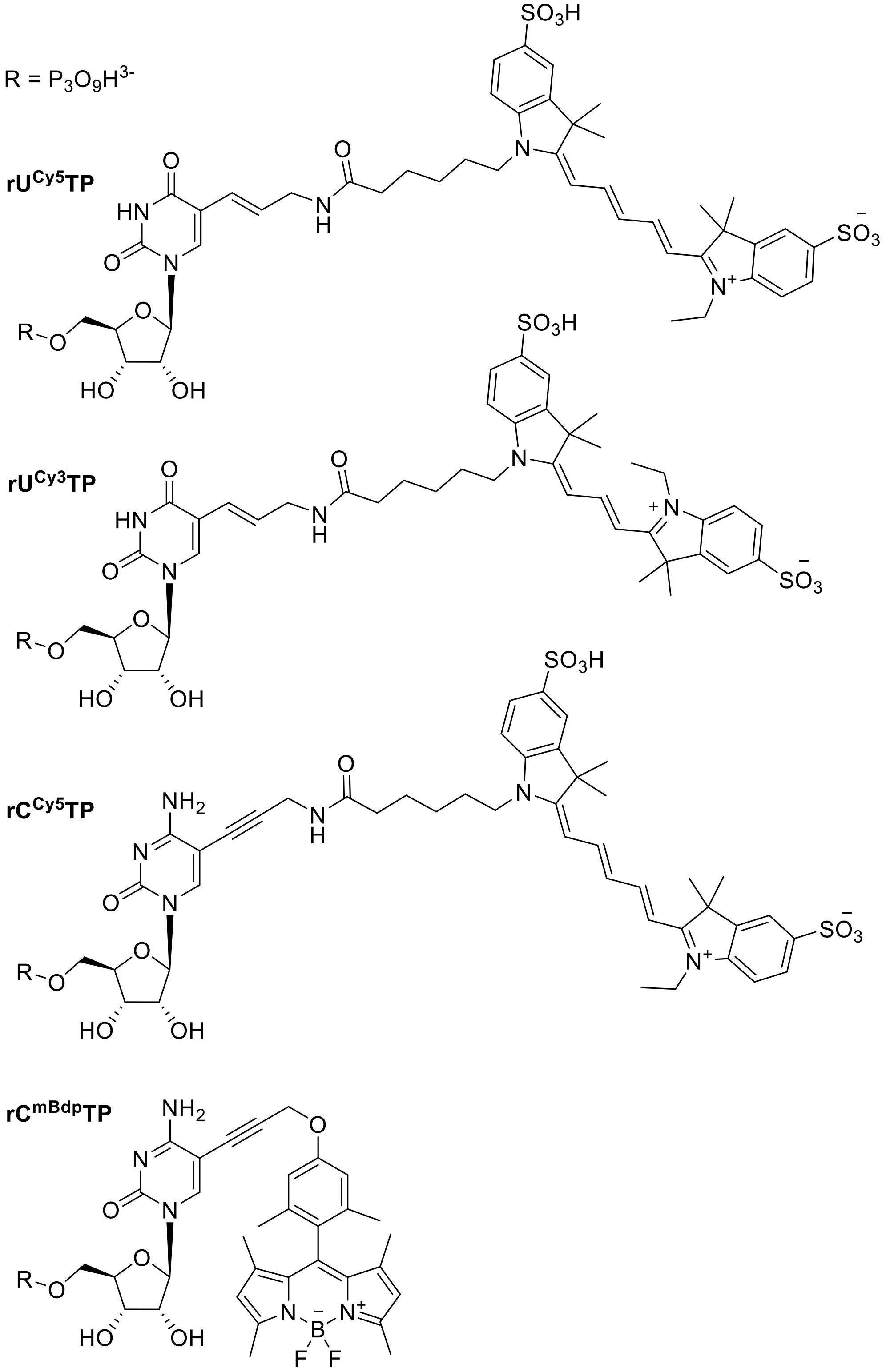

Figure S3. Chemical structures of Cy5-, Cy3-, mBdp-modified **rN^{X}TPs**.

2.3.3 Chemical structures of hapten base-modified rN^XTPs

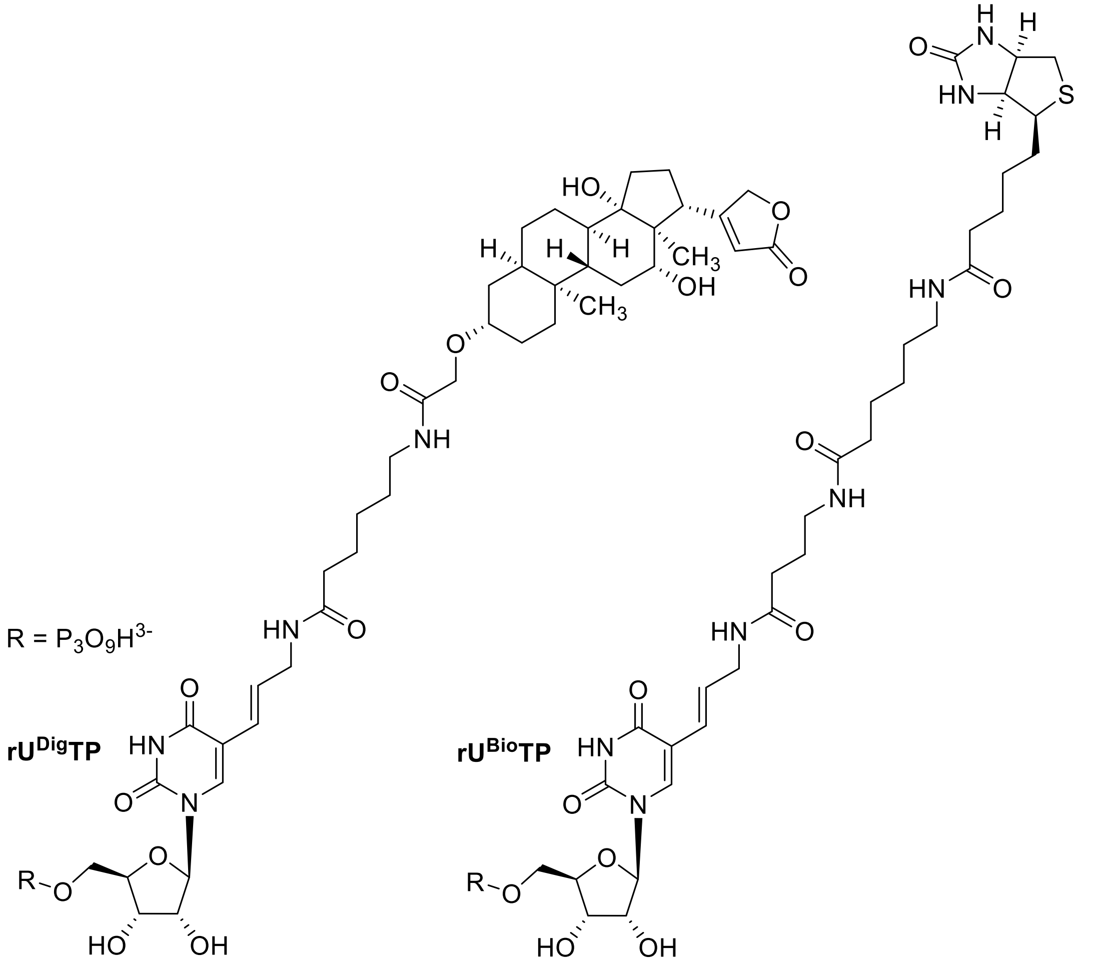

Figure S4. Chemical structures of Dig-, Bio-modified **rN^X^TP**s.

2.3.4 Chemical structures of reactive base-modified rN^{X}TPs

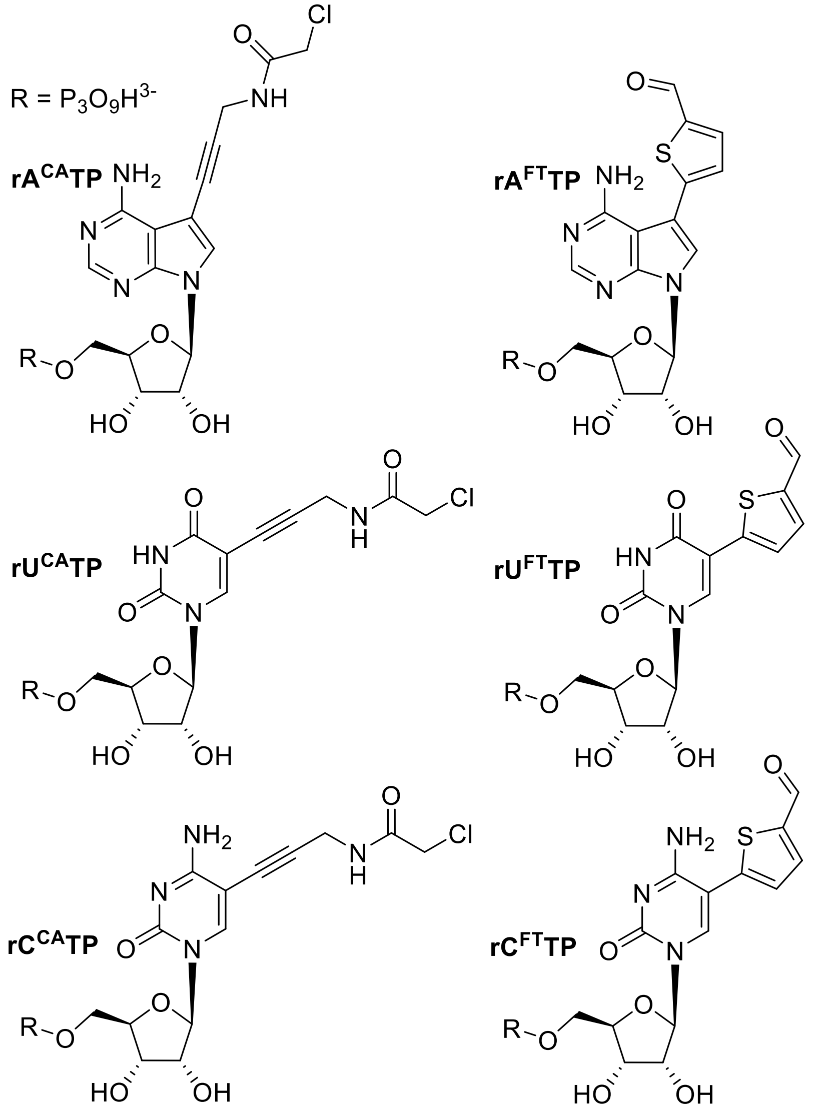

Figure S5. Chemical structures of CA-, FT-modified **rN^{X}TP**s.

2.4 Engineered DNA polymerases – expression and purification

2.4.1 Protocol for TGK polymerase[12,13]

NEB 10-beta cells were transformed by pGDR11-TGLK plasmid (kind gift from J. C. Chaput) and outgrown in 20 mL 2X YT medium containing 100 µg/mL ampicillin, overnight. Next day, cells were harvested and resuspended in 10 mL of fresh 2X YT medium, which was used to inoculate 2X 1 L flasks with 2X YT medium containing 100 µg/mL ampicillin. Cultures were shaken at 37 °C until OD_{600} = 1.0. Expression was triggered with IPTG (1 mM) for 5 h at 37 °C. Cells were harvested at 8 000 g, 4 °C, 10 min and the pellet was stored at -80 °C. Pellet was then resuspended in buffer containing TRIS (10 mM), NaCl (200 mM), glycerol (20%), pH 8.5 and lysed at 80 °C for 1 h, followed by cooling on ice for 20 min. Precipitates were removed

at 100 000 g, 4 °C, 30 min. First purification was carried out on HiTrap Heparin HP 5 mL column by gradient from 200 to 1000 mM NaCl. Pure fractions were combined and repurified on HisTrap HP 5 mL column by elution with gradient of imidazole (from 0 to 300 mM) in 50 mM TRIS, 500 mM NaCl, 10% glycerol, pH 8.5 buffer. Pure fractions were combined, concentrated and buffer exchanged on 30 kDa MWCO Vivaspin concentrators with buffer containing HEPES (40 mM), KCl (250 mM), EDTA (1 mM), pH 7.4, quantified by Nanodrop and stored as 50% glycerol solution at -80 °C.

2.4.2 Protocol for SFM4-3 polymerase[14]

BL21(DE3) pLysS cells containing expression plasmid (pET-SFM4-3-His_6, kind gift from F. E. Romesberg) were outgrown from frozen glycerol stock in 100 mL LB media (100 µg/mL ampicillin, 34 µg/mL chloramphenicol) overnight at 34 °C, 200 rpm. Next day, 5 mL of media was used to inoculate 1 L of LB containing antibiotics and cells were grown until OD_{600} = 0.5. Induction was triggered with IPTG (0.4 mM) and let to proceed at 30 °C for 6 hr. Cells were harvested by centrifugation at 3 200 g at 4 °C, 30 min. Pellet was resuspended in lysis buffer containing TRIS (50 mM), NaCl (300 mM), imidazole (10 mM), pH 7.4 and cells were disrupted by sonication. Lysate was then incubated at 70 °C for 30 minutes. Precipitates were removed by centrifugation at 20 000 g, 30 min, 4 °C. Supernatant was loaded on HisTrap FF 1 mL column connected to ÄKTA pure machine and eluted by gradient from 10 to 500 mM imidazole. Eluted fractions were pooled, concentrated and buffer exchanged on 30 kDa MWCO Vivaspin concentrators to buffer containing TRIS (50 mM), EDTA (0.5 mM), pH 8.5 quantified by Nanodrop and stored as 50% glycerol solution at -80 °C.

2.5 General procedures

2.5.1 Sample preparation protocol for dPAGE analysis

Samples were diluted with H_2O to final 0.2 µM concentration. 10 µL of this solution were combined with 10 µL of 2X stop solution (95% [v/v] formamide, 0.5 mM EDTA, 0.025% [w/v] bromophenol blue, 0.025% [w/v] SDS in H_2O), denatured by heating at 95 °C for 2 min and then immediately cooled on ice. Aliquots of the denatured samples (10 µL) were subjected to vertical gel electrophoresis on either 12.5% or 22.5% (as indicated by gels) dPAGE (19:1 mono:bis acrylamide) containing TBE buffer (1X, pH 8.0) and urea (7 M) at 25 mA. Gels were visualised by fluorescence imaging.

2.5.2 Urea agarose gel analysis

Aliquots of **Cy5-IRES-RNA** or **Cy5-IRES-RNA_prolonged** used as standards and each of the quantified, fluorescently labelled mRNA samples (150 ng) were combined with 2X stop solution (95% [v/v] formamide, 0.5 mM EDTA, 0.025% [w/v] bromophenol blue, 0.025% [w/v] SDS in H_2O) and loaded on gel. 5 µL of each RNA ladders (RiboRuler High Range, RiboRuler

Low Range), prepared according to manufacturer´s procedure was loaded on gel. All samples were analysed on 1.5% agarose gel containing 2 M urea, TAE buffer (1X) and GelRed (1X) in TAE running buffer (1X) with addition of 2 M urea at 100 V at room temperature. The gel was afterwards visualised by a fluorescent scanner.

2.5.3 Preparation of dsDNA templates for IVT reactions

Solution of complementary ssDNA oligonucleotides (10 µL, 100 µM of each) in annealing buffer containing TRIS (10 mM), NaCl (50 mM), EDTA (1 mM), pH 7.8 was heated up to 95 °C for 5 min and then slowly cooled down to 25 °C (0.02 °C s^{-1}) in a thermal cycler (with heated lid to 110 °C).

2.5.4 Standard conditions for analytical scale PEX reaction using templ_19nt_X (X = A, U, C, G)

Reaction mixture was heated up to 95 °C for 30 sec followed by incubation at 60 °C for 2 h in a thermal cycler with heated lid (100 °C). Afterwards, TurboDNase (2 U) was added, and the mixture was further incubated at 37 °C for 15 min, prior to dPAGE analysis. For preparation of the samples for dPAGE see section 2.5.1.

2.5.5 Modified conditions for analytical scale PEX reaction using templ_19nt_X (X = A, U, C)

Both primer and template in presence of ThermoPol buffer were heated up to 95 °C for 30 sec and then cooled down to 3 °C (0.1 °C s^{-1}). After addition of remaining components, the mixture was incubated at 60 °C for 2 h in a thermal cycler with heated lid (100 °C). After this time, TurboDNase (2 U) was added, and the mixture was further incubated at 37 °C for 15 min prior to dPAGE analysis. For preparation of the samples for dPAGE see section 2.5.1.

2.5.6 Procedure for analytical scale PEX reaction at mild conditions using templ_19nt_X (X = A, C)

Both primer and template in presence of ThermoPol buffer were heated up to 95 °C for 30 sec and then cooled down to 3 °C (0.1 °C s^{-1}). After addition of remaining components, the mixture was incubated at 37 °C for 2 h in a thermal cycler with heated lid (65 °C). After this time, TurboDNase (2 U) was added, and the mixture was further incubated at 37 °C for 15 min prior to dPAGE analysis. For preparation of the samples for dPAGE see section 2.5.1.

2.5.7 Standard conditions for analytical scale PEX reaction using 5´-(TINA)-templ_31nt

Reaction mixture was heated up to 95 °C for 30 sec followed by incubation at 60 °C for 2 h in a thermal cycler with heated lid (100 °C). After this time, TurboDNase (4 U) was added, and the mixture was further incubated at 37 °C for 30 min prior to dPAGE analysis. For preparation of the samples for dPAGE see section 2.5.1.

2.5.8 Standard conditions for analytical scale PEX reaction using four different base-modified $rN^{X}TPs$ and variously long templates

Both primer and template in presence of ThermoPol buffer were heated up to 95 °C for 30 sec and then cooled down to 3 °C (0.1 °C s^{-1}). After addition of remaining components, the mixture was incubated at 60 °C for 1 h (**templ_19nt_mix**), 2 h (**5´-(TINA)-templ_31nt**), 4 h (**templ_65nt**) or 6 h (**templ_98nt**) in a thermal cycler with heated lid (100 °C). After this time, TurboDNase (2 U for template – **templ_19nt_mix**; 4 U for templates – **5´-(TINA)-templ_31nt**; **templ_65nt**; **templ_98nt**) was added, and the mixture was further incubated at 37 °C for 30 min prior to dPAGE analysis. For preparation of the samples for dPAGE see section 2.5.1.

2.5.9 Standard conditions for semi-preparative scale PEX reaction using templ_19nt_X (X = A, U, C, G) followed by spin column purification

Reaction mixture was heated up to 95 °C for 30 sec followed by incubation at 60 °C for 2 h in a thermal cycler with heated lid (100 °C). After this time, TurboDNase (10 U) was added and the mixture was further incubated at 37 °C for 30 min. Sample was purified using Monarch RNA cleanup kit (50 µg), according to standard supplier´s protocol and eluted into DNase/RNase free tube with 50 µL of H_2O. Sample was evaporated to dryness and diluted again in 10 µL of H_2O prior to mass spectrometry analysis.

2.5.10 Modified conditions I for semi-preparative scale PEX reaction using templ_19nt_X (X = A, C) followed by spin column purification

Both primer and template in presence of ThermoPol buffer were heated up to 95 °C for 30 sec and then cooled down to 3 °C (0.1 °C s^{-1}). After addition of remaining components, the mixture was incubated at 60 °C for 15 min in a thermal cycler with heated lid (100 °C). After this time, TurboDNase (10 U) was added, and the mixture was further incubated at 37 °C for 30 min. The sample was purified using Monarch RNA cleanup kit (50 µg), according to standard supplier´s protocol and eluted into DNase/RNase free tube with 50 µL of H_2O. Sample was evaporated to dryness and diluted again in 10 µL of H_2O prior to mass spectrometry analysis.

2.5.11 Modified conditions II for semi-preparative scale PEX reaction using templ_19nt_X (X = A, U, C) followed by spin column purification

Both primer and template in presence of ThermoPol buffer were heated up to 95 °C for 30 sec and then cooled down to 3 °C (0.1 °C s^{-1}). After addition of remaining components, the mixture was incubated at 60 °C for 2 h in a thermal cycler with heated lid (100 °C). After this time, TurboDNase (10 U) was added, and the mixture was further incubated at 37 °C for 30 min. The sample was purified using Monarch RNA cleanup kit (50 µg), according to standard supplier´s protocol and eluted into DNase/RNase free tube with 50 µL of H_2O. Sample was evaporated to dryness and diluted again in 10 µL of H_2O prior to mass spectrometry analysis.

2.5.12 Procedure for semi-preparative scale PEX reaction at mild conditions using templ_19nt_X (X = A, C) followed by spin column purification

Both primer and template in presence of ThermoPol buffer were heated up to 95 °C for 30 sec and then cooled down to 3 °C (0.1 °C s^{-1}). After addition of remaining components, the mixture was incubated at 37 °C for 2 h in a thermal cycler with heated lid (65 °C). After this time, TurboDNase (10 U) was added, and the mixture was further incubated at 37 °C for 30 min. The sample was purified using Monarch RNA cleanup kit (50 µg), according to standard supplier´s protocol and eluted into DNase/RNase free tube with 50 µL of H_2O. Sample was evaporated to dryness and diluted again in 10 µL of H_2O prior to mass spectrometry analysis.

2.5.13 Conditions for semi-preparative scale PEX reaction using 5´-(TINA)-templ_31nt followed by spin column purification

Reaction mixture was heated up to 95 °C for 30 sec followed by incubation at 60 °C for 2 h in a thermal cycler with heated lid (100 °C). Afterwards, TurboDNase (20 U) was added, and the mixture was further incubated at 37 °C for 30 min. Sample was purified using Monarch RNA cleanup kit (50 µg), according to standard supplier´s protocol. Elution was performed into DNase/RNase free tube with 50 µL of H_2O. Sample was evaporated to dryness and diluted again in 10 µL of H_2O prior to mass spectrometry analysis.

2.5.14 Conditions for semi-preparative scale PEX reaction using 5´-(dual-Bio)-templ_31nt followed by magnetoseparation

Both primer and template in presence of ThermoPol buffer were heated up to 95 °C for 30 sec and then cooled down to 3 °C (0.1 °C s^{-1}). After addition of remaining components, the mixture was incubated at 60 °C for 6 h in a thermal cycler with heated lid (100 °C). After PEX reaction, the ssRNA strand was generated by magnetoseparation. Briefly, pre-washed SMB (100 µL) were combined with crude PEX reaction and 100 µL of binding buffer [TRIS (10 mM), EDTA (1 mM), NaCl (100 mM), pH 7.5] and incubated at 25 °C for 1 h at 800 rpm in a thermal mixer. Then, SMB were washed successively with wash buffer [TRIS (10 mM), EDTA (1 mM), NaCl (500 mM), pH 7.5] and water. Strand separation was performed in 50 µL of water by heating up the mixture to 70 °C for 5 min. Isolated product was cooled down, additionally combined with TurboDNase (10 U) and incubated at 37 °C for 1 h. Sample was evaporated to dryness and diluted again in 10 µL of H_2O prior to mass spectrometry analysis.

2.5.15 Conditions for semi-preparative scale PEX reaction using four different base-modified $rN^{X}TPs$ and variously long templates

Both primer and template in presence of ThermoPol buffer were heated up to 95 °C for 30 sec and then cooled down to 3 °C (0.1 °C s^{-1}). After addition of remaining components, the mixture was incubated at 60 °C for 1 h (**templ_19nt_mix**), 2 h (**5´-(TINA)-templ_31nt**), 4 h (**templ_65nt**) or 6 h (**templ_98nt**) in a thermal cycler with heated lid (100 °C). After this time, TurboDNase

(10 U for template – **templ_19nt_mix**; 20 U for templates – **5´-(TINA)-templ_31nt**; **templ_65nt**; **templ_98nt**) was added, and the mixture was further incubated at 37 °C for 30 min. Crude sample was further purified using QIAquick nucleotide removal kit according to standard supplier´s protocol. Elution was performed into DNase/RNase free tube with 50 µL of H_2O. Sample was evaporated to dryness and diluted again in 10 µL of H_2O prior to mass spectrometry analysis.

2.5.16 Standard protocol for RNA digestion and mass spectrometry analysis

Samples prepared either by PEX reaction with **rA^{CA}TP** (**19RNA_A^{CA}**) following the protocol in section 2.8.2 and/or by PEX reaction with **rC^{CA}TP** (**19RNA_C^{CA}**) following the protocol in section 2.8.12 were purified, lyophilised and again diluted in 17 µL of H_2O. Samples were combined with nucleoside digestion mix reaction buffer (2 µL, 10X) and nucleoside digestion mix (1 µL) followed by incubation at 37 °C for 1 h in a thermal cycler with heated lid (65 °C). 1 µL of the reaction was then directly analysed on Waters UPLC-H system coupled to Xevo G2-XS mass spectrometer in ESI$^+$ mode with internal calibration standard Leucine Enkephalin (556.2771 Da). Separation was performed on Luna C18 PS 2.1×150 mm column using mobile phases: (A) 0.1% formic acid in H_2O and (B) 0.1% formic acid in acetonitrile. Gradient was set as follows: 1.5 min hold 1% B then 7.5 min gradient from 1 to 50% B. Mass spectrometer settings were collection mass range 50-1200 Da, scan duration 0.5 s, 3.0 kV capillary voltage, 40 V sampling cone, 100 °C source temperature, 250 °C desolvation temperature, 50 L/h cone gas flow, 600 L/h desolvation gas flow.

2.5.17 Standard magnetoseparation procedure for generation of ssDNA templates after PCR

Generation of ssDNA templates was ensured by magnetoseparation procedure. Briefly, pre-washed SMB (100 µL per 6.5 µg dsDNA template) were combined with purified PCR product and binding buffer [TRIS (10 mM), EDTA (1 mM), NaCl (100 mM), TWEEN 20 (0.1%), pH 7.5] and incubated overnight at 25 °C on Hula-mixer. Then, SMB were successively washed with wash buffer [TRIS (10 mM), EDTA (1 mM), NaCl (500 mM), TWEEN 20 (0.1%), pH 7.5] and water. Strand separation was performed by addition of NaOH (50 mM), followed by neutralisation with TRIS (1 M, pH 7.5) and HCl (1 M). The generated ssDNA product was additionally purified using QIAquick nucleotide removal kit, according to standard supplier´s protocol.

2.5.18 Standard conditions for PCR reaction

PCR reaction was performed in a thermal cycler as follows: denaturation at 98 °C for 30 sec, then 25 cycles of denaturation at 98 °C for 15 sec, annealing at 68 °C for 30 sec and extension at 72 °C for 1 min, followed by final elongation step at 72 °C for 2 min.

2.5.19 Standard work up protocol for either IRES-RNA or IRES-RNA_prolonged

After PEX, the mixture (10 µL) was combined with TurboDNase (2 U) and incubated at 37 °C for 30 min followed by Proteinase K (0.8 U) digestion at 37 °C for 30 min. Final RNA was purified according to standard supplier´s protocol using RNA Clean & Concentrator-25 kit and quantified by fluorescence (Cy5) using Tecan Spark Multimode Reader. For 10X scaled-up reactions, the amount of TurboDNase (20 U) and Proteinase K (8 U) was increased accordingly.

2.5.20 Standard work up protocol for mRNA

After PEX, the mixture (10 µL) was combined with TurboDNase (2 U) and incubated at 37 °C for 30 min followed by Proteinase K (0.8 U) digestion at 37 °C for 30 min, prior to urea gel analysis according to protocol 2.5.2. Scaled-up reactions (30 µL) were treated with TurboDNase (6 U) and Proteinase K (2.4 U) following same conditions. Final mRNA mixtures were purified using magnetic mRNA isolation kit with a slightly modified protocol. Briefly, 500 µL of oligo-dT_{25}-MB pre-washed with binding buffer were incubated with mRNA (30 µL) at 25 °C for 1.5 h at 300 rpm in a thermal shaker. Afterwards, the oligo-dT_{25}-MB were washed with wash buffer 1, followed by wash buffer 2 and low salt buffer (each 1X 1 mL). The elution was performed with 100 µL of elution buffer at 50 °C for 3 min at 300 rpm in a thermal shaker. After lyophilisation to dryness, the mRNA was again diluted in 20 µL of H_2O, quantified by fluorescence (Cy5) using Tecan Spark Multimode Reader and used in *in vitro* and/or *in cellulo* translation studies in section 2.20.

2.5.21 Standard reaction conditions for RT-PCR

Reaction was treated as follows. For RT incubation at 50 °C for 10 min, then at 55 °C for 10 min was performed, followed by enzyme denaturation at 98 °C for 1 min. For PCR, 35 cycles of denaturation at 98 °C for 10 sec, annealing at 53 °C for 30 sec and extension at 72 °C for 40 sec were applied, followed by final elongation step at 72 °C for 5 min.

2.6 Analytical scale PEX reaction with templ_19nt_X (X = A, U, C, G) and TGK polymerase (incorporation of 1 modification)

2.6.1 Incorporation of $\mathbf{rA^{E}TP}$, $\mathbf{rA^{Pent}TP}$, $\mathbf{rA^{Ph}TP}$

Reaction was performed in total volume of 10 µL in ThermoPol buffer (1X) containing ssDNA template – **templ_19nt_A** (4.8 µM), 5´-(6-FAM)-labelled RNA primer – **FAM-RNA-prim_15nt** (4.0 µM), TGK polymerase (0.5 µM), rGTP (0.1 mM) and one of the modified $\mathbf{rA^{E}TP}$, $\mathbf{rA^{Pent}TP}$ or $\mathbf{rA^{Ph}TP}$ (0.1 mM). Positive control was performed under same conditions with natural rATP (0.1 mM) instead of modified $\mathbf{rA^{X}TP}$s. For negative control reaction H_2O was used instead of rATP. Reactions were carried out following the standard protocol in section 2.5.4. For dPAGE analysis of **19RNA_A** or $\mathbf{19RNA\_A^{E}}$, $\mathbf{19RNA\_A^{Pent}}$, $\mathbf{19RNA\_A^{Ph}}$ see Figure S6.

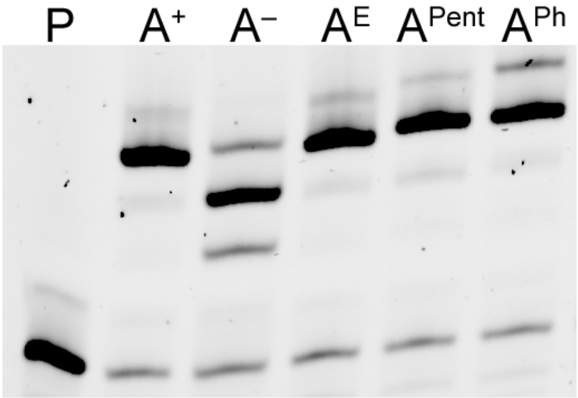

Figure S6. 22.5% dPAGE analysis of PEX reaction. (P) RNA primer; (A^{+}) positive control, mixture of rATP, rGTP; (A^{-}) negative control, mixture of rGTP and H_2O; (A^{E}) modification, mixture of **rA^{E}TP**, rGTP; (A^{Pent}) modification, mixture of **rA^{Pent}TP**, rGTP; (A^{Ph}) modification, mixture of **rA^{Ph}TP**, rGTP. FAM scan.

2.6.2 Incorporation of rA^{CA}TP, rA^{FT}TP

Reaction was performed in total volume of 10 µL in ThermoPol buffer (1X) containing ssDNA template – **templ_19nt_A** (4.8 µM), 5´-(6-FAM)-labelled RNA primer – **FAM-RNA-prim_15nt** (4.0 µM), TGK polymerase (0.25 µM), rGTP (0.1 mM) and one of the modified **rA^{CA}TP** or **rA^{FT}TP** (0.05 mM). Positive control was performed under same conditions with natural rATP (0.05 mM) instead of modified **rA^{X}TP**s. For negative control reaction H_2O was used instead of rATP. Reactions were carried out following the standard protocol in section 2.5.5. For dPAGE analysis of **19RNA_A** or **19RNA_A^{CA}**, **19RNA_A^{FT}** see Figure S7.

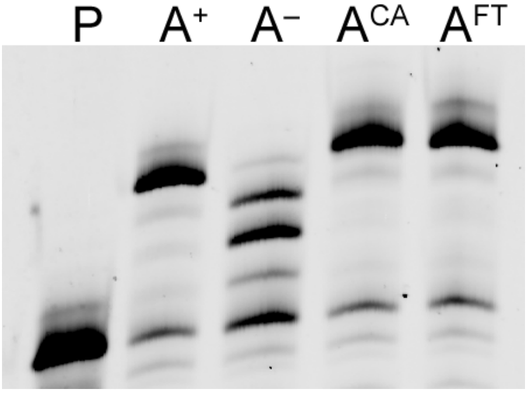

Figure S7. 22.5% dPAGE analysis of PEX reaction. (P) RNA primer; (A^{+}) positive control, mixture of rATP, rGTP; (A^{-}) negative control, mixture of rGTP and H_2O; (A^{CA}) modification, mixture of **rA^{CA}TP**, rGTP; (A^{FT}) modification, mixture of **rA^{FT}TP**, rGTP. FAM scan.

2.6.3 Incorporation of rA^{CA}TP at mild conditions

Reaction was performed in total volume of 10 µL in ThermoPol buffer (1X) containing ssDNA template – **templ_19nt_A** (4.8 µM), 5´-(6-FAM)-labelled RNA primer – **FAM-RNA-prim_15nt** (4.0 µM), TGK polymerase (0.5 µM), rGTP (0.2 mM) and the modified **rA^{CA}TP** (0.05 mM). Positive control was performed under same conditions with natural rATP (0.05 mM) instead of modified **rA^{CA}TP**. For negative control reaction H_2O was used instead of rATP. Reactions were carried out following the standard protocol in section 2.5.6. For dPAGE analysis of **19RNA_A** or **19RNA_A^{CA}** see Figure S8.

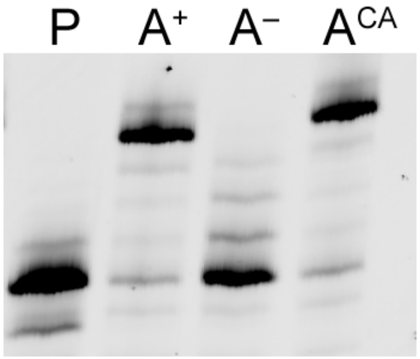

Figure S8. 22.5% dPAGE analysis of PEX reaction. (P) RNA primer; (A^{+}) positive control, mixture of rATP, rGTP; (A^{-}) negative control, mixture of rGTP and H_2O; (A^{CA}) modification, mixture of **rA^{CA}TP**, rGTP. FAM scan.

2.6.4 Incorporation of rU^{E}TP, rU^{Pent}TP, rU^{Ph}TP

Reaction was performed in total volume of 10 µL in ThermoPol buffer (1X) containing ssDNA template – **templ_19nt_U** (4.8 µM), 5´-(6-FAM)-labelled RNA primer – **FAM-RNA-prim_15nt** (4.0 µM), TGK polymerase (0.75 µM), rGTP (0.05 mM) and one of the modified **rU^{E}TP**, **rU^{Pent}TP** or **rU^{Ph}TP** (0.1 mM). Positive control was performed under same conditions with natural rUTP (0.1 mM) instead of modified **rU^{X}TP**s. For negative control reaction H_2O was used instead of rUTP. Reactions were carried out following the standard protocol in section 2.5.4. For dPAGE analysis of **19RNA_U** or **19RNA_U^{E}**, **19RNA_U^{Pent}**, **19RNA_U^{Ph}** see Figure S9.

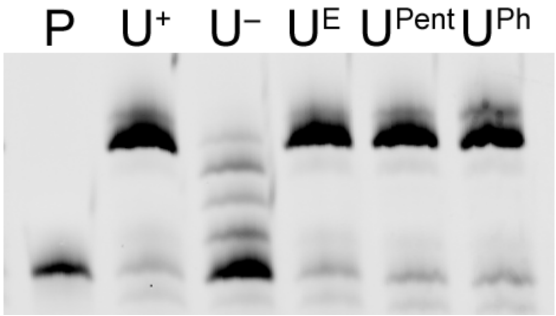

Figure S9. 22.5% dPAGE analysis of PEX reaction. (P) RNA primer; (U^+) positive control, mixture of rUTP, rGTP; ($U^–$) negative control, mixture of rGTP and H_2O; (U^E) modification, mixture of **rU^{E}TP**, rGTP; (U^{Pent}) modification, mixture of **rU^{Pent}TP**, rGTP; (U^{Ph}) modification, mixture of **rU^{Ph}TP**, rGTP. FAM scan.

2.6.5 Incorporation of rU^{Bio}TP, rU^{Dig}TP

Reaction was performed in total volume of 10 µL in ThermoPol buffer (1X) containing ssDNA template – **templ_19nt_U** (4.8 µM), 5´-(6-FAM)-labelled RNA primer – **FAM-RNA-prim_15nt** (4.0 µM), TGK polymerase (1.0 µM), rGTP (0.05 mM) and one of the modified **rU^{Bio}TP** or **rU^{Dig}TP** (0.1 mM). Positive control was performed under same conditions with natural rUTP (0.1 mM) instead of modified **rU^{X}TP**s. For negative control reaction H_2O was used instead of rUTP. Reactions were carried out following the standard protocol in section 2.5.4. For dPAGE analysis of **19RNA_U** or **19RNA_U^{Bio}**, **19RNA_U^{Dig}** see Figure S10.

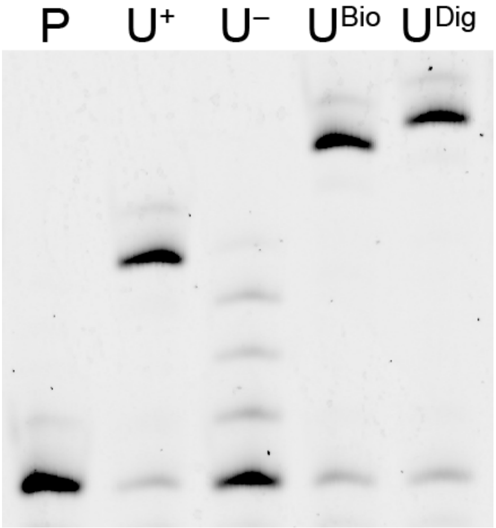

Figure S10. 22.5% dPAGE analysis of PEX reaction. (P) RNA primer; (U^+) positive control, mixture of rUTP, rGTP; ($U^–$) negative control, mixture of rGTP and H_2O; (U^{Bio}) modification, mixture of **rU^{Bio}TP**, rGTP; (U^{Dig}) modification, mixture of **rU^{Dig}TP**, rGTP. FAM scan.

2.6.6 Incorporation of rU^{CA}TP, rU^{FT}TP

Reaction was performed in total volume of 10 µL in ThermoPol buffer (1X) containing ssDNA template – **templ_19nt_U** (4.8 µM), 5´-(6-FAM)-labelled RNA primer – **FAM-RNA-prim_15nt** (4.0 µM), TGK polymerase (0.5 µM), rGTP (0.05 mM) and one of the modified **rU^{CA}TP** or **rU^{FT}TP** (0.05 mM). Positive control was performed under same conditions with natural rUTP (0.05 mM) instead of modified **rU^{X}TPs**. For negative control reaction H_2O was used instead of rUTP. Reactions were carried out following the standard protocol in section 2.5.5. For dPAGE analysis of **19RNA_U** or **19RNA_U^{CA}**, **19RNA_U^{FT}** see Figure S11.

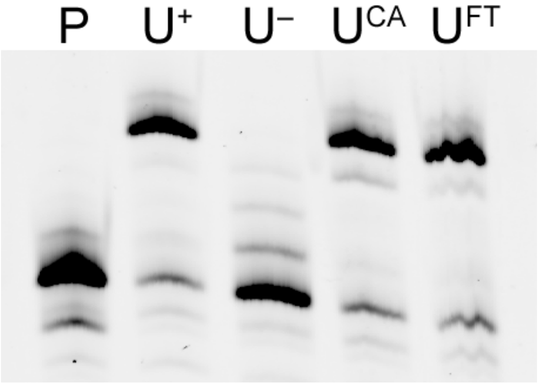

Figure S11. 22.5% dPAGE analysis of PEX reaction. (P) RNA primer; (U^{+}) positive control, mixture of rUTP, rGTP; (U$^{–}$) negative control, mixture of rGTP and H_2O; (U^{CA}) modification, mixture of **rU^{CA}TP**, rGTP; (U^{FT}) modification, mixture of **rU^{FT}TP**, rGTP. FAM scan.

2.6.7 Incorporation of rC^{Me}TP

Reaction was performed in total volume of 10 µL in ThermoPol buffer (1X) containing ssDNA template – **templ_19nt_C** (4.8 µM), 5´-(6-FAM)-labelled RNA primer – **FAM-RNA-prim_15nt** (4.0 µM), TGK polymerase (0.5 µM), rGTP (0.1 mM) and the modified **rC^{Me}TP** (0.1 mM). Positive control was performed under same conditions with natural rCTP (0.1 mM) instead of modified **rC^{Me}TP**. For negative control reaction H_2O was used instead of rCTP. Reactions were carried out following the standard protocol in section 2.5.4. For dPAGE analysis of **19RNA_C** or **19RNA_C^{Me}** see Figure S12.

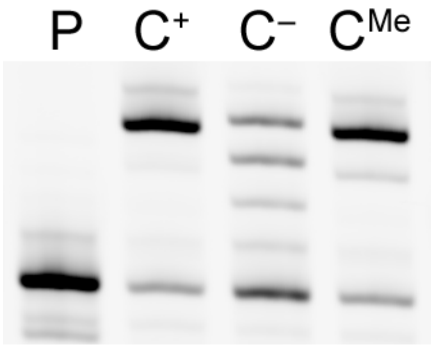

Figure S12. 22.5% dPAGE analysis of PEX reaction. (P) RNA primer; (C^{+}) positive control, mixture of rCTP, rGTP; ($C^{–}$) negative control, mixture of rGTP and H_2O; (C^{Me}) modification, mixture of **rC^{Me}TP**, rGTP. FAM scan.

2.6.8 Incorporation of rC^{E}TP, rC^{Pent}TP, rC^{Ph}TP

Reaction was performed in total volume of 10 µL in ThermoPol buffer (1X) containing ssDNA template – **templ_19nt_C** (4.8 µM), 5´-(6-FAM)-labelled RNA primer – **FAM-RNA-prim_15nt** (4.0 µM), TGK polymerase (0.5 µM), rGTP (0.2 mM) and one of the modified **rC^{E}TP**, **rC^{Pent}TP** or **rC^{Ph}TP** (0.1 mM). Positive control was performed under same conditions with natural rCTP (0.1 mM) instead of modified **rC^{X}TP**s. For negative control reaction H_2O was used instead of rCTP. Reactions were carried out following the standard protocol in section 2.5.4. For dPAGE analysis of **19RNA_C** or **19RNA_C^{E}**, **19RNA_C^{Pent}**, **19RNA_C^{Ph}** see Figure S13.

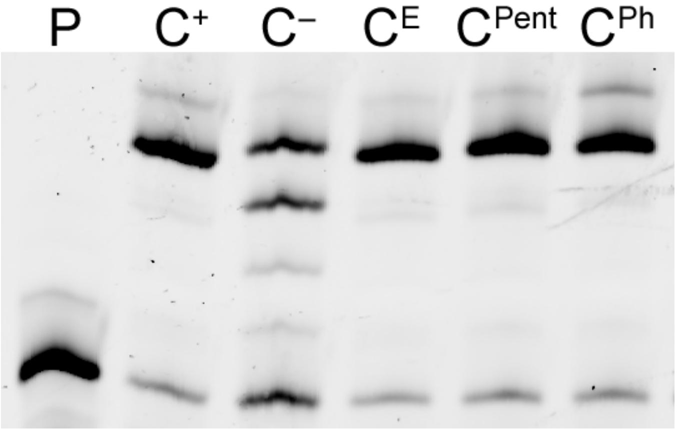

Figure S13. 22.5% dPAGE analysis of PEX reaction. (P) RNA primer; (C^{+}) positive control, mixture of rCTP, rGTP; ($C^{–}$) negative control, mixture of rGTP and H_2O; (C^{E}) modification, mixture of **rC^{E}TP**, rGTP; (C^{Pent}) modification, mixture of **rC^{Pent}TP**, rGTP; (C^{Ph}) modification, mixture of **rC^{Ph}TP**, rGTP. FAM scan.

2.6.9 Incorporation of rC^{mBdp}TP

Reaction was performed in total volume of 10 µL in ThermoPol buffer (1X) containing ssDNA template – **templ_19nt_C** (4.8 µM), 5´-(Cy5)-labelled RNA primer – **Cy5-RNA-prim_15nt** (4.0 µM), TGK polymerase (2.0 µM), rGTP (0.05 mM) and the modified **rC^{mBdp}TP** (0.1 mM). Positive control was performed under same conditions with natural rCTP (0.1 mM) instead of modified **rC^{mBdp}TP**. For negative control reaction H_2O was used instead of rCTP. Reactions were carried out following the standard protocol in section 2.5.4. For dPAGE analysis of **19RNA_C** or **19RNA_C^{mBdp}** see Figure S14.

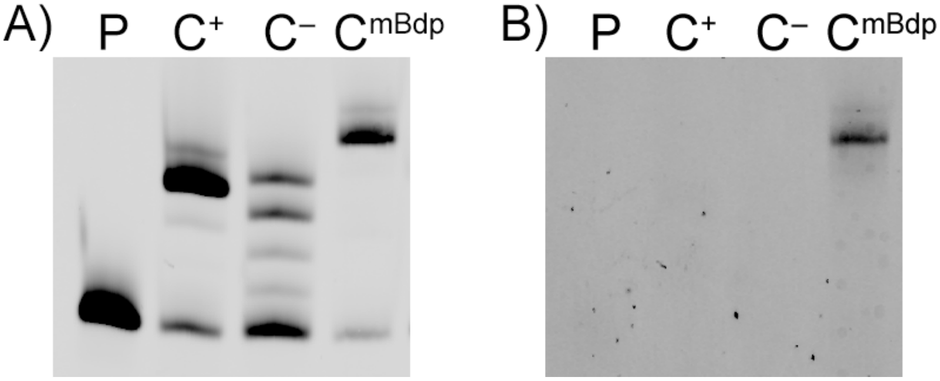

Figure S14. 22.5% dPAGE analysis of PEX reaction. (P) RNA primer; (C$^+$) positive control, mixture of rCTP, rGTP; (C$^–$) negative control, mixture of rGTP and H_2O; (C^{mBdp}) modification, mixture of **rC^{mBdp}TP**, rGTP. A) Cy5 scan; B) FAM scan (visualisation of mBdp-modification).

2.6.10 Incorporation of rC^{Cy5}TP

Reaction was performed in total volume of 10 µL in ThermoPol buffer (1X) containing ssDNA template – **templ_19nt_C** (4.8 µM), 5´-(6-FAM)-labelled RNA primer – **FAM-RNA-prim_15nt** (4.0 µM), TGK polymerase (2.0 µM), rGTP (0.05 mM) and the modified **rC^{Cy5}TP** (0.1 mM). Positive control was performed under same conditions with natural rCTP (0.1 mM) instead of modified **rC^{Cy5}TP**. For negative control reaction H_2O was used instead of rCTP. Reactions were carried out following the standard protocol in section 2.5.4. For dPAGE analysis of **19RNA_C** or **19RNA_C^{Cy5}** see Figure S15.

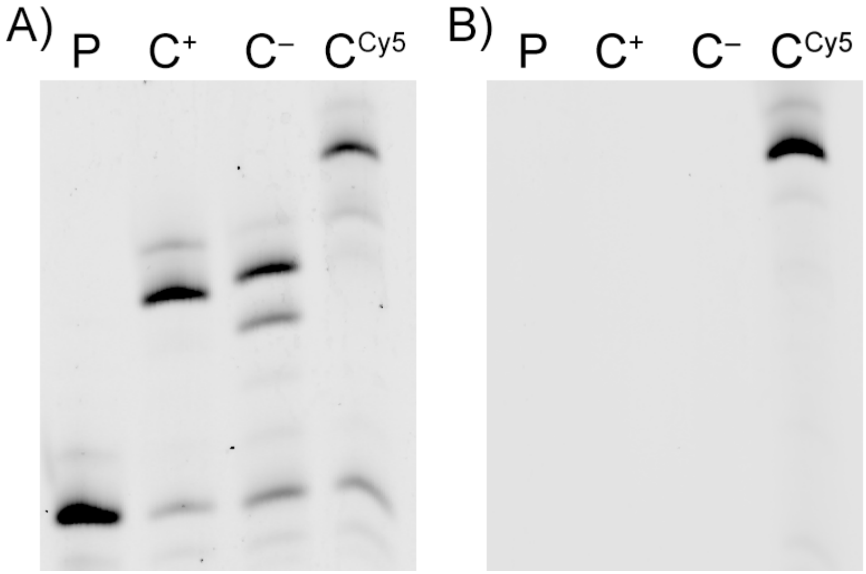

Figure S15. 22.5% dPAGE analysis of PEX reaction. (P) RNA primer; (C^{+}) positive control, mixture of rCTP, rGTP; (C^{-}) negative control, mixture of rGTP and H_2O; (C^{Cy5}) modification, mixture of **rC^{Cy5}TP**, rGTP. A) FAM scan; B) Cy5 scan (visualisation of Cy5-modification).

2.6.11 Incorporation of rC^{CA}TP, rC^{FT}TP

Reaction was performed in total volume of 10 µL in ThermoPol buffer (1X) containing ssDNA template – **templ_19nt_C** (4.8 µM), 5´-(6-FAM)-labelled RNA primer – **FAM-RNA-prim_15nt** (4.0 µM), TGK polymerase (0.5 µM), rGTP (0.2 mM) and one of the modified **rC^{CA}TP** or **rC^{FT}TP** (0.05 mM). Positive control was performed under same conditions with natural rCTP (0.05 mM) instead of modified **rC^{X}TP**s. For negative control reaction H_2O was used instead of rCTP. Reactions were carried out following the standard protocol in section 2.5.5. For dPAGE analysis of **19RNA_C** or **19RNA_C^{CA}**, **19RNA_C^{FT}** see Figure S16.

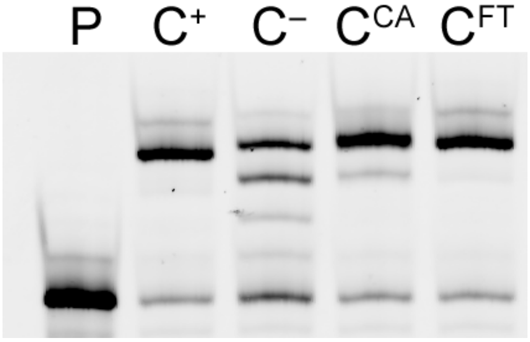

Figure S16. 22.5% dPAGE analysis of PEX reaction. (P) RNA primer; (C^{+}) positive control, mixture of rCTP, rGTP; (C^{-}) negative control, mixture of rGTP and H_2O; (C^{CA}) modification, mixture of **rC^{CA}TP**, rGTP; (C^{FT}) modification, mixture of **rC^{FT}TP**, rGTP. FAM scan.

2.6.12 Incorporation of rC^{CA}TP at mild conditions

Reaction was performed in total volume of 10 µL in ThermoPol buffer (1X) containing ssDNA template – **templ_19nt_C** (4.8 µM), 5´-(6-FAM)-labelled RNA primer – **FAM-RNA-prim_15nt** (4.0 µM), TGK polymerase (1.5 µM), rGTP (0.4 mM) and the modified **rC^{CA}TP** (0.05 mM). Positive control was performed under same conditions with natural rCTP (0.05 mM) instead of modified **rC^{CA}TP**. For negative control reaction H_2O was used instead of rCTP. Reactions were carried out following the standard protocol in section 2.5.6. For dPAGE analysis of **19RNA_C** or **19RNA_C^{CA}** see Figure S17.

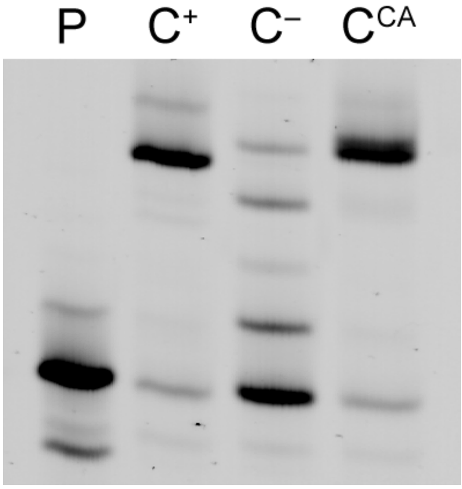

Figure S17. 22.5% dPAGE analysis of PEX reaction. (P) RNA primer; (C^{+}) positive control, mixture of rCTP, rGTP; (C$^{–}$) negative control, mixture of rGTP and H_2O; (C^{CA}) modification, mixture of **rC^{CA}TP**, rGTP. FAM scan.

2.6.13 Incorporation of rG^{E}TP, rG^{Pent}TP, rG^{Ph}TP

Reaction was performed in total volume of 10 µL in ThermoPol buffer (1X) containing ssDNA **templ_19nt_G** (4.8 µM), 5´-(6-FAM)-labelled RNA primer – **FAM-RNA-prim_15nt** (4.0 µM), TGK polymerase (1.0 µM), rUTP (0.2 mM) and one of the modified **rG^{E}TP**, **rG^{Pent}TP** or **rG^{Ph}TP** (0.1 mM). Positive control was performed under same conditions with natural rGTP (0.1 mM) instead of modified **rG^{X}TP**s. For negative control reaction H_2O was used instead of rGTP. Reactions were carried out following the standard protocol in section 2.5.4. For dPAGE analysis of **19RNA_G** or **19RNA_G^{E}**, **19RNA_G^{Pent}**, **19RNA_G^{Ph}** see Figure S18.

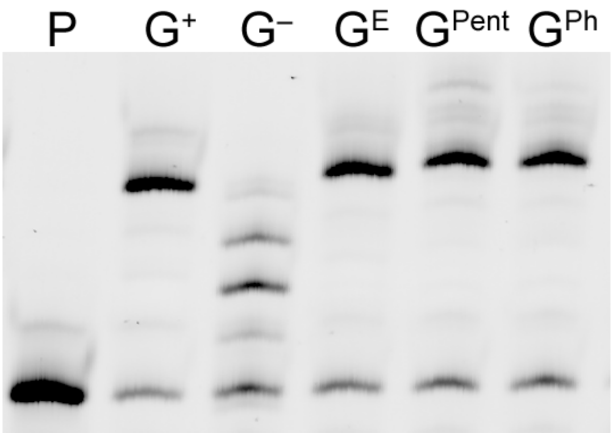

Figure S18. 22.5% dPAGE analysis of PEX reaction. (P) RNA primer; (G^+) positive control, mixture of rUTP, rGTP; ($G^–$) negative control, mixture of rUTP and H_2O; (G^E) modification, mixture of rUTP, **rG^{E}TP**; (G^{Pent}) modification, mixture of rUTP, **rG^{Pent}TP**; (G^{Ph}) modification, mixture of rUTP, **rG^{Ph}TP**. FAM scan.

2.7 Analytical scale PEX reaction with templ_19nt_X (X = A, U, C, G) and SFM4-3 polymerase (incorporation of 1 modification)

2.7.1 Incorporation of rA^{E}TP, rA^{Pent}TP, rA^{Ph}TP

Reaction was performed in total volume of 10 µL in ThermoPol buffer (1X) containing ssDNA template – **templ_19nt_A** (4.8 µM), 5´-(6-FAM)-labelled RNA primer – **FAM-RNA-prim_15nt** (4.0 µM), SFM4-3 polymerase (0.5 µM), rGTP (0.1 mM) and one of the modified **rA^{E}TP**, **rA^{Pent}TP** or **rA^{Ph}TP** (0.1 mM). Positive control was performed under same conditions with natural rATP (0.1 mM) instead of modified **rA^{X}TP**s. For negative control reaction H_2O was used instead of rATP. Reactions were carried out following the standard protocol in section 2.5.4. For dPAGE analysis of **19RNA_A** or **19RNA_A^E**, **19RNA_A^{Pent}**, **19RNA_A^{Ph}** see Figure S19.

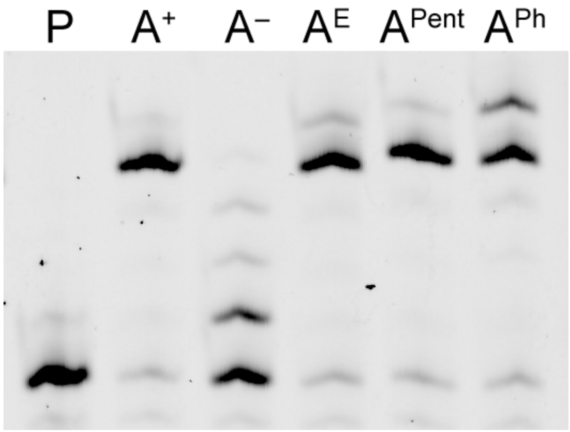

Figure S19. 22.5% dPAGE analysis of PEX reaction. (P) RNA primer; (A^{+}) positive control, mixture of rATP, rGTP; (A^{-}) negative control, mixture of rGTP and H_2O; (A^{E}) modification, mixture of **rA^E^TP**, rGTP; (A^{Pent}) modification, mixture of **rA^Pent^TP**, rGTP; (A^{Ph}) modification, mixture of **rA^Ph^TP**, rGTP. FAM scan.

2.7.2 Incorporation of rA^CA^TP, rA^FT^TP

Reaction was performed in total volume of 10 µL in ThermoPol buffer (1X) containing ssDNA template – **templ_19nt_A** (4.8 µM), 5´-(6-FAM)-labelled RNA primer – **FAM-RNA-prim_15nt** (4.0 µM), SFM4-3 polymerase (0.5 µM), rGTP (0.2 mM) and one of the modified **rA^CA^TP** or **rA^FT^TP** (0.05 mM). Positive control was performed under same conditions with natural rATP (0.05 mM) instead of modified **rA^X^TPs**. For negative control reaction H_2O was used instead of rATP. Reactions were carried out following the standard protocol in section 2.5.5. For dPAGE analysis of **19RNA_A** or **19RNA_A^CA^**, **19RNA_A^FT^** see Figure S20.

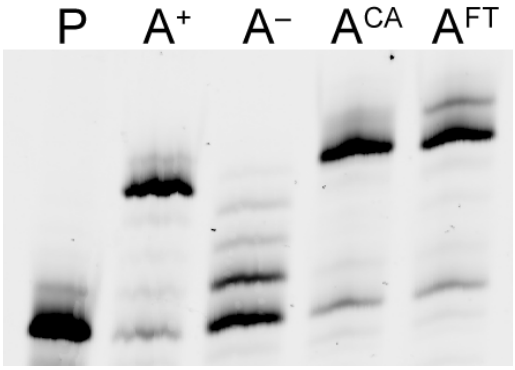

Figure S20. 22.5% dPAGE analysis of PEX reaction. (P) RNA primer; (A^{+}) positive control, mixture of rATP, rGTP; (A^{-}) negative control, mixture of rGTP and H_2O; (A^{CA}) modification, mixture of **rA^CA^TP**, rGTP; (A^{FT}) modification, mixture of **rA^FT^TP**, rGTP. FAM scan.

2.7.3 Incorporation of rA^{CA}TP at mild conditions

Reaction was performed in total volume of 10 µL in ThermoPol buffer (1X) containing ssDNA template – **templ_19nt_A** (4.8 µM), 5´-(6-FAM)-labelled RNA primer – **FAM-RNA-prim_15nt** (4.0 µM), SFM4-3 polymerase (1.0 µM), rGTP (0.4 mM) and the modified **rA^{CA}TP** (0.05 mM). Positive control was performed under same conditions with natural rATP (0.05 mM) instead of modified **rA^{CA}TP**. For negative control reaction H_2O was used instead of rATP. Reactions were carried out following the standard protocol in section 2.5.6. For dPAGE analysis of **19RNA_A** or **19RNA_A^{CA}** see Figure S21.

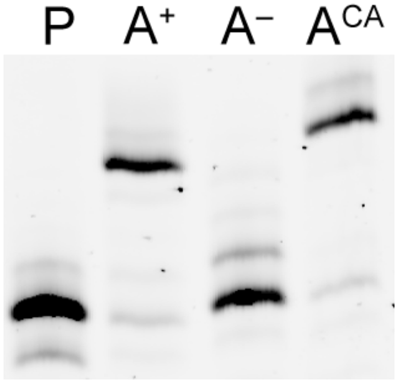

Figure S21. 22.5% dPAGE analysis of PEX reaction. (P) RNA primer; (A$^+$) positive control, mixture of rATP, rGTP; (A$^-$) negative control, mixture of rGTP and H_2O; (A^{CA}) modification, mixture of **rA^{CA}TP**, rGTP. FAM scan.

2.7.4 Incorporation of rU^{E}TP, rU^{Pent}TP, rU^{Ph}TP

Reaction was performed in total volume of 10 µL in ThermoPol buffer (1X) containing ssDNA template – **templ_19nt_U** (4.8 µM), 5´-(6-FAM)-labelled RNA primer – **FAM-RNA-prim_15nt** (4.0 µM), SFM4-3 polymerase (0.5 µM), rGTP (0.1 mM) and one of the modified **rU^{E}TP**, **rU^{Pent}TP** or **rU^{Ph}TP** (0.1 mM). Positive control was performed under same conditions with natural rUTP (0.1 mM) instead of modified **rU^{X}TPs**. For negative control reaction H_2O was used instead of rUTP. Reactions were carried out following the standard protocol in section 2.5.4. For dPAGE analysis of **19RNA_U** or **19RNA_U^{E}**, **19RNA_U^{Pent}**, **19RNA_U^{Ph}** see Figure S22.

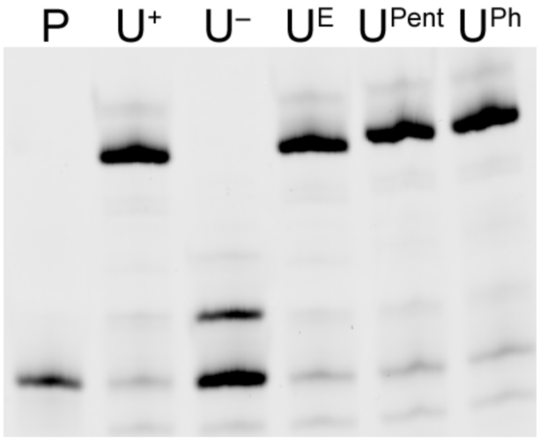

Figure S22. 22.5% dPAGE analysis of PEX reaction. (P) RNA primer; (U^{+}) positive control, mixture of rUTP, rGTP; (U^{-}) negative control, mixture of rGTP and H_2O; (U^{E}) modification, mixture of **rU^{E}TP**, rGTP; (U^{Pent}) modification, mixture of **rU^{Pent}TP**, rGTP; (U^{Ph}) modification, mixture of **rU^{Ph}TP**, rGTP. FAM scan.

2.7.5 Incorporation of rU^{Bio}TP, rU^{Dig}TP

Reaction was performed in total volume of 10 µL in ThermoPol buffer (1X) containing ssDNA template – **templ_19nt_U** (4.8 µM), 5´-(6-FAM)-labelled RNA primer – **FAM-RNA-prim_15nt** (4.0 µM), SFM4-3 polymerase (2.0 µM), rGTP (0.05 mM) and one of the modified **rU^{Bio}TP** or **rU^{Dig}TP** (0.1 mM). Positive control was performed under same conditions with natural rUTP (0.1 mM) instead of modified **rU^{X}TP**s. For negative control reaction H_2O was used instead of rUTP. Reactions were carried out following the standard protocol in section 2.5.4. For dPAGE analysis of **19RNA_U** or **19RNA_U^{Bio}**, **19RNA_U^{Dig}** see Figure S23.

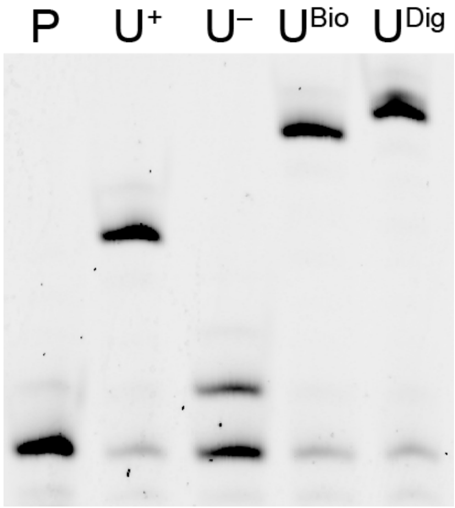

Figure S23. 22.5% dPAGE analysis of PEX reaction. (P) RNA primer; (U^{+}) positive control, mixture of rUTP, rGTP; (U^{-}) negative control, mixture of rGTP and H_2O; (U^{Bio}) modification, mixture of **rU^{Bio}TP**, rGTP; (U^{Dig}) modification, mixture of **rU^{Dig}TP**, rGTP. FAM scan.

2.7.6 Incorporation of rU^{CA}TP, rU^{FT}TP

Reaction was performed in total volume of 10 µL in ThermoPol buffer (1X) containing ssDNA template – **templ_19nt_U** (4.8 µM), 5´-(6-FAM)-labelled RNA primer – **FAM-RNA-prim_15nt** (4.0 µM), SFM4-3 polymerase (1.0 µM), rGTP (0.1 mM) and one of the modified **rU^{CA}TP** or **rU^{FT}TP** (0.05 mM). Positive control was performed under same conditions with natural rUTP (0.05 mM) instead of modified **rU^{X}TP**s. For negative control reaction H_2O was used instead of rUTP. Reactions were carried out following the standard protocol in section 2.5.5. For dPAGE analysis of **19RNA_U** or **19RNA_U^{CA}**, **19RNA_U^{FT}** see Figure S24.

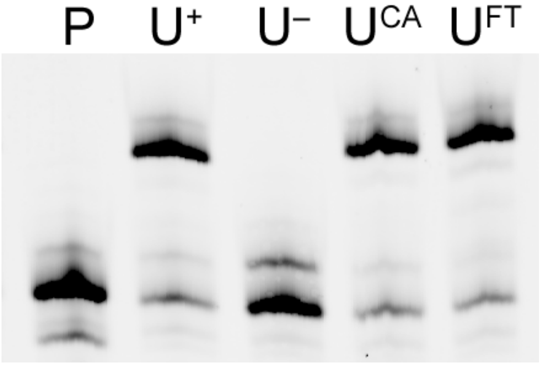

Figure S24. 22.5% dPAGE analysis of PEX reaction. (P) RNA primer; (U^{+}) positive control, mixture of rUTP, rGTP; (U$^{–}$) negative control, mixture of rGTP and H_2O; (U^{CA}) modification, mixture of **rU^{CA}TP**, rGTP; (U^{FT}) modification, mixture of **rU^{FT}TP**, rGTP. FAM scan.

2.7.7 Incorporation of rC^{E}TP, rC^{Pent}TP, rC^{Ph}TP

Reaction was performed in total volume of 10 µL in ThermoPol buffer (1X) containing ssDNA template – **templ_19nt_C** (4.8 µM), 5´-(6-FAM)-labelled RNA primer – **FAM-RNA-prim_15nt** (4.0 µM), SFM4-3 polymerase (0.5 µM), rGTP (0.1 mM) and one of the modified **rC^{E}TP**, **rC^{Pent}TP** or **rC^{Ph}TP** (0.1 mM). Positive control was performed under same conditions with natural rCTP (0.1 mM) instead of modified **rC^{X}TP**s. For negative control reaction H_2O was used instead of rCTP. Reactions were carried out following the standard protocol in section 2.5.4. For dPAGE analysis of **19RNA_C** or **19RNA_C^{E}**, **19RNA_C^{Pent}**, **19RNA_C^{Ph}** see Figure S25.

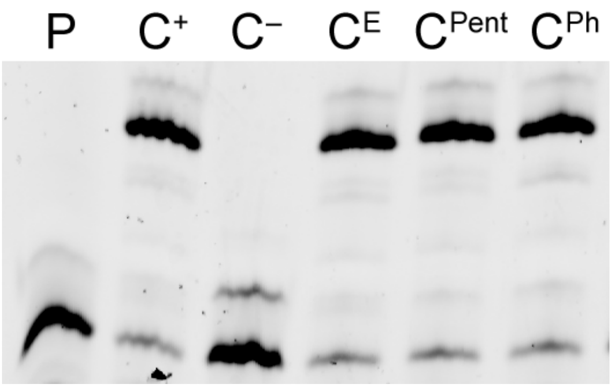

Figure S25. 22.5% dPAGE analysis of PEX reaction. (P) RNA primer; (C^+) positive control, mixture of rCTP, rGTP; ($C^–$) negative control, mixture of rGTP and H_2O; (C^E) modification, mixture of **rC^{E}TP**, rGTP; (C^{Pent}) modification, mixture of **rC^{Pent}TP**, rGTP; (C^{Ph}) modification, mixture of **rC^{Ph}TP**, rGTP. FAM scan.

2.7.8 Incorporation of rC^{mBdp}TP

Reaction was performed in total volume of 10 µL in ThermoPol buffer (1X) containing ssDNA template – **templ_19nt_C** (4.8 µM), 5´-(Cy5)-labelled RNA primer – **Cy5-RNA-prim_15nt** (4.0 µM), SFM4-3 polymerase (2.0 µM), rGTP (0.05 mM) and the modified **rC^{mBdp}TP** (0.1 mM). Positive control was performed under same conditions with natural rCTP (0.1 mM) instead of modified **rC^{mBdp}TP**. For negative control reaction H_2O was used instead of rCTP. Reactions were carried out following the standard protocol in section 2.5.4. For dPAGE analysis of **19RNA_C** or **19RNA_C^{mBdp}** see Figure S26.

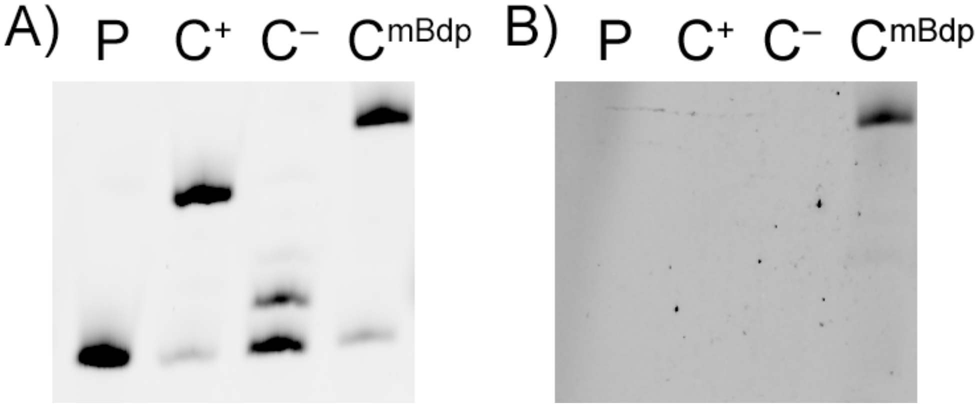

Figure S26. 22.5% dPAGE analysis of PEX reaction. (P) RNA primer; (C^+) positive control, mixture of rCTP, rGTP; ($C^–$) negative control, mixture of rGTP and H_2O; (C^{mBdp}) modification, mixture of **rC^{mBdp}TP**, rGTP. A) Cy5 scan; B) FAM scan (visualisation of mBdp-modification).

2.7.9 Incorporation of rC^{Cy5}TP

Reaction was performed in total volume of 10 µL in ThermoPol buffer (1X) containing ssDNA templare – **templ_19nt_C** (4.8 µM), 5´-(6-FAM)-labelled RNA primer – **FAM-RNA-prim_15nt** (4.0 µM), SFM4-3 polymerase (2.0 µM), rGTP (0.05 mM) and the modified **rC^{Cy5}TP** (0.1 mM). Positive control was performed under same conditions with natural rCTP (0.1 mM) instead of modified **rC^{Cy5}TP**. For negative control reaction H_2O was used instead of rCTP. Reactions were carried out following the standard protocol in section 2.5.4. For dPAGE analysis of **19RNA_C** or **19RNA_C^{Cy5}** see Figure S27.

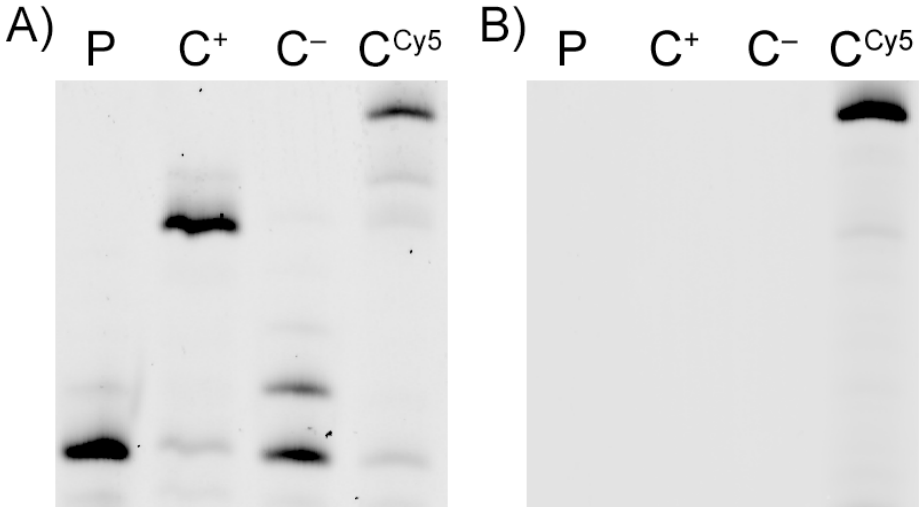

Figure S27. 22.5% dPAGE analysis of PEX reaction. (P) RNA primer; (C^{+}) positive control, mixture of rCTP, rGTP; (C^{-}) negative control, mixture of rGTP and H_2O; (C^{Cy5}) modification, mixture of **rC^{Cy5}TP**, rGTP. A) FAM scan; B) Cy5 scan (visualisation of Cy5-modification).

2.7.10 Incorporation of rC^{CA}TP, rC^{FT}TP

Reaction was performed in total volume of 10 µL in ThermoPol buffer (1X) containing ssDNA template – **templ_19nt_C** (4.8 µM), 5´-(6-FAM)-labelled RNA primer – **FAM-RNA-prim_15nt** (4.0 µM), SFM4-3 polymerase (0.5 µM), rGTP (0.2 mM) and one of the modified **rC^{CA}TP** or **rC^{FT}TP** (0.05 mM). Positive control was performed under same conditions with natural rCTP (0.05 mM) instead of modified **rC^{X}TPs**. For negative control reaction H_2O was used instead of rCTP. Reactions were carried out following the standard protocol in section 2.5.5. For dPAGE analysis of **19RNA_C** or **19RNA_C^{CA}**, **19RNA_C^{FT}** see Figure S28.

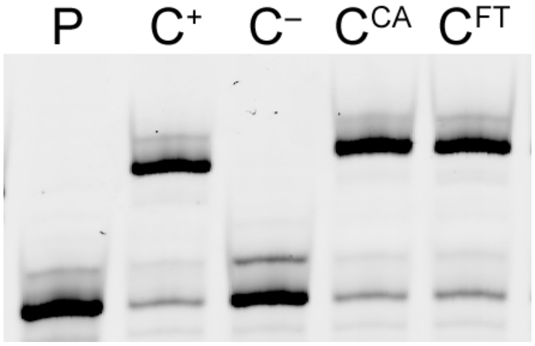

Figure S28. 22.5% dPAGE analysis of PEX reaction. (P) RNA primer; (C^+) positive control, mixture of rCTP, rGTP; ($C^–$) negative control, mixture of rGTP and H_2O; (C^{CA}) modification, mixture of **rC^{CA}TP**, rGTP; (C^{FT}) modification, mixture of **rC^{FT}TP**, rGTP. FAM scan.

2.7.11 Incorporation of rC^{CA}TP at mild conditions

Reaction was performed in total volume of 10 µL in ThermoPol buffer (1X) containing ssDNA template – **templ_19nt_C** (4.8 µM), 5´-(6-FAM)-labelled RNA primer – **FAM-RNA-prim_15nt** (4.0 µM), SFM4-3 polymerase (1.0 µM), rGTP (0.4 mM) and the modified **rC^{CA}TP** (0.05 mM). Positive control was performed under same conditions with natural rCTP (0.05 mM) instead of modified **rC^{CA}TP**. For negative control reaction H_2O was used instead of rCTP. Reactions were carried out following the standard protocol in section 2.5.6. For dPAGE analysis of **19RNA_C** or **19RNA_C^{CA}** see Figure S29.

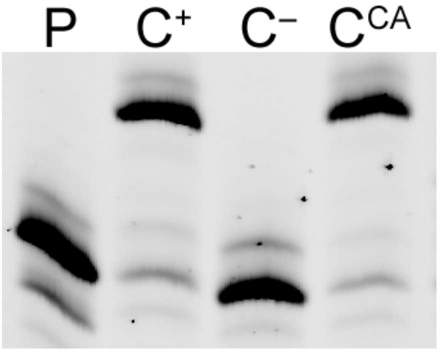

Figure S29. 22.5% dPAGE analysis of PEX reaction. (P) RNA primer; (C^+) positive control, mixture of rCTP, rGTP; ($C^–$) negative control, mixture of rGTP and H_2O; (C^{CA}) modification, mixture of **rC^{CA}TP**, rGTP. FAM scan.

2.7.12 Incorporation of rG^{E}TP, rG^{Pent}TP, rG^{Ph}TP

Reaction was performed in total volume of 10 µL in ThermoPol buffer (1X) containing ssDNA template – **templ_19nt_G** (4.8 µM), 5´-(6-FAM)-labelled RNA primer – **FAM-RNA-prim_15nt** (4.0 µM), SFM4-3 polymerase (1.5 µM), rUTP (0.4 mM) and one of the modified **rG^{E}TP**,

rG^{Pent}TP or **rG^{Ph}TP** (0.1 mM). Positive control was performed under same conditions with natural rGTP (0.1 mM) instead of modified **rG^{X}TP**s. For negative control reaction H_2O was used instead of rGTP. Reactions were carried out following the standard protocol in section 2.5.4. For dPAGE analysis of **19RNA_G** or **19RNA_G^{E}**, **19RNA_G^{Pent}**, **19RNA_G^{Ph}** see Figure S30.

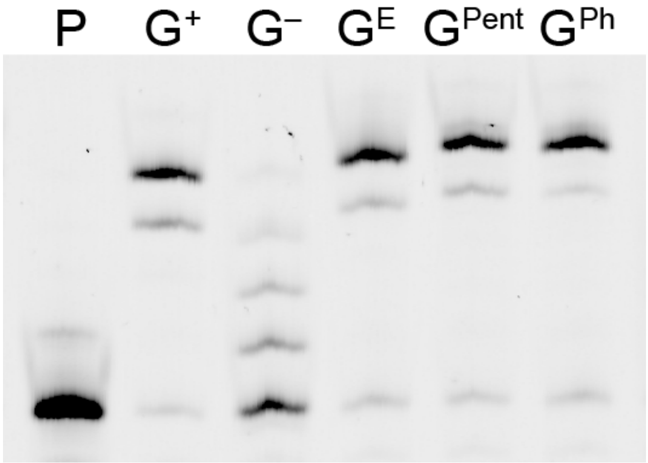

Figure S30. 22.5% dPAGE analysis of PEX reaction. (P) RNA primer; (G^{+}) positive control, mixture of rUTP, rGTP; (G^{-}) negative control, mixture of rUTP and H_2O; (G^{E}) modification, mixture of rUTP, **rG^{E}TP**; (G^{Pent}) modification, mixture of rUTP, **rG^{Pent}TP**; (G^{Ph}) modification, mixture of rUTP, **rG^{Ph}TP**. FAM scan.

2.8 Semi-preparative scale PEX reaction with templ_19nt_X (X = A, U, C, G) and TGK polymerase (incorporation of 1 modification)

2.8.1 Incorporation of rA^{E}TP, rA^{Pent}TP, rA^{Ph}TP

Reaction was performed in total volume of 50 µL in ThermoPol buffer (1X) containing ssDNA template – **templ_19nt_A** (4.8 µM), 5´-(6-FAM)-labelled RNA primer – **FAM-RNA-prim_15nt** (4.0 µM), TGK polymerase (0.5 µM), rGTP (0.1 mM) and one of the modified **rA^{E}TP**, **rA^{Pent}TP** or **rA^{Ph}TP** (0.1 mM). Reactions were carried out following the standard protocol in section 2.5.9. For mass spectrometry analysis see Figure S126 (**19RNA_A^{E}**), Figure S127 (**19RNA_A^{Pent}**), Figure S128 (**19RNA_A^{Ph}**).

2.8.2 Incorporation of rA^{CA}TP, rA^{FT}TP

Reaction was performed in total volume of 50 µL in ThermoPol buffer (1X) containing ssDNA template – **templ_19nt_A** (4.8 µM), 5´-(6-FAM)-labelled RNA primer – **FAM-RNA-prim_15nt** (4.0 µM), TGK polymerase (0.25 µM), rGTP (0.1 mM) and one of the modified **rA^{CA}TP** or **rA^{FT}TP** (0.05 mM). Reactions were carried out following the standard protocol in section 2.5.11. For mass spectrometry analysis see Figure S129, Figure S130 (**19RNA_A^{CA}**), Figure S131 (**19RNA_A^{FT}**). The sample prepared by PEX reaction with **rA^{CA}TP** was further used in digestion

reaction according to procedure in section 2.5.16, prior to UPLC analysis. For mass spectrometry analysis of the **19RNA_A^{CA}** digest see Figure S211 and Figure S261.

2.8.3 Incorporation of rA^{CA}TP at reduced time

Reaction was performed in total volume of 50 µL in ThermoPol buffer (1X) containing ssDNA template – **templ_19nt_A** (4.8 µM), 5´-(6-FAM)-labelled RNA primer – **FAM-RNA-prim_15nt** (4.0 µM), TGK polymerase (0.25 µM), rGTP (0.1 mM) and the modified **rA^{CA}TP** (0.05 mM). Reaction was carried out following the standard protocol in section 2.5.10. For mass spectrometry analysis see Figure S132 (**19RNA_A^{CA}**).

2.8.4 Incorporation of rA^{CA}TP at mild conditions

Reaction was performed in total volume of 50 µL in ThermoPol buffer (1X) containing ssDNA template – **templ_19nt_A** (4.8 µM), 5´-(6-FAM)-labelled RNA primer – **FAM-RNA-prim_15nt** (4.0 µM), TGK polymerase (0.5 µM), rGTP (0.2 mM) and the modified **rA^{CA}TP** (0.05 mM). Reaction was carried out following the standard protocol in section 2.5.12. For mass spectrometry analysis see Figure S133 (**19RNA_A^{CA}**).

2.8.5 Incorporation of rU^{E}TP, rU^{Pent}TP, rU^{Ph}TP

Reaction was performed in total volume of 50 µL in ThermoPol buffer (1X) containing ssDNA template – **templ_19nt_U** (4.8 µM), 5´-(6-FAM)-labelled RNA primer – **FAM-RNA-prim_15nt** (4.0 µM), TGK polymerase (0.75 µM), rGTP (0.05 mM) and one of the modified **rU^{E}TP**, **rU^{Pent}TP** or **rU^{Ph}TP** (0.1 mM). Reactions were carried out following the standard protocol in section 2.5.9. For mass spectrometry analysis see Figure S134 (**19RNA_U^{E}**), Figure S135 (**19RNA_U^{Pent}**), Figure S136 (**19RNA_U^{Ph}**).

2.8.6 Incorporation of rU^{Bio}TP, rU^{Dig}TP

Reaction was performed in total volume of 50 µL in ThermoPol buffer (1X) containing ssDNA template – **templ_19nt_U** (4.8 µM), 5´-(6-FAM)-labelled RNA primer – **FAM-RNA-prim_15nt** (4.0 µM), TGK polymerase (1.0 µM), rGTP (0.05 mM) and one of the modified **rU^{Bio}TP** or **rU^{Dig}TP** (0.1 mM). Reactions were carried out following the standard protocol in section 2.5.9. For mass spectrometry analysis see Figure S137 (**19RNA_U^{Bio}**), Figure S138 (**19RNA_U^{Dig}**).

2.8.7 Incorporation of rU^{CA}TP, rU^{FT}TP

Reaction was performed in total volume of 50 µL in ThermoPol buffer (1X) containing ssDNA template – **templ_19nt_U** (4.8 µM), 5´-(6-FAM)-labelled RNA primer – **FAM-RNA-prim_15nt** (4.0 µM), TGK polymerase (0.5 µM), rGTP (0.05 mM) and one of the modified **rU^{CA}TP** or **rU^{FT}TP** (0.05 mM). Reactions were carried out following the standard protocol in section 2.5.11. For mass spectrometry analysis see Figure S139 (**19RNA_U^{CA}**), Figure S140 (**19RNA_U^{FT}**).

2.8.8 Incorporation of **rC^{Me}TP**

Reaction was performed in total volume of 50 µL in ThermoPol buffer (1X) containing ssDNA template – **templ_19nt_C** (4.8 µM), RNA primer – **RNA-prim_15nt** (4.0 µM), TGK polymerase (0.5 µM), rGTP (0.1 mM) and the modified **rC^{Me}TP** (0.1 mM). Reaction was carried out following the standard protocol in section 2.5.9. For mass spectrometry analysis see Figure S141 (**19RNA_C^{Me}**).

2.8.9 Incorporation of rC^{E}TP, rC^{Pent}TP, rC^{Ph}TP

Reaction was performed in total volume of 50 µL in ThermoPol buffer (1X) containing ssDNA template – **templ_19nt_C** (4.8 µM), 5´-(6-FAM)-labelled RNA primer – **FAM-RNA-prim_15nt** (4.0 µM), TGK polymerase (0.5 µM), rGTP (0.2 mM) and one of the modified **rC^{E}TP**, **rC^{Pent}TP** or **rC^{Ph}TP** (0.1 mM). Reactions were carried out following the standard protocol in section 2.5.9. For mass spectrometry analysis see Figure S142 (**19RNA_C^{E}**), Figure S143 (**19RNA_C^{Pent}**), Figure S144 (**19RNA_C^{Ph}**).

2.8.10 Incorporation of rC^{mBdp}TP

Reaction was performed in total volume of 50 µL in ThermoPol buffer (1X) containing ssDNA template – **templ_19nt_C** (2.4 µM), 5´-(Cy5)-labelled RNA primer – **Cy5-RNA-prim_15nt** (2.0 µM), TGK polymerase (2.0 µM), rGTP (0.05 mM) and the modified **rC^{mBdp}TP** (0.1 mM). Reaction was carried out following the standard protocol in section 2.5.9. For mass spectrometry analysis see Figure S145 (**19RNA_C^{mBdp}**).

2.8.11 Incorporation of rC^{Cy5}TP

Reaction was performed in total volume of 50 µL in ThermoPol buffer (1X) containing ssDNA template – **templ_19nt_C** (4.8 µM), 5´-(6-FAM)-labelled RNA primer – **FAM-RNA-prim_15nt** (4.0 µM), TGK polymerase (2.0 µM), rGTP (0.05 mM) and the modified **rC^{Cy5}TP** (0.1 mM). Reaction was carried out following the standard protocol in section 2.5.9. For mass spectrometry analysis see Figure S146 (**19RNA_C^{Cy5}**).

2.8.12 Incorporation of rC^{CA}TP, rC^{FT}TP

Reaction was performed in total volume of 50 µL in ThermoPol buffer (1X) containing ssDNA template – **templ_19nt_C** (4.8 µM), 5´-(6-FAM)-labelled RNA primer – **FAM-RNA-prim_15nt** (4.0 µM), TGK polymerase (0.5 µM), rGTP (0.2 mM) and one of the modified **rC^{CA}TP** or **rC^{FT}TP** (0.05 mM). Reactions were carried out following the standard protocol in section 2.5.11. For mass spectrometry analysis see Figure S147, Figure S148 (**19RNA_C^{CA}**), Figure S149 (**19RNA_C^{FT}**). The sample prepared by PEX reaction with **rC^{CA}TP** was further used in digestion reaction according to procedure in section 2.5.16, prior to UPLC analysis. For mass spectrometry analysis of the **19RNA_C^{CA}** digest see Figure S212 and Figure S262.

2.8.13 Incorporation of $rC^{CA}TP$ at reduced time

Reaction was performed in total volume of 50 µL in ThermoPol buffer (1X) containing ssDNA template – **templ_19nt_C** (4.8 µM), 5´-(6-FAM)-labelled RNA primer – **FAM-RNA-prim_15nt** (4.0 µM), TGK polymerase (0.5 µM), rGTP (0.2 mM) and the modified **$rC^{CA}TP$** (0.05 mM). Reaction was carried out following the standard protocol in section 2.5.10. For mass spectrometry analysis see Figure S150 (**$19RNA\_C^{CA}$**).

2.8.14 Incorporation of $rC^{CA}TP$ at mild conditions

Reaction was performed in total volume of 50 µL in ThermoPol buffer (1X) containing ssDNA template – **templ_19nt_C** (4.8 µM), 5´-(6-FAM)-labelled RNA primer – **FAM-RNA-prim_15nt** (4.0 µM), TGK polymerase (1.5 µM), rGTP (0.4 mM) and the modified **$rC^{CA}TP$** (0.05 mM). Reaction was carried out following the standard protocol in section 2.5.12. For mass spectrometry analysis see Figure S151 (**$19RNA\_C^{CA}$**).

2.8.15 Incorporation of $rG^{E}TP$, $rG^{Pent}TP$, $rG^{Ph}TP$

Reaction was performed in total volume of 50 µL in ThermoPol buffer (1X) containing ssDNA template – **templ_19nt_G** (4.8 µM), 5´-(6-FAM)-labelled RNA primer – **FAM-RNA-prim_15nt** (4.0 µM), TGK polymerase (1.0 µM), rUTP (0.2 mM) and one of the modified **$rG^{E}TP$**, **$rG^{Pent}TP$** or **$rG^{Ph}TP$** (0.1 mM). Reactions were carried out following the standard protocol in section 2.5.9. For mass spectrometry analysis see Figure S152 (**$19RNA\_G^{E}$**), Figure S153 (**$19RNA\_G^{Pent}$**), Figure S154 (**$19RNA\_G^{Ph}$**).

2.9 Semi-preparative scale PEX reaction with templ_19nt_X (X = A, U, C, G) and SFM4-3 polymerase (incorporation of 1 modification)

2.9.1 Incorporation of $rA^{E}TP$, $rA^{Pent}TP$, $rA^{Ph}TP$ using SFM4-3 polymerase

Reaction was performed in total volume of 50 µL in ThermoPol buffer (1X) containing ssDNA template – **templ_19nt_A** (4.8 µM), 5´-(6-FAM)-labelled RNA primer – **FAM-RNA-prim_15nt** (4.0 µM), SFM4-3 polymerase (0.5 µM), rGTP (0.1 mM) and one of the modified **$rA^{E}TP$**, **$rA^{Pent}TP$** or **$rA^{Ph}TP$** (0.1 mM). Reactions were carried out following the standard protocol in section 2.5.9. For mass spectrometry analysis see Figure S155 (**$19RNA\_A^{E}$**), Figure S156 (**$19RNA\_A^{Pent}$**), Figure S157 (**$19RNA\_A^{Ph}$**).

2.9.2 Incorporation of $rA^{CA}TP$, $rA^{FT}TP$

Reaction was performed in total volume of 50 µL in ThermoPol buffer (1X) containing ssDNA template – **templ_19nt_A** (4.8 µM), 5´-(6-FAM)-labelled RNA primer – **FAM-RNA-prim_15nt** (4.0 µM), SFM4-3 polymerase (0.5 µM), rGTP (0.2 mM) and one of the modified **$rA^{CA}TP$** or **$rA^{FT}TP$** (0.05 mM). Reactions were carried out following the standard protocol in section 2.5.11.

For mass spectrometry analysis see Figure S158, Figure S159 (**19RNA_A^{CA}**), Figure S160 (**19RNA_A^{FT}**).

2.9.3 Incorporation of rA^{CA}TP at mild conditions

Reaction was performed in total volume of 50 µL in ThermoPol buffer (1X) containing ssDNA template – **templ_19nt_A** (4.8 µM), 5´-(6-FAM)-labelled RNA primer – **FAM-RNA-prim_15nt** (4.0 µM), SFM4-3 polymerase (1.0 µM), rGTP (0.4 mM) and the modified **rA^{CA}TP** (0.05 mM). Reaction was carried out following the standard protocol in section 2.5.12. For mass spectrometry analysis see Figure S161 (**19RNA_A^{CA}**).

2.9.4 Incorporation of rU^{E}TP, rU^{Pent}TP, rU^{Ph}TP

Reaction was performed in total volume of 50 µL in ThermoPol buffer (1X) containing ssDNA template – **templ_19nt_U** (4.8 µM), 5´-(6-FAM)-labelled RNA primer – **FAM-RNA-prim_15nt** (4.0 µM), SFM4-3 polymerase (0.5 µM), rGTP (0.1 mM) and one of the modified **rU^{E}TP**, **rU^{Pent}TP** or **rU^{Ph}TP** (0.1 mM). Reactions were carried out following the standard protocol in section 2.5.9. For mass spectrometry analysis see Figure S162 (**19RNA_U^{E}**), Figure S163 (**19RNA_U^{Pent}**), Figure S164 (**19RNA_U^{Ph}**).

2.9.5 Incorporation of rU^{Bio}TP, rU^{Dig}TP

Reaction was performed in total volume of 50 µL in ThermoPol buffer (1X) containing ssDNA template – **templ_19nt_U** (4.8 µM), 5´-(6-FAM)-labelled RNA primer – **FAM-RNA-prim_15nt** (4.0 µM), SFM4-3 polymerase (2.0 µM), rGTP (0.05 mM) and one of the modified **rU^{Bio}TP** or **rU^{Dig}TP** (0.1 mM). Reactions were carried out following the standard protocol in section 2.5.9. For mass spectrometry analysis see Figure S165 (**19RNA_U^{Bio}**), Figure S166 (**19RNA_U^{Dig}**).

2.9.6 Incorporation of rU^{CA}TP, rU^{FT}TP

Reaction was performed in total volume of 50 µL in ThermoPol buffer (1X) containing ssDNA template – **templ_19nt_U** (4.8 µM), 5´-(6-FAM)-labelled RNA primer – **FAM-RNA-prim_15nt** (4.0 µM), SFM4-3 polymerase (1.0 µM), rGTP (0.1 mM) and one of the modified **rU^{CA}TP** or **rU^{FT}TP** (0.05 mM). Reactions were carried out following the standard protocol in section 2.5.11. For mass spectrometry analysis see Figure S167 (**19RNA_U^{CA}**), Figure S168 (**19RNA_U^{FT}**).

2.9.7 Incorporation of rC^{E}TP, rC^{Pent}TP, rC^{Ph}TP

Reaction was performed in total volume of 50 µL in ThermoPol buffer (1X) containing ssDNA template – **templ_19nt_C** (4.8 µM), 5´-(6-FAM)-labelled RNA primer – **FAM-RNA-prim_15nt** (4.0 µM), SFM4-3 polymerase (0.5 µM), rGTP (0.1 mM) and one of the modified **rC^{E}TP**, **rC^{Pent}TP** or **rC^{Ph}TP** (0.1 mM). Reactions were carried out following the standard protocol

in section 2.5.9. For mass spectrometry analysis see Figure S169 (**19RNA_C^{E}**), Figure S170 (**19RNA_C^{Pent}**), Figure S171 (**19RNA_C^{Ph}**).

2.9.8 Incorporation of rC^{mBdp}TP

Reaction was performed in total volume of 50 µL in ThermoPol buffer (1X) containing ssDNA template – **templ_19nt_C** (4.8 µM), 5´-(Cy5)-labelled RNA primer – **Cy5-RNA-prim_15nt** (4.0 µM), SFM4-3 polymerase (2.0 µM), rGTP (0.05 mM) and the modified **rC^{mBdp}TP** (0.1 mM). Reaction was carried out following the standard protocol in section 2.5.9. For mass spectrometry analysis see Figure S172 (**19RNA_C^{mBdp}**).

2.9.9 Incorporation of rC^{Cy5}TP

Reaction was performed in total volume of 50 µL in ThermoPol buffer (1X) containing ssDNA template – **templ_19nt_C** (4.8 µM), 5´-(6-FAM)-labelled RNA primer – **FAM-RNA-prim_15nt** (4.0 µM), SFM4-3 polymerase (2.0 µM), rGTP (0.05 mM) and the modified **rC^{Cy5}TP** (0.1 mM). Reaction was carried out following the standard protocol in section 2.5.9. For mass spectrometry analysis see Figure S173 (**19RNA_C^{Cy5}**).

2.9.10 Incorporation of rC^{CA}TP, rC^{FT}TP

Reaction was performed in total volume of 50 µL in ThermoPol buffer (1X) containing ssDNA template – **templ_19nt_C** (4.8 µM), 5´-(6-FAM)-labelled RNA primer – **FAM-RNA-prim_15nt** (4.0 µM), SFM4-3 polymerase (0.5 µM), rGTP (0.2 mM) and one of the modified **rC^{CA}TP** or **rC^{FT}TP** (0.05 mM). Reactions were carried out following the standard protocol in section 2.5.11. For mass spectrometry analysis see Figure S174, Figure S175 (**19RNA_C^{CA}**), Figure S176 (**19RNA_C^{FT}**).

2.9.11 Incorporation of rC^{CA}TP at mild conditions

Reaction was performed in total volume of 50 µL in ThermoPol buffer (1X) containing ssDNA template – **templ_19nt_C** (4.8 µM), 5´-(6-FAM)-labelled RNA primer – **FAM-RNA-prim_15nt** (4.0 µM), SFM4-3 polymerase (1.0 µM), rGTP (0.4 mM) and the modified **rC^{CA}TP** (0.05 mM). Reaction was carried out following the standard protocol in section 2.5.12. For mass spectrometry analysis see Figure S177 (**19RNA_C^{CA}**).

2.9.12 Incorporation of rG^{E}TP, rG^{Pent}TP, rG^{Ph}TP

Reaction was performed in total volume of 50 µL in ThermoPol buffer (1X) containing ssDNA template – **templ_19nt_G** (4.8 µM), 5´-(6-FAM)-labelled RNA primer – **FAM-RNA-prim_15nt** (4.0 µM), SFM4-3 polymerase (1.5 µM), rUTP (0.4 mM) and one of the modified **rG^{E}TP**, **rG^{Pent}TP** or **rG^{Ph}TP** (0.1 mM). Reactions were carried out following the standard protocol

in section 2.5.9. For mass spectrometry analysis see Figure S178 (**19RNA_G^{E}**), Figure S179 (**19RNA_G^{Pent}**), Figure S180 (**19RNA_G^{Ph}**).

2.10 Analytical scale PEX reaction with 5´-(TINA)-templ_31nt and TGK polymerase (incorporation of 4 modifications)

2.10.1 Incorporation of rA^{E}TP, rA^{Pent}TP, rA^{Ph}TP

Reaction was performed in total volume of 10 µL in ThermoPol buffer (1X) containing labelled ssDNA template – **5´-(TINA)-templ_31nt** (4.8 µM), 5´-(6-FAM)-labelled RNA primer – **FAM-RNA-prim_15nt** (4.0 µM), TGK polymerase (0.25 µM), mixture of rUTP, rCTP, rGTP (0.4 mM) and one of the modified **rA^{E}TP**, **rA^{Pent}TP** or **rA^{Ph}TP** (0.2 mM). Positive control was performed under same conditions with natural rATP (0.2 mM) instead of modified **rA^{X}TP**s. For negative control reaction H_2O was used instead of rATP. Reactions were carried out following the standard protocol in section 2.5.7. For dPAGE analysis of **31RNA_4A** or **31RNA_4A^{E}**, **31RNA_4A^{Pent}**, **31RNA_4A^{Ph}** see Figure S31.

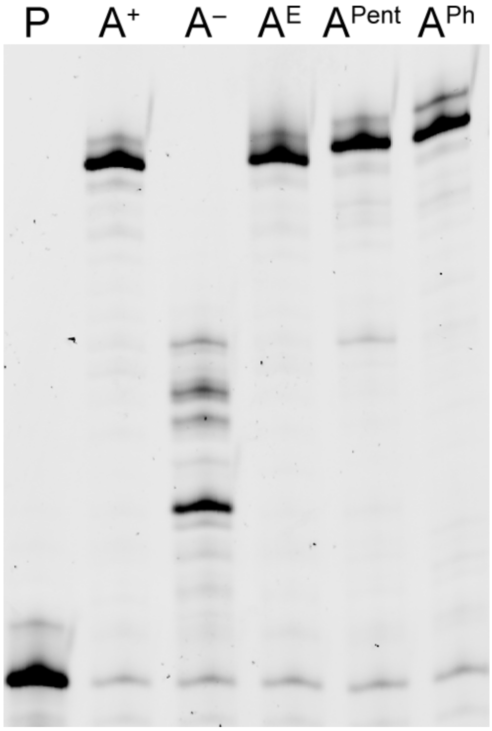

Figure S31. 22.5% dPAGE analysis of PEX reaction. (P) RNA primer; (A^{+}) positive control, all natural rNTPs; (A^{-}) negative control, mixture of rUTP, rCTP, rGTP and H_2O; (A^{E}) modification, mixture of **rA^{E}TP**, rUTP, rCTP, rGTP; (A^{Pent}) modification, mixture of **rA^{Pent}TP**, rUTP, rCTP, rGTP; (A^{Ph}) modification, mixture of **rA^{Ph}TP**, rUTP, rCTP, rGTP. FAM scan.

2.10.2 Incorporation of $rU^{E}TP$, $rU^{Pent}TP$, $rU^{Ph}TP$

Reaction was performed in total volume of 10 µL in ThermoPol buffer (1X) containing labelled ssDNA template – **5´-(TINA)-templ_31nt** (4.8 µM), 5´-(6-FAM)-labelled RNA primer – **FAM-RNA-prim_15nt** (4.0 µM), TGK polymerase (0.5 µM), mixture of rATP, rCTP, rGTP (0.4 mM) and one of the modified **$rU^{E}TP$**, **$rU^{Pent}TP$** or **$rU^{Ph}TP$** (0.2 mM). Positive control was performed under same conditions with natural rUTP (0.2 mM) instead of modified **$rU^{X}TP$**s. For negative control reaction H_2O was used instead of rUTP. Reactions were carried out following the standard protocol in section 2.5.7. For dPAGE analysis of **31RNA_4U** or **31RNA_4U^{E}**, **31RNA_4U^{Pent}**, **31RNA_4U^{Ph}** see Figure S32.

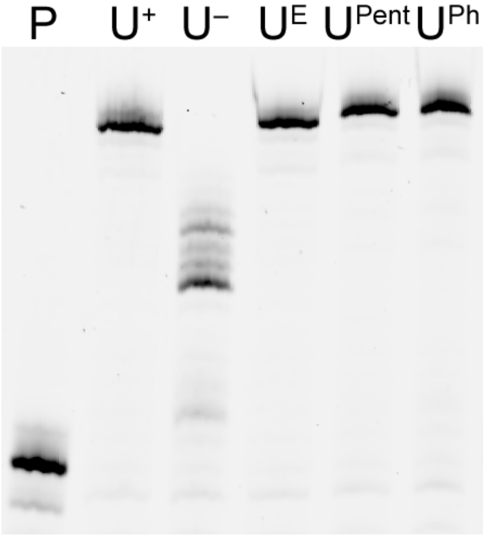

Figure S32. 22.5% dPAGE analysis of PEX reaction. (P) RNA primer; (U^{+}) positive control, all natural rNTPs; (U^{-}) negative control, mixture of rATP, rCTP, rGTP and H_2O; (U^{E}) modification, mixture of rATP, **$rU^{E}TP$**, rCTP, rGTP; (U^{Pent}) modification, mixture of rATP, **$rU^{Pent}TP$**, rCTP, rGTP; (U^{Ph}) modification, mixture of rATP, **$rU^{Ph}TP$**, rCTP, rGTP. FAM scan.

2.10.3 Incorporation of $rU^{Bio}TP$, $rU^{Dig}TP$

Reaction was performed in total volume of 10 µL in ThermoPol buffer (1X) containing labelled ssDNA template – **5´-(TINA)-templ_31nt** (4.8 µM), 5´-(6-FAM)-labelled RNA primer – **FAM-RNA-prim_15nt** (4.0 µM), TGK polymerase (1.0 µM), mixture of rATP, rCTP, rGTP (0.2 mM) and one of the modified **$rU^{Bio}TP$** or **$rU^{Dig}TP$** (0.2 mM). Positive control was performed under same conditions with natural rUTP (0.2 mM) instead of modified **$rU^{X}TP$**s. For negative control reaction H_2O was used instead of rUTP. Reactions were carried out following the standard protocol in section 2.5.7. For dPAGE analysis of **31RNA_4U** or **31RNA_4U^{Bio}**, **31RNA_4U^{Dig}** see Figure S33.

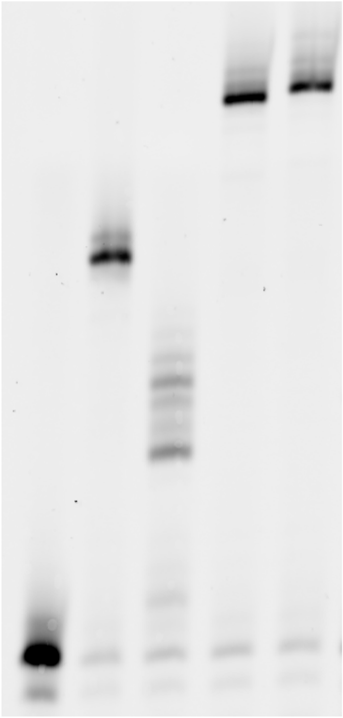

Figure S33. 22.5% dPAGE analysis of PEX reaction. (P) RNA primer; (U^{+}) positive control, all natural rNTPs; ($U^{–}$) negative control, mixture of rATP, rCTP, rGTP and H_2O; (U^{Bio}) modification, mixture of rATP, **rU^{Bio}TP**, rCTP, rGTP; (U^{Dig}) modification, mixture of rATP, **rU^{Dig}TP**, rCTP, rGTP. FAM scan.

2.10.4 Incorporation of rC^{Me}TP

Reaction was performed in total volume of 10 µL in ThermoPol buffer (1X) containing ssDNA template – **5´-(TINA)-templ_31nt** (4.8 µM), 5´-(6-FAM)-labelled RNA primer – **FAM-RNA-prim_15nt** (4.0 µM), TGK polymerase (0.5 µM), mixture of rATP, rUTP, rGTP (0.4 mM) and the modified **rC^{Me}TP** (0.2 mM). Positive control was performed under same conditions with natural rCTP (0.2 mM) instead of modified **rC^{Me}TP**. For negative control reaction H_2O was used instead of rCTP. Reactions were carried out following the standard protocol in section 2.5.7. For dPAGE analysis of **31RNA_4C** or **31RNA_4C^{Me}** see Figure S34.

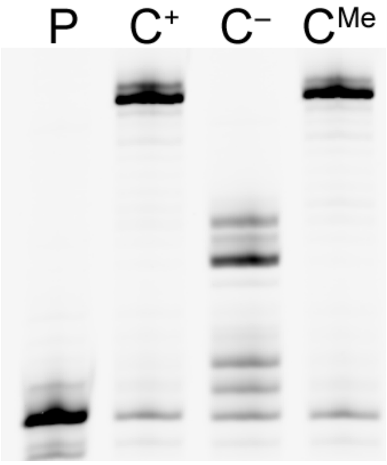

Figure S34. 22.5% dPAGE analysis of PEX reaction. (P) RNA primer; (C^+) positive control, all natural rNTPs; ($C^–$) negative control, mixture of rATP, rUTP, rGTP and H_2O; (C^{Me}) modification, mixture of rATP, rUTP, **rC^{Me}TP**, rGTP. FAM scan.

2.10.5 Incorporation of rC^{E}TP, rC^{Pent}TP, rC^{Ph}TP

Reaction was performed in total volume of 10 µL in ThermoPol buffer (1X) containing labelled ssDNA template – **5´-(TINA)-templ_31nt** (4.8 µM), 5´-(6-FAM)-labelled RNA primer – **FAM-RNA-prim_15nt** (4.0 µM), TGK polymerase (0.5 µM), mixture of rATP, rUTP, rGTP (0.4 mM) and one of the modified **rC^{E}TP**, **rC^{Pent}TP** or **rC^{Ph}TP** (0.2 mM). Positive control was performed under same conditions with natural rCTP (0.2 mM) instead of modified **rC^{X}TP**s. For negative control reaction H_2O was used instead of rCTP. Reactions were carried out following the standard protocol in section 2.5.7. For dPAGE analysis of **31RNA_4C** or **31RNA_4C^{E}**, **31RNA_4C^{Pent}**, **31RNA_4C^{Ph}** see Figure S35.

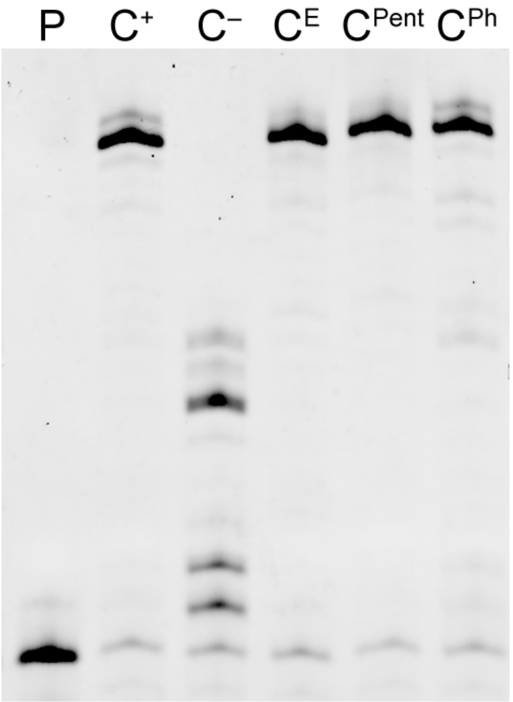

Figure S35. 22.5% dPAGE analysis of PEX reaction. (P) RNA primer; (C^+) positive control, all natural rNTPs; ($C^–$) negative control, mixture of rATP, rUTP, rGTP and H_2O; (C^E) modification, mixture of rATP, rUTP, **rC^{E}TP**, rGTP; (C^{Pent}) modification, mixture of rATP, rUTP, **rC^{Pent}TP**, rGTP; (C^{Ph}) modification, mixture of rATP, rUTP, **rC^{Ph}TP**, rGTP. FAM scan.

2.10.6 Incorporation of rC^{mBdp}TP

Reaction was performed in total volume of 10 µL in ThermoPol buffer (1X) containing labelled ssDNA template – **5´-(TINA)-templ_31nt** (2.4 µM), 5´-(Cy5)-labelled RNA primer – **Cy5-RNA-prim_15nt** (2.0 µM), TGK polymerase (2.0 µM), mixture of rATP, rUTP, rGTP (0.4 mM) and the modified **rC^{mBdp}TP** (0.8 mM). Positive control was performed under same conditions with natural rCTP (0.4 mM) instead of modified **rC^{mBdp}TP**. For negative control reaction H_2O was used instead of rCTP. Reactions were carried out following the standard protocol in section 2.5.7. For dPAGE analysis of **31RNA_4C** or **31RNA_4C^{mBdp}** see Figure S36.

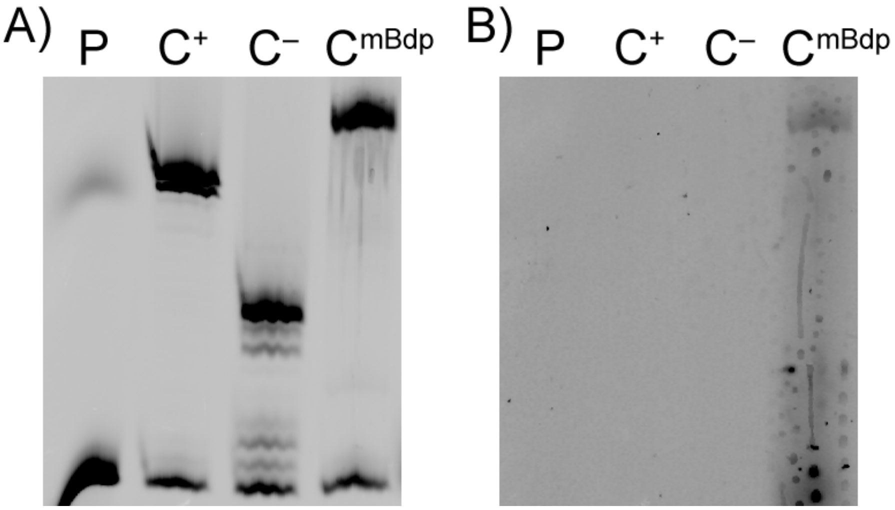

Figure S36. 22.5% dPAGE analysis of PEX reaction. (P) RNA primer; (C^+) positive control, all natural rNTPs; ($C^–$) negative control, mixture of rATP, rUTP, rGTP and H_2O; (C^{mBdp}) modification, mixture of rATP, rUTP, **rC^{mBdp}TP**, rGTP. A) Cy5 scan; B) FAM scan (visualisation of mBdp-modification).

2.10.7 Incorporation of rG^{E}TP, rG^{Pent}TP, rG^{Ph}TP

Reaction was performed in total volume of 10 µL in ThermoPol buffer (1X) containing labelled ssDNA template – **5´-(TINA)-templ_31nt** (4.8 µM), 5´-(6-FAM)-labelled RNA primer – **FAM-RNA-prim_15nt** (4.0 µM), TGK polymerase (1.5 µM), mixture of rATP, rUTP, rCTP (0.8 mM) and one of the modified **rG^{E}TP**, **rG^{Pent}TP** or **rG^{Ph}TP** (0.4 mM). Positive control was performed under same conditions with natural rGTP (0.4 mM) instead of modified **rG^{X}TP**s. For negative control reaction H_2O was used instead of rGTP. Reactions were carried out following the standard protocol in section 2.5.7. For dPAGE analysis of **31RNA_4G** or **31RNA_4G^E**, **31RNA_4G^{Pent}**, **31RNA_4G^{Ph}** see Figure S37.

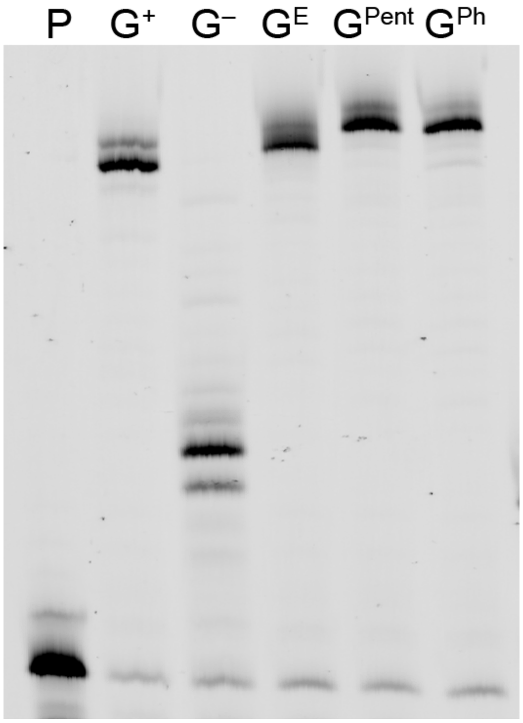

Figure S37. 22.5% dPAGE analysis of PEX reaction. (P) RNA primer; (G^+) positive control, all natural rNTPs; (G^-) negative control, mixture of rATP, rUTP, rCTP and H_2O; (G^E) modification, mixture of rATP, rUTP, rCTP, **rG^ETP**; (G^{Pent}) modification, mixture of rATP, rUTP, rCTP, **rG^{Pent}TP**; (G^{Ph}) modification, mixture of rATP, rUTP, rCTP, **rG^{Ph}TP**. FAM scan.

2.11 Analytical scale PEX reaction with 5´-(TINA)-templ_31nt and SFM4-3 polymerase (incorporation of 4 modifications)

2.11.1 Incorporation of rA^ETP, rA^{Pent}TP, rA^{Ph}TP

Reaction was performed in total volume of 10 µL in ThermoPol buffer (1X) containing labelled ssDNA template – **5´-(TINA)-templ_31nt** (4.8 µM), 5´-(6-FAM)-labelled RNA primer – **FAM-RNA-prim_15nt** (4.0 µM), SFM4-3 polymerase (0.25 µM), mixture of rUTP, rCTP, rGTP (0.4 mM) and one of the modified **rA^ETP**, **rA^{Pent}TP** or **rA^{Ph}TP** (0.2 mM). Positive control was performed under same conditions with natural rATP (0.2 mM) instead of modified **rA^XTP**s. For negative control reaction H_2O was used instead of rATP. Reactions were carried out following the standard protocol in section 2.5.7. For dPAGE analysis of **31RNA_4A** or **31RNA_4A^E**, **31RNA_4A^{Pent}**, **31RNA_4A^{Ph}** see Figure S38.

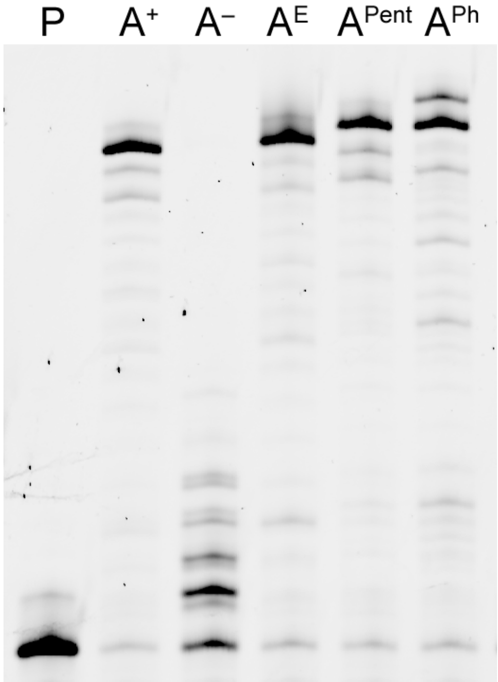

Figure S38. 22.5% dPAGE analysis of PEX reaction. (P) RNA primer; (A^{+}) positive control, all natural rNTPs; (A^{-}) negative control, mixture of rUTP, rCTP, rGTP and H_2O; (A^{E}) modification, mixture of **rA^{E}TP**, rUTP, rCTP, rGTP; (A^{Pent}) modification, mixture of **rA^{Pent}TP**, rUTP, rCTP, rGTP; (A^{Ph}) modification, mixture of **rA^{Ph}TP**, rUTP, rCTP, rGTP. FAM scan.

2.11.2 Incorporation of rU^{E}TP, rU^{Pent}TP, rU^{Ph}TP

Reaction was performed in total volume of 10 µL in ThermoPol buffer (1X) containing labelled ssDNA template – **5´-(TINA)-templ_31nt** (4.8 µM), 5´-(6-FAM)-labelled RNA primer – **FAM-RNA-prim_15nt** (4.0 µM), SFM4-3 polymerase (0.75 µM), mixture of rATP, rCTP, rGTP (0.4 mM) and one of the modified **rU^{E}TP**, **rU^{Pent}TP** or **rU^{Ph}TP** (0.2 mM). Positive control was performed under same conditions with natural rUTP (0.2 mM) instead of the modified **rU^{X}TP**s. For negative control reaction H_2O was used instead of rUTP. Reactions were carried out following the standard protocol in section 2.5.7. For dPAGE analysis of **31RNA_4U** or **31RNA_4U^{E}**, **31RNA_4U^{Pent}**, **31RNA_4U^{Ph}** see Figure S39.

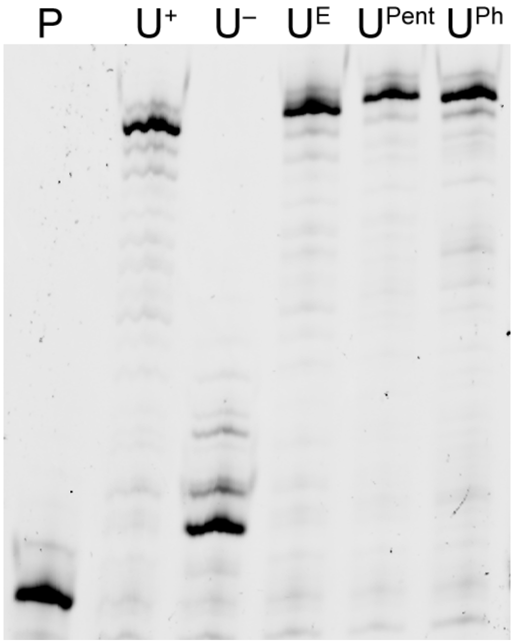

Figure S39. 22.5% dPAGE analysis of PEX reaction. (P) RNA primer; (U^{+}) positive control, all natural rNTPs; (U^{-}) negative control, mixture of rATP, rCTP, rGTP and H_2O; (U^{E}) modification, mixture of rATP, **rU^{E}TP**, rCTP, rGTP; (U^{Pent}) modification, mixture of rATP, **rU^{Pent}TP**, rCTP, rGTP; (U^{Ph}) modification, mixture of rATP, **rU^{Ph}TP**, rCTP, rGTP. FAM scan.

2.11.3 Incorporation of rU^{Bio}TP, rU^{Dig}TP

Reaction was performed in total volume of 10 µL in ThermoPol buffer (1X) containing labelled ssDNA template – **5´-(TINA)-templ_31nt** (4.8 µM), 5´-(6-FAM)-labelled RNA primer – **FAM-RNA-prim_15nt** (4.0 µM), SFM4-3 polymerase (1.0 µM), mixture of rATP, rCTP, rGTP (0.2 mM) and one of the modified **rU^{Bio}TP** or **rU^{Dig}TP** (0.2 mM). Positive control was performed under same conditions with natural rUTP (0.2 mM) instead of modified **rU^{X}TP**s. For negative control reaction H_2O was used instead of rUTP. Reactions were carried out following the standard protocol in section 2.5.7. For dPAGE analysis of **31RNA_4U** or **31RNA_4U^{Bio}**, **31RNA_4U^{Dig}** see Figure S40.

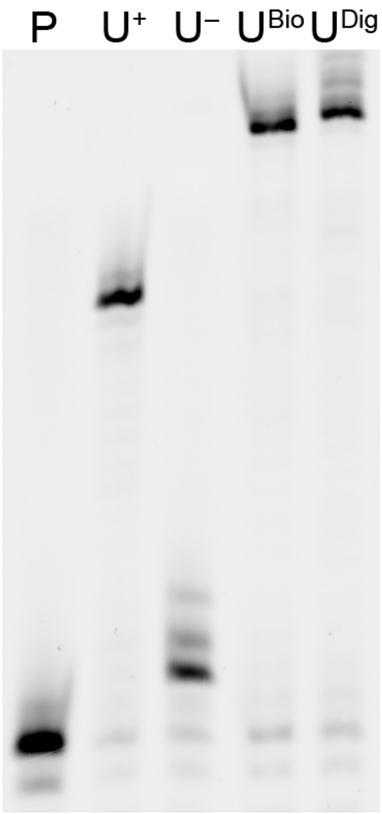

Figure S40. 22.5% dPAGE analysis of PEX reaction. (P) RNA primer; (U^+) positive control, all natural rNTPs; ($U^–$) negative control, mixture of rATP, rCTP, rGTP and H_2O; (U^{Bio}) modification, mixture of rATP, **rU^{Bio}TP**, rCTP, rGTP; (U^{Dig}) modification, mixture of rATP, **rU^{Dig}TP**, rCTP, rGTP. FAM scan.

2.11.4 Incorporation of rC^{E}TP, rC^{Pent}TP, rC^{Ph}TP

Reaction was performed in total volume of 10 µL in ThermoPol buffer (1X) containing labelled ssDNA template **5´-(TINA)-templ_31nt** (4.8 µM), 5´-(6-FAM)-labelled RNA primer – **FAM-RNA-prim_15nt** (4.0 µM), SFM4-3 polymerase (0.5 µM), mixture of rATP, rUTP, rGTP (0.4 mM) and one of the modified **rC^{E}TP**, **rC^{Pent}TP** or **rC^{Ph}TP** (0.2 mM). Positive control was performed under same conditions with natural rCTP (0.2 mM) instead of modified **rC^{X}TP**s. For negative control reaction H_2O was used instead of rCTP. Reactions were carried out following the standard protocol in section 2.5.7. For dPAGE analysis of **31RNA_4C** or **31RNA_4C^{E}**, **31RNA_4C^{Pent}**, **31RNA_4C^{Ph}** see Figure S41.

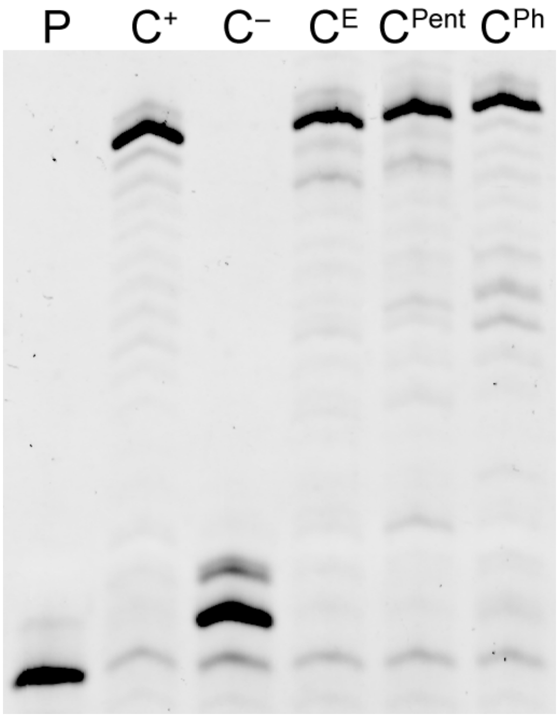

Figure S41. 22.5% dPAGE analysis of PEX reaction. (P) RNA primer; (C^+) positive control, all natural rNTPs; (C^-) negative control, mixture of rATP, rUTP, rGTP and H_2O; (C^E) modification, mixture of rATP, rUTP, **rC^{E}TP**, rGTP; (C^{Pent}) modification, mixture of rATP, rUTP, **rC^{Pent}TP**, rGTP; (C^{Ph}) modification, mixture of rATP, rUTP, **rC^{Ph}TP**, rGTP. FAM scan.

2.11.5 Incorporation of rC^{mBdp}TP

Reaction was performed in total volume of 10 µL in ThermoPol buffer (1X) containing labelled ssDNA template – **5´-(TINA)-templ_31nt** (2.4 µM), 5´-(Cy5)-labelled RNA primer – **Cy5-RNA-prim_15nt** (2.0 µM), SFM4-3 polymerase (2.0 µM), mixture of rATP, rUTP, rGTP (0.4 mM) and the modified **rC^{mBdp}TP** (0.8 mM). Positive control was performed under same conditions with natural rCTP (0.4 mM) instead of modified **rC^{mBdp}TP**. For negative control reaction H_2O was used instead of rCTP. Reactions were carried out following the standard protocol in section 2.5.7. For dPAGE analysis of **31RNA_4C** or **31RNA_4C^{mBdp}** see Figure S42.

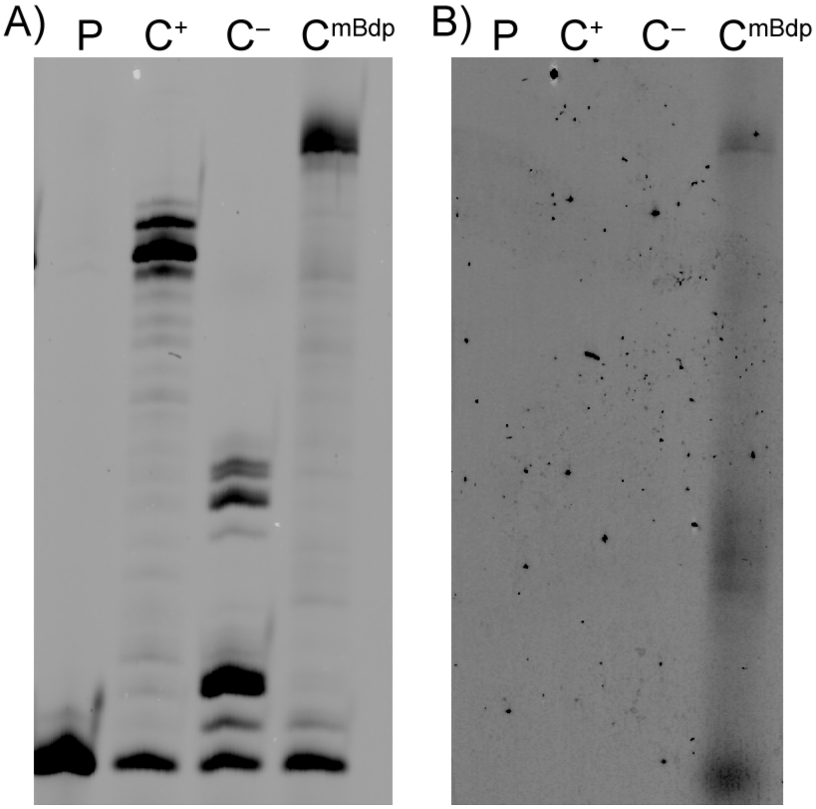

Figure S42. 22.5% dPAGE analysis of PEX reaction. (P) RNA primer; (C^{+}) positive control, all natural rNTPs; (C^{-}) negative control, mixture of rATP, rUTP, rGTP and H_2O; (C^{mBdp}) modification, mixture of rATP, rUTP, **rC^{mBdp}TP**, rGTP. A) Cy5 scan; B) FAM scan (visualisation of mBdp-modification).

2.11.6 Incorporation of rG^{E}TP, rG^{Pent}TP, rG^{Ph}TP

Reaction was performed in total volume of 10 µL in ThermoPol buffer (1X) containing labelled ssDNA template – **5´-(TINA)-templ_31nt** (4.8 µM), 5´-(6-FAM)-labelled RNA primer – **FAM-RNA-prim_15nt** (4.0 µM), SFM4-3 polymerase (2.0 µM), mixture of rATP, rUTP, rCTP (1.0 mM) and one of the modified **rG^{E}TP**, **rG^{Pent}TP** or **rG^{Ph}TP** (0.4 mM). Positive control was performed under same conditions with natural rGTP (0.4 mM) instead of modified **rG^{X}TP**s. For negative control reaction H_2O was used instead of rGTP. Reactions were carried out following the standard protocol in section 2.5.7. For dPAGE analysis of **31RNA_4G** or **31RNA_4G^{E}**, **31RNA_4G^{Pent}**, **31RNA_4G^{Ph}** see Figure S43.

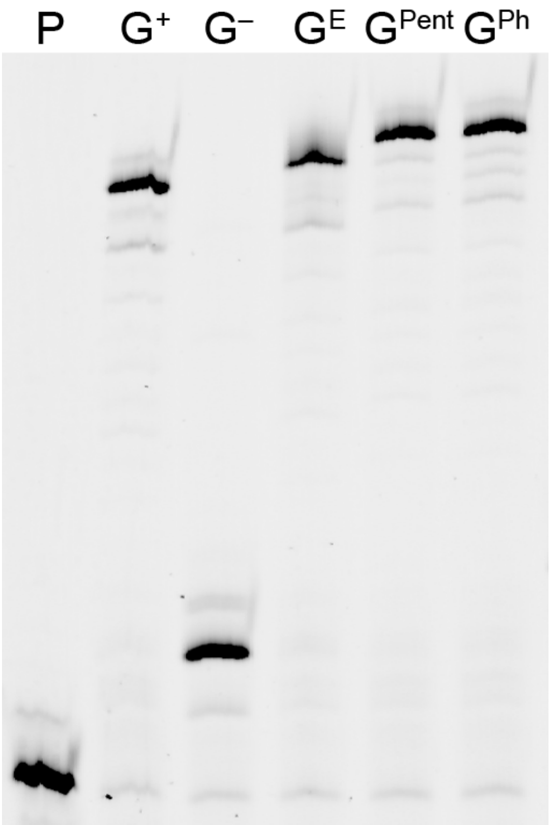

Figure S43. 22.5% dPAGE analysis of PEX reaction. (P) RNA primer; (G^+) positive control, all natural rNTPs; ($G^–$) negative control, mixture of rATP, rUTP, rCTP and H_2O; (G^E) modification, mixture of rATP, rUTP, rCTP, **rG^E^TP**; (G^{Pent}) modification, mixture of rATP, rUTP, rCTP, **rG^{Pent}TP**; (G^{Ph}) modification, mixture of rATP, rUTP, rCTP, **rG^{Ph}TP**. FAM scan.

2.12 Semi-preparative scale PEX reaction with 5´-(TINA)-templ_31nt or 5´-(dual-Bio)-templ_31nt and preparative scale PEX with templ_31nt and TGK polymerase (incorporation of 4 modifications)

2.12.1 Incorporation of rA^{E}TP, rA^{Pent}TP, rA^{Ph}TP

Reaction was performed in total volume of 50 µL in ThermoPol buffer (1X) containing labelled ssDNA template – **5´-(TINA)-templ_31nt** (4.8 µM), 5´-(6-FAM)-labelled RNA primer – **FAM-RNA-prim_15nt** (4.0 µM), TGK polymerase (0.25 µM), mixture of rUTP, rCTP, rGTP (0.4 mM) and one of the modified **rA^{E}TP**, **rA^{Pent}TP** or **rA^{Ph}TP** (0.2 mM). Reactions were carried out following the standard protocol in section 2.5.13. For mass spectrometry analysis see Figure S181 (**31RNA_4A^E**), Figure S182 (**31RNA_4A^{Pent}**), Figure S183 (**31RNA_4A^{Ph}**).

2.12.2 Incorporation of rU^{E}TP, rU^{Pent}TP, rU^{Ph}TP

Reaction was performed in total volume of 50 µL in ThermoPol buffer (1X) containing labelled ssDNA template – **5´-(TINA)-templ_31nt** (4.8 µM), 5´-(6-FAM)-labelled RNA primer – **FAM-**

RNA-prim_15nt (4.0 µM), TGK polymerase (0.5 µM), mixture of rATP, rCTP, rGTP (0.4 mM) and one of the modified **rU^{E}TP**, **rU^{Pent}TP** or **rU^{Ph}TP** (0.2 mM). Reactions were carried out following the standard protocol in section 2.5.13. For mass spectrometry analysis see Figure S184 (**31RNA_4U^{E}**), Figure S185 (**31RNA_4U^{Pent}**), Figure S186, Figure S187 (**31RNA_4U^{Ph}**).

2.12.3 Incorporation of rU^{Bio}TP, rU^{Dig}TP

Reaction was performed in total volume of 50 µL in ThermoPol buffer (1X) containing labelled ssDNA template – **5´-(TINA)-templ_31nt** (4.8 µM), 5´-(6-FAM)-labelled RNA primer – **FAM-RNA-prim_15nt** (4.0 µM), TGK polymerase (1.0 µM), mixture of rATP, rCTP, rGTP (0.2 mM) and one of the modified **rU^{Bio}TP** or **rU^{Dig}TP** (0.2 mM). Reactions were carried out following the standard protocol in section 2.5.13. For mass spectrometry analysis see Figure S188 (**31RNA_4U^{Bio}**), Figure S189 (**31RNA_4U^{Dig}**).

2.12.4 Incorporation of rC^{Me}TP

Reaction was performed in total volume of 50 µL in ThermoPol buffer (1X) containing ssDNA template – **5´-(TINA)-templ_31nt** (4.8 µM), RNA primer – **RNA-prim_15nt** (4.0 µM), TGK polymerase (0.5 µM), mixture of rATP, rUTP, rGTP (0.4 mM) and the modified **rC^{Me}TP** (0.2 mM). Reaction was carried out following the standard protocol in section 2.5.13. For mass spectrometry analysis see Figure S190 (**31RNA_4C^{Me}**).

2.12.5 Incorporation of rC^{E}TP, rC^{Pent}TP, rC^{Ph}TP

Reaction was performed in total volume of 50 µL in ThermoPol buffer (1X) containing labelled ssDNA template – **5´-(TINA)-templ_31nt** (4.8 µM), 5´-(6-FAM)-labelled RNA primer – **FAM-RNA-prim_15nt** (4.0 µM), TGK polymerase (0.5 µM), mixture of rATP, rUTP, rGTP (0.4 mM) and one of the modified **rC^{E}TP**, **rC^{Pent}TP** or **rC^{Ph}TP** (0.2 mM). Reactions were carried out following the standard protocol in section 2.5.13. For mass spectrometry analysis see Figure S191 (**31RNA_4C^{E}**), Figure S192 (**31RNA_4C^{Pent}**), Figure S193 (**31RNA_4C^{Ph}**).

2.12.6 Incorporation of rC^{mBdp}TP

Reaction was performed in total volume of 100 µL in ThermoPol buffer (1X) containing labelled ssDNA template – **5´-(dual-Bio)-templ_31nt** (2.4 µM), RNA primer – **RNA-prim_15nt** (2.0 µM), TGK polymerase (2.0 µM), mixture of rATP, rUTP, rGTP (0.4 mM) and the modified **rC^{mBdp}TP** (0.8 mM). Reaction was carried out following the standard protocol in section 2.5.14. For mass spectrometry analysis see Figure S213 and Figure S214 (**31RNA_4C^{mBdp}**).

2.12.7 Incorporation of rG^{E}TP, rG^{Pent}TP, rG^{Ph}TP

Reaction was performed in total volume of 50 µL in ThermoPol buffer (1X) containing labelled ssDNA template – **5´-(TINA)-templ_31nt** (4.8 µM), 5´-(6-FAM)-labelled RNA primer – **FAM-**

RNA-prim_15nt (4.0 µM), TGK polymerase (1.5 µM), mixture of rATP, rUTP, rCTP (0.8 mM) and one of the modified **rG^{E}TP**, **rG^{Pent}TP** or **rG^{Ph}TP** (0.4 mM). Reactions were carried out following the standard protocol in section 2.5.13. For mass spectrometry analysis see Figure S194 (**31RNA_4G^{E}**), Figure S195 (**31RNA_4G^{Pent}**), Figure S196 (**31RNA_4G^{Ph}**).

2.12.8 Preparative (1 nmol scale) PEX reaction with natural rNTPs

Reaction was performed in duplicate in a total volume of 250 µL in ThermoPol buffer (1X) containing ssDNA template – **templ_31nt** (4.8 µM), 5´-(6-FAM)-labelled RNA primer – **FAM-RNA-prim_15nt** (4.0 µM), TGK polymerase (0.5 µM), mixture of rATP, rUTP, rCTP, rGTP (0.4 mM). Reactions were carried out following the standard protocol in section 2.5.13. The purification was performed with QIAquick nucleotide removal kit according to standard supplier´s protocol. After purifcation, 1 µL aliquot was removed, dilluted with H_2O (20X) and combined with 20 µL of 2X stop solution (95% [v/v] formamide, 0.5 mM EDTA, 0.025% [w/v] bromophenol blue, 0.025% [w/v] SDS in H_2O), denatured by heating at 95 °C for 2 min and then immediately cooled on ice. Aliquots of the denatured samples (10 µL) were subjected to vertical gel electrophoresis. For mass spectrometry analysis see Figure S241 – Figure S244 (**31RNA_nat**) and for quantification of reaction yield and purity of the final product see Figure S44 and Table S8.

2.12.9 Preparative (1 nmol scale) PEX reaction with a mixture of rA^{E}TP, rUTP, rCTP, rGTP

Reaction was performed in duplicate in a total volume of 250 µL in ThermoPol buffer (1X) containing ssDNA template – **templ_31nt** (4.8 µM), 5´-(6-FAM)-labelled RNA primer – **FAM-RNA-prim_15nt** (4.0 µM), TGK polymerase (0.25 µM), mixture of rUTP, rCTP, rGTP (0.4 mM) and the modified **rA^{E}TP** (0.2 mM). Reactions were carried out following the standard protocol in section 2.5.13. The purification was performed with QIAquick nucleotide removal kit according to standard supplier´s protocol. After puriifcation, 1 µL aliquot was removed, dilluted with H_2O (20X) and combined with 20 µL of 2X stop solution (95% [v/v] formamide, 0.5 mM EDTA, 0.025% [w/v] bromophenol blue, 0.025% [w/v] SDS in H_2O), denatured by heating at 95 °C for 2 min and then immediately cooled on ice. Aliquots of the denatured samples (10 µL) were subjected to vertical gel electrophoresis. For mass spectrometry analysis see Figure S245 – Figure S248 (**31RNA_4A^{E}**) and for quantification of reaction yield and purity of the final product see Figure S44 and Table S8.

2.12.10 Preparative (1 nmol scale) PEX reaction with a mixture of rATP, rU^{Bio}TP, rCTP, rGTP

Reaction was performed in duplicate in a total volume of 250 µL in ThermoPol buffer (1X) containing ssDNA template – **templ_31nt** (4.8 µM), 5´-(6-FAM)-labelled RNA primer – **FAM-RNA-prim_15nt** (4.0 µM), TGK polymerase (1.0 µM), mixture of rATP, rCTP, rGTP (0.2 mM) and the modified **rU^{Bio}TP** (0.2 mM). Reactions were carried out following the standard protocol

in section 2.5.13. The purification was performed with QIAquick nucleotide removal kit according to standard supplier´s protocol. After puriifcation, 1 µL aliquot was removed, dilluted with H_2O (20X) and combined with 20 µL of 2X stop solution (95% [v/v] formamide, 0.5 mM EDTA, 0.025% [w/v] bromophenol blue, 0.025% [w/v] SDS in H_2O), denatured by heating at 95 °C for 2 min and then immediately cooled on ice. Aliquots of the denatured samples (10 µL) were subjected to vertical gel electrophoresis. For mass spectrometry analysis see Figure S249 – Figure S252 (**31RNA_4U^{Bio}**) and for quantification of reaction yield and purity of the final product see Figure S44 and Table S8.

2.12.11 Preparative (1 nmol scale) PEX reaction with a mixture of rATP, rUTP, rC^{Me}TP, rGTP

Reaction was performed in duplicate in a total volume of 250 µL in ThermoPol buffer (1X) containing ssDNA template – **templ_31nt** (4.8 µM), 5´-(6-FAM)-labelled RNA primer – **FAM-RNA-prim_15nt** (4.0 µM), TGK polymerase (0.5 µM), mixture of rATP, rUTP, rGTP (0.4 mM) and the modified **rC^{Me}TP** (0.2 mM). Reactions were carried out following the standard protocol in section 2.5.13. The purification was performed with QIAquick nucleotide removal kit according to standard supplier´s protocol. After puriifcation, 1 µL aliquot was removed, dilluted with H_2O (20X) and combined with 20 µL of 2X stop solution (95% [v/v] formamide, 0.5 mM EDTA, 0.025% [w/v] bromophenol blue, 0.025% [w/v] SDS in H_2O), denatured by heating at 95 °C for 2 min and then immediately cooled on ice. Aliquots of the denatured samples (10 µL) were subjected to vertical gel electrophoresis. For mass spectrometry analysis see Figure S253 – Figure S256 (**31RNA_4C^{Me}**) and for quantification of reaction yield and purity of the final product see Figure S44 and Table S8.

2.12.12 Preparative (1 nmol scale) PEX reaction with a mixture of rATP, rUTP, rCTP, rG^{Pent}TP

Reaction was performed in duplicate in a total volume of 250 µL in ThermoPol buffer (1X) containing ssDNA template – **templ_31nt** (4.8 µM), 5´-(6-FAM)-labelled RNA primer – **FAM-RNA-prim_15nt** (4.0 µM), TGK polymerase (1.5 µM), mixture of rATP, rUTP, rCTP (0.8 mM) and the modified **rG^{Pent}TP** (0.4 mM). Reactions were carried out following the standard protocol in section 2.5.13. The purification was performed with QIAquick nucleotide removal kit according to standard supplier´s protocol. After puriifcation, 1 µL aliquot was removed, dilluted with H_2O (20X) and combined with 20 µL of 2X stop solution (95% [v/v] formamide, 0.5 mM EDTA, 0.025% [w/v] bromophenol blue, 0.025% [w/v] SDS in H_2O), denatured by heating at 95 °C for 2 min and then immediately cooled on ice. Aliquots of the denatured samples (10 µL) were subjected to vertical gel electrophoresis. For mass spectrometry analysis see Figure S257 – Figure S260 (**31RNA_4G^{Pent}**) and for quantification of reaction yield and purity of the final product see Figure S44 and Table S8.

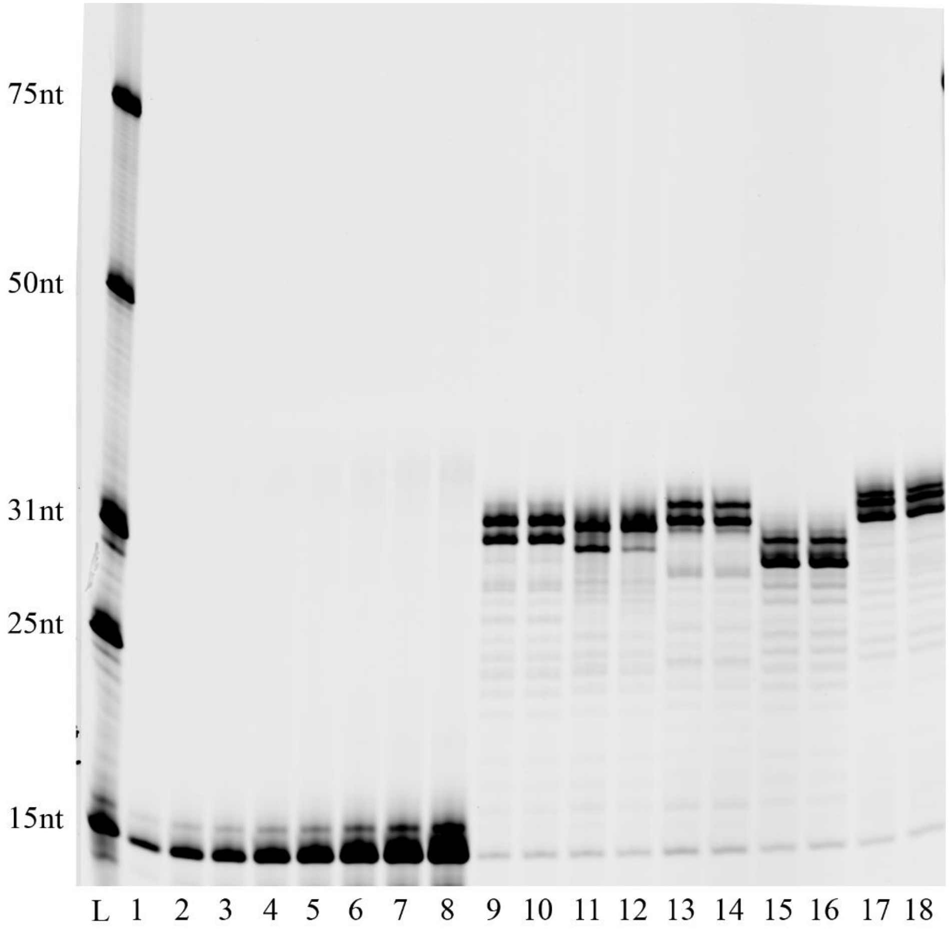

Figure S44. 12.5% dPAGE analysis. Preparative (1 nmol) scale PEX reaction with TGK polymerase and either all four natural rNTPs or a mixture of natural and modified **rN^{X}TP**s. (1-8) 5´-(6-FAM)-labelled RNA standards of increasing concentration (0.02 µM, 0.04 µM, 0.05 µM, 0.075 µM, 0.1 µM, 0.15 µM, 0.2 µM, 0.3 µM); (9) product of PEX reaction with natural rNTPs, 1st replicate; (10) product of PEX reaction with natural rNTPs, 2nd replicate; (11) product of PEX reaction with a mixture of **rA^{E}TP**, rUTP, rCTP, rGTP, 1st replicate; (12) product of PEX reaction with a mixture of **rA^{E}TP**, rUTP, rCTP, rGTP, 2nd replicate; (13) product of PEX reaction with a mixture of rATP, **rU^{Bio}TP**, rCTP, rGTP, 1st replicate; (14) product of PEX reaction with a mixture of rATP, **rU^{Bio}TP**, rCTP, rGTP, 2nd replicate; (15) product of PEX reaction with a mixture of rATP, rUTP, **rC^{Me}TP**, rGTP, 1st replicate; (16) product of PEX reaction with a mixture of rATP, rUTP, **rC^{Me}TP**, rGTP, 2nd replicate; (17) product of PEX reaction with a mixture of rATP, rUTP, rCTP, **rG^{Pent}TP**, 1st replicate; (18) product of PEX reaction with a mixture of rATP, rUTP, rCTP, **rG^{Pent}TP**, 2nd replicate; (L) RNA ladder composed of FAM-labelled RNA oligonucleotides of indicated length. FAM scan.

Table S8. Yield determination of large scale PEX reactions in 1 nmol scale. Yields were determined by comparison of product bands densities with standard curve constructed from serial dilution of known concentration of synthetic FAM-labelled oligonucleotide in ImageJ.

RNA type	Replicate No.	Yield [%]	Yield [pmol]
**31RNA_nat**	1	69	639
	2	74	738
**31RNA_4A^{E}**	1	72	718
	2	75	754
**31RNA_4U^{Bio}**	1	63	627
	2	64	636
**31RNA_4C^{Me}**	1	78	776
	2	74	736
**31RNA_4G^{Pent}**	1	74	744
	2	71	711

2.13 Semi-preparative scale PEX reaction with with 5´-(TINA)-templ_31nt or 5´-(dual-Bio)-templ_31nt and SFM4-3 polymerase (incorporation of 4 modifications)

2.13.1 Incorporation of rA^{E}TP, rA^{Pent}TP, rA^{Ph}TP

Reaction was performed in total volume of 50 µL in ThermoPol buffer (1X) containing labelled ssDNA template **5´-(TINA)-templ_31nt** (4.8 µM), 5´-(6-FAM)-labelled RNA primer – **FAM-RNA-prim_15nt** (4.0 µM), SFM4-3 polymerase (0.25 µM), mixture of rUTP, rCTP, rGTP (0.4 mM) and one of the modified **rA^{E}TP**, **rA^{Pent}TP** or **rA^{Ph}TP** (0.2 mM). Reactions were carried out following the standard protocol in section 2.5.13. For mass spectrometry analysis see Figure S197 (**31RNA_4A^{E}**), Figure S198 (**31RNA_4A^{Pent}**), Figure S199 (**31RNA_4A^{Ph}**).

2.13.2 Incorporation of rU^{E}TP, rU^{Pent}TP, rU^{Ph}TP

Reaction was performed in total volume of 10 µL in ThermoPol buffer (1X) containing labelled ssDNA template – **5´-(TINA)-templ_31nt** (4.8 µM), 5´-(6-FAM)-labelled RNA primer – **FAM-RNA-prim_15nt** (4.0 µM), SFM4-3 polymerase (0.75 µM), mixture of rATP, rCTP, rGTP (0.4 mM) and one of the modified **rU^{E}TP**, **rU^{Pent}TP** or **rU^{Ph}TP** (0.2 mM). Reactions were carried out following the standard protocol in section 2.5.13. For mass spectrometry analysis see Figure S200 (**31RNA_4U^{E}**), Figure S201 (**31RNA_4U^{Pent}**), Figure S202 (**31RNA_4U^{Ph}**).

2.13.3 Incorporation of rU^{Bio}TP, rU^{Dig}TP

Reaction was performed in total volume of 50 µL in ThermoPol buffer (1X) containing labelled ssDNA template – **5´-(TINA)-templ_31nt** (4.8 µM), 5´-(6-FAM)-labelled RNA primer – **FAM-RNA-prim_15nt** (4.0 µM), SFM4-3 polymerase (1.0 µM), mixture of rATP, rCTP, rGTP (0.2 mM) and one of the modified **rU^{Bio}TP** or **rU^{Dig}TP** (0.2 mM). Reactions were carried out

following the standard protocol in section 2.5.13. For mass spectrometry analysis see Figure S203 (**31RNA_4U^{Bio}**), Figure S204 (**31RNA_4U^{Dig}**).

2.13.4 Incorporation of rC^{E}TP, rC^{Pent}TP, rC^{Ph}TP

Reaction was performed in total volume of 50 µL in ThermoPol buffer (1X) containing labelled ssDNA template – **5´-(TINA)-templ_31nt** (4.8 µM), 5´-(6-FAM)-labelled RNA primer – **FAM-RNA-prim_15nt** (4.0 µM), SFM4-3 polymerase (0.5 µM), mixture of rATP, rUTP, rGTP (0.4 mM) and one of the modified **rC^{E}TP**, **rC^{Pent}TP** or **rC^{Ph}TP** (0.2 mM). Reactions were carried out following the standard protocol in section 2.5.13. For mass spectrometry analysis see Figure S205 (**31RNA_4C^{E}**), Figure S206 (**31RNA_4C^{Pent}**), Figure S207 (**31RNA_4C^{Ph}**).

2.13.5 Incorporation of rC^{mBdp}TP

Reaction was performed in total volume of 100 µL in ThermoPol buffer (1X) containing labelled ssDNA template – **5´-(dual-Bio)-templ_31nt** (2.4 µM), RNA primer – **RNA-prim_15nt** (2.0 µM), SFM4-3 polymerase (2.0 µM), mixture of rATP, rUTP, rGTP (0.4 mM) and the modified **rC^{mBdp}TP** (0.8 mM). Reaction was carried out following the standard protocol in section 2.5.14. For mass spectrometry analysis see Figure S208 (**31RNA_4C^{mBdp}**).

2.13.6 Incorporation of rG^{E}TP, rG^{Pent}TP, rG^{Ph}TP

Reaction was performed in total volume of 50 µL in ThermoPol buffer (1X) containing labelled ssDNA template – **5´-(TINA)-templ_31nt** (4.8 µM), 5´-(6-FAM)-labelled RNA primer – **FAM-RNA-prim_15nt** (4.0 µM), SFM4-3 polymerase (2.0 µM), mixture of rATP, rUTP, rCTP (1.0 mM) and one of the modified **rG^{E}TP**, **rG^{Pent}TP** or **rG^{Ph}TP** (0.4 mM). Reactions were carried out following the standard protocol in section 2.5.13. For mass spectrometry analysis see Figure S215 and Figure S216 (**31RNA_4G^{E}**), Figure S209 (**31RNA_4G^{Pent}**), Figure S210 (**31RNA_4G^{Ph}**).

2.14 Analytical scale PEX reaction with combination of four different base-modified rN^{X}TPs and variously long templates

2.14.1 PEX with rA^{E}TP, rU^{Bio}TP, rC^{Ph}TP, rG^{Pent}TP and templ_19nt_mix using TGK polymerase

Reaction was performed in total volume of 10 µL in ThermoPol buffer (1X) containing ssDNA template – **templ_19nt_mix** (4.8 µM), 5´-(6-FAM)-labelled RNA primer – **FAM-RNA-prim_15nt** (4.0 µM), TGK polymerase (1.0 µM), mixture of **rA^{E}TP**, **rU^{Bio}TP**, **rC^{Ph}TP**, **rG^{Pent}TP** (0.1 mM). Positive control was performed under same conditions with a mixture of natural rNTPs (0.1 mM) instead of modified **rN^{X}TP**s. Five different negative control reactions were performed under same conditions using in each reaction a different mixture of three natural rNTPs [0.1 mM; r(AUC), r(AUG), r(ACG), r(UCG)] and H_2O or with complete replacement of all rNTPs by H_2O.

Reactions were carried out following the standard protocol in section 2.5.8. For dPAGE analysis of **19RNA_AUCG** or **19RNA_A^EU^{Bio}C^{Ph}G^{Pent}** see Figure S45 and Figure S49.

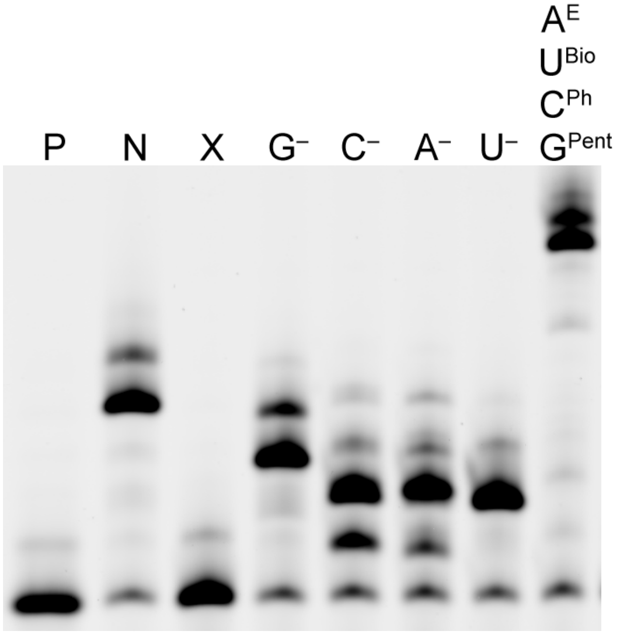

Figure S45. 12.5% dPAGE analysis of PEX reaction. (P) RNA primer; (N) positive control, all natural rNTPs; (X) negative control, no rNTPs; (G^-) negative control, mixture of rATP, rUTP, rCTP and H_2O; (C^-) negative control, mixture of rATP, rUTP, rGTP and H_2O; (A^-) negative control, mixture of rUTP, rCTP, rGTP and H_2O; (U^-) negative control, mixture of rATP, rCTP, rGTP and H_2O; (A^E, U^{Bio}, C^{Ph}, G^{Pent}) modification, mixture of **rA^ETP**, **rU^{Bio}TP**, **rC^{Ph}TP**, **rG^{Ph}TP**. FAM scan.

2.14.2 PEX with rA^ETP, rU^{Bio}TP, rC^{Ph}TP, rG^{Pent}TP and 5´-(TINA)-templ_31nt using TGK polymerase

Reaction was performed in total volume of 10 µL in ThermoPol buffer (1X) containing labelled ssDNA template – **5´-(TINA)-templ_31nt** (4.8 µM), 5´-(6-FAM)-labelled RNA primer – **FAM-RNA-prim_15nt** (4.0 µM), TGK polymerase (2.0 µM), mixture of **rA^ETP**, **rU^{Bio}TP**, **rC^{Ph}TP**, **rG^{Pent}TP** (0.2 mM). Positive control was performed under same conditions with a mixture of natural rNTPs (0.2 mM) instead of modified **rN^XTP**s. Five different negative control reactions were performed under same conditions using in each reaction a different mixture of three natural rNTPs [0.2 mM; r(AUC), r(AUG), r(ACG), r(UCG)] and H_2O or with complete replacement of all rNTPs by H_2O. Reactions were carried out following the standard protocol in section 2.5.8. For dPAGE analysis of **31RNA_AUCG** or **31RNA_A^{E}U^{Bio}C^{Ph}G^{Pent}** see Figure S46 and Figure S49.

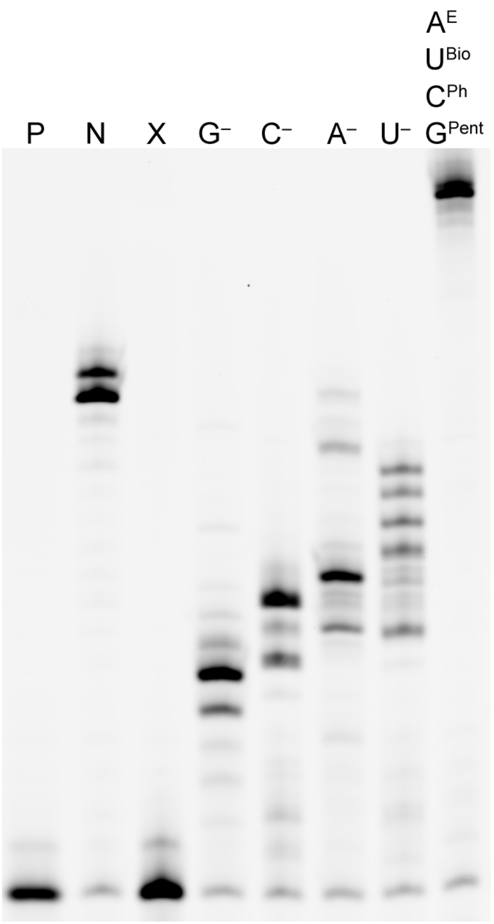

Figure S46. 12.5% dPAGE analysis of PEX reaction. (P) RNA primer; (N) positive control, all natural rNTPs; (X) negative control, no rNTPs; (G$^{–}$) negative control, mixture of rATP, rUTP, rCTP and H_2O; (C$^{–}$) negative control, mixture of rATP, rUTP, rGTP and H_2O; (A$^{–}$) negative control, mixture of rUTP, rCTP, rGTP and H_2O; (U$^{–}$) negative control, mixture of rATP, rCTP, rGTP and H_2O; (A^{E}, U^{Bio}, C^{Ph}, G^{Pent}) modification, mixture of **rA^{E}TP**, **rU^{Bio}TP**, **rC^{Ph}TP**, **rG^{Ph}TP**. FAM scan.

2.14.3 PEX with rA^{E}TP, rU^{Bio}TP, rC^{Ph}TP, rG^{Pent}TP and templ_65nt using TGK polymerase

Reaction was performed in total volume of 10 µL in ThermoPol buffer (1X) containing ssDNA template – **templ_65nt** (4.8 µM), 5´-(6-FAM)-labelled RNA primer – **FAM-RNA-prim_15nt** (4.0 µM), TGK polymerase (2.5 µM), mixture of **rA^{E}TP**, **rU^{Bio}TP**, **rC^{Ph}TP**, **rG^{Pent}TP** (0.4 mM). Positive control was performed under same conditions with a mixture of natural rNTPs (0.4 mM) instead of modified **rN^{X}TP**s. Five different negative control reactions were performed under same conditions using in each reaction a different mixture of three natural rNTPs [0.4 mM; r(AUC), r(AUG), r(ACG), r(UCG)] and H_2O or with complete replacement of all rNTPs by H_2O. Reactions were carried out following the standard protocol in section 2.5.8. For dPAGE analysis of **65RNA_AUCG** or **65RNA_A^{E}U^{Bio}C^{Ph}G^{Pent}** see Figure S47 and Figure S49.

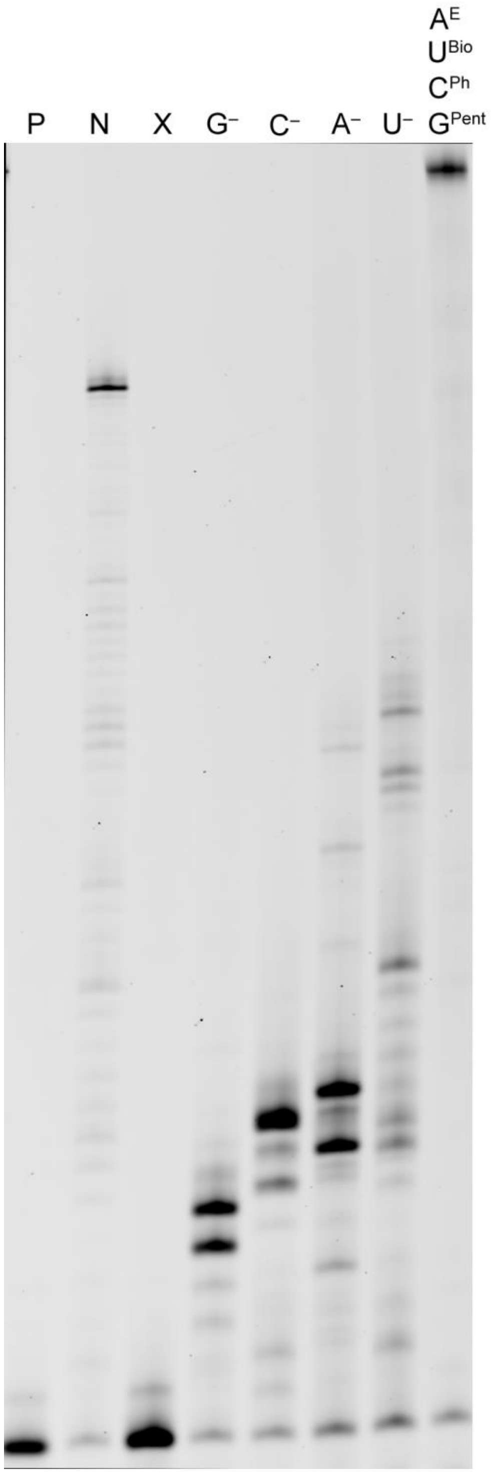

Figure S47. 12.5% dPAGE analysis of PEX reaction. (P) RNA primer; (N) positive control, all natural rNTPs; (X) negative control, no rNTPs; (G^-) negative control, mixture of rATP, rUTP, rCTP and H_2O; (C^-) negative control, mixture of rATP, rUTP, rGTP and H_2O; (A^-) negative control, mixture of rUTP, rCTP, rGTP and H_2O; (U^-) negative control, mixture of rATP, rCTP, rGTP and H_2O; (A^E, U^{Bio}, C^{Ph}, G^{Pent}) modification, mixture of **rA^ETP**, **rU^{Bio}TP**, **rC^{Ph}TP**, **rG^{Ph}TP**. FAM scan.

2.14.4 PEX with rA^{E}TP, rU^{Bio}TP, rC^{Ph}TP, rG^{Pent}TP and templ_98nt using TGK polymerase

Reaction was performed in total volume of 10 µL in ThermoPol buffer (1X) containing ssDNA template – **templ_98nt** (4.8 µM), 5´-(6-FAM)-labelled RNA primer – **FAM-RNA-prim_15nt** (4.0 µM), TGK polymerase (5.0 µM), mixture of **rA^{E}TP**, **rU^{Bio}TP**, **rC^{Ph}TP**, **rG^{Pent}TP** (0.4 mM). Positive control was performed under same conditions with a mixture of natural rNTPs (0.4 mM) instead of modified **rN^{X}TP**s. Five different negative control reactions were performed under same conditions using in each reaction a different mixture of three natural rNTPs [0.4 mM; r(AUC), r(AUG), r(ACG), r(UCG)] and H_2O or with complete replacement of all rNTPs by H_2O. Reactions were carried out following the standard protocol in section 2.5.8. For dPAGE analysis of **98RNA_AUCG** or **98RNA_A^{E}U^{Bio}C^{Ph}G^{Pent}** see Figure S48 and Figure S49.

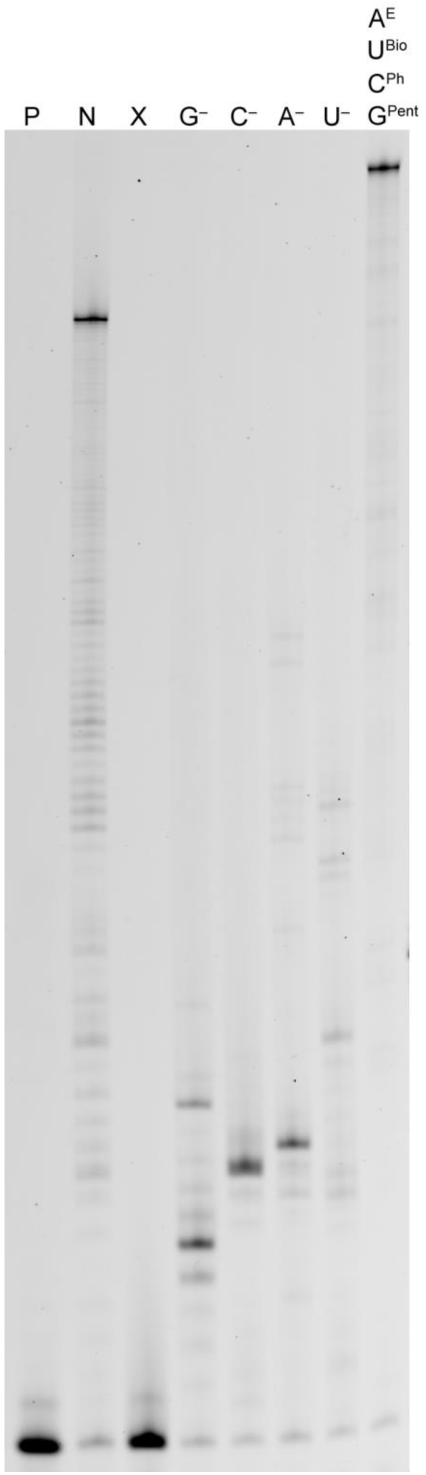

Figure S48. 12.5% dPAGE analysis of PEX reaction. (P) RNA primer; (N) positive control, all natural rNTPs; (X) negative control, no rNTPs; (G⁻) negative control, mixture of rATP, rUTP, rCTP and H_2O; (C⁻) negative control, mixture of rATP, rUTP, rGTP and H_2O; (A⁻) negative control, mixture of rUTP, rCTP, rGTP and H_2O; (U⁻) negative control, mixture of rATP, rCTP, rGTP and H_2O; (A^{E}, U^{Bio}, C^{Ph}, G^{Pent}) modification, mixture of **rA^{E}TP**, **rU^{Bio}TP**, **rC^{Ph}TP**, **rG^{Ph}TP**. FAM-scan.

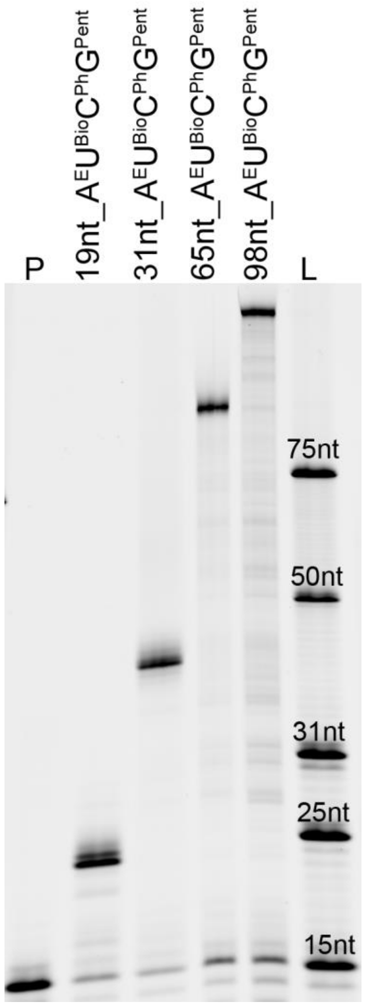

Figure S49. 12.5% dPAGE analysis of PEX reaction. (P) RNA primer; (19nt_A^{E}U^{Bio}C^{Ph}G^{Pent}) modified RNA product **19RNA_A^{E}U^{Bio}C^{Ph}G^{Pent}** of PEX reaction prepared in section 2.14.1; (31nt_A^{E}U^{Bio}C^{Ph}G^{Pent}) modified RNA product **31RNA_A^{E}U^{Bio}C^{Ph}G^{Pent}** of PEX reaction prepared in section 2.14.2; (65nt_A^{E}U^{Bio}C^{Ph}G^{Pent}) modified RNA product **65RNA_A^{E}U^{Bio}C^{Ph}G^{Pent}** of PEX reaction prepared in section 2.14.3; (98nt_A^{E}U^{Bio}C^{Ph}G^{Pent}) modified RNA product **98RNA_A^{E}U^{Bio}C^{Ph}G^{Pent}** of PEX reaction prepared in section 2.14.4; (L) RNA ladder composed of FAM-labelled RNA oligonucleotides of indicated length. FAM scan.

2.14.5 PEX with rA^{E}TP, rU^{Bio}TP, rC^{Ph}TP, rG^{Pent}TP and templ_19nt_mix using SFM4-3 polymerase

Reaction was performed in total volume of 10 µL in ThermoPol buffer (1X) containing ssDNA template – **templ_19nt_mix** (4.8 µM), 5´-(6-FAM)-labelled RNA primer – **FAM-RNA-prim_15nt** (4.0 µM), SFM4-3 polymerase (1.0 µM), mixture of **rA^{E}TP**, **rU^{Bio}TP**, **rC^{Ph}TP**, **rG^{Pent}TP** (0.1 mM). Positive control was performed under same conditions with a mixture of natural rNTPs (0.1 mM) instead of modified **rN^{X}TP**s. Five different negative control reactions were performed under same conditions using in each reaction a different mixture of three natural

rNTPs [0.1 mM; r(AUC), r(AUG), r(ACG), r(UCG)] and H_2O or with complete replacement of all rNTPs by H_2O. Reactions were carried out following the standard protocol in section 2.5.8. For dPAGE analysis of **19RNA_AUCG** or **19RNA_A^{E}U^{Bio}C^{Ph}G^{Pent}** see Figure S50.

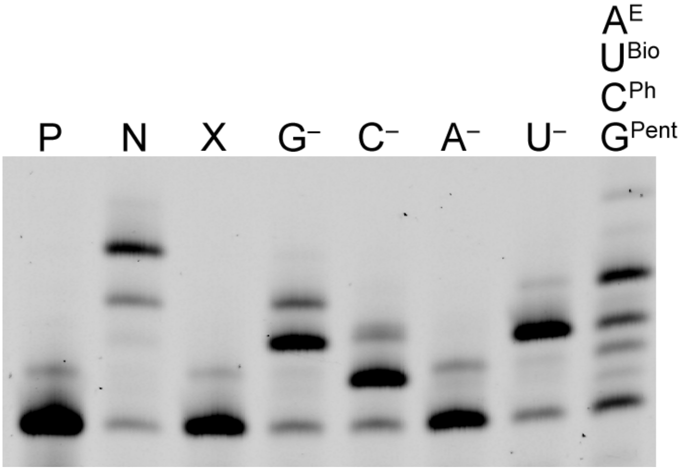

Figure S50. 12.5% dPAGE analysis of PEX reaction. (P) RNA primer; (N) positive control, all natural rNTPs; (X) negative control, no rNTPs; (G^-) negative control, mixture of rATP, rUTP, rCTP and H_2O; (C^-) negative control, mixture of rATP, rUTP, rGTP and H_2O; (A^-) negative control, mixture of rUTP, rCTP, rGTP and H_2O; (U^-) negative control, mixture of rATP, rCTP, rGTP and H_2O; (A^E, U^{Bio}, C^{Ph}, G^{Pent}) modification, mixture of **rA^{E}TP**, **rU^{Bio}TP**, **rC^{Ph}TP**, **rG^{Ph}TP**. FAM scan.

2.14.6 PEX with rA^{E}TP, rU^{Bio}TP, rC^{Ph}TP, rG^{Pent}TP and 5´-(TINA)-templ_31nt using SFM4-3 polymerase

Reaction was performed in total volume of 10 µL in ThermoPol buffer (1X) containing labelled ssDNA template – **5´-(TINA)-templ_31nt** (4.8 µM), 5´-(6-FAM)-labelled RNA primer – **FAM-RNA-prim_15nt** (4.0 µM), SFM4-3 polymerase (2.0 µM), mixture of **rA^{E}TP**, **rU^{Bio}TP**, **rC^{Ph}TP**, **rG^{Pent}TP** (0.2 mM). Positive control was performed under same conditions with a mixture of natural rNTPs (0.2 mM) instead of modified **rN^{X}TP**s. Five different negative control reactions were performed under same conditions using in each reaction a different mixture of three natural rNTPs [0.2 mM; r(AUC), r(AUG), r(ACG), r(UCG)] and H_2O or with complete replacement of all rNTPs by H_2O. Reactions were carried out following the standard protocol in section 2.5.8. For dPAGE analysis of **31RNA_AUCG** or **31RNA_A^{E}U^{Bio}C^{Ph}G^{Pent}** see Figure S51.

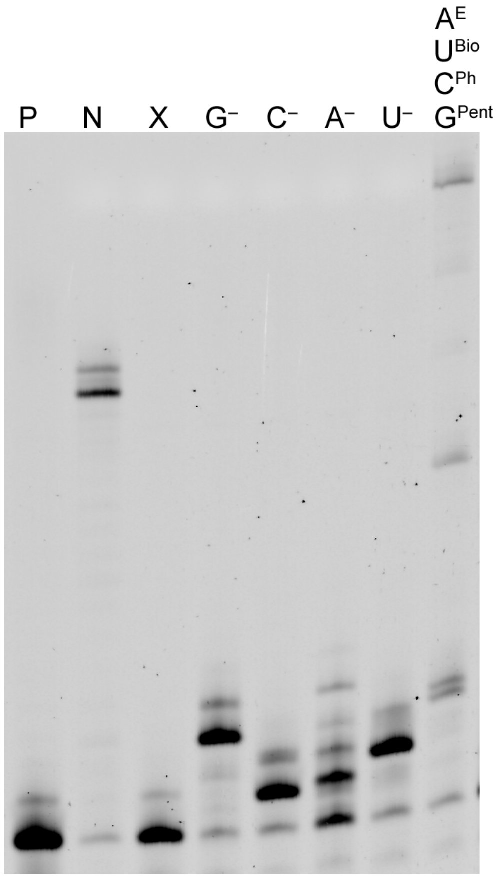

Figure S51. 12.5% dPAGE analysis of PEX reaction. (P) RNA primer; (N) positive control, all natural rNTPs; (X) negative control, no rNTPs; (G⁻) negative control, mixture of rATP, rUTP, rCTP and H_2O; (C⁻) negative control, mixture of rATP, rUTP, rGTP and H_2O; (A⁻) negative control, mixture of rUTP, rCTP, rGTP and H_2O; (U⁻) negative control, mixture of rATP, rCTP, rGTP and H_2O; (A^{E}, U^{Bio}, C^{Ph}, G^{Pent}) modification, mixture of **rA^{E}TP**, **rU^{Bio}TP**, **rC^{Ph}TP**, **rG^{Ph}TP**. FAM scan.

2.14.7 PEX with rA^{E}TP, rU^{Bio}TP, rC^{Ph}TP, rG^{Pent}TP and templ_65nt using SFM4-3 polymerase

Reaction was performed in total volume of 10 µL in ThermoPol buffer (1X) containing ssDNA template – **templ_65nt** (4.8 µM), 5´-(6-FAM)-labelled RNA primer – **FAM-RNA-prim_15nt** (4.0 µM), SFM4-3 polymerase (2.5 µM), mixture of **rA^{E}TP**, **rU^{Bio}TP**, **rC^{Ph}TP**, **rG^{Pent}TP** (0.4 mM). Positive control was performed under same conditions with a mixture of natural rNTPs (0.4 mM) instead of modified **rN^{X}TP**s. Five different negative control reactions were performed under same conditions using in each reaction a different mixture of three natural rNTPs [0.4 mM; r(AUC), r(AUG), r(ACG), r(UCG)] and H_2O or with complete replacement of all rNTPs by H_2O. Reactions were carried out following the standard protocol in section 2.5.8. For dPAGE analysis of **65RNA_AUCG** or **65RNA_$A^{E}U^{Bio}C^{Ph}G^{Pent}$** see Figure S52.

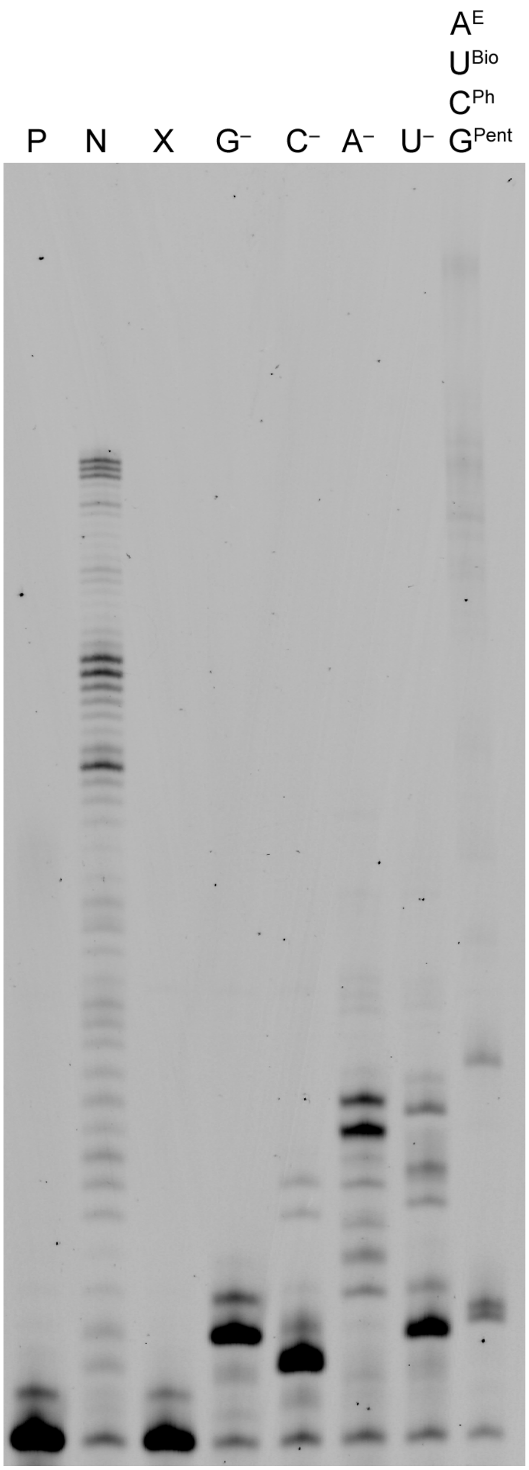

Figure S52. 12.5% dPAGE analysis of PEX reaction. (P) RNA primer; (N) positive control, all natural rNTPs; (X) negative control, no rNTPs; ($G^{–}$) negative control, mixture of rATP, rUTP, rCTP and H_2O; ($C^{–}$) negative control, mixture of rATP, rUTP, rGTP and H_2O; ($A^{–}$) negative control, mixture of rUTP, rCTP, rGTP and H_2O; ($U^{–}$) negative control, mixture of rATP, rCTP, rGTP and H_2O; (A^{E}, U^{Bio}, C^{Ph}, G^{Pent}) modification, mixture of **rA^{E}TP**, **rU^{Bio}TP**, **rC^{Ph}TP**, **rG^{Ph}TP**. FAM scan.

2.14.8 PEX with $rA^{E}TP$, $rU^{Bio}TP$, $rC^{Ph}TP$, $rG^{Pent}TP$ and templ_98nt using SFM4-3 polymerase

Reaction was performed in total volume of 10 µL in ThermoPol buffer (1X) containing ssDNA template – **templ_98nt** (4.8 µM), 5´-(6-FAM)-labelled RNA primer – **FAM-RNA-prim_15nt** (4.0 µM), SFM4-3 polymerase (5.0 µM), mixture of **$rA^{E}TP$**, **$rU^{Bio}TP$**, **$rC^{Ph}TP$**, **$rG^{Pent}TP$** (0.4 mM). Positive control was performed under same conditions with a mixture of natural rNTPs (0.4 mM) instead of modified **$rN^{X}TP$**s. Five different negative control reactions were performed under same conditions using in each reaction a different mixture of three natural rNTPs [0.4 mM; r(AUC), r(AUG), r(ACG), r(UCG)] and H_2O or with complete replacement of all rNTPs by H_2O. Reactions were carried out following the standard protocol in section 2.5.8. For dPAGE analysis of **98RNA_AUCG** or **$98RNA\_A^{E}U^{Bio}C^{Ph}G^{Pent}$** see Figure S53.

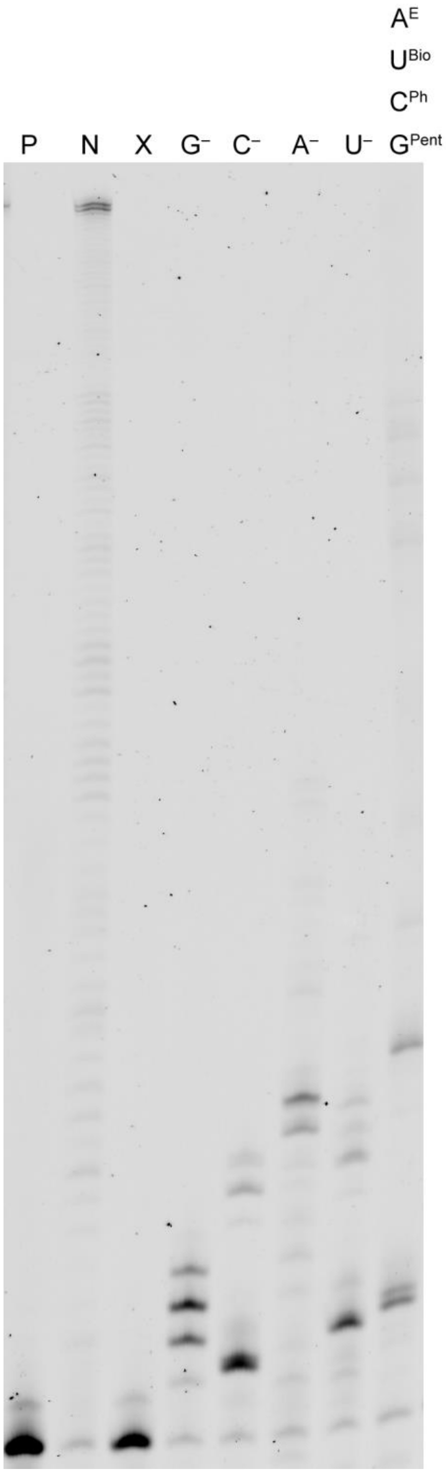

Figure S53. 12.5% dPAGE analysis of PEX reaction. (P) RNA primer; (N) positive control, all natural rNTPs; (X) negative control, no rNTPs; ($G^{–}$) negative control, mixture of rATP, rUTP, rCTP and H_2O; ($C^{–}$) negative control, mixture of rATP, rUTP, rGTP and H_2O; ($A^{–}$) negative control, mixture of rUTP, rCTP, rGTP and H_2O; ($U^{–}$) negative control, mixture of rATP, rCTP, rGTP and H_2O; (A^{E}, U^{Bio}, C^{Ph}, G^{Pent}) modification, mixture of **rA^{E}TP**, **rU^{Bio}TP**, **rC^{Ph}TP**, **rG^{Ph}TP**. FAM scan.

2.15 Semi-preparative scale PEX reaction with combination of four different base-modified $rN^{X}TPs$ and variously long templates

2.15.1 PEX with $rA^{E}TP$, $rU^{Bio}TP$, $rC^{Ph}TP$, $rG^{Pent}TP$ and templ_19nt_mix using TGK polymerase

Reaction was performed in total volume of 50 µL in ThermoPol buffer (1X) containing ssDNA template – **templ_19nt_mix** (4.8 µM), RNA primer – **RNA-prim_15nt** (4.0 µM), TGK polymerase (1.0 µM), mixture of **$rA^{E}TP$**, **$rU^{Bio}TP$**, **$rC^{Ph}TP$**, **$rG^{Pent}TP$** (0.1 mM). Reaction was carried out following the standard protocol in section 2.5.15. For mass spectrometry analysis see Figure S217 and Figure S218 (**$19RNA\_A^{E}U^{Bio}C^{Ph}G^{Pent}$**).

2.15.2 PEX with $rA^{E}TP$, $rU^{Bio}TP$, $rC^{Ph}TP$, $rG^{Pent}TP$ and 5´-(TINA)-templ_31nt using TGK polymerase

Reaction was performed in total volume of 50 µL in ThermoPol buffer (1X) containing labelled ssDNA template – **5´-(TINA)-templ_31nt** (4.8 µM), RNA primer – **RNA-prim_15nt** (4.0 µM), TGK polymerase (2.0 µM), mixture of **$rA^{E}TP$**, **$rU^{Bio}TP$**, **$rC^{Ph}TP$**, **$rG^{Pent}TP$** (0.2 mM). Reaction was carried out following the standard protocol in section 2.5.15. For mass spectrometry analysis see Figure S219 and Figure S220 (**$31RNA\_A^{E}U^{Bio}C^{Ph}G^{Pent}$**).

2.15.3 PEX with $rA^{E}TP$, $rU^{Bio}TP$, $rC^{Ph}TP$, $rG^{Pent}TP$ and templ_65nt using TGK polymerase

Reaction was performed in total volume of 50 µL in ThermoPol buffer (1X) containing ssDNA template – **templ_65nt** (4.8 µM), RNA primer – **RNA-prim_15nt** (4.0 µM), TGK polymerase (2.5 µM), mixture of **$rA^{E}TP$**, **$rU^{Bio}TP$**, **$rC^{Ph}TP$**, **$rG^{Pent}TP$** (0.4 mM). Reaction was carried out following the standard protocol in section 2.5.15. For mass spectrometry analysis see Figure S221 and Figure S222 (**$65RNA\_A^{E}U^{Bio}C^{Ph}G^{Pent}$**).

2.15.4 PEX with $rA^{E}TP$, $rU^{Bio}TP$, $rC^{Ph}TP$, $rG^{Pent}TP$ and templ_98nt using TGK polymerase

Reaction was performed in total volume of 50 µL in ThermoPol buffer (1X) containing ssDNA template – **templ_98nt** (4.8 µM), RNA primer – **RNA-prim_15nt** (4.0 µM), TGK polymerase (5.0 µM), mixture of **$rA^{E}TP$**, **$rU^{Bio}TP$**, **$rC^{Ph}TP$**, **$rG^{Pent}TP$** (0.4 mM). Reaction was carried out following the standard protocol in section 2.5.15. For mass spectrometry analysis see Figure S223 and Figure S224 (**$98RNA\_A^{E}U^{Bio}C^{Ph}G^{Pent}$**).

2.15.5 PEX with $rA^{E}TP$, $rU^{Bio}TP$, $rC^{Ph}TP$, $rG^{Pent}TP$ and templ_98nt for RT analysis

Reactions were performed in total volume of 50 µL in ThermoPol buffer (1X) containing ssDNA template – **templ_98nt** (4.8 µM), 5´-(6-FAM)-labelled RNA primer – **FAM-RNA-prim_15nt** (4.0 µM), TGK polymerase (5.0 µM) and a mixture of either natural rNTPs (0.4 mM) or modified **$rA^{E}TP$**, **$rU^{Bio}TP$**, **$rC^{Ph}TP$**, **$rG^{Pent}TP$** (0.4 mM). Reactions were carried out following the standard protocol in section 2.5.15.

2.15.6 RT analysis of natural or base-modified RNA

Reaction was performed in total volume of 10 µL in SSIV reaction buffer (1X). Briefly, 5´-(FAM)-labelled base-modified RNA template (0.4 µM; **98RNA_A^{E}U^{Bio}C^{Ph}G^{Pent}**) prepared according to protocol in section 2.15.5 and 5´-(Cy5)-labelled DNA primer – **Cy5-DNA-prim_20nt** (0.2 µM) in presence of SSIV reaction buffer were heated up to 95 °C for 30 sec and then cooled down to 3 °C (0.1 °C s^{-1}). After addition of SSIV RT (200 U), DTT (5 mM) and dNTPs (0.5 mM), the mixture was incubated at 55 °C for 5 h in a thermal cycler with heated lid (95 °C). Positive control was performed under same conditions with 5´-(FAM)-labelled natural RNA template (**98RNA_AUCG**) prepared according to section 2.15.5 and negative control was performed in absence of RNA template. After reaction, the samples were purified using QIAquick nucleotide removal kit according to standard supplier´s protocol, eluted in 10 µL of H_2O and prepared for analysis according to protocol in section 2.5.1. For dPAGE analysis see Figure S54.

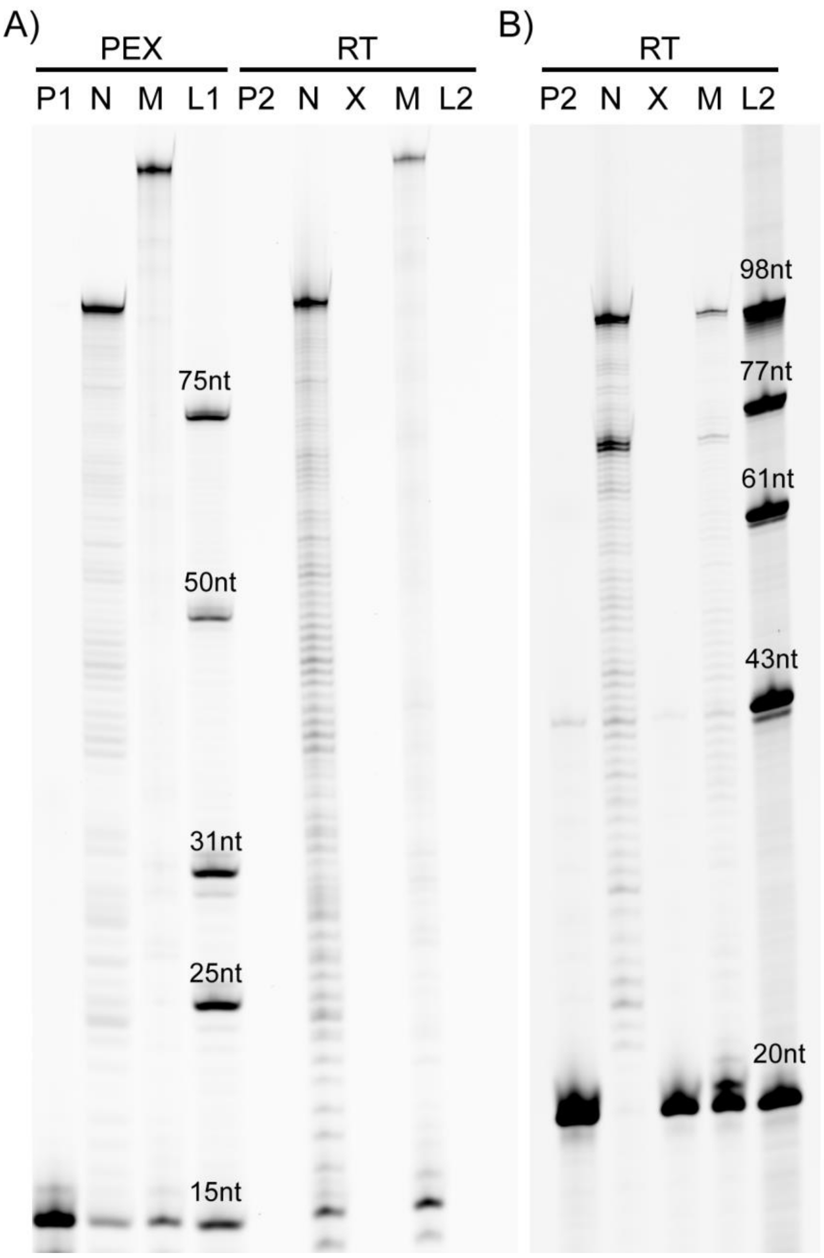

Figure S54. 12.5% dPAGE analysis. (P1) RNA primer; (N) natural RNA; (M) modified RNA; (L1) RNA ladder composed of FAM-labelled RNA oligonucleotides of indicated length; (P2) DNA primer; (N) cDNA from natural RNA; (X) negative control, no RNA; (M) cDNA from modified RNA; (L2) DNA ladder composed of Cy5-labelled DNA oligonucleotides of indicated length. A) FAM scan (visualisation of RNA templates); B) Cy5 scan, (visualisation of cDNA product).

2.16 Comparison of TGK polymerase, SFM4-3 polymerase and T7 RNAP in enzymatic synthesis of RNA

2.16.1 Analysis of PEX reaction with RNA primer and various amounts of TGK or SFM4-3 polymerase

Reactions were performed in total volume of 10 µL in ThermoPol buffer (1X) containing ssDNA template – **templ_16nt** (0.24 µM), 5´-(Cy5)-labelled RNA primer – **Cy5-RNA-prim_15nt** (0.2 µM), the modified **rC^{mBdp}TP** (0.1 mM) and either TGK or SFM4-3 polymerase at different concentrations (0.01 µM, 0.03 µM, 0.05 µM, 0.07 µM, 0.1 µM, 0.2 µM). Reaction mixtures were heated up to 95 °C for 30 sec, followed by incubation at 60 °C for 1 h in a thermal cycler with heated lid (100 °C). For sample preparation for dPAGE analysis see protocol in section 2.5.1. For dPAGE analysis of **16RNA_C^{mBdp}** see Figure S55 and for conversion analysis of RNA primer to product see Figure S56.

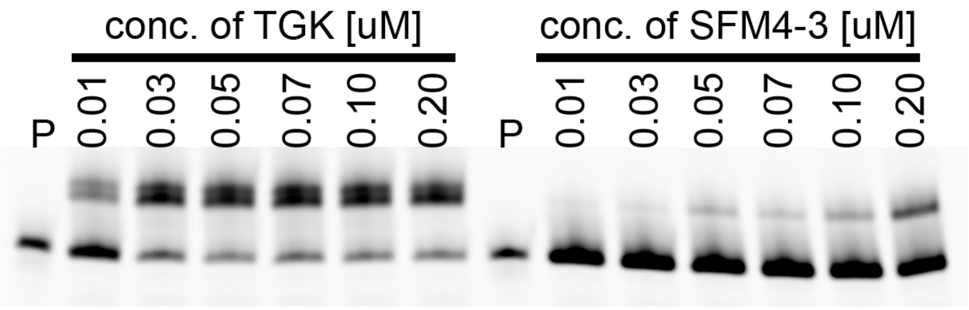

Figure S55. 22.5% dPAGE analysis. SNI of **rC^{mBdp}TP** with Cy5-labelled RNA primer and increasing concentration (0.01, 0.03, 0.05, 0.07, 0.1, 0.2 µM) of either TGK or SFM4-3 polymerase. (P) RNA primer. Cy5 scan.

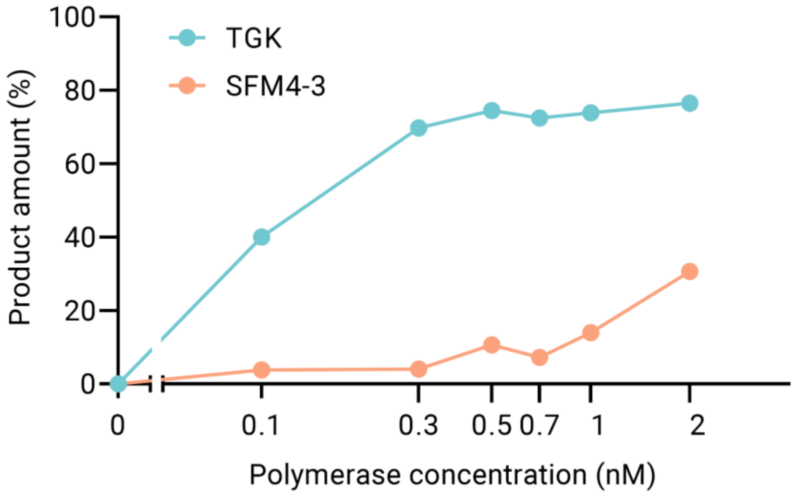

Figure S56. Efficiency of SNI with **rC^{mBdp}TP** and various concentrations of either TGK or SFM4-3 polymerase represented by percentage of primer conversion to product.

2.16.2 Kinetic studies of PEX reaction with RNA primer and TGK or SFM4-3 polymerase
Reactions were performed in total volume of 10 µL in ThermoPol buffer (1X) containing ssDNA template – **templ_16nt** (0.24 µM), 5´-(Cy5)-labelled RNA primer – **Cy5-RNA-prim_15nt** (0.2 µM), the modified **rC^{mBdp}TP** (0.1 mM) and either TGK (0.1 µM) or SFM4-3 (0.1 µM) polymerase. Reaction mixtures were heated up to 95 °C for 30 sec, followed by incubation at 60 °C for different time periods (1 min, 5 min, 10 min, 15 min, 30 min, 1 h) in a thermal cycler with heated lid (100 °C). For sample preparation for dPAGE analysis see protocol in section 2.5.1. For dPAGE analysis of **16RNA_C^{mBdp}** and quantification see Figure S57.

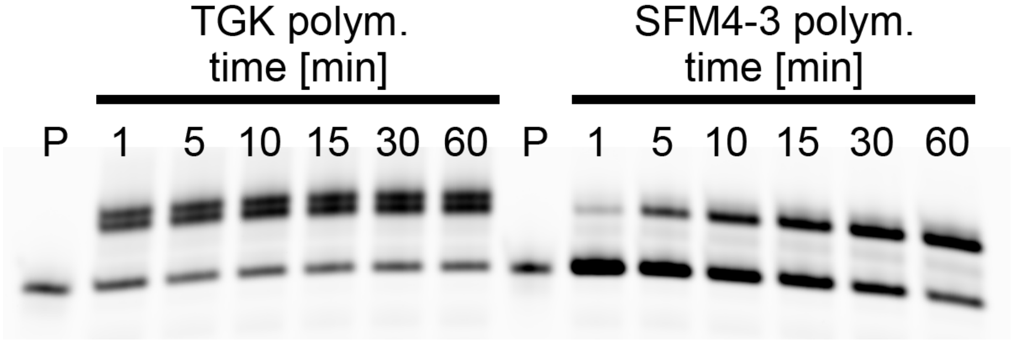

Figure S57. 22.5% dPAGE analysis. Kinetics of SNI reaction with **rC^{mBdp}TP** and RNA primer using TGK or SFM4-3 polymerase for different time periods (1, 5, 10, 15, 30, 60 min). (P) RNA primer. Cy5 scan.

2.16.3 Kinetic studies of PEX reaction with DNA primer and TGK or SFM4-3 polymerase
Reactions were performed in total volume of 10 µL in ThermoPol buffer (1X) containing ssDNA template – **templ_16nt** (0.24 µM), 5´-(Cy5)-labelled DNA primer – **Cy5-DNA-prim_15nt** (0.2 µM), the modified **rC^{mBdp}TP** (0.1 mM) and either TGK (0.1 µM) or SFM4-3 (0.1 µM) polymerase. Reaction mixtures were heated up to 95 °C for 30 sec, followed by incubation at 60 °C for different time periods (1 min, 5 min, 10 min, 15 min, 30 min, 1 h) in a thermal cycler with heated lid (100 °C). For sample preparation for dPAGE analysis see protocol in section 2.5.1. For dPAGE analysis of **16DNA-RNA_C^{mBdp}** and quantification see Figure S58.

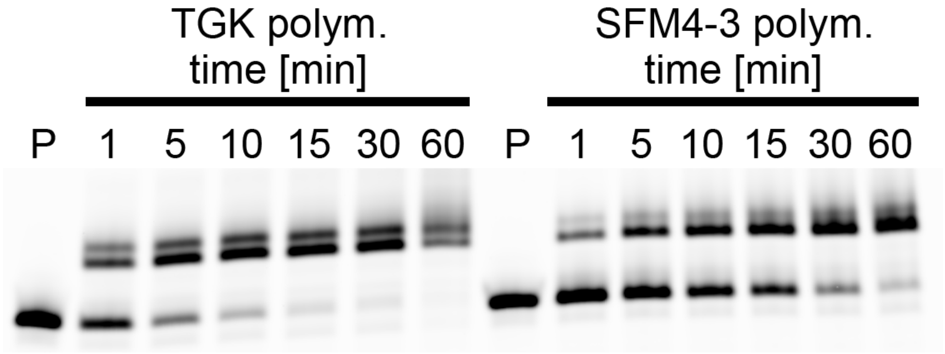

Figure S58. 22.5% dPAGE analysis. Kinetics of SNI reaction with **rC^{mBdp}TP** and DNA primer using TGK or SFM4-3 polymerase for different time periods (1, 5, 10, 15, 30, 60 min). (P) DNA primer. Cy5 scan.

2.16.4 PEX with templ_poly-U and TGK or SFM4-3 polymerase

Reaction was performed in total volume of 10 µL in ThermoPol buffer (1X). ssDNA template – **templ_poly-U** (0.24 µM) and 5´-(6-FAM)-labelled RNA primer – **FAM-RNA-prim_15nt** (0.2 µM) in presence of ThermoPol buffer were heated up to 95 °C for 30 sec and then cooled down to 3 °C (0.1 °C s^{-1}). After addition of a mixture of rATP, rCTP, rGTP (0.1 mM), the modified **rU^{Bio}TP** (0.1 mM) and either TGK (0.1 µM) or SFM4-3 (0.1 µM) polymerase, the reaction mixtures were incubated at 60 °C for 1.5 h in a thermal cycler with heated lid (100 °C). Positive control was performed under same conditions with natural rUTP (0.1 mM) instead of the modified **rU^{Bio}TP**. For negative control reaction H_2O was used instead of rUTP. Reaction mixtures were combined with TurboDNase (2 U) and incubated at 37 °C for 15 min. For sample preparation for dPAGE analysis of **45RNA_poly-U** or **45RNA_poly-U^{Bio}** see protocol in section 2.5.1. For dPAGE analysis see Figure S59.

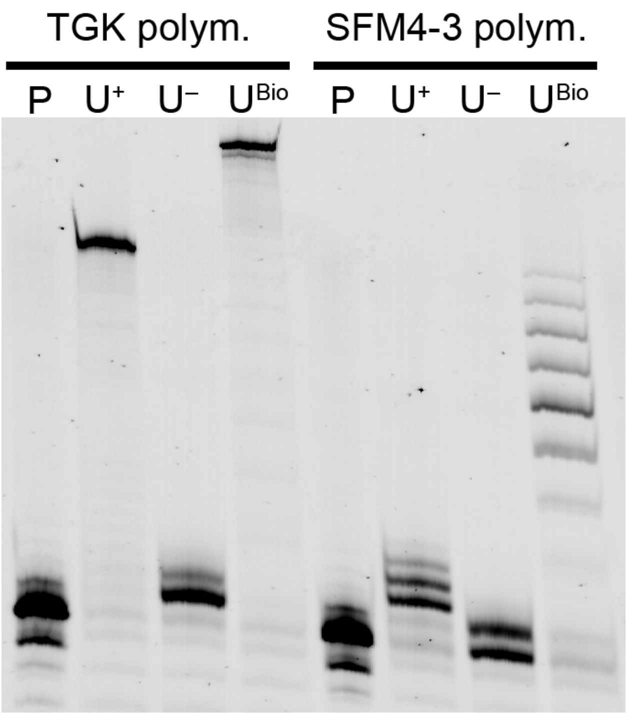

Figure S59. 12.5% dPAGE analysis. (P) RNA primer; (U^+) positive control, all natural rNTPs; ($U^–$) negative control, mixture of rATP, rCTP, rGTP and H_2O; (U^{Bio}) modification, mixture of rATP, **rU^{Bio}TP**, rCTP, rGTP. FAM scan.

2.16.5 PEX reaction with templ_50nt and TGK or SFM4-3 polymerase

Reaction was performed in total volume of 10 µL in ThermoPol buffer (1X). ssDNA template – **templ_50nt** (0.24 µM) and 5´-(Cy5)-labelled RNA primer – **Cy5-RNA-prim_15nt** (0.2 µM) in presence of ThermoPol buffer were heated up to 95 °C for 30 sec and then cooled down to 3 °C (0.1 °C s^{-1}). After addition of rGTP (0.1 mM), a mixture of the modified **rA^{Ph}TP**, **rU^{Ph}TP**, **rC^{Ph}TP** (0.1 mM, Mix-1); **rA^{E}TP**, **rU^{Bio}TP**, **rC^{Ph}TP** (0.1 mM, Mix-2); **rA^{Ph}TP**, **rU^{Bio}TP**, **rC^{mBdp}TP** (0.1 mM, Mix-3) and either TGK (0.1 µM) or SFM4-3 (0.1 µM) polymerase, the reaction mixtures were incubated at 60 °C for 2 h in a thermal cycler with heated lid (100 °C). Positive control was performed under same conditions with a mixture of natural rNTPs (0.1 mM). Six different negative control reactions were performed under same conditions using in each reaction either a different mixture of natural rNTPs [0.1 mM; r(AUC); r(AUG); r(ACG); r(UCG)] and H_2O or a mixture of rGTP (0.1 mM) and H_2O or the natural rNTPs were completely replaced by H_2O. Reaction mixtures were combined with TurboDNase (2 U) and incubated at 37 °C for 15 min, followed by incubation with Proteinase K (0.8 U) at 37 °C for 15 min. For sample preparation for dPAGE analysis see protocol in section 2.5.1. For dPAGE analysis of **50RNA_AUCG** or **50RNA_A^{Ph}U^{Ph}C^{Ph}G**, **50RNA_A^{E}U^{Bio}C^{Ph}G**, **50RNA_A^{Ph}U^{Bio}C^{mBdp}G** prepared by PEX reaction with TGK polymerase see Figure S60 and for dPAGE analysis of PEX reaction with SFM4-3 polymerase see Figure S61.

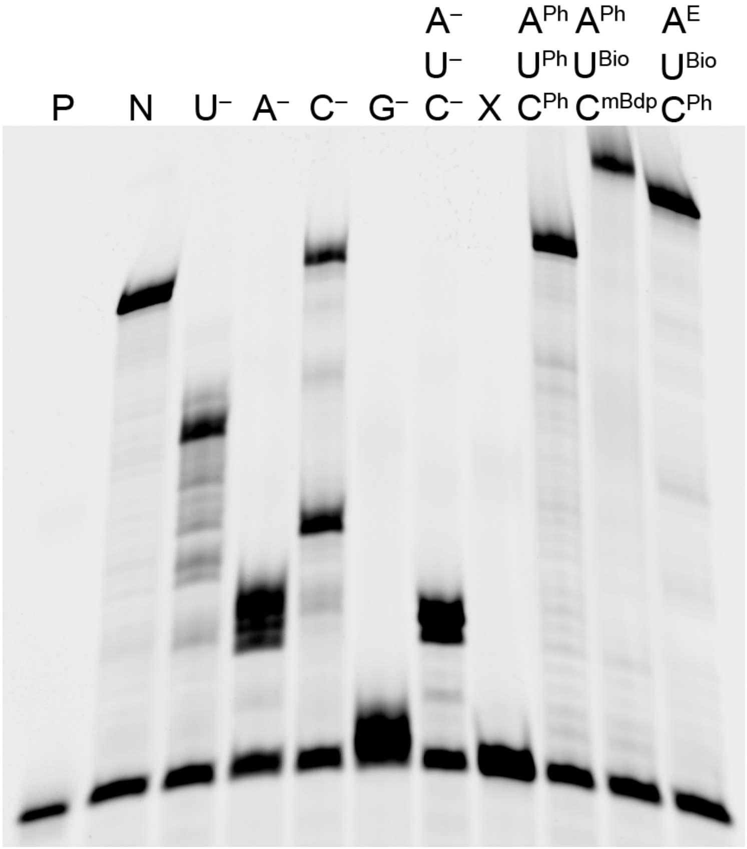

Figure S60. 12.5% dPAGE analysis of PEX reaction. (P) RNA primer; (N) positive control, all natural rNTPs; (U^{-}) negative control, mixture of rATP, rCTP, rGTP and H_2O; (A^{-}) negative control, mixture of rUTP, rCTP, rGTP and H_2O; (C^{-}) negative control, mixture of rATP, rUTP, rGTP and H_2O; (G^{-}) negative control, mixture of rATP, rUTP, rCTP and H_2O; (A^{-}, U^{-}, C^{-}) negative control, mixture of GTP and H_2O; (X) negative control, no rNTPs; (A^{Ph}, U^{Ph}, C^{Ph}) modification, mixture of **rA^{Ph}TP**, **rU^{Ph}TP**, **rC^{Ph}TP** (Mix-1) and GTP; (A^{Ph}, U^{Bio}, C^{mBdp}) modification, mixture of **rA^{Ph}TP**, **rU^{Bio}TP**, **rC^{mBdp}TP** (Mix-3) and GTP; (A^{E}, U^{Bio}, C^{Ph}) modification, mixture of **rA^{E}TP**, **rU^{Bio}TP**, **rC^{Ph}TP** (Mix-2) and GTP. Cy5 scan.

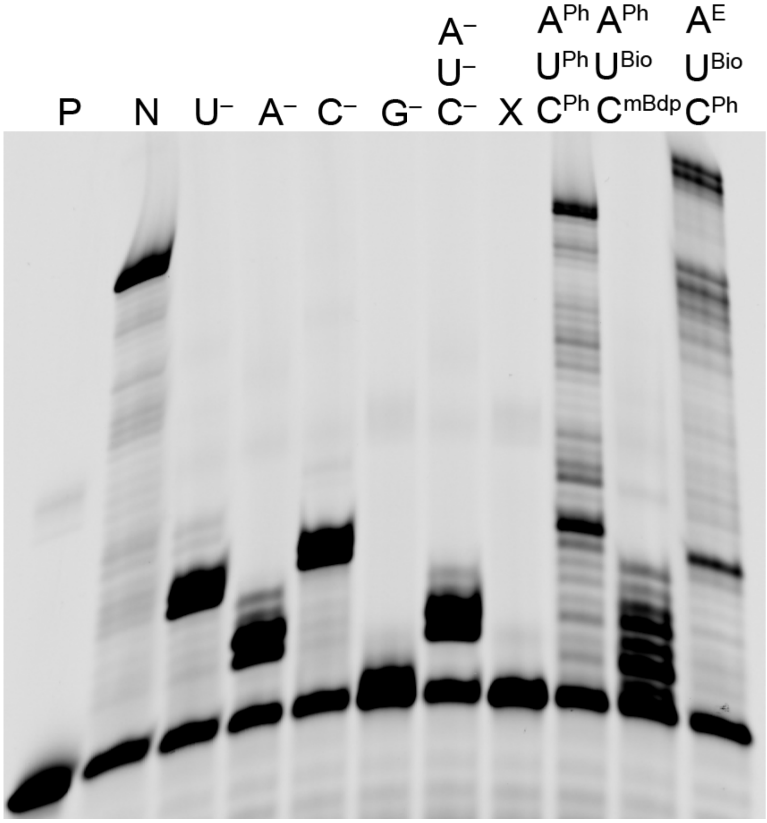

Figure S61. 12.5% dPAGE analysis of PEX reaction. (P) RNA primer; (N) positive control, all natural rNTPs; ($U^–$) negative control, mixture of rATP, rCTP, rGTP and H_2O; ($A^–$) negative control, mixture of rUTP, rCTP, rGTP and H_2O; ($C^–$) negative control, mixture of rATP, rUTP, rGTP and H_2O; ($G^–$) negative control, mixture of rATP, rUTP, rCTP and H_2O; ($A^–$, $U^–$, $C^–$) negative control, mixture of GTP and H_2O; (X) negative control, no rNTPs; (A^{Ph}, U^{Ph}, C^{Ph}) modification, mixture of **rA^{Ph}TP**, **rU^{Ph}TP**, **rC^{Ph}TP** (Mix-1) and GTP; (A^{Ph}, U^{Bio}, C^{mBdp}) modification, mixture of **rA^{Ph}TP**, **rU^{Bio}TP**, **rC^{mBdp}TP** (Mix-3) and GTP; (A^{E}, U^{Bio}, C^{Ph}) modification, mixture of **rA^{E}TP**, **rU^{Bio}TP**, **rC^{Ph}TP** (Mix-2) and GTP. Cy5 scan.

2.16.6 IVT reaction with ds-templ_poly-U and T7 RNAP

Reaction was performed in total volume of 10 µL in transcription reaction buffer (1X) containing dsDNA template – **ds-templ_poly-U** (0.5 µM, prepared according to protocol in section 2.5.3), mixture of rATP, rCTP, rGTP (0.1 mM), the modified **rU^{Bio}TP** (0.1 mM), DTT (10 mM), $MgCl_2$ (15 mM), RiboLock RNase inhibitor (10 U), Triton X-100 (0.1%), T7 RNAP (15 U) and [α-^{32}P]-GTP (111 TBq/mmol, 370 MBq/mL, 0.1 µL). Positive control was performed under same conditions with natural rUTP (0.1 mM) instead of the modified **rU^{Bio}TP**. For negative control reaction H_2O was used instead of rUTP. Transcription reactions were performed at 37 °C for 2 h in a thermal cycler with heated lid (65 °C). Crude reaction mixtures were combined with 10 µL of 2X stop solution, denatured by heating at 65 °C for 10 min and then immediately cooled on ice. Aliquots of the samples (10 µL) were subjected to vertical gel electrophoresis on 12.5% dPAGE

(19:1 mono:bis acrylamide) containing TBE buffer (1X, pH 8.0) and urea (7 M) at 40 mA, until the dye migrated to the bottom third of the gel. The gel was autoradiographed at least for 1 h and then visualised by a phosphor imager. For dPAGE analysis of **33RNA_poly-U** or **33RNA_poly-U^{Bio}** see Figure S62.

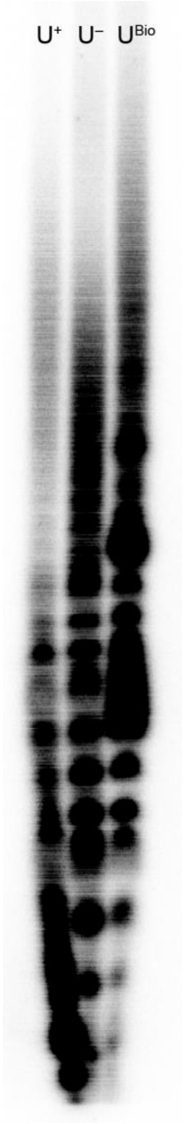

Figure S62. 12.5% dPAGE analysis. (U$^+$) positive control, all natural rNTPs; (U$^-$) negative control, mixture of rATP, rCTP, rGTP and H_2O; (U^{Bio}) modification, mixture of rATP, **rU^{Bio}TP**, rCTP, GTP/[α-^{32}P]-GTP. Phosphor imaging.

2.16.7 IVT reaction with ds-templ_52bp and T7 RNAP

Reaction was performed in total volume of 10 µL in transcription reaction buffer (1X) containing dsDNA template – **ds-templ_52bp** (0.5 µM, prepared according to protocol in section 2.5.3), rGTP (0.1 mM), a mixture of the modified **rA^{Ph}TP**, **rU^{Ph}TP**, **rC^{Ph}TP** (0.1 mM, Mix-1); **rA^{E}TP**,

rU^{Bio}TP, **rC^{Ph}TP** (0.1 mM, Mix-2); **rA^{Ph}TP**, **rU^{Bio}TP**, **rC^{mBdp}TP** (0.1 mM, Mix-3), DTT (10 mM), $MgCl_2$ (15 mM), RiboLock RNase inhibitor (10 U), Triton X-100 (0.1%), T7 RNAP (15 U) and [α-^{32}P]-GTP (111 TBq/mmol, 370 MBq/mL, 0.1 µL). Positive control was performed under same conditions with a mixture of natural rNTPs (0.1 mM). Four different negative control reactions were performed under same conditions using in each reaction either a different mixture of natural rNTPs [0.1 mM; r(AUG); r(ACG); r(UCG)] and H_2O or a mixture of rGTP (0.1 mM) and H_2O. Transcription reactions were performed at 37 °C for 2 h in a thermal cycler with heated lid (65 °C). Crude reaction mixtures were combined with 10 µL of 2X stop solution, denatured by heating at 65 °C for 10 min and then immediately cooled on ice. Aliquots of the samples (10 µL) were subjected to vertical gel electrophoresis on 12.5% dPAGE (19:1 mono:bis acrylamide) containing TBE buffer (1X, pH 8.0) and urea (7 M) at 40 mA, until the dye migrated to the bottom third of the gel. Gel was autoradiographed at least for 1 h and then visualised by a phosphor imager. For dPAGE analysis of **35RNA_AUCG** or **35RNA_A^{Ph}U^{Ph}C^{Ph}G**, **35RNA_A^{E}U^{Bio}C^{Ph}G**, **35RNA_A^{Ph}U^{Bio}C^{mBdp}G** see Figure S63.

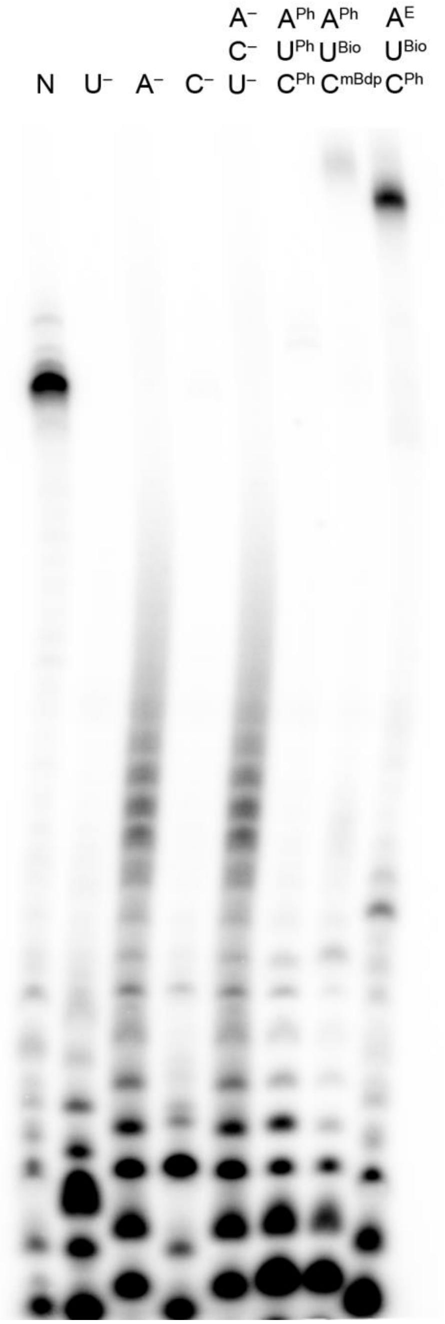

Figure S63. 12.5% dPAGE analysis of IVT reaction. (N) positive control, all natural rNTPs; ($U^–$) negative control, mixture of rATP, rCTP, rGTP and H_2O; ($A^–$) negative control, mixture of rUTP, rCTP, rGTP and H_2O; ($C^–$) negative control, mixture of rATP, rUTP, rGTP and H_2O; ($A^–$, $U^–$, $C^–$) negative control, mixture of GTP and H_2O; (A^{Ph}, U^{Ph}, C^{Ph}) modification, mixture of **rA^{Ph}TP**, **rU^{Ph}TP**, **rC^{Ph}TP** (Mix-1) and GTP/[α-^{32}P]-GTP; (A^{Ph}, U^{Bio}, C^{mBdp}) modification, mixture of **rA^{Ph}TP**, **rU^{Bio}TP**, **rC^{mBdp}TP** (Mix-3) and GTP/[α-^{32}P]-GTP; (A^E, U^{Bio}, C^{Ph}) modification, mixture of **rA^{E}TP**, **rU^{Bio}TP**, **rC^{Ph}TP** (Mix-2) and GTP/[α-^{32}P]-GTP. Phosphor imaging.

2.16.8 Analysis of TGK or SFM4-3 polymerase fidelity with imbalanced nucleotide mixture

Reactions were performed in total volume of 10 µL in ThermoPol buffer (1X) containing ssDNA template – **templ_16nt** (0.3 µM), 5´-(Cy5)-labelled RNA primer – **Cy5-RNA-prim_15nt** (0.2 µM), either TGK (0.1 µM) or SFM4-3 (0.1 µM) polymerase, mixture of rATP, rUTP, rGTP (30 µM) and the modified **rC^{mBdp}TP** at different concentrations (0.2 µM, 0.5 µM, 1.0 µM, 2.5 µM, 5.0 µM). Control reactions were performed under same conditions either only with a mixture of rATP, rUTP, rGTP (30 µM) for misincorporation or with **rC^{mBdp}TP** (5 µM) for correct nucleotide incorporation. Reaction mixtures were heated up to 95 °C for 30 sec, followed by incubation at 60 °C for 1 h in a thermal cycler with heated lid (100 °C). For sample preparation for dPAGE analysis see protocol in section 2.5.1. For dPAGE analysis of **16RNA_C^{mBdp}** and quantification see Figure S64 and Figure S65.

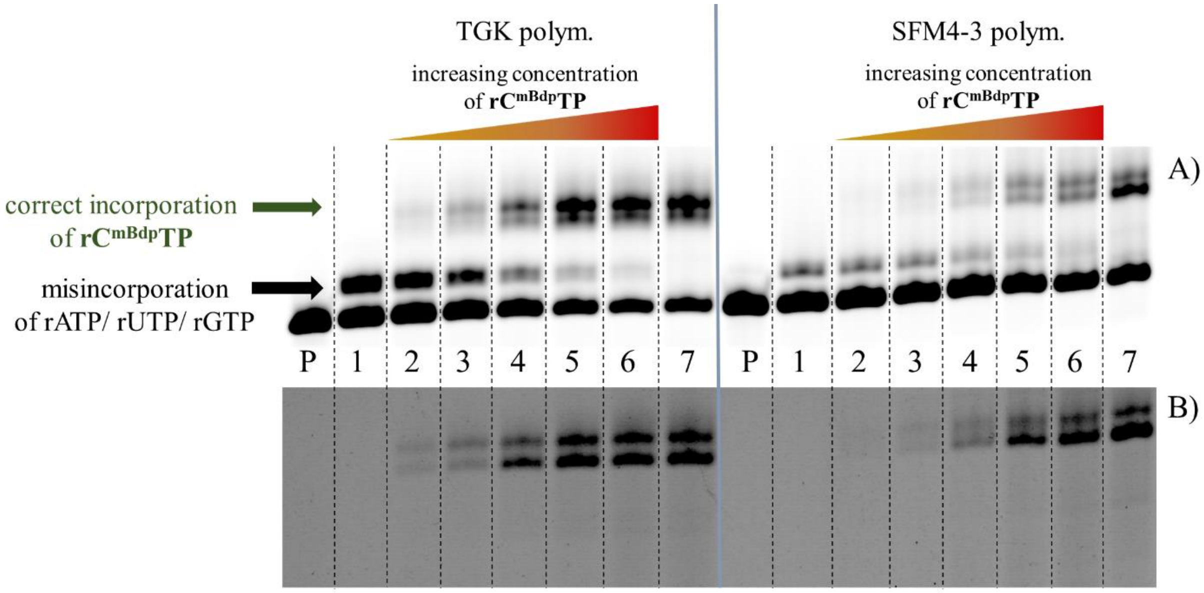

Figure S64. 15% dPAGE analysis. TGK or SFM4-3 polymerase fidelity with various concentrations of **rC^{mBdp}TP** (0.2, 0.5, 1.0, 2.5, 5.0 µM) in presence of a mixture of rATP, rUTP, rGTP (30 µM). (P) RNA primer; (1) misincorporation, PEX reaction only with a mixture of rATP, rUTP, rGTP (30 µM); (2) competitive incorporation, PEX reaction with **rC^{mBdp}TP** (0.2 µM) in presence of rATP, rUTP, rGTP (30 µM); (3) competitive incorporation, PEX reaction with **rC^{mBdp}TP** (0.5 µM) in presence of rATP, rUTP, rGTP (30 µM); (4) competitive incorporation, PEX reaction with **rC^{mBdp}TP** (1.0 µM) in presence of rATP, rUTP, rGTP (30 µM); (5) competitive incorporation, PEX reaction with **rC^{mBdp}TP** (2.5 µM) in presence of rATP, rUTP, rGTP (30 µM); (6) competitive incorporation, PEX reaction with **rC^{mBdp}TP** (5.0 µM) in presence of rATP, rUTP, rGTP (30 µM); (7) correct incorporation, PEX reaction only with **rC^{mBdp}TP** (5.0 µM). A) Cy5 scan; B) FAM scan (visualisation of mBdp-modification).

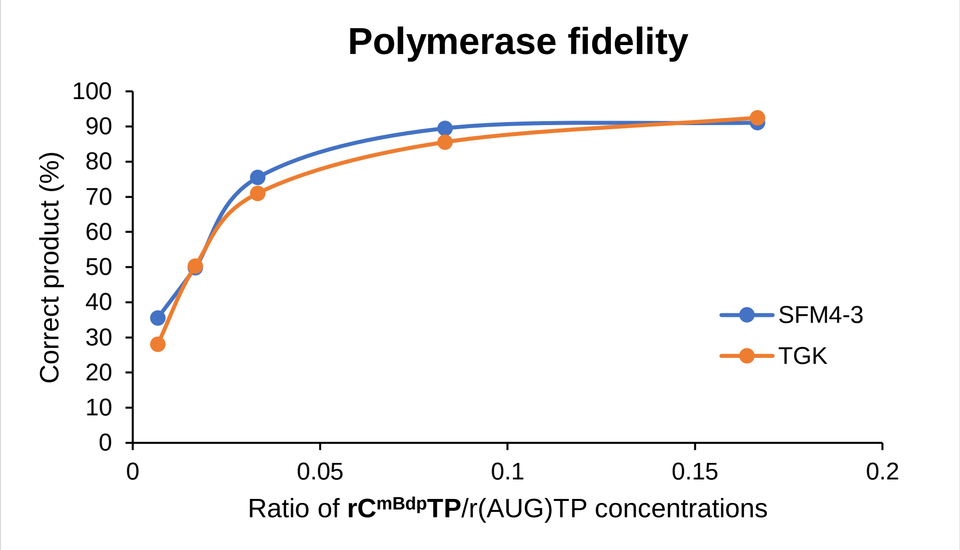

Figure S65. Determination of misincorporation rate with base-modified **rC^{mBdp}TP** in presence of rATP, rUTP, rGTP. Conversions to correct product were determined as ratios of misincorporated r(AUG)TP and correct nucleotide (**rC^{mBdp}TP**) containing gel bands densities by ImageJ.

2.17 Synthesis of base-modified RNA with cleavable DNA primer

2.17.1 Analytical PEX reaction with rC^{Cy5}TP and fluorescently labelled DNA primer

Reaction was performed in total volume of 10 µL in ThermoPol buffer (1X). ssDNA template – **templ_36nt** (4.8 µM) and 5´-(6-FAM)-labelled DNA primer – **FAM-DNA-prim_15nt** (4.0 µM) in ThermoPol buffer were heated up to 95 °C for 30 sec and then cooled down to 3 °C (0.1 °C s^{-1}). After addition of TGK polymerase (2.0 µM), mixture of rATP, rUTP, rGTP (0.1 mM) and the modified **rC^{Cy5}TP** (0.1 mM), the reaction was incubated at 60 °C for 2 h in a thermal cycler with heated lid (100 °C). Positive control was performed under same conditions with natural rCTP (0.1 mM) instead of modified **rC^{Cy5}TP**. For negative control reaction H_2O was used instead of rCTP. After PEX, the crude reaction mixture was purified using QIAquick nucleotide removal kit, followed by elution with 10 µL of H_2O. For sample preparation for dPAGE analysis see protocol in section 2.5.1. For optimalisation of DNA primer cleavage, the PEX reaction with **rC^{Cy5}TP** was 3X scaled-up and the generated modified **36DNA-RNA_C^{Cy5}** was purified using QIAquick nucleotide removal kit, followed by elution with 10 µL of H_2O. The purified mixture (10 µL) was combined with various amounts of TurboDNase (2 U, 6 U, 10 U) and incubated at 37 °C for 2 h to generate the desired cleavage product (**RNA_C^{Cy5}**). After this time, aliquots for gel analysis were removed and the reaction was further incubated at 37 °C for another 2 h (4 h in total). Afterwards, aliquots were removed for gel analysis. For sample preparation for dPAGE analysis see protocol in section 2.5.1. For dPAGE analysis of PEX reaction followed by TurboDNase cleavage see Figure S66.

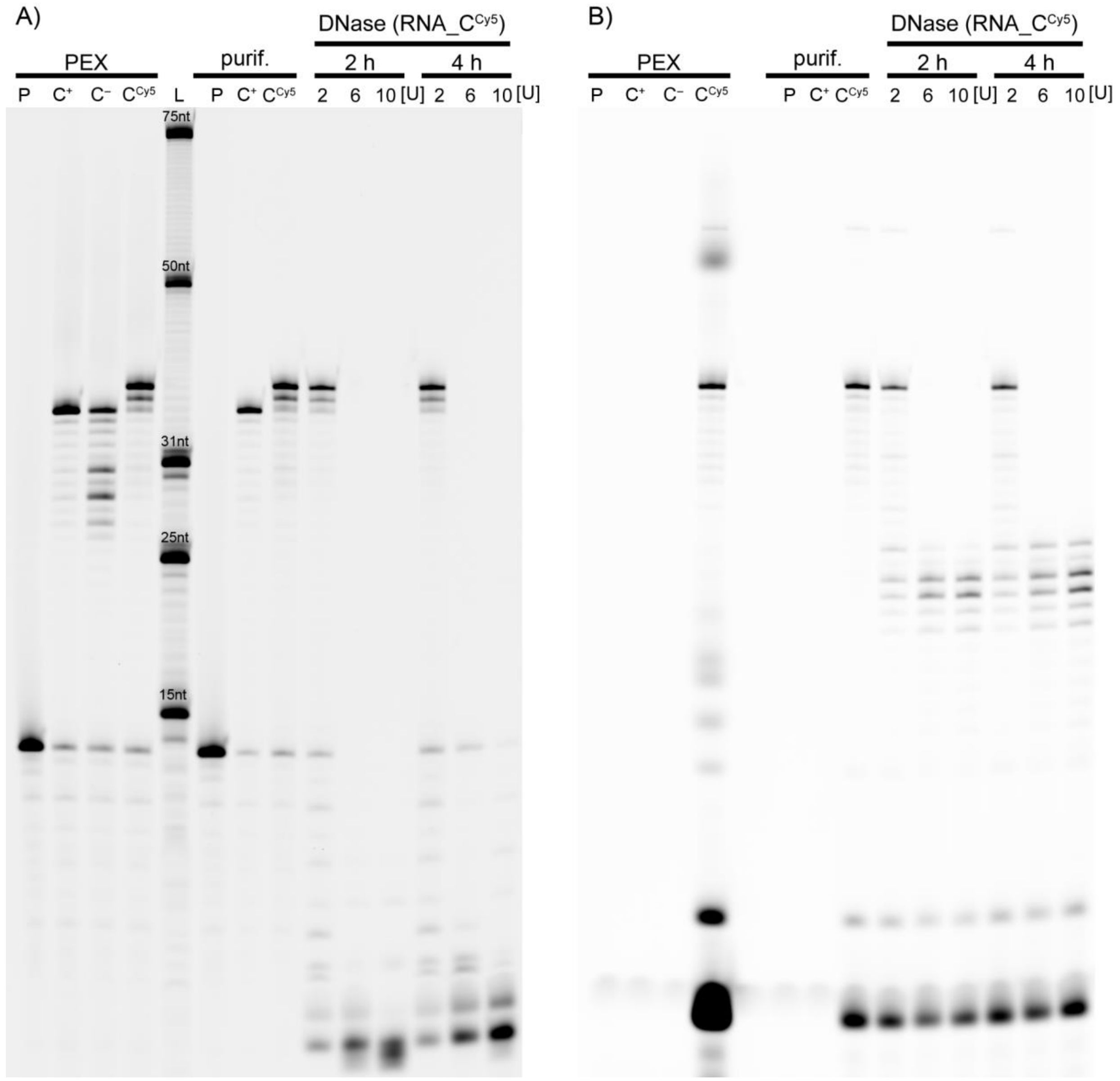

Figure S66. 12.5% dPAGE analysis. (P) DNA primer; (C^+) positive control, all natural rNTPs; (C^-) negative control, mixture of rATP, rUTP, rGTP and H_2O; (C^{Cy5}) modification, mixture of rATP, rUTP, **rC^{Cy5}TP**, rGTP; (L) RNA ladder composed of FAM-labelled RNA oligonucleotides of indicated length. TurboDNase cleavage of modified **36DNA-RNA_C^{Cy5}** with 2 U, 6 U, 10 U of the enzyme for 2 h or 4 h incubation time. A) FAM scan; B) Cy5 scan (visualisation of Cy5-modification).

2.17.2 Analytical PEX reaction with rC^{Cy5}TP and fluorescently labelled DNA primer with internal dU modification

Reaction was performed in total volume of 10 µL in ThermoPol buffer (1X). ssDNA template – **templ_36nt** (4.8 µM) and 5´-(6-FAM)-labelled DNA primer with internal dU modification –

FAM-DNA-prim_dU (4.0 µM) in ThermoPol buffer were heated up to 95 °C for 30 sec and then cooled down to 3 °C (0.1 °C s^{-1}). After addition of TGK polymerase (1.5 µM), mixture of rATP, rUTP, rGTP (0.1 mM) and the modified **rC^{Cy5}TP** (0.1 mM), the reaction was incubated at 60 °C for 2 h in a thermal cycler with heated lid (100 °C). Positive control was performed under same conditions with natural rCTP (0.1 mM) instead of the modified **rC^{Cy5}TP**. For negative control reaction H_2O was used instead of rCTP. Crude reaction mixture was purified using QIAquick nucleotide removal kit, followed by elution with 10 µL of H_2O. For sample preparation for dPAGE analysis see protocol in section 2.5.1. For cleavage of the DNA primer, the purified mixture (**36DNA_dU-RNA_C^{Cy5}**) was combined with UDG buffer (1 µL, 10X), UDG (2 U) and further incubated at 37 °C for 30 min. Solution of DMEDA (1.2 µL, 1 M, pH 8.5) was added and again incubated at 37 °C for further 30 min to generate the desired cleavage product (**RNA_C^{Cy5}**). After that, TurboDNase (2 U) was added and incubated at 37 °C for 1 h. For sample preparation for dPAGE analysis see protocol in section 2.5.1. For dPAGE analysis of PEX reaction followed by UDG cleavage see Figure S67.

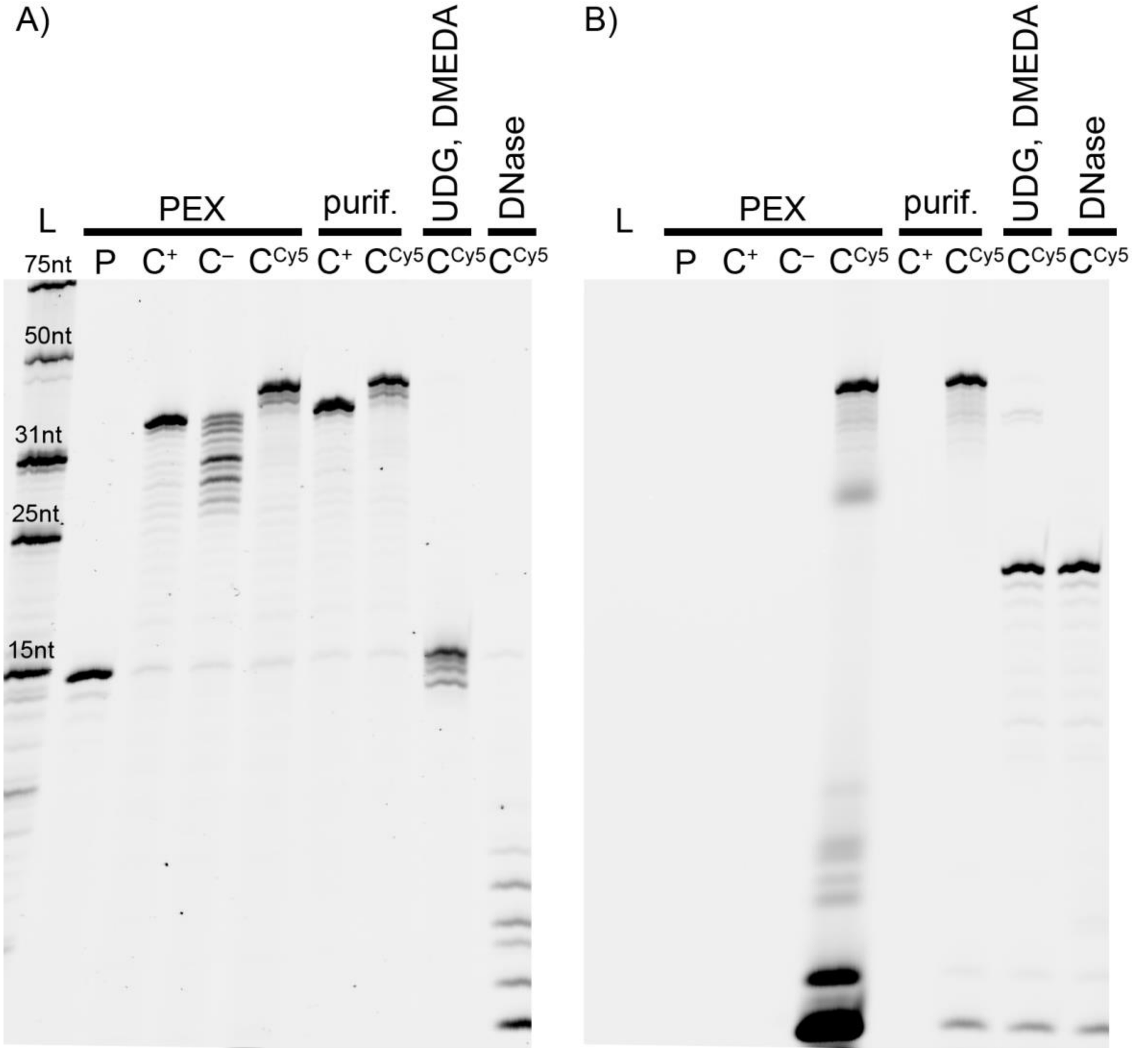

Figure S67. 22.5% dPAGE analysis. (L) RNA ladder composed of FAM-labelled RNA oligonucleotides of indicated length; (P) DNA primer; (C$^+$) positive control, all natural rNTPs; (C$^-$) negative control, mixture of rATP, rUTP, rGTP and H_2O; (C^{Cy5}) modification, mixture of rATP, rUTP, **rC^{Cy5}TP**, rGTP. (UDG, DMEDA) Sample analysis after UDG followed by DMEDA cleavage. (DNase) Sample analysis after TurboDNase cleavage. A) FAM scan; B) Cy5 scan (visualisation of Cy5-modification).

2.17.3 Analytical PEX reaction with rA^{E}TP, rU^{Bio}TP, rC^{Cy5}TP, rG^{Pent}TP and fluorescently labelled DNA primer with internal dU modification

Reaction was performed in total volume of 10 µL in ThermoPol buffer (1X). ssDNA template – **templ_36nt** (4.8 µM) and 5´-(6-FAM)-labelled DNA primer with internal dU modification – **FAM-DNA-prim_dU** (4.0 µM) in ThermoPol buffer were heated up to 95 °C for 30 sec and then cooled down to 3 °C (0.1 °C s^{-1}). After addition of TGK polymerase (1.5 µM) and a mixture of **rA^{E}TP**, **rU^{Bio}TP**, **rC^{Cy5}TP**, **rG^{Pent}TP** (0.1 mM), the reaction was incubated at 60 °C for 2 h in a thermal cycler with heated lid (100 °C). Positive control was performed under same conditions

with a mixture of natural rNTPs (0.1 mM) instead of the modified **rN^{X}TP**s. Five different negative control reactions were performed under same conditions using in each reaction a different mixture of three natural rNTPs [0.1 mM; r(AUC); r(AUG); r(ACG); r(UCG)] and H_2O or by complete replacement of natural rNTPs by H_2O. Crude reaction mixture was purified using QIAquick nucleotide removal kit, followed by elution with 10 µL of H_2O. For sample preparation for dPAGE analysis see protocol in section 2.5.1. For cleavage of the DNA primer, the purified mixture (**36DNA_dU-RNA_A^{E}U^{Bio}C^{Cy5}G^{Pent}**) was combined with UDG buffer (1 µL, 10X), UDG (2 U) and further incubated at 37 °C for 30 min to generate the desired cleavage product (**RNA_A^{E}U^{Bio}C^{Cy5}G^{Pent}**). Solution of DMEDA (1.2 µL, 1 M, pH 8.5) was added and again incubated at 37 °C for further 30 min. After that, TurboDNase (2 U) was added and incubated at 37 °C for 1 h. For sample preparation for dPAGE analysis see protocol in section 2.5.1. For dPAGE analysis of PEX reaction see Figure S68 and for UDG cleavage see Figure S69.

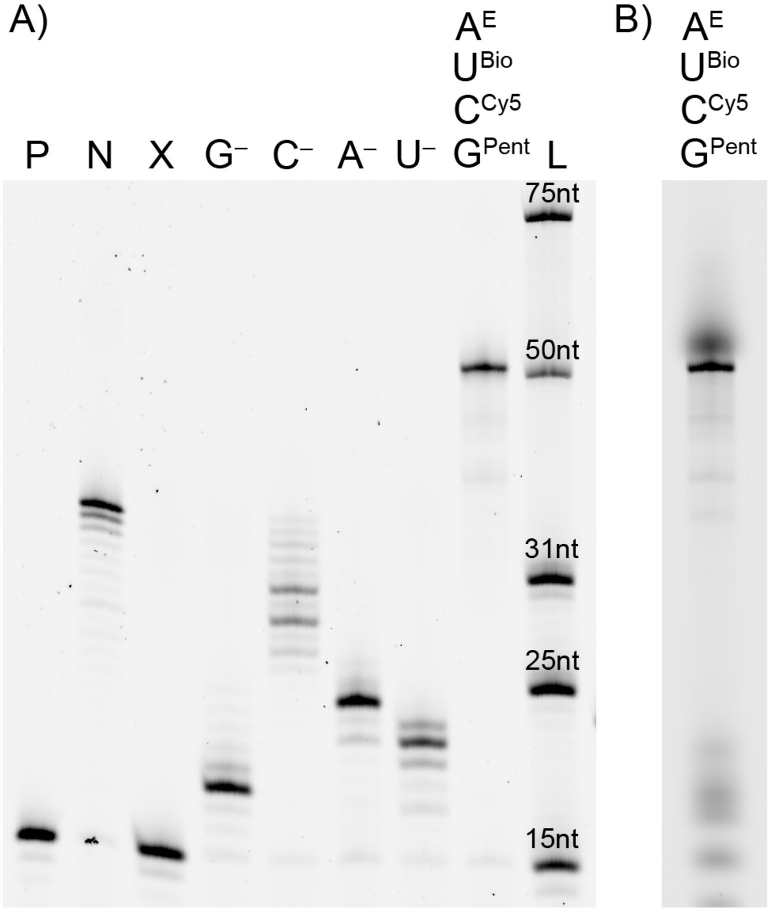

Figure S68. 12.5% dPAGE analysis of PEX reaction. (P) DNA primer; (N) positive control, all natural rNTPs; (X) negative control, no rNTPs; (G^{-}) negative control, mixture of rATP, rUTP, rCTP and H_2O; (C^{-}) negative control, mixture of rATP, rUTP, rGTP and H_2O; (A^{-}) negative control, mixture of rUTP, rCTP, rGTP and H_2O; (U^{-}) negative control, mixture of rATP, rCTP, rGTP and H_2O; (A^{E}, U^{Bio}, C^{Cy5}, G^{Pent}) modification, mixture of **rA^{E}TP**, **rU^{Bio}TP**, **rC^{Cy5}TP**, **rG^{Pent}TP**; (L) RNA ladder composed of FAM-labelled RNA oligonucleotides of indicated length. A) FAM scan; B) Cy5 scan (visualisation of Cy5-modification).

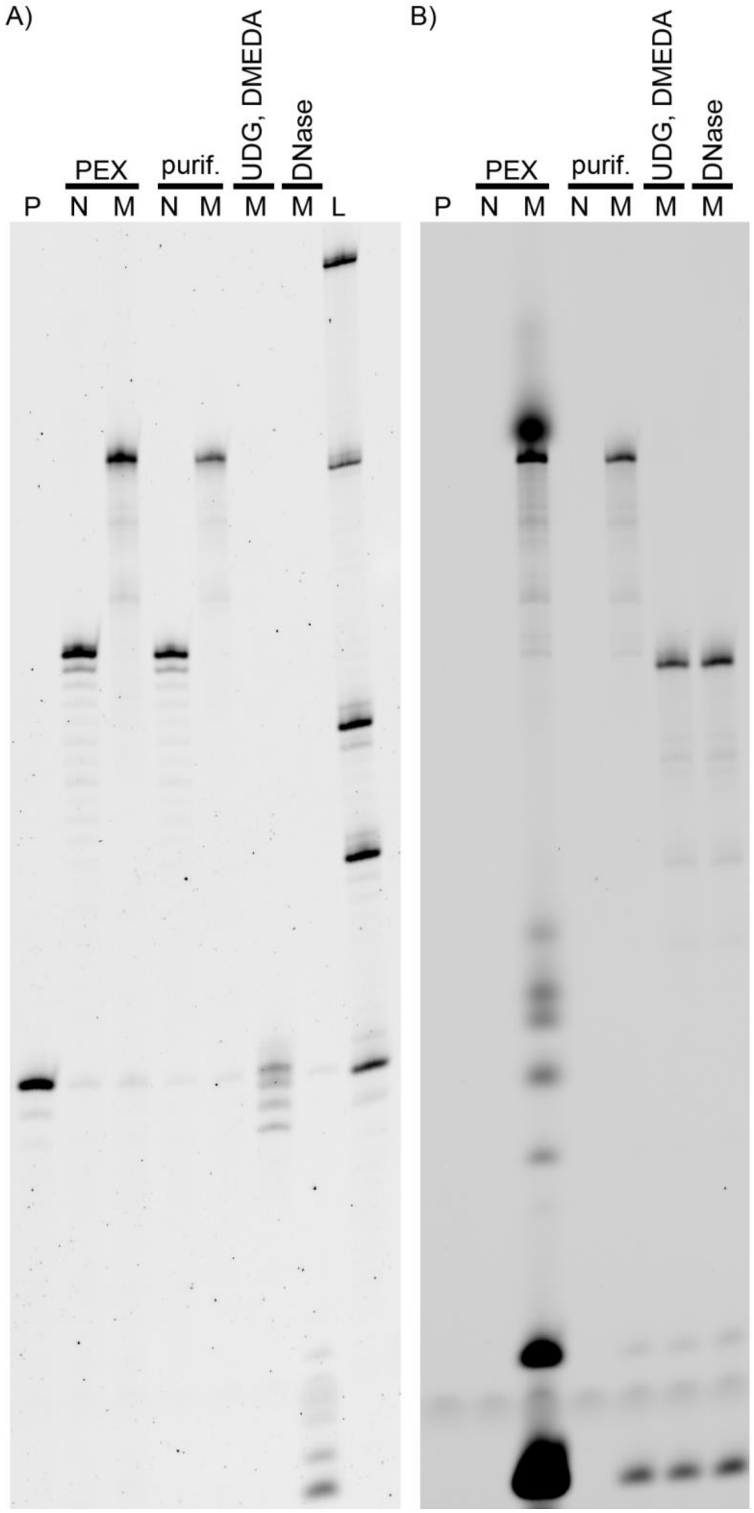

Figure S69. 12.5% dPAGE analysis of PEX reaction. (P) DNA primer; (N) positive control, all natural rNTPs; (M) modification, mixture of **rA^{E}TP**, **rU^{Bio}TP**, **rC^{Cy5}TP**, **rG^{Pent}TP**; (L) RNA ladder composed of FAM-labelled RNA oligonucleotides of indicated length. (UDG, DMEDA) Sample analysis after UDG followed by DMEDA cleavage. (DNase) Sample analysis after TurboDNase cleavage. A) FAM scan; B) Cy5 scan (visualisation of Cy5-modification).

2.17.4 Analytical PEX reaction with rA^{E}TP, rU^{Bio}TP, rC^{Ph}TP, rG^{Pent}TP and dual fluorescently labelled DNA primer with internal dU modification

Reaction was performed in total volume of 10 µL in ThermoPol buffer (1X). ssDNA template – **templ_35nt** (4.8 µM) and dually labelled DNA primer with 5´-(Cy5) and internal dU and dT-(FAM) modification – **Cy5-DNA-prim_dU_dT-FAM** (4.0 µM) in ThermoPol buffer were heated up to 95 °C for 30 sec and then cooled down to 3 °C (0.1 °C s^{-1}). After addition of TGK polymerase (2.0 µM) and a mixture of **rA^{E}TP**, **rU^{Bio}TP**, **rC^{Ph}TP**, **rG^{Pent}TP** (0.2 mM), the reaction was incubated at 60 °C for 2 h in a thermal cycler with heated lid (100 °C). Positive control was performed under same conditions with a mixture of natural rNTPs (0.2 mM) instead of the modified **rN^{X}TP**s. Five different negative control reactions were performed under same conditions using in each reaction a different mixture of three natural rNTPs [0.2 mM; r(AUC), r(AUG), r(ACG), r(UCG)] and H_2O or by complete replacement of natural rNTPs by H_2O. For sample preparation for dPAGE analysis see protocol in section 2.5.1. For cleavage of the DNA primer, the crude mixture (**35DNA_dU_dT^{FAM}-RNA_A^{E}U^{Bio}C^{Ph}G^{Pent}**) was combined with UDG buffer (1 µL, 10X), UDG (2 U) and further incubated at 37 °C for 30 min. Solution of DMEDA (1.2 µL, 1 M, pH 8.5) was added and again incubated at 37 °C for further 30 min to generate the desired cleavage product (**dT^{FAM}-RNA_A^{E}U^{Bio}C^{Ph}G^{Pent}**). After that, TurboDNase (2 U) was added and incubated at 37 °C for 1 h. For sample preparation for dPAGE analysis see protocol in section 2.5.1. For dPAGE analysis of PEX reaction see Figure S70 and for UDG cleavage see Figure S71.

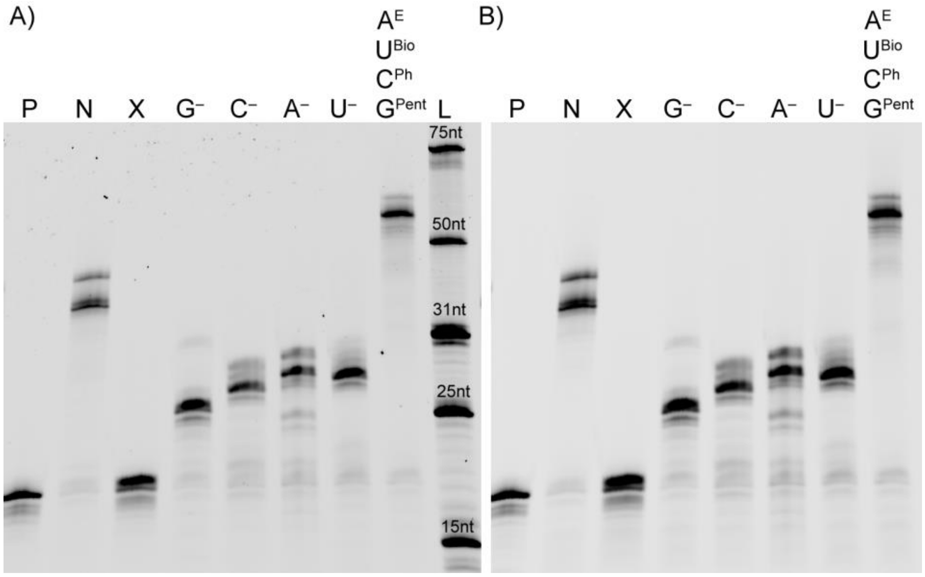

Figure S70. 22.5% dPAGE analysis of PEX reaction. (P) DNA primer; (N) positive control, all natural rNTPs; (X) negative control, no rNTPs; (G^{-}) negative control, mixture of rATP, rUTP, rCTP and H_2O; (C^{-}) negative control, mixture of rATP, rUTP, rGTP and H_2O; (A^{-}) negative control, mixture of rUTP, rCTP, rGTP and H_2O; (U^{-}) negative control, mixture of rATP, rCTP, rGTP and H_2O; (A^{E}, U^{Bio}, C^{Ph}, G^{Pent}) modification, mixture of **rA^{E}TP**, **rU^{Bio}TP**, **rC^{Ph}TP**, **rG^{Pent}TP**; (L) RNA ladder composed of FAM-labelled RNA oligonucleotides of indicated length. A) FAM-scan; B) Cy5-scan.

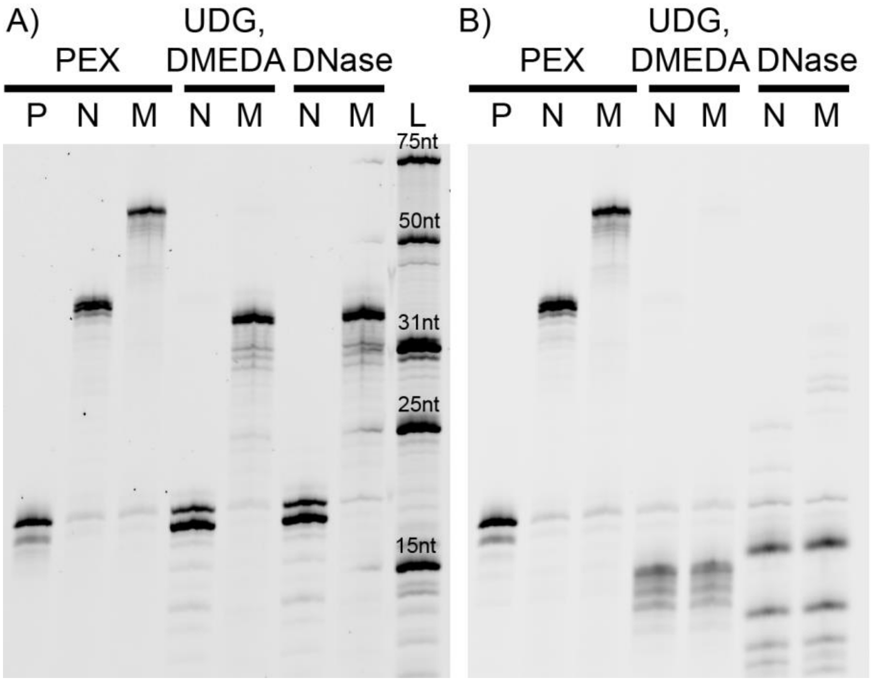

Figure S71. 22.5% dPAGE analysis. (P) DNA primer; (N) positive control, all natural rNTPs; (M) modification, mixture of **rA^{E}TP**, **rU^{Bio}TP**, **rC^{Ph}TP**, **rG^{Pent}TP**; (L) RNA ladder composed of FAM-labelled RNA oligonucleotides of indicated length. (UDG, DMEDA) Sample analysis after UDG followed by DMEDA cleavage. (DNase) Sample analysis after TurboDNase cleavage. A) FAM scan; B) Cy5 scan.

2.17.5 Semi-preparative PEX reaction with rC^{Cy5}TP and fluorescently labelled DNA primer with internal dU modification

Reaction was performed in total volume of 50 µL in ThermoPol buffer (1X). ssDNA template – **templ_36nt** (4.8 µM) and 5´-(6-FAM)-labelled DNA primer with internal dU modification – **FAM-DNA-prim_dU** (4.0 µM) in ThermoPol buffer were heated up to 95 °C for 30 sec and then cooled down to 3 °C (0.1 °C s^{-1}). After addition of TGK polymerase (1.5 µM), mixture of rATP, rUTP, rGTP (0.1 mM) and the modified **rC^{Cy5}TP** (0.1 mM), the reaction was incubated at 60 °C for 2 h in a thermal cycler with heated lid (100 °C). Crude reaction mixture was purified using QIAquick nucleotide removal kit, followed by elution with 50 µL of H_2O. For cleavage of the DNA primer, the purified mixture (**36DNA_dU-RNA_C^{Cy5}**) was combined with UDG buffer (5 µL, 10X), UDG (10 U) and further incubated at 37 °C for 30 min. Solution of DMEDA (6 µL, 1 M, pH 8.5) was added and again incubated at 37 °C for further 30 min. After that, TurboDNase (10 U) was added and incubated at 37 °C for 1 h. Sample was purified using Microspin G-25 columns, evaporated to dryness, again diluted in 10 µL of water prior to analysis. For mass spectrometry analysis of the cleaved product **RNA_C^{Cy5}** see Figure S225 and Figure S226.

2.17.6 Semi-preparative PEX reaction with rA^{E}TP, rU^{Bio}TP, rC^{Cy5}TP, rG^{Pent}TP and fluorescently labelled DNA primer with internal dU modification

Reaction was performed in total volume of 50 µL in ThermoPol buffer (1X). ssDNA template – **templ_36nt** (4.8 µM) and 5´-(6-FAM)-labelled DNA primer with internal dU modification – **FAM-DNA-prim_dU** (4.0 µM) in ThermoPol buffer were heated up to 95 °C for 30 sec and then cooled down to 3 °C (0.1 °C s^{-1}). After addition of TGK polymerase (1.5 µM) and a mixture of **rA^{E}TP**, **rU^{Bio}TP**, **rC^{Cy5}TP**, **rG^{Pent}TP** (0.1 mM), the reaction was incubated at 60 °C for 2 h in a thermal cycler with heated lid (100 °C). Crude reaction mixture was purified using QIAquick nucleotide removal kit, followed by elution with 50 µL of H_2O. For cleavage of the DNA primer, the purified mixture (**36DNA_dU-RNA_A^{E}U^{Bio}C^{Cy5}G^{Pent}**) was combined with UDG buffer (5 µL, 10X), UDG (10 U) and further incubated at 37 °C for 30 min. Solution of DMEDA (6 µL, 1 M, pH 8.5) was added and again incubated at 37 °C for further 30 min. After that, TurboDNase (10 U) was added and incubated at 37 °C for 1 h. Sample was purified using Microspin G-25 columns, evaporated to dryness, again diluted in 10 µL of water prior to analysis. For mass spectrometry analysis of the cleaved product **RNA_A^{E}U^{Bio}C^{Cy5}G^{Pent}** see Figure S227 and Figure S228.

2.17.7 Semi-preparative PEX reaction with rA^{E}TP, rU^{Bio}TP, rC^{Ph}TP, rG^{Pent}TP and dual fluorescently labelled DNA primer with internal dU modification

Reaction was performed in total volume of 50 µL in ThermoPol buffer (1X). ssDNA template – **templ_35nt** (4.8 µM) and dually labelled DNA primer with 5´-(Cy5) and internal dU and dT-(FAM) modification – **Cy5-DNA-prim_dU_dT-FAM** (4.0 µM) in ThermoPol buffer were heated up to 95 °C for 30 sec and then cooled down to 3 °C (0.1 °C s^{-1}). After addition of TGK polymerase (2.0 µM) and a mixture of **rA^{E}TP**, **rU^{Bio}TP**, **rC^{Ph}TP**, **rG^{Pent}TP** (0.2 mM), the reaction was incubated at 60 °C for 2 h in a thermal cycler with heated lid (100 °C). The crude mixture containing the desired PEX product (**35DNA_dU_dT^{FAM}-RNA_A^{E}U^{Bio}C^{Ph}G^{Pent}**) was combined with UDG buffer (5 µL, 10X), UDG (10 U) and further incubated at 37 °C for 30 min. Solution of DMEDA (6 µL, 1 M, pH 8.5) was added and again incubated at 37 °C for further 30 min. After that, TurboDNase (10 U) was added and incubated at 37 °C for 1 h. Sample was purified using Microspin G-25 columns, evaporated to dryness, again diluted in 10 µL of water prior to analysis. For mass spectrometry analysis of the cleaved product **dT^{FAM}-RNA_A^{E}U^{Bio}C^{Ph}G^{Pent}** see Figure S229 and Figure S230.

2.18 Selective fluorescent RNA labelling at specific position for structural studies

2.18.1 Analytical scale PEX reaction for preparation of FAM-Cy5-Cy3-riboswitch

Reaction mixture containing ssDNA template – **templ_ribosw71_A** (0.48 µL, 100 µM) and 5´-(6-FAM)-labelled RNA primer – **FAM-RNA-prim_23nt** (0.4 µL, 100 µM) in presence of ThermoPol buffer (1 µL, 10X) was heated up to 95 °C for 30 sec and then cooled down to 3 °C (0.1 °C s^{-1}). SNI-1: After additon of **rU^{Cy5}TP** (1 µL, 1 mM) and TGK polymerase (0.4 µL, 50 µM), the final reaction mixture (7 µL) was incubated at 45 °C for 15 min. Mixture was combined with

rSAP (1 U) and incubated at 37 °C for 15 min, followed by heat denaturation at 65 °C for 10 min. PEX-1: Reaction was combined with a mixture of natural rNTPs (2.0 µL, 4 mM), additional amount of TGK polymerase (1.0 µL, 10 µM) and incubated at 45 °C for 15 min, followed by incubation at 60 °C for 15 min. Then the mixture was combined with rSAP (1 U), TurboDNase (2 U) and incubated at 37 °C for 30 min, followed by heat denaturation at 75 °C for 15 min. After addition of second ssDNA template – **templ_ribosw71_B** (0.5 µL, 100 µM), the mixture was heated up to 95 °C for 30 sec and then cooled down to 3 °C (0.1 °C s^{-1}). SNI-2: After addition of **rU^{Cy3}TP (**1 µL, 1 mM) and TGK polymerase (1.5 µL, 10 µM) the mixture was incubated at 60 °C for 15 min. PEX-2: Followed by addition of a mixture of natural rNTPs (2.0 µL, 16 mM), TGK polymerase (2.0 µL, 10 µM) and further incubation at 60 °C for 2 h. Finally, sample (**FAM-Cy5-Cy3-riboswitch**) was treated by rSAP (1 U) and TurboDNase (2 U) at 37 °C for 30 min, followed by addition of Proteinase K (1.6 U) and further incubation at 37 °C for 30 min. Crude reaction mixtures containing either the reaction intermediates (after SNI-1, PEX-1, SNI-2) or the final product (after PEX-2, **FAM-Cy5-Cy3-riboswitch**) were after TurboDNase and Proteinase K treatment purified using QIAquick nucleotide removal kit according to standard supplier´s protocol, prior to dPAGE analysis following the protocol in section 2.5.1. For dPAGE analysis see Figure S72. For quantification of the reaction conversion from crude reaction mixture using ImageJ see Figure S73.

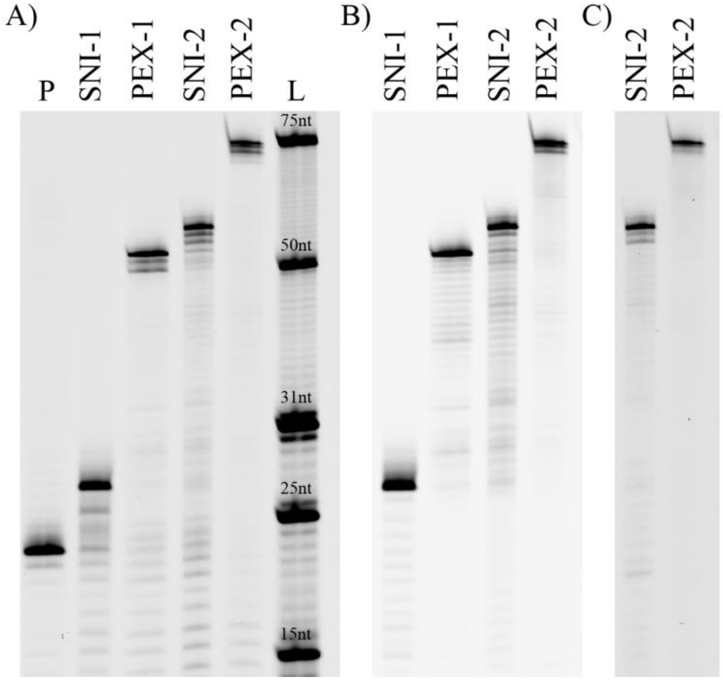

Figure S72. 12.5% dPAGE analysis of purified RNA products. (P) RNA primer; (SNI-1) Product after SNI incorporation of **rU^{Cy5}TP**; (PEX-1) Product after following PEX with natural rNTPs; (SNI-2) Product after following SNI of **rU^{Cy3}TP**; (PEX-2) Product after final PEX with natural rNTPs; (L) RNA ladder composed of FAM-labelled RNA oligonucleotides of indicated length. A) FAM-scan; B) Cy5-scan (visualisation of Cy5-modification); C) Cy3-scan (visualisation of Cy3-modification).

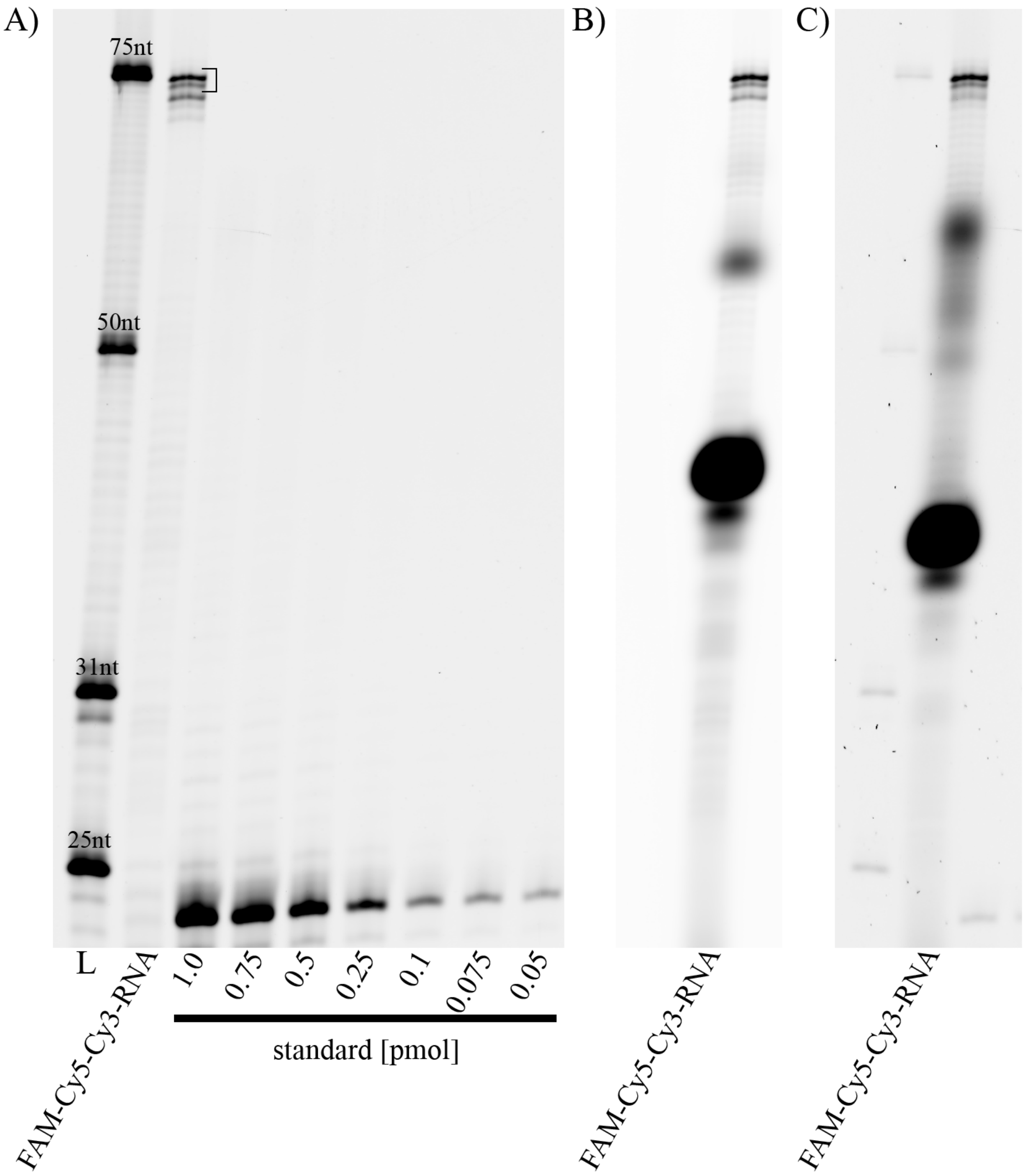

Figure S73. 12.5% dPAGE analysis of crude RNA product. (L) RNA ladder composed of FAM-labelled RNA oligonucleotides of indicated length. (FAM-Cy5-Cy3-RNA) Standard of full- length **FAM-Cy5-Cy3-riboswitch**; (standard) Decreasing amount (1.0, 0.75, 0.5, 0.25, 0.1, 0.075, 0.05 pmol) of FAM-labelled RNA oligonucleotide standard. A) FAM scan; B) Cy5 scan (visualisation of Cy5-modification); C) Cy3 scan (visualisation of Cy3-modification). Marked area in FAM scan was used for ImageJ quantification.

2.18.2 Semi-preparative scale PEX reaction for preparation of FAM-Cy5-Cy3-riboswitch

Reaction mixture containing ssDNA template – **templ_ribosw71_A** (0.96 µL, 100 µM) and 5´-(6-FAM)-labelled RNA primer – **FAM-RNA-prim_23nt** (0.8 µL, 100 µM) in presence of ThermoPol buffer (2 µL, 10X) was heated up to 95 °C for 30 sec and then cooled down to 3 °C

(0.1 °C s^{-1}) in a thermal cycler with heated lid (100 °C). SNI-1: After additon of **rU^{Cy5}TP** (2 µL, 1 mM) and TGK polymerase (0.8 µL, 50 µM), the final reaction mixture (14 µL) was incubated at 45 °C for 15 min. Mixture was combined with rSAP (1 U) and incubated at 37 °C for 30 min, followed by heat denaturation at 65 °C for 10 min. PEX-1: Reaction was combined with a mixture of natural rNTPs (4.0 µL, 4 mM), additional amount of TGK polymerase (2.0 µL, 10 µM) and incubated at 45 °C for 15 min, followed by incubation at 60 °C for 15 min. Then the mixture was combined with rSAP (2 U), TurboDNase (4 U) and incubated at 37 °C for 30 min, followed by heat denaturation at 75 °C for 15 min. After addition of second ssDNA template – **templ_ribosw71_B** (1.0 µL, 100 µM), the mixture was heated up to 95 °C for 30 sec and then cooled down to 3 °C (0.1 °C s^{-1}). SNI-2: After addition of **rU^{Cy3}TP (**2 µL, 1 mM) and TGK polymerase (3 µL, 10 µM) the mixture was incubated at 60 °C for 15 min. PEX-2: Followed by addition of a mixture of natural rNTPs (4.0 µL, 16 mM), TGK polymerase (4.0 µL, 10 µM) and further incubation at 60 °C for 2 h. The final product (**FAM-Cy5-Cy3-riboswitch**) was treated with TurboDNase (6 U) at 37 °C for 30 min, followed by addition of Proteinase K (2.4 U) and further incubation at 37 °C for 30 min. Crude reaction mixtures containing either reaction intermediates (after SNI-1, PEX-1, SNI-2) or the final product (after PEX-2, **FAM-Cy5-Cy3-riboswitch**) were after TurboDNase and Proteinase K treatment purified using QIAquick nucleotide removal kit according to standard supplier´s protocol. Samples were two times eluted with 15 µL of H_2O, lyophilised to dryness and again diluted in 10 µL of H_2O prior to analysis. For mass spectrometry analysis of the reaction intermediates or final product see Figure S231 and Figure S232 (SNI-1), Figure S233 and Figure S234 (PEX-1), Figure S235 and Figure S236 (SNI-2), Figure S237 and Figure S238 (PEX-2, final **FAM-Cy5-Cy3-riboswitch**).

2.18.3 Preparative scale PEX reaction for preparation of Cy5-Cy3-riboswitch

Reaction was performed following the procedure in section 2.18.2, just 10X scaled up and with usage of non-fluorescently labelled RNA primer – **RNA-prim_23nt**. Crude reaction mixture was purified using QIAquick nucleotide removal kit according to standard supplier´s protocol and evaporated to dryness. Aliquot of the sample was again diluted in 10 µL of H_2O prior to mass analysis and the remaining sample was used in section 2.18.4. For mass spectrometry analysis see Figure S239 and Figure S240.

2.18.4 FRET measurements of the Cy5-Cy3-riboswitch

The sample (**Cy5-Cy3-riboswitch**) from section 2.18.3 was diluted with TRIS buffer (50 mM, pH 7.5), NaCl (50 mM) and $MgSO_4$ (4 mM) to final 1 µM concentration. Reaction mixture (100 µL) was heated up to 95 °C for 30 sec and then cooled down to 3 °C (0.1 °C s^{-1}). Afterwards, the reaction mix was combined with urea (4 M) and increasing concentration of adenine ligand (5 µM, 10 µM, 100 µM, 1 mM, 1.5 mM, 2 mM, 2.5 mM, 3 mM, 4 mM, 8 mM). The mixture was transferred to a 100 µL quartz cuvette and fluorescent spectra were measured at 25 °C. Fluorescence spectrum was recorded with the excitation wavelength λex = 530 nm and the range

of the emission spectra was 540-800 nm. The negative control FRET measurements were performed under same conditions with usage of a mixture of Cy5-labelled (**Cy5-DNA-oligo**) and Cy3-labelled (**Cy3-DNA-oligo**) ssDNA oligonucleotides. All experiments were performed in triplicates. For analysis of normalized FRET measurements see Figure S74.

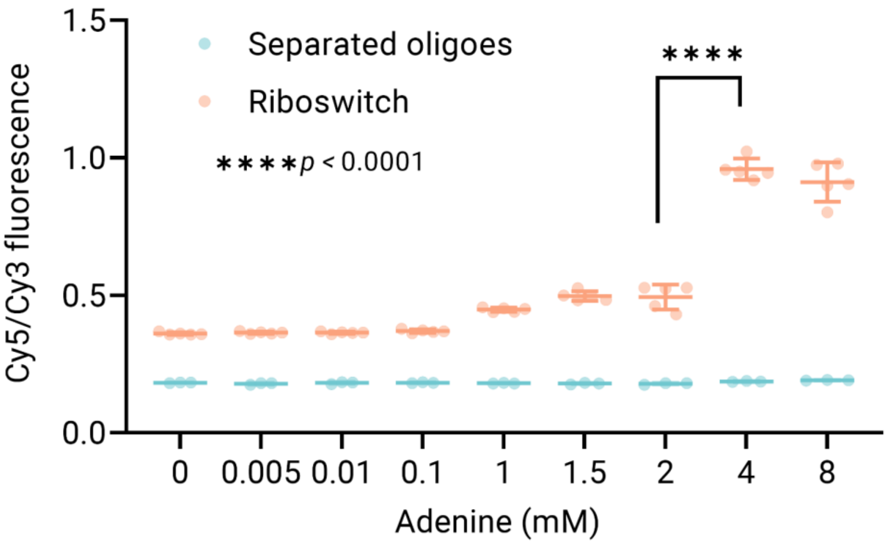

Figure S74. Normalised FRET measurement of either **Cy5-Cy3-riboswitch** or a mixture of Cy5- and Cy3-labelled DNA oligonucleotides with increasing concentration of adenine ligand (0.005, 0.01, 0.1, 1.0, 1.5, 2.0, 4.0, 8.0 mM).

2.19 Enzymatic synthesis of mRNA

2.19.1 Preparation of ssDNA template – templ_IRES

Reaction mixture (50 µL) containing Q5 reaction buffer (1X), mixture of dNTPs (200 µM), plasmid (5 ng, see sections 6.1 and 6.2), labelled forward DNA primer – **5´-(dual-Bio)-DNA-FOR-prim_29nt** (0.5 µM), reverse DNA primer – **DNA-REV-prim_26nt** (0.5 µM) and Q5 HF DNA polymerase (1 U) was incubated following the standard protocol in section 2.5.18. The reaction mixture was purified according to standard supplier´s protocol using QIAquick PCR purification kit, followed by magnetoseparation according to protocol in section 2.5.17. For generation of larger quantities of dsDNA template, the PCR reaction was 20X scaled-up, purified using the NucleoSpin gel and PCR clean-up midi kit, followed by magnetoseparation for preparation of ssDNA templates. Aliquots of either dsDNA or ssDNA samples were combined with TriTrack DNA loading dye (6X) and analysed on 1% agarose gel containing TAE (1X) and GelRed (1X) in TAE running buffer (1X) at 120 V. For agarose gel analysis see Figure S75.

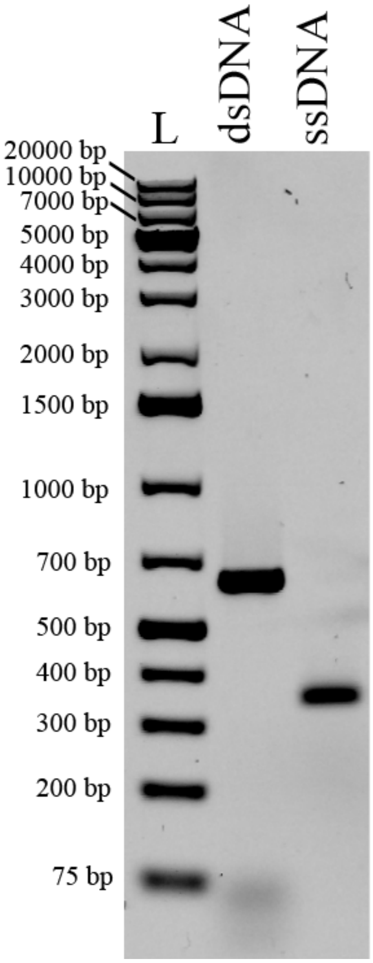

Figure S75. Native 1% agarose gel analysis. (L) dsDNA ladder; (dsDNA) aliquot of dsDNA template; (ssDNA) aliquot of ssDNA template. GelRed scan.

2.19.2 Preparation of ssDNA template – templ_IRES-prolonged

Reaction mixture (50 µL) containing Q5 reaction buffer (1X), mixture of dNTPs (200 µM), plasmid (5 ng, see sections 6.1 and 6.2), labelled forward DNA primer – **5´-(dual-Bio)-DNA-FOR-prim_29nt** (0.5 µM), reverse DNA primer – **DNA-REV-prim_24nt** (0.5 µM) and Q5 HF DNA polymerase (1 U) was incubated following the standard protocol in section 2.5.18. The reaction mixture was purified according to standard supplier´s protocol using QIAquick PCR purification kit, followed by magnetoseparation according to protocol in section 2.5.17. For generation of larger quantities of dsDNA template, the PCR reaction was 20X scaled-up, purified using the NucleoSpin gel and PCR clean-up midi kit, followed by magnetoseparation for preparation of ssDNA templates. Aliquots of either dsDNA or ssDNA samples were combined with TriTrack DNA loading dye (6X) and analysed on 1% agarose gel containing TAE (1X) and GelRed (1X) in TAE running buffer (1X) at 120 V. For agarose gel analysis see Figure S76.

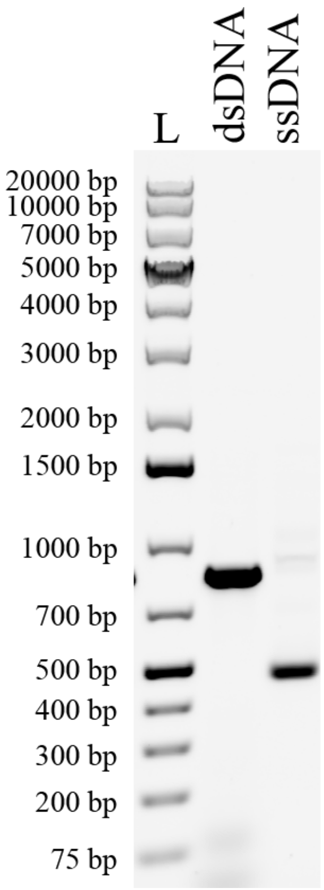

Figure S76. Native 1% agarose gel analysis. (L) GeneRuler 1 kb Plus DNA ladder; (dsDNA) aliquot of dsDNA template; (ssDNA) aliquot of ssDNA template. GelRed scan.

2.19.3 Preparation of ssDNA template – templ_IRES-nLuc

Reaction mixture (50 µL) containing Q5 reaction buffer (1X), mixture of dNTPs (200 µM), plasmid (5 ng, see sections 6.1 and 6.2), labelled forward DNA primer – **5´-(dual-Bio)-DNA-FOR-prim_29nt** (0.5 µM), reverse DNA primer – **DNA-REV-prim_48nt** (0.5 µM) and Q5 HF DNA polymerase (1 U) was incubated following the standard protocol in section 2.5.18. The reaction mixture was purified according to standard supplier´s protocol using QIAquick PCR purification kit, followed by magnetoseparation according to protocol in section 2.5.17. For generation of larger quantities of dsDNA template, the PCR reaction was 20X scaled-up, purified using the NucleoSpin gel and PCR clean-up midi kit, followed by magnetoseparation for preparation of ssDNA templates. Aliquots of either dsDNA or ssDNA samples were combined with TriTrack DNA loading dye (6X) and analysed on 1% agarose gel containing TAE (1X) and GelRed (1X) in TAE running buffer (1X) at 120 V. For agarose gel analysis see Figure S77.

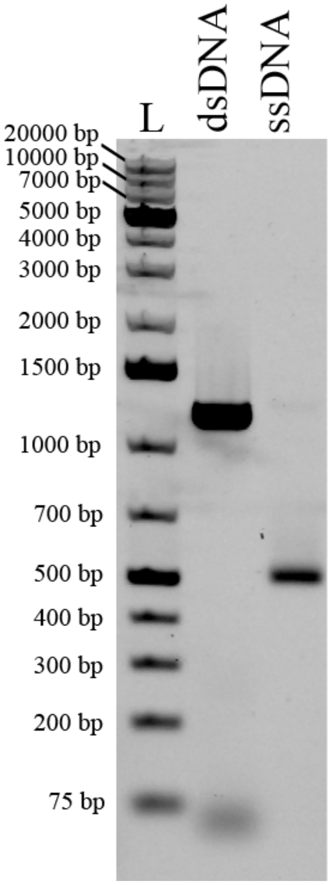

Figure S77. Native 1% agarose gel analysis. (L) GeneRuler 1 kb Plus DNA ladder; (dsDNA) aliquot of dsDNA template; (ssDNA) aliquot of ssDNA template. GelRed scan.

2.19.4 Preparation of templ_(dual-Bio)-IRES-nLuc

Reaction mixture (50 µL) containing Q5 reaction buffer (1X), mixture of dNTPs (200 µM), plasmid (5 ng, see sections 6.1 and 6.2), labelled forward DNA primer – **5´-(P)-DNA-FOR-prim_29nt** (0.5 µM), reverse DNA primer – **5´-(dual-Bio)-DNA -REV-prim_48nt** (0.5 µM) and Q5 HF DNA polymerase (1 U) was incubated following the standard protocol in section 2.5.18. 5 µL aliquot was combined with TriTrack DNA loading dye (1X) and analysed on 1% agarose gel containing TAE (1X) and GelRed (1X) in TAE running buffer (1X) at 150 V. For agarose gel analysis see Figure S78. For generation of larger quantities of dsDNA template, the PCR reaction was 48X scaled-up and purified using the NucleoSpin gel and PCR clean-up midi kit. Generation of ssDNA **templ_(dual-Bio)-IRES-nLuc** was ensured by magnetoseparation procedure. Briefly, pre-washed SMB (300 µL) were combined with purified PCR product (65 µg of dsDNA template) and binding buffer [TRIS (10 mM), EDTA (1 mM), NaCl (100 mM), TWEEN 20 (0.1%), pH 7.5] and incubated overnight at 25 °C on Hula-mixer. Then, SMB were successively washed with wash buffer [TRIS (10 mM), EDTA (1 mM), NaCl (500 mM), TWEEN 20 (0.1%), pH 7.5] and water. Strand separation was performed by addition of NaOH (50 mM), followed by neutralisation

with TRIS (1 M, pH 7.5) and HCl (1 M). SMB were successively washed with binding buffer. Quantification of **templ_(dual-Bio)-IRES-nLuc** bound to SMB was performed by Nanodrop analysis of the second complementary (eluted) ssDNA strand. The SMB with immobilised (dual-Bio)-ssDNA template were stored in binding buffer at 3 °C. The SMB were washed with ThermoPol buffer (1X) prior to use in PEX reaction (section 2.19.20 and section 2.19.21).

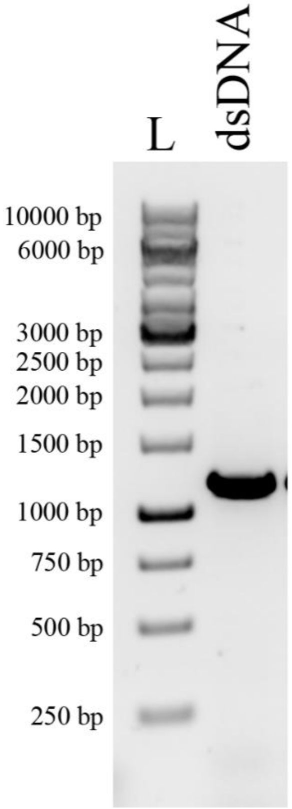

Figure S78. Native 1% agarose gel analysis. (L) GeneRuler 1 kb DNA ladder; (dsDNA) aliquot of dsDNA template. GelRed scan.

2.19.5 Preparation of IRES-RNA

Reaction was performed in total volume of 10 µL in ThermoPol buffer (1X). The ssDNA template – **templ_IRES** (0.24 µM, prepared according to section 2.19.1) and 5´-(Cy5)-labelled RNA primer – **Cy5-mRNA-prim** (0.2 µM) in presence of ThermoPol buffer were heated up to 95 °C for 30 sec and then cooled down to 3 °C (0.1 °C s^{-1}). After addition of a mixture of rNTPs (0.4 mM) and TGK polymerase (0.1 µM) the reaction was incubated at 65 °C for 4 h, followed by final work up using the standard protocol in section 2.5.19. For generation of larger quantities of **IRES-RNA**, the reaction was 10X scaled-up. For sample preparation protocol for gel analysis see section 2.5.2. For urea agarose gel analysis see Figure S79.

2.19.6 Synthesis of IRES-RNA_prolonged

Reaction was performed in total volume of 10 µL in ThermoPol buffer (1X). The ssDNA template – **templ_IRES-prolonged** (0.24 µM, prepared according to section 2.19.2) and 5´-(Cy5)-labelled RNA primer – **Cy5-mRNA-prim** (0.2 µM) in presence of ThermoPol buffer were heated up to 95 °C for 30 sec and then cooled down to 3 °C (0.1 °C s^{-1}). After addition of a mixture of rNTPs (0.4 mM) and TGK polymerase (1.0 µM) the reaction was incubated at 65 °C for 4 h, followed by final work up using the standard protocol in section 2.5.19. For generation of larger quantities of **IRES-RNA_prolonged**, the reaction was 10X scaled-up. For sample preparation protocol for gel analysis see section 2.5.2. For urea agarose gel analysis see Figure S79.

2.19.7 Synthesis of natural mRNA (mRNA-nat)

Reaction was performed in total volume of 10 µL in ThermoPol buffer (1X). The ssDNA template – **templ_IRES-nLuc** (0.24 µM, prepared according to section 2.19.3) and 5´-(Cy5)-labelled RNA primer – **Cy5-mRNA-prim** (0.2 µM) in presence of ThermoPol buffer were heated up to 95 °C for 30 sec and then cooled down to 3 °C (0.1 °C s^{-1}). After addition of a mixture of rNTPs (0.4 mM) and TGK polymerase (0.75 µM) the reaction was incubated at 65 °C for 4 h, followed by final work up using the standard protocol in section 2.5.20. For generation of larger quantities of **mRNA-nat**, the reaction was 3X scaled-up. For sample preparation protocol for gel analysis see section 2.5.2. For urea agarose gel analysis see Figure S79.

2.19.8 Synthesis of fully modified mRNA (mRNA-full)

Reaction was performed in total volume of 10 µL in ThermoPol buffer (1X). The ssDNA template – **templ_IRES-nLuc** (0.24 µM, prepared according to section 2.19.3) and 5´-(Cy5)-labelled RNA primer – **Cy5-mRNA-prim** (0.2 µM) in presence of ThermoPol buffer were heated up to 95 °C for 30 sec and then cooled down to 3 °C (0.1 °C s^{-1}). After addition of a mixture of rATP, rUTP, **rC^{Me}TP**, rGTP (0.4 mM) and TGK polymerase (0.75 µM) the reaction was incubated at 65 °C for 4 h, followed by final work up using the standard protocol in section 2.5.20. For generation of larger quantities of **mRNA-full**, the reaction was 3X scaled-up. For sample preparation protocol for gel analysis see section 2.5.2. For urea agarose gel analysis see Figure S79.

2.19.9 Synthesis of gene modified mRNA (mRNA-gene)

Reaction mixture (10 µL) containing ThermoPol buffer (1X), mixture of rATP, rUTP, **rC^{Me}TP**, rGTP (0.2 mM), ssDNA template – **templ_IRES-nLuc** (0.24 µM, prepared according to section 2.19.3), 5´-(Cy5)-labelled RNA primer – **Cy5-IRES-RNA** (0.2 µM, prepared according to section 2.19.5) and TGK polymerase (0.75 µM) was heated up to 95 °C for 30 sec followed by incubation at 65 °C for 4 h. Final work up was performed according to standard protocol in section 2.5.20. For generation of larger quantities of **mRNA-gene**, the reaction was 3X scaled-up. For sample

preparation protocol for gel analysis see section 2.5.2. For urea agarose gel analysis see Figure S79.

2.19.10Synthesis of single-site modified mRNA (mRNA-init)

Reaction mixture (10 µL) containing ThermoPol buffer (1X), **rC^{Me}TP** (0.2 mM), ssDNA template – **templ_IRES-nLuc** (0.24 µM, prepared according to section 2.19.3), 5´-(Cy5)-labelled RNA primer **– Cy5-IRES-RNA** (0.2 µM, prepared according to section 2.19.5) and TGK polymerase (0.75 µM) was heated up to 95 °C for 30 sec followed by incubation at 65 °C for 20 min. After this, the mixture was combined with rSAP (1 U) and incubated at 37 °C for 30 min, followed by enzyme denaturation at 65 °C for 5 min. Then a mixture of rNTPs (4 mM, 1 µL) was added and the reaction was further incubated at 65 °C for 4 h, followed by final work up using the standard protocol in section 2.5.20. For generation of larger quantities of **mRNA-init** the reaction was 3X scaled-up. For sample preparation protocol for gel analysis see section 2.5.2. For urea agarose gel analysis see Figure S79.

2.19.11 Synthesis of single-site modified mRNA (mRNA-mid)

Reaction mixture (10 µL) containing ThermoPol buffer (1X), **rC^{Me}TP** (0.2 mM), ssDNA template – **templ_IRES-nLuc** (0.24 µM, prepared according to section 2.19.3), 5´-(Cy5)-labelled RNA primer – **Cy5-IRES-RNA_prolonged** (0.2 µM, prepared according to section 2.19.6) and TGK polymerase (0.75 µM) was heated up to 95 °C for 30 sec followed by incubation at 65 °C for 20 min. After this, the mixture was combined with rSAP (1 U) and incubated at 37 °C for 30 min, followed by enzyme denaturation at 65 °C for 5 min. Then a mixture of rNTPs (4 mM, 1 µL) was added and the reaction was further incubated at 65 °C for 4 h, followed by final work up using the standard protocol in section 2.5.20. For generation of larger quantities of **mRNA-mid**, the reaction was 3X scaled-up. For sample preparation protocol for gel analysis see section 2.5.2. For urea agarose gel analysis see Figure S79.

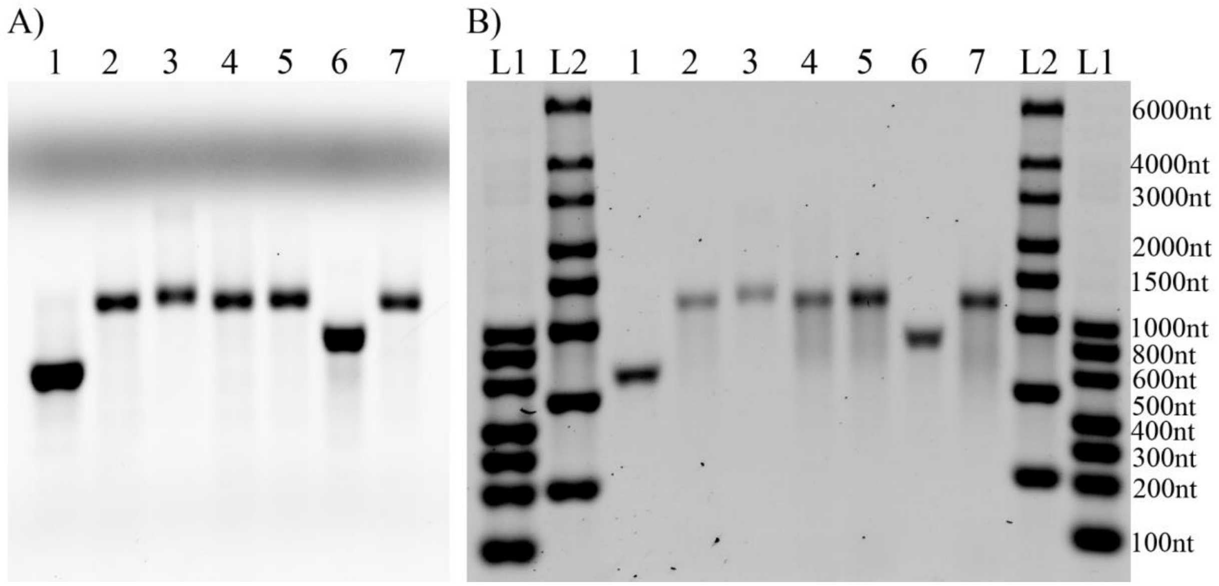

Figure S79. 1.5% urea agarose gel. (1) **IRES-RNA**; (2) **mRNA-nat**; (3) **mRNA-full**; (4) **mRNA-gene**; (5) **mRNA-init**; (6) **IRES-RNA_prolonged**; (7) **mRNA-mid**. (L1) RiboRuler Low Range RNA ladder; (L2) RiboRuler High Range RNA ladder. A) Cy5 scan; B) GelRed scan.

2.19.12 Bisulfite conversion of mRNA with single modification

Approximately 20 ng of single-site modified mRNA (prepared according to sections 2.19.10 and 2.19.11) were treated with EZ RNA methylation kit according to supplier´s protocol, followed by elution with 15 µL of water. Converted mRNA was directly used in RT-PCR reaction in section 2.19.13.

2.19.13 One step RT-PCR reaction of bisulfite-treated mRNA

Reaction mixture (12.5 µL) containing bisulfite-converted mRNA (1 µL, prepared according to section 2.19.12), forward DNA primer – **DNA-FOR-prim_26nt** (0.5 µM), reverse DNA primer – **DNA-REV-prim_21nt** (0.5 µM), LunaScript Multiplex one-step RT-PCR reaction mix (1X) and LunaScript Multiplex one-step RT-PCR enzyme mix (1X) was incubated according to standard protocol in section 2.5.21. Crude reaction mix was combined with TriTrack DNA loading dye (1X) and analysed on 1% agarose gel containing TAE (1X) and SybrSafe (1X) in TAE running buffer (1X) at 120 V. For generation of larger quantities of the desired product, the reaction was 10X scaled-up. The desired PCR products were visualised by UV shadowing, excised, and purified according to standard supplier´s protocol using QIAquick gel extraction kit, followed by elution with H_2O.

2.19.14 Sample preparation for Sanger sequencing

Mixture (10 µL) containing 45 ng of sample prepared according to section 2.19.13 (from **mRNA-init**) and DNA primer – **DNA-REV-prim_21nt** (5 pmol) was subjected to Sanger sequencing analysis. For raw data see section 5.1 and Figure S263. Mixture (10 µL) containing 100 ng of sample prepared according to section 2.19.13 (from **mRNA-mid**) and DNA primer – **DNA-REV-prim_21nt** (25 pmol) was subjected to Sanger sequencing analysis. For raw data see section 5.1 and Figure S264.

2.19.15 PEX with natural rNTPs or a mixture of rA^{E}TP, rU^{E}TP, rC^{Me}TP, rG^{Pent}TP and templ_IRES, templ_IRES-prolonged, templ_IRES-nLuc using TGK polymerase

Reaction was performed in total volume of 10 µL in ThermoPol buffer (1X). The ssDNA template – **templ_IRES**, **templ_IRES-prolonged** or **templ_IRES-nLuc** (0.24 µM, prepared according to sections 2.19.1, 2.19.2, 2.19.3) and 5´-(Cy5)-labelled RNA primer – **Cy5-mRNA-prim** (0.2 µM) in presence of ThermoPol buffer were heated up to 95 °C for 30 sec and then cooled down to 3 °C (0.1 °C s^{-1}). After addition of TGK polymerase (1.0 µM) and a mixture of **rA^{E}TP**, **rU^{E}TP**, **rC^{Me}TP**, **rG^{Pent}TP** (0.4 mM) the reaction was incubated at 50 °C for 30 min and then at 65 °C for 6 h, followed by final work up with Turbo DNase and proteinase K using the standard protocol in section 2.5.20. Positive control was performed under same conditions with a mixture of natural rNTPs (0.4 mM) instead of modified **rN^{X}TP**s. 5 µL aliquot of the crude mixture was combined with 5 µL of 2X stop solution (95% [v/v] formamide, 0.5 mM EDTA, 0.025% [w/v] bromophenol blue, 0.025% [w/v] SDS in H_2O) and loaded on gel. 5 µL of RNA ladder (RiboRuler High Range), prepared according to manufacturer´s procedure was loaded on gel. All samples were analysed on 1% agarose gel containing 2 M urea, TAE buffer (1X) and GelRed (1X) in TAE running buffer (1X) with addition of 2 M urea at 100 V at room temperature. The gel was afterwards visualised by a fluorescent scanner. For urea agarose gel analysis see Figure S80. For generation of larger quantities of fully modified **IRES-RNA_A^{E}U^{E}C^{Me}G^{Pent}**, **IRES-RNA_prolonged_A^{E}U^{E}C^{Me}G^{Pent}** and **mRNA_A^{E}U^{E}C^{Me}G^{Pent}** the reactions were 3X scaled-up and purified with QIAquick nucleotide removal kit according to standard supplier´s protocol.

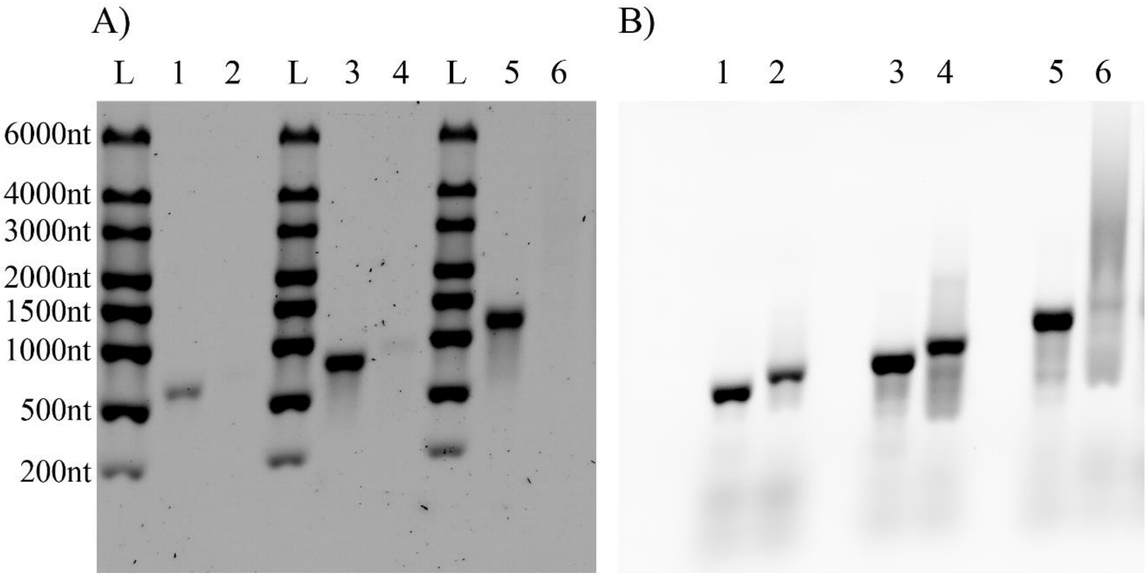

Figure S80. 1% urea agarose gel. (1) PEX reaction with **templ_IRES** and a mixture of rATP, rUTP, rCTP, rGTP; (2) PEX reaction with **templ_IRES** and a mixture of **rA^{E}TP**, **rU^{E}TP**, **rC^{Me}TP**, **rG^{Pent}TP**; (3) PEX reaction with **templ_IRES-prolonged** and a mixture of rATP, rUTP, rCTP, rGTP; (4) PEX reaction with **templ_IRES-prolonged** and a mixture of **rA^{E}TP**, **rU^{E}TP**, **rC^{Me}TP**, **rG^{Pent}TP**; (5) PEX reaction with **templ_IRES-nLuc** and a mixture of rATP, rUTP, rCTP, rGTP; (6) PEX reaction with **templ_IRES-nLuc** and a mixture of **rA^{E}TP**, **rU^{E}TP**, **rC^{Me}TP**, **rG^{Pent}TP**; (L) RiboRuler High Range RNA ladder. A) GelRed scan; B) Cy5 scan.

2.19.16 Reverse transcription of natural IRES-RNA and fully modified IRES-RNA_A^{E}U^{E}C^{Me}G^{Pent}

Reaction mixture containing 4 µL of purified either natural **IRES-RNA** or fully modified **IRES-RNA_A^{E}U^{E}C^{Me}G^{Pent}** (prepared according to section 2.19.15), 0.5 µL of dNTPs (10 mM), 0.5 µL of **DNA-REV-prim_26nt** (10 µM) and 1.5 µL of DEPC H_2O was heated at 65 °C for 5 min followed by cooling to 4 °C in a thermal cycler with heated lid. Then 4 µL of SSIV reaction buffer (5X), 0.5 µL of DTT (100 mM), 0.5 µL of Protector RNase inhibitor (40 U/µL) and 0.5 µL of SSIV RT (200 U/µL) were added. The mixture was incubated at 55 °C for 14 h, followed by cooling to 4 °C. Control reactions were performed under same conditions with replacement of either RNA template or SSIV RT by DEPC H_2O. The generated cDNA product was purified by Zymo DNA Clean & Concentrator kit according to standard supplier´s protocol and amplified by qPCR (section 2.19.18).

2.19.17 Reverse transcription of fully modified IRES-RNA_prolonged_A^{E}U^{E}C^{Me}G^{Pent} and mRNA_A^{E}U^{E}C^{Me}G^{Pent}

Reaction mixture containing 0.5 µL of purified fully modified either **IRES-RNA_prolonged_A^{E}U^{E}C^{Me}G^{Pent}** or **mRNA_A^{E}U^{E}C^{Me}G^{Pent}** (prepared according to section

2.19.15), 0.5 µL of dNTPs (10 mM), 0.5 µL of **DNA-REV-prim_24nt** (10 µM) for **IRES-RNA_prolonged_A^{E}U^{E}C^{Me}G^{Pent}** or **DNA-REV-prim_48nt** (10 µM) for **mRNA_A^{E}U^{E}C^{Me}G^{Pent}** and 1.5 µL of DEPC H_2O was heated at 95 °C for 1 min followed by cooling to 4 °C in a thermal cycler with heated lid. Then 4 µL of SSIV reaction buffer (5X), 0.5 µL of DTT (100 mM), 0.5 µL of Protector RNase inhibitor (40 U/µL) and 0.5 µL of SSIV RT (200 U/µL) were added. The mixture was incubated at 55 °C for 14 h, followed by cooling to 4 °C. Control reactions were performed under same conditions with replacement of either RNA template or or SSIV RT by DEPC H_2O. The generated cDNA product was purified by Zymo DNA Clean & Concentrator kit according to standard supplier´s protocol and amplified by qPCR (section 2.19.19).

2.19.18 qPCR amplification of cDNA from IRES-RNA or IRES-RNA_A^{E}U^{E}C^{Me}G^{Pent} and gel analysis

Reaction mixture containing 3 µL of purified cDNA product (prepared according to section 2.19.16), 2 µL of Q5 reaction buffer (5X), 0.1 µL of Q5 HF DNA polymerase (2 U/µL), 0.5 µL **DNA-REV-prim_26nt** (10 µM), 0.5 µL **5´-(dual-Bio)-DNA-FOR-prim_29nt** (10 µM), 1 µL of SybrGreen (10X), 1 µL of dNTPs (2 mM) and 1.9 µL of H_2O was incubated in a real time PCR cycler (BioRad C1000) as follows: denaturation at 98 °C for 15 sec followed by 15 cycles of denaturation at 98 °C for 10 sec, annealing at 54 °C for 30 sec and extension at 72 °C for 45 sec followed by cooling to 4 °C. Control reactions were performed either without RNA template or SSIV RT or by adding water to PCR reaction. The crude mixtures were combined with 2 µL of Gel loading dye, purple (6X) and 4 µL aliquots were analysed on 1% agarose gel containing TAE (1X) and GelRed (1X) in TAE running buffer (1X) at 150 V. For agarose gel analysis see Figure S81 and for qPCR curves see Figure S82.

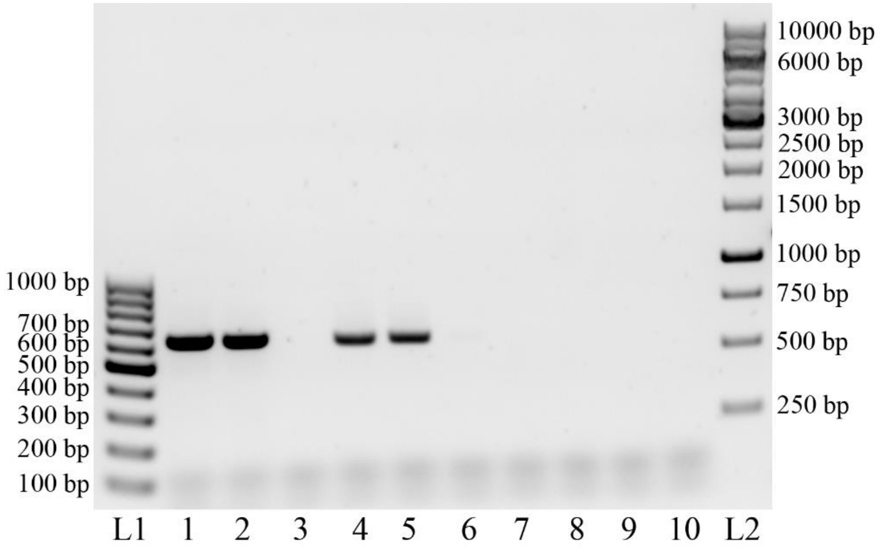

Figure S81. Native 1% agarose gel analysis. (1) amplification of cDNA from natural **IRES-RNA**, 1st replicate; (2) amplification of cDNA from natural **IRES-RNA**, 2nd replicate; (3) control reaction with natural **IRES-RNA** without SSIV RT; (4) amplification of cDNA from modified **IRES-RNA_A^{E}U^{E}C^{Me}G^{Pent}**, 1st replicate; (5) amplification of cDNA from modified **IRES-RNA_A^{E}U^{E}C^{Me}G^{Pent}**, 2nd replicate; (6) control reaction with modified **IRES-RNA_A^{E}U^{E}C^{Me}G^{Pent}** without SSIV RT; (7) control reaction without RNA template; (8) control reaction without RNA template; (9) control reaction without RNA template and SSIV RT; (10) control without RNA template and reverse transcription; (L1) GeneRuler 100 bp DNA ladder; (L2) GeneRuler 1 kb DNA ladder. GelRed scan.

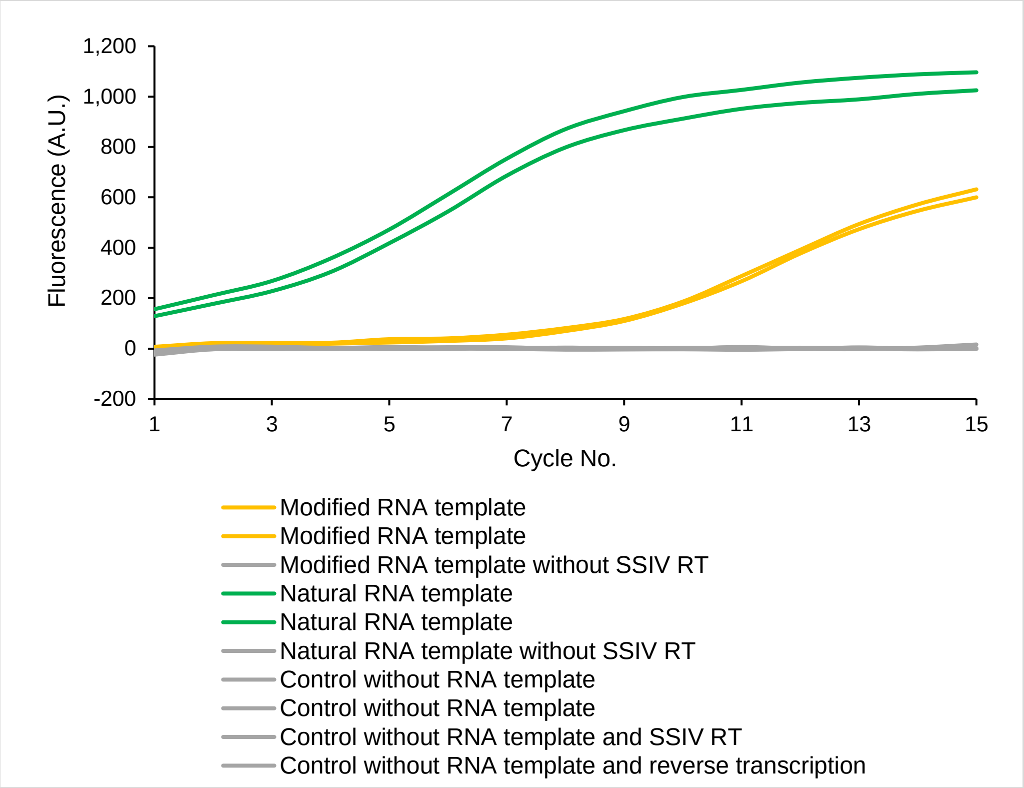

Figure S82. Quantitative PCR curves representing SybrGreen fluorescent intensity as a function of cycle number (for natural RNA template, **IRES-RNA** or for modified RNA template, **IRES-RNA_A^{E}U^{E}C^{Me}G^{Pent}**).

2.19.19 qPCR amplification of cDNA from IRES-RNA_prolonged_A^{E}U^{E}C^{Me}G^{Pent} or mRNA_A^{E}U^{E}C^{Me}G^{Pent} and gel analysis

Reaction mixture containing 5 µL of purified cDNA product (prepared according to section 2.19.17), 2 µL of Q5 reaction buffer (5X), 0.1 µL of Q5 HF DNA polymerase (2 U/µL), 0.5 µL of **DNA-REV-prim_24nt** (10 µM) for **IRES-RNA_prolonged_A^{E}U^{E}C^{Me}G^{Pent}** or **DNA-REV-prim_48nt** (10 µM) for **mRNA_A^{E}U^{E}C^{Me}G^{Pent}**, 0.5 µL **5´-(dual-Bio)-DNA-FOR-prim_29nt** (10 µM), 1 µL of SybrGreen (10X), 1 µL of dNTPs (2 mM) and 1.9 µL of H_2O was incubated in a real time PCR cycler (BioRad C1000) as follows: denaturation at 98 °C for 15 sec followed by 16 cycles of denaturation at 98 °C for 10 sec, annealing at 54 °C for 30 sec and extension at 72 °C for 45 sec followed by cooling to 4 °C (for cDNA from **IRES-RNA_prolonged_A^{E}U^{E}C^{Me}G^{Pent}**) or denaturation at 98 °C for 15 sec followed by 19 cycles of denaturation at 98 °C for 10 sec, annealing at 54 °C for 30 sec and extension at 72 °C for 90 sec followed by cooling to 4 °C (for cDNA from **mRNA_A^{E}U^{E}C^{Me}G^{Pent}**). Control reactions were performed either without RNA template or SSIV RT or by adding water to PCR reaction. The crude mixtures were combined with 2 µL of Gel loading dye, purple (6X) and 12 µL aliquots were analysed on 1% agarose gel containing TAE (1X) and GelRed (1X) in TAE running buffer (1X) at 150 V. For agarose gel analysis see Figure S83 and for qPCR curves see Figure S84 (from **IRES-**

RNA_prolonged_A^{E}U^{E}C^{Me}G^{Pent} template) and Figure S85 (from **mRNA_A^{E}U^{E}C^{Me}G^{Pent}** template).

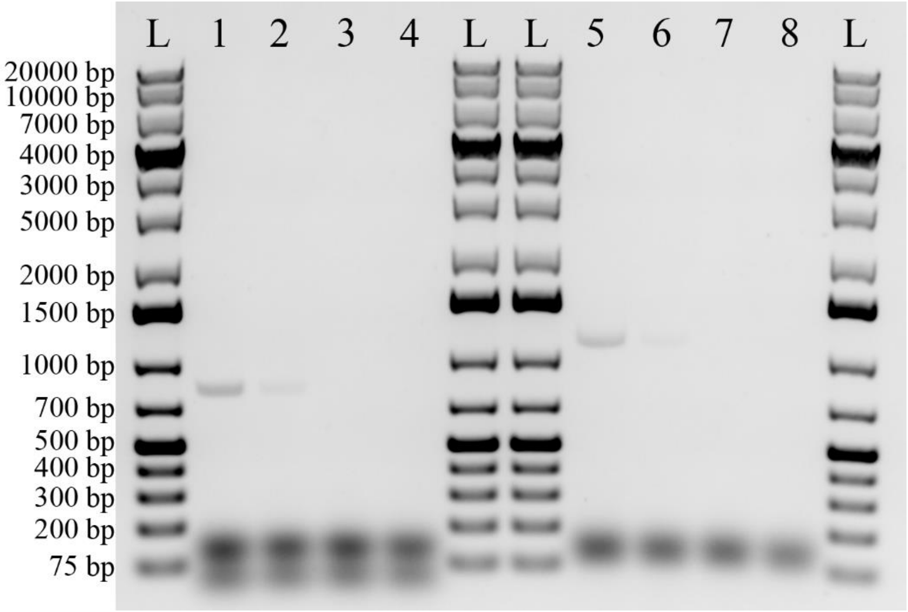

Figure S83. Native 1% agarose gel analysis. (1) amplification of cDNA from modified **IRES-RNA_prolonged_A^{E}U^{E}C^{Me}G^{Pent}**; (2) control reaction with modified **IRES-RNA_prolonged_A^{E}U^{E}C^{Me}G^{Pent}** without SSIV RT; (3) control reaction without RNA template and SSIV RT; (4) control without RNA template and reverse transcription; (5) amplification of cDNA from modified **mRNA_A^{E}U^{E}C^{Me}G^{Pent}**; (6) control reaction with modified **mRNA_A^{E}U^{E}C^{Me}G^{Pent}** without SSIV RT; (7) control reaction without RNA template and SSIV RT; (8) control without RNA template and reverse transcription; (L) GeneRuler 1 kb Plus DNA ladder. GelRed scan.

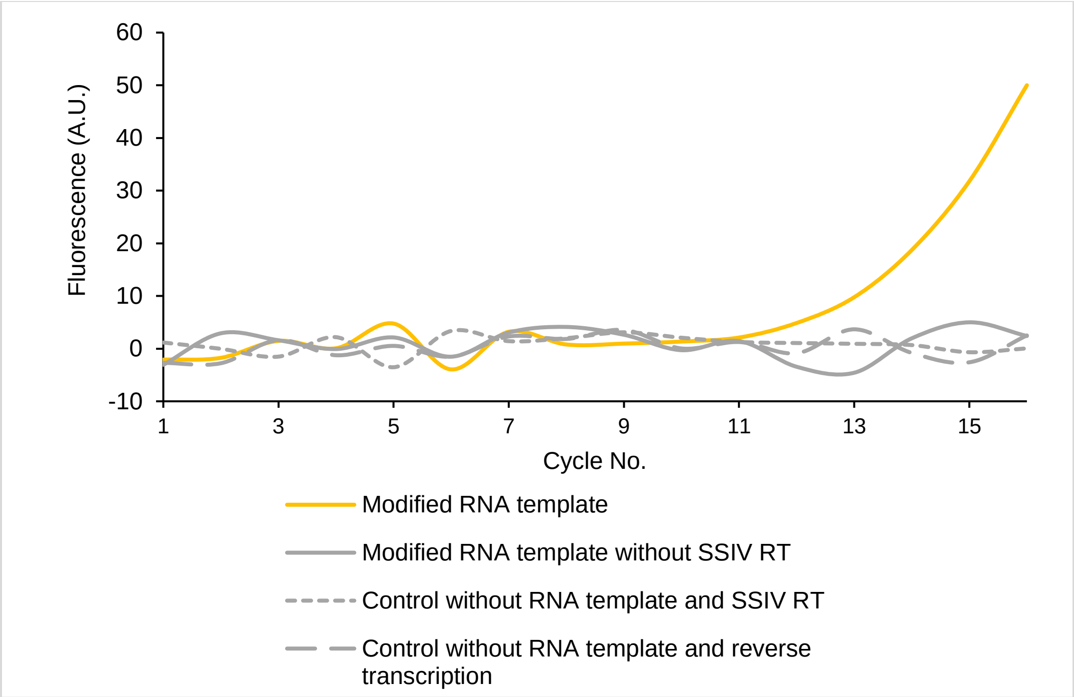

Figure S84. Quantitative PCR curves representing SybrGreen fluorescent intensity as a function of cycle number (for modified RNA template, **IRES-RNA_prolonged_A^{E}U^{E}C^{Me}G^{Pent}**).

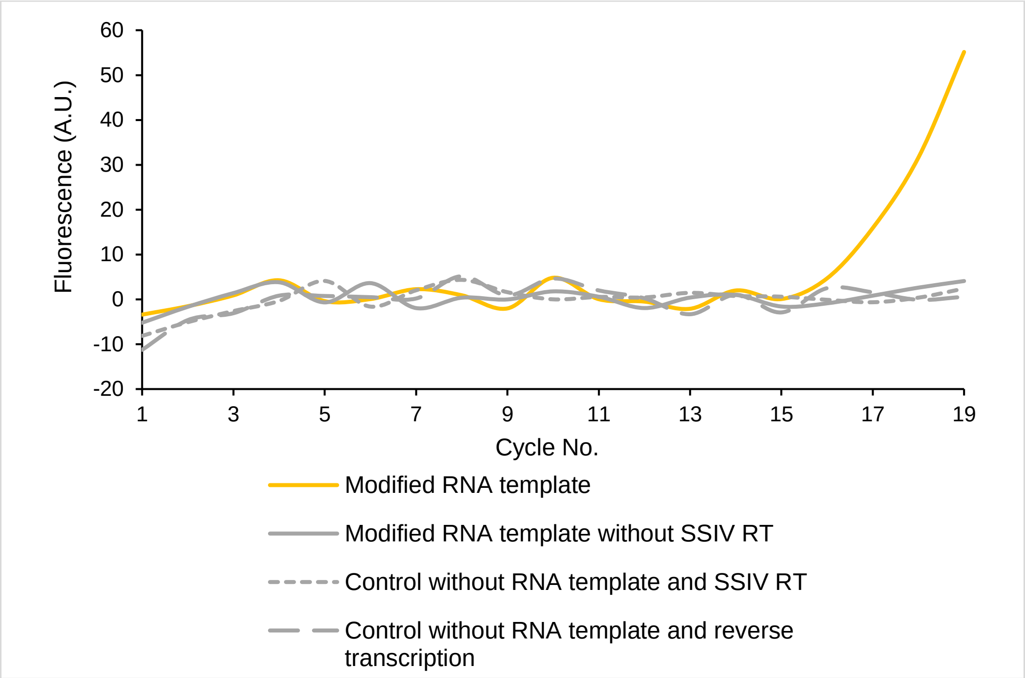

Figure S85. Quantitative PCR curves representing SybrGreen fluorescent intensity as a function of cycle number (for modified RNA template, **mRNA_A^{E}U^{E}C^{Me}G^{Pent}**).

2.19.20 PEX reaction with natural rNTPs and templ_(dual-Bio)-IRES-nLuc immobilised on streptavidin magnetic beads (SMB)

Reaction mixture (50 µL) containing 5 µL of ThermoPol buffer (10X), 1 µL of TGK polymerase (50 µM), 1 µL of **Cy5-mRNA-prim** (10 µM), 1 µL of rNTPs (20 mM), 5.5 pmol of **templ_(dual-Bio)-IRES-nLuc** immobilised on SMB (prepared according to section 2.19.4) and 42 µL of DEPC H_2O was incubated at 65 °C for 35 min in a thermal shaker at 800 rpm. After this time, the solution was removed, SMB were successively washed with wash buffer [TRIS (10 mM), EDTA (1 mM), NaCl (500 mM), TWEEN 20 (0.1%), pH 7.5] and water. Strand separation and elution of RNA product (**mRNA-nat**) was performed by addition of NaOH (50 mM), followed by neutralisation with TRIS (1 M, pH 7.5) and HCl (1 M). The SMB were washed with binding buffer [TRIS (10 mM), EDTA (1 mM), NaCl (100 mM), TWEEN 20 (0.1%), pH 7.5] and ThermoPol buffer (1X) prior to next cycle of PEX reaction performed under same conditions. Aliquots (9.1 µL) of RNA product were mixed with 2X stop solution (95% [v/v] formamide, 0.5 mM EDTA, 0.025% [w/v] bromophenol blue, 0.025% [w/v] SDS in H_2O) and loaded on gel. 5 µL of RNA ladder (RiboRuler High Range), prepared according to manufacturer´s procedure were loaded on gel. All samples were analysed on 1% agarose gel containing 2 M urea, TAE buffer (1X) and GelRed (1X) in TAE running buffer (1X) with addition of 2 M urea at 100 V at room temperature. The gel was afterwards visualised by a fluorescent scanner. For agarose gel analysis see Figure S86. Yields (in %) of each reaction were quantified relatively to first cycle of PEX reaction by ImageJ.

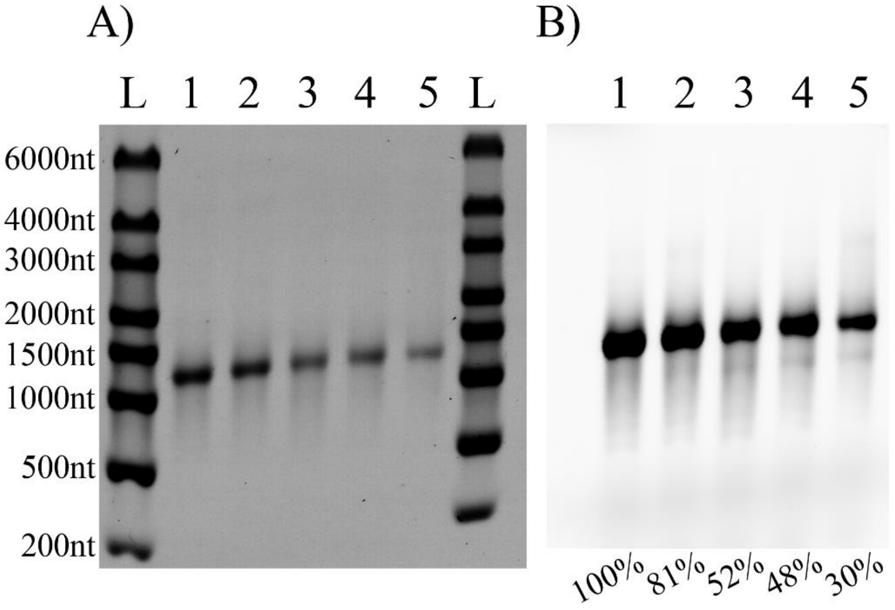

Figure S86. 1% urea agarose gel. (1) PEX reaction no. 1; (2) PEX reaction no. 2; (3) PEX reaction no. 3; (4) PEX reaction no. 4; (5) PEX reaction no. 5; (L) RiboRuler High Range RNA ladder. A) GelRed scan; B) Cy5 scan.

2.19.21 PEX reaction with a mixture of rATP, rUTP, $rC^{Me}TP$, rGTP and templ_(dual-Bio)-IRES-nLuc immobilised on streptavidin magnetic beads (SMB)

Reaction mixture (50 µL) containing 5 µL of ThermoPol buffer (10X), 2 µL of TGK polymerase (50 µM), 1 µL of **Cy5-mRNA-prim** (10 µM), 2 µL of rATP, rUTP, **$rC^{Me}TP$**, rGTP mixture (20 mM), 5.5 pmol of **templ_(dual-Bio)-IRES-nLuc** immobilised on SMB (prepared according to section 2.19.4) and 40 µL of DEPC H_2O was incubated at 65 °C for 1h in a thermal shaker at 800 rpm. After this time, the solution was removed, SMB were successively washed with wash buffer [TRIS (10 mM), EDTA (1 mM), NaCl (500 mM), TWEEN 20 (0.1%), pH 7.5] and water. Strand separation and elution of RNA product (**mRNA-full**) was performed by addition of NaOH (50 mM), followed by neutralisation with TRIS (1 M, pH 7.5) and HCl (1 M). The SMB were washed with binding buffer [TRIS (10 mM), EDTA (1 mM), NaCl (100 mM), TWEEN 20 (0.1%), pH 7.5] and ThermoPol buffer (1X) prior to next cycle of PEX reaction performed under same conditions. Aliquots (9.1 µL) of RNA product were mixed with 2X stop solution (95% [v/v] formamide, 0.5 mM EDTA, 0.025% [w/v] bromophenol blue, 0.025% [w/v] SDS in H_2O) and loaded on gel. 5 µL of RNA ladder (RiboRuler High Range), prepared according to manufacturer´s procedure were loaded on gel. All samples were analysed on 1% agarose gel containing 2 M urea, TAE buffer (1X) and GelRed (1X) in TAE running buffer (1X) with addition of 2 M urea at 100 V at room temperature. The gel was afterwards visualised by a fluorescent scanner. For agarose gel analysis see Figure S87. Yields (in %) of each reaction were quantified relatively to first cycle of PEX reaction by ImageJ.

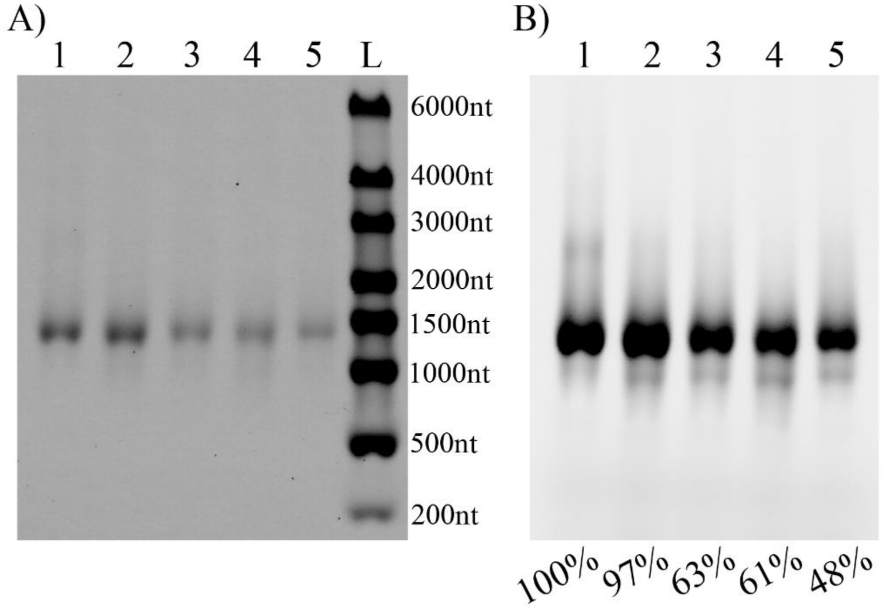

Figure S87. 1% urea agarose gel. (1) PEX reaction no. 1; (2) PEX reaction no. 2; (3) PEX reaction no. 3; (4) PEX reaction no. 4; (5) PEX reaction no. 5; (L) RiboRuler High Range RNA ladder. A) GelRed scan; B) Cy5 scan.

2.20 mRNA translation studies

2.20.1 Protocol for *in vitro* translation in rabbit reticulocyte lysate system

Reaction mixture (10 µL) containing 50 ng of mRNA, Ribolock RNase inhibitor (40 U), complete amino acids mixture (50 µM) and nuclease-treated rabbit reticulocyte lysate was incubated at 30 °C for 1.5 h in a thermal cycler with heated lid (65 °C). Translation reactions were stopped by addition of cycloheximide solution (100 µM, in DMSO). 1 µL aliquot of the quenched reaction mixture was combined with 20 µL of reconstituted Nano-Glo luciferase assay reagent prepared by combining Nano-Glo luciferase assay substrate and Nano-Glo luciferase assay buffer (1:50) according to manufacturer´s protocol. The mixture was incubated at room temperature for 3 min and 20 µL aliquots were transferred to white opaque 384-well microplate prior to luminescence measurements with 1000 ms integration time on Tecan Spark Multimode Reader. The data are reported as counts/sec. For negative control, mRNA was omitted.

2.20.2 Protocol for *in cellulo* translation in HEK293T cell system

3×10^4 HEK293T cells were seeded in 96-well plate (black wells, clear bottom) 18 h prior to transfection in 40 µL Dulbecco's Modified Eagle Medium containing 10% fetal bovine serum. Lipofectamine MessengerMAX (0.125 µL) was diluted in 5 µL OptiMEM medium. At the same time mRNA (25 ng) was mixed with 5 µL OptiMEM and incubated for 10 min. Both mixes were combined and incubated for 5 min at ambient temperature. Afterwards, the combined transfection mix was added to cells. After 4 h at 37 °C, 5% CO_2 in humidified incubator, cells were equilibrated at 25 °C for 10 min. Cells were combined with 50 uL of reconstituted Nano-Glo luciferase assay reagent prepared by combining Nano-Glo luciferase assay substrate and Nano-Glo luciferase assay buffer (1:50) according to manufacturer´s protocol. Then, the plate was mixed for 3 min at 1 mm circular diameter on Tecan Spark Multimode Reader, followed by luminescence measurement with 1000 ms integration time. The data are reported as counts/sec. For negative controls, mRNA and/or Lipofectamine was omitted.

2.21 Amplification PEX reaction with either all four natural rNTPs or a mixture rATP, rUTP, rC^{Me}TP, rGTP using a 98-nt long ssDNA template

Reactions were performed in total volume of 10 µL in ThermoPol buffer (1X) containing ssDNA template – **templ_98nt** (0.01 µM), 5´-(Cy5)-labelled RNA primer – **Cy5-RNA-prim_15nt** (1.0 µM), TGK polymerase (1.0 µM) and either a mixture of rATP, rUTP, rCTP, rGTP (0.4 mM) or a mixture of rATP, rUTP, **rC^{Me}TP**, rGTP (0.4 mM). Amplification PEX reactions were performed in a thermal cycler with heated lid as follows: 41 cycles of denaturation at 95 °C for 10 sec, extension at 50 °C for 1 min followed by extension at 65 °C for 3 min. Control single PEX reactions were prepared under same conditions performed in a thermal cycler with heated lid as follows: denaturation at 95 °C for 10 sec, extension at 50 °C for 1 min followed by extension at 65 °C for 3 min. The reaction mixtures were combined with Turbo DNase (2 U) and incubated at

37 °C for 30 min followed by incubation with proteinase K (0.8 U) at 37 °C for additional 30 min. Then 5 µL of the crude reaction mixture were combined with 5 µL of 2X stop solution (95% [v/v] formamide, 0.5 mM EDTA, 0.025% [w/v] bromophenol blue, 0.025% [w/v] SDS in H_2O), denatured by heating at 95 °C for 2 min and then immediately cooled on ice. Aliquots of the denatured samples (10 µL) were subjected to vertical gel electrophoresis. For dPAGE analysis see Figure S88.

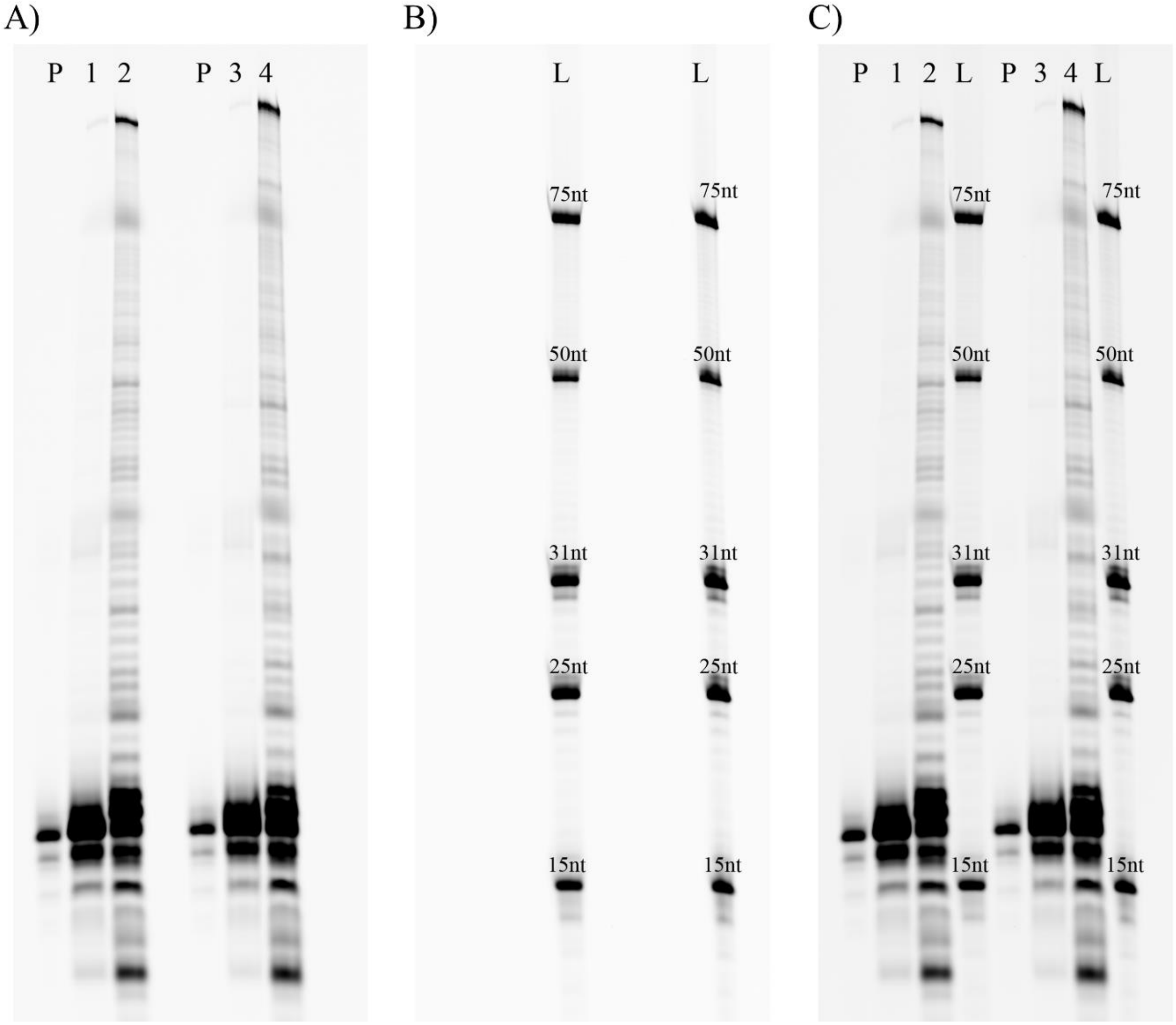

Figure S88. 12.5% dPAGE analysis. (P) RNA primer; (1) Single PEX reaction with a mixture of rATP, rUTP, rCTP, rGTP; (2) Amplified, 41 cycles of PEX reaction with a mixture of rATP, rUTP, rCTP, rGTP; (3) Single PEX reaction with a mixture of rATP, rUTP, **rC^{Me}TP**, rGTP; (4) Amplified, 41 cycles of PEX reaction with a mixture of rATP, rUTP, **rC^{Me}TP**, rGTP. (L) RNA ladder composed of FAM-labelled RNA oligonucleotides of indicated length. A) Cy5 scan, B) FAM scan, C) merged Cy5 and FAM scans.

3 Copies of NMR spectra

3.1 NMR spectra of nucleosides

3.1.1 Spectra of 7-(pent-1-yn-1-yl)-7-deazaadenosine (rA^{Pent})

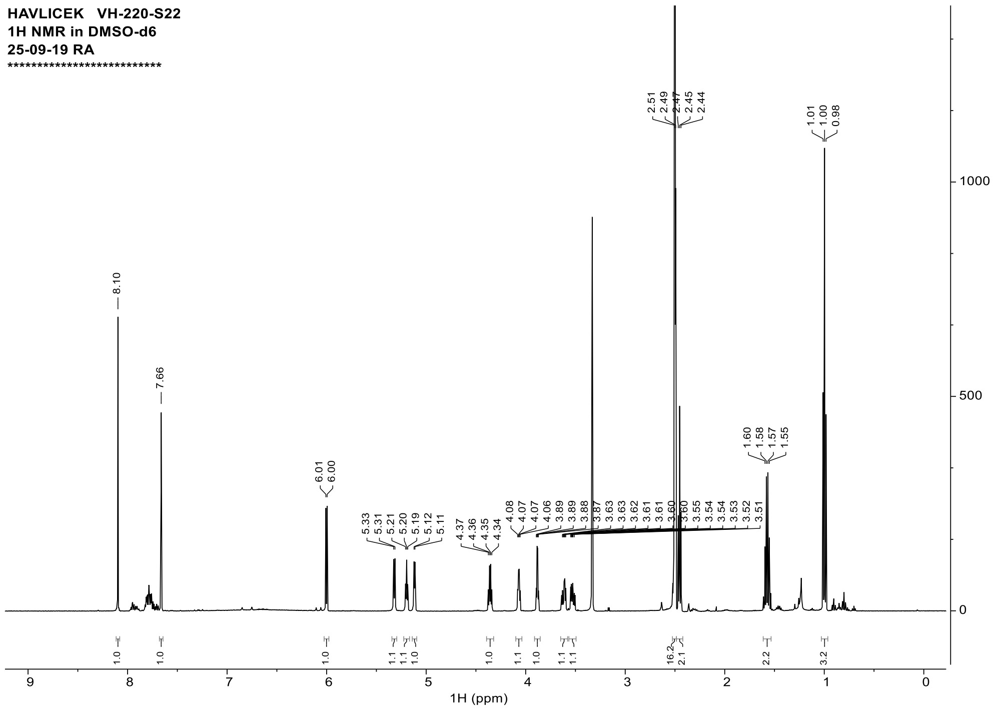

Figure S89. ^{1}H NMR spectrum.

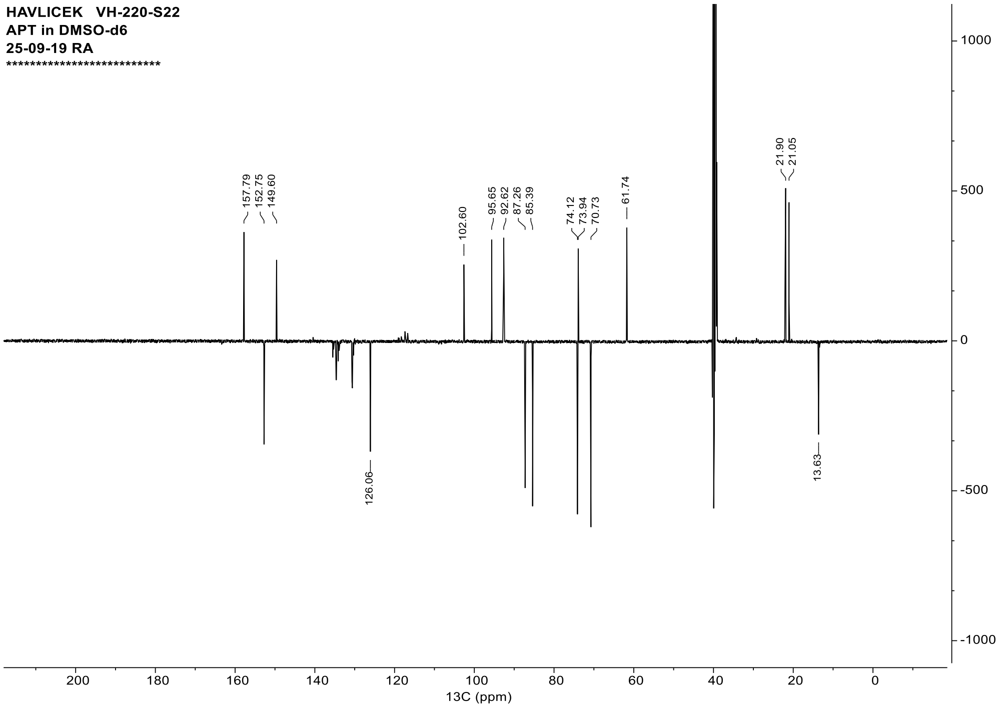

Figure S90. ^{13}C NMR spectrum.

3.1.2 Spectra of 5-{3-[4-(5,5-difluoro-1,3,7,9-tetramethyl-5*H*-4λ[4],5λ[4]-dipyrrolo[1,2-c:2',1'-f][1,3,2]diazaborinin-10-yl)-3,5-dimethylphenoxy]prop-1-yn-1-yl}-cytidine (rC^{mBdp})

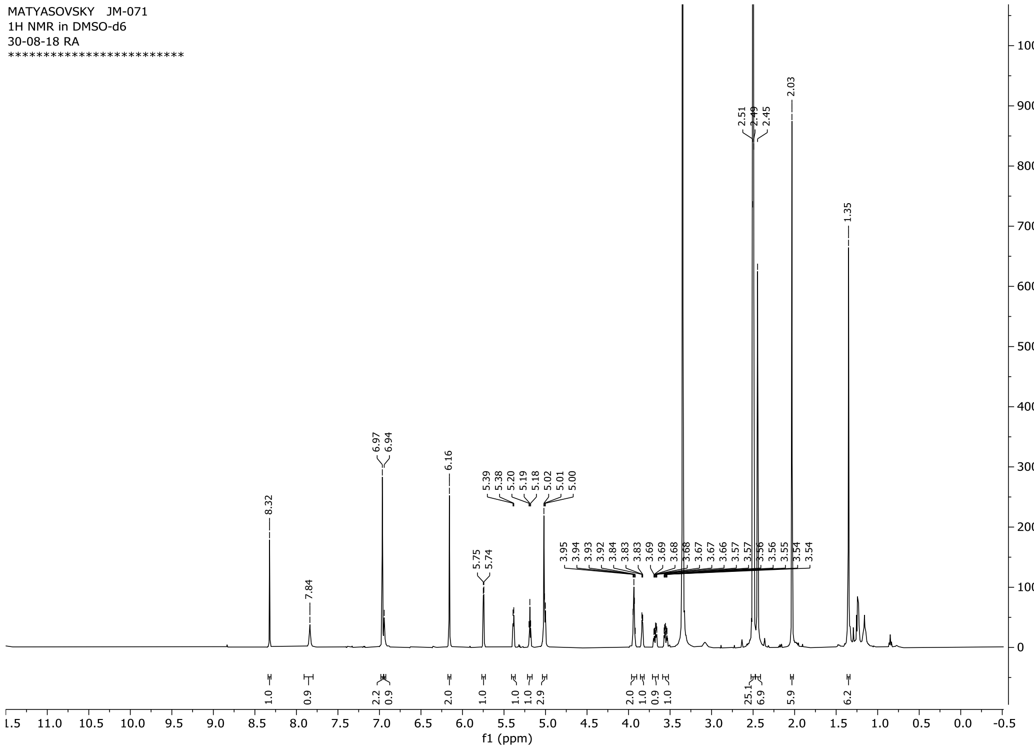

Figure S91. ^{1}H NMR spectrum.

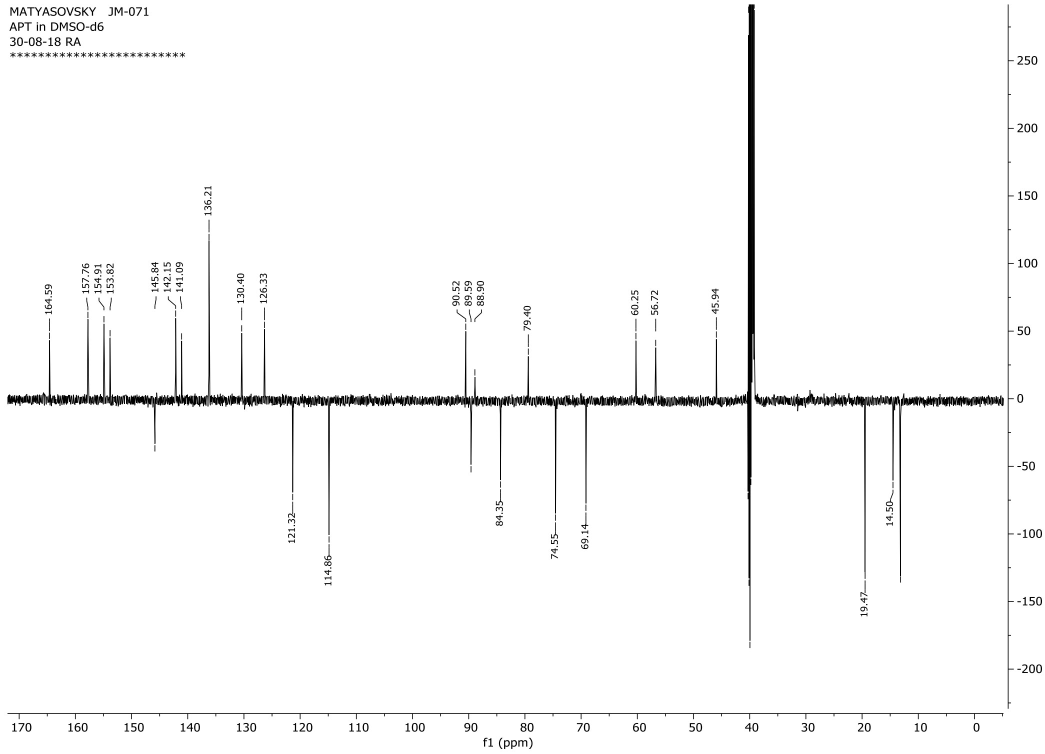

Figure S92. ^{13}C NMR spectrum.

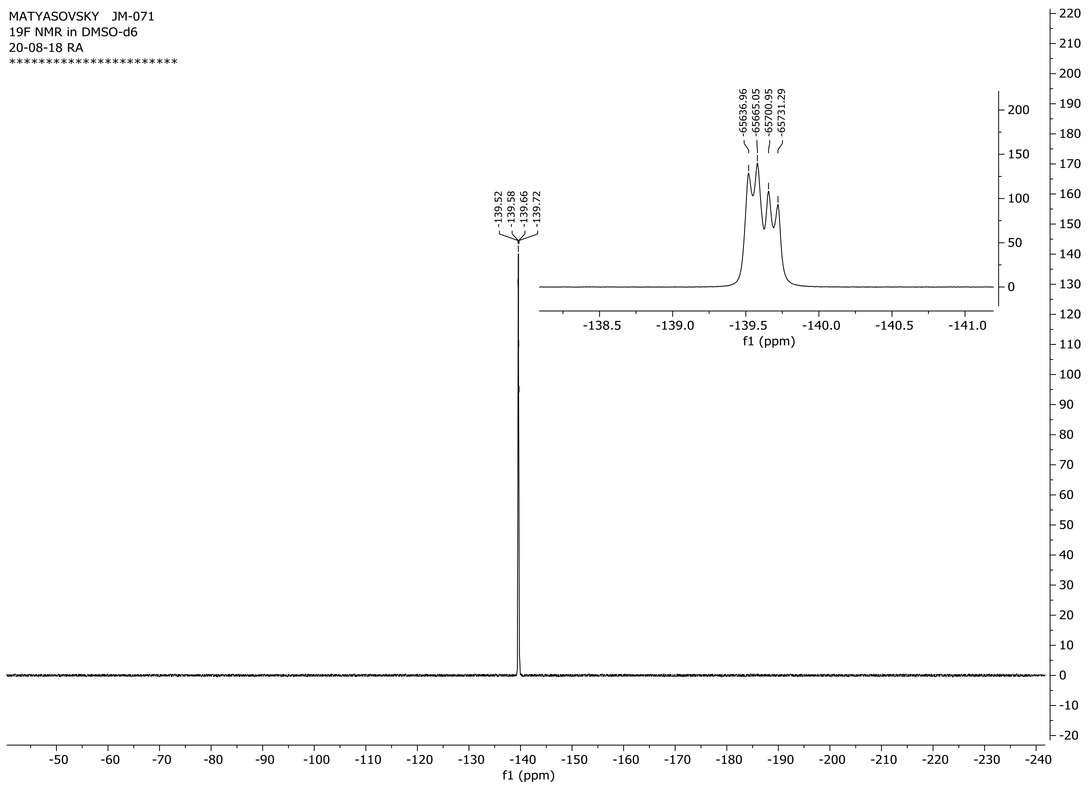

Figure S93. ^{19}F NMR spectrum.

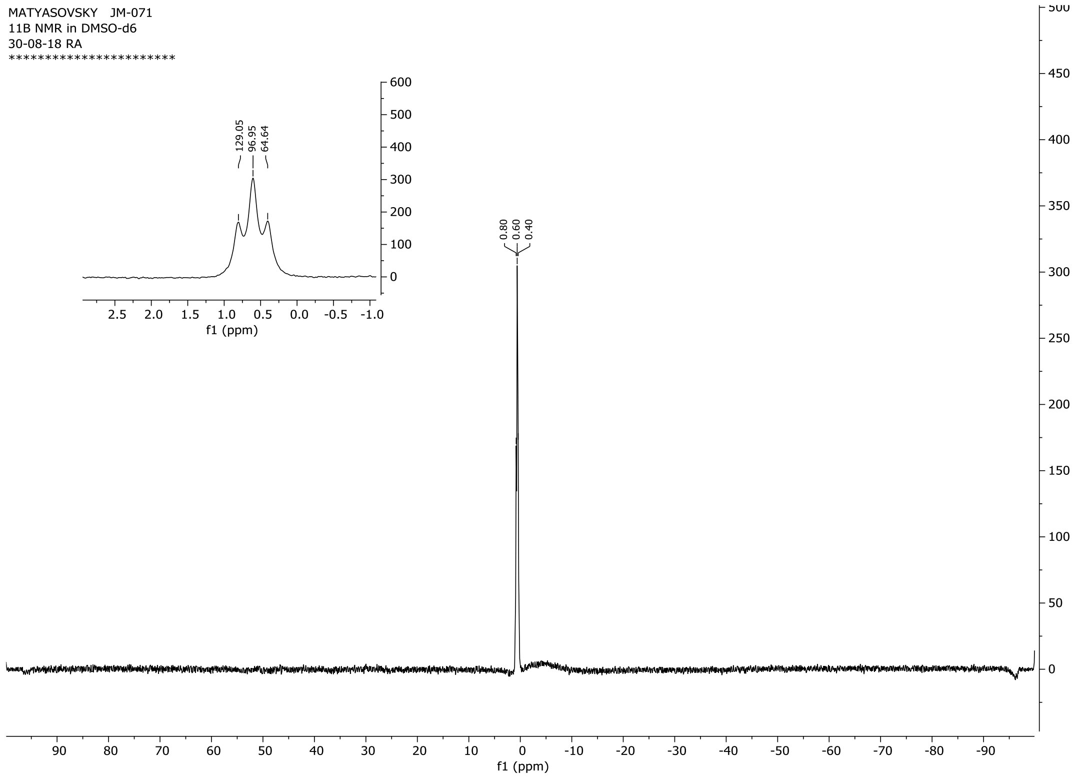

Figure S94. ^{11}B NMR spectrum.

3.1.3 Spectra of 5-(pent-1-yn-1-yl)-5-cytidine (rC^{Pent})

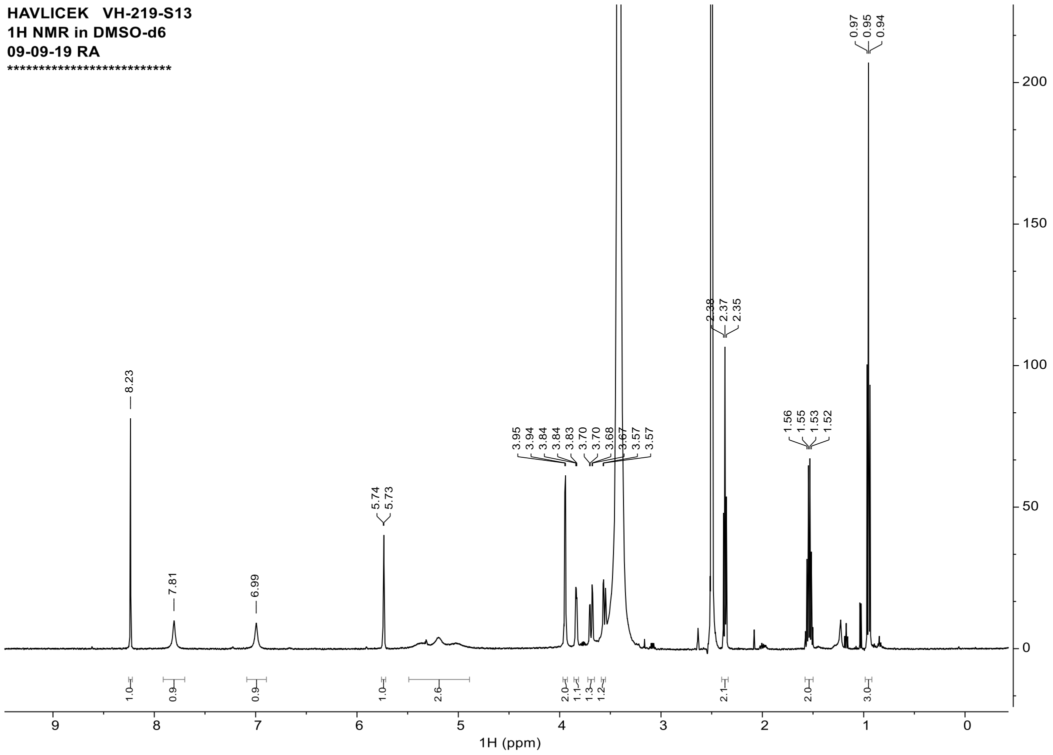

Figure S95. ^{1}H NMR spectrum.

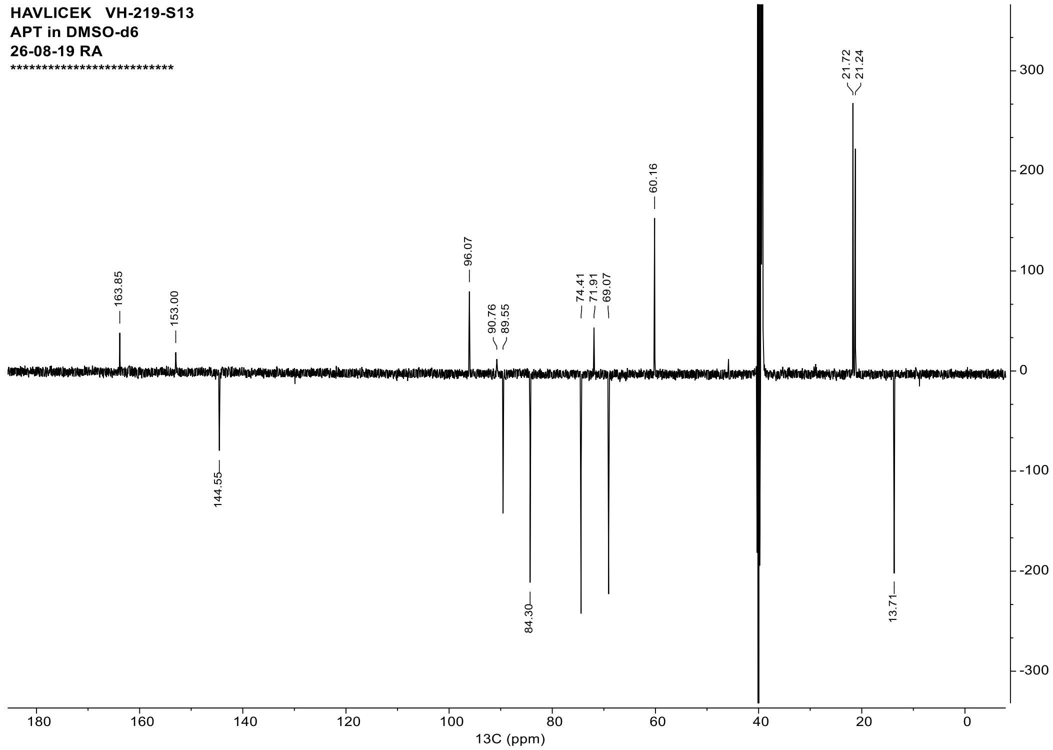

Figure S96. ^{13}C NMR spectrum.

3.1.4 Spectra of 7-(pent-1-yn-1-yl)-7-deazaguanosine (rG^{Pent})

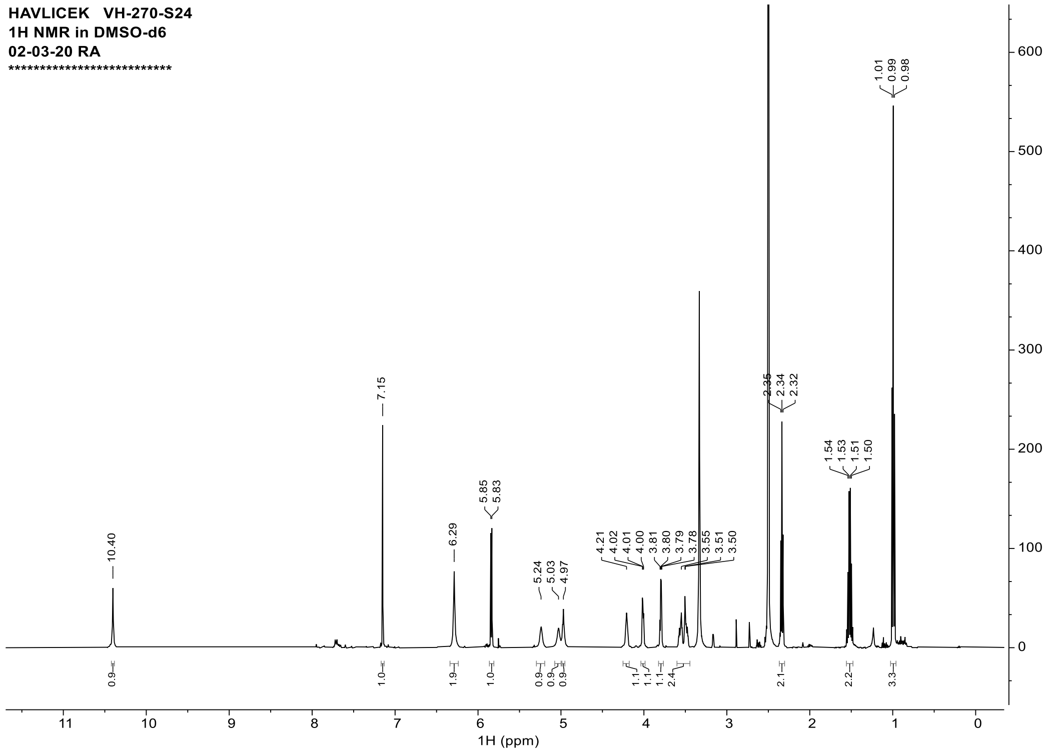

Figure S97. ^{1}H NMR spectrum.

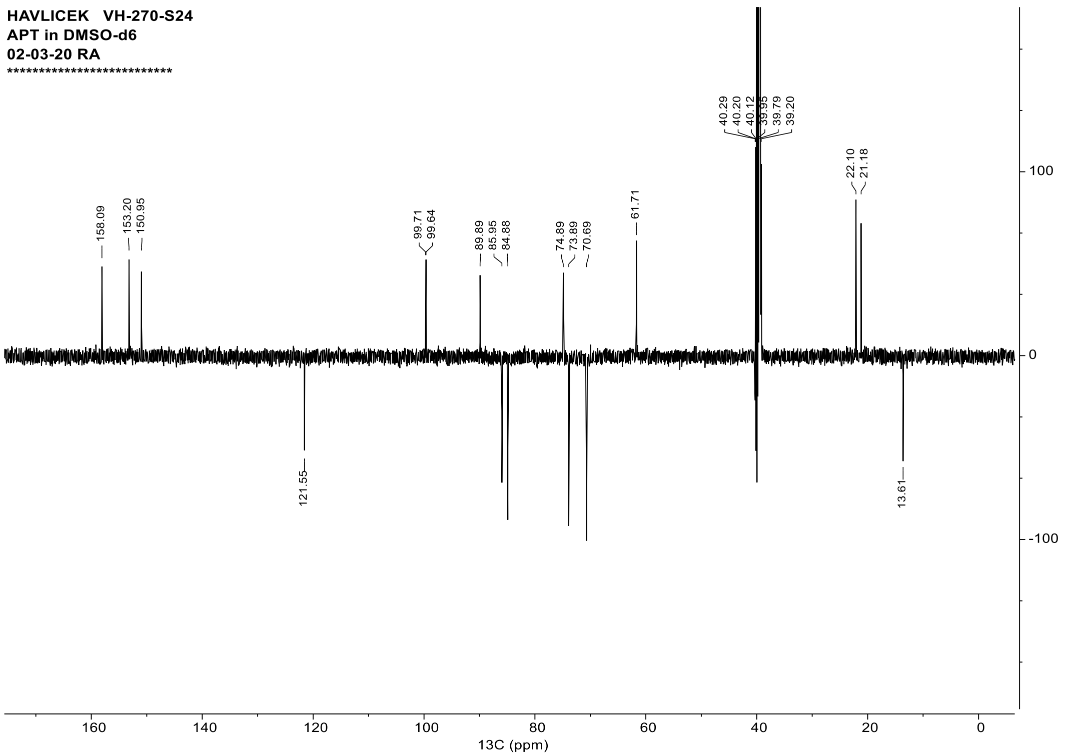

Figure S98. ^{13}C NMR spectrum.

3.2 NMR spectra of nucleotides

3.2.1 Spectra of 7-(5-formylthien-2-yl)-7-deazaadenosine-5'-*O*-triphosphate (rA^{FT}TP)

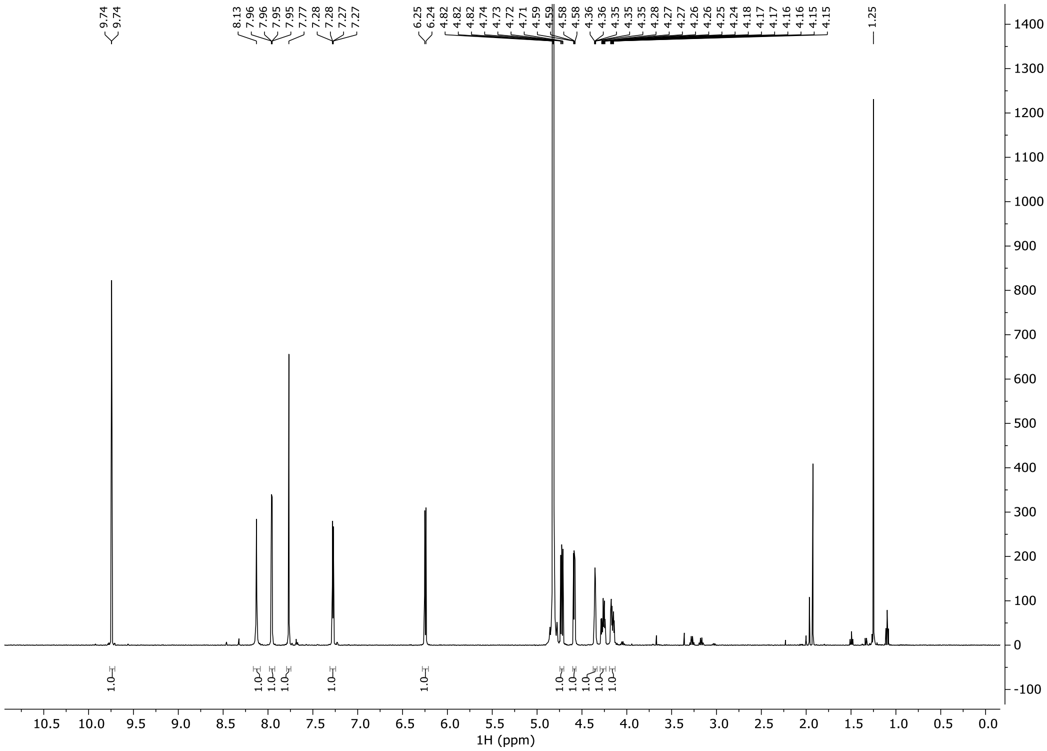

Figure S99. ^{1}H NMR spectrum.

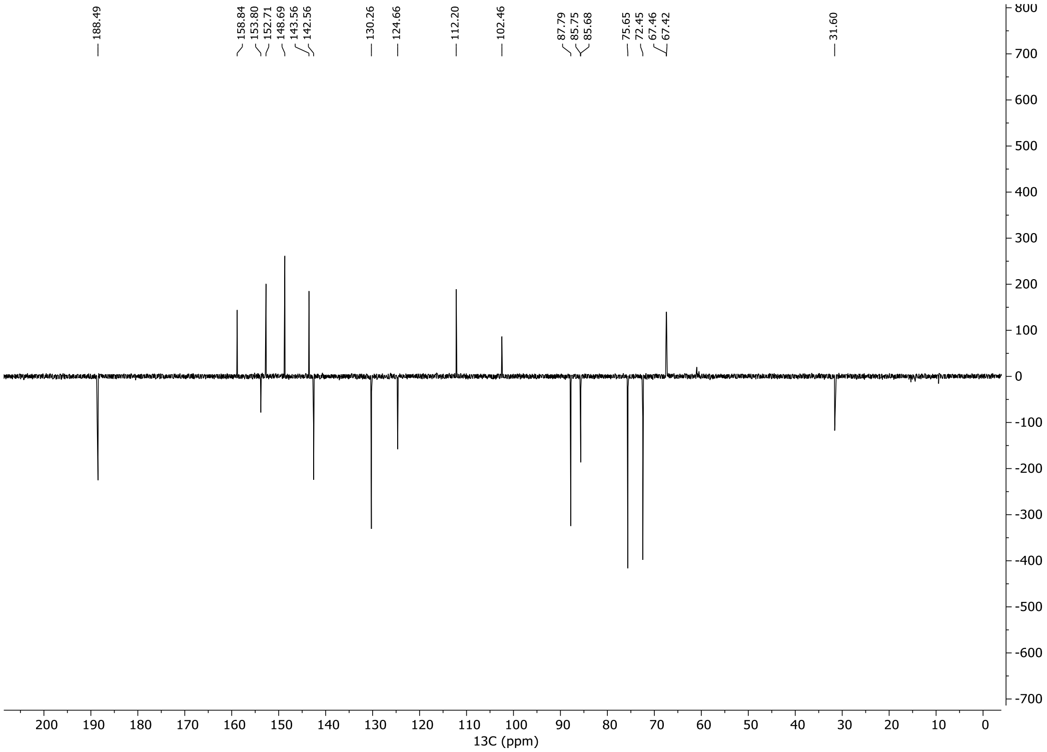

Figure S100. ^{13}C NMR spectrum.

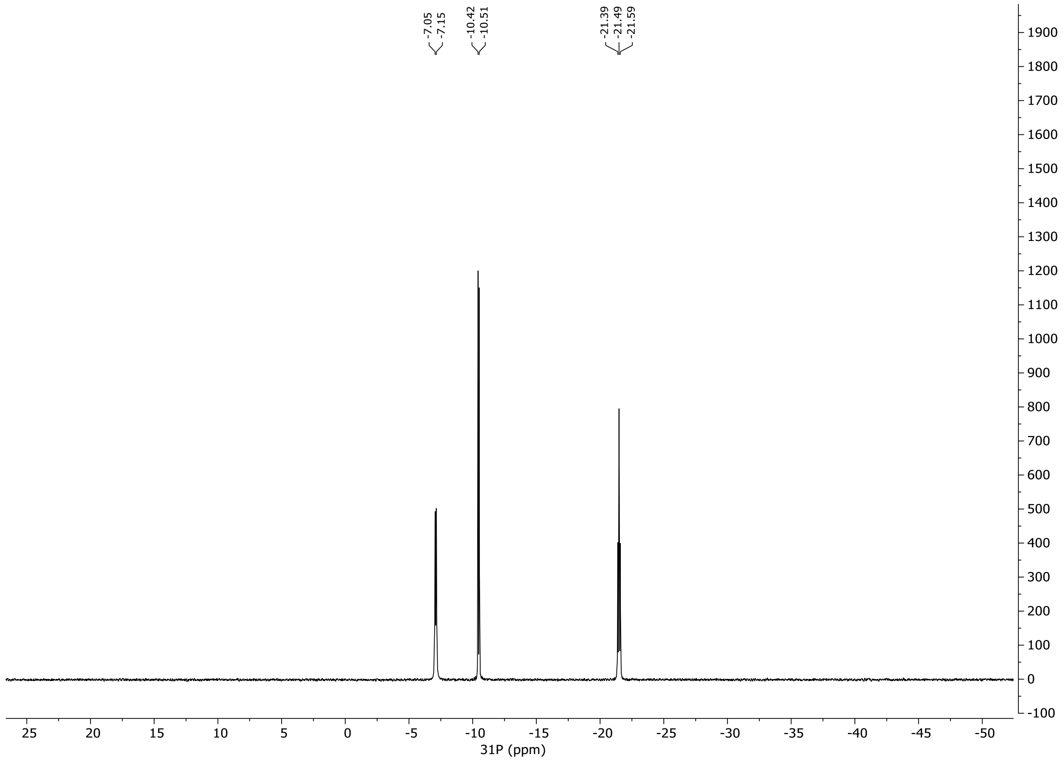

Figure S101. ^{31}P NMR spectrum.

3.2.2 Spectra of 7-(pent-1-yn-1-yl)-7-deazaadenosine-5'-*O*-triphosphate (rA^{Pent}TP)

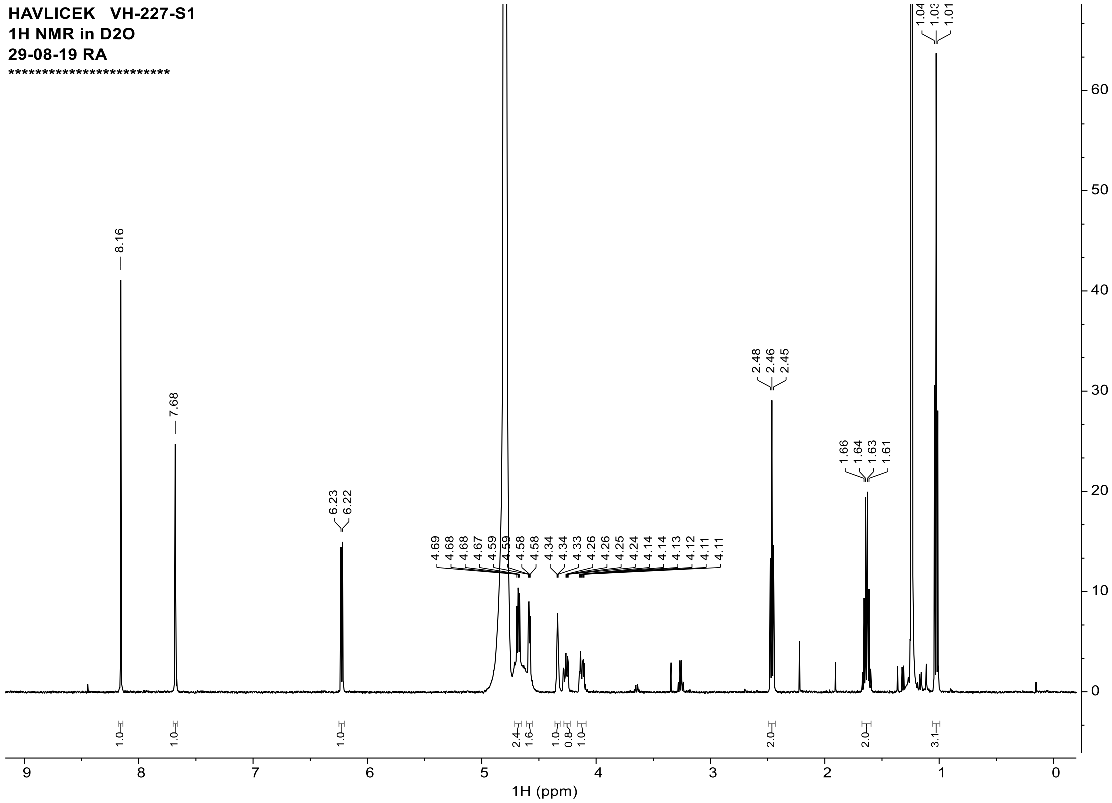

Figure S102. ^{1}H NMR spectrum.

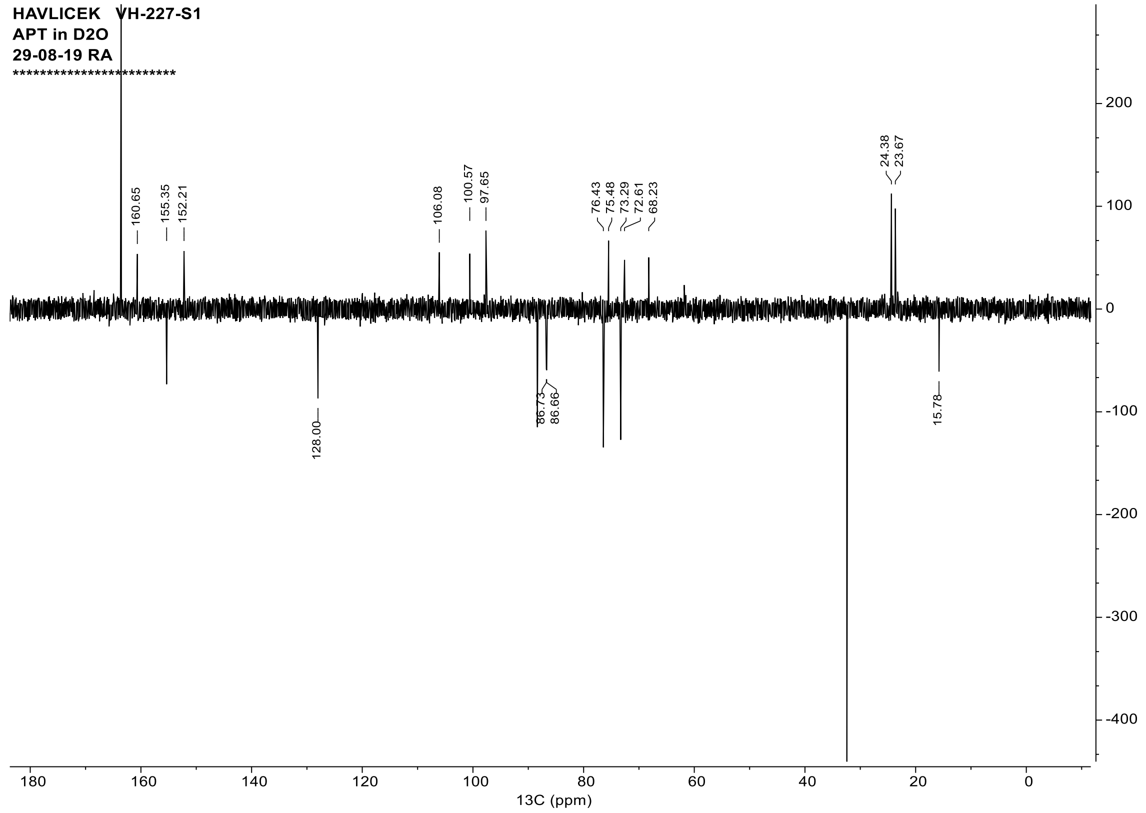

Figure S103. ^{13}C NMR spectrum.

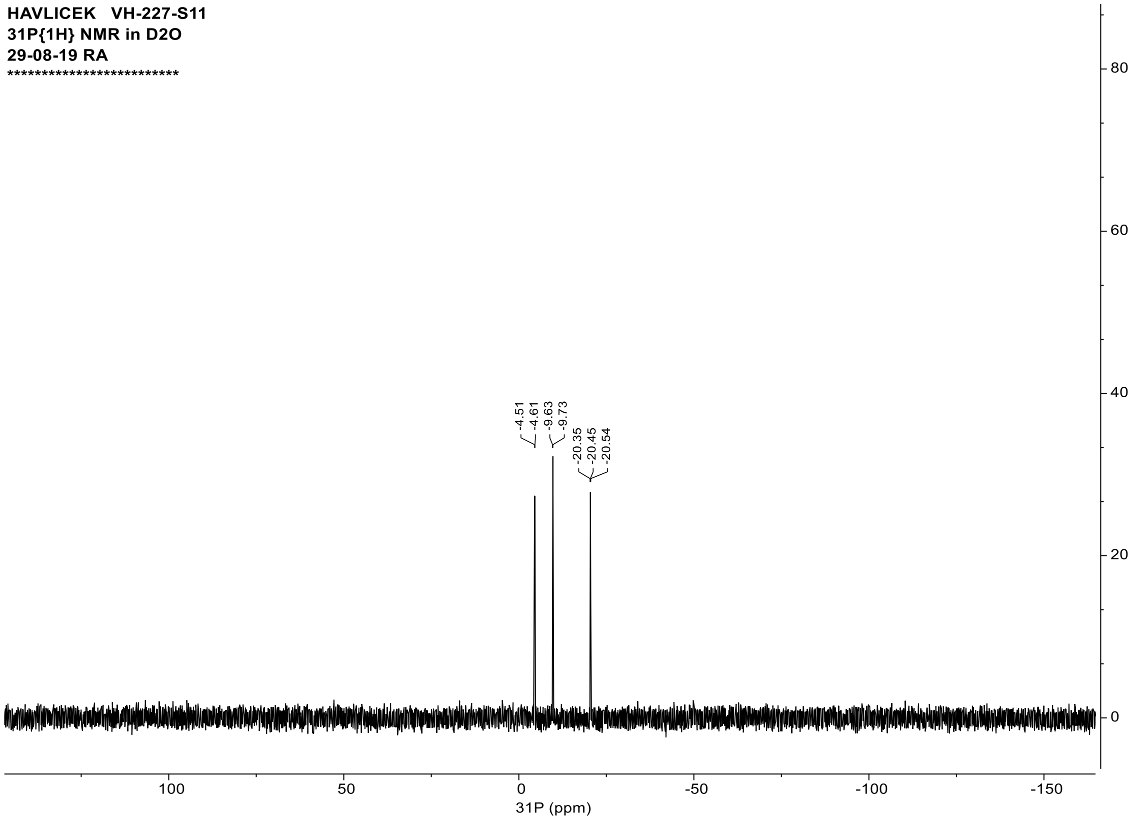

Figure S104. ^{31}P NMR spectrum.

3.2.3 Spectra of 5-{3-[N-(2-chloroacetamido)]-prop-2-yn-1-yl}-uridine-5'-*O*-triphosphate (rU^{CA}TP)

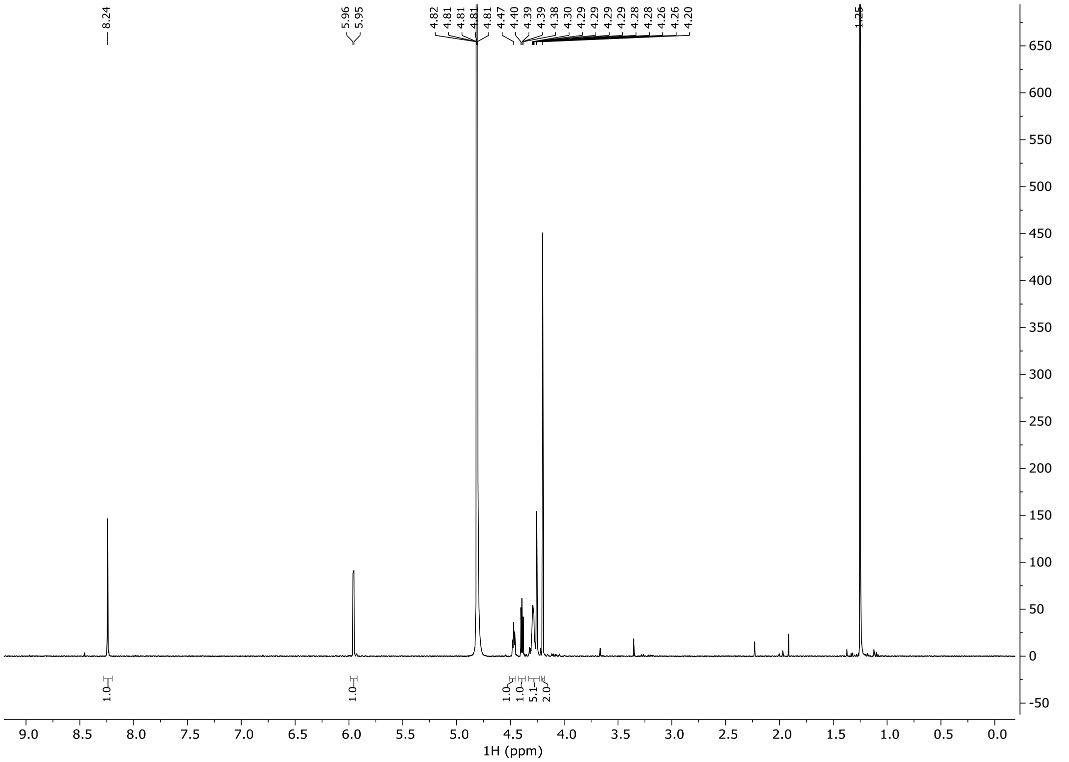

Figure S105. ^{1}H NMR spectrum.

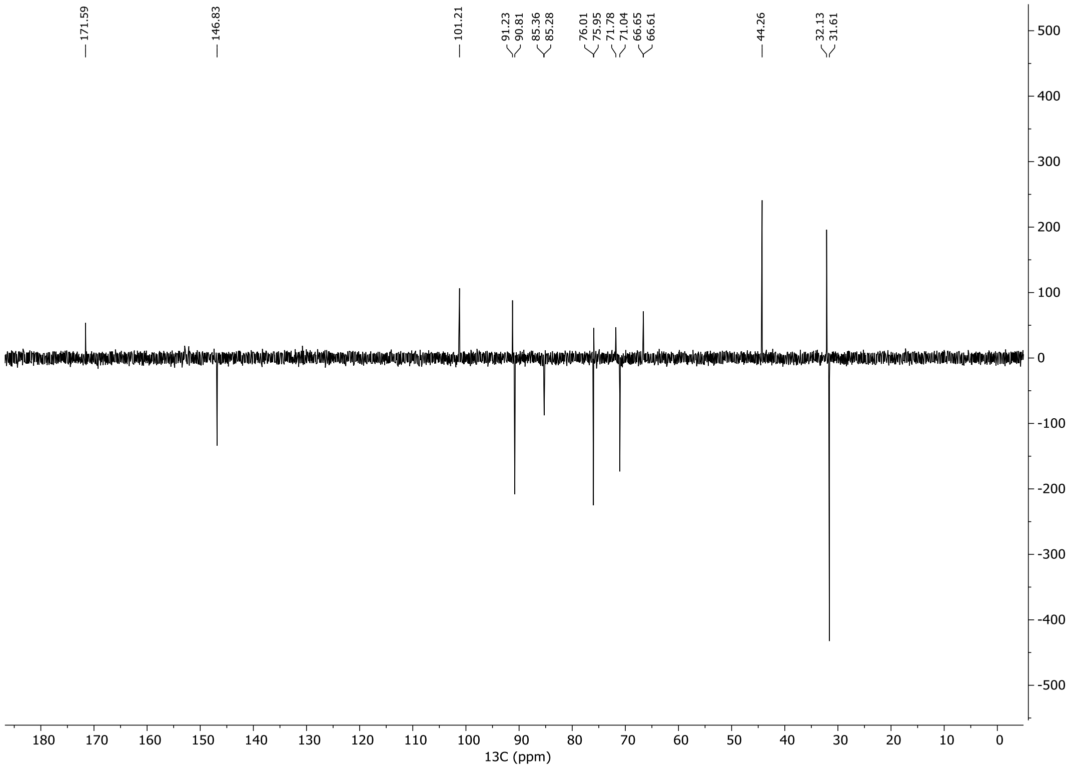

Figure S106. ^{13}C NMR spectrum.

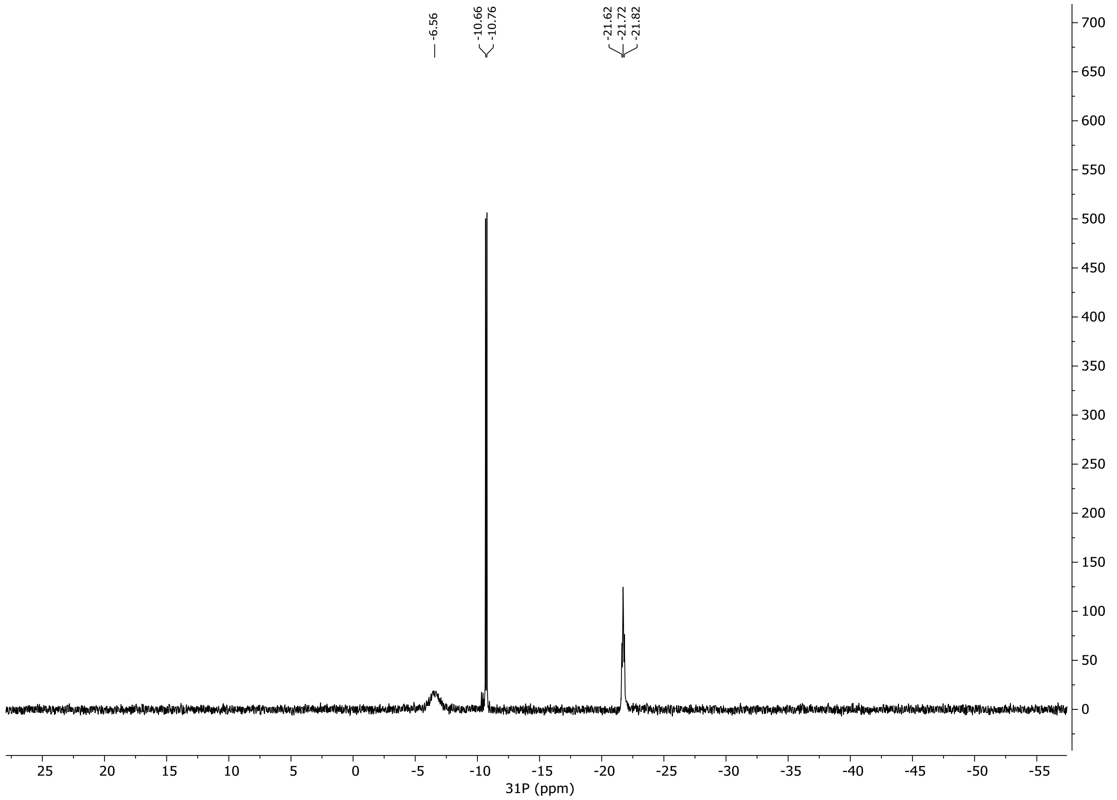

Figure S107. ^{31}P NMR spectrum.

3.2.4 Spectra of 5-(pent-1-yn-1-yl)-5-uridine-5’-*O*-triphosphate (rU^{Pent}TP)

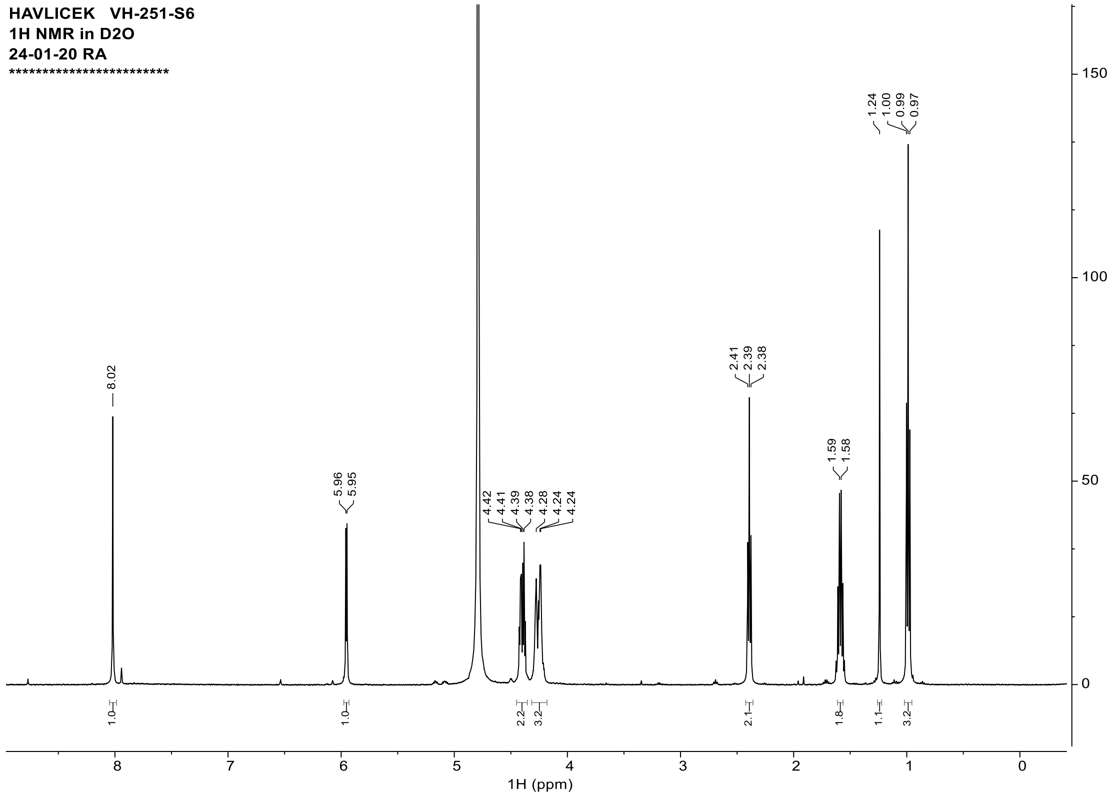

Figure S108. ^{1}H NMR spectrum.

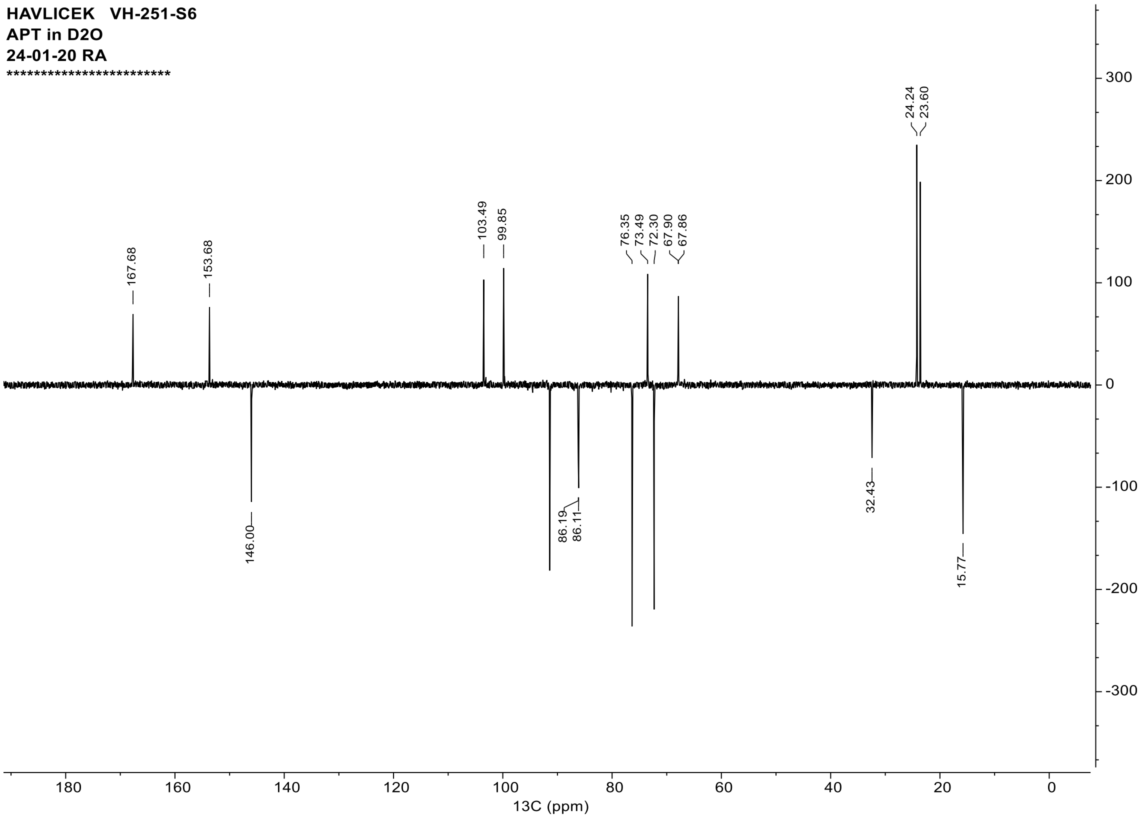

Figure S109. ^{13}C NMR spectrum.

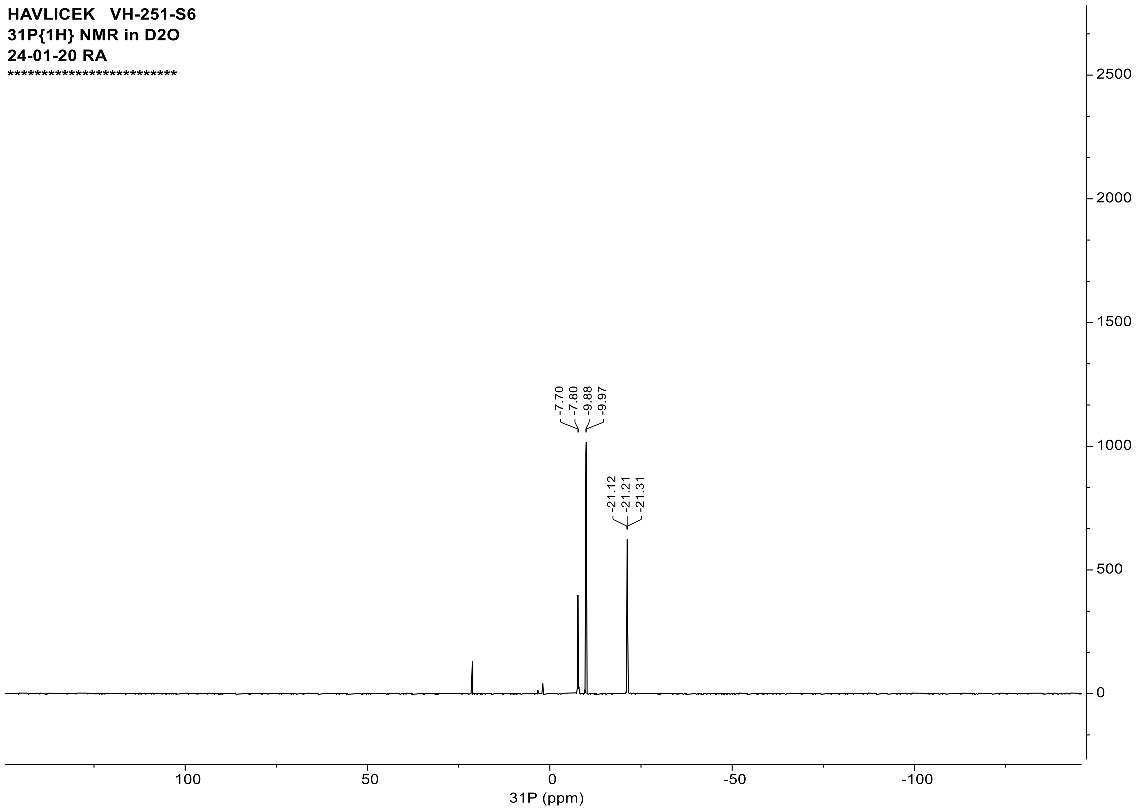

Figure S110. ^{31}P NMR spectrum.

3.2.5 5-{3-[N-(2-chloroacetamido)]-prop-2-yn-1-yl}-cytidine-5'-*O*-triphosphate (rC^{CA}TP)

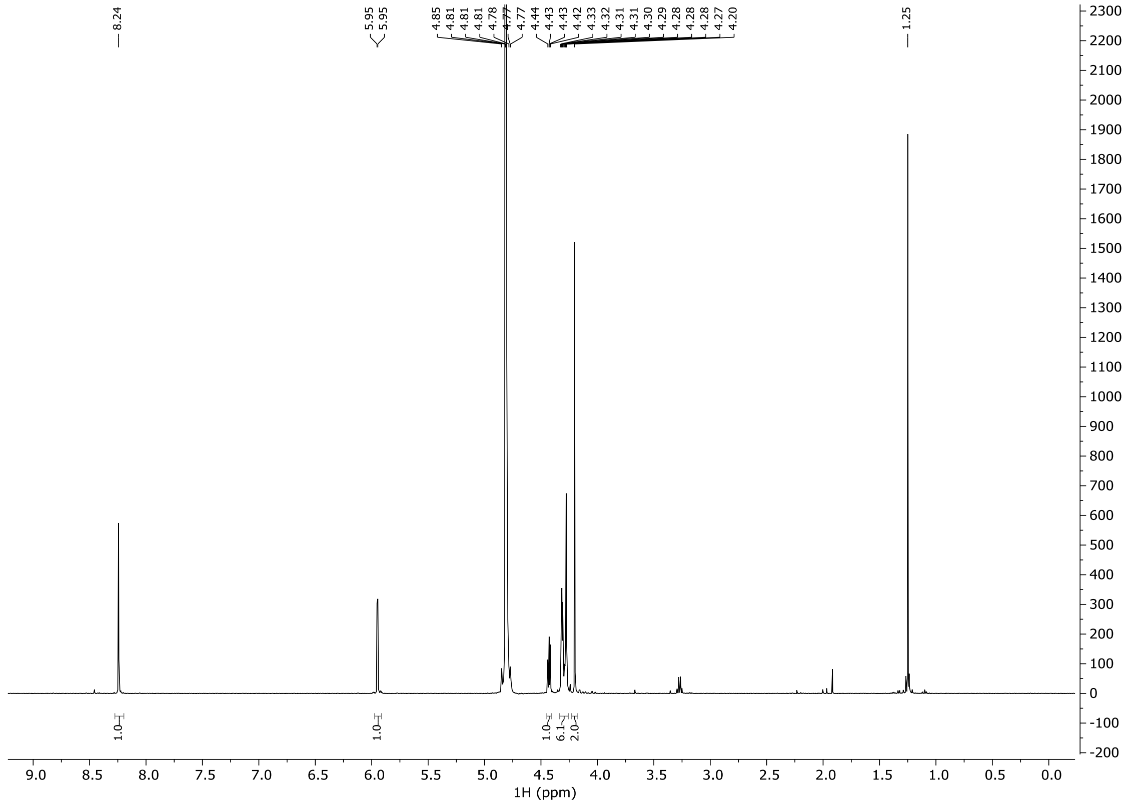

Figure S111. ^{1}H NMR spectrum.

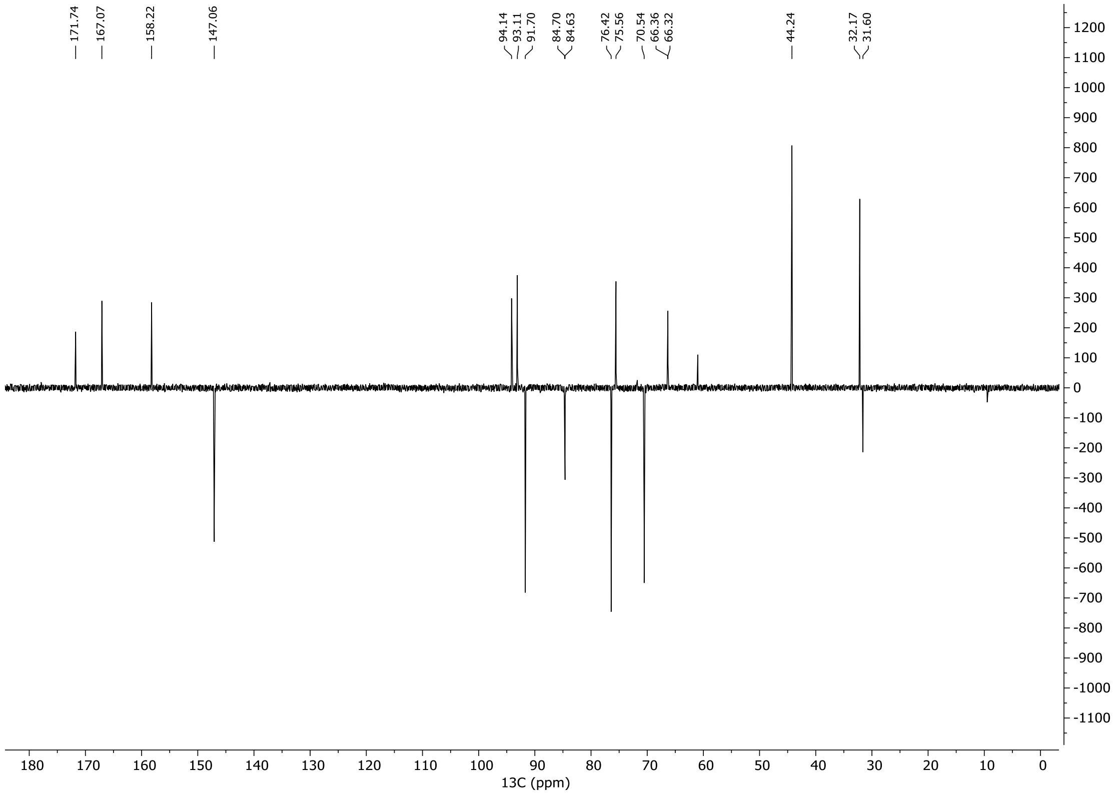

Figure S112. ^{13}C NMR spectrum.

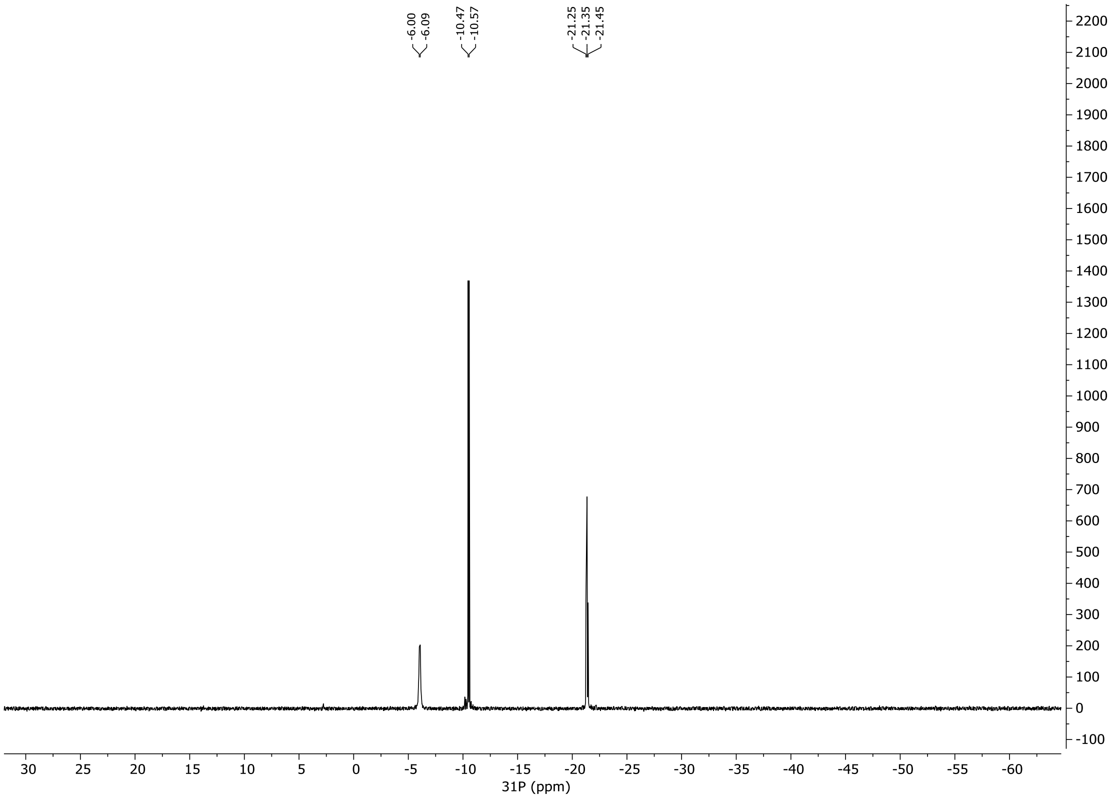

Figure S113. ^{31}P NMR spectrum.

3.2.6 5-(5-formylthien-2-yl)-cytidine-5'-*O*-triphosphate (rC^{FT}TP)

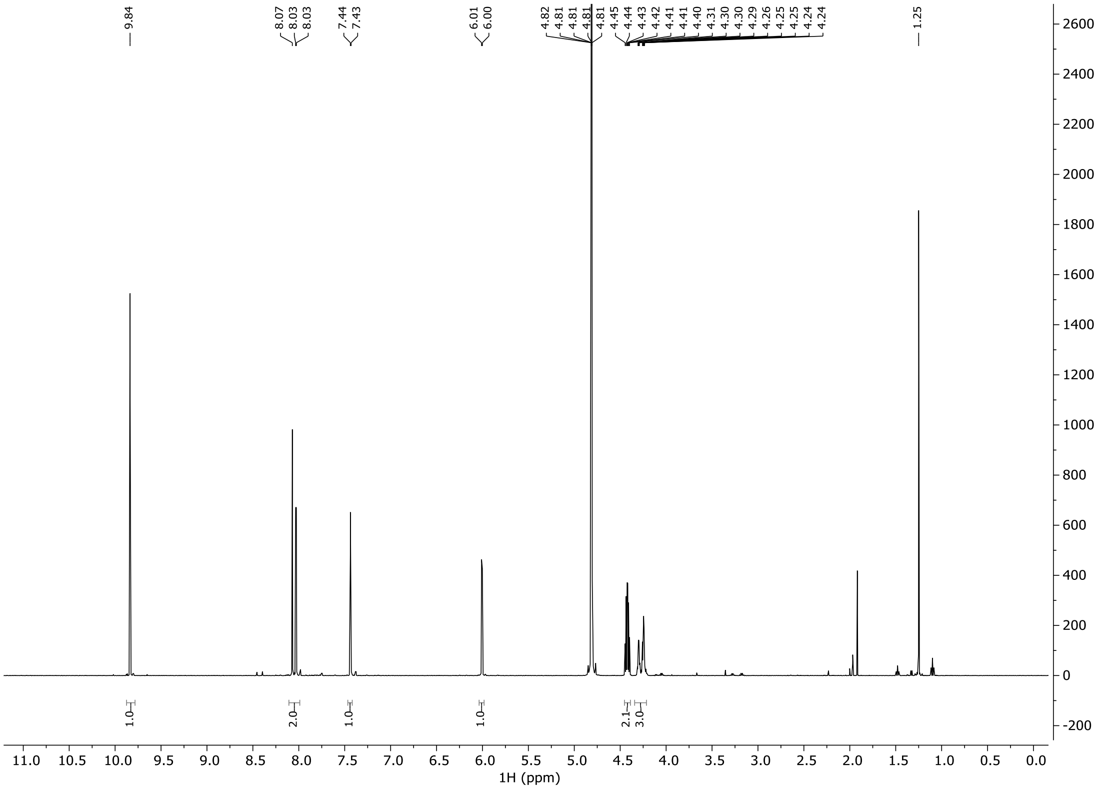

Figure S114. ^{1}H NMR spectrum.

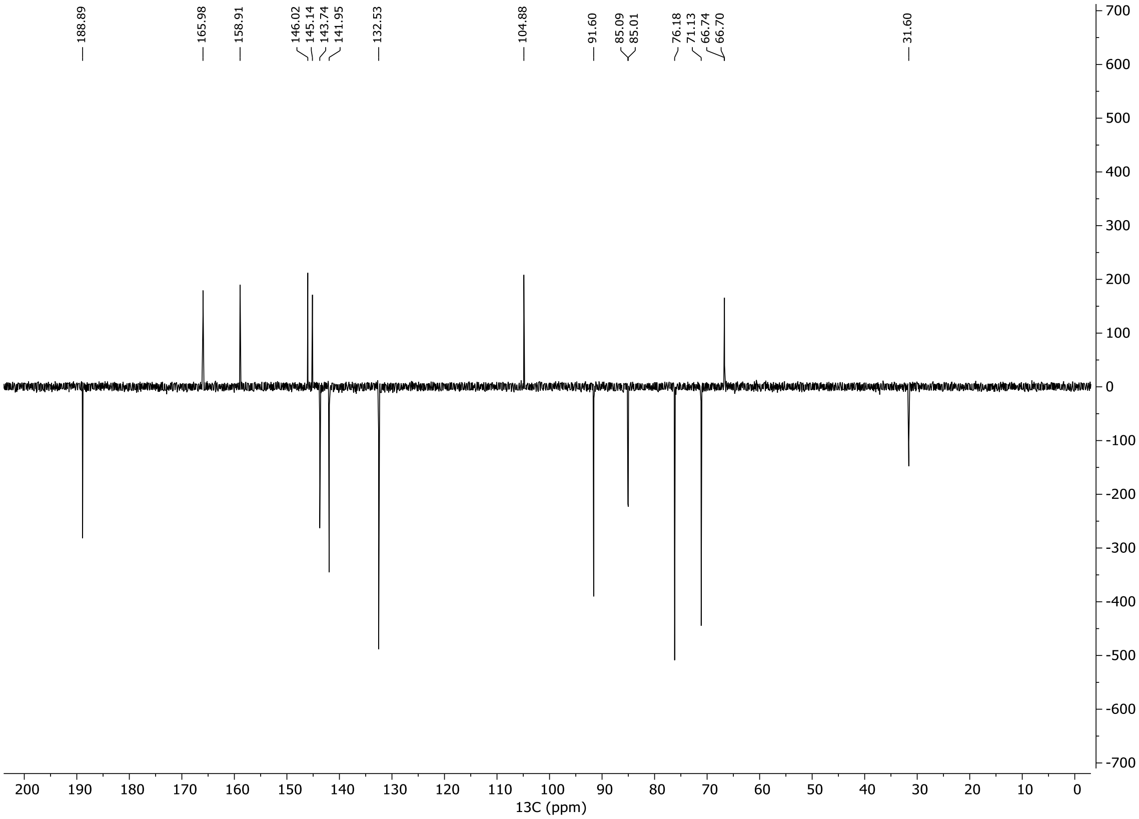

Figure S115. ^{13}C NMR spectrum.

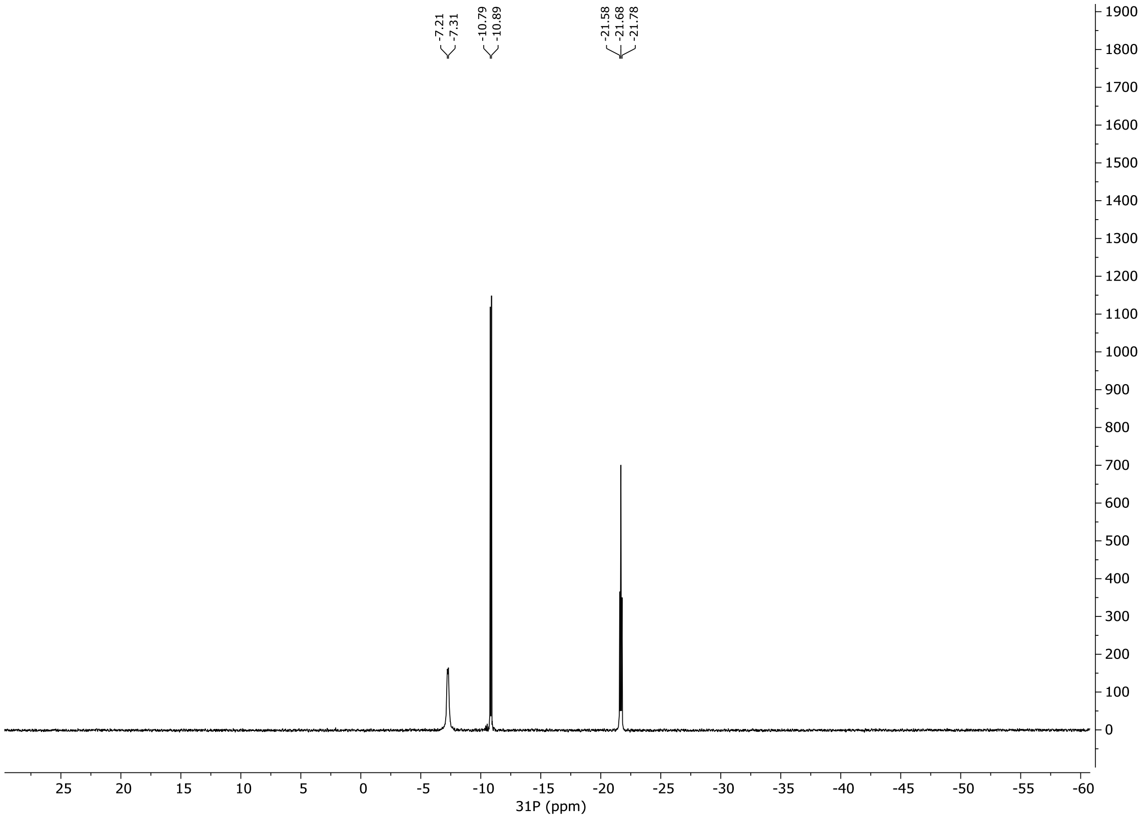

Figure S116. ^{31}P NMR spectrum.

3.2.7 Spectra of 5-{3-[4-(5,5-difluoro-1,3,7,9-tetramethyl-5*H*-4λ^4,5λ^4-dipyrrolo[1,2-c:2',1'-f][1,3,2]diazaborinin-10-yl)-3,5-dimethylphenoxy]prop-1-yn-1-yl}-cytidine-5´-*O*-triphosphate (rC^{mBdp}TP)

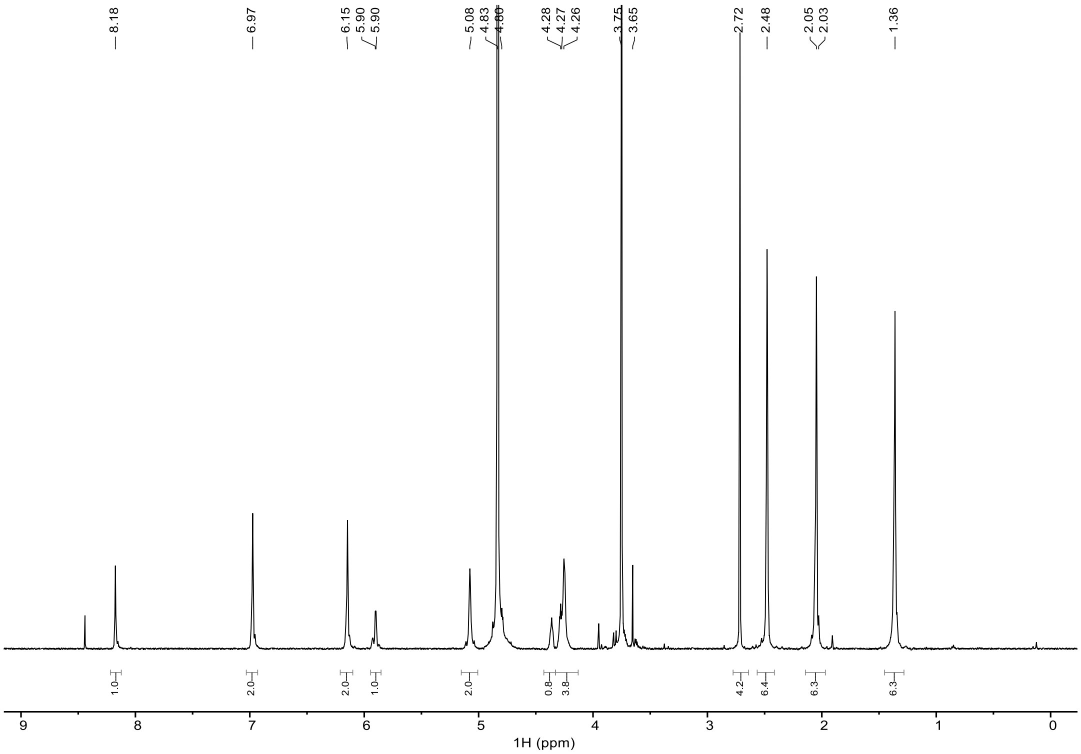

Figure S117. ^{1}H NMR spectrum.

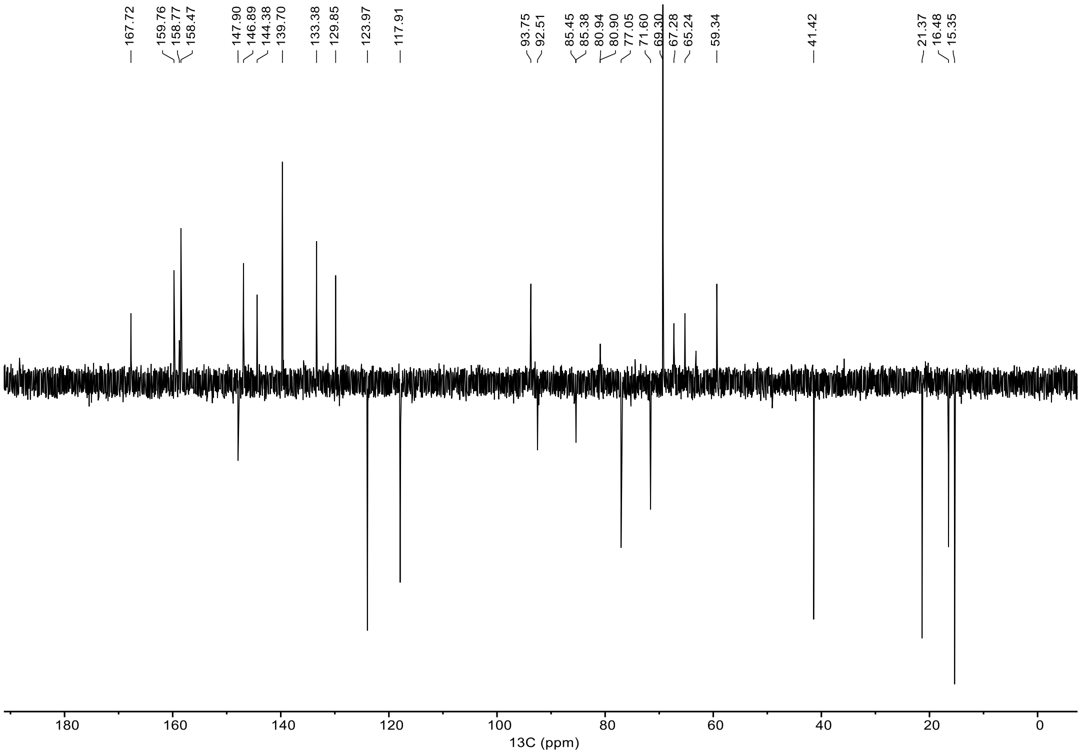

Figure S118. ^{13}C NMR spectrum.

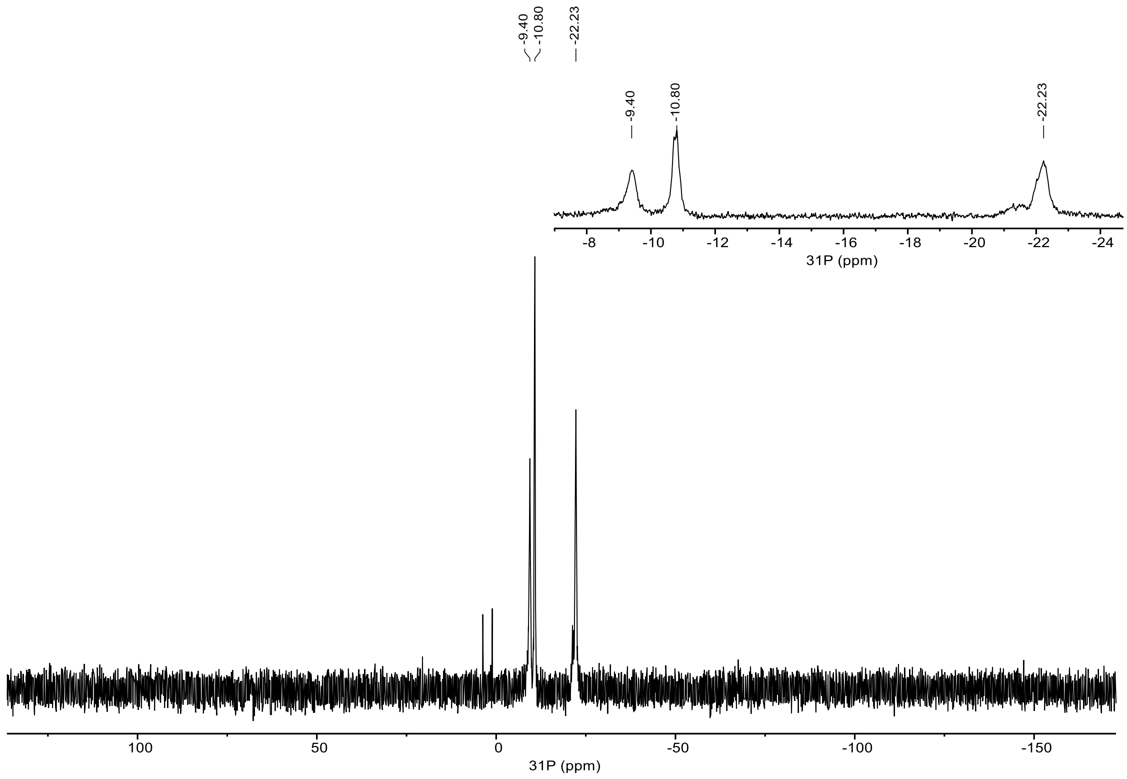

Figure S119. ^{31}P NMR spectrum.

3.2.8 Spectra of 5-(pent-1-yn-1-yl)-5-cytidine-5'-*O*-triphosphate (rC^{Pent}TP)

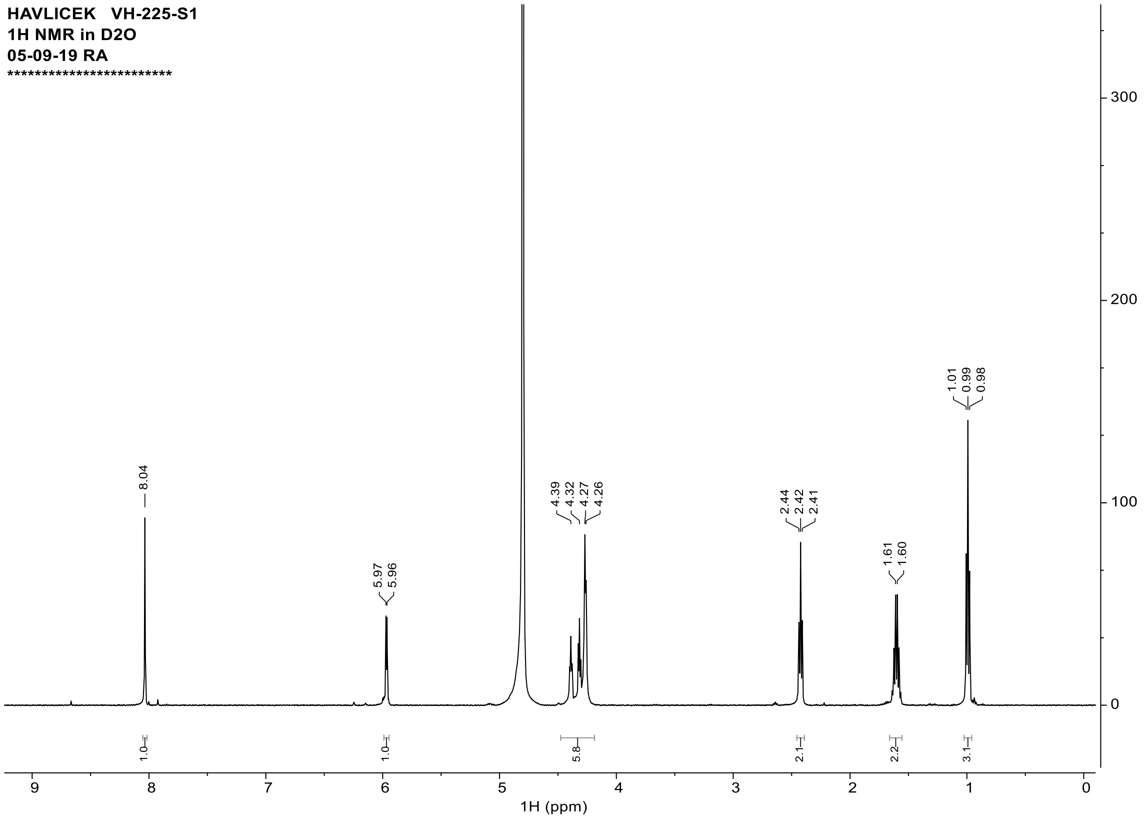

Figure S120. ^{1}H NMR spectrum.

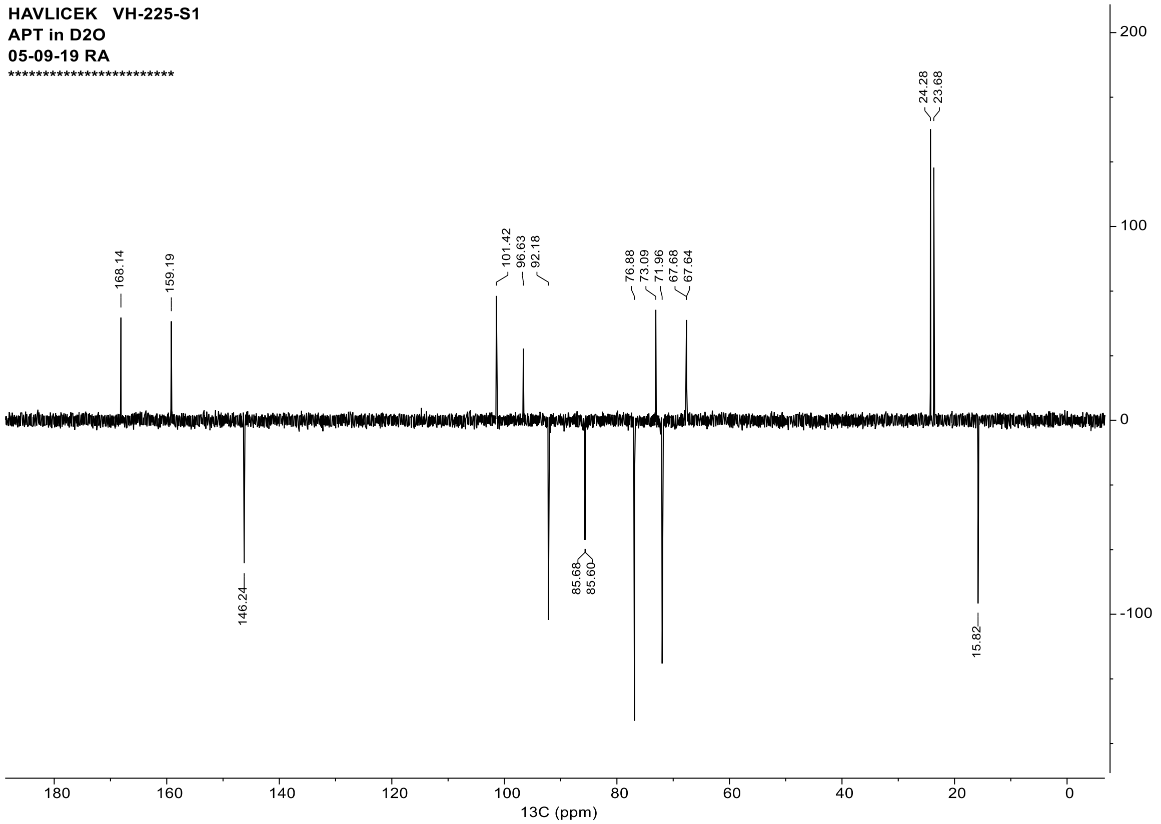

Figure S121. ^{13}C NMR spectrum.

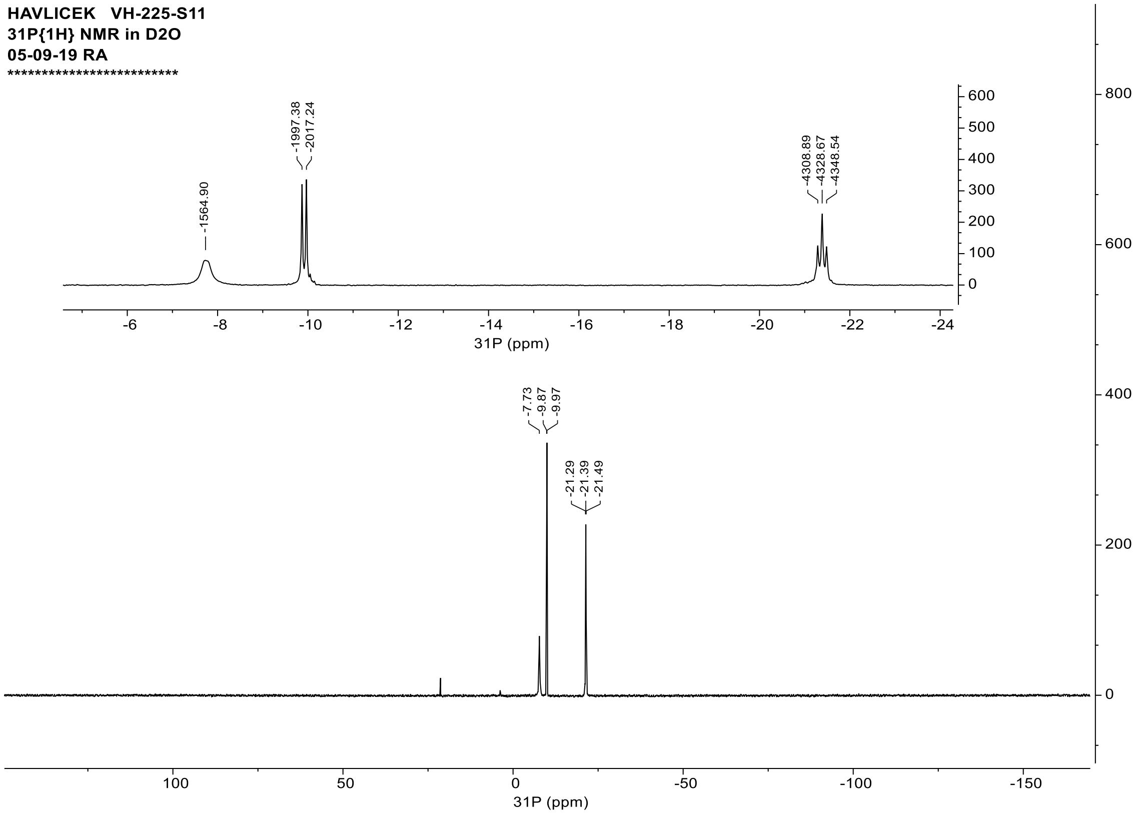

Figure S122. ^{31}P NMR spectrum.

3.2.9 Spectra of 7-(pent-1-yn-1-yl)-7-deazaguanosine-5'-*O*-triphosphate (rG^{Pent}TP)

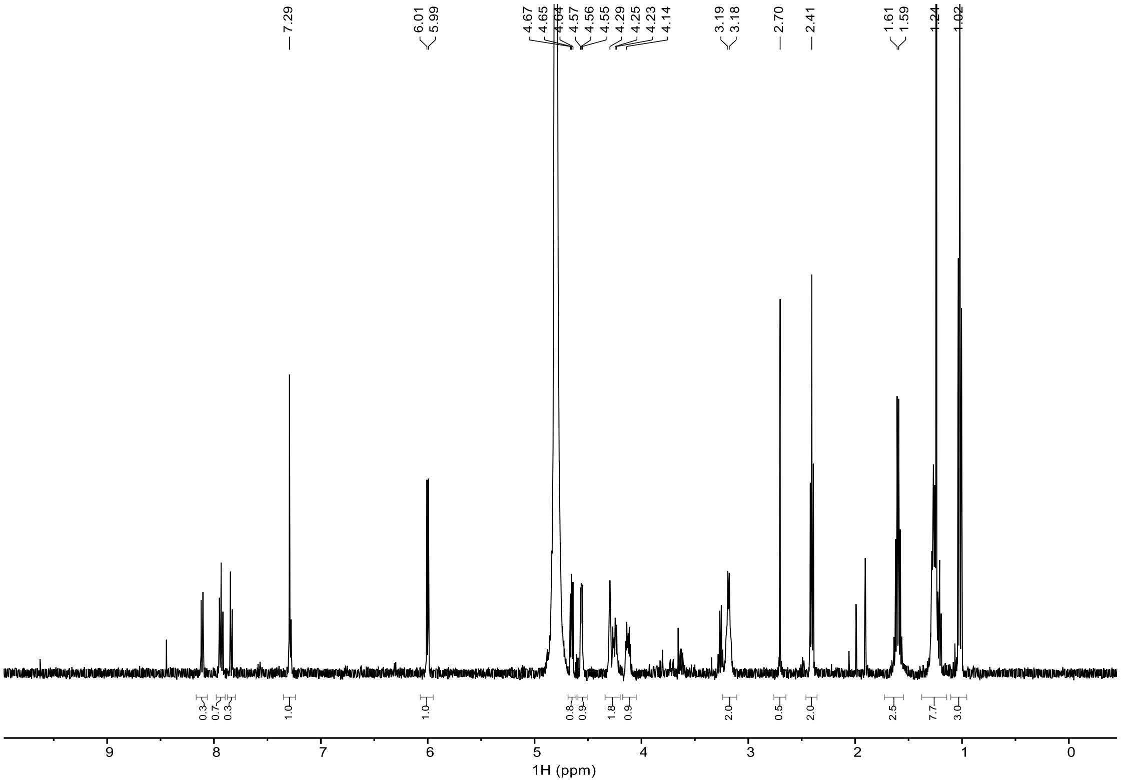

Figure S123. ^{1}H NMR spectrum.

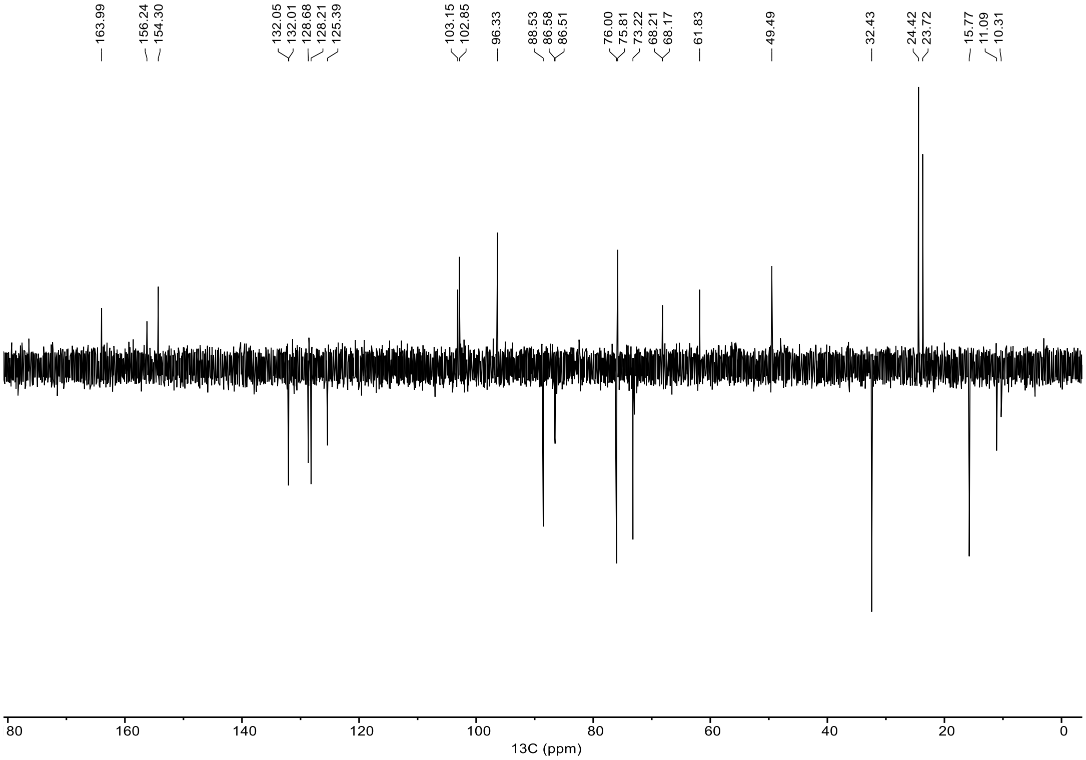

Figure S124. ^{13}C NMR spectrum.

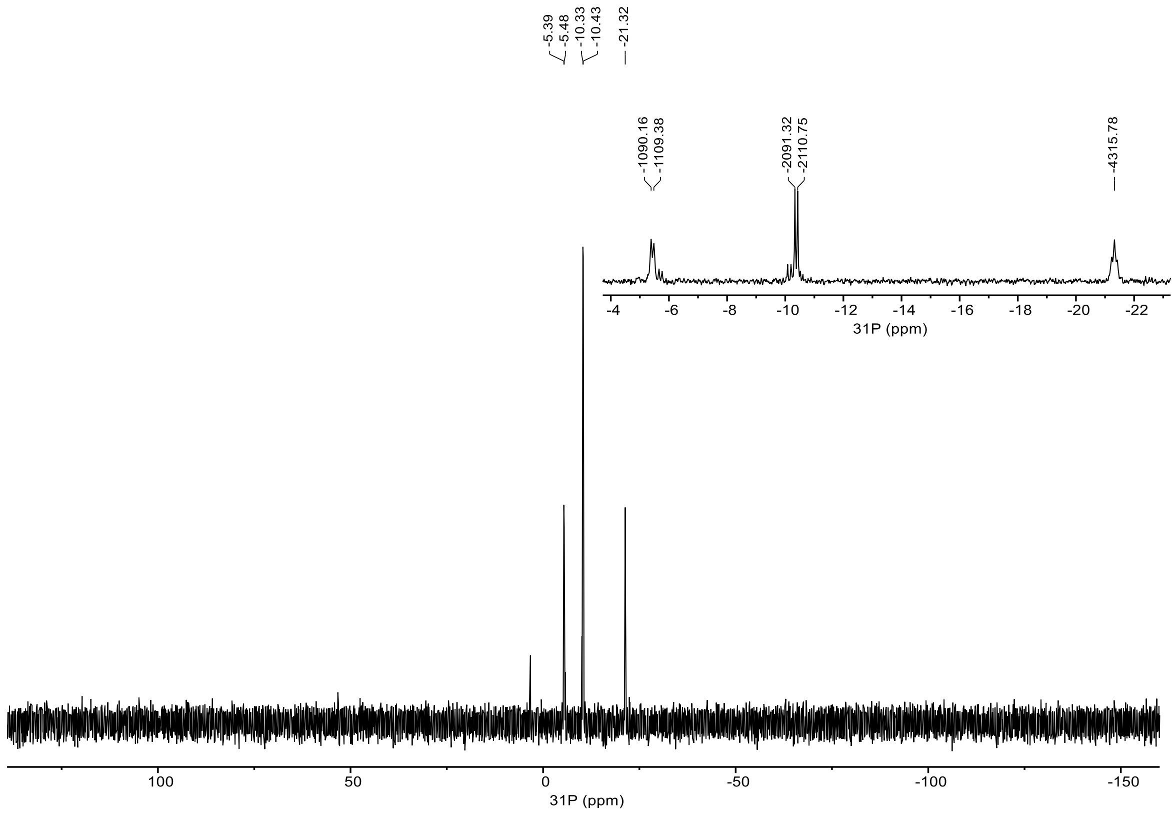

Figure S125. ^{31}P NMR spectrum.

4 MS analysis

4.1 MS-MALDI-TOF spectra

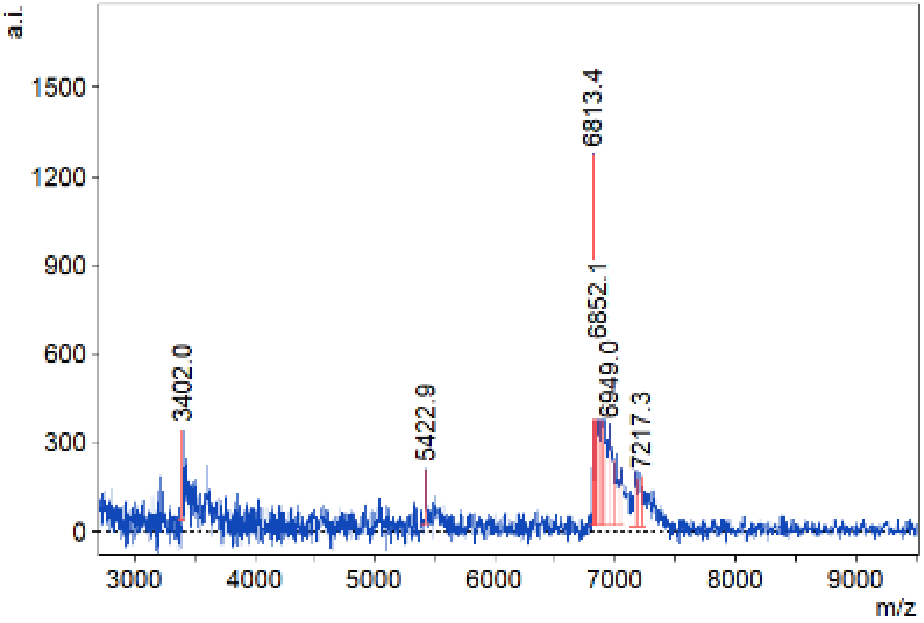

Figure S126. 19RNA_A^{E}, calculated mass: 6810.8 Da, found mass: 6813.4 Da (product).

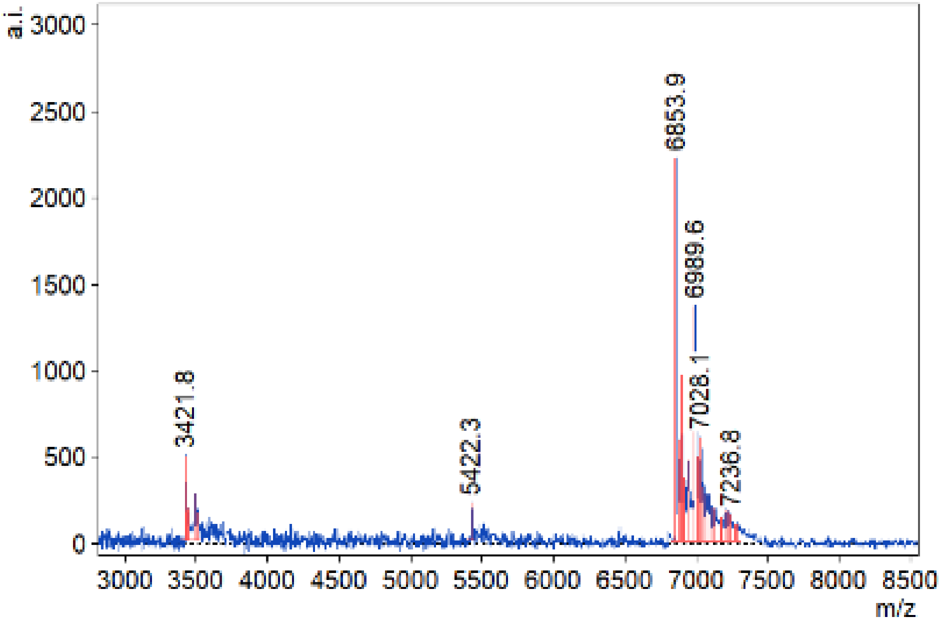

Figure S127. 19RNA_A^{Pent}, calculated mass: 6852.9 Da, found mass: 6853.9 Da (product).

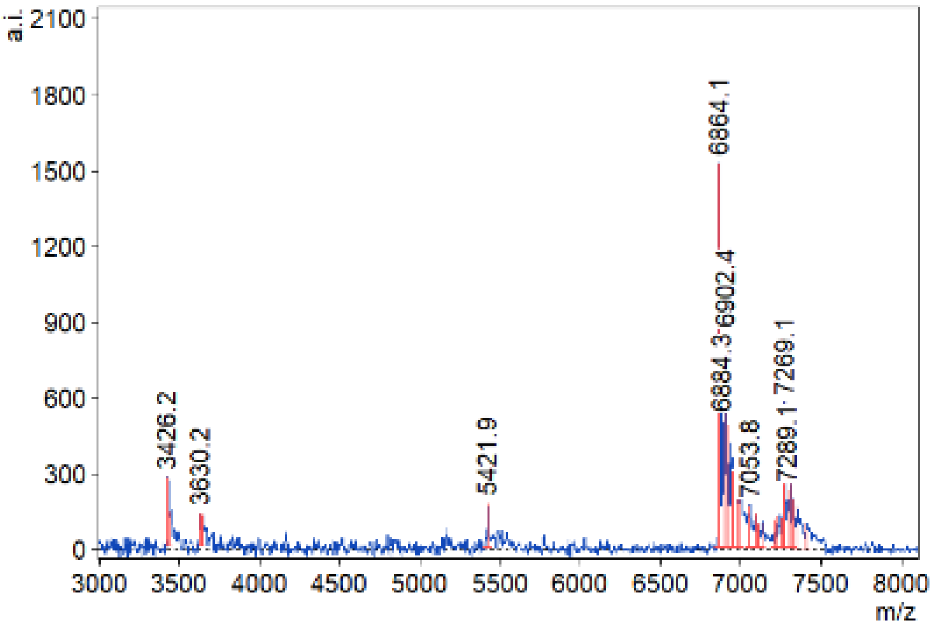

Figure S128. 19RNA_A^{Ph}, calculated mass: 6862.9 Da, found mass: 6864.1 Da (product).

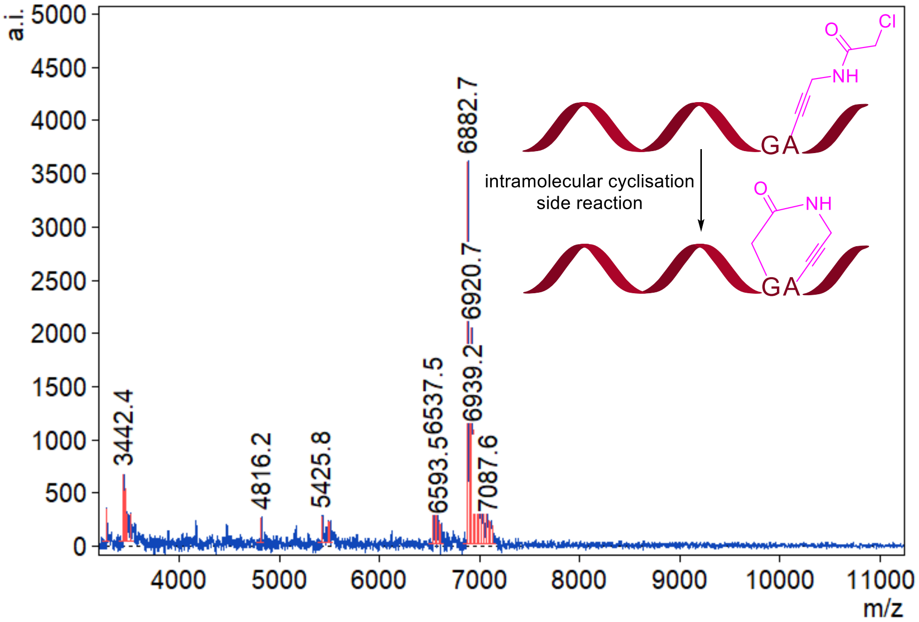

Figure S129. (full spectrum); **19RNA_A^{CA}**, calculated mass: 6915.6 Da, found mass: 6920.7 Da (product); found mass: 6882.7 Da (product of intramolecular cyclisation side reaction = product – HCl).

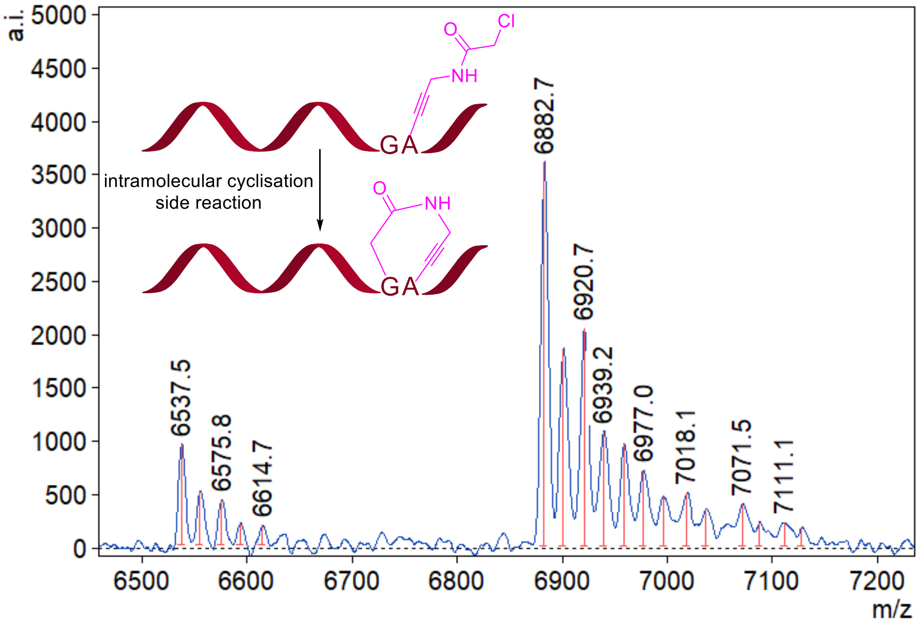

Figure S130. (magnified area of interest); **19RNA_A^{CA}**, calculated mass: 6915.6 Da, found mass: 6920.7 Da (product); found mass: 6882.7 Da (product of intramolecular cyclisation side reaction = product – HCl).

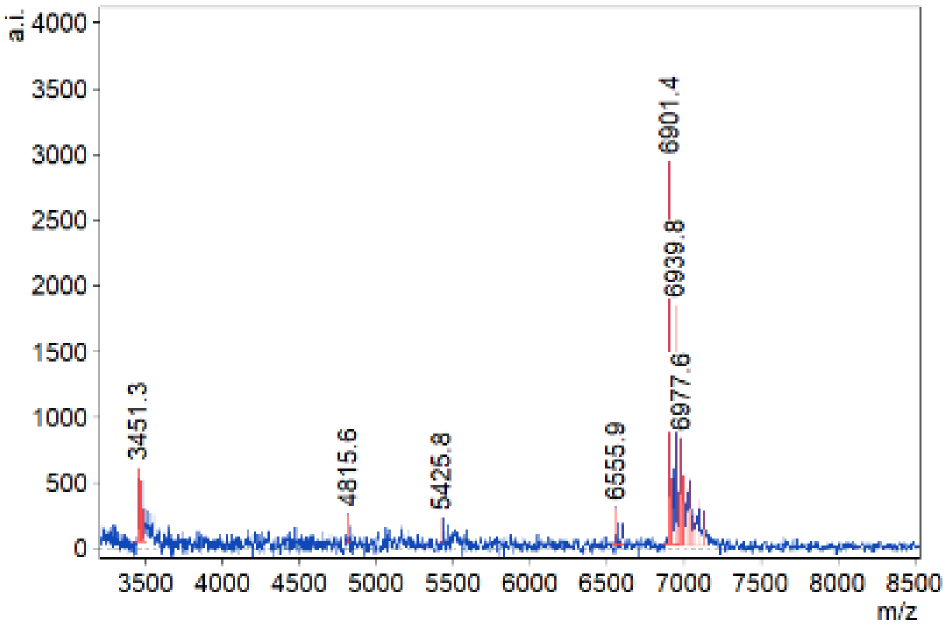

Figure S131. 19RNA_A^{FT}, calculated mass: 6896.9 Da, found mass: 6901.4 Da (product).

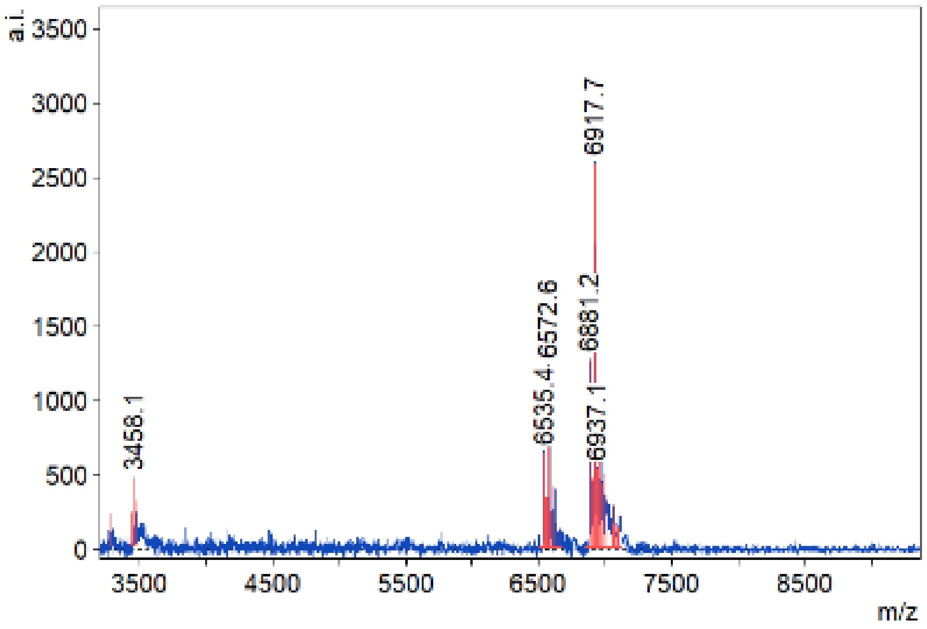

Figure S132. 19RNA_A^{CA}, calculated mass: 6915.6 Da, found mass: 6917.7 Da (product).

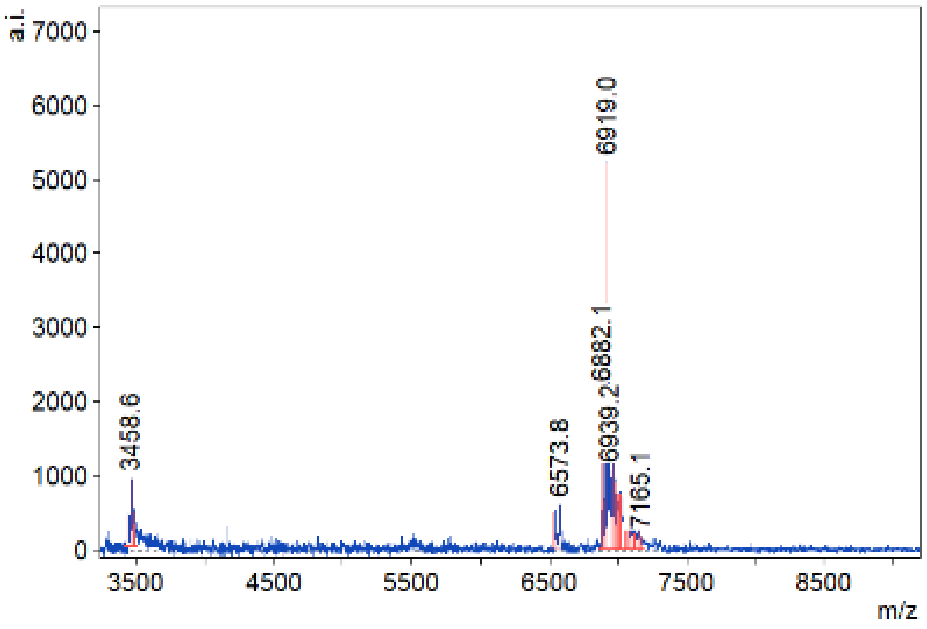

Figure S133. 19RNA_A^{CA}, calculated mass: 6915.6 Da, found mass: 6919.0 Da (product).

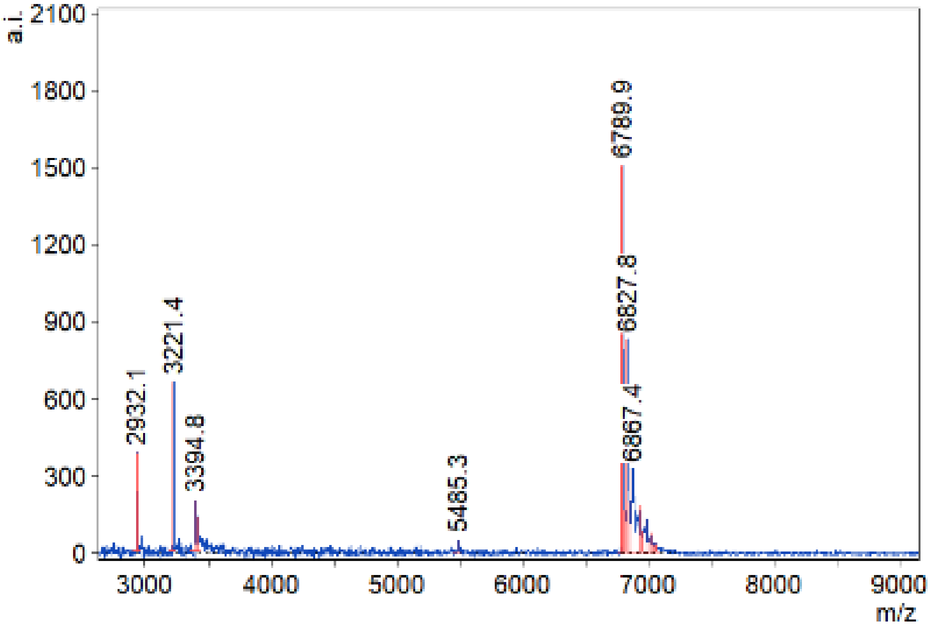

Figure S134. 19RNA_U^{E}, calculated mass: 6788.7 Da, found mass: 6789.90 Da (product).

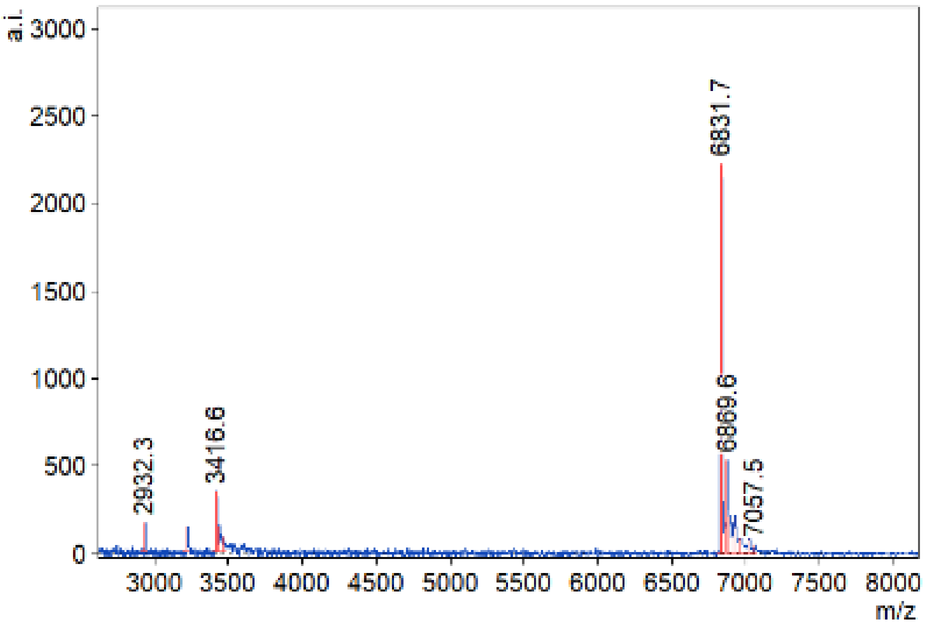

Figure S135. 19RNA_U^{Pent}, calculated mass: 6830.8 Da, found mass: 6831.7 Da (product).

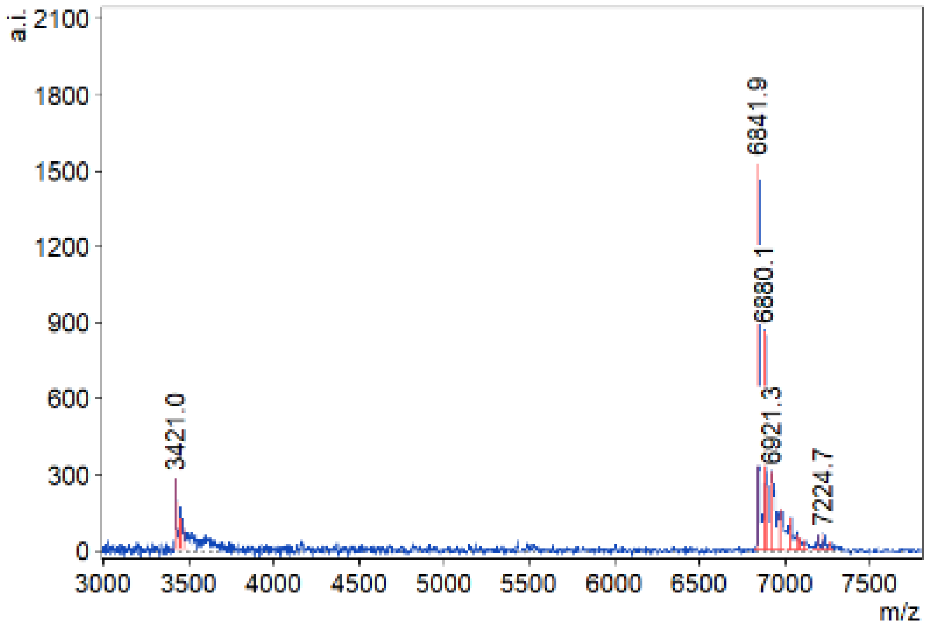

Figure S136. 19RNA_U^{Ph}, calculated mass: 6840.8 Da, found mass: 6841.9 Da (product).

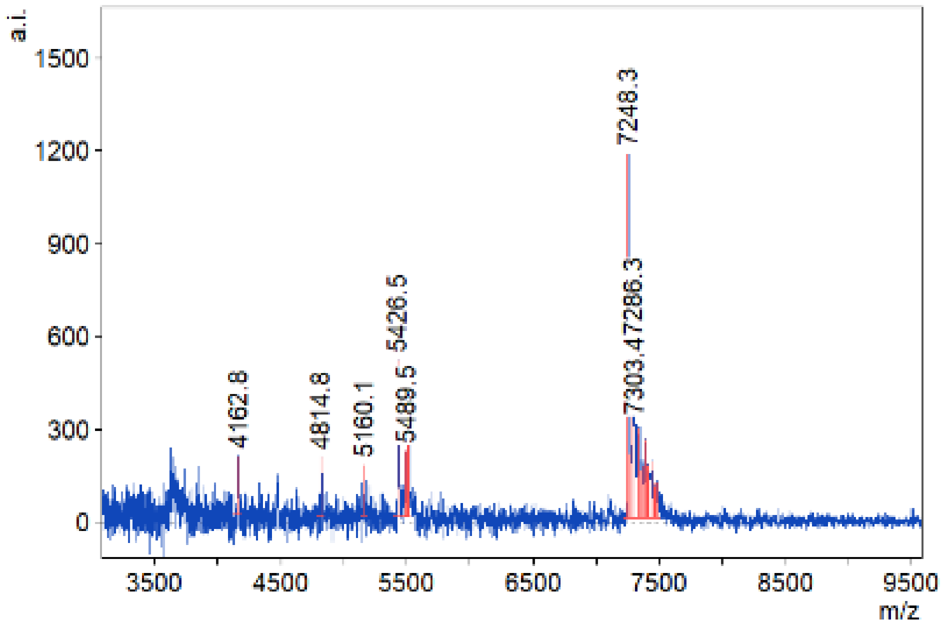

Figure S137. 19RNA_U^{Bio}, calculated mass: 7244.3 Da, found mass: 7248.3 Da (product).

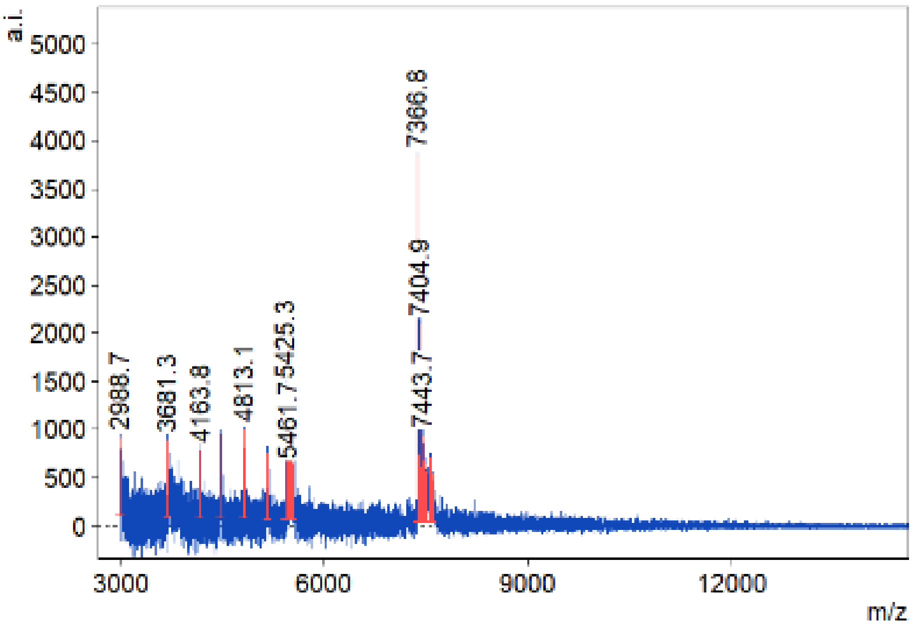

Figure S138. 19RNA_U^{Dig}, calculated mass: 7363.5 Da, found mass: 7366.8 Da (product).

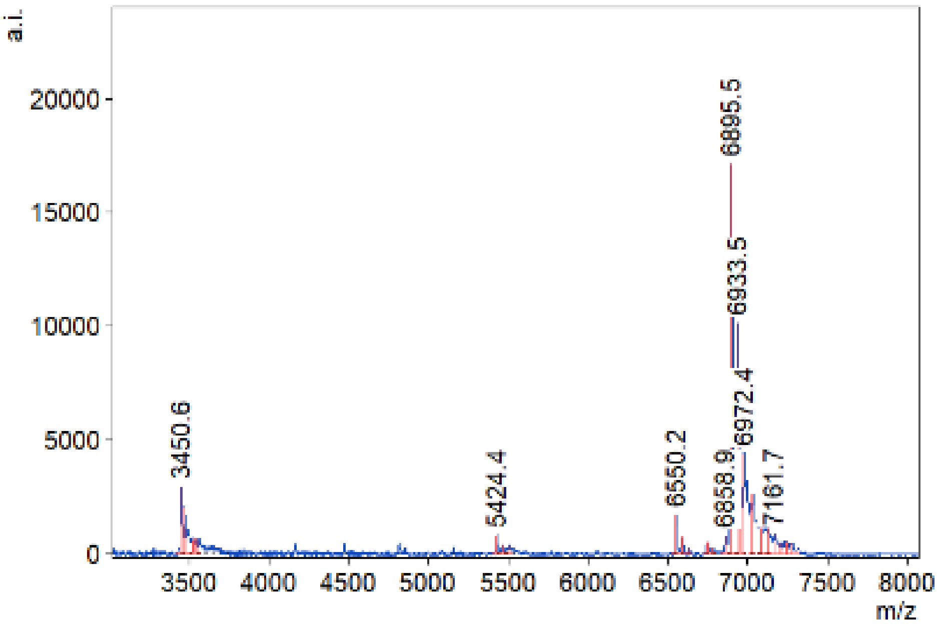

Figure S139. 19RNA_U^{CA}, calculated mass: 6894.3 Da, found mass: 6895.5 Da (product).

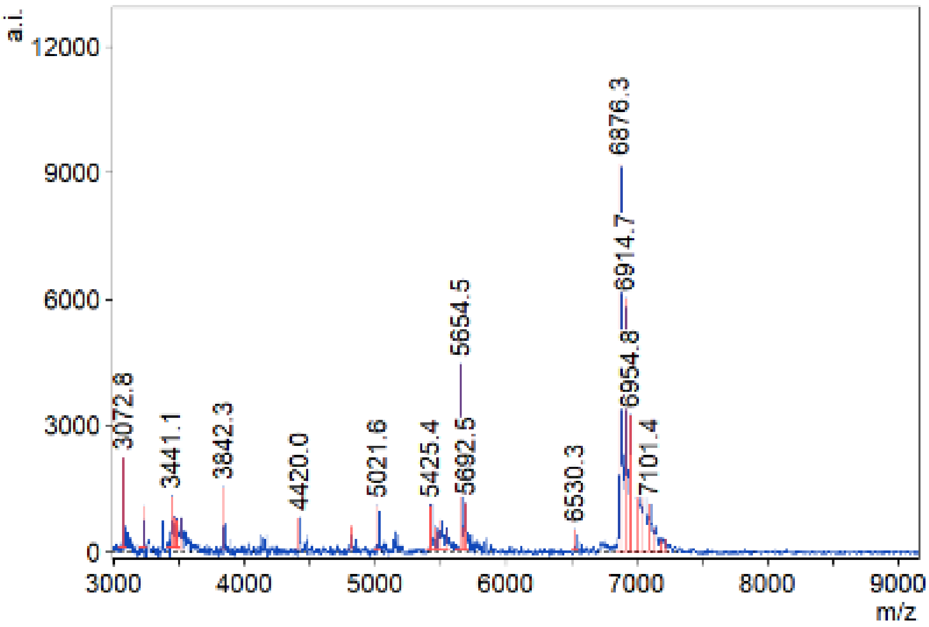

Figure S140. 19RNA_U^{FT}, calculated mass: 6874.8 Da, found mass: 6876.3 Da (product).

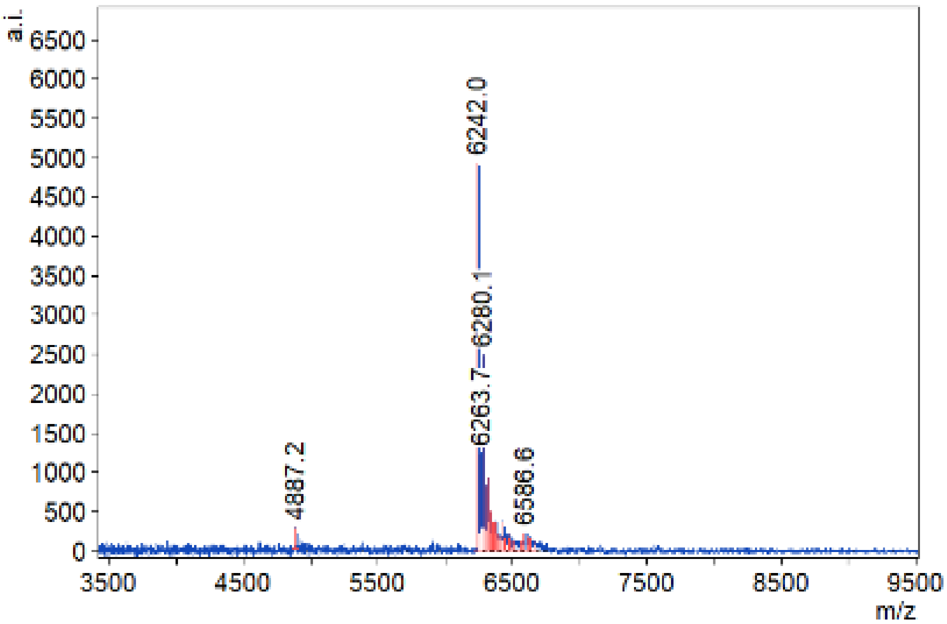

Figure S141. 19RNA_C^{Me}, calculated mass: 6240.8 Da, found mass: 6242.0 Da (product).

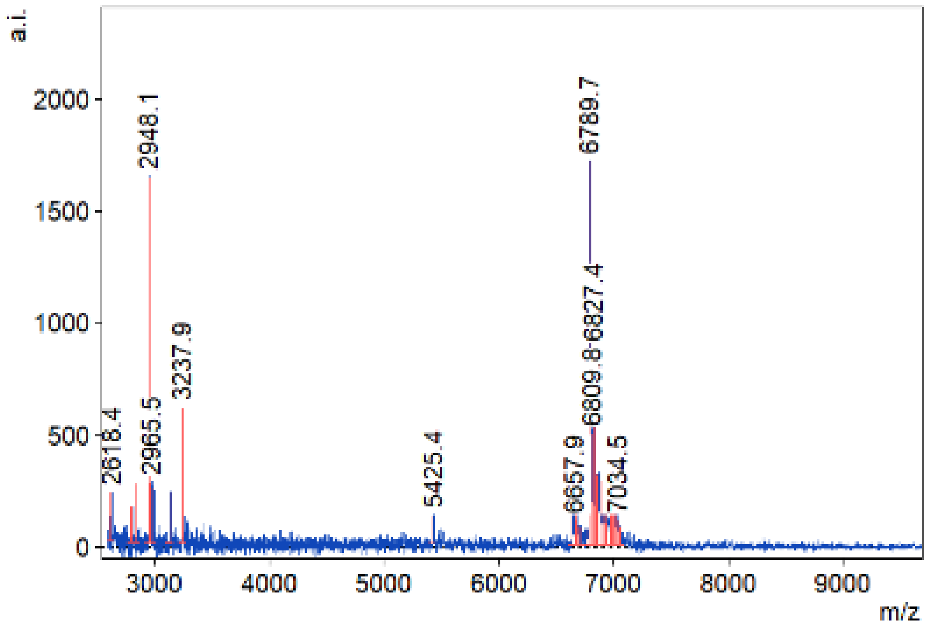

Figure S142. 19RNA_C^{E}, calculated mass: 6787.8 Da, found mass: 6789.7 Da (product).

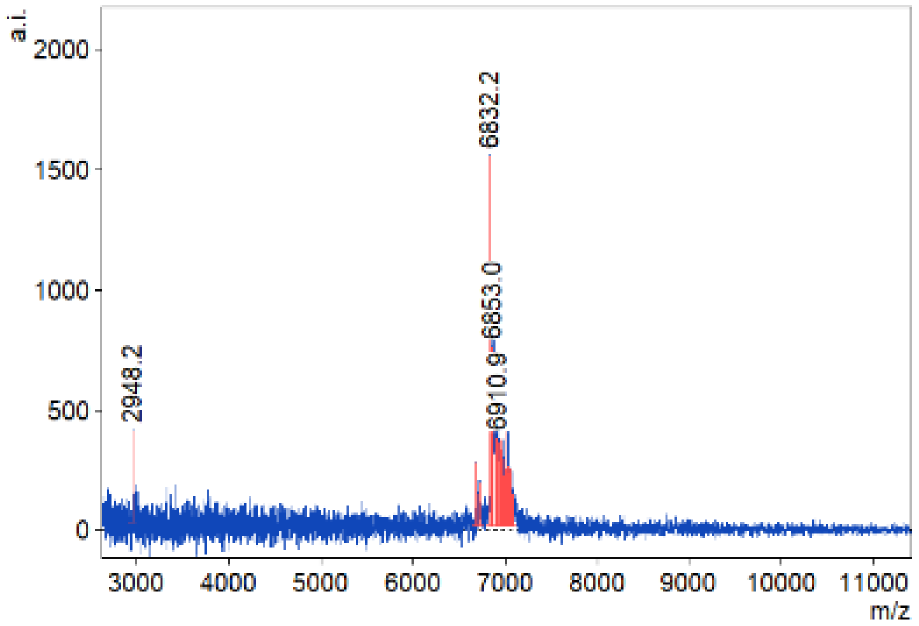

Figure S143. 19RNA_C^{Pent}, calculated mass: 6829.9 Da, found mass: 6832.2 Da (product).

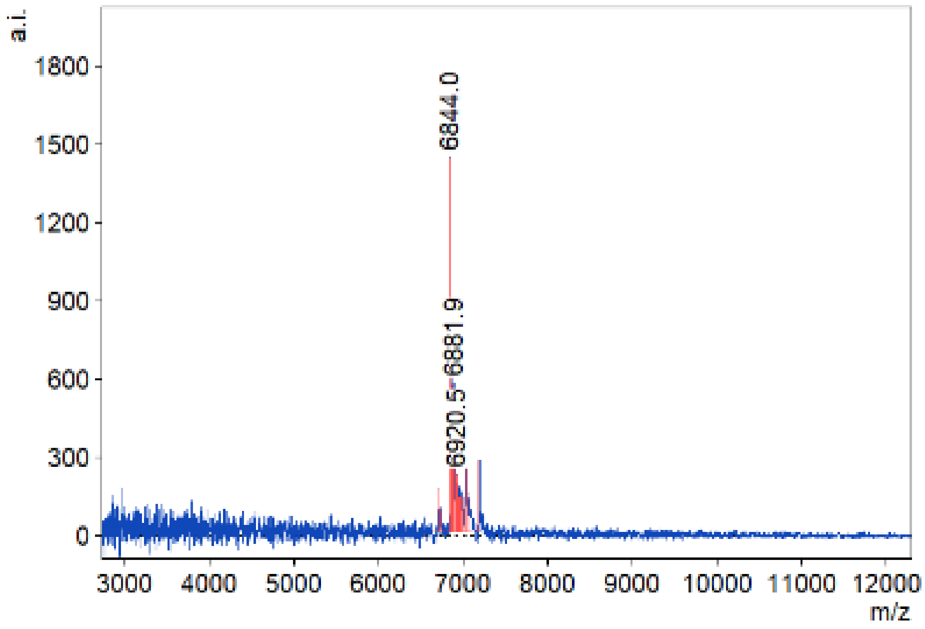

Figure S144. 19RNA_C^{Ph}, calculated mass: 6839.9 Da, found mass: 6844.0 Da (product).

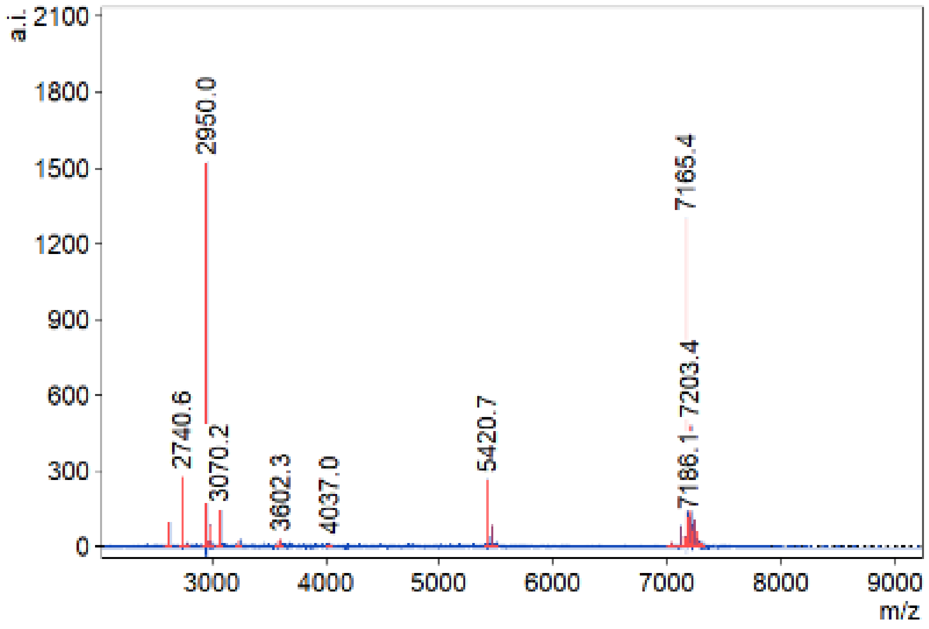

Figure S145. 19RNA_C^{mBdp}, calculated mass: 7164.9 Da, found mass: 7165.4 Da (product).

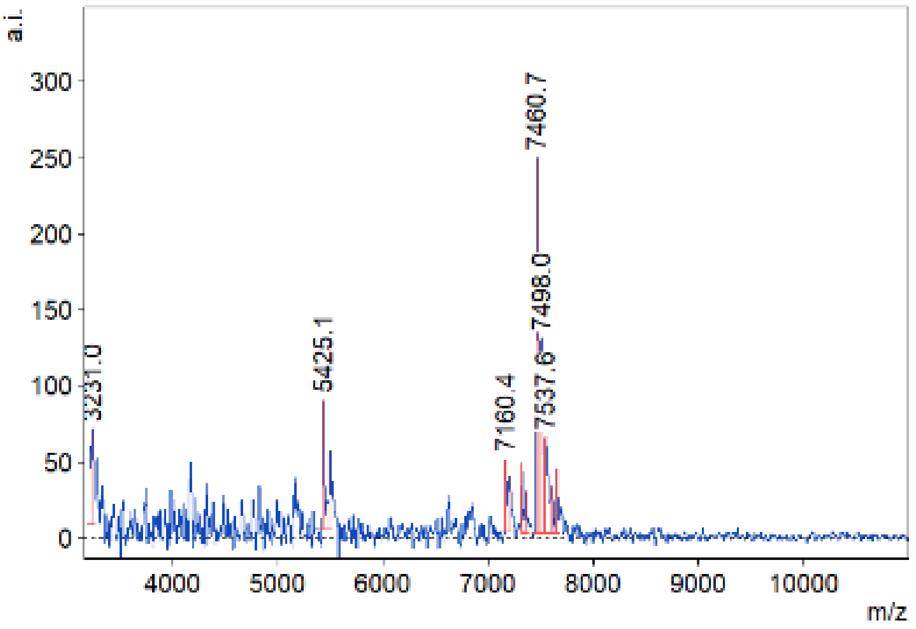

Figure S146. 19RNA_C^{Cy5}, calculated mass: 7455.6 Da, found mass: 7460.7 Da (product).

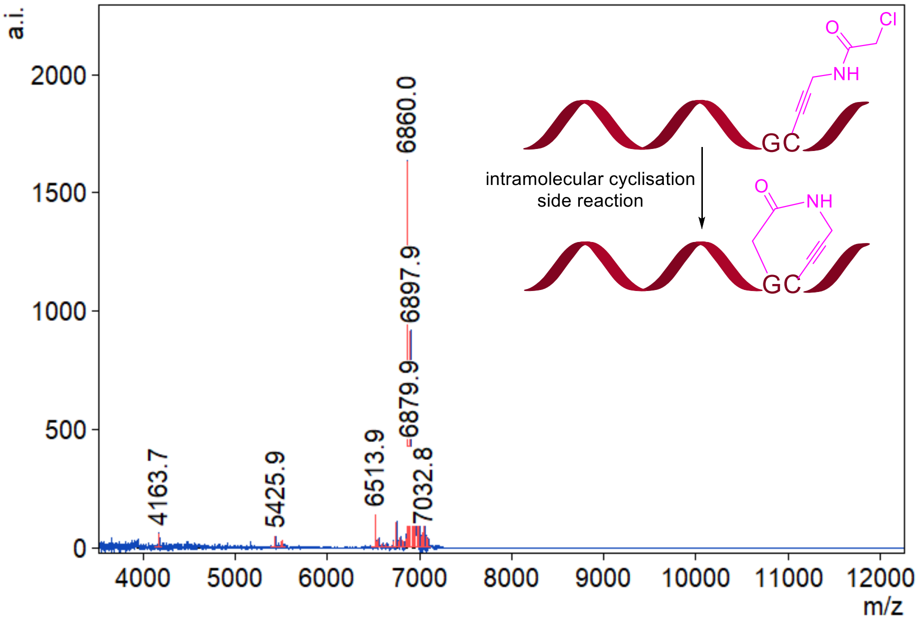

Figure S147. (full spectrum); **19RNA_C^{CA}**, calculated mass: 6893.3 Da, found mass: 6897.9 Da (product); found mass: 6860.0 Da (product of intramolecular cyclisation side reaction = product – HCl).

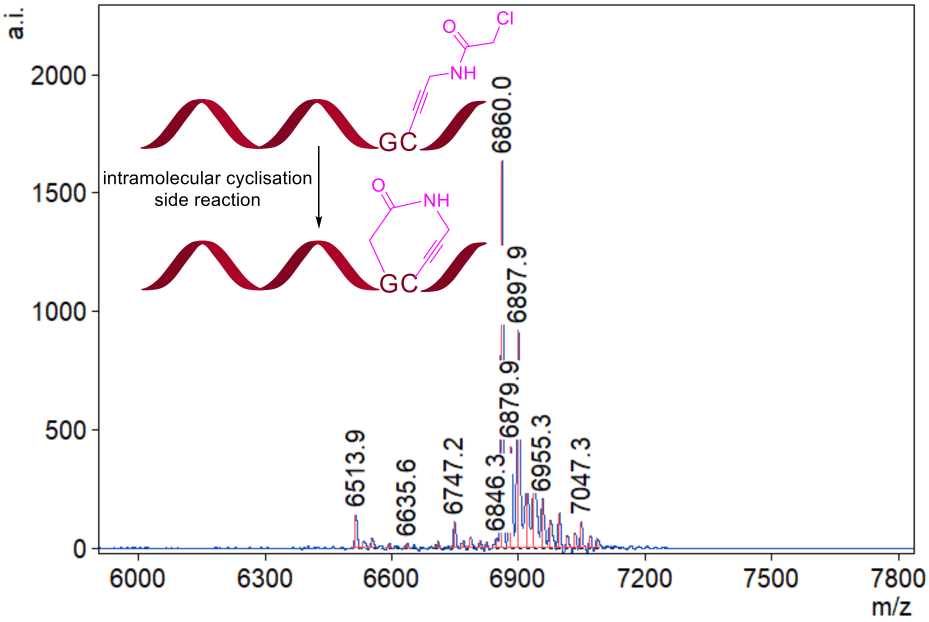

Figure S148. (magnified area of interest); **19RNA_C^{CA}**, calculated mass: 6893.3 Da, found mass: 6897.9 Da (product); found mass: 6860.0 Da (product of intramolecular cyclisation side reaction = product – HCl).

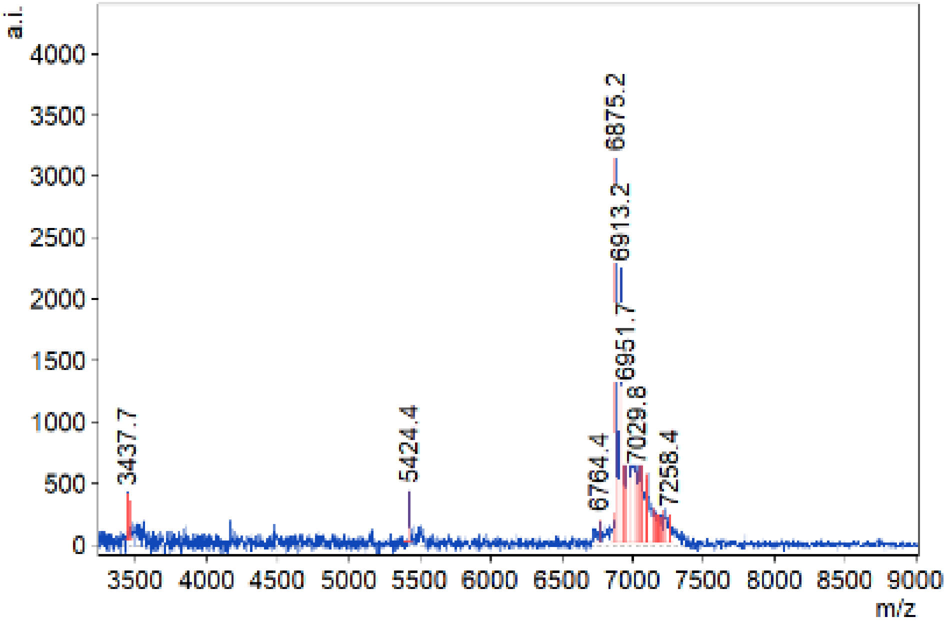

Figure S149. 19RNA_C^{FT}, calculated mass: 6873.9 Da, found mass: 6875.2 Da (product).

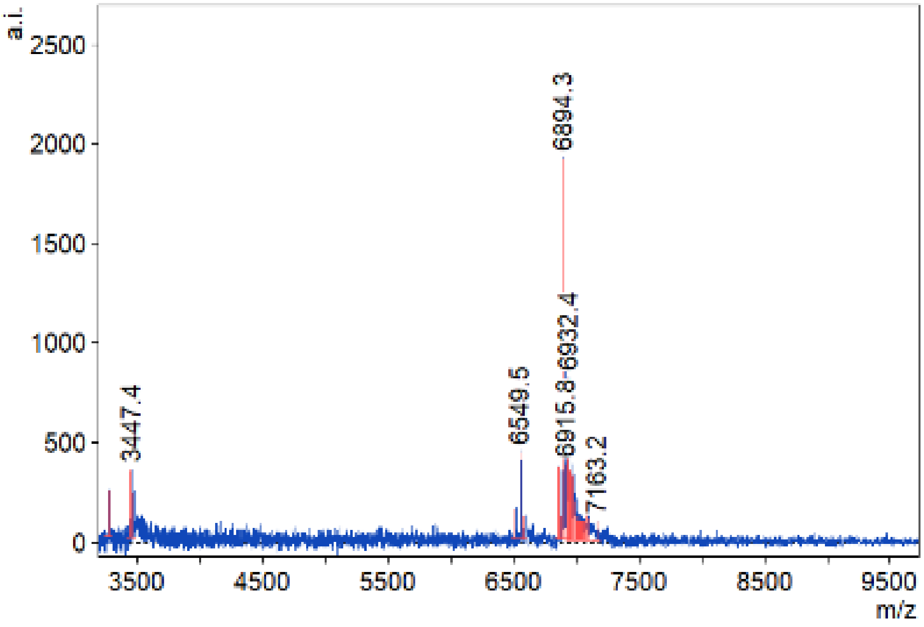

Figure S150. 19RNA_C^{CA}, calculated mass: 6893.3 Da, found mass: 6894.3 Da (product).

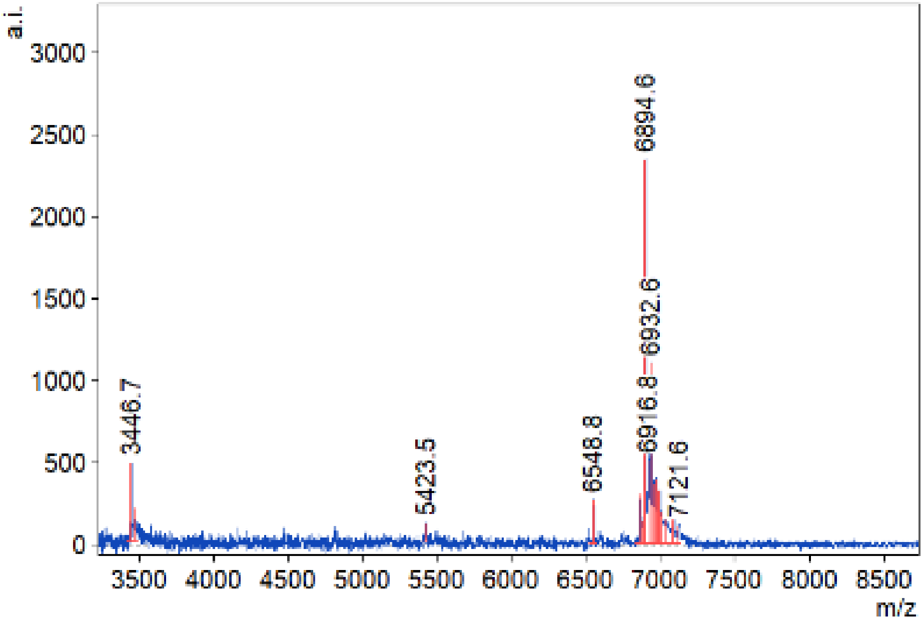

Figure S151. 19RNA_C^{CA}, calculated mass: 6893.3 Da, found mass: 6894.6 Da (product).

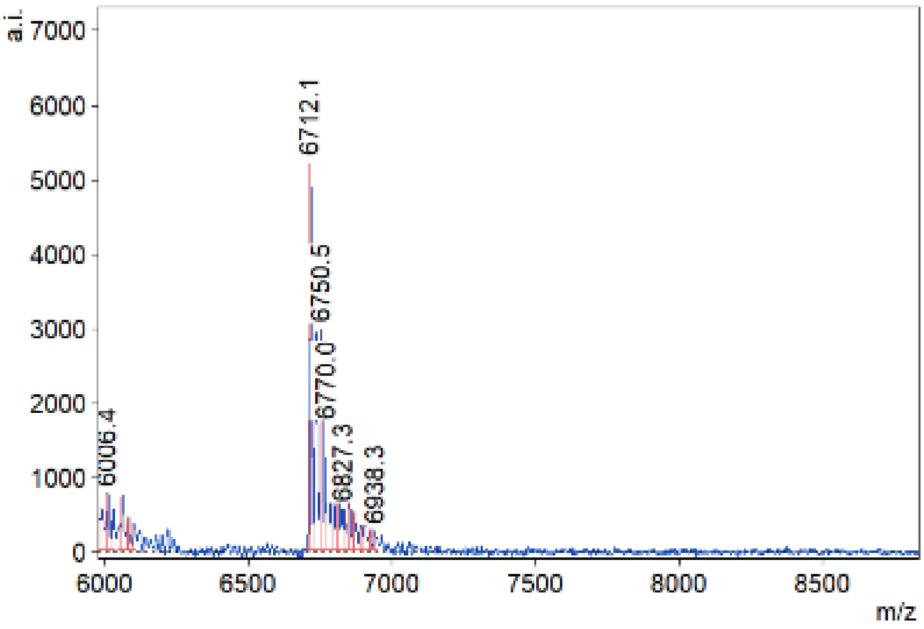

Figure S152. 19RNA_G^{E}, calculated mass: 6709.7 Da, found mass: 6712.1 Da (product).

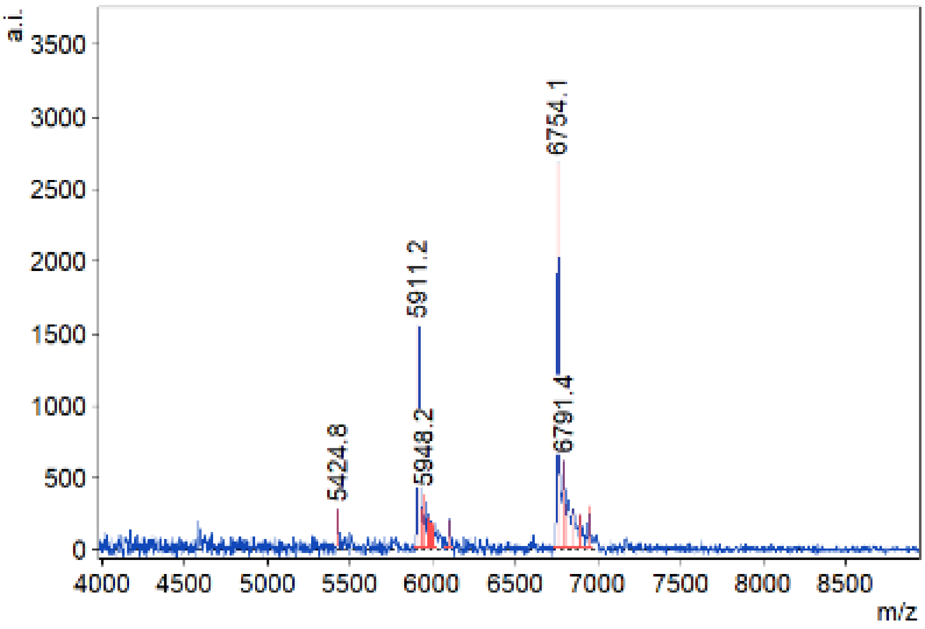

Figure S153. 19RNA_G^{Pent}, calculated mass: 6751.8 Da, found mass: 6754.1 Da (product).

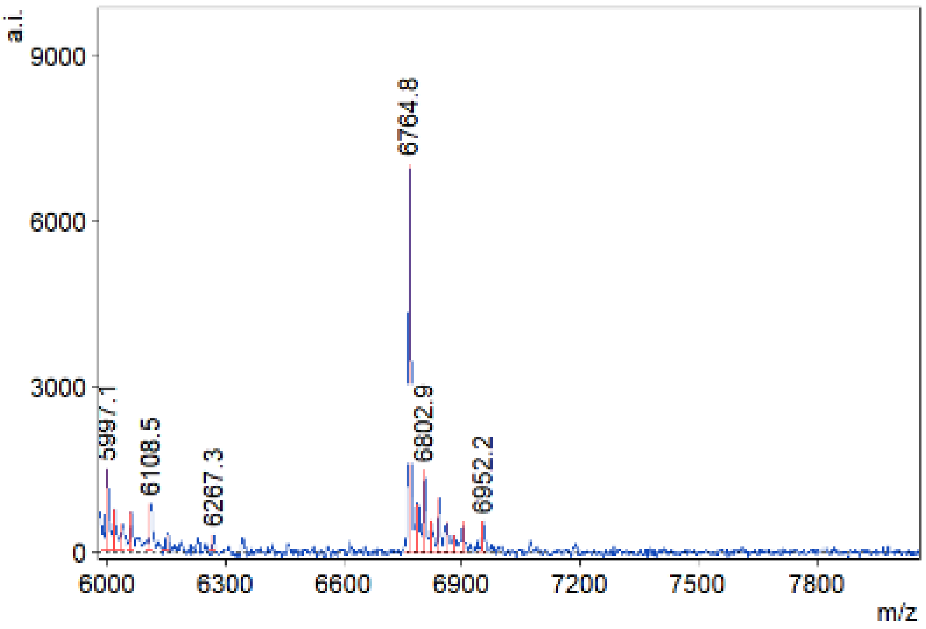

Figure S154. 19RNA_G^{Ph}, calculated mass: 6761.8 Da, found mass: 6764.8 Da (product).

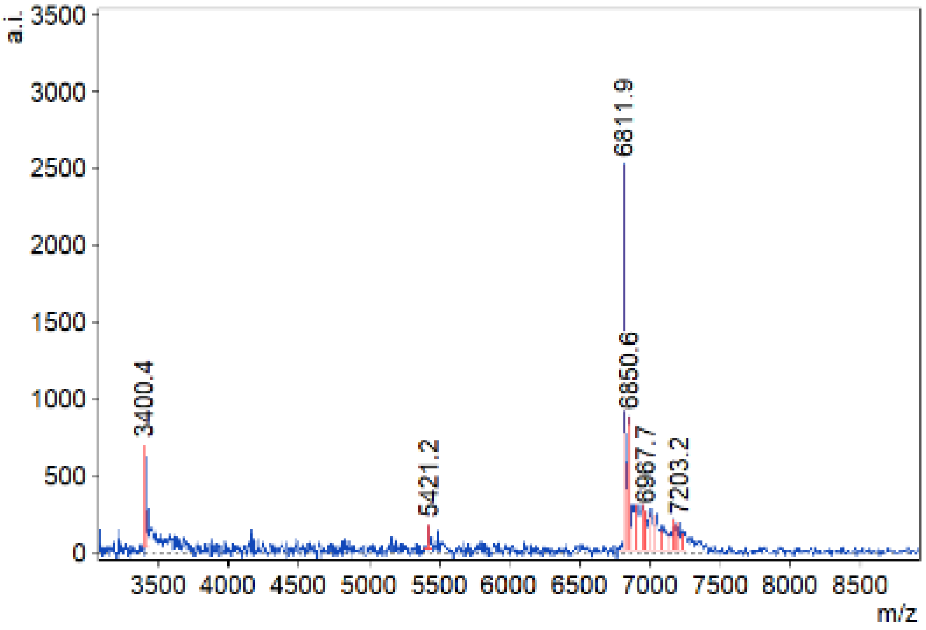

Figure S155. 19RNA_A^{E}, calculated mass: 6810.8 Da, found mass: 6811.9 Da (product).

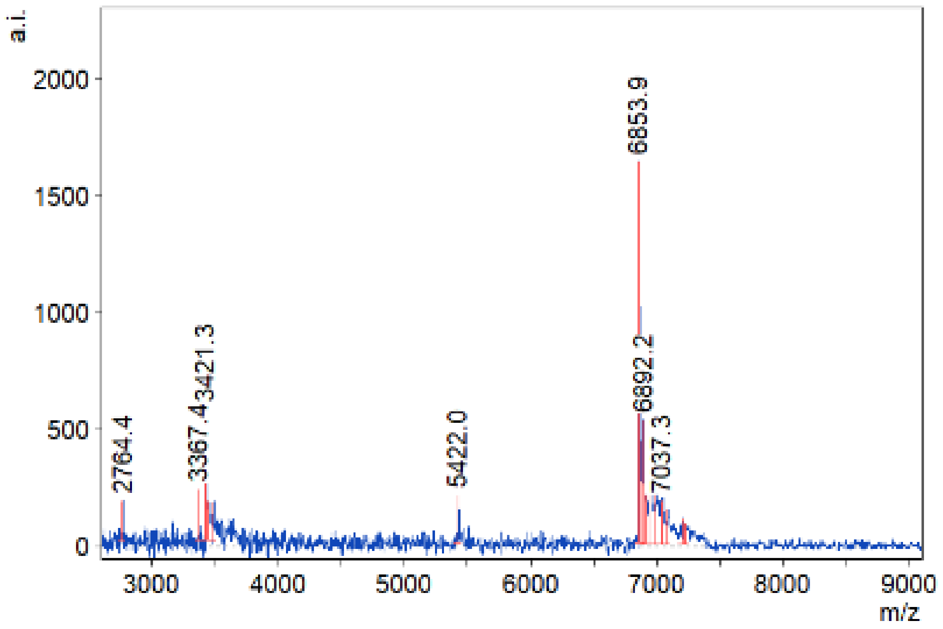

Figure S156. 19RNA_A^{Pent}, calculated mass: 6852.9 Da, found mass: 6853.9 Da (product).

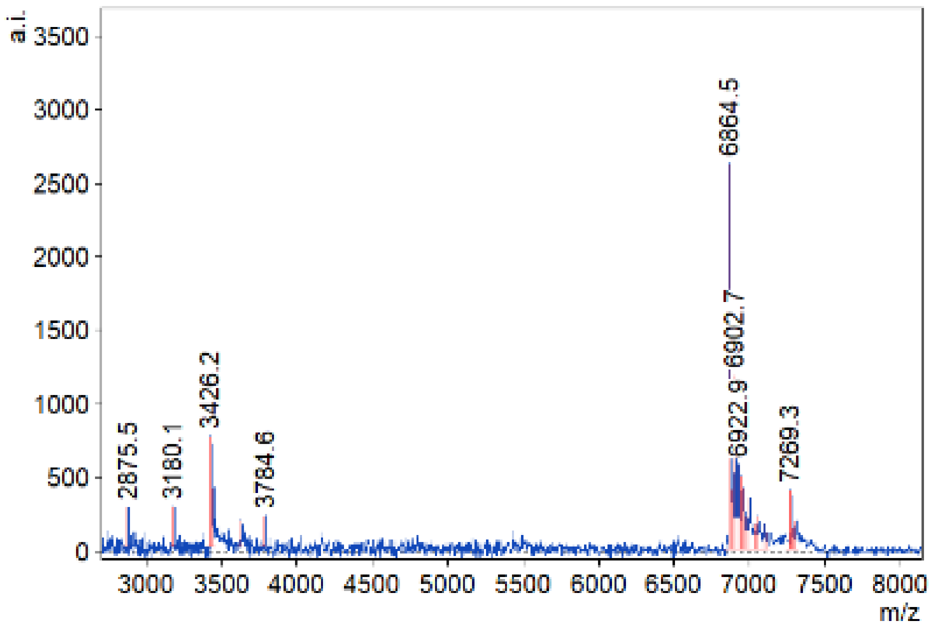

Figure S157. 19RNA_A^{Ph}, calculated mass: 6862.9 Da, found mass: 6864.5 Da (product).

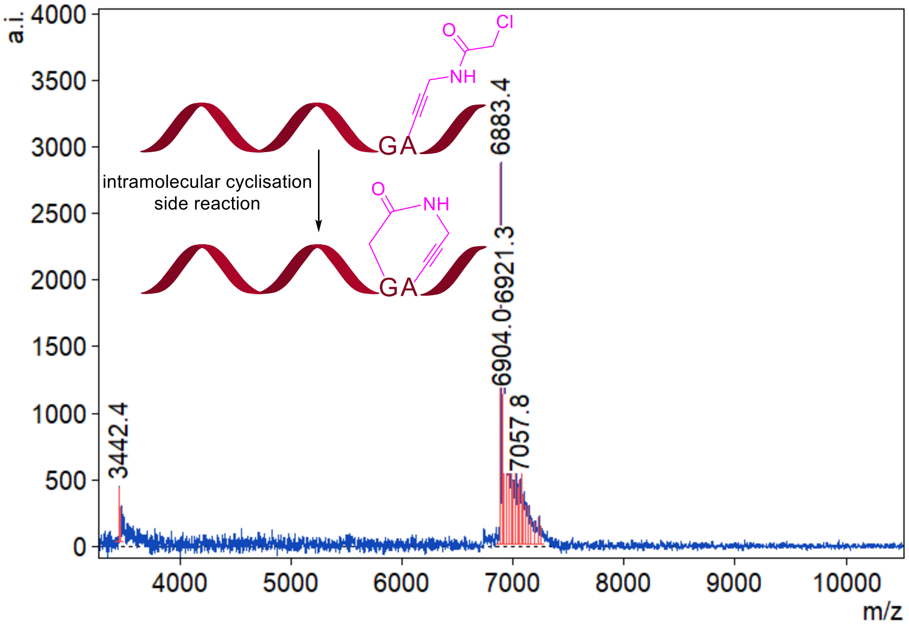

Figure S158. (full spectrum); **19RNA_A**$^{\mathbf{CA}}$, calculated mass: 6915.6 Da, found mass: 6921.3 (product); found mass: 6883.4 Da (product of intramolecular cyclisation side reaction = product – HCl).

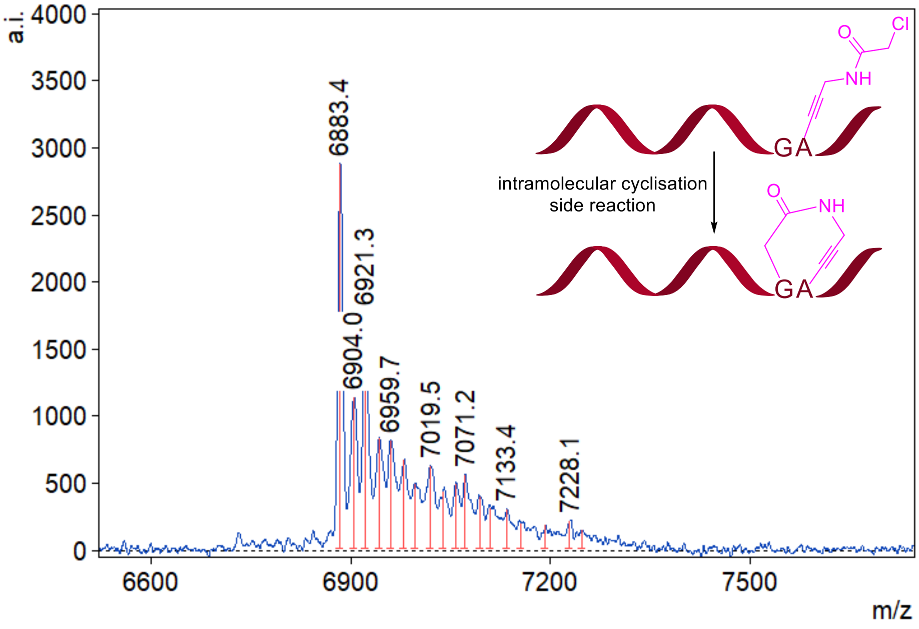

Figure S159. (magnified area of interest); **19RNA_A**$^{\mathbf{CA}}$, calculated mass: 6915.6 Da, found mass: 6921.3 (product); found mass: 6883.4 Da (product of intramolecular cyclisation side reaction = product – HCl).

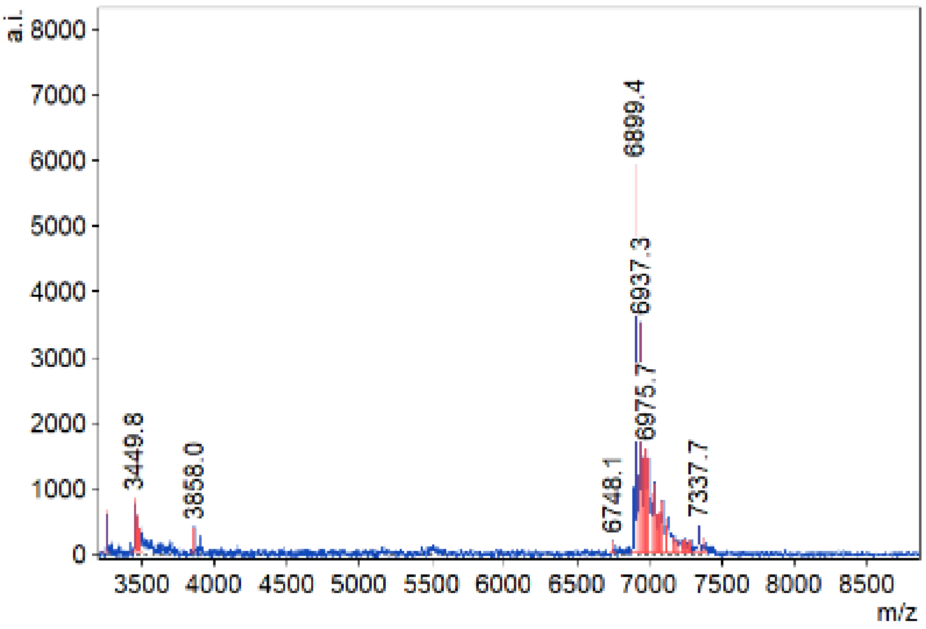

Figure S160. 19RNA_A^{FT}, calculated mass: 6896.9 Da, found mass: 6899.4 Da (product).

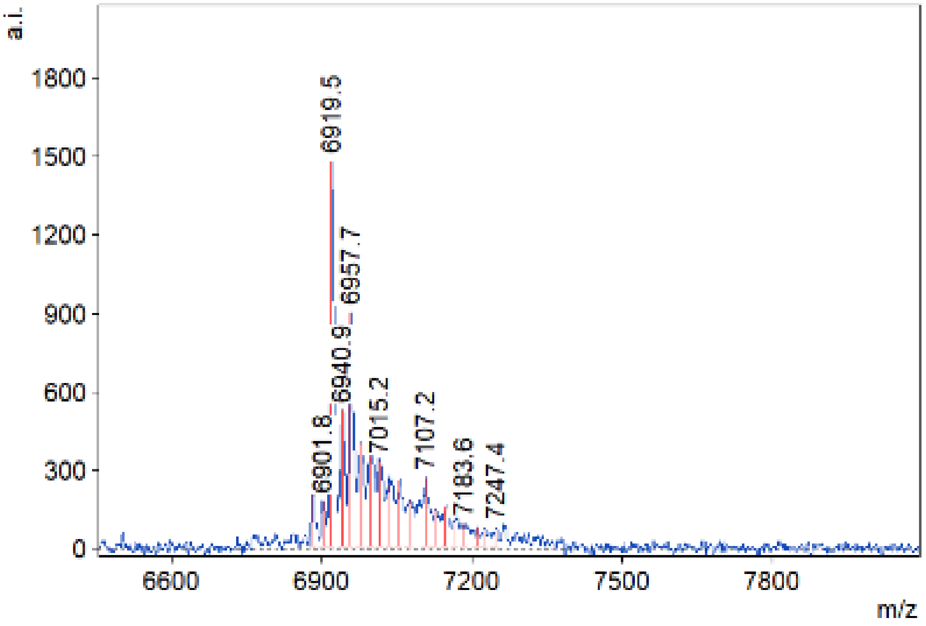

Figure S161. 19RNA_A^{CA}, calculated mass: 6915.6 Da, found mass: 6919.5 Da (product).

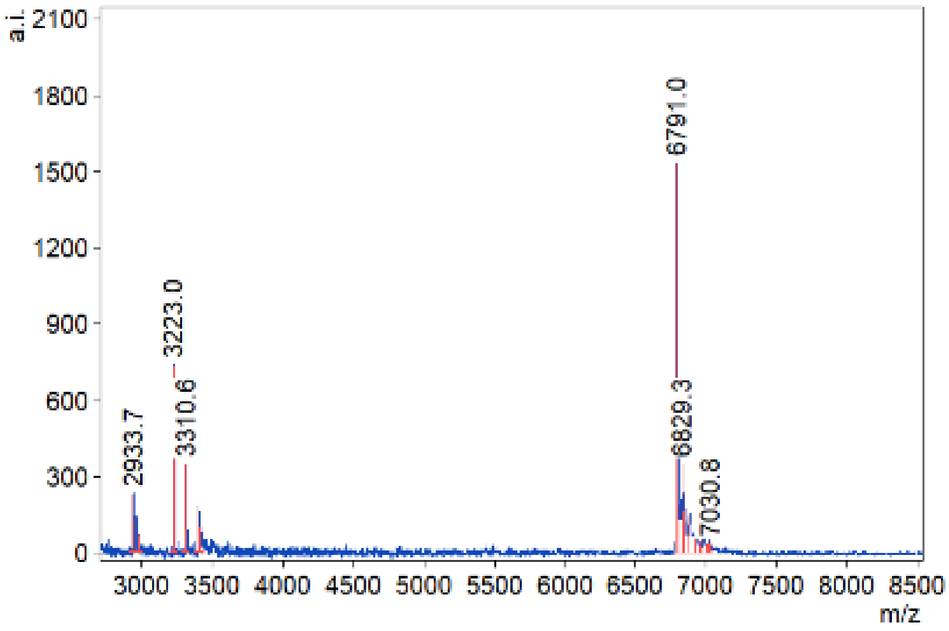

Figure S162. 19RNA_U$^{\mathbf{E}}$, calculated mass: 6788.7 Da, found mass: 6791.0 Da (product).

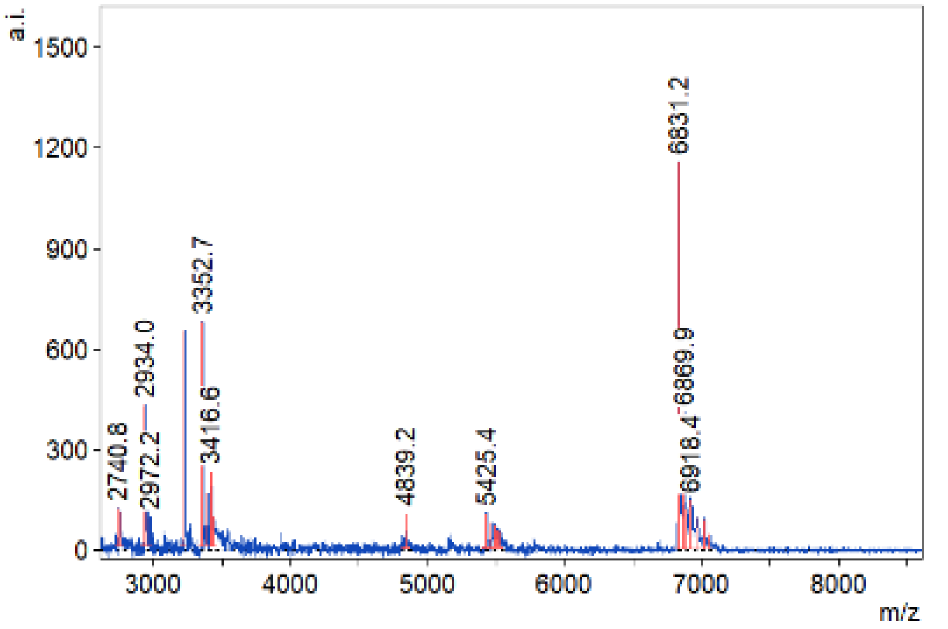

Figure S163. 19RNA_U$^{\mathbf{Pent}}$, calculated mass: 6830.8 Da, found mass: 6831.2 Da (product).

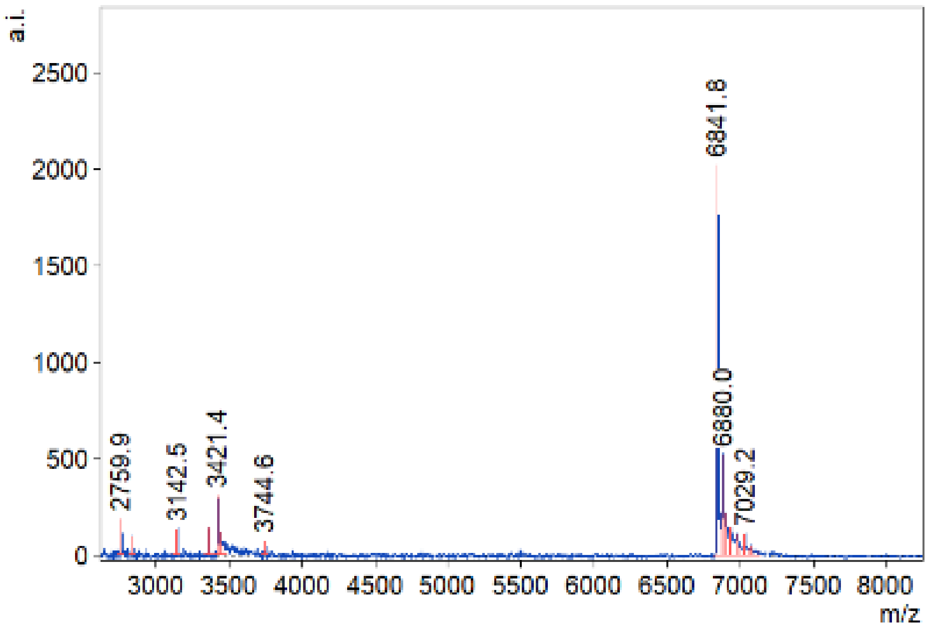

Figure S164. 19RNA_U^{Ph}, calculated mass: 6840.8 Da, found mass: 6841.8 Da (product).

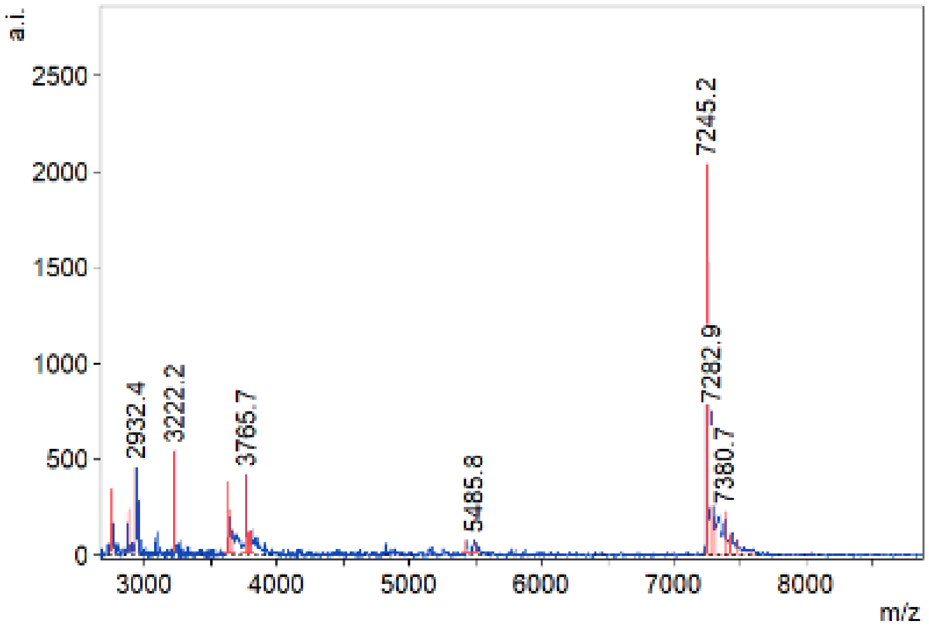

Figure S165. 19RNA_U^{Bio}, calculated mass: 7244.3 Da, found mass: 7245.2 Da (product).

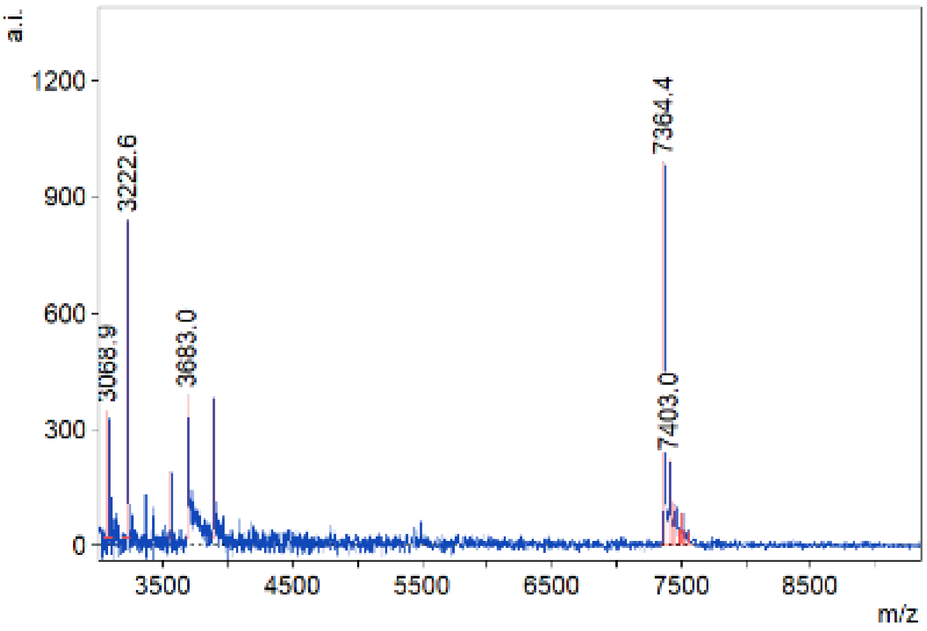

Figure S166. 19RNA_U^{Dig}, calculated mass: 7363.5 Da, found mass: 7364.4 Da (product).

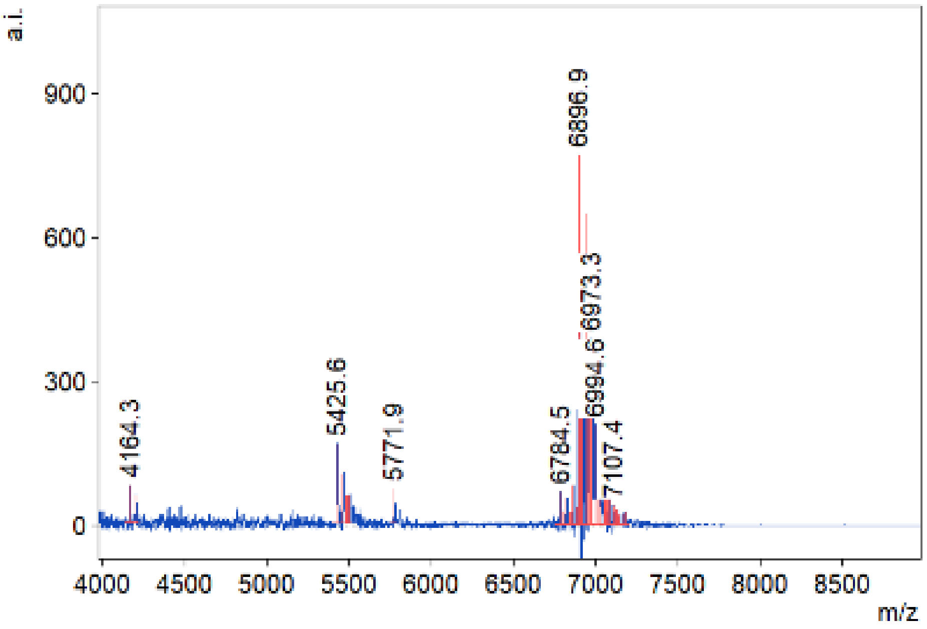

Figure S167. 19RNA_U^{CA}, calculated mass: 6894.3 Da, found mass: 6896.9 Da (product).

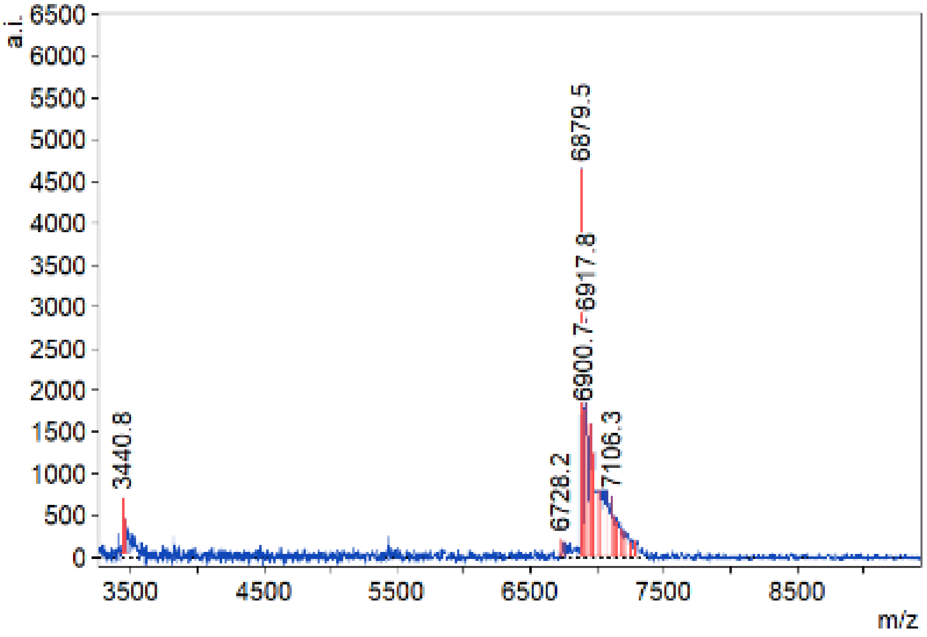

Figure S168. 19RNA_U^{FT}, calculated mass: 6874.8 Da, found mass: 6879.5 Da (product).

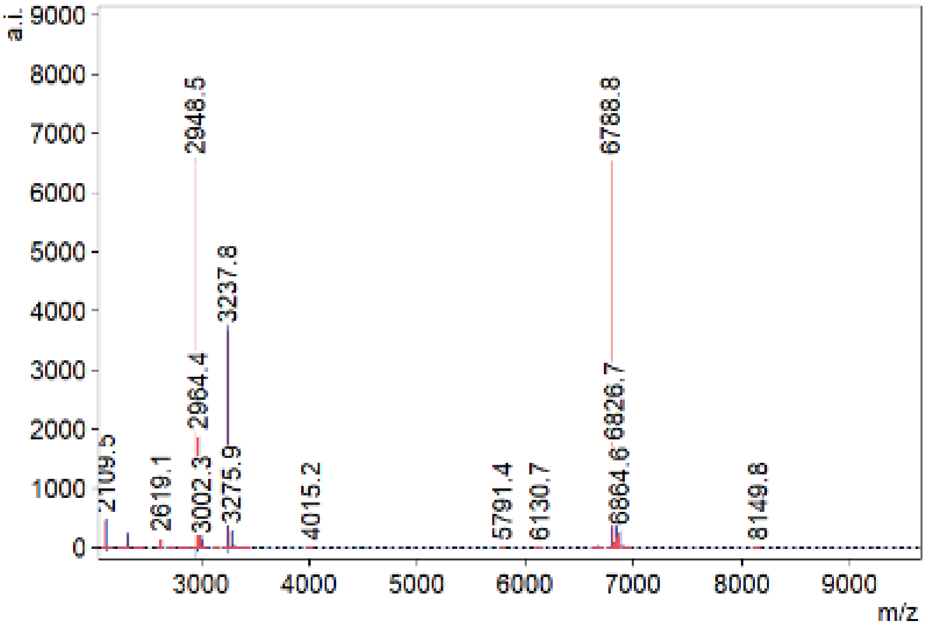

Figure S169. 19RNA_C^{E}, calculated mass: 6787.8 Da, found mass: 6788.8 Da (product).

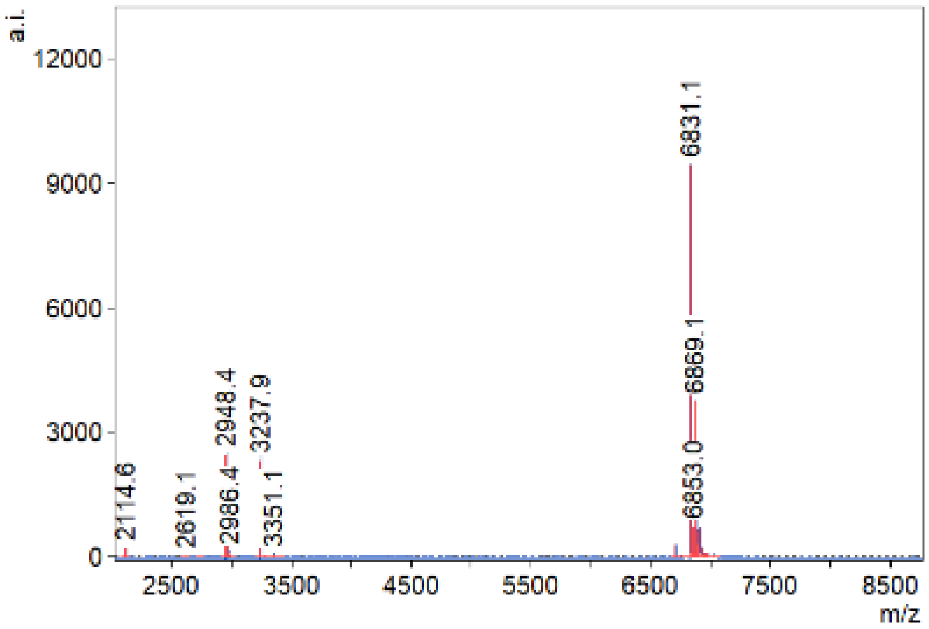

Figure S170. 19RNA_C^{Pent}, calculated mass: 6829.9 Da, found mass: 6831.1 Da (product).

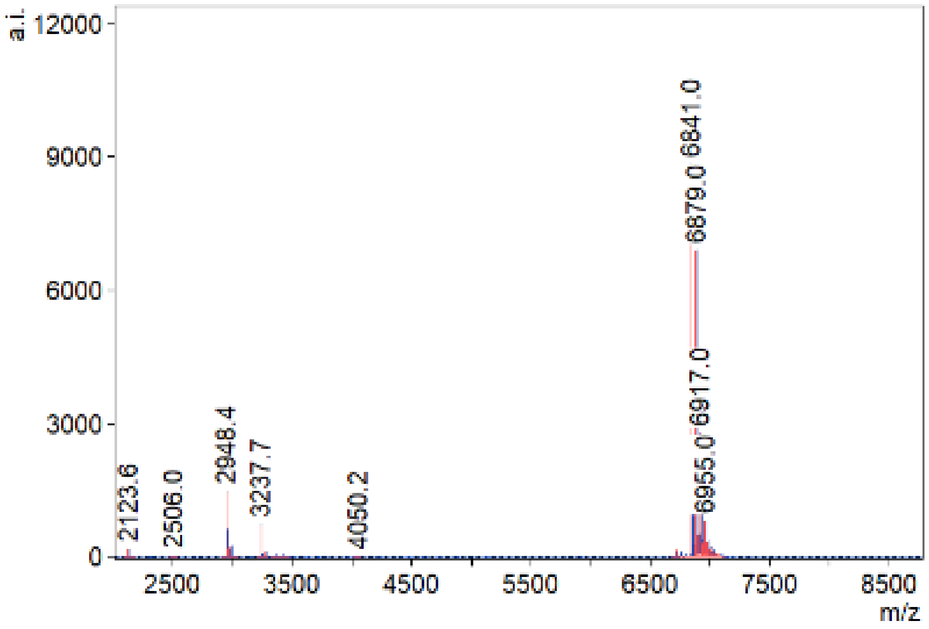

Figure S171. 19RNA_C^{Ph}, calculated mass: 6839.9 Da, found mass: 6841.0 Da (product).

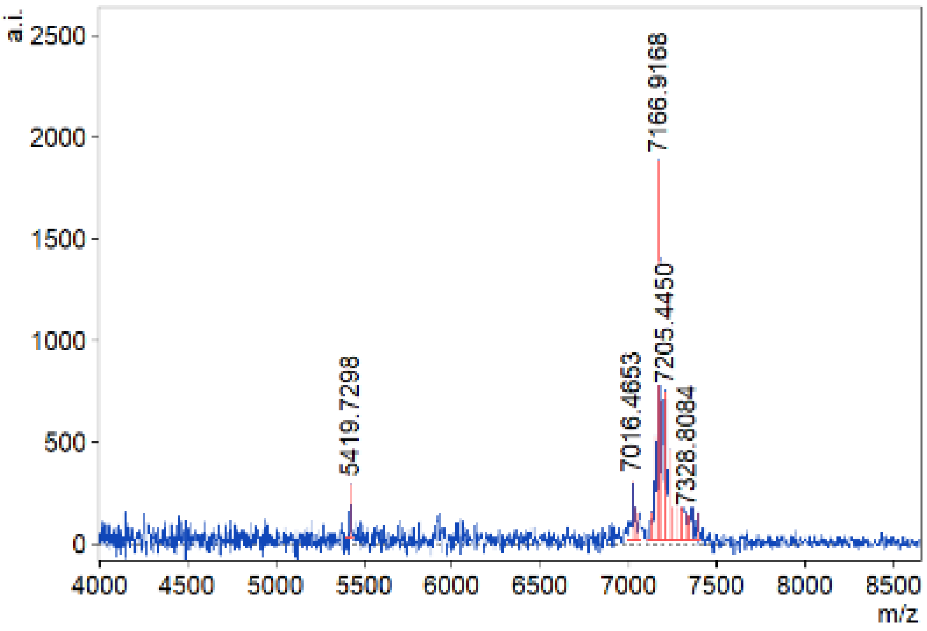

Figure S172. 19RNA_C^{mBdp}, calculated mass: 7164.9 Da, found mass: 7166.9 Da (product).

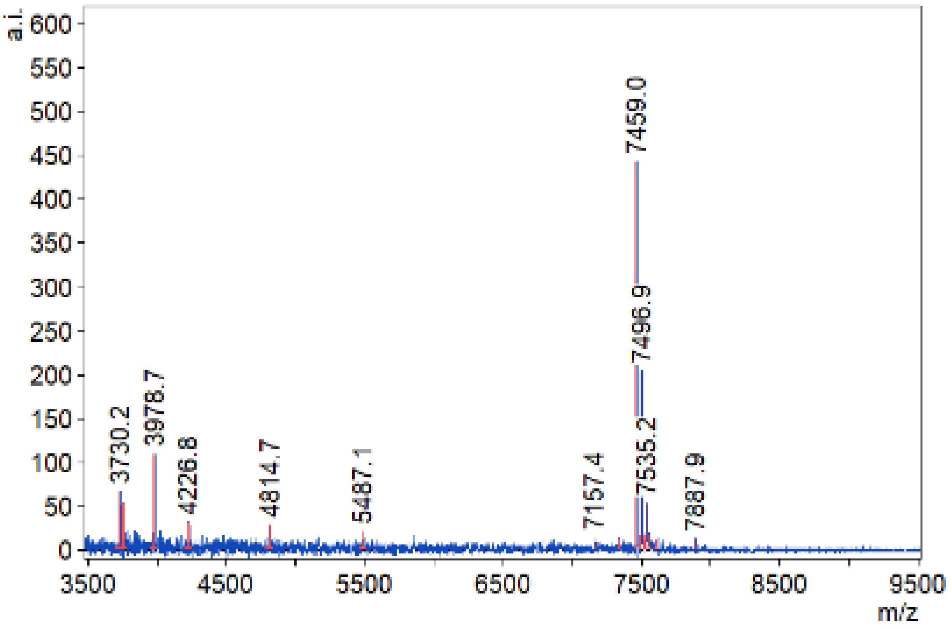

Figure S173. 19RNA_C^{Cy5}, calculated mass: 7455.7 Da, found mass: 7459.0 Da (product).

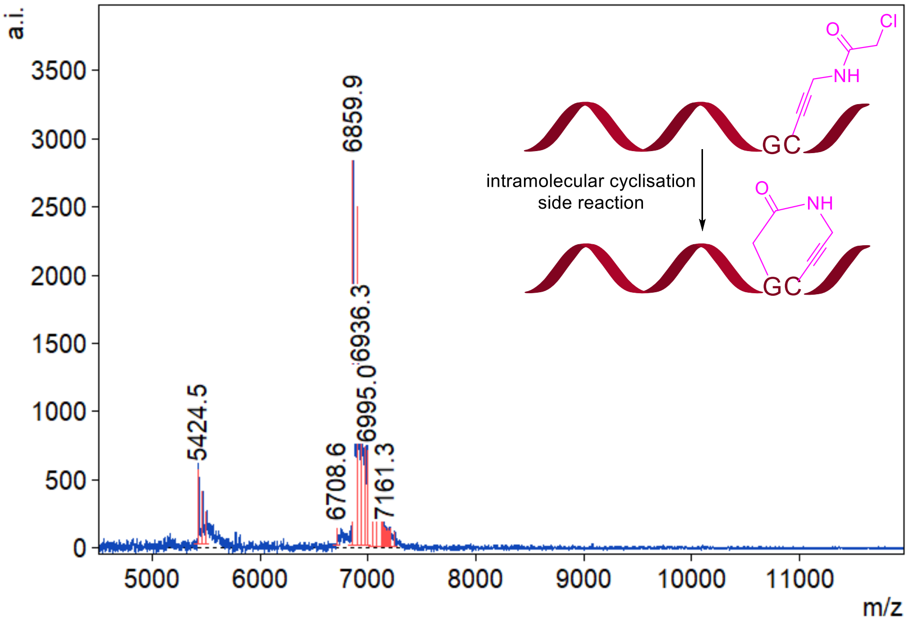

Figure S174. (full spectrum); **19RNA_C^{CA}**, calculated mass: 6893.3 Da, found mass: 6897.8 Da (product); found mass: 6859.9 Da (product of intramolecular cyclisation side reaction = product – HCl).

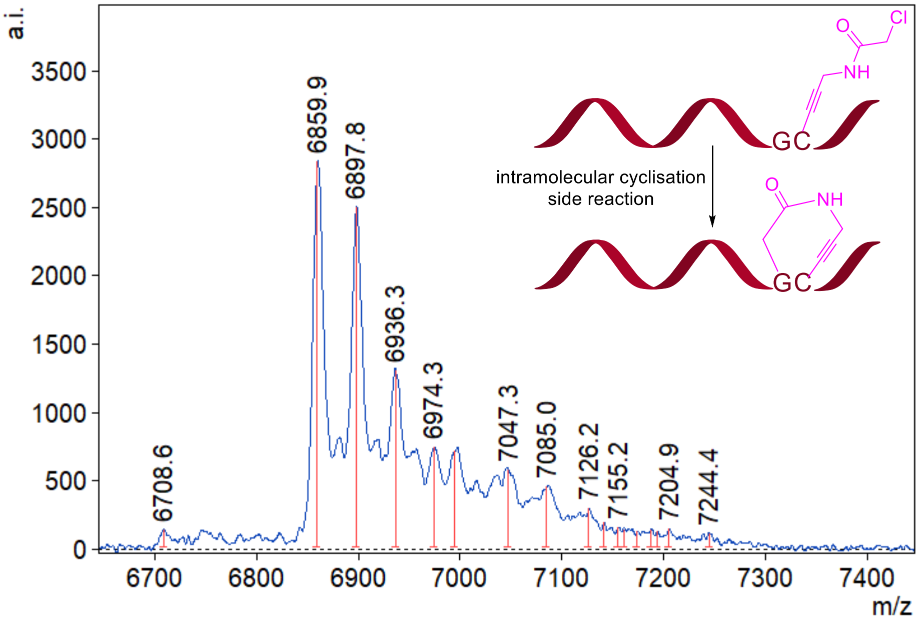

Figure S175. (magnified area of interest); **19RNA_C^{CA}**, calculated mass: 6893.3 Da, found mass: 6897.8 Da (product); found mass: 6859.9 Da (product of intramolecular cyclisation side reaction = product – HCl).

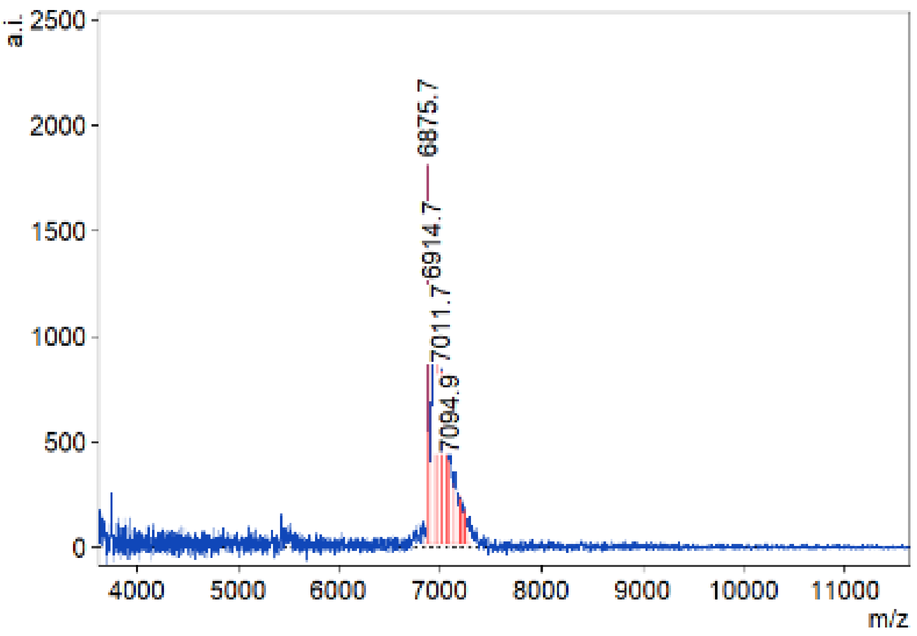

Figure S176. 19RNA_C^{FT}, calculated mass: 6873.9 Da, found mass: 6875.7 Da (product).

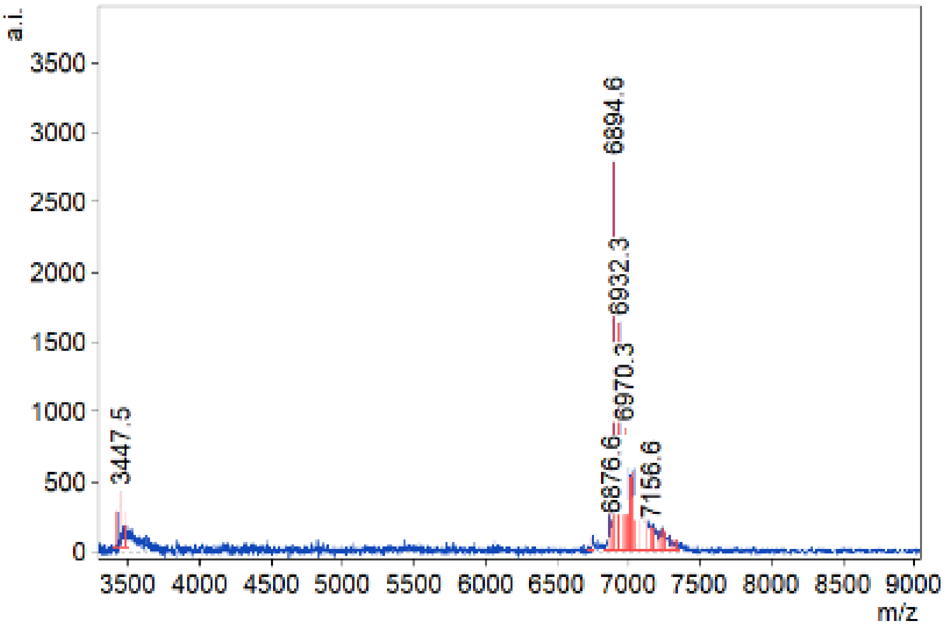

Figure S177. 19RNA_C^{CA}, calculated mass: 6893.3 Da, found mass: 6894.6 Da (product).

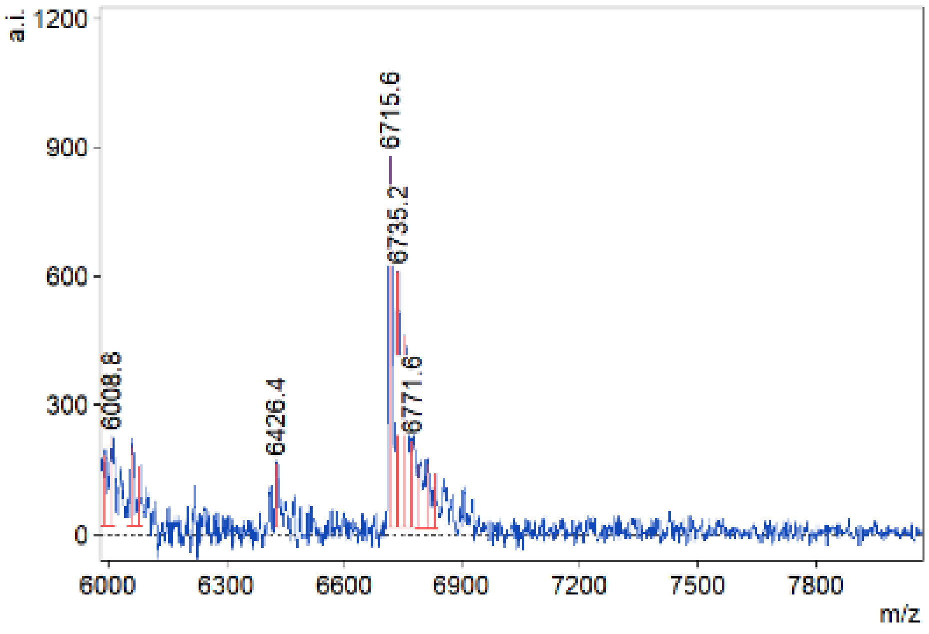

Figure S178. 19RNA_G^{E}, calculated mass: 6709.7 Da, found mass: 6715.6 Da (product).

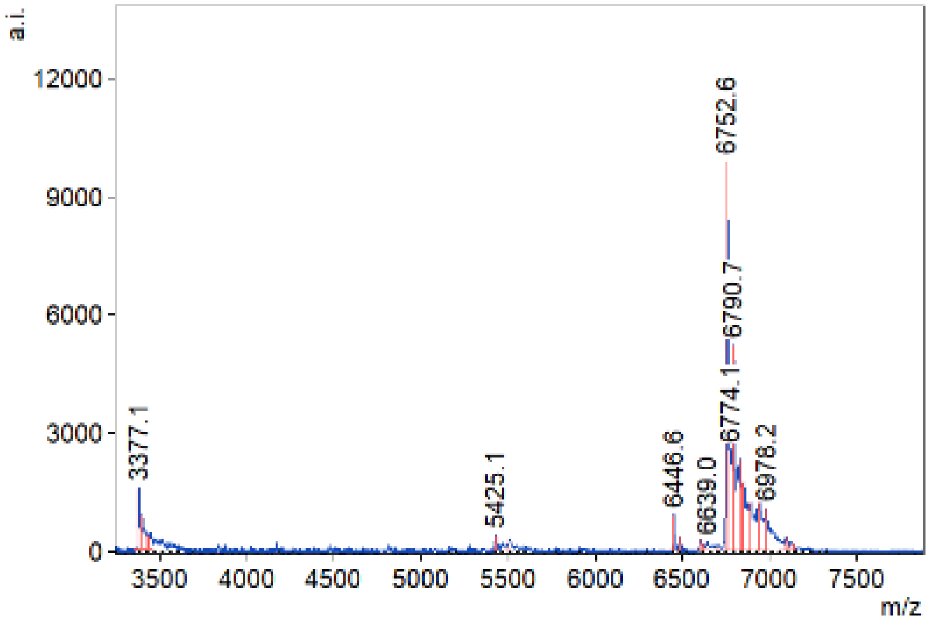

Figure S179. 19RNA_G^{Pent}, calculated mass: 6751.8 Da, found mass: 6752.6 Da (product).

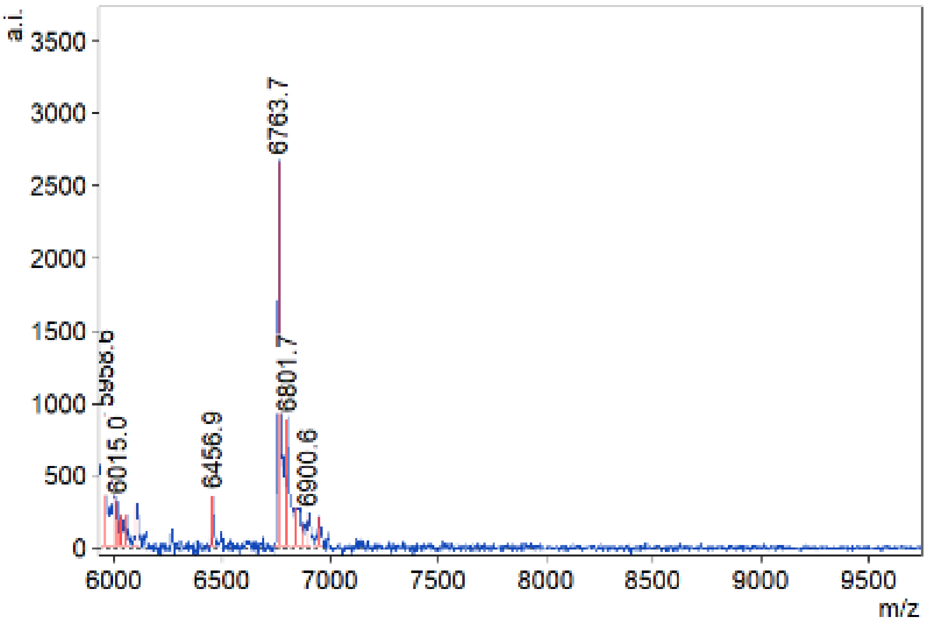

Figure S180. 19RNA_G^{Ph}, calculated mass: 6761.8 Da, found mass: 6763.7 Da (product).

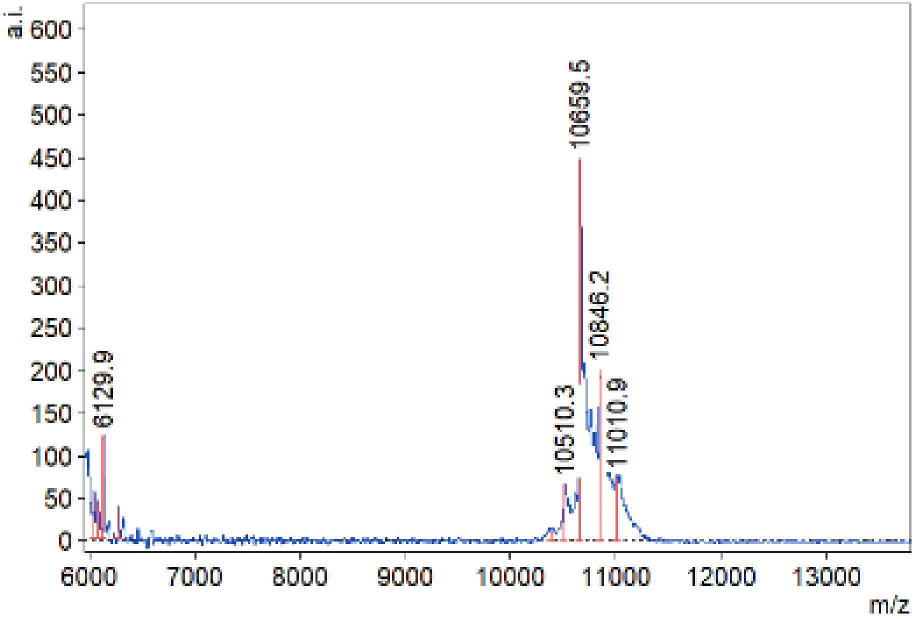

Figure S181. 31RNA_4A^{E}, calculated mass: 10658.1 Da, found mass: 10659.5 Da (product).

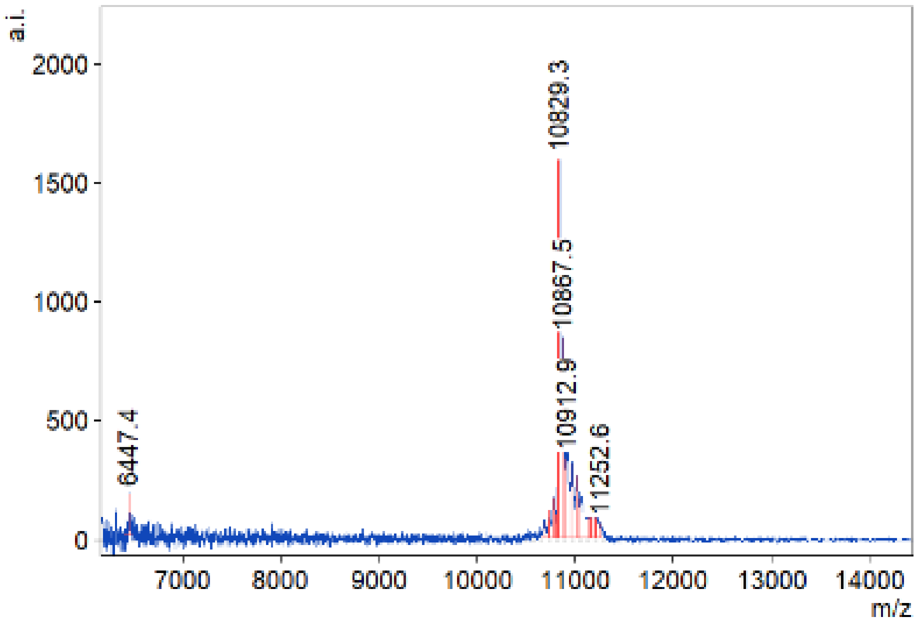

Figure S182. 31RNA_4A^{Pent}, calculated mass: 10826.4 Da, found mass: 10829.3 Da (product).

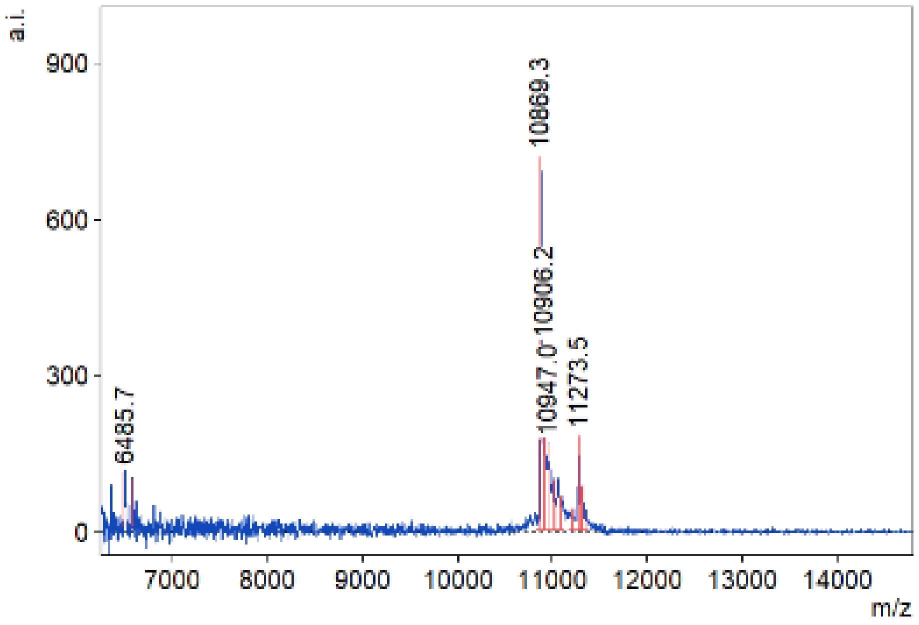

Figure S183. 31RNA_4A^{Ph}, calculated mass: 10866.4 Da, found mass: 10869.3 Da (product).

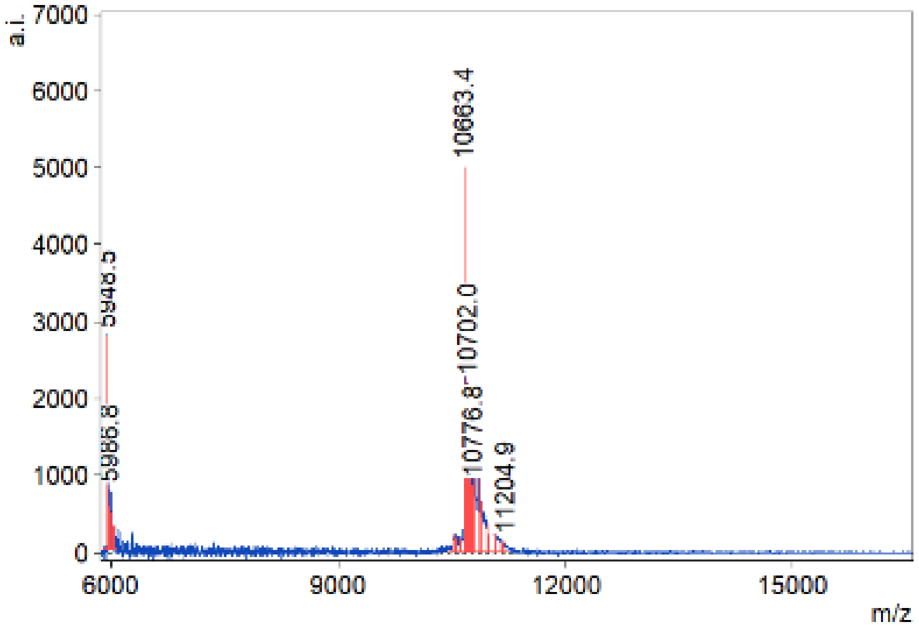

Figure S184. 31RNA_4U^{E}, calculated mass: 10662.1 Da, found mass: 10663.4 Da (product).

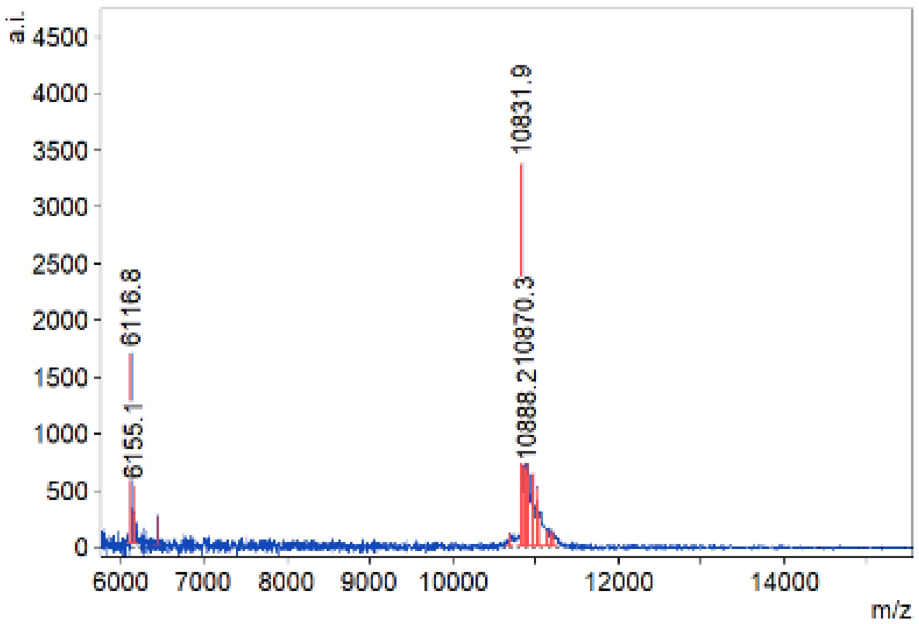

Figure S185. 31RNA_4U^{Pent}, calculated mass: 10830.4 Da, found mass: 10831.9 Da (product).

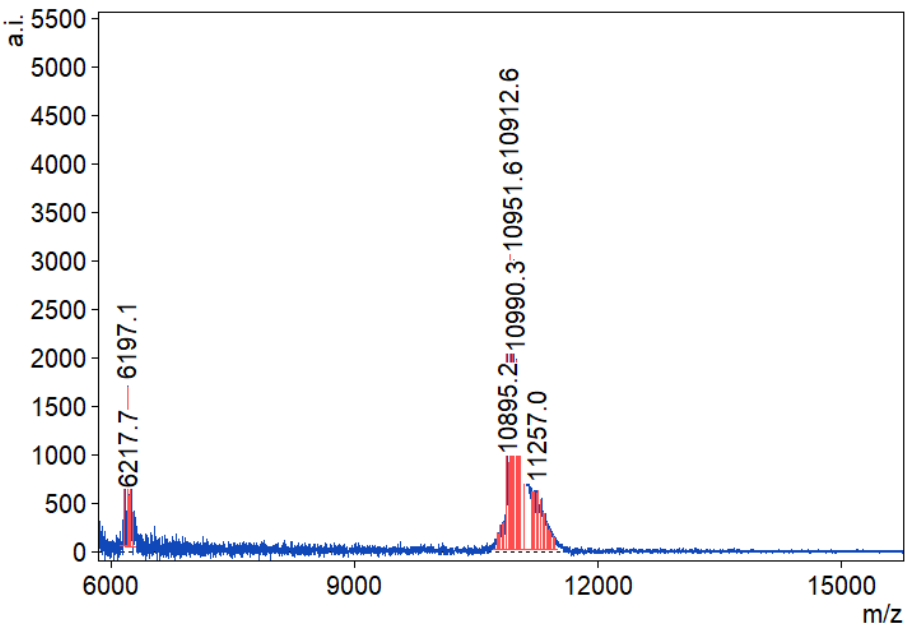

Figure S186. (full spectrum); **31RNA_4U^{Ph}**, calculated mass: 10870.4 Da, found mass: 10873.3 Da (product); found mass: 10912.6 (product + K^+); found mass: 10951.6 Da (product + $2K^+$).

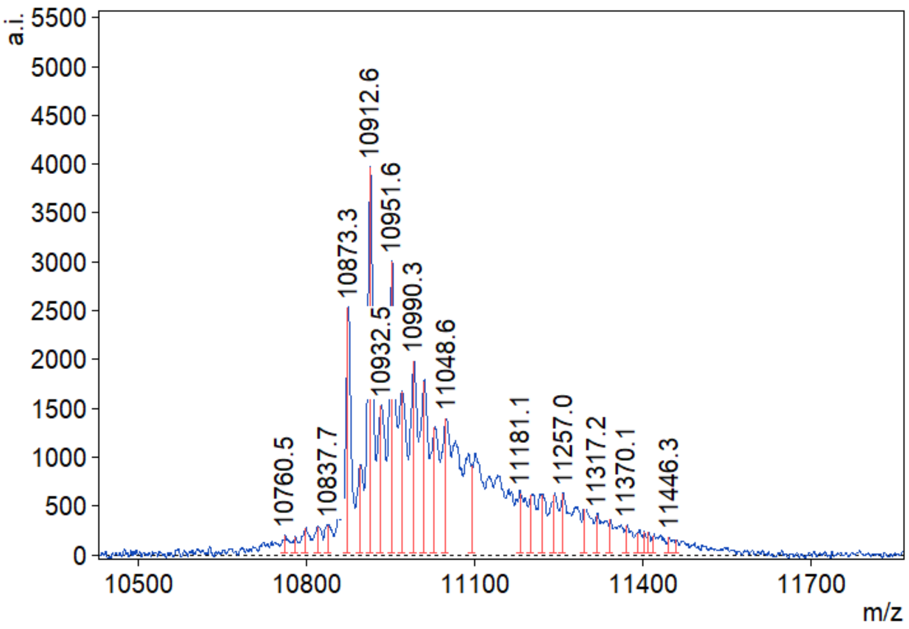

Figure S187. (magnified area of interest); **31RNA_4U^{Ph}**, calculated mass: 10870.4 Da, found mass: 10873.3 Da (product); found mass: 10912.6 (product + K^+); found mass: 10951.6 Da (product + $2K^+$).

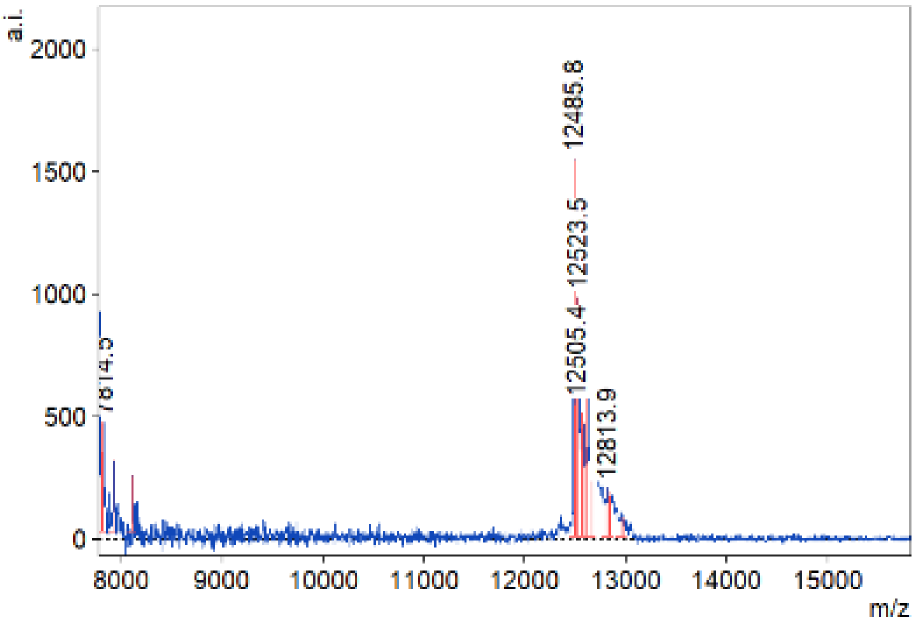

Figure S188. 31RNA_4U^{Bio}, calculated mass: 12484.6 Da, found mass: 12485.8 Da (product).

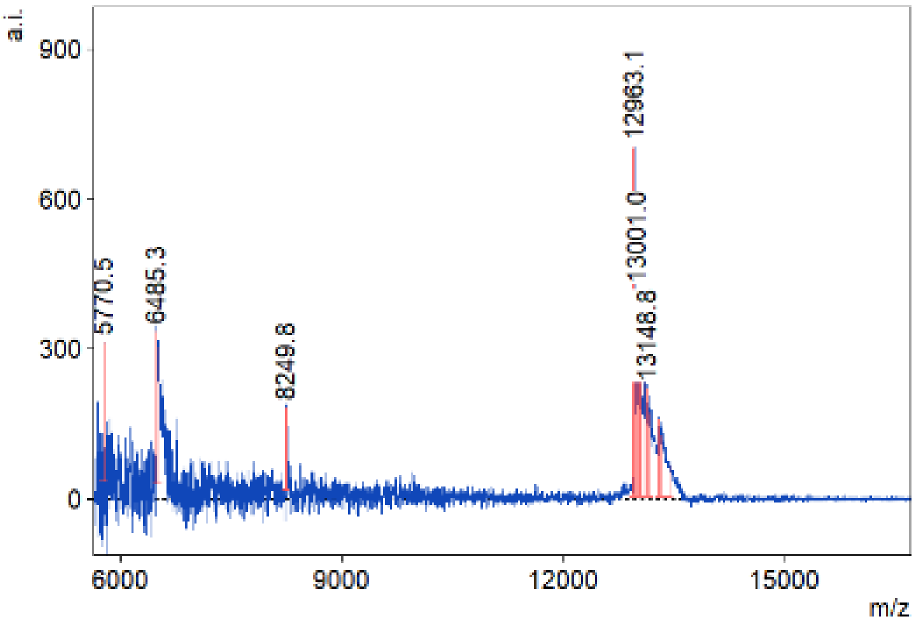

Figure S189. 31RNA_4U^{Dig}, calculated mass: 12961.2 Da, found mass: 12963.1 Da (product).

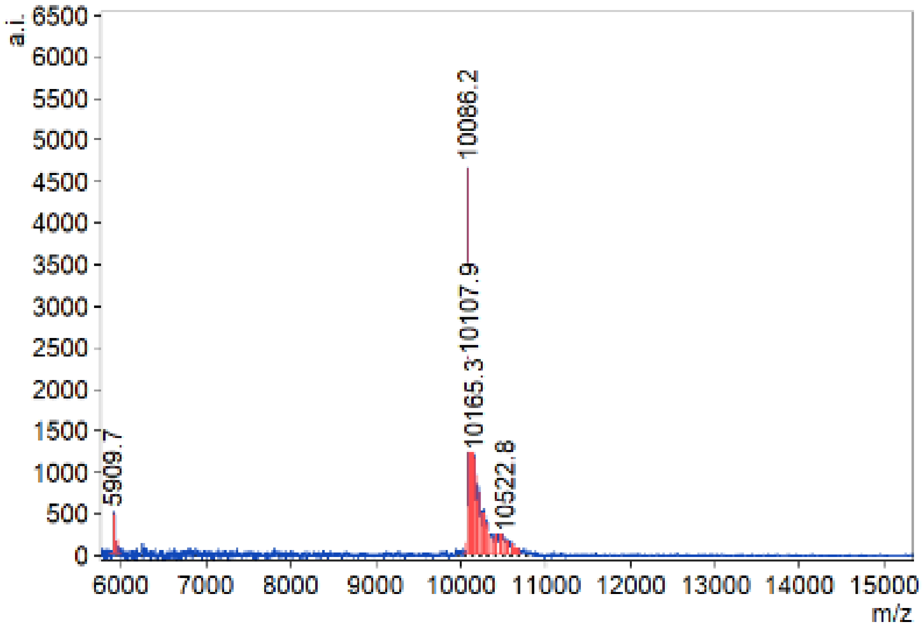

Figure S190. 31RNA_4C^{Me}, calculated mass: 10085.1 Da, found mass: 10086.2 Da (product).

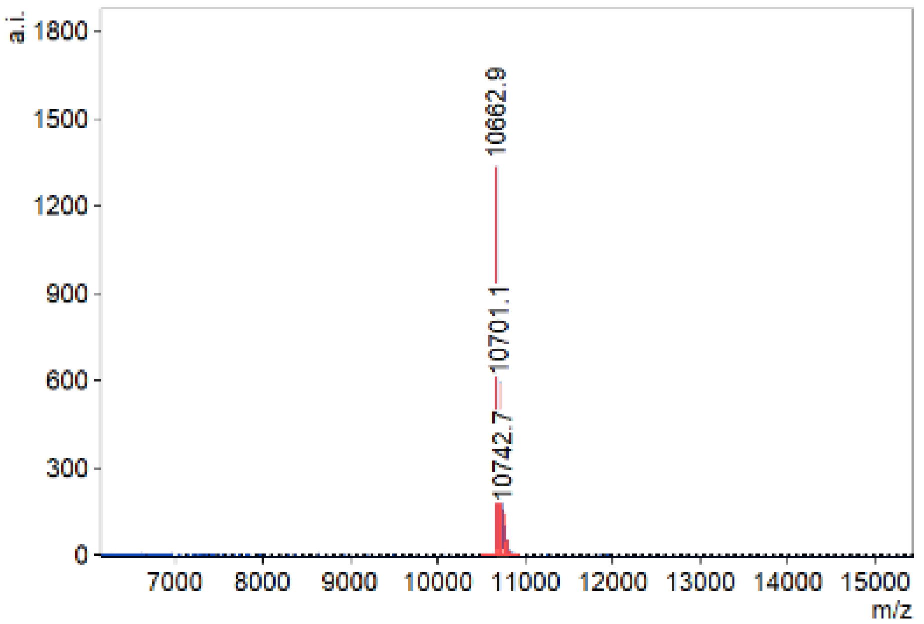

Figure S191. 31RNA_4C^{E}, calculated mass: 10662.1 Da, found mass: 10662.9 Da (product).

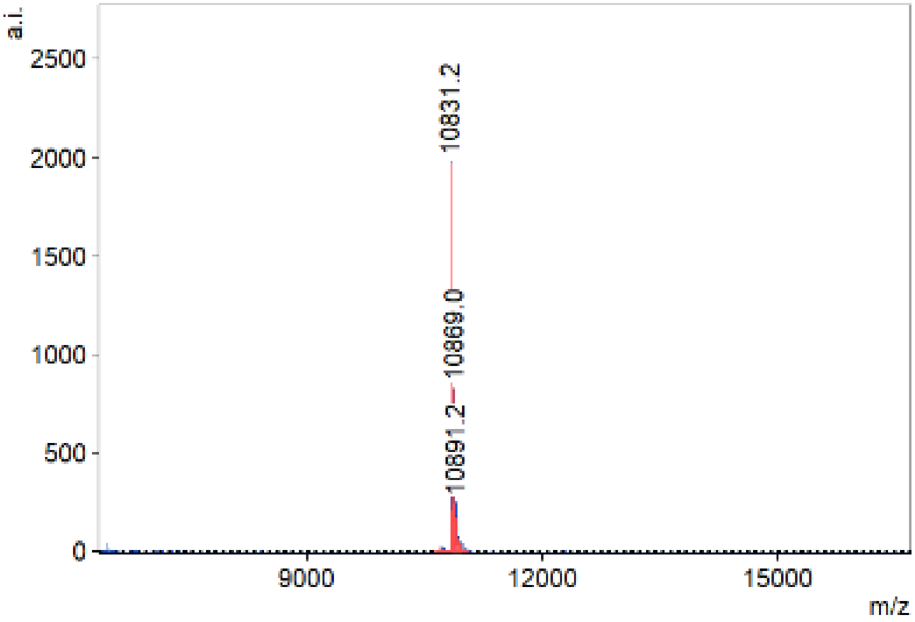

Figure S192. 31RNA_4C^{Pent}, calculated mass: 10830.4 Da, found mass: 10831.2 Da (product).

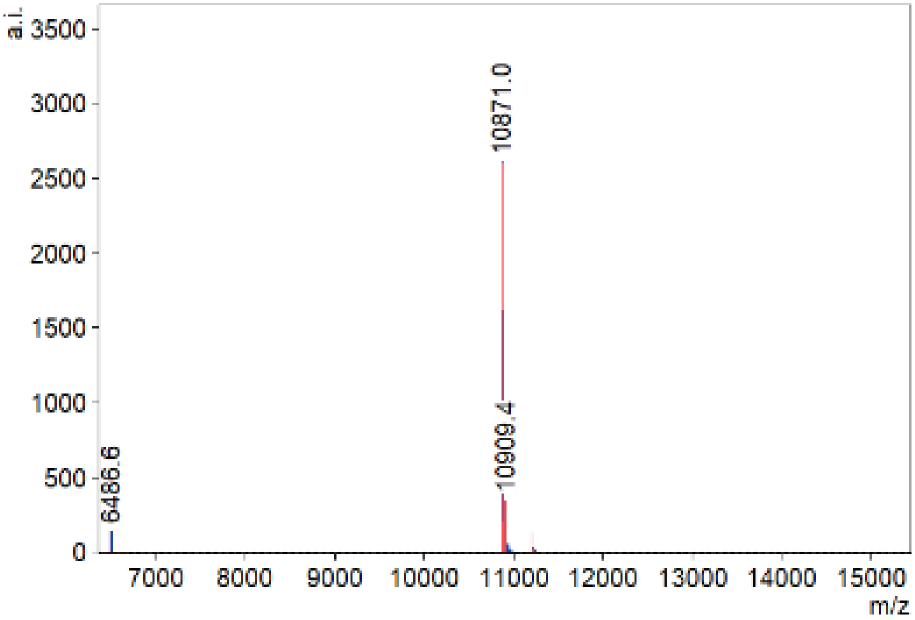

Figure S193. 31RNA_4C^{Ph}, calculated mass: 10870.4 Da, found mass: 10871.0 Da (product).

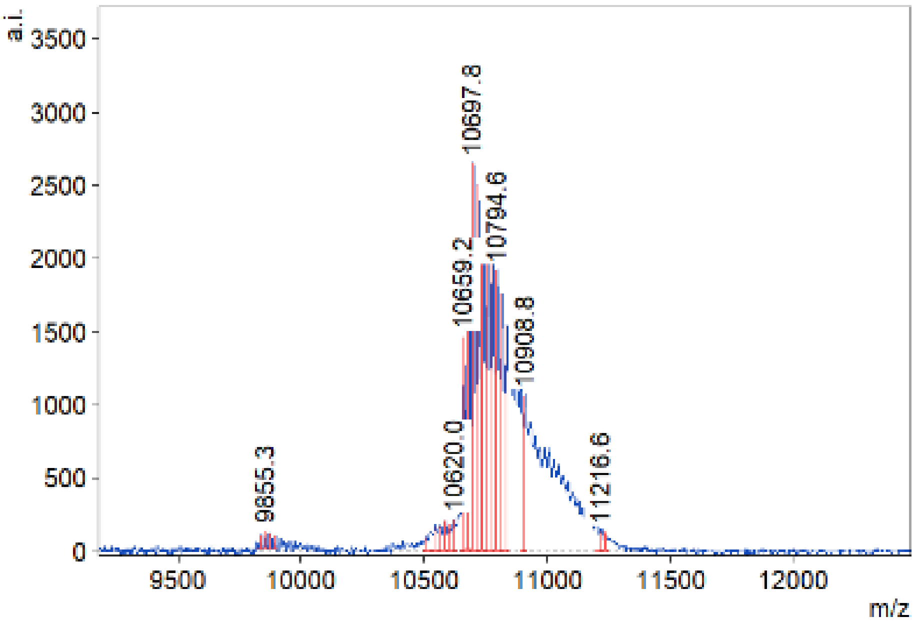

Figure S194. 31RNA_4G^{E}, calculated mass: 10658.16 Da, found mass: 10659.2 Da (product); found mass: 10697.8 (addition of H_2O = product + $2H_2O$).

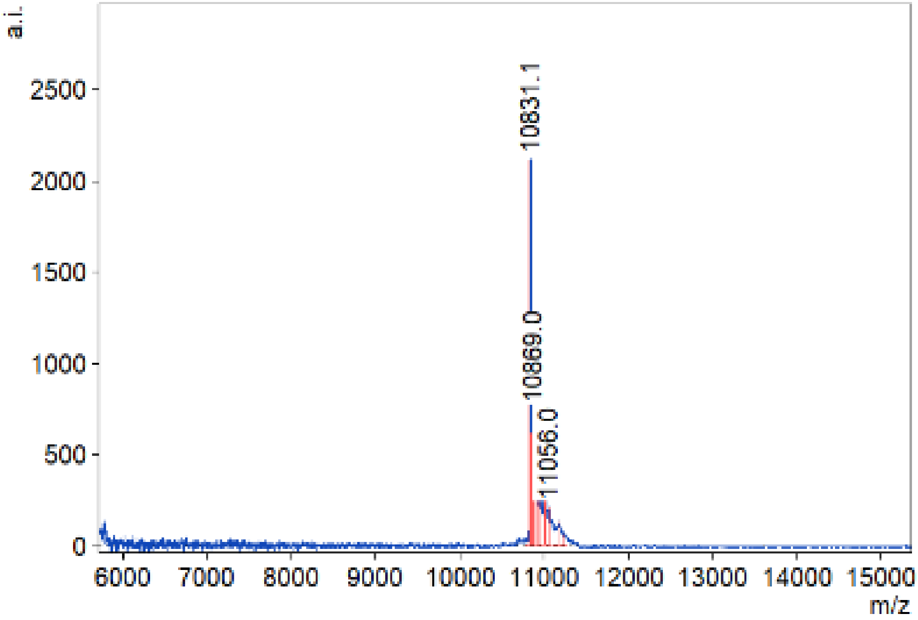

Figure S195. 31RNA_4G^{Pent}, calculated mass: 10826.5 Da, found mass: 10831.1 Da (product).

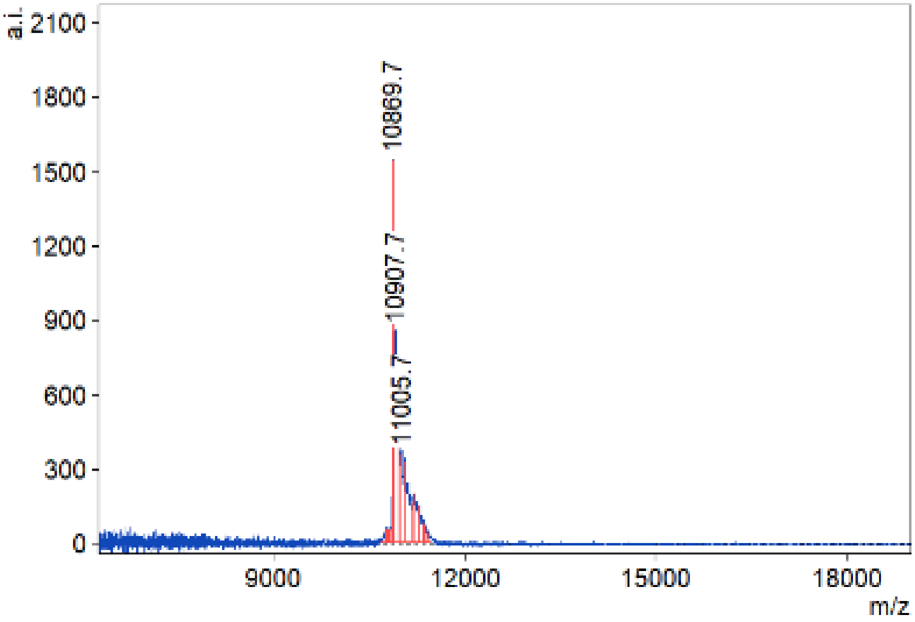

Figure S196. 31RNA_4G^{Ph}, calculated mass: 10866.4 Da, found mass: 10869.7 Da (product).

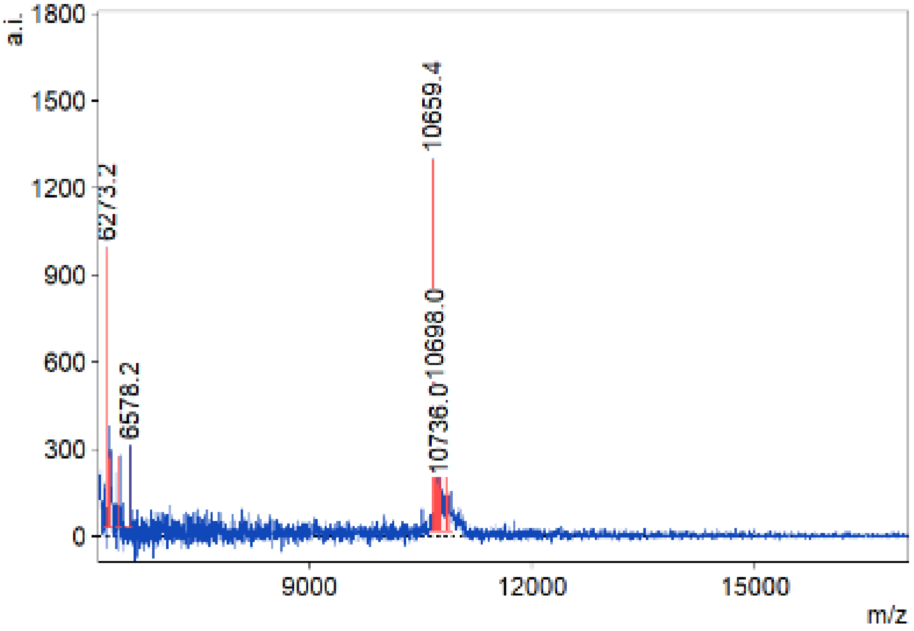

Figure S197. 31RNA_4A^{E}, calculated mass: 10658.1 Da, found mass: 10659.4 Da (product).

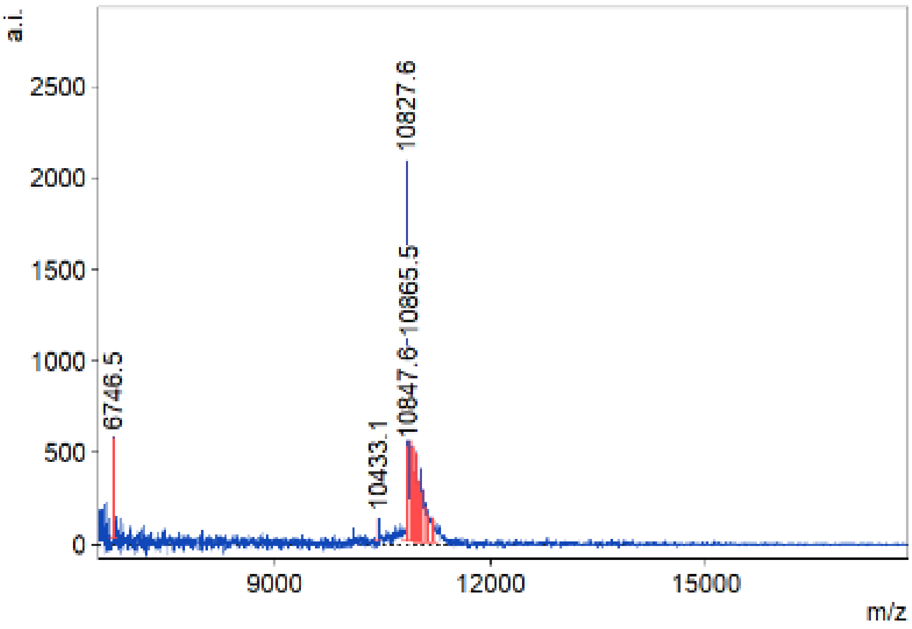

Figure S198. 31RNA_4A^{Pent}, calculated mass: 10826.4 Da, found mass: 10827.6 Da (product).

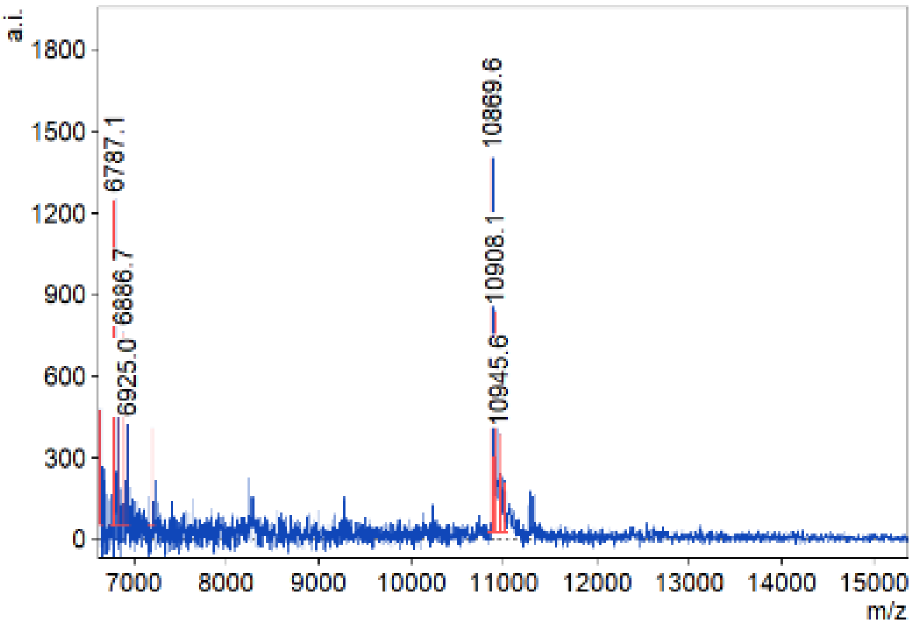

Figure S199. 31RNA_4A^{Ph}, calculated mass: 10866.4 Da, found mass: 10869.6 Da (product).

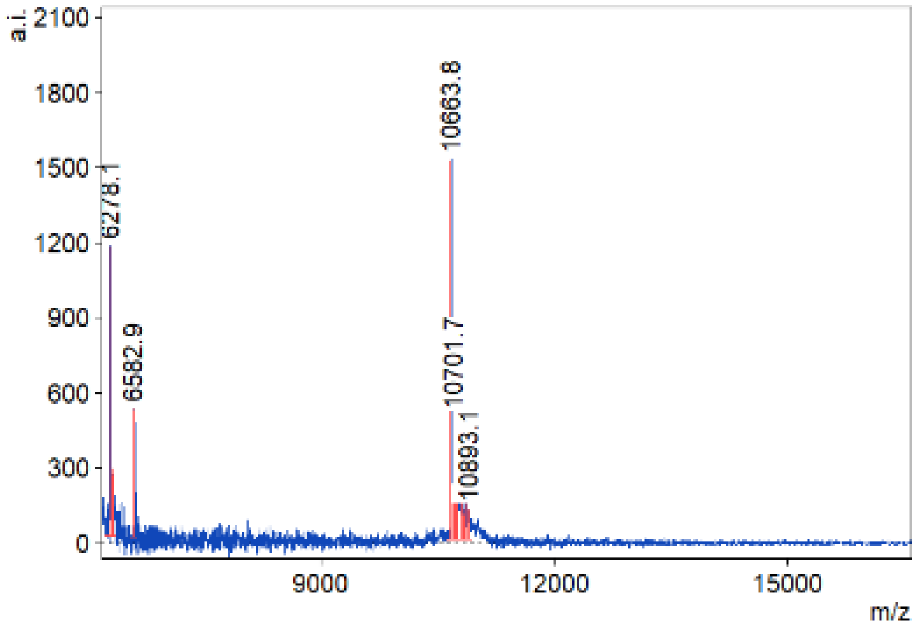

Figure S200. 31RNA_4U^{E}, calculated mass: 10662.1 Da, found mass: 10663.8 Da (product).

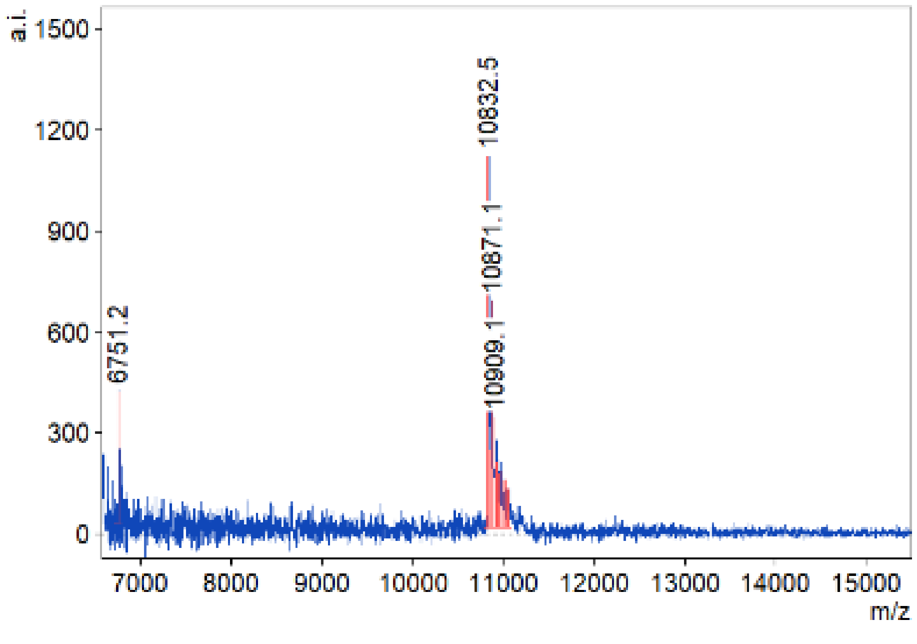

Figure S201. 31RNA_4U^{Pent}, calculated mass: 10830.4 Da, found mass: 10832.5 Da (product).

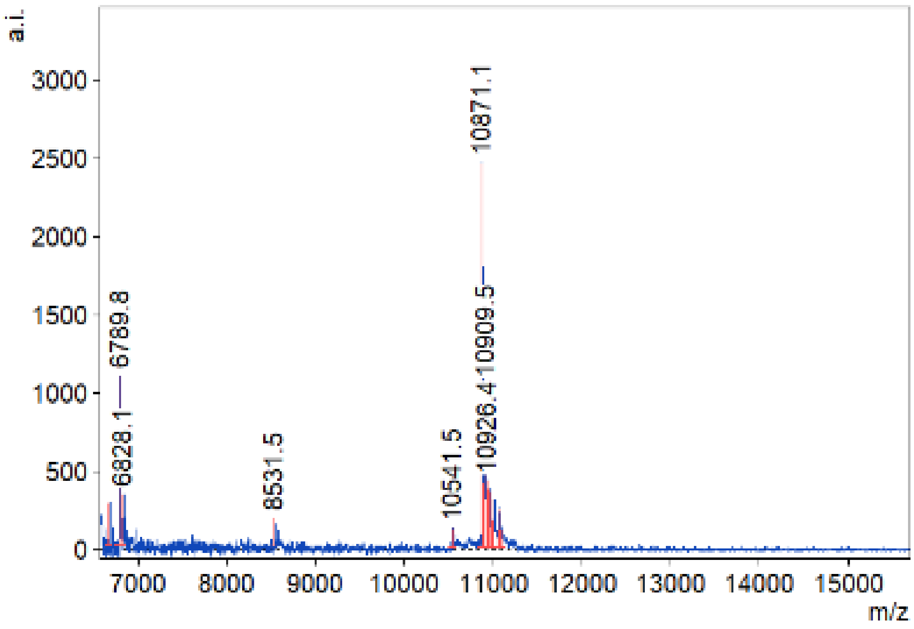

Figure S202. 31RNA_4U^{Ph}, calculated mass: 10870.4 Da, found mass: 10871.1 Da (product).

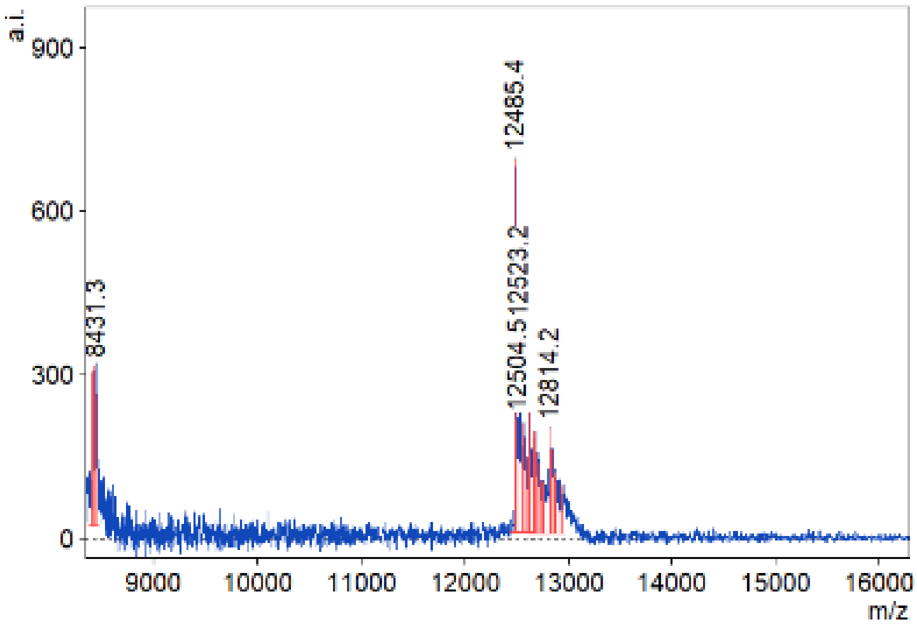

Figure S203. 31RNA_4U^{Bio}, calculated mass: 12484.6 Da, found mass: 12485.4 Da (product).

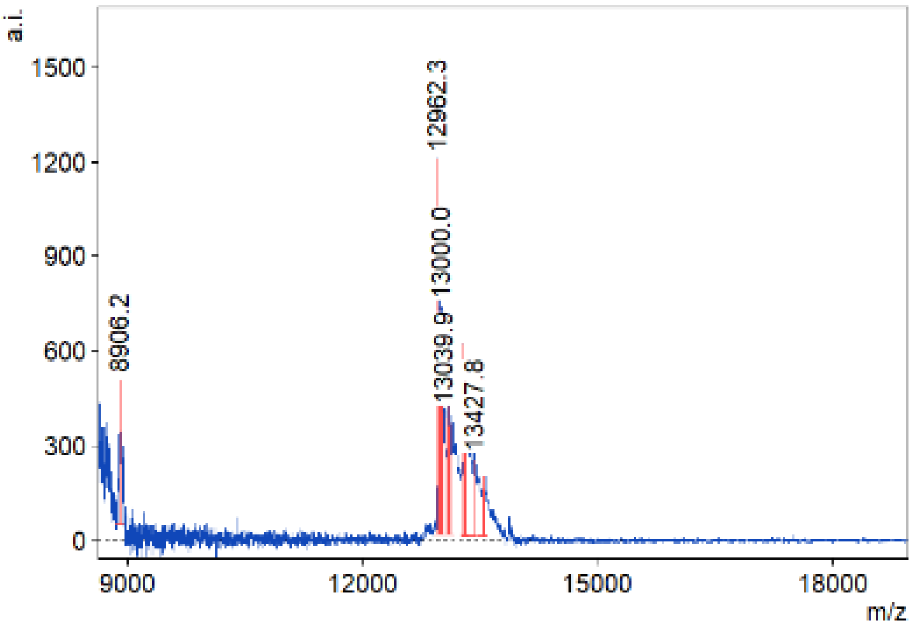

Figure S204. 31RNA_4U^{Dig}, calculated mass: 12961.1 Da, found mass: 12962.3 Da (product).

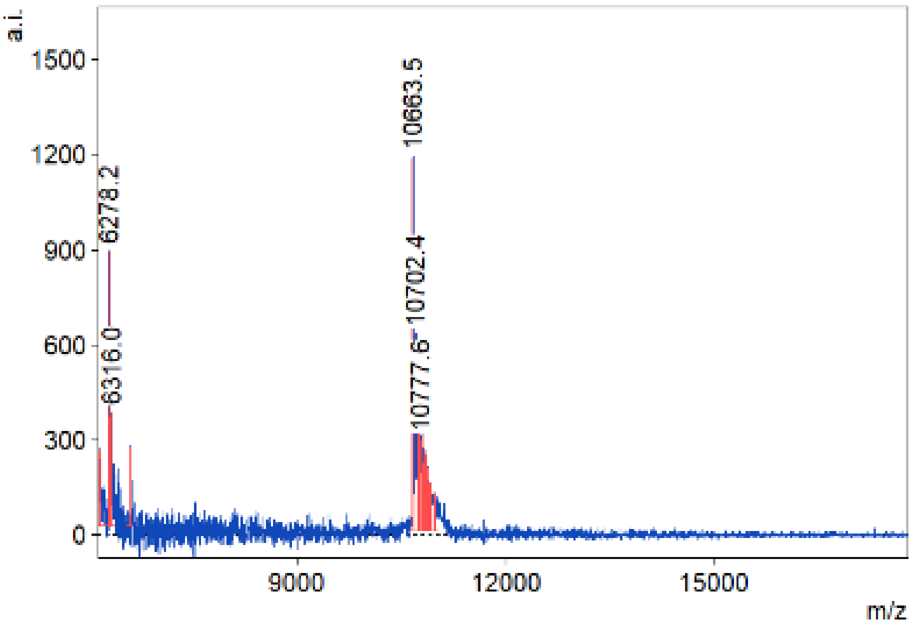

Figure S205. 31RNA_4C^{E}, calculated mass: 10662.1 Da, found mass: 10663.5 Da (product).

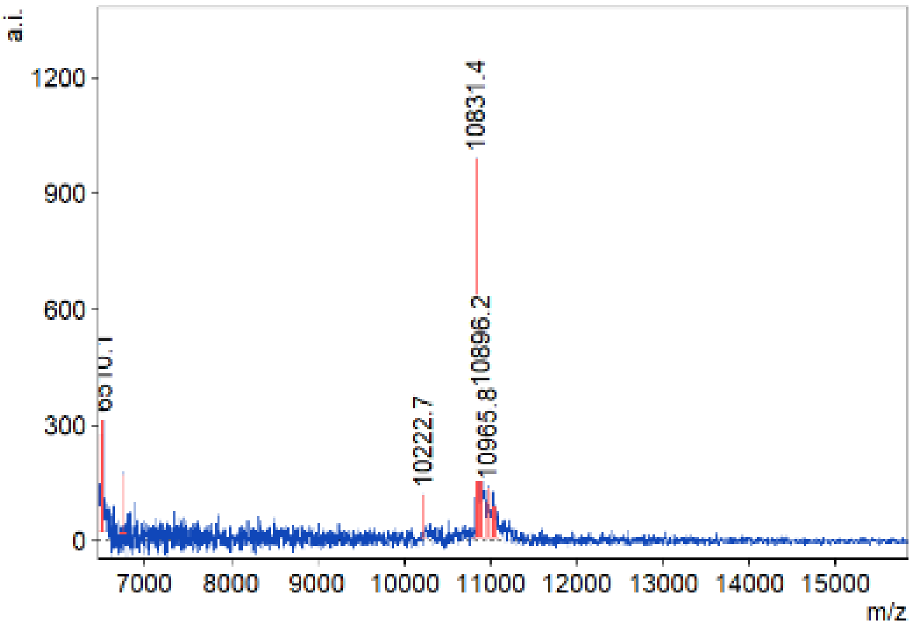

Figure S206. 31RNA_4C^{Pent}, calculated mass: 10830.4 Da, found mass: 10831.4 Da (product).

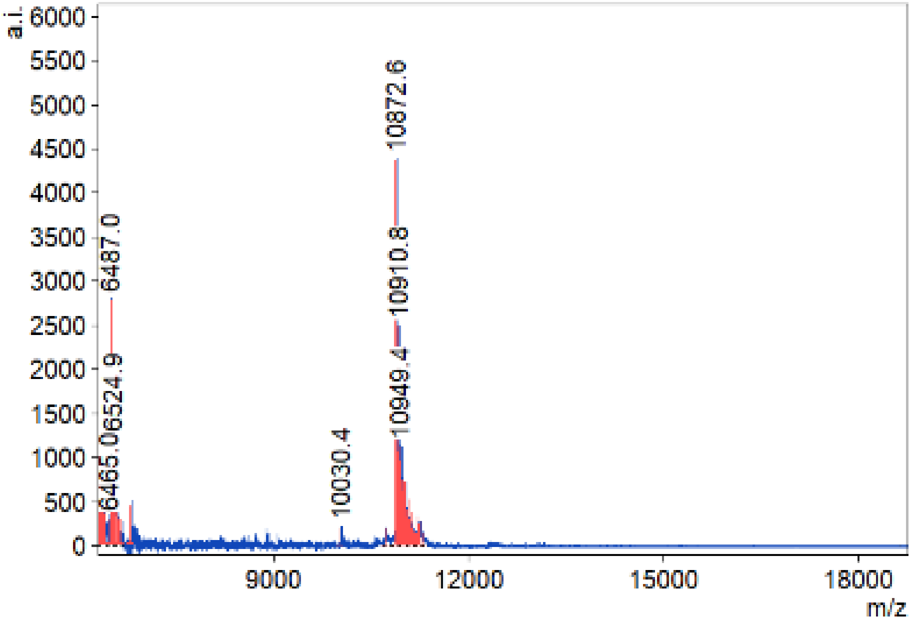

Figure S207. 31RNA_4C^{Ph}, calculated mass: 10870.4 Da, found mass: 10872.6 Da (product).

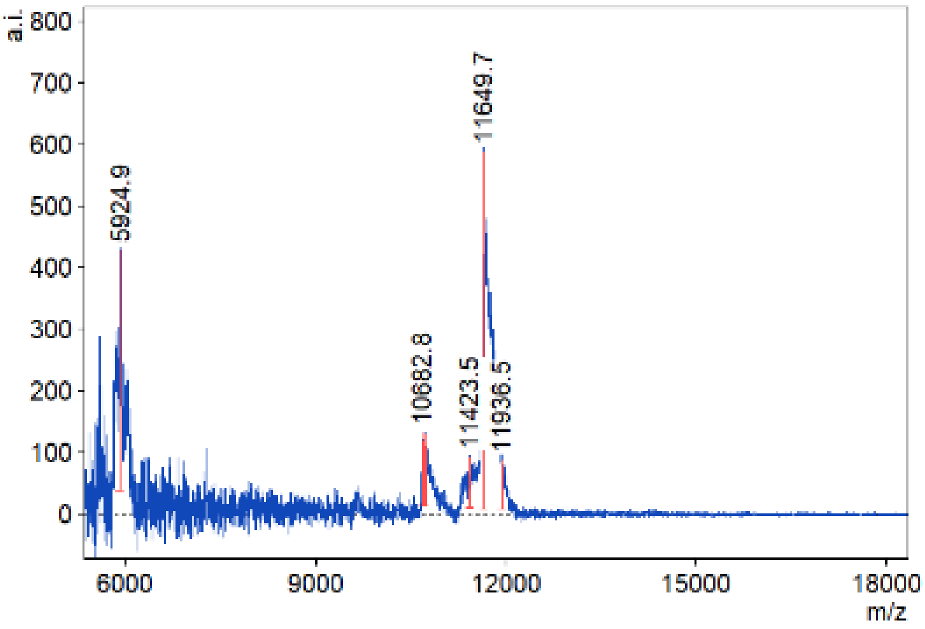

Figure S208. 31RNA_4C^{mBdp}, calculated mass: 11646.1 Da, found mass: 11649.7 Da (product).

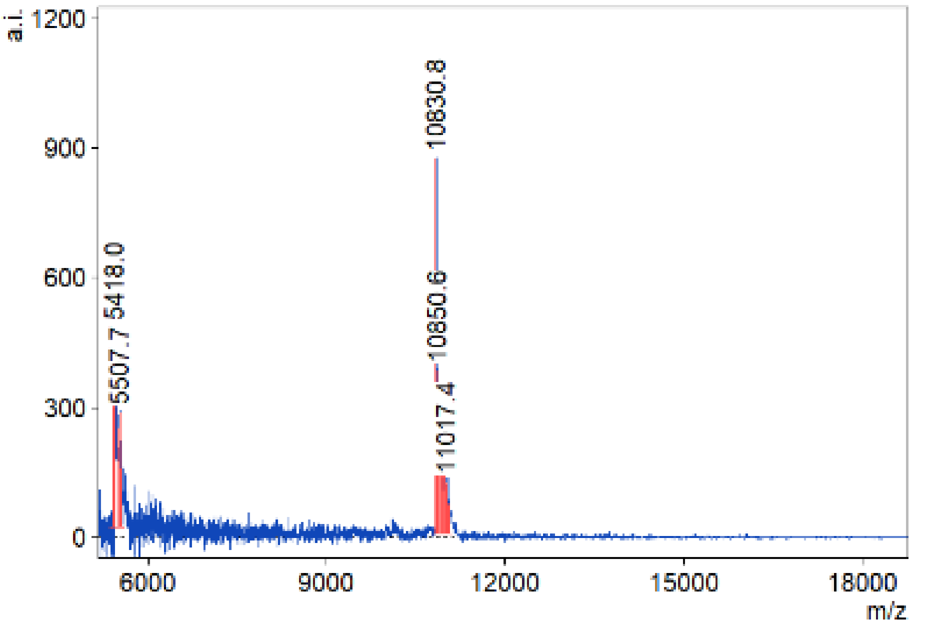

Figure S209. 31RNA_4G^{Pent}, calculated mass: 10826.5 Da, found mass: 10830.8 Da (product).

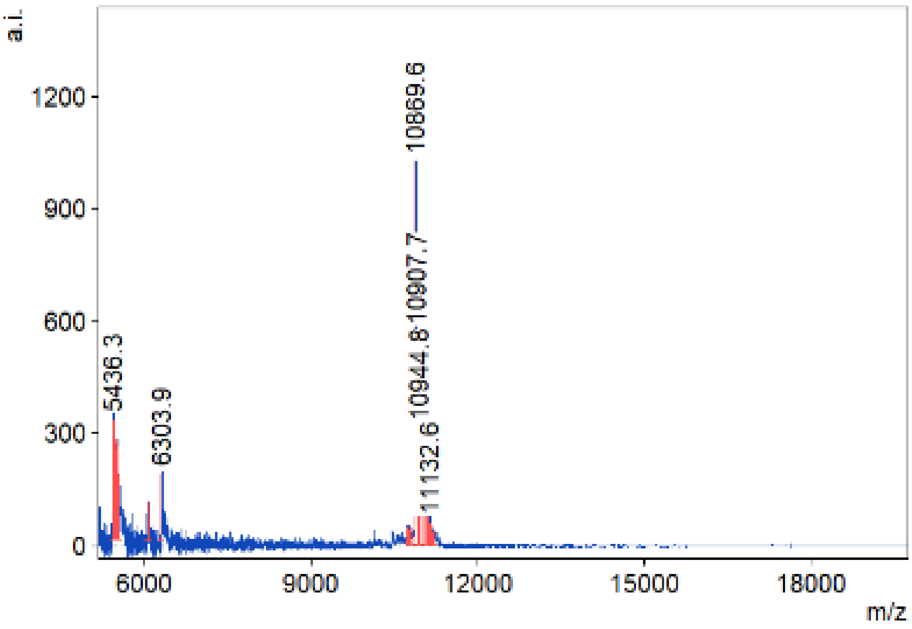

Figure S210. 31RNA_4G^{Ph}, calculated mass: 10866.4 Da, found mass: 10869.6 Da (product).

4.2 LC-ESI-MS spectra

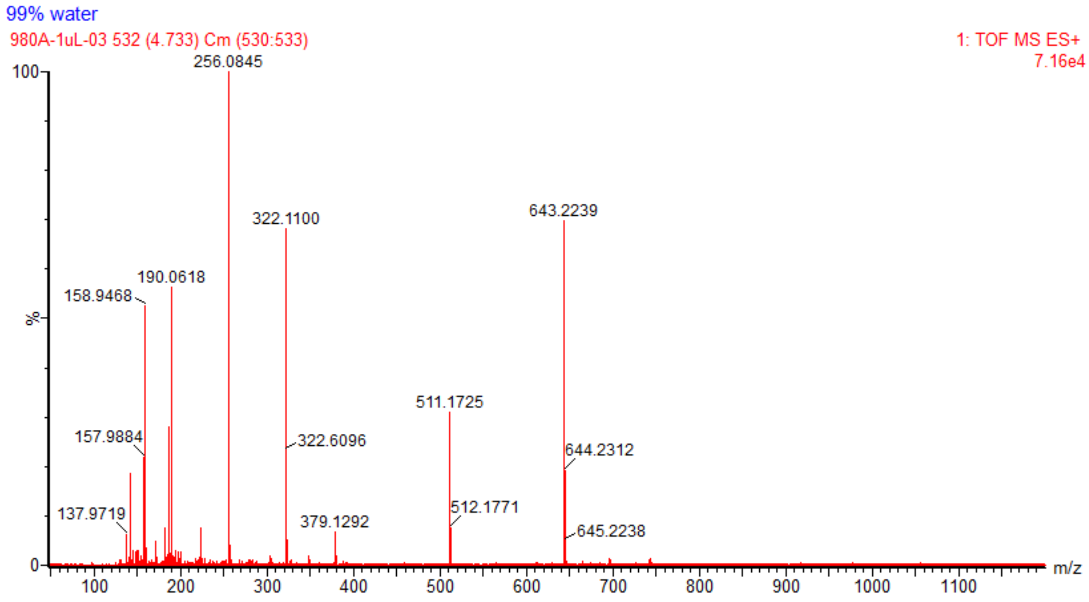

Figure S211. 19RNA_A^{CA} digest, raw spectrum, calculated mass: 643.2225 Da, found mass: 643.2239 Da (product).

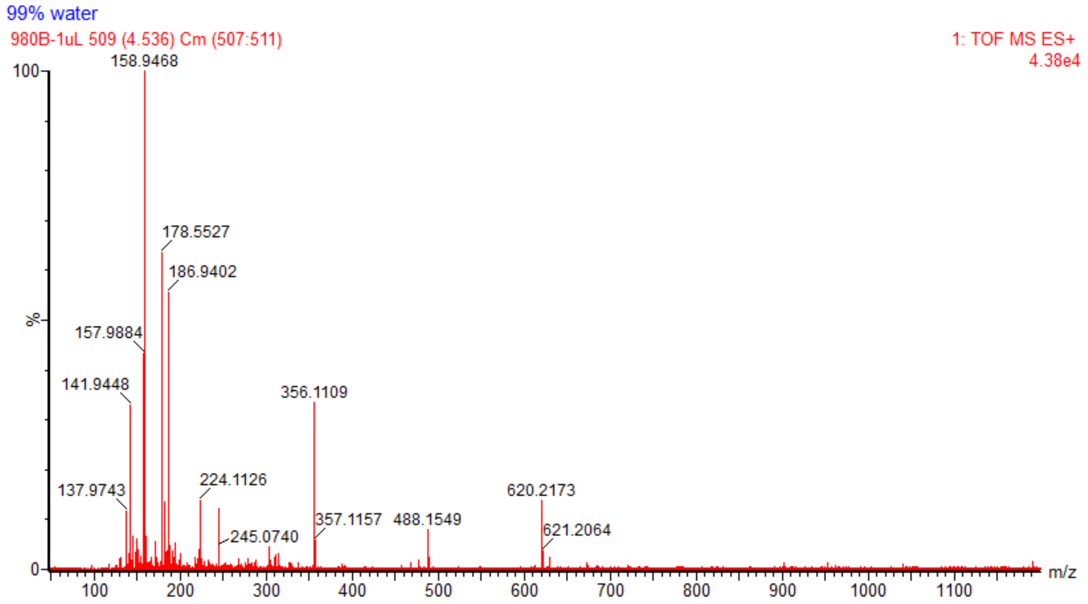

Figure S212. 19RNA_C^{CA} digest, raw spectrum, calculated mass: 620.2065 Da, found mass: 620.2173 Da (product).

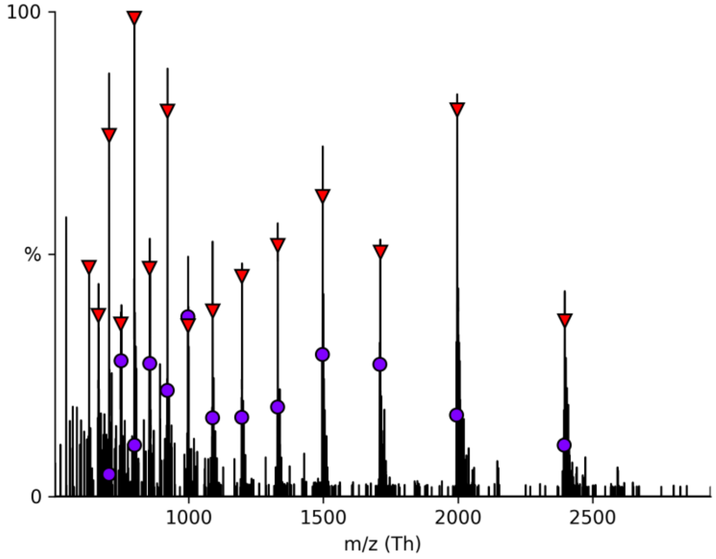

Figure S213. 31RNA_4C^{mBdp}, raw spectrum.

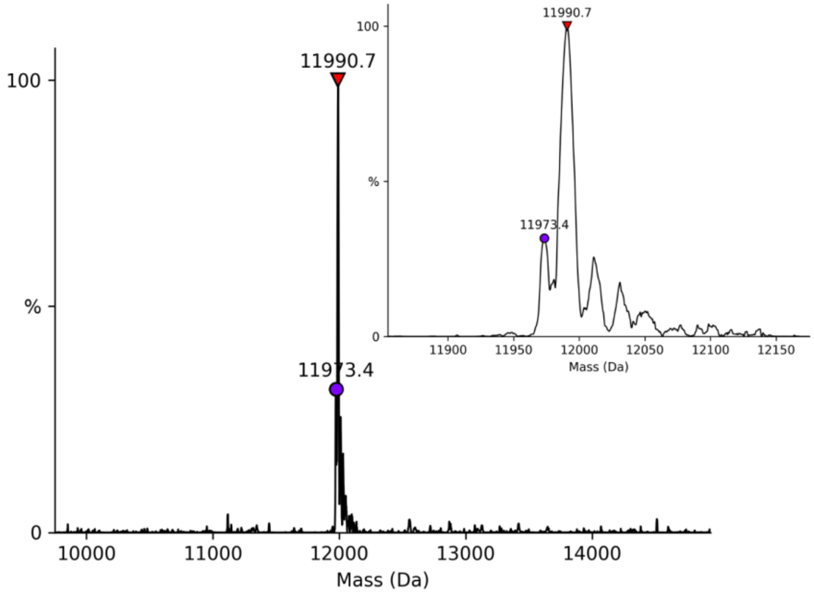

Figure S214. 31RNA_4C^{mBdp}, deconvoluted spectrum, calculated mass: 11646.1 Da, found mass: 11990.7 Da (product + rGMP); found mass: 11973.4 Da (product + rAMP).

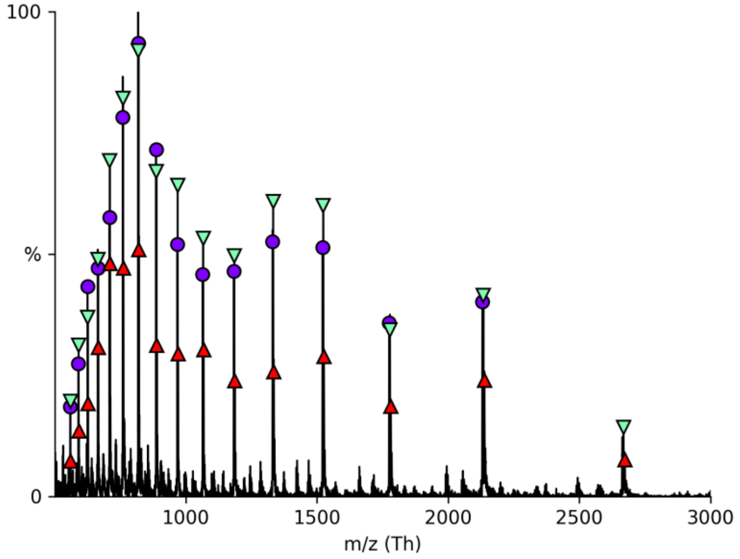

Figure S215. 31RNA_4G^{E}, raw spectrum.

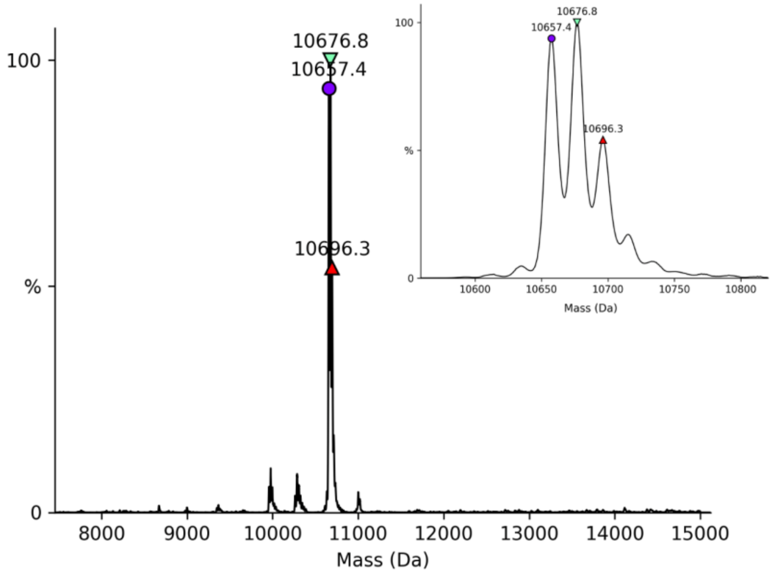

Figure S216. 31RNA_4G^{E}, deconvoluted spectrum, calculated mass: 10658.2 Da, found mass: 10657.4 Da (product); found mass: 10676.8 (addition of H_2O = product + H_2O); found mass: 10696.3 (addition of H_2O = product + $2H_2O$).

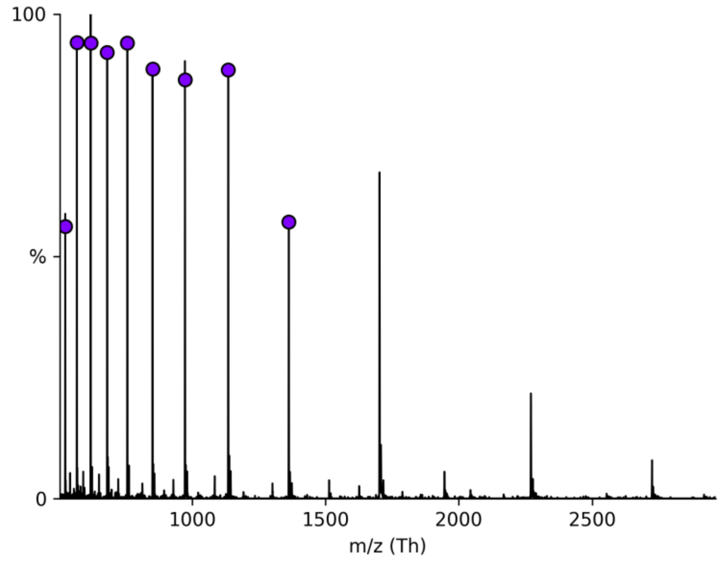

Figure S217. 19RNA_A^{E}U^{Bio}C^{Ph}G^{Pent}, raw spectrum.

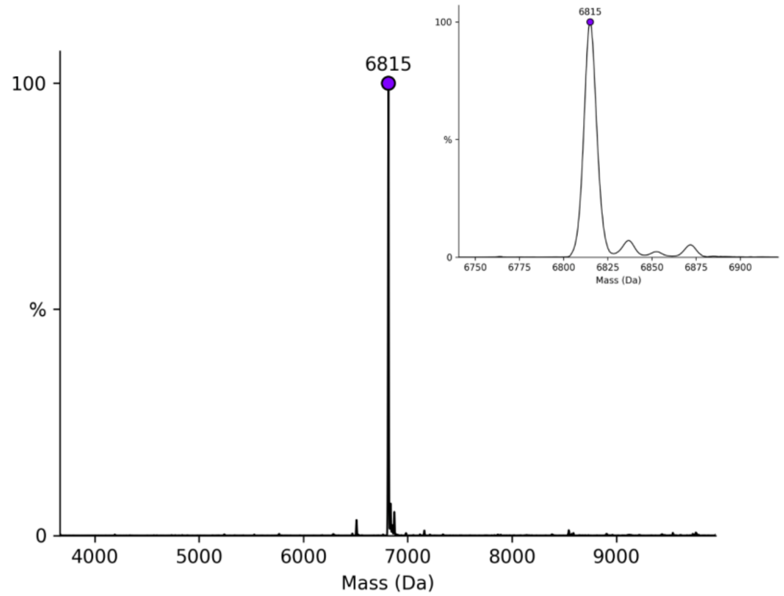

Figure S218. 19RNA_A^{E}U^{Bio}C^{Ph}G^{Pent}, deconvoluted spectrum, calculated mass: 6815.6 Da, found mass: 6815.0 Da (product).

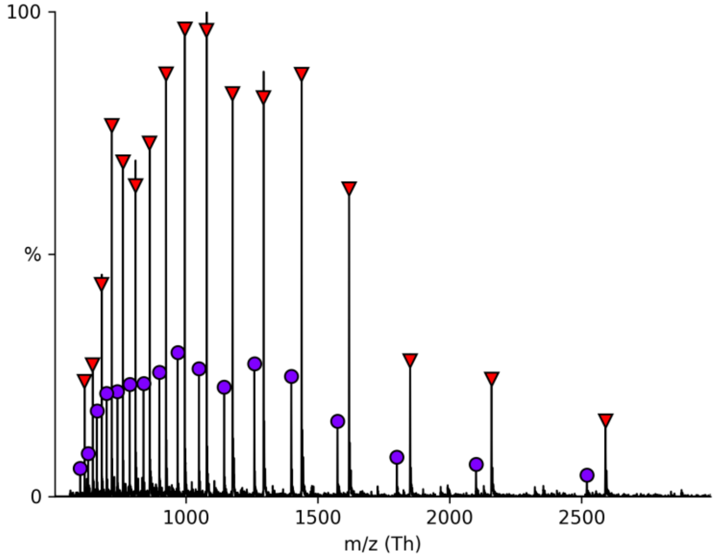

Figure S219. 31RNA_A^{E}U^{Bio}C^{Ph}G^{Pent}, raw spectrum.

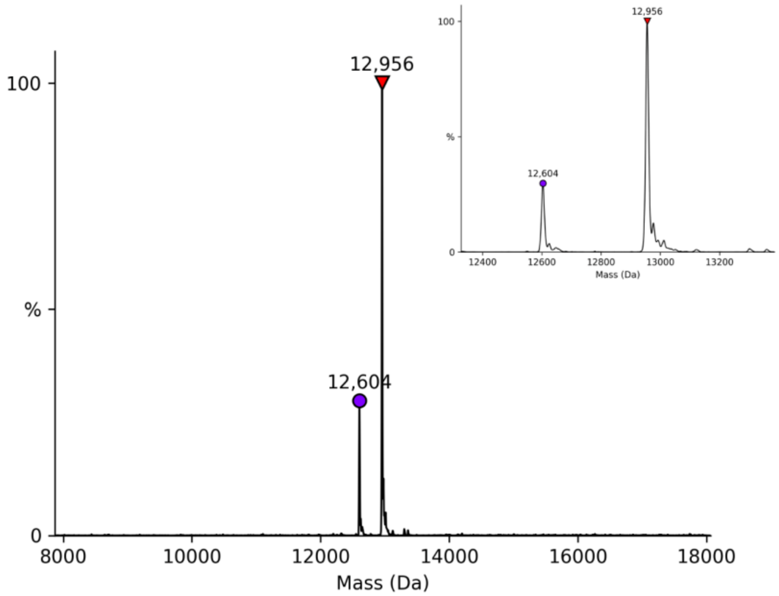

Figure S220. 31RNA_A^{E}U^{Bio}C^{Ph}G^{Pent}, deconvoluted spectrum, calculated mass: 12604.6 Da, found mass: 12604.0 Da (product); found mass: 12956.0 (product + **rA^{E}MP**).

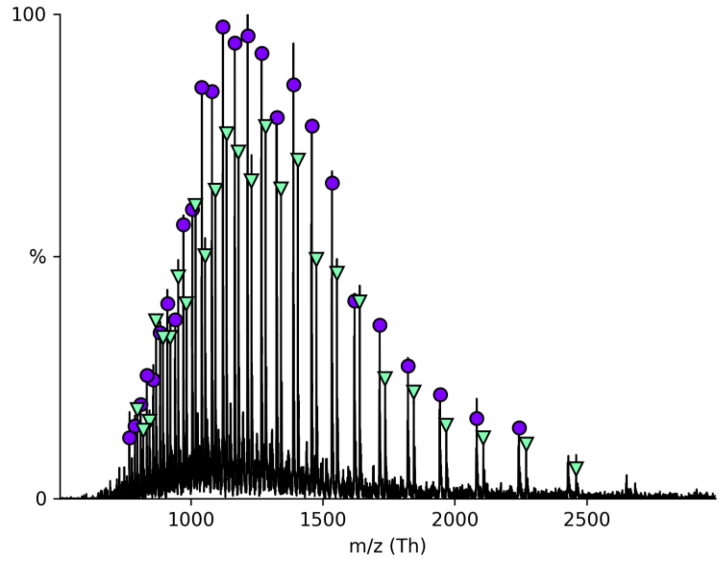

Figure S221. 65RNA_A^{E}U^{Bio}C^{Ph}G^{Pent}, raw spectrum.

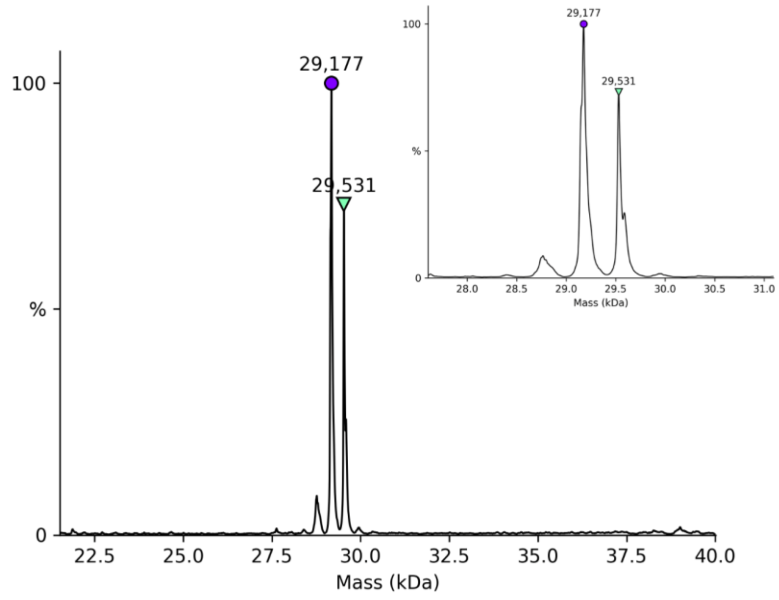

Figure S222. 65RNA_A^{E}U^{Bio}C^{Ph}G^{Pent}, deconvoluted spectrum, calculated mass: 29179.8 Da, found mass: 29177.0 Da (product); found mass: 29531.0 (product + **rA^{E}MP**).

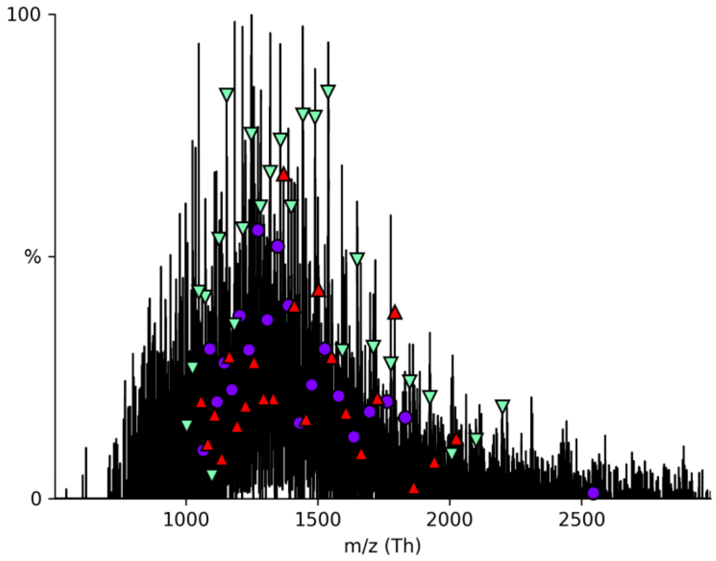

Figure S223. 98RNA_A^{E}U^{Bio}C^{Ph}G^{Pent}, raw spectrum.

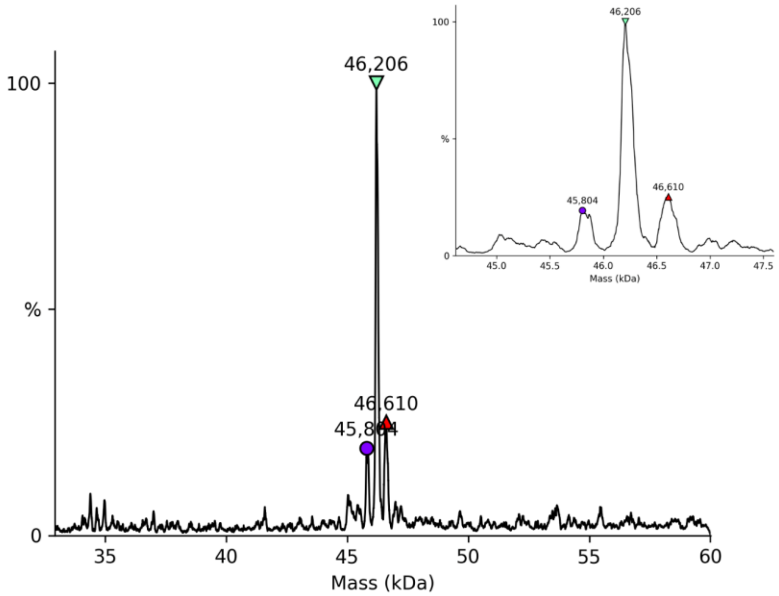

Figure S224. 98RNA_A^{E}U^{Bio}C^{Ph}G^{Pent}, deconvoluted spectrum, calculated mass: 45836.3 Da, found mass: 45804.0 Da (product); found mass: 46206.0 (product + **rA^{E}MP** or product + **rC^{Ph}MP**).

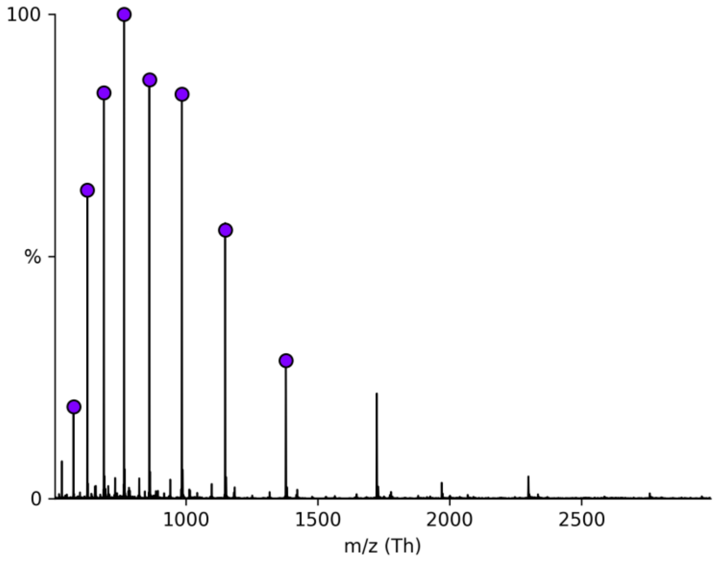

Figure S225. RNA_C^{Cy5}, raw spectrum.

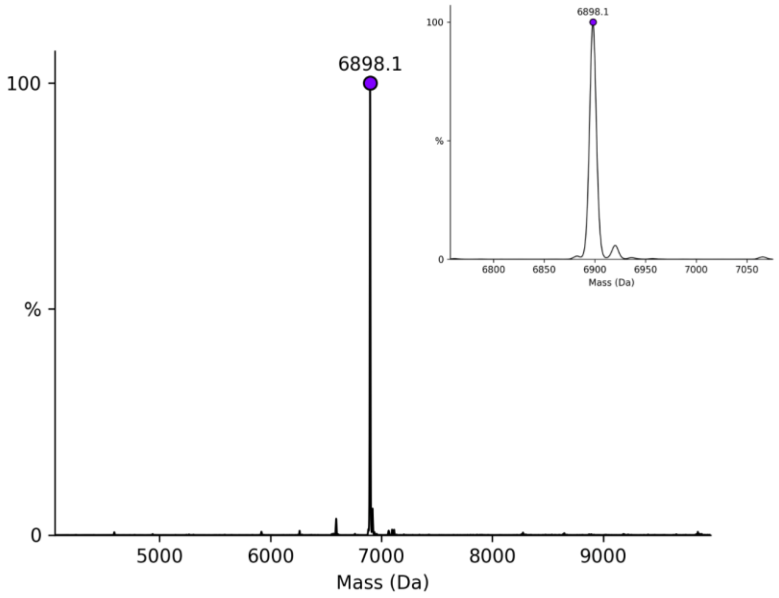

Figure S226. RNA_C^{Cy5}, deconvoluted spectrum, calculated mass: 6898.6 Da, found mass: 6898.1 Da (product).

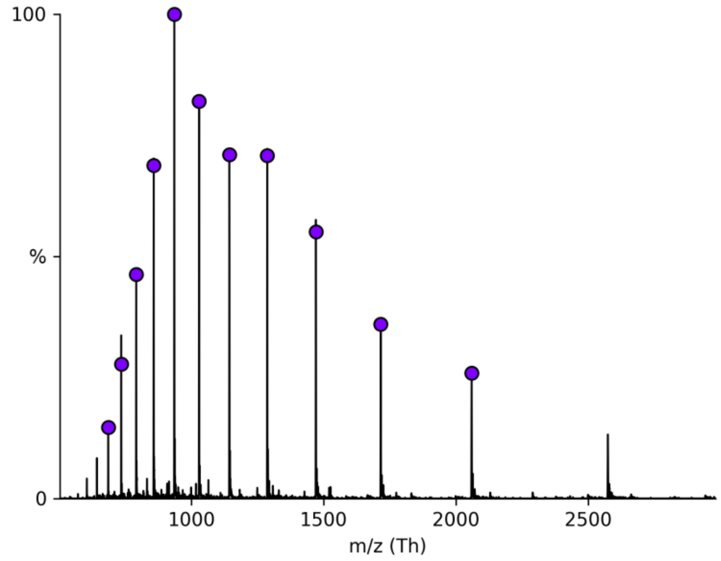

Figure S227. RNA_A^{E}U^{Bio}C^{Cy5}G^{Pent}, raw spectrum.

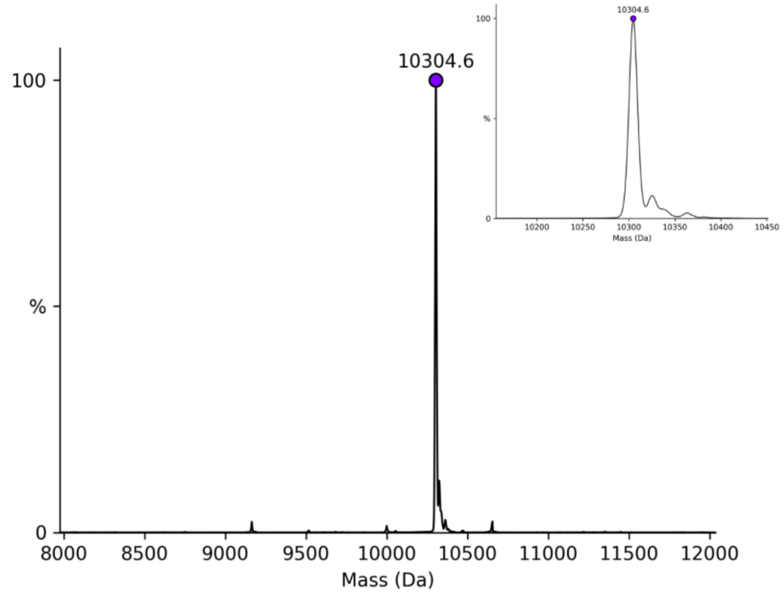

Figure S228. RNA_A^{E}U^{Bio}C^{Cy5}G^{Pent}, deconvoluted spectrum, calculated mass: 10305.3 Da, found mass: 10304.6 Da (product).

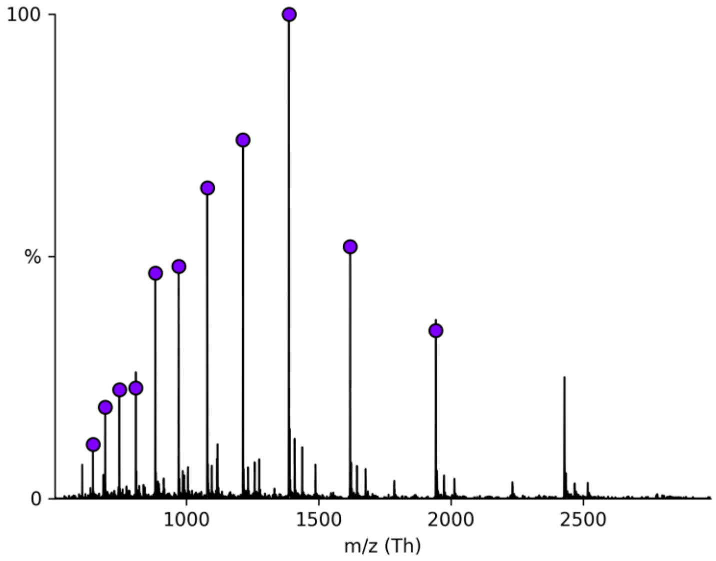

Figure S229. dT^{FAM}-RNA_A^{E}U^{Bio}C^{Ph}G^{Pent}, raw spectrum.

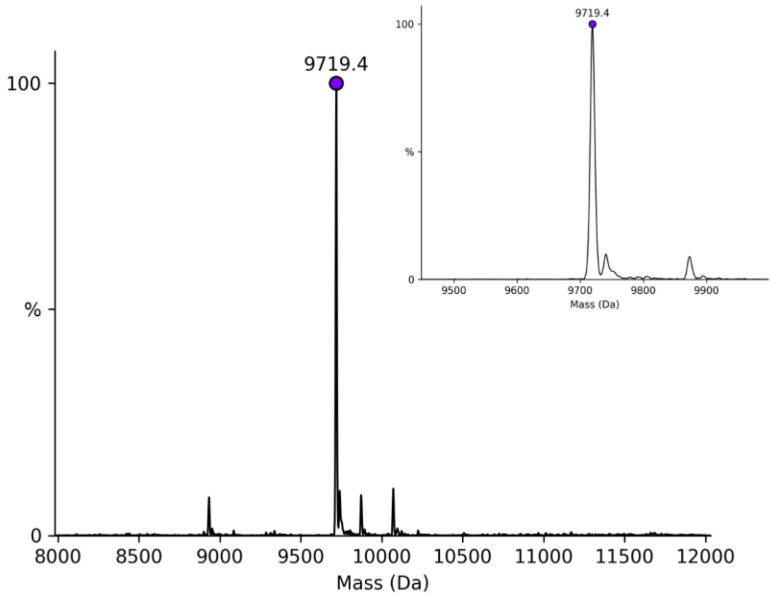

Figure S230. dT^{FAM}-RNA_A^{E}U^{Bio}C^{Ph}G^{Pent}, deconvoluted spectrum, calculated mass: 9719.4 Da, found mass: 9719.4 Da (product).

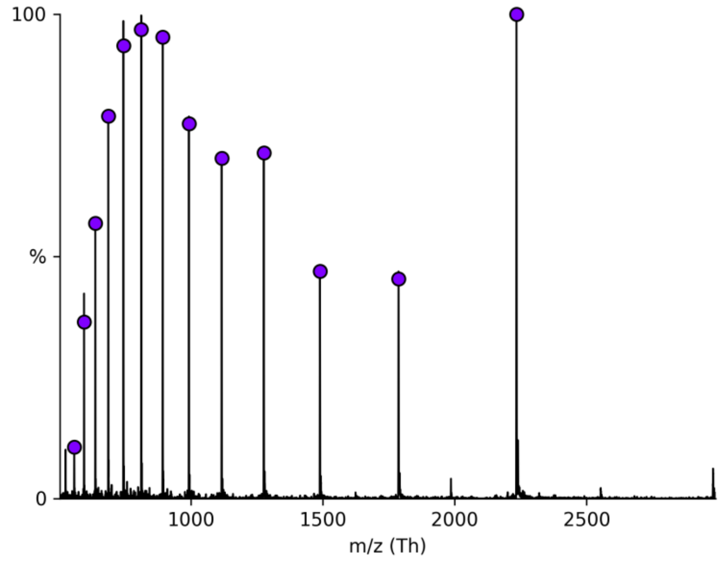

Figure S231. FAM-Cy5-Cy3-riboswitch_SNI-1, raw spectrum.

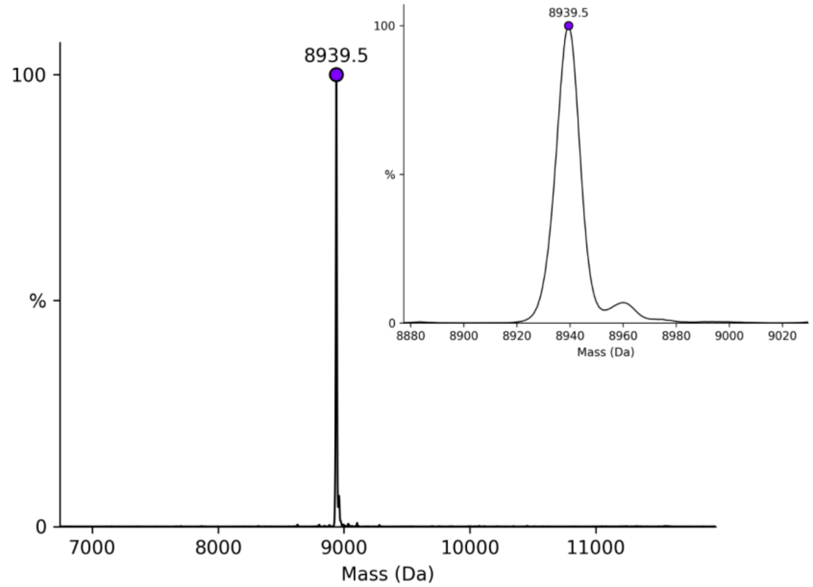

Figure S232. FAM-Cy5-Cy3-riboswitch_SNI-1, deconvoluted spectrum, calculated mass: 8940.5 Da, found mass: 8939.5 Da (product).

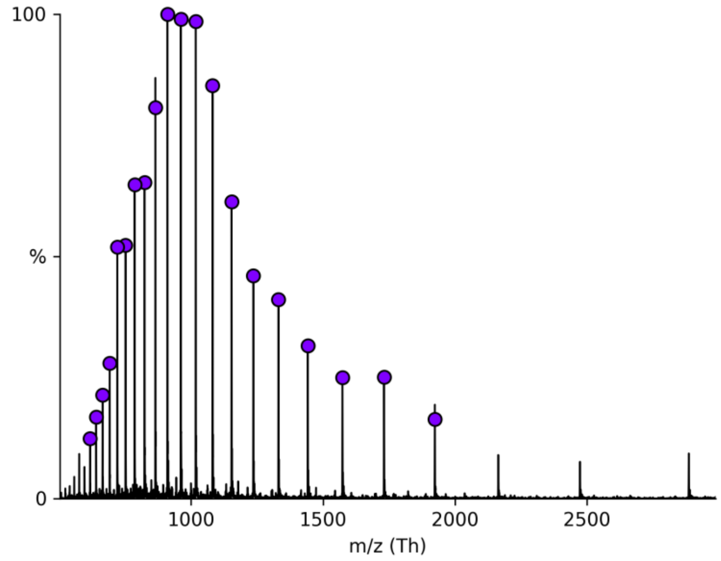

Figure S233. FAM-Cy5-Cy3-riboswitch_PEX-1, raw spectrum.

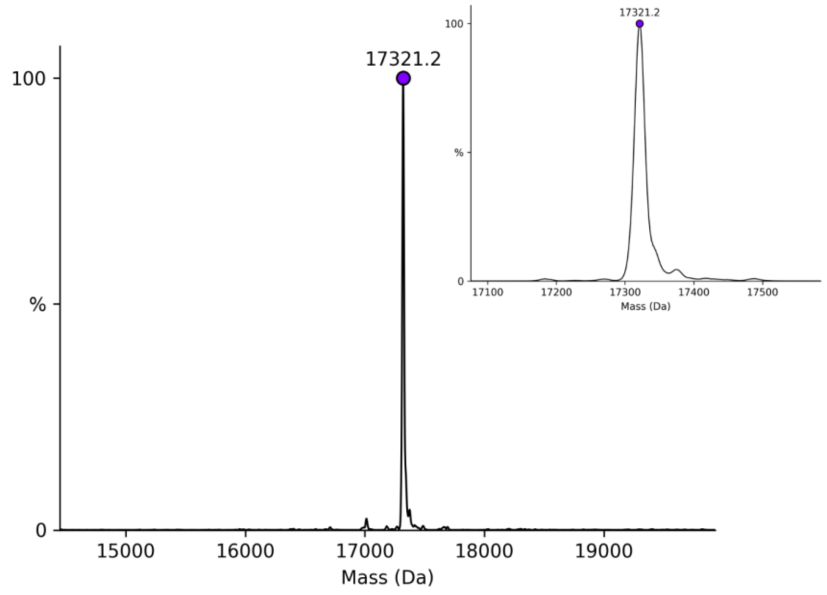

Figure S234. FAM-Cy5-Cy3-riboswitch_PEX-1, deconvoluted spectrum, calculated mass: 17323.5 Da, found mass: 17321.2 Da (product).

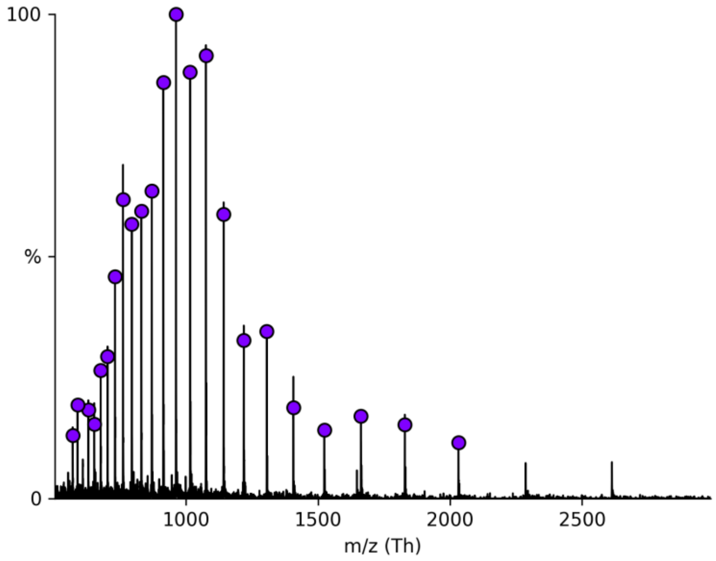

Figure S235. FAM-Cy5-Cy3-riboswitch_SNI-2, raw spectrum.

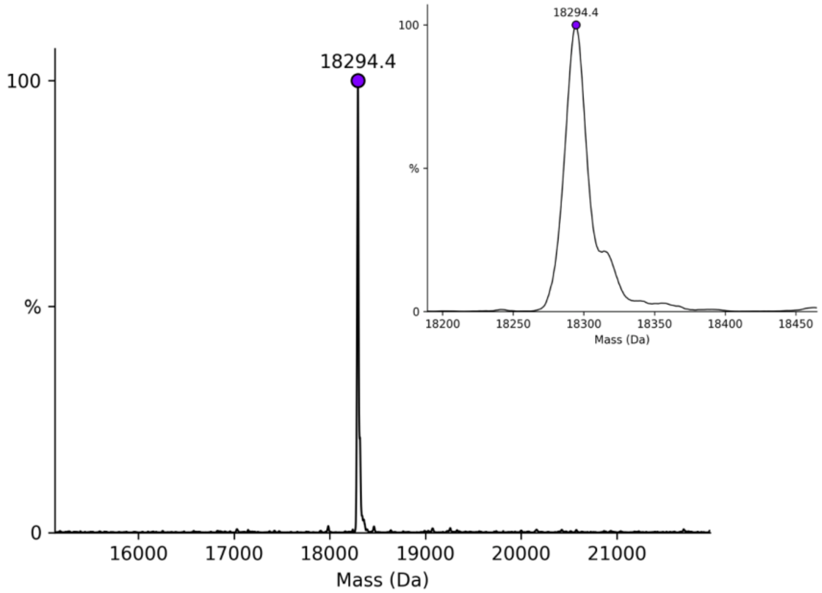

Figure S236. FAM-Cy5-Cy3-riboswitch_SNI-2, deconvoluted spectrum, calculated mass: 18297.4 Da, found mass: 18294.4 Da (product).

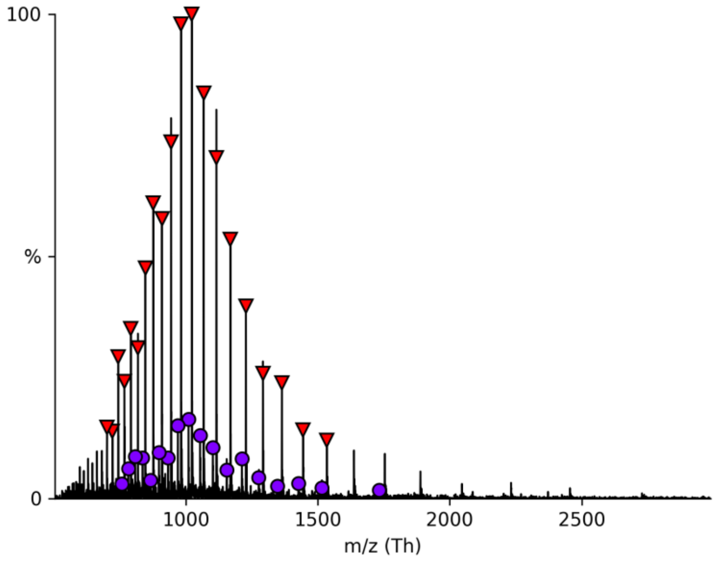

Figure S237. FAM-Cy5-Cy3-riboswitch_PEX-2, raw spectrum.

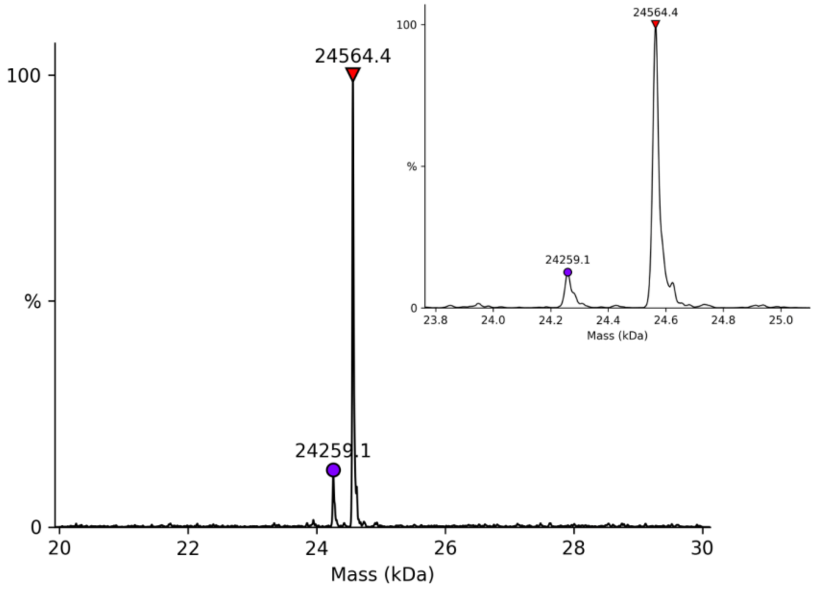

Figure S238. FAM-Cy5-Cy3-riboswitch_PEX-2, deconvoluted spectrum, calculated mass: 24569.1 Da, found mass: 24564.4 Da (product); found mass: 24259.1 Da (product - rCMP).

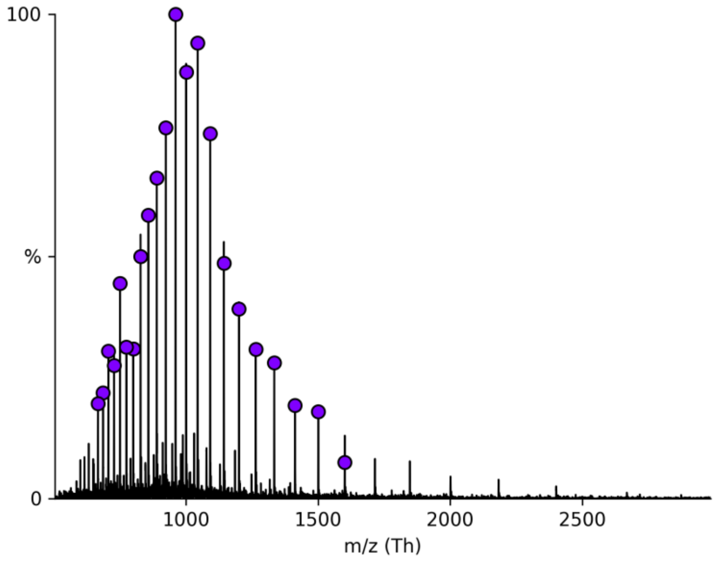

Figure S239. Cy5-Cy3-riboswitch, raw spectrum.

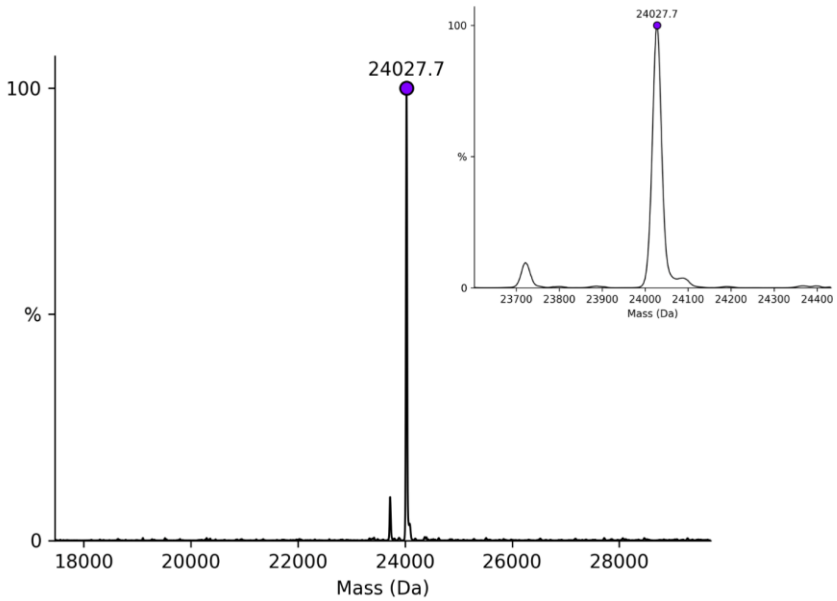

Figure S240. Cy5-Cy3-riboswitch, deconvoluted spectrum, calculated mass: 24032.1 Da, found mass: 24027.7 Da (product).

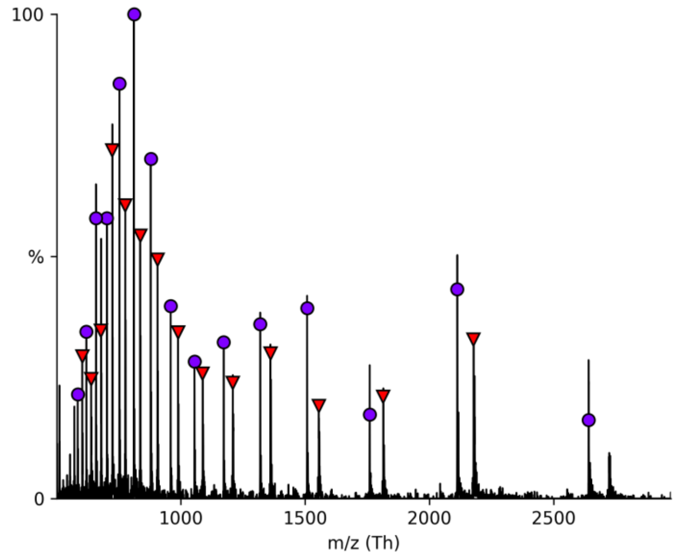

Figure S241. 31RNA_nat, 1st replicate, raw spectrum.

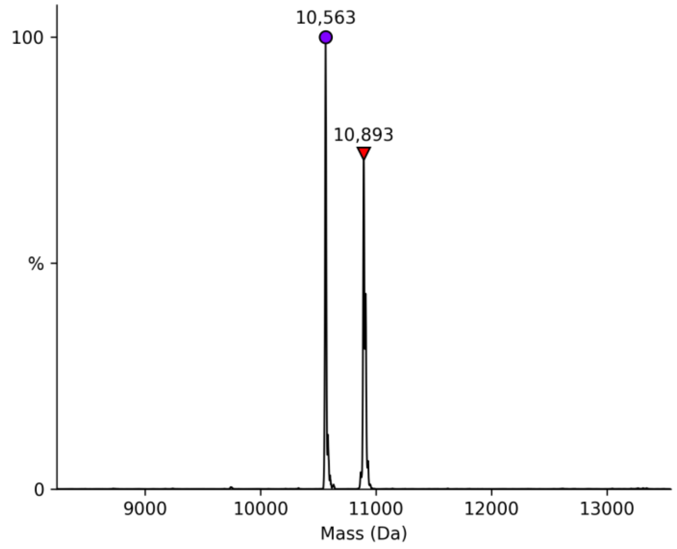

Figure S242. 31RNA_nat, 1st replicate, deconvoluted spectrum, calculated mass: 10566.0 Da, found mass: 10563.0 Da (product); found mass: 10893.0 Da (product + rAMP).

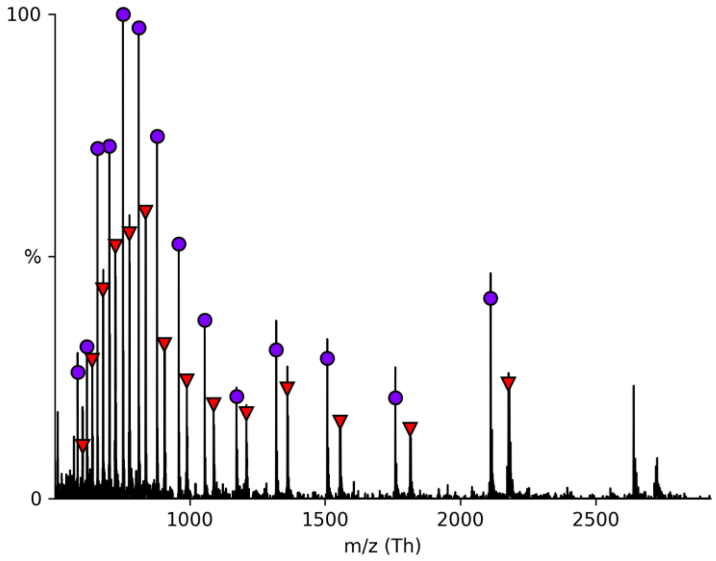

Figure S243. 31RNA_nat, 2nd replicate, raw spectrum.

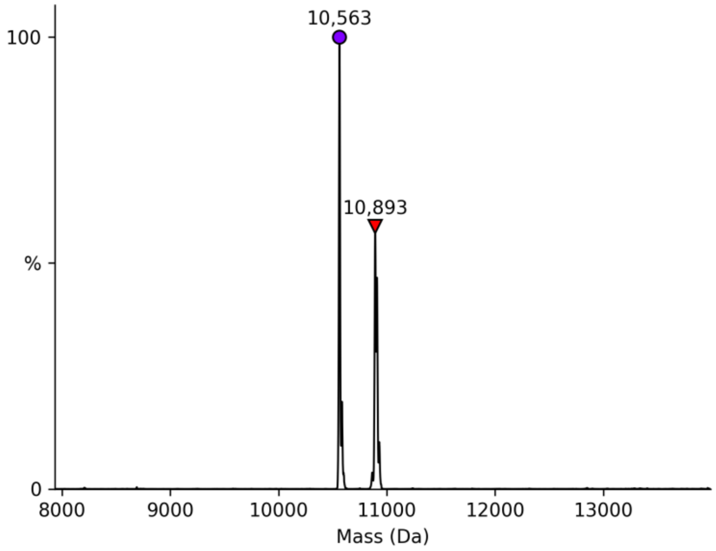

Figure S244. 31RNA_nat, 2nd replicate, deconvoluted spectrum, calculated mass: 10566.0 Da, found mass: 10563.0 Da (product); found mass: 10893.0 Da (product + rAMP).

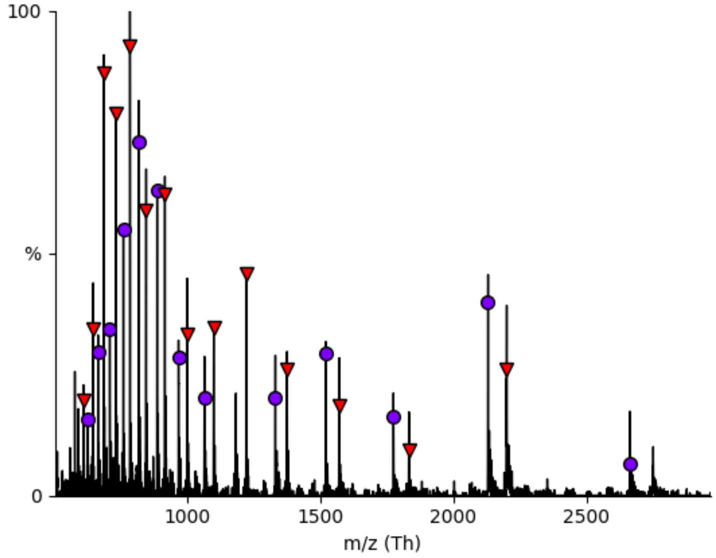

Figure S245. 31RNA_4A^{E}, 1st replicate, raw spectrum.

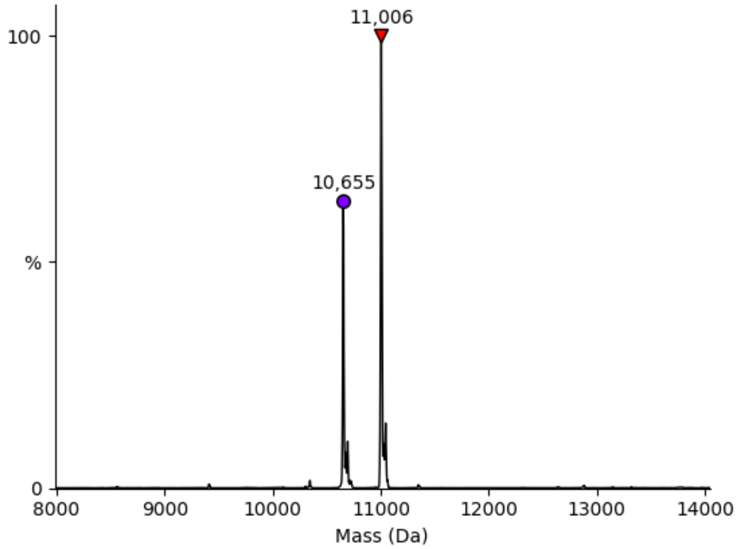

Figure S246. 31RNA_4A^{E}, 1st replicate, deconvoluted spectrum, calculated mass: 10658.1 Da, found mass: 10655.0 Da (product); found mass: 11006.0 Da (product + **rA^{E}MP**).

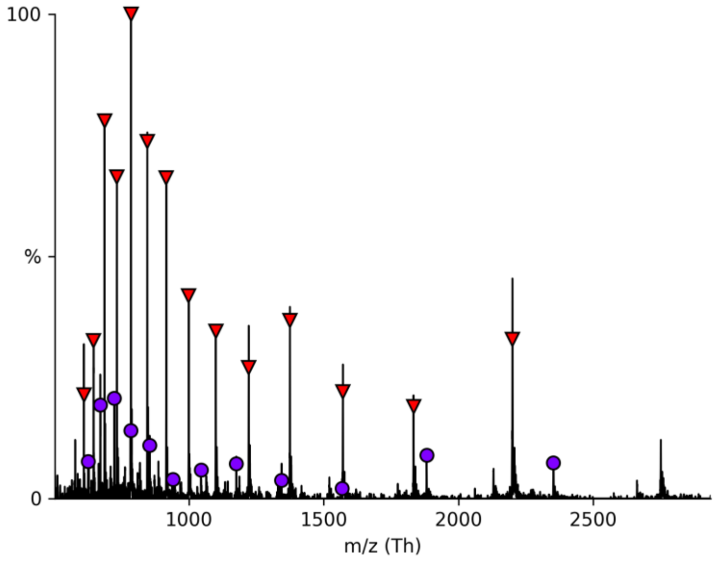

Figure S247. 31RNA_4A^{E}, 2nd replicate, raw spectrum.

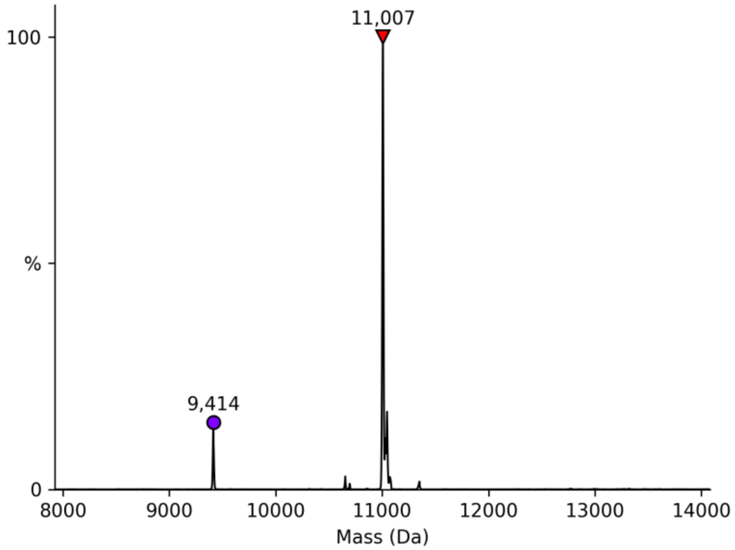

Figure S248. 31RNA_4A^{E}, 2nd replicate, deconvoluted spectrum, calculated mass: 10658.1 Da, found mass: 11007.0 Da (product + **rA^{E}MP**).

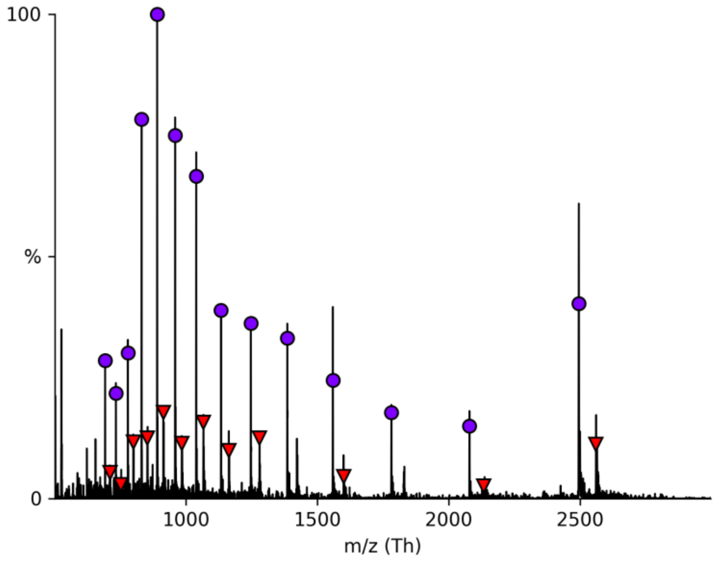

Figure S249. 31RNA_4U^{Bio}, 1st replicate, raw spectrum.

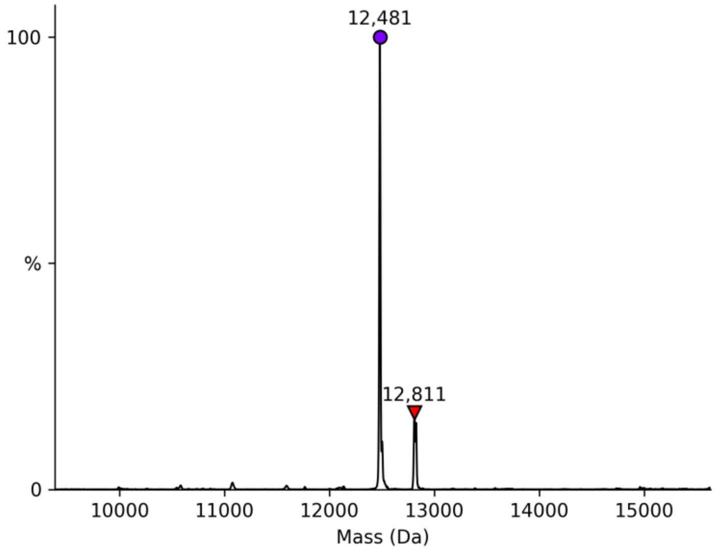

Figure S250. 31RNA_4U^{Bio}, 1st replicate, deconvoluted spectrum, calculated mass: 12484.6 Da, found mass: 12481.0 Da (product).

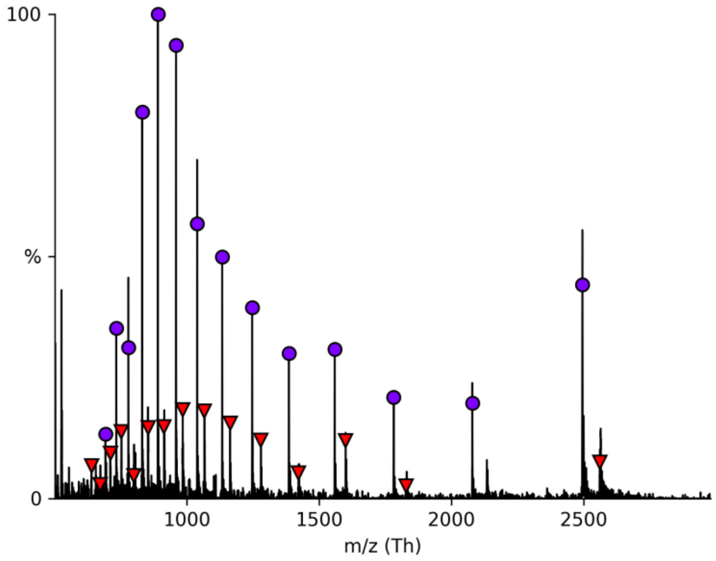

Figure S251. 31RNA_4U^{Bio}, 2nd replicate, raw spectrum.

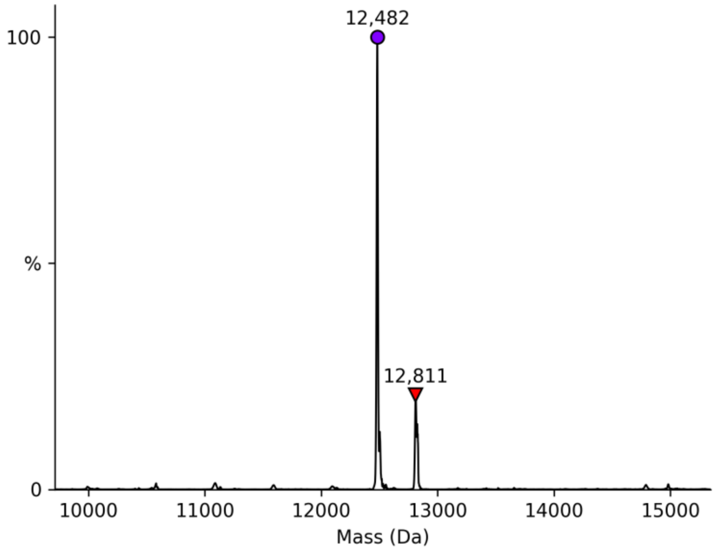

Figure S252. 31RNA_4U^{Bio}, 2nd replicate, deconvoluted spectrum, calculated mass: 12484.6 Da, found mass: 12482.0 Da (product).

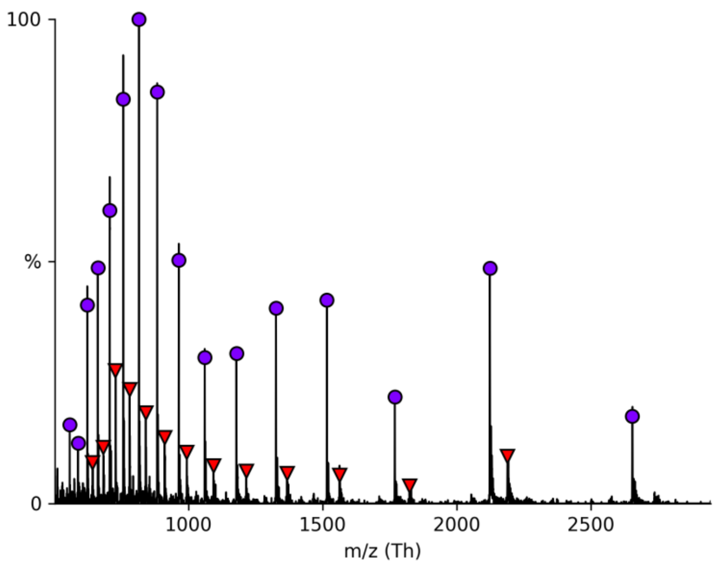

Figure S253. 31RNA_4C^{Me}, 1st replicate, raw spectrum.

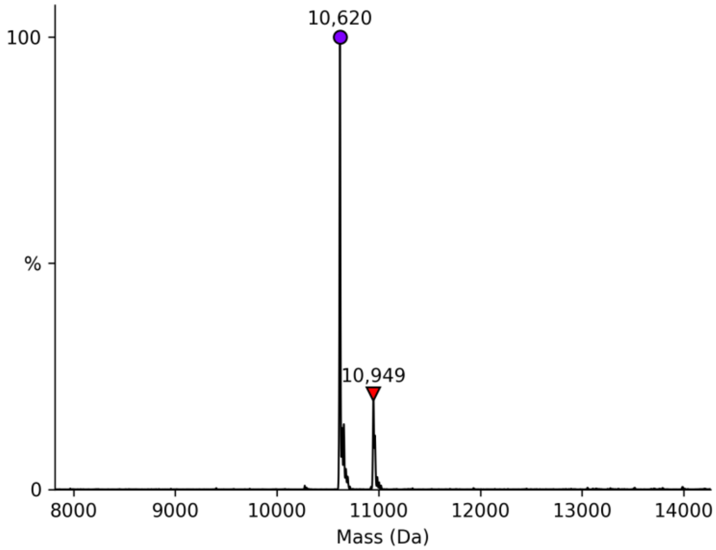

Figure S254. 31RNA_4C^{Me}, 1st replicate, deconvoluted spectrum, calculated mass: 10622.1 Da, found mass: 10620.0 Da (product).

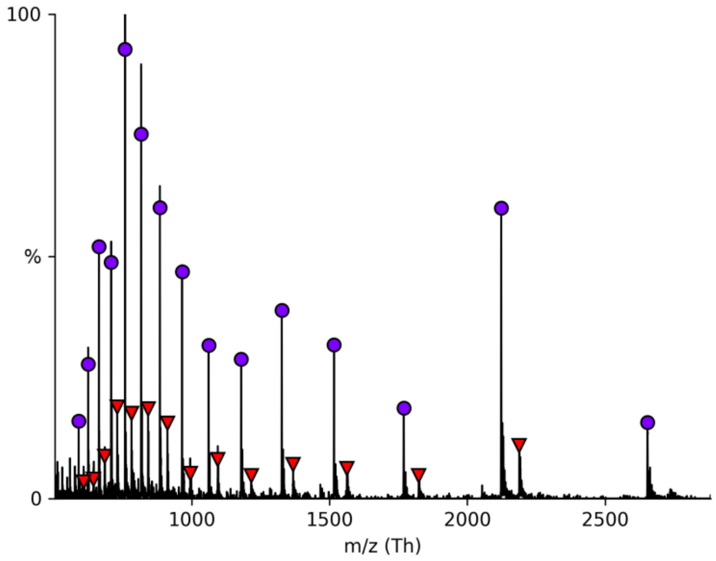

Figure S255. 31RNA_4C^{Me}, 2nd replicate, raw spectrum.

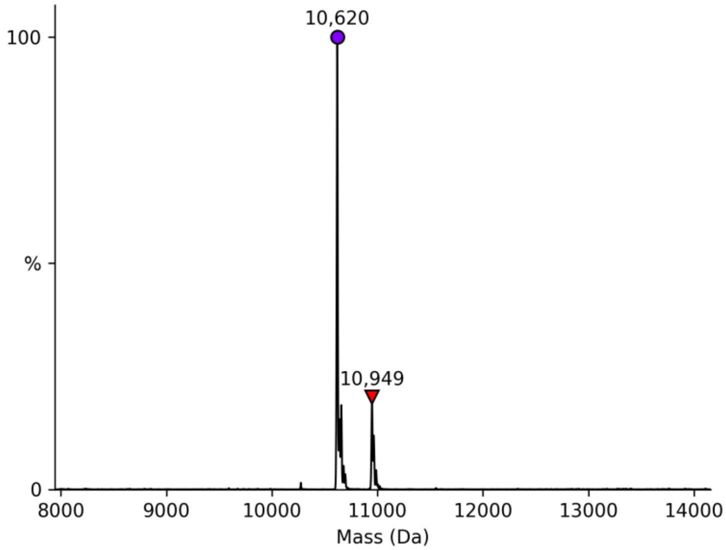

Figure S256. 31RNA_4C^{Me}, 2nd replicate, deconvoluted spectrum, calculated mass: 10622.1 Da, found mass: 10620.0 Da (product).

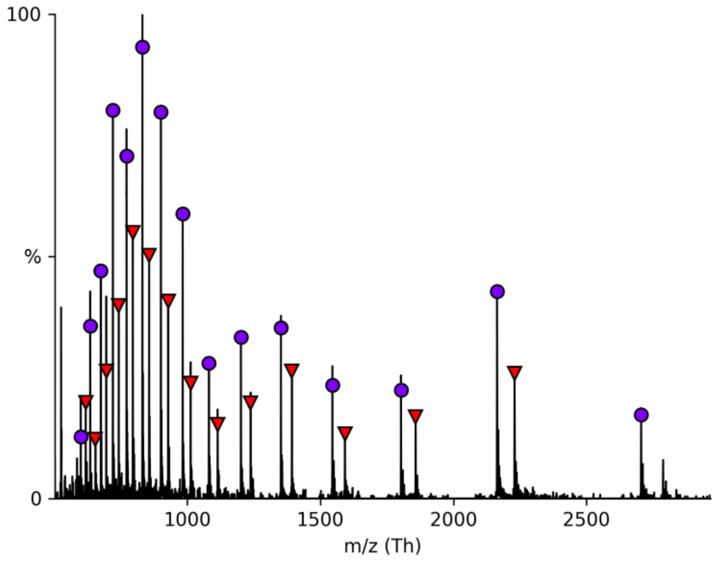

Figure S257. 31RNA_4G^{Pent}, 1st replicate, raw spectrum.

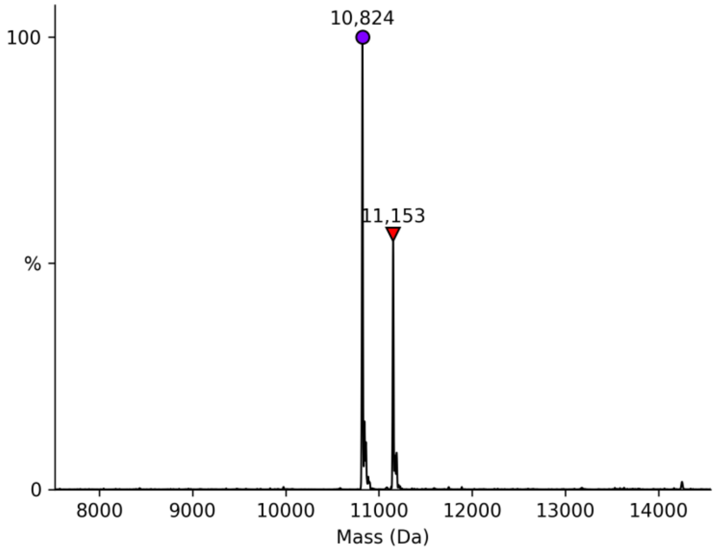

Figure S258. 31RNA_4G^{Pent}, 1st replicate, deconvoluted spectrum, calculated mass: 10826.5 Da, found mass: 10824.0 Da (product); found mass: 11153.0 Da (product + rAMP).

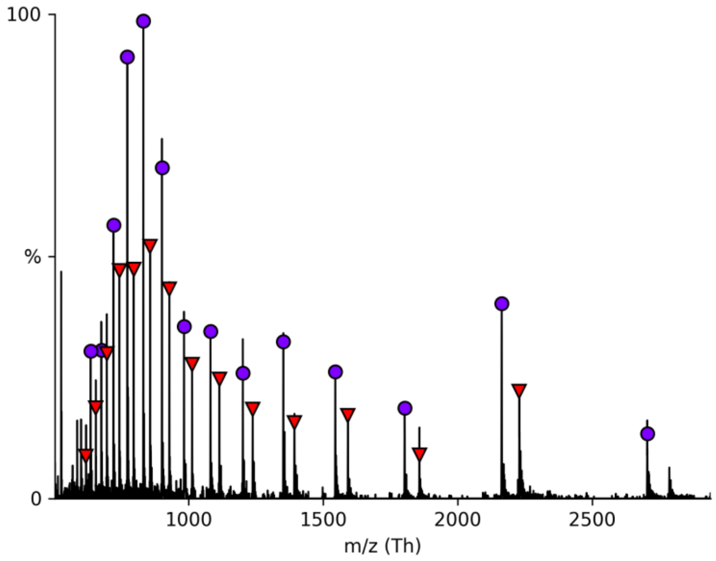

Figure S259. 31RNA_4G^{Pent}, 2nd replicate, raw spectrum.

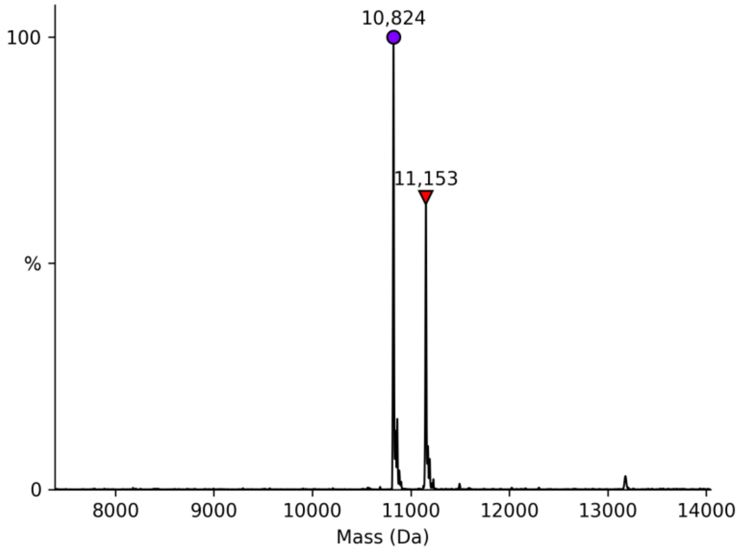

Figure S260. 31RNA_4G^{Pent}, 2nd replicate, deconvoluted spectrum, calculated mass: 10826.5 Da, found mass: 10824.0 Da (product); found mass: 11153.0 Da (product + rAMP).

4.3 LC-MS chromatograms

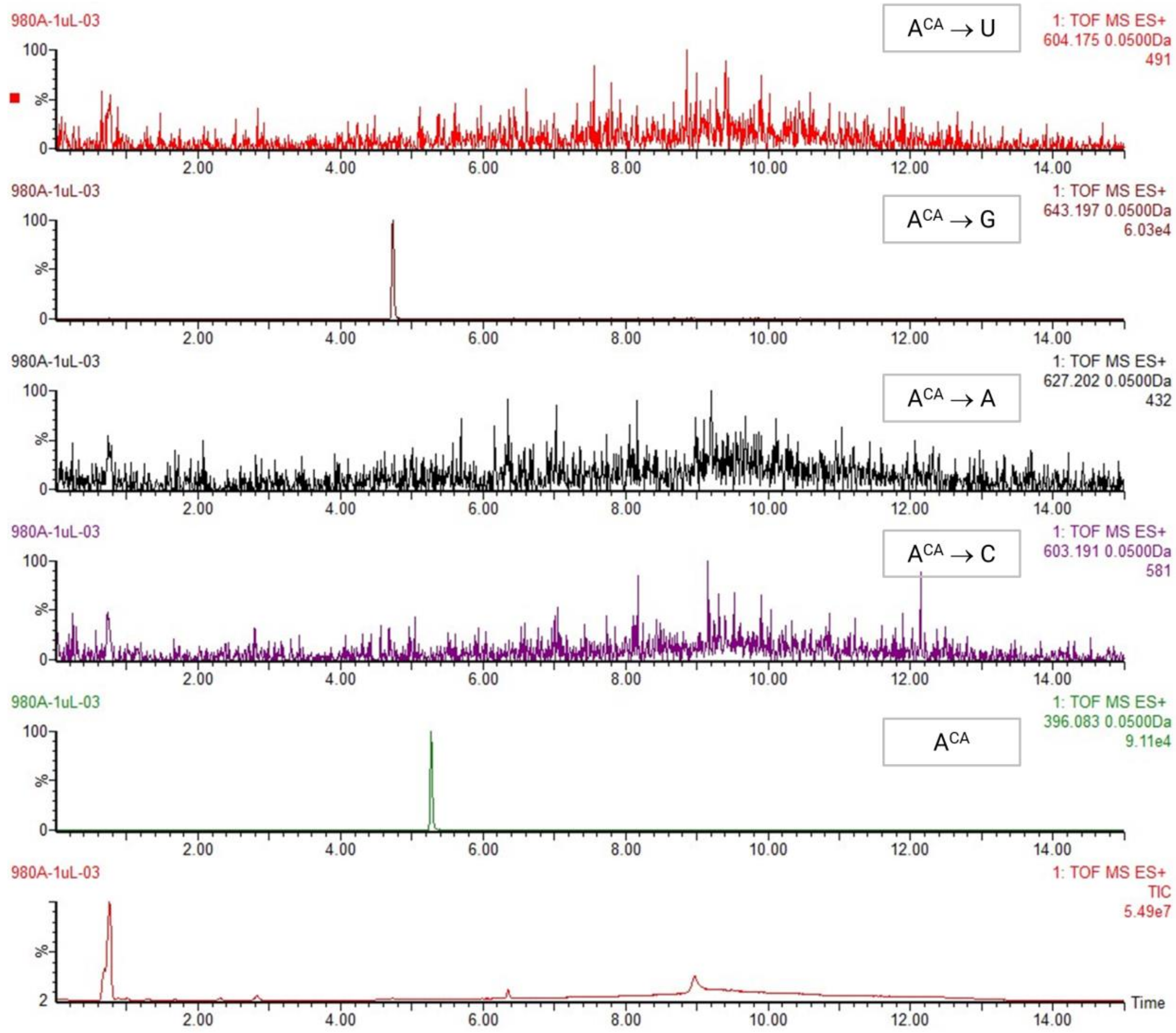

Figure S261. Chromatogram from oligonucleotide **19RNA_A^{CA}** digest prepared according to section 2.8.2. Extracted mass chromatograms for dinucleoside conjugates of **A^{CA}** to putative indicated nucleosides generated after RNA digestion. Lowest panel represents TIC chromatogram.

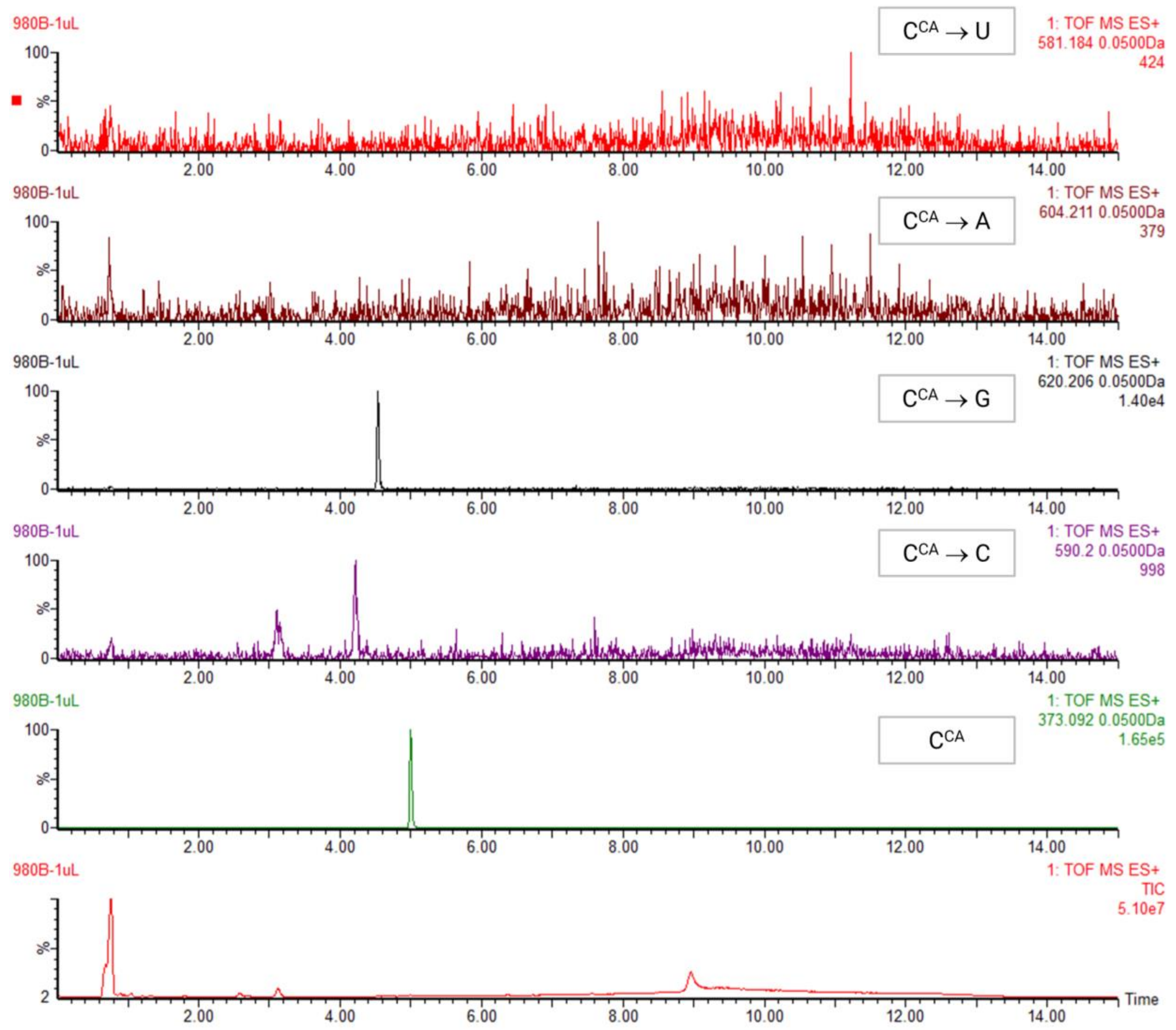

Figure S262. Chromatogram from oligonucleotide **19RNA_C^{CA}** digest prepared according to section 2.8.12. Extracted mass chromatograms for dinucleoside conjugates of **C^{CA}** to putative indicated nucleosides generated after RNA digestion. Lowest panel represents TIC chromatogram.

5 Sanger sequencing

5.1 Raw Sanger sequencing data

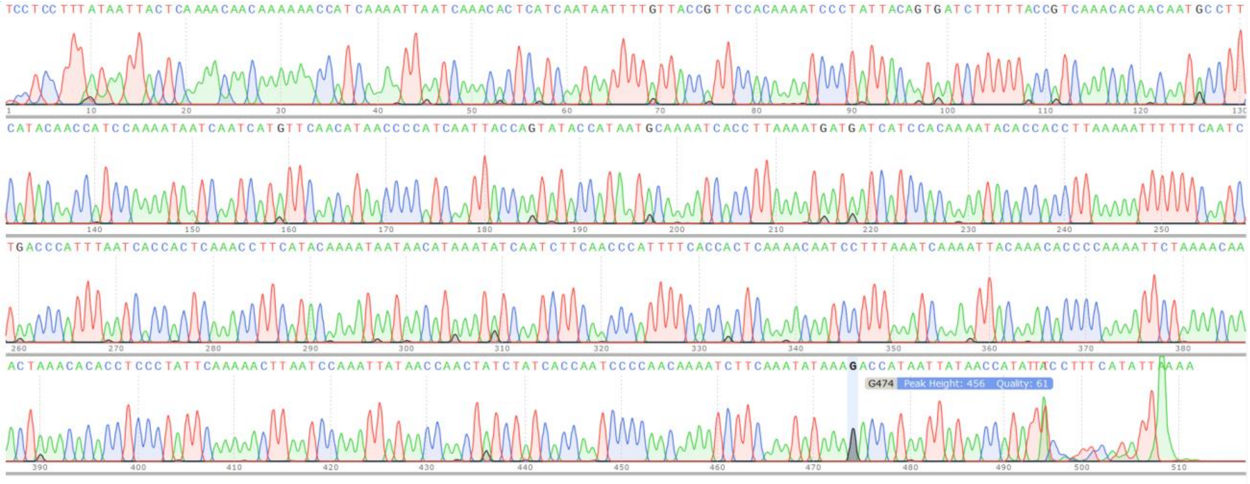

Figure S263. Sequencing data from **mRNA-init**.

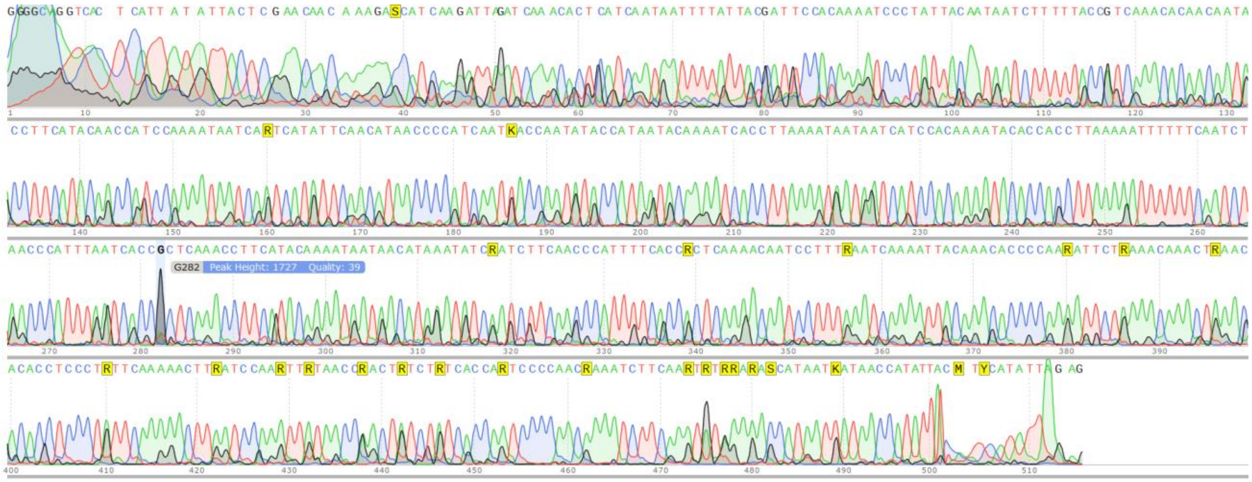

Figure S264. Sequencing data from **mRNA-mid**.

6 Additional information

6.1 Plasmid sequence (5´→3´ direction)

GACTCACTATAGGGCCCCTCTCCCTCCCCCCCCCTAACGTTACTGGCCGAAGCCGCTTGGAAT
AAGGCCGGTGTGCGTTTGTCTATATGTTATTTTCCACCATATTGCCGTCTTTTGGCAATGTGAG
GGCCCGGAAACCTGGCCCTGTCTTCTTGACGAGCATTCCTAGGGGTCTTTCCCCTCTCGCCAAA
GGAATGCAAGGTCTGTTGAATGTCGTGAAGGAAGCAGTTCCTCTGGAAGCTTCTTGAAGACAAA
CAACGTCTGTAGCGACCCTTTGCAGGCAGCGGAACCCCCCACCTGGCGACAGGTGCCTCTGCGG
CCAAAAGCCACGTGTATAAGATACACCTGCAAAGGCGGCACAACCCCAGTGCCACGTTGTGAGT
TGGATAGTTGTGGAAAGAGTCAAATGGCTCTCCTCAAGCGTATTCAACAAGGGGCTGAAGGATG
CCCAGAAGGTACCCCATTGTATGGGATCTGATCTGGGGCCTCGGTGCACATGCTTTACATGTGT
TTAGTCGAGGTTAAAAAAACGTCTAGGCCCCCCGAACCACGGGGACGTGGTTTTCCTTTGAAAA
ACACGATGATAATATGGCCACAACCATGGTCTTCACACTCGAAGATTTCGTTGGGGACTGGCGA
CAGACAGCCGGCTACAACCTGGACCAAGTCCTTGAACAGGGAGGTGTGTCCAGTTTGTTTCAGA
ATCTCGGGGTGTCCGTAACTCCGATCCAAAGGATTGTCCTGAGCGGTGAAAATGGGCTGAAGAT
CGACATCCATGTCATCATCCCGTATGAAGGTCTGAGCGGCGACCAAATGGGCCAGATCGAAAAA
ATTTTTAAGGTGGTGTACCCTGTGGATGATCATCACTTTAAGGTGATCCTGCACTATGGCACAC
TGGTAATCGACGGGGTTACGCCGAACATGATCGACTATTTCGGACGGCCGTATGAAGGCATCGC
CGTGTTCGACGGCAAAAAGATCACTGTAACAGGGACCCTGTGGAACGGCAACAAAATTATCGAC
GAGCGCCTGATCAACCCCGACGGCTCCCTGCTGTTCCGAGTAACCATCAACGGAGTGACCGGCT
GGCGGCTGTGCGAACGCATTCTGGCGTAAAAAAAAAAAAAAAAAAAAAAAAAAAAAAATGAAG
AGCCGTACGGGCGCGCCTAGGCGATTCCGCTTCCTCGCTCACTGACTCGCTGCGCTCGGTCG
TTCGGCTGCGGCGAGCGGTATCAGCTCACTCAAAGGCGGTAATACGGTTATCCACAGAATCAGG
GGATAACGCAGGAAAGAACATGTGAGCAAAAGGCCAGCAAAAGGCCAGGAACCGTAAAAAGGCC
GCGTTGCTGGCGTTTTTCCATAGGCTCCGCCCCCCTGACGAGCATCACAAAAATCGACGCTCAA
GTCAGAGGTGGCGAAACCCGACAGGACTATAAAGATACCAGGCGTTTCCCCCTGGAAGCTCCCT
CGTGCGCTCTCCTGTTCCGACCCTGCCGCTTACCGGATACCTGTCCGCCTTTCTCCCTTCGGGA
AGCGTGGCGCTTTCTCATAGCTCACGCTGTAGGTATCTCAGTTCGGTGTAGGTCGTTCGCTCCA
AGCTGGGCTGTGTGCACGAACCCCCCGTTCAGCCCGACCGCTGCGCCTTATCCGGTAACTATCG
TCTTGAGTCCAACCCGGTAAGACACGACTTATCGCCACTGGCAGCAGCCACTGGTAACAGGATT
AGCAGAGCGAGGTATGTAGGCGGTGCTACAGAGTTCTTGAAGTGGTGGCCTAACTACGGCTACA
CTAGAAGAACAGTATTTGGTATCTGCGCTCTGCTGAAGCCAGTTACCTTCGGAAAAAGAGTTGG
TAGCTCTTGATCCGGCAAACAAACCACCGCTGGTAGCGGTGGTTTTTTTGTTTGCAAGCAGCAG
ATTACGCGCAGAAAAAAAGGATCTCAAGAAGATCCTTTGATCTTTTCTACGGGGTCTGACGCTC
AGTGGAACGAAAACTCACGTTAAGGGATTTTGGTCATGAGATTATCAAAAAGGATCTTCACCTA
GATCCTTTTAAATTAAAAATGAAGTTTTAAATCAATCTAAAGTATATATGAGTAAACTTGGTCT
GACAGTTACCAATGCTTAATCAGTGAGGCACCTATCTCAGCGATCTGTCTATTTCGTTCATCCA
TAGTTGCCTGACTCCCCGTCGTGTAGATAACTACGATACGGGAGGGCTTACCATCTGGCCCCAG
TGCTGCAATGATACCGCGAGATCCACGCTCACCGGCTCCAGATTTATCAGCAATAAACCAGCCA
GCCGGAAGGGCCGAGCGCAGAAGTGGTCCTGCAACTTTATCCGCCTCCATCCAGTCTATTAATT
GTTGCCGGGAAGCTAGAGTAAGTAGTTCGCCAGTTAATAGTTTGCGCAACGTTGTTGCCATTGC
TACAGGCATCGTGGTGTCACGCTCGTCGTTTGGTATGGCTTCATTCAGCTCCGGTTCCCAACGA

TCAAGGCGAGTTACATGATCCCCCATGTTGTGCAAAAAAGCGGTTAGCTCCTTCGGTCCTCCGA
TCGTTGTCAGAAGTAAGTTGGCCGCAGTGTTATCACTCATGGTTATGGCAGCACTGCATAATTC
TCTTACTGTCATGCCATCCGTAAGATGCTTTTCTGTGACTGGTGAGTACTCAACCAAGTCATTC
TGAGAATAGTGTATGCGGCGACCGAGTTGCTCTTGCCCGGCGTCAATACGGGATAATACCGCGC
CACATAGCAGAACTTTAAAAGTGCTCATCATTGGAAAACGTTCTTCGGGGCGAAAACTCTCAAG
GATCTTACCGCTGTTGAGATCCAGTTCGATGTAACCCACTCGTGCACCCAACTGATCTTCAGCA
TCTTTTACTTTCACCAGCGTTTCTGGGTGAGCAAAAACAGGAAGGCAAAATGCCGCAAAAAAGG
GAATAAGGGCGACACGGAAATGTTGAATACTCATACTCTTCCTTTTTCAATATTATTGAAGCAT
TTATCAGGGTTATTGTCTCATGAGCGGATACATATTTGAATGTATTTAGAAAAATAAACAAATA
GGGGTTCCGCGCACATTTCCCCGAAAAGTGCCACCTGACGTCTAAGAAACCATTATTATCATGA
CATTAACCTATAAAAATAGGCGTATCACGAGGCCCTTTCGTCTAATAC

IRES coding part

nLuc gene coding part

C single modification in the initial gene part

C single modification in the middle gene part

6.2 mRNA coding plasmid construct

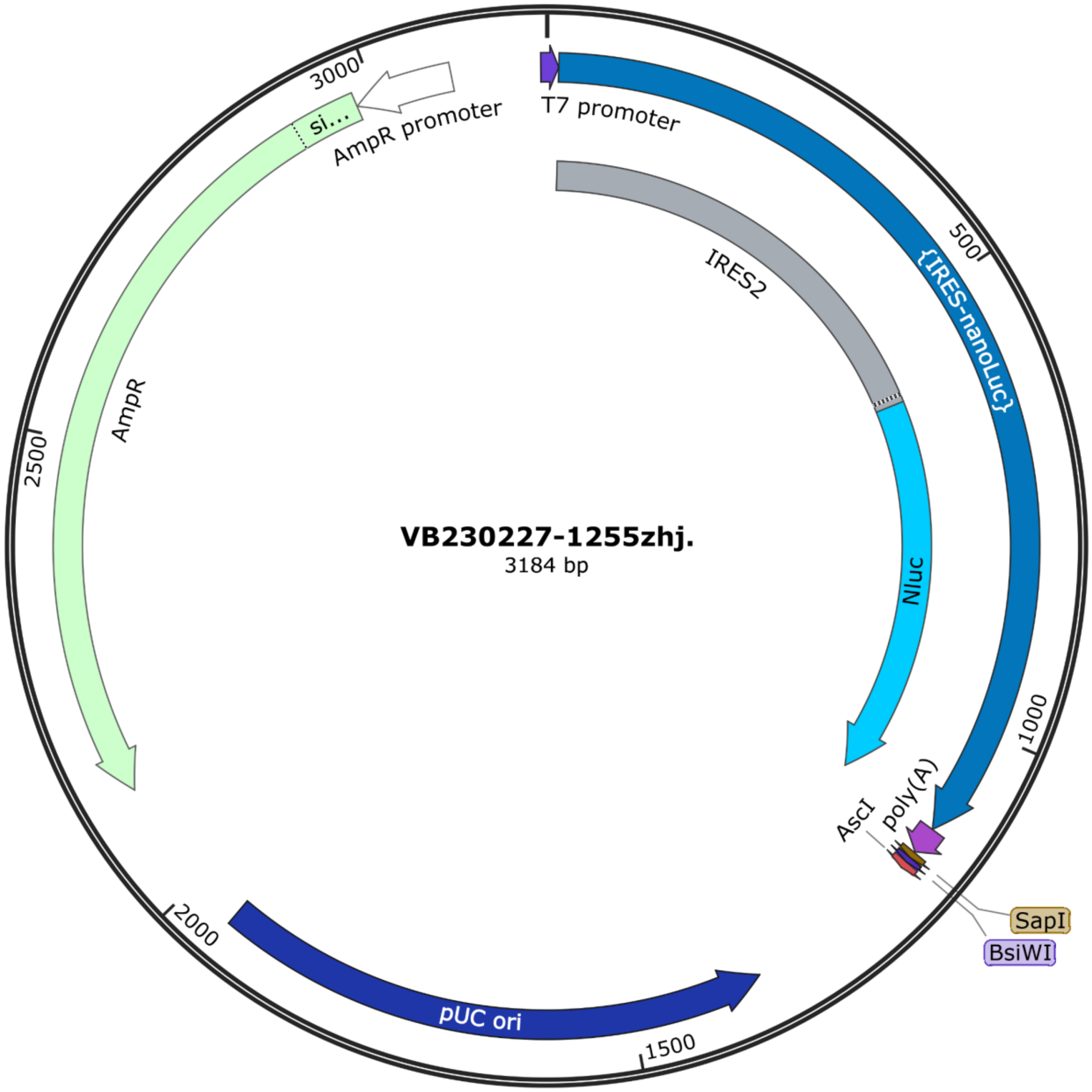

Figure S265. Plasmid construct for generation of DNA templates for mRNA synthesis.

6.3 Overview of enzymatic syntheses with engineered DNA polymerases and T7 RNAP

A

Modified rN^{X}TP	templ_19nt_X (X = A, U, C, G)				5´-(TINA)-templ_31nt or 5´-(dual-Bio)-templ_31nt			
	TGK	Gel/MS	SFM4-3	Gel/MS	TGK	Gel/MS	SFM4-3	Gel/MS
rA^{E}TP	+	+/+	+	+/+	+	+/+	+	+/+
rA^{Pent}TP	+	+/+	+	+/+	+	+/+	+	+/+
rA^{Ph}TP	+	+/+	+	+/+	+	+/+	+	+/+
rC^{E}TP	+	+/+	+	+/+	+	+/+	+	+/+
rC^{Pent}TP	+	+/+	+	+/+	+	+/+	+	+/+
rC^{Ph}TP	+	+/+	+	+/+	+	+/+	+	+/+
rU^{E}TP	+	+/+	+	+/+	+	+/+	+	+/+
rU^{Pent}TP	+	+/+	+	+/+	+	+/+	+	+/+
rU^{Ph}TP	+	+/+	+	+/+	+	+/+	+	+/+
rG^{E}TP	+	+/+	+	+/+	+	+/+	+	+/+
rG^{Pent}TP	+	+/+	+	+/+	+	+/+	+	+/+
rG^{Ph}TP	+	+/+	+	+/+	+	+/+	+	+/+
rC^{Me}TP	+	+/+	N/A	N/A	+	+/+	N/A	N/A
rC^{mBdp}TP	+	+/+	+	+/+	+	+/+	+	+/+
rC^{Cy5}TP	+	+/+	+	+/+	N/A	N/A	N/A	N/A
rU^{Bio}TP	+	+/+	+	+/+	+	+/+	+	+/+
rU^{Dig}TP	+	+/+	+	+/+	+	+/+	+	+/+
rA^{CA}TP	+	+/+	+	+/+	N/A	N/A	N/A	N/A
rC^{CA}TP	+	+/+	+	+/+	N/A	N/A	N/A	N/A
rU^{CA}TP	+	+/+	+	+/+	N/A	N/A	N/A	N/A
rA^{FT}TP	+	+/+	+	+/+	N/A	N/A	N/A	N/A
rC^{FT}TP	+	+/+	+	+/+	N/A	N/A	N/A	N/A
rU^{FT}TP	+	+/+	+	+/+	N/A	N/A	N/A	N/A

B

rA^{E}TP, rU^{Bio}TP, rC^{Ph}TP, rG^{Pent}TP	templ_19nt_mix			
	TGK	Gel/MS	SFM4-3	Gel/MS
	+	+/+	*	+/-
	5´-(TINA)-templ_31nt			
	TGK	Gel/MS	SFM4-3	Gel/MS
	+	+/+	*	+/-
	templ_65nt			
	TGK	Gel/MS	SFM4-3	Gel/MS
	+	+/+	-	+/-
	templ_98nt			
	TGK	Gel/RT-qPCR	SFM4-3	Gel/MS
	+	+/+	-	+/-
rA^{E}TP, rU^{E}TP, rC^{Me}TP, rG^{Pent}TP	templ_IRES			
	TGK		Gel/RT-qPCR	
	+		+/+	
	templ_IRES-prolonged			
	TGK		Gel/RT-qPCR	
	+		+/+	
	templ_IRES-nLuc			
	TGK		Gel/RT-qPCR	
	*		+/+	

E

	TGK	Gel/MS
mRNA	+	+/-
Cy5-Cy3-riboswitch	+	-/+
FAM-Cy5-Cy3-riboswitch	+	+/+
RNA with cleavable DNA primer	+	+/+

F

rATP, rUTP, rCTP, rGTP	templ_98nt – thermal cycling	
	TGK	Gel/MS
	+	+/-
rATP, rUTP, rC^{Me}TP, rGTP	templ_98nt – thermal cycling	
	TGK	Gel/MS
	+	+/-
rATP, rUTP, rCTP, rGTP	templ_(dual-Bio)-IRES-nLuc – solid phase recycling	
	TGK	Gel/MS
	+	+/-
rATP, rUTP, rC^{Me}TP, rGTP	templ_(dual-Bio)-IRES-nLuc – solid phase recycling	
	TGK	Gel/MS
	+	+/-

C

	templ_poly-U				ds-templ_poly-U	
	TGK	Gel/MS	SFM4-3	Gel/MS	T7 RNAP	Gel/MS
rU^{Bio}TP	+	+/-	x	+/-	x	+/-
	templ_50nt				ds-templ_52bp	
	TGK	Gel/MS	SFM4-3	Gel/MS	T7 RNAP	Gel/MS
rA^{Ph}TP, rU^{Ph}TP, rC^{Ph}TP, rGTP	+	+/-	*	+/-	-	+/-
rA^{E}TP, rU^{Bio}TP, rC^{Ph}TP, rGTP	+	+/-	*	+/-	+	+/-
rA^{Ph}TP, rU^{Bio}TP, rC^{mBdp}TP, rGTP	+	+/-	-	+/-	-	+/-

D

rC^{mBdp}TP	templ_16nt – time series			
	TGK	Gel/MS	SFM4-3	Gel/MS
	+	+/-	+	+/-
rC^{mBdp}TP	templ_16nt – concentration series			
	TGK	Gel/MS	SFM4-3	Gel/MS
	+	+/-	+	+/-
rC^{mBdp}TP, rATP, rUTP, rGTP	templ_16nt – fidelity assay			
	TGK	Gel/MS	SFM4-3	Gel/MS
	+	+/-	+	+/-

Table legend

+	full-length product formation
*	traces of full-length product
-	no full-length product
x	failed also with natural rNTPs in synthesis of natural RNA
N/A	not assessed
Gel/MS = +/+	product characterized by gel electrophoresis and MS analysis
Gel/MS = +/-	product characterized only by gel electrophoresis
Gel/MS = -/+	product characterized only by MS analysis

Figure S266. A, PEX with two screening templates encoding for one or four modified sites performed with both engineered DNA polymerases. **B**, PEX with all four base-modified **rN^{X}TP**s on templates of increasing length. **C**, PEX with both engineered DNA polymerases or IVT with T7 RNAP and templates encoding for challenging poly(U) sequences. PEX or IVT with various combinations of three base-modified **rN^{X}TP**s with templates encoding for same synthesized RNA sequence. **D**, Engineered DNA polymerase activity studies including SNI kinetics, polymerase titration and competitive fidelity assay. **E**, Applications of methodology to long mRNA synthesis, two-site- or three-site-labelled structured functional riboswitch and to synthesis of primer-free RNA probe. **F**, Amplification PEX or solid-phase PEX for generation of super-stoichiometric amounts of either natural or modified RNA probes. All experiments are in detail summarised in Supplementary Data Section 2 (Biochemical part).

7 Abbreviations and symbols used in this study

DEPC	Diethyl pyrocarbonate
DMEDA	*N*,*N*-Dimethylethylenediamine
dNTPs	deoxyribonucleoside triphosphates
dPAGE	polyacrylamide gel electrophoresis
dsDNA	double-stranded DNA
DTT	Dithiothreitol
EDTA	Ethylenediaminetetraacetic acid
FLC	flash liquid chromatography system
FRET	Förster (fluorescence) resonance energy transfer
HF	high-fidelity
HFIP	1,1,1,3,3,3-Hexafluoro-2-propanol
IVT	*in vitro* transcription
oligo-dT_{25}-MB	oligo-$d(T)_{25}$-magnetic beads
PCR	polymerase chain reaction
PEX	primer extension reaction
$POCl_3$	Phosphoryl chloride
$PO(OMe)_3$	Trimethyl phosphate
ppm	parts per million
rNTPs	ribonucleoside triphosphates
**rN^XTP**s	base-modified ribonucleoside triphosphates
rSAP	Shrimp Alkaline Phosphatase
RT	reverse transcription
RT-PCR	reverse transcription polymerase chain reaction
SMB	streptavidin magnetic beads
SNI	single nucleotide incorporation
ssDNA	single-stranded DNA
SSIV RT	SuperScript IV reverse transcriptase
T7 RNAP	T7 RNA polymerase
TAE	TRIS-acetate-EDTA
TBE	TRIS-borate-EDTA

TEA	Triethylamine
TEAB	Triethylammonium bicarbonate
TLC	thin-layer chromatography
TRIS	Tris(hydroxymethyl)aminomethane
UDG	Uracil-DNA Glycosylase

8 References

1. Seela, F. & Peng, X. 7-Functionalized 7-deazapurine ribonucleosides related to 2-aminoadenosine, guanosine, and xanthosine: glycosylation of pyrrolo[2,3-*d*]pyrimidines with 1-*O*-acetyl-2,3,5-tri-*O*-benzoyl-D-ribofuranose. *J. Org. Chem.* **71**, 81–90 (2006).
2. Seela, F. & Ming, X. 7-Functionalized 7-deazapurine β-D and β-L-ribonucleosides related to tubercidin and 7-deazainosine: glycosylation of pyrrolo[2,3-d]pyrimidines with 1-*O*-acetyl-2,3,5-tri-*O*-benzoyl-β-D or β-L-ribofuranose. *Tetrahedron* **63**, 9850–9861 (2007).
3. Rai, D. *et al.* Design and Studies of Novel 5-Substituted Alkynylpyrimidine Nucleosides as Potent Inhibitors of Mycobacteria. *J. Med. Chem.* **48**, 7012–7017 (2005).
4. Brunderová, M. *et al.* Chloroacetamide-Modified Nucleotide and RNA for Bioconjugations and Cross-Linking with RNA-Binding Proteins. *Angew. Chemie Int. Ed.* **62**, e202213764 (2023).
5. Pesnot, T., Tedaldi, L. M., Jambrina, P. G., Rosta, E. & Wagner, G. K. Exploring the role of the 5-substituent for the intrinsic fluorescence of 5-aryl and 5-heteroaryl uracil nucleotides: a systematic study. *Org. Biomol. Chem.* **11**, 6357–6371 (2013).
6. Milisavljevič, N., Perlíková, P., Pohl, R. & Hocek, M. Enzymatic synthesis of base-modified RNA by T7 RNA polymerase. A systematic study and comparison of 5-substituted pyrimidine and 7-substituted 7-deazapurine nucleoside triphosphates as substrates. *Org. Biomol. Chem.* **16**, 5800–5807 (2018).
7. Güixens-Gallardo, P. *et al.* Brightly Fluorescent 2′-Deoxyribonucleoside Triphosphates Bearing Methylated Bodipy Fluorophore for in Cellulo Incorporation to DNA, Imaging, and Flow Cytometry. *Bioconjug. Chem.* **29**, 3906–3912 (2018).
8. Bourderioux, A. *et al.* Synthesis and significant cytostatic activity of 7-hetaryl-7-deazaadenosines. *J. Med. Chem.* **54**, 5498–5507 (2011).
9. Milne, M., Chicas, K., Li, A., Bartha, R. & Hudson, R. H. E. ParaCEST MRI contrast agents capable of derivatizationvia "click" chemistry. *Org. Biomol. Chem.* **10**, 287–292 (2012).
10. Krause, A., Hertl, A., Muttach, F. & Jäschke, A. Phosphine-Free Stille–Migita Chemistry for the Mild and Orthogonal Modification of DNA and RNA. *Chem. – A Eur. J.* **20**, 16613–16619 (2014).
11. Marty, M. T. *et al.* Bayesian deconvolution of mass and ion mobility spectra: From binary interactions to polydisperse ensembles. *Anal. Chem.* **87**, 4370–4376 (2015).
12. Cozens, C., Pinheiro, V. B., Vaisman, A., Woodgate, R. & Holliger, P. A short adaptive path from DNA to RNA polymerases. *Proc. Natl. Acad. Sci. U. S. A.* **109**, 8067–8072 (2012).
13. Dunn, M. R., Otto, C., Fenton, K. E. & Chaput, J. C. Improving Polymerase Activity with Unnatural Substrates by Sampling Mutations in Homologous Protein Architectures. *ACS Chem. Biol.* **11**, 1210–1219 (2016).
14. Chen, T. *et al.* Evolution of thermophilic DNA polymerases for the recognition and amplification of C2′-modified DNA. *Nat. Chem.* **8**, 556–562 (2016).

9 Source data files: uncropped scans of gels in Supplementary Figures

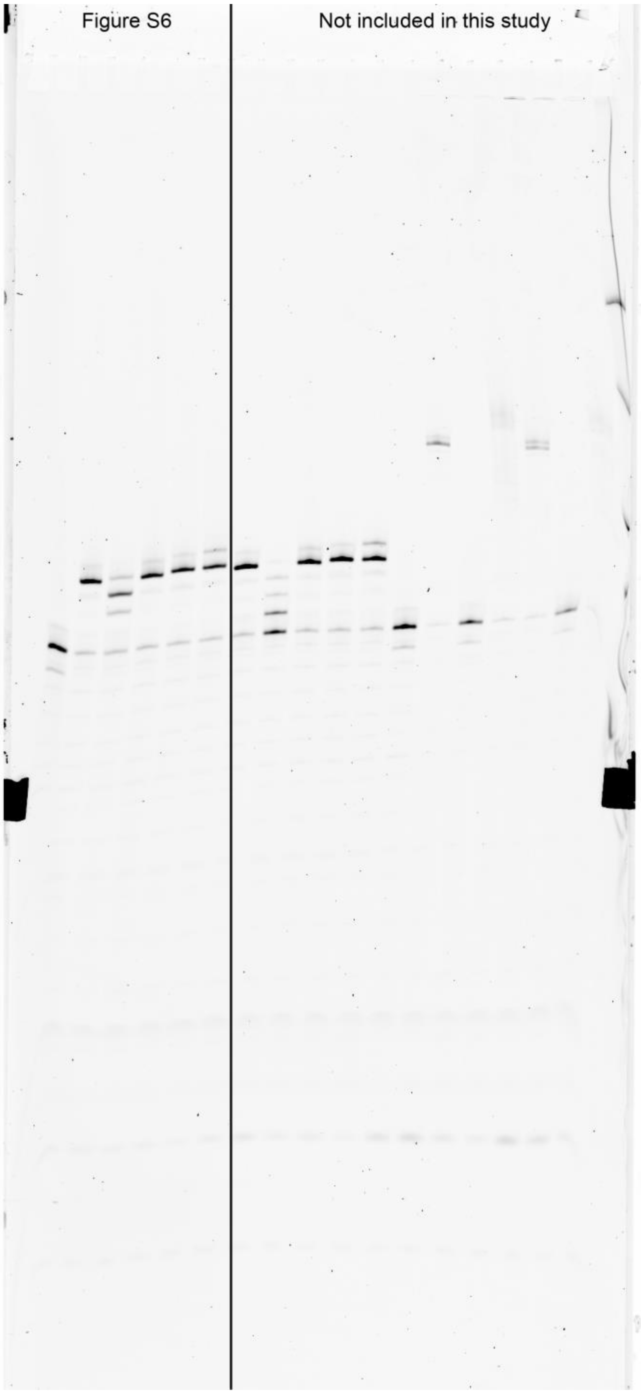

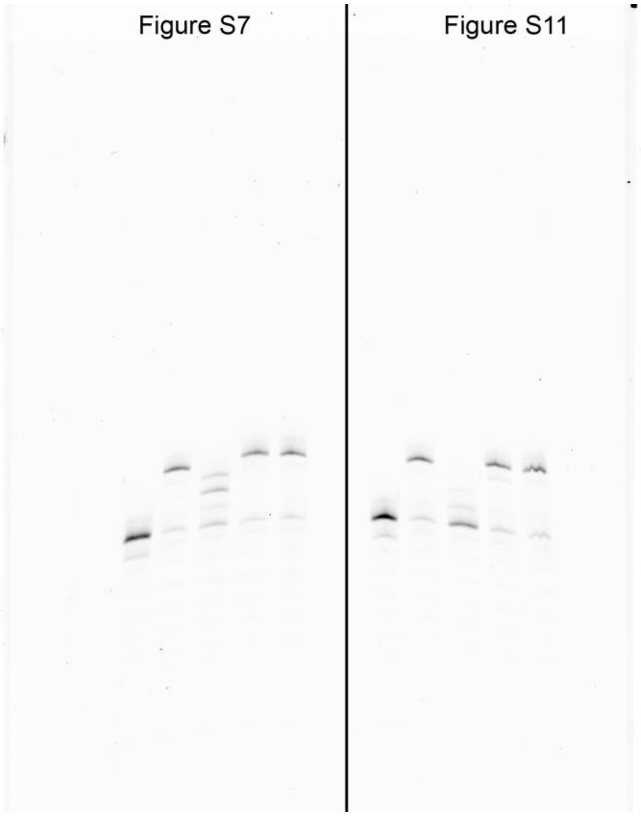
Figure S7
Figure S11

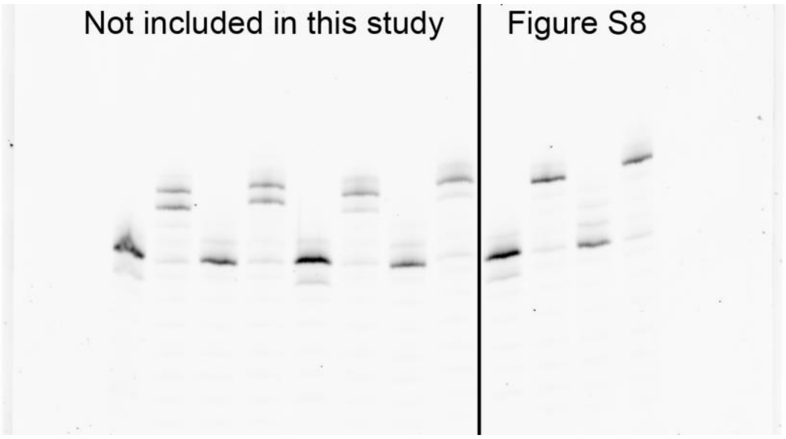
Not included in this study
Figure S8

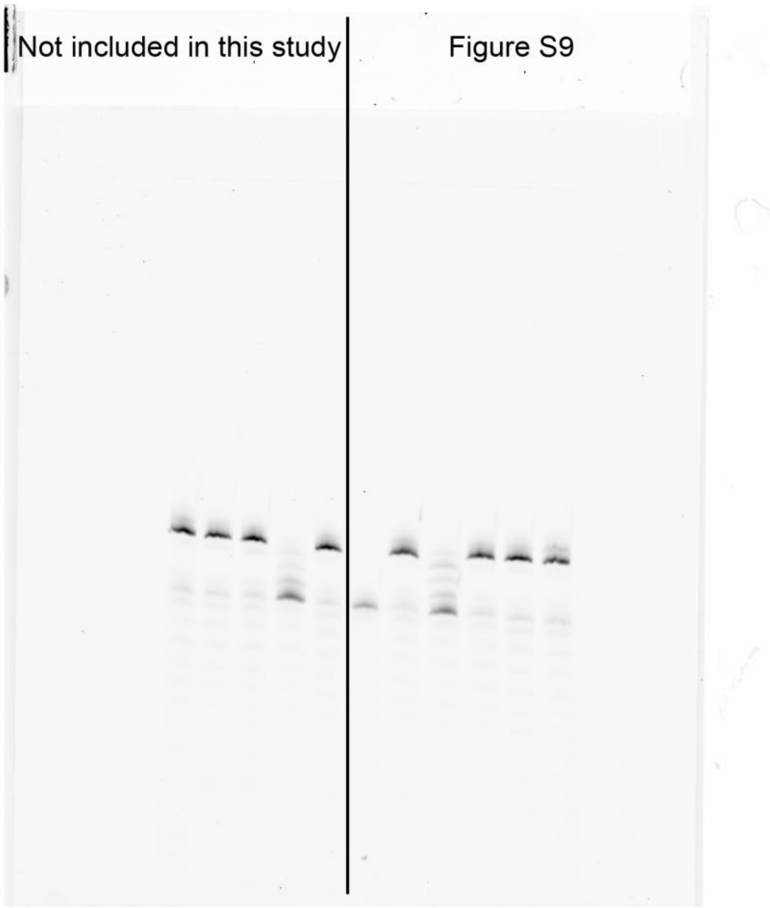
Not included in this study
Figure S9

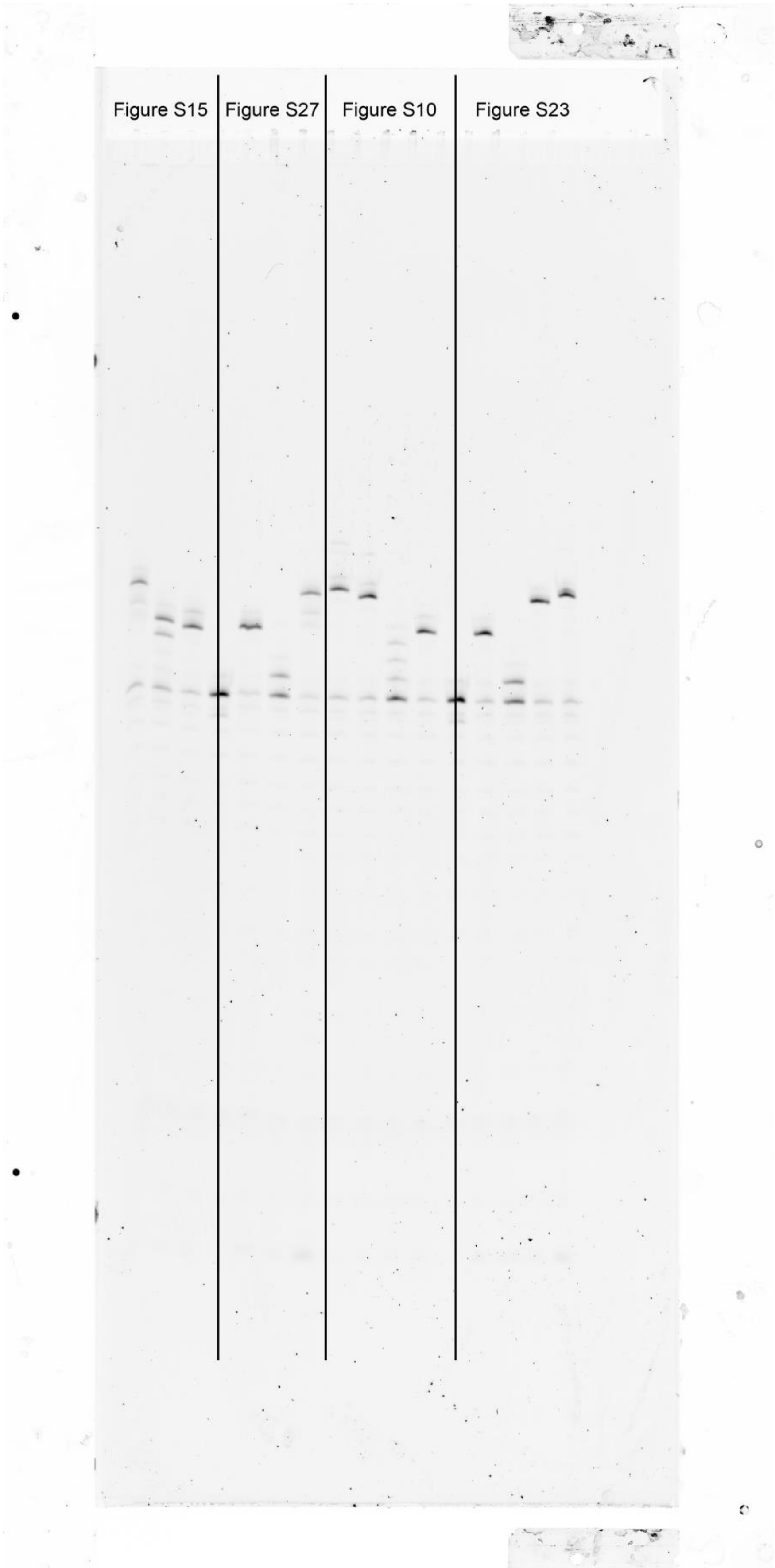
Figure S15
Figure S27
Figure S10
Figure S23

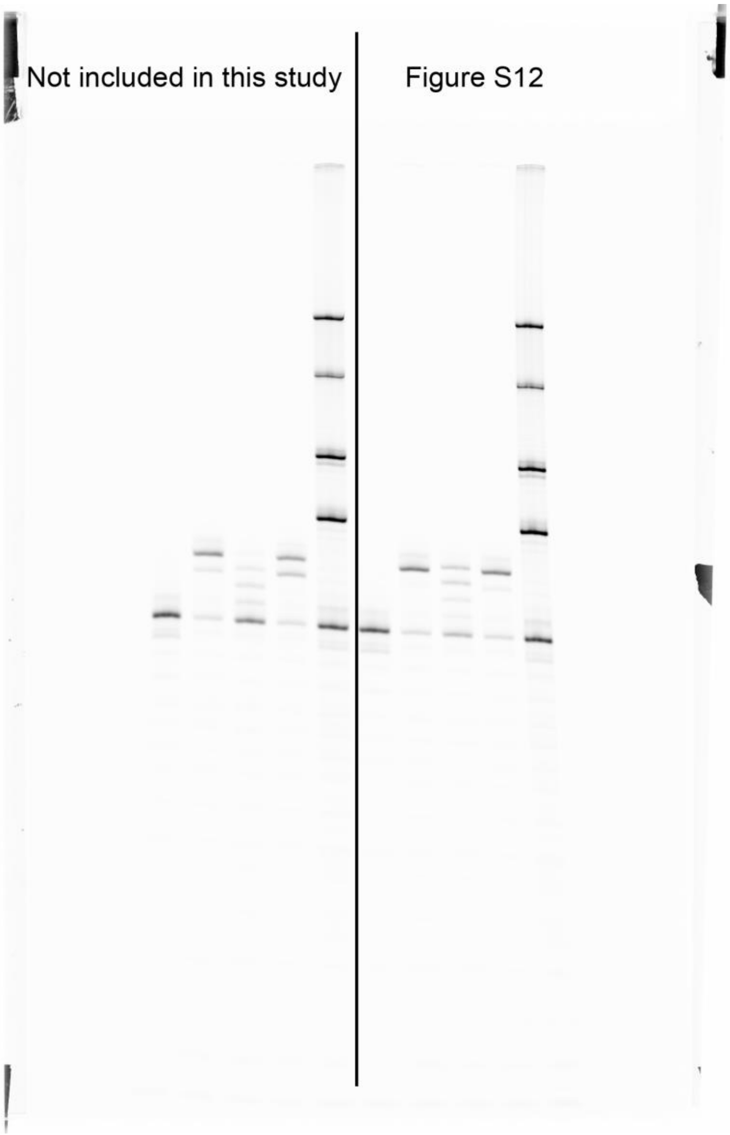
Not included in this study
Figure S12

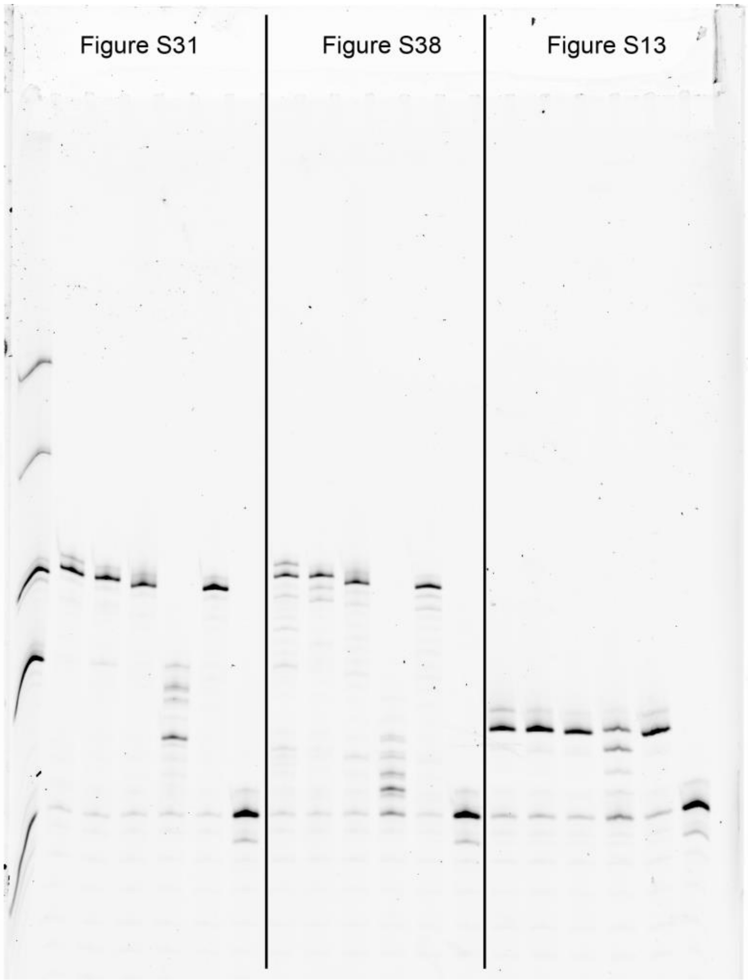
Figure S31
Figure S38
Figure S13

•

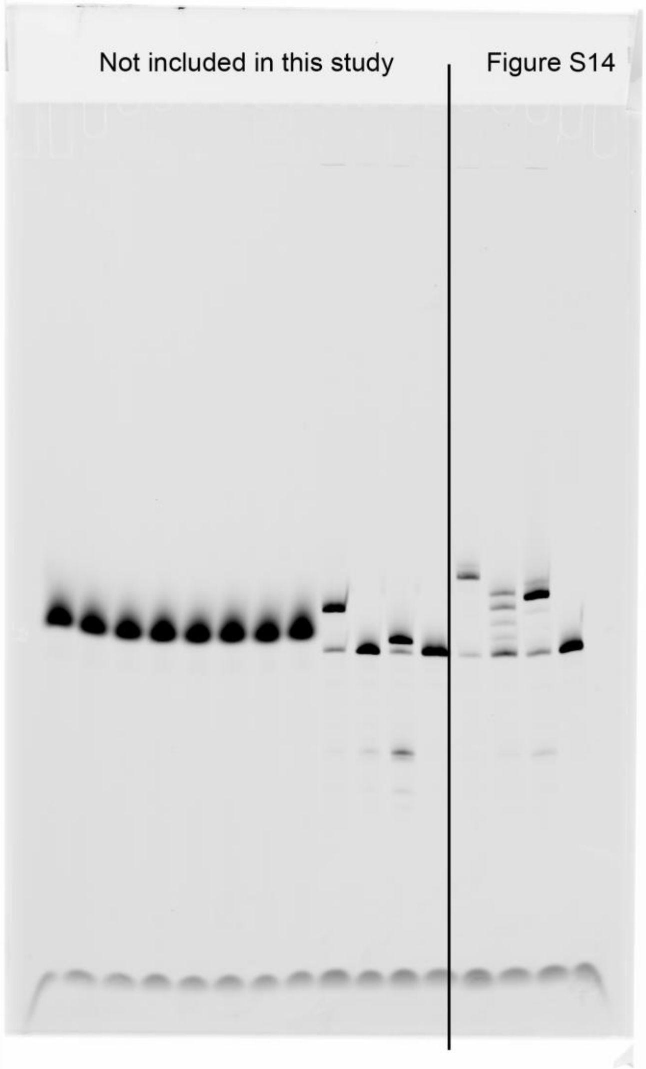

Not included in this study
Figure S14

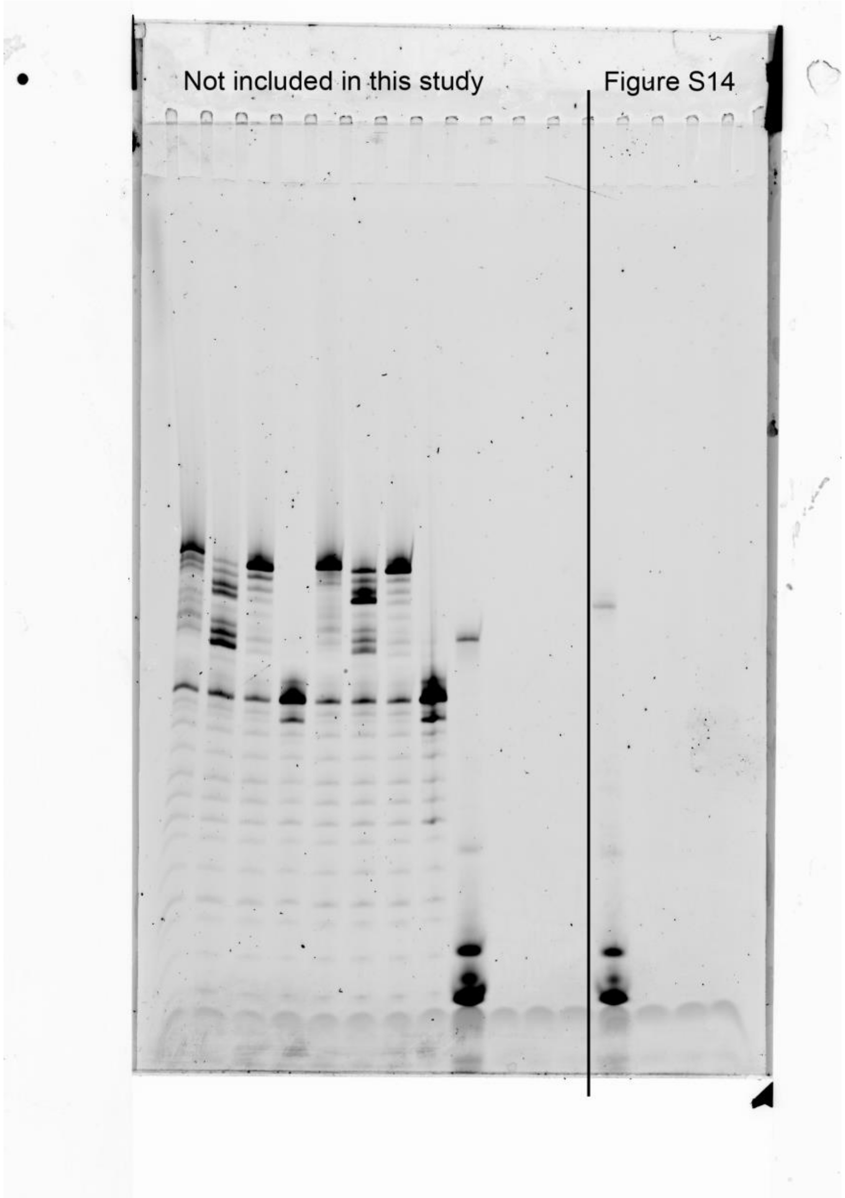
Not included in this study
Figure S14

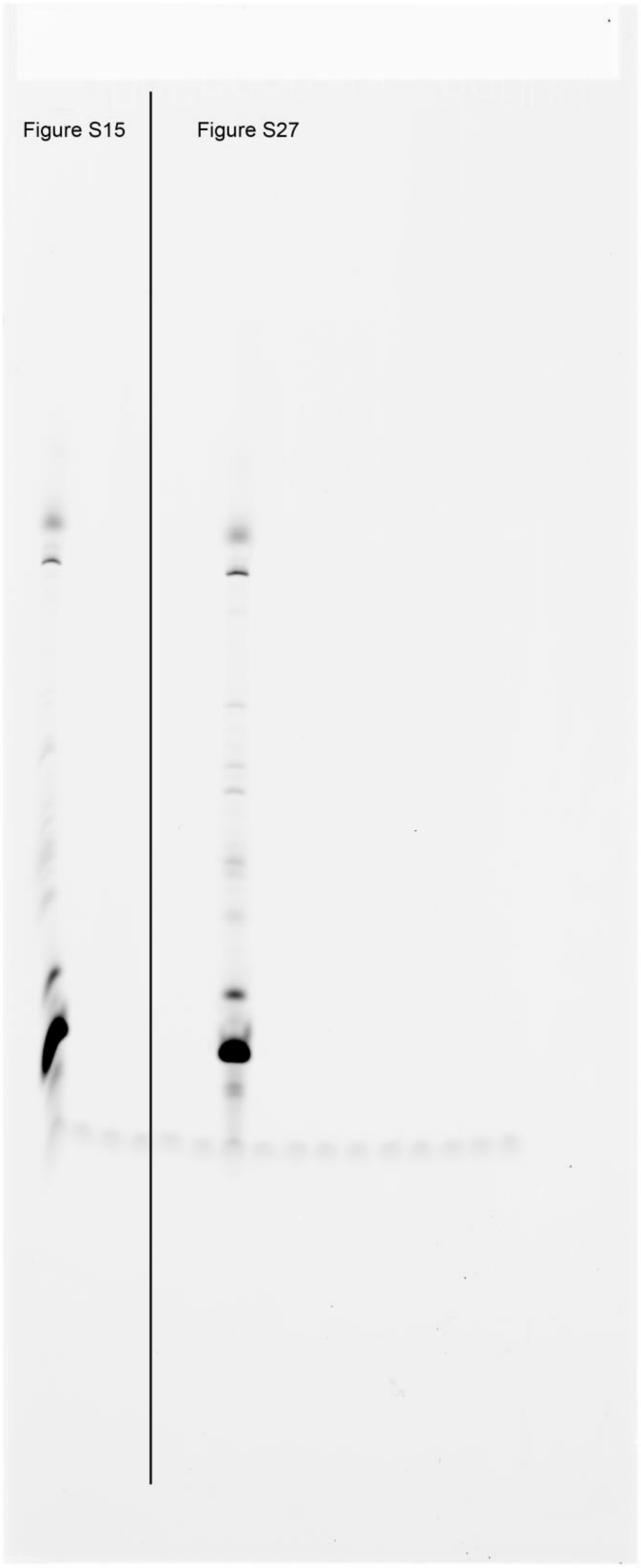
Figure S15
Figure S27

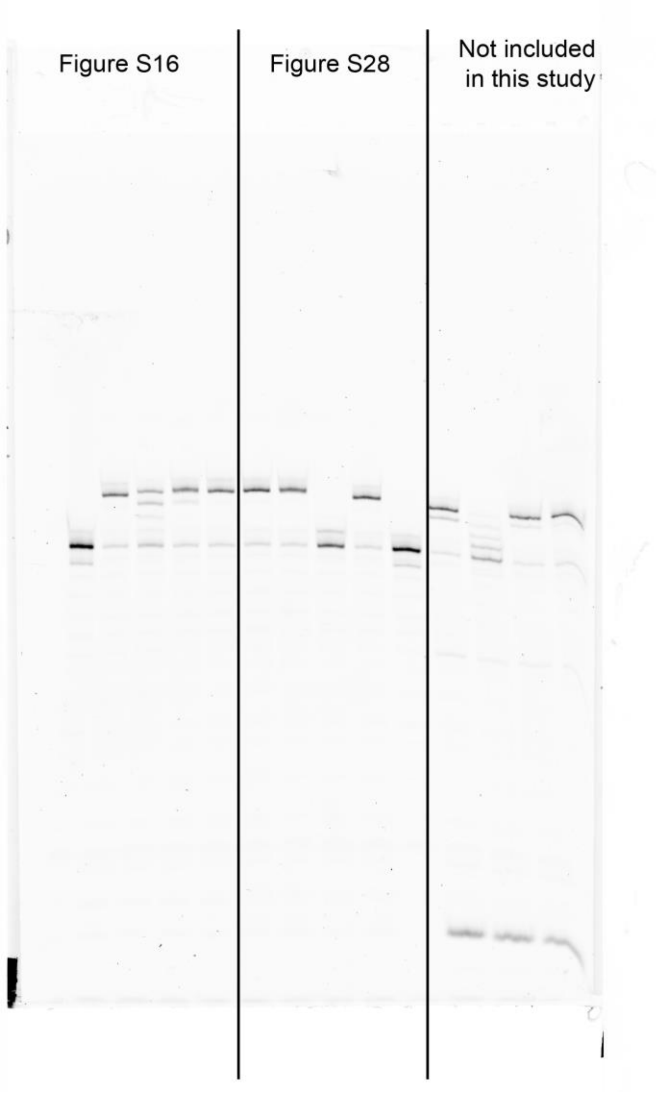
Figure S16
Figure S28
Not included in this study

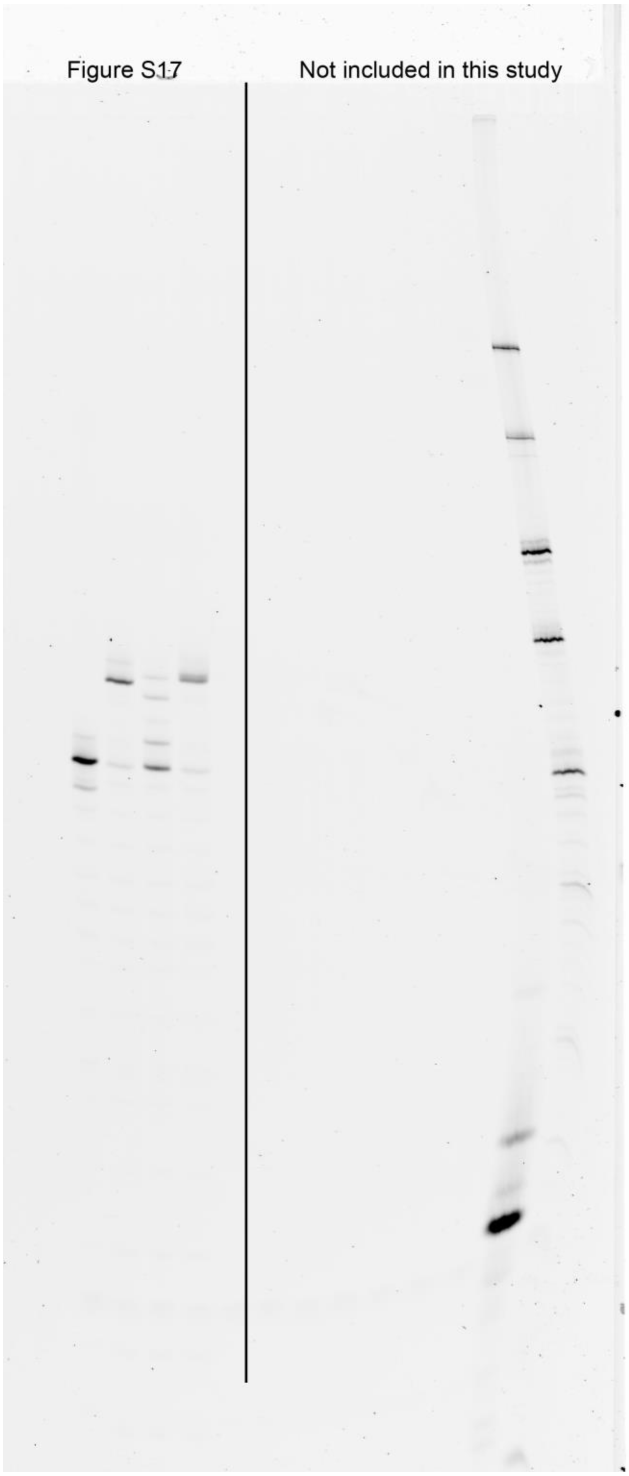
Figure S17
Not included in this study

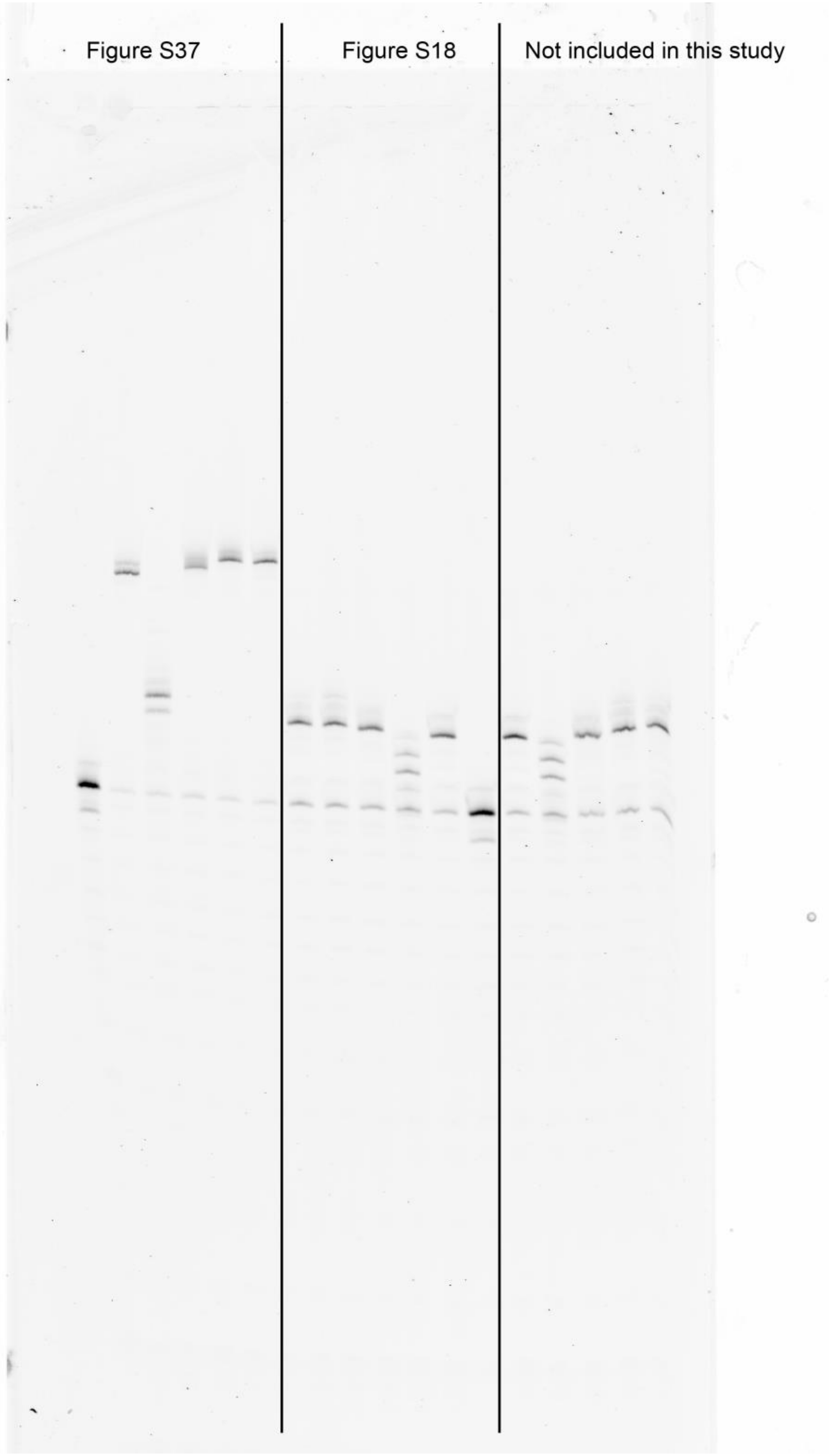
Figure S37
Figure S18
Not included in this study

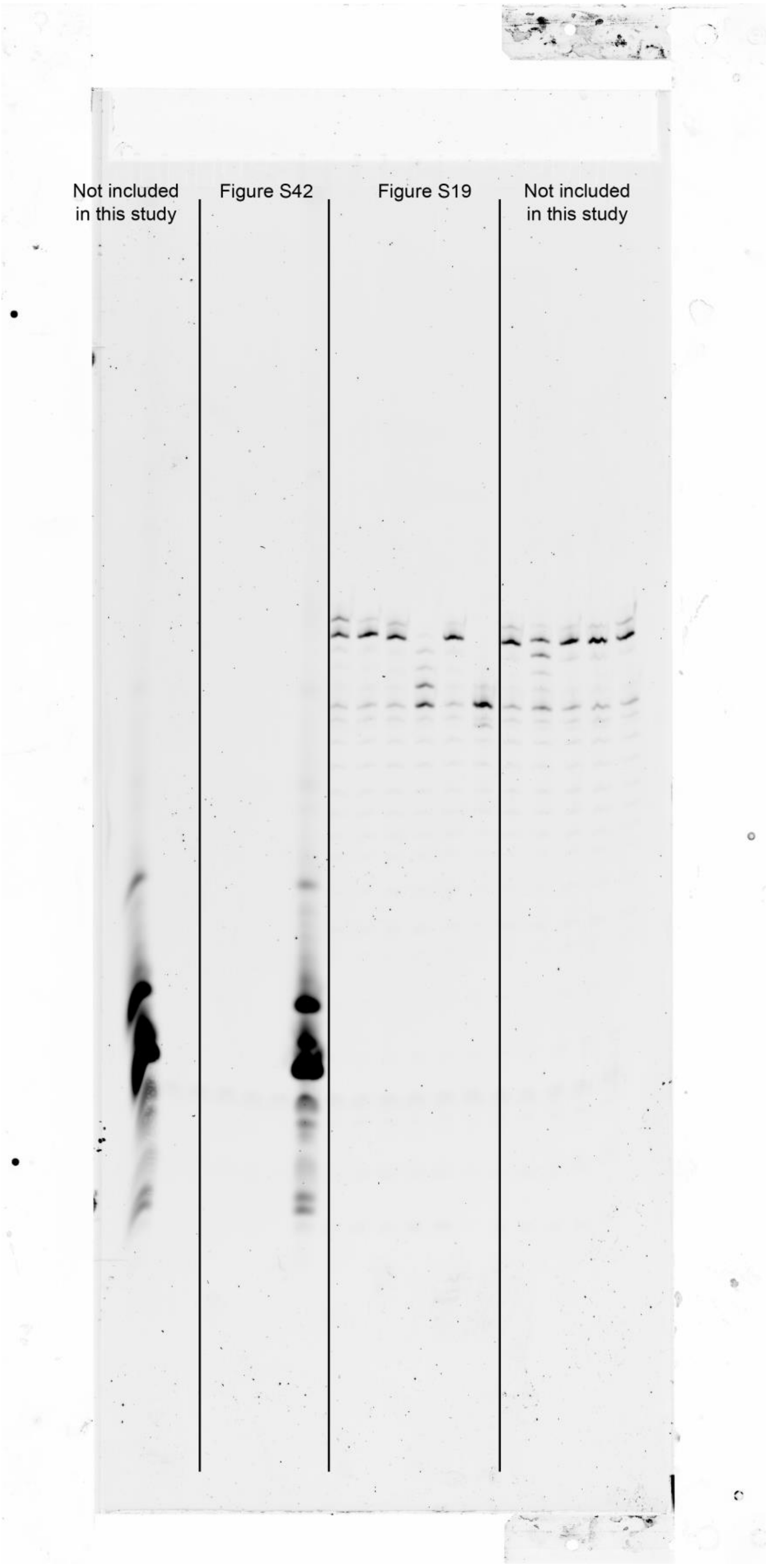

Not included in this study
Figure S42
Figure S19
Not included in this study

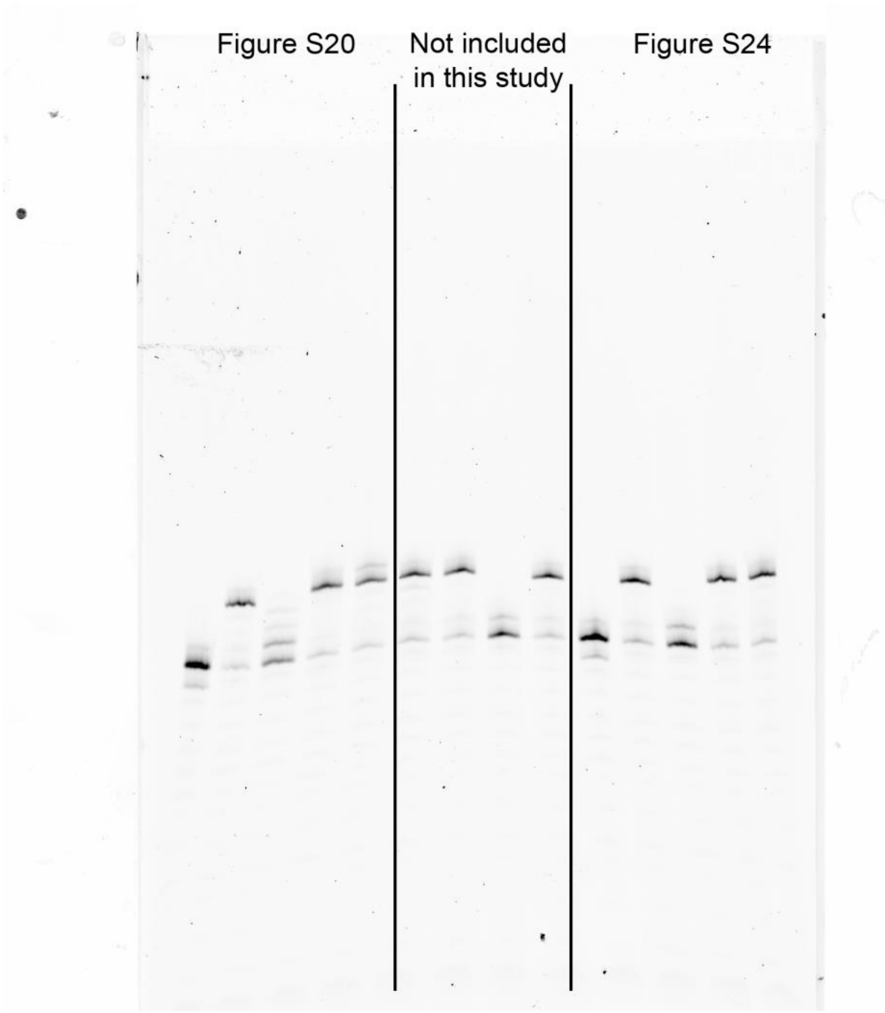
Figure S20
Not included in this study
Figure S24

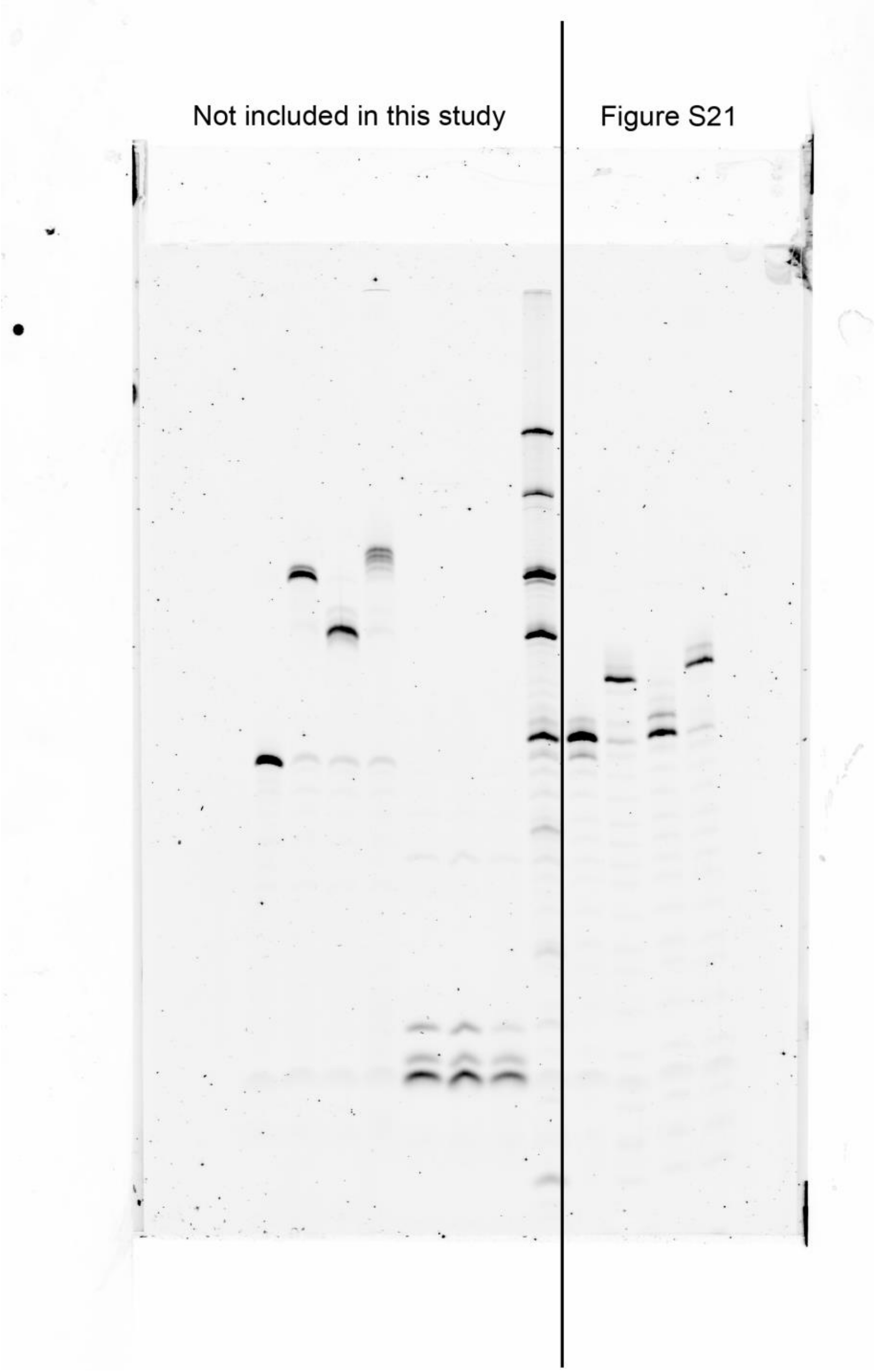
Not included in this study
Figure S21

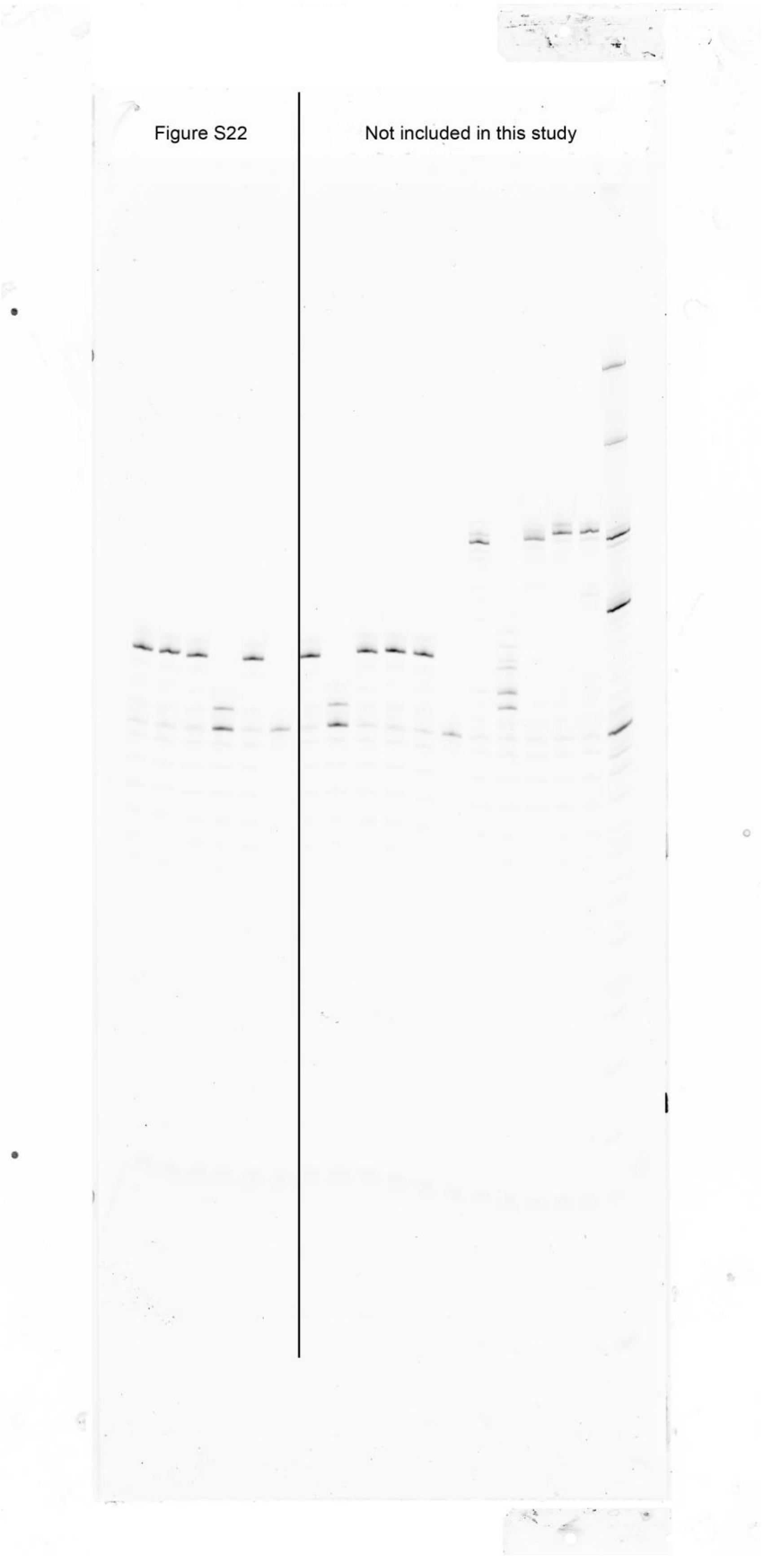
Figure S22
Not included in this study

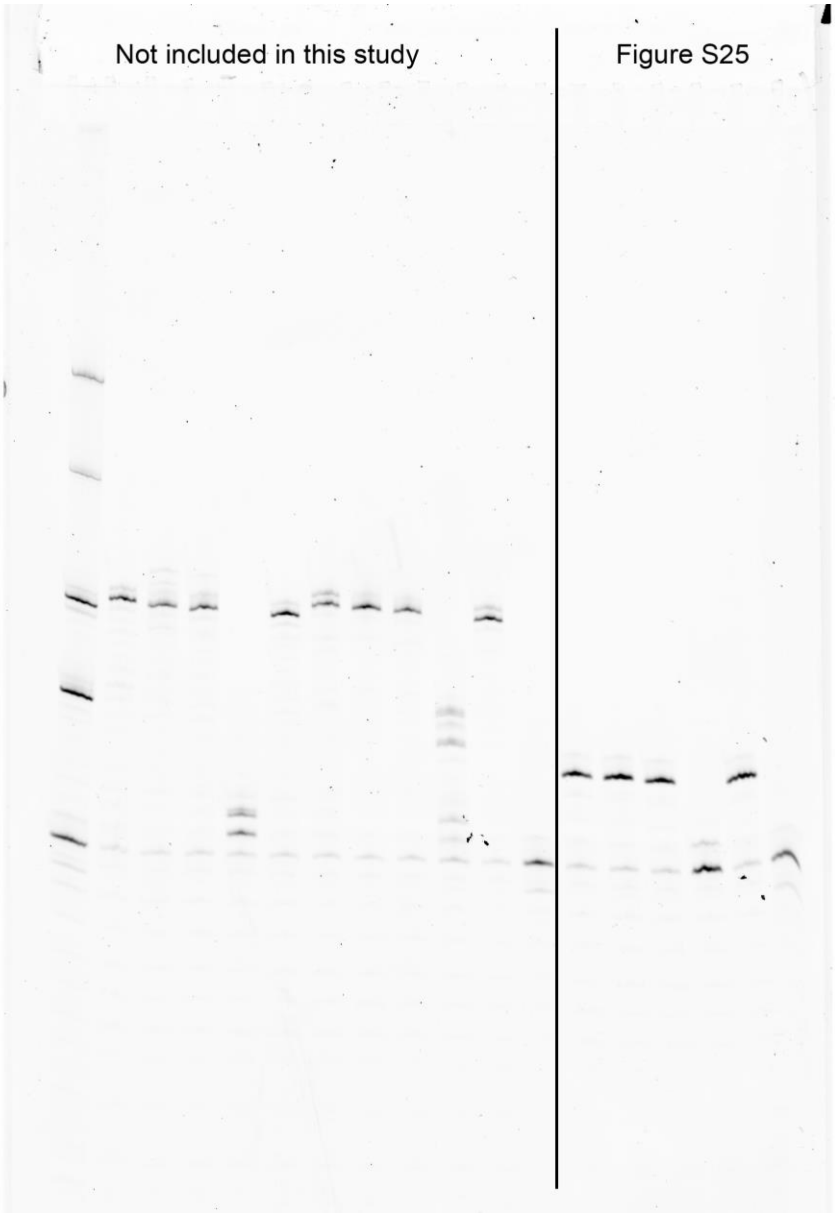
Not included in this study
Figure S25

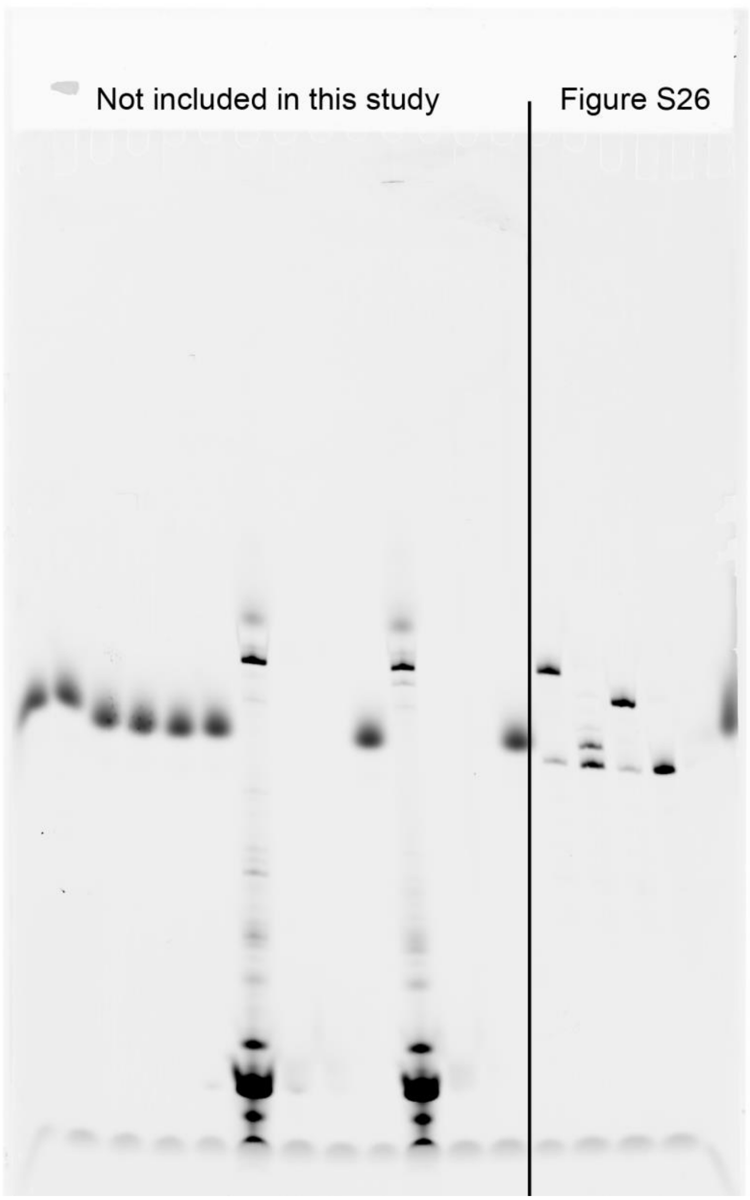
Not included in this study
Figure S26

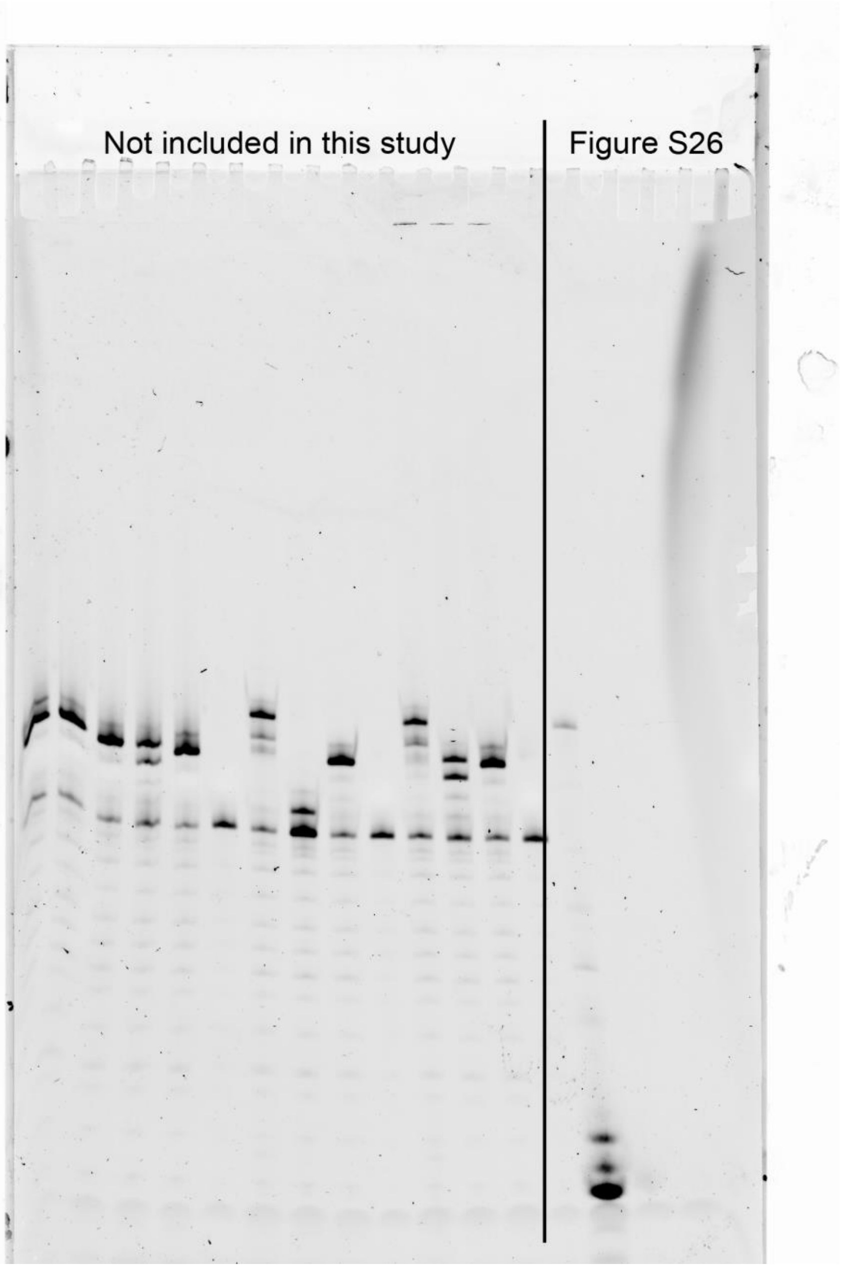
Not included in this study
Figure S26

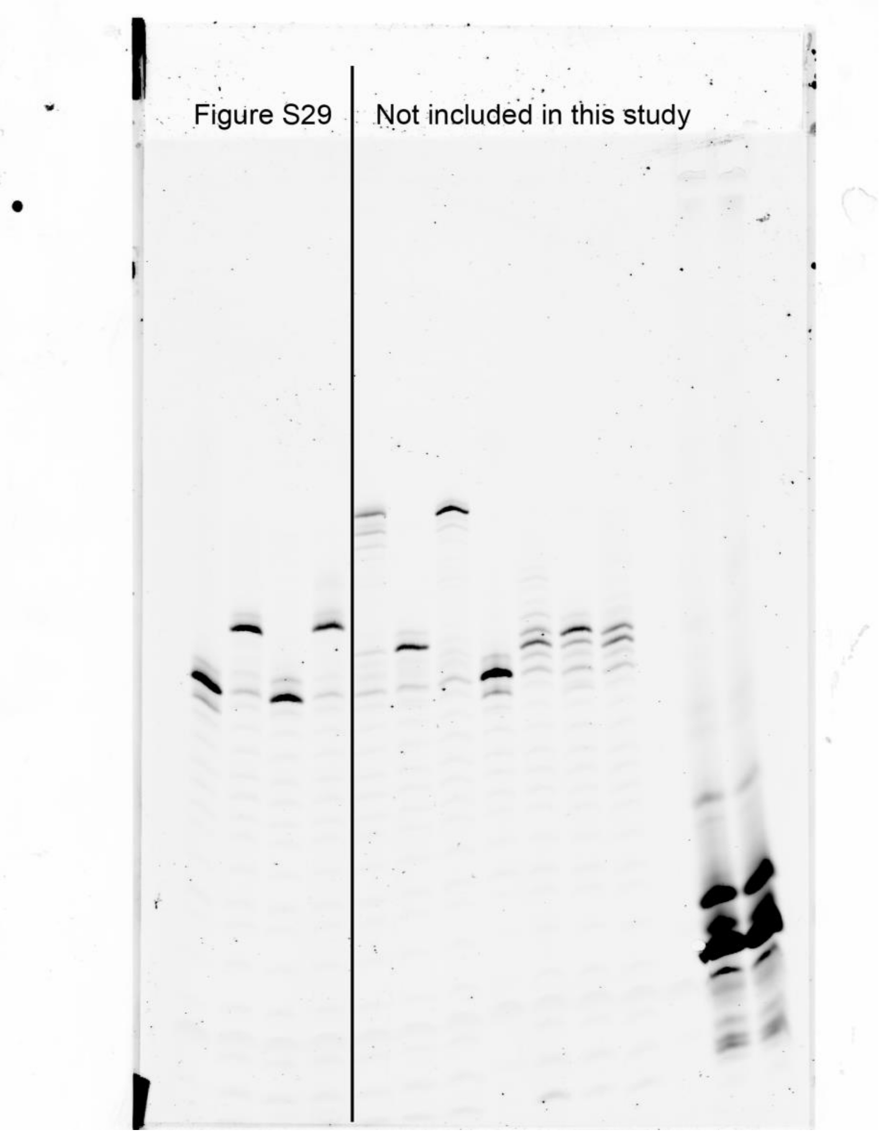
Figure S29
Not included in this study

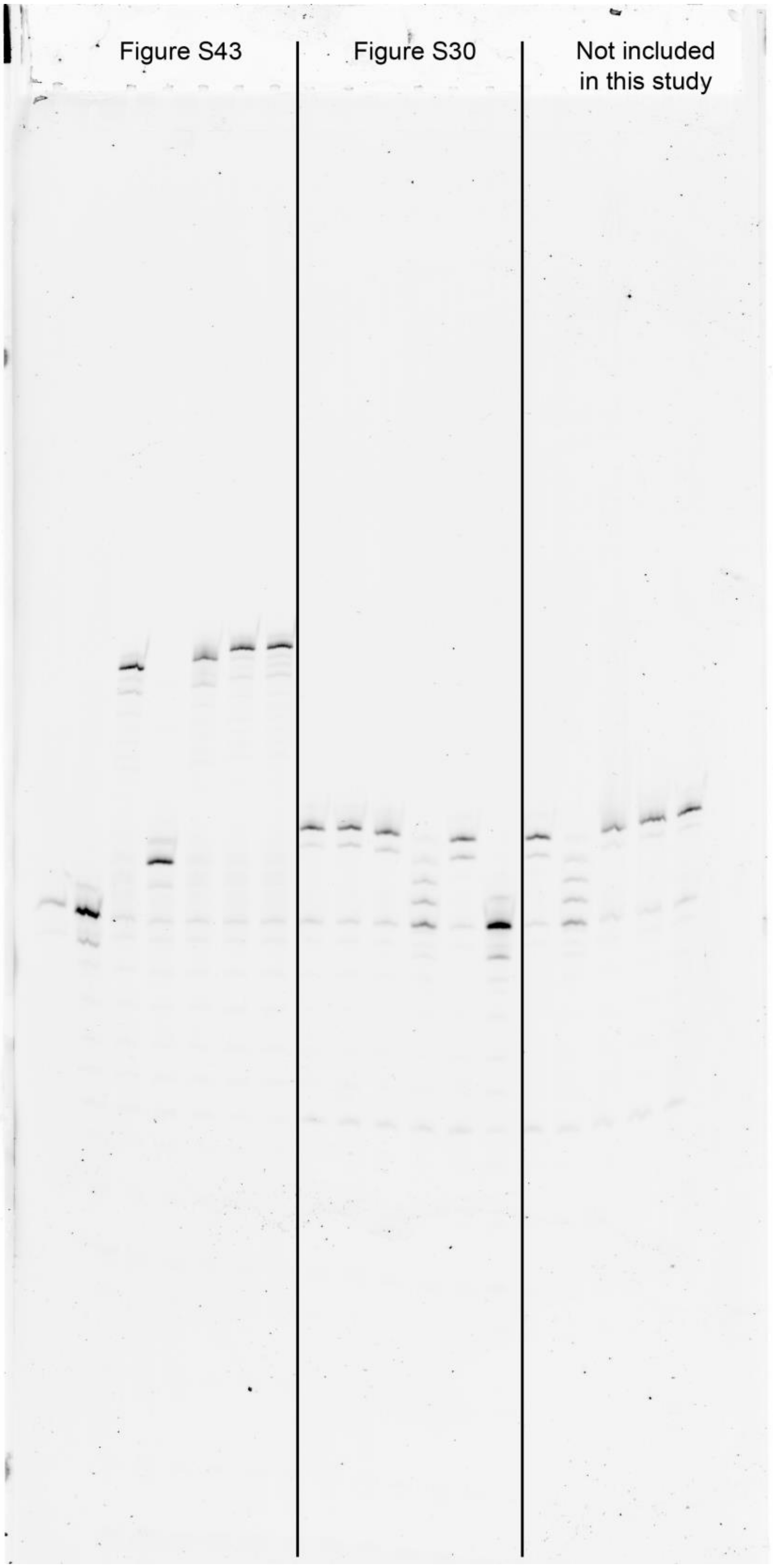
Figure S43
Figure S30
Not included in this study

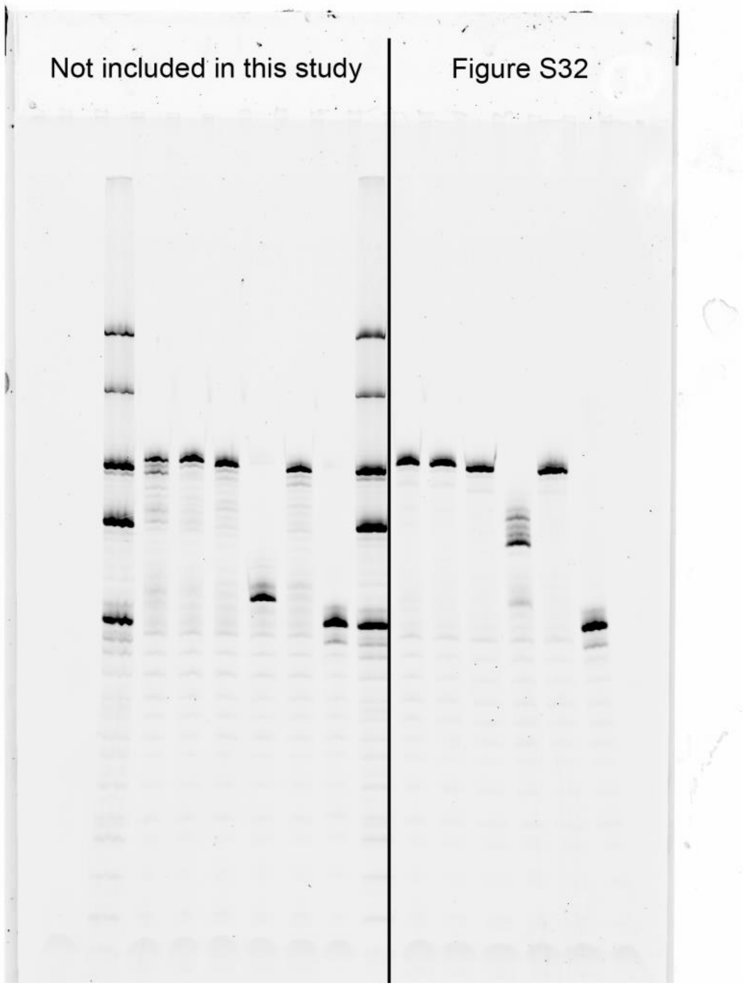
Not included in this study
Figure S32

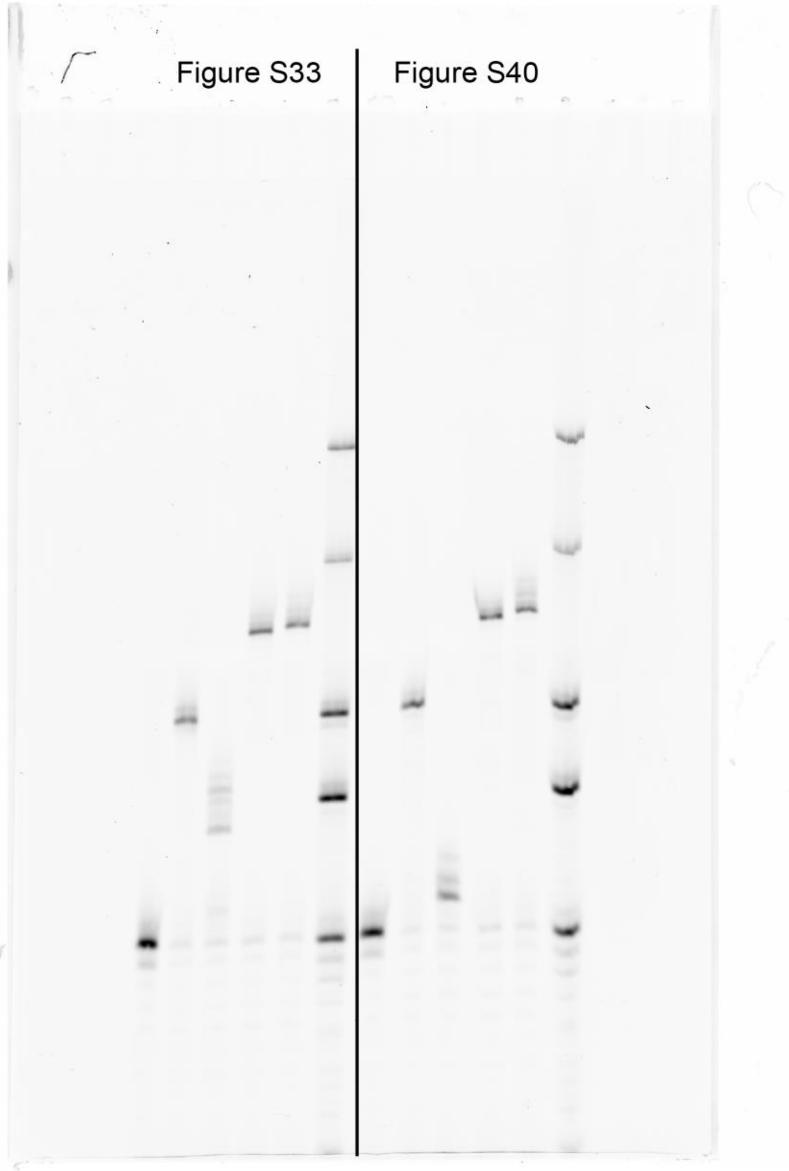
Figure S33
Figure S40

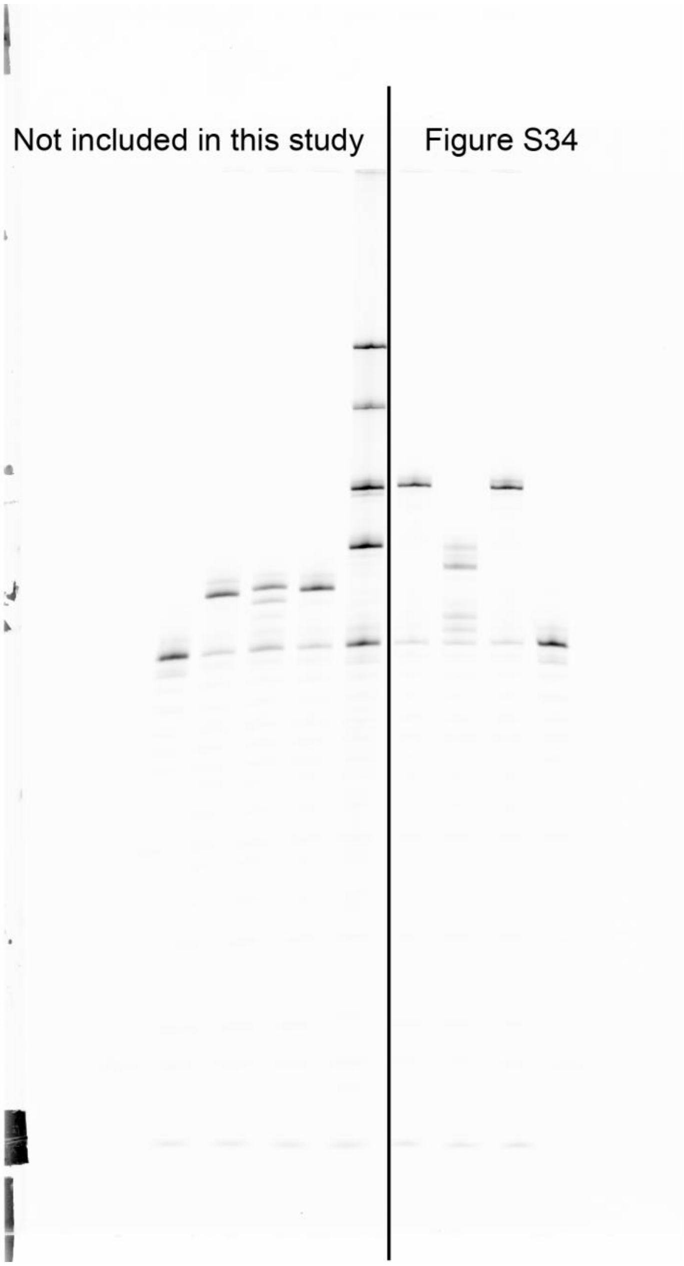
Not included in this study
Figure S34

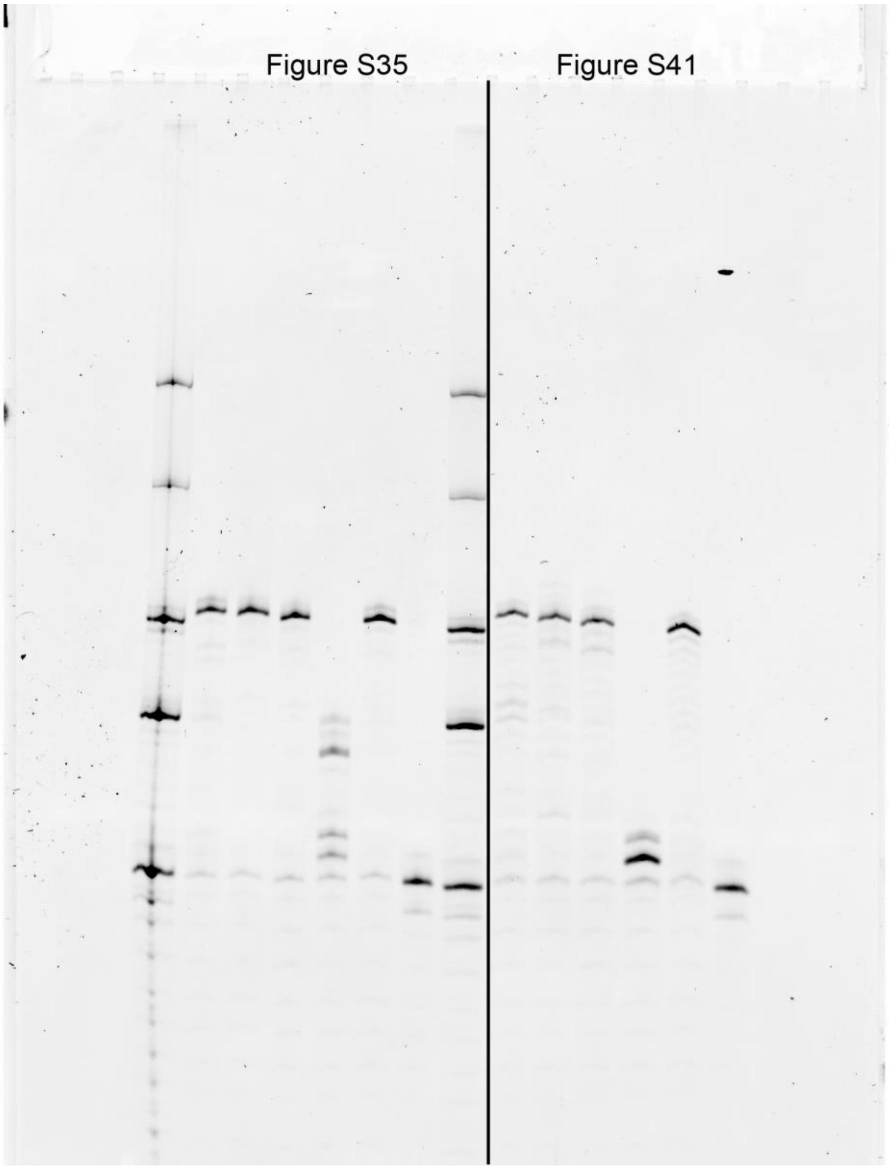
Figure S35
Figure S41

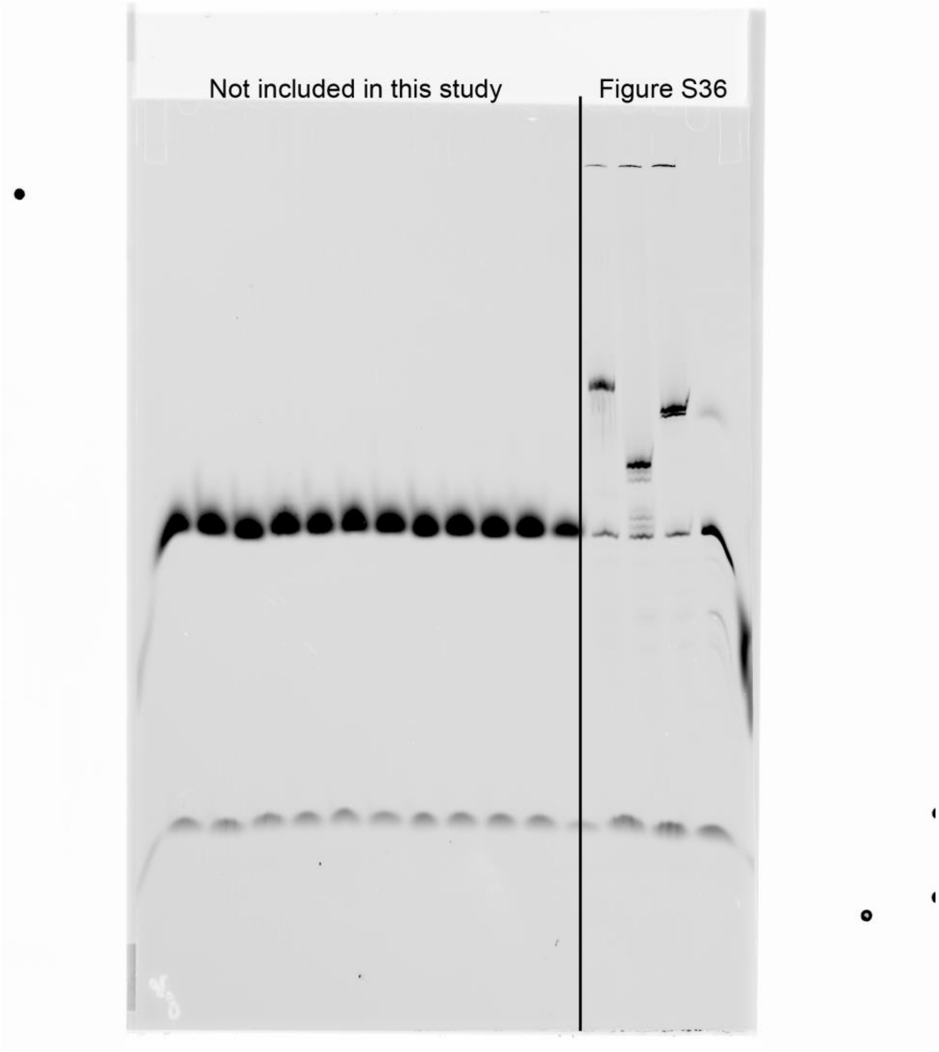
Not included in this study
Figure S36

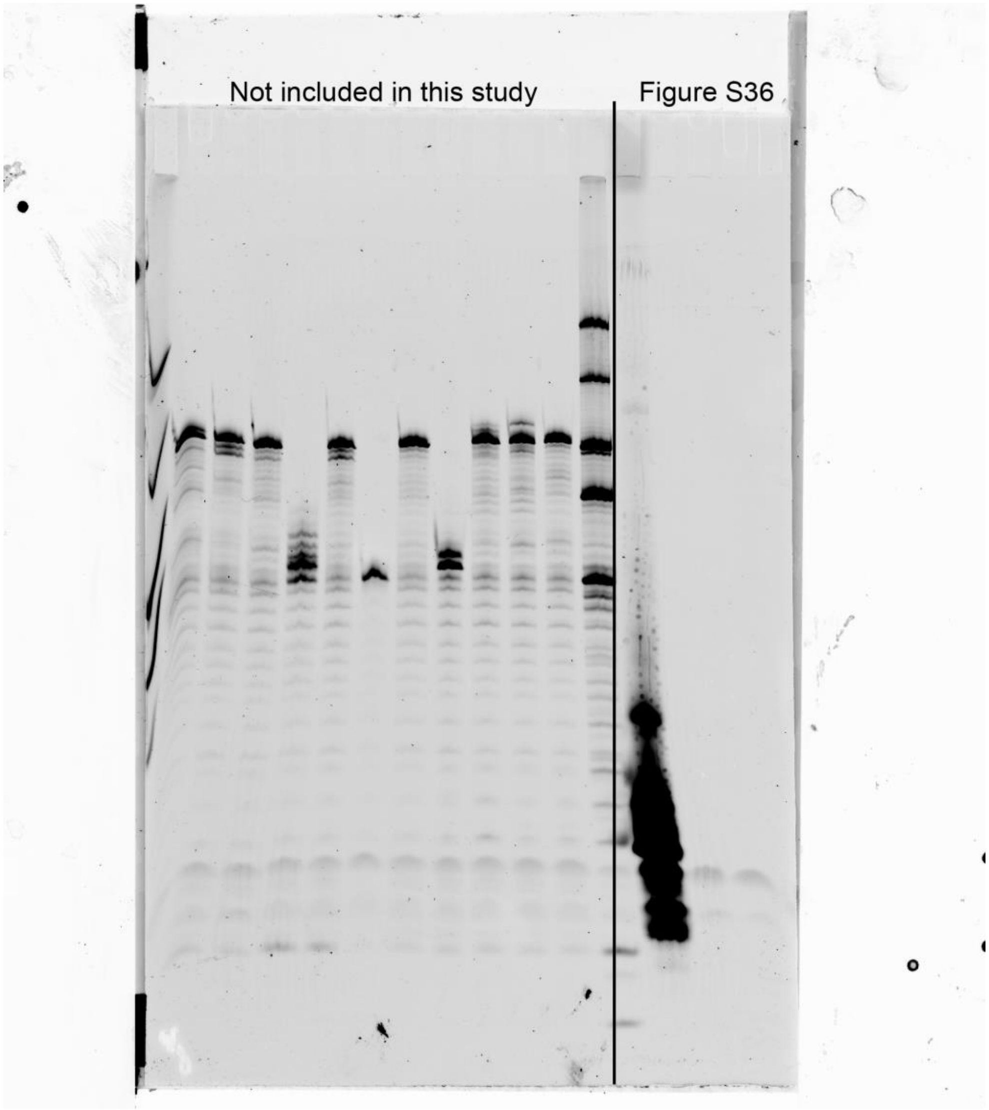
Not included in this study
Figure S36

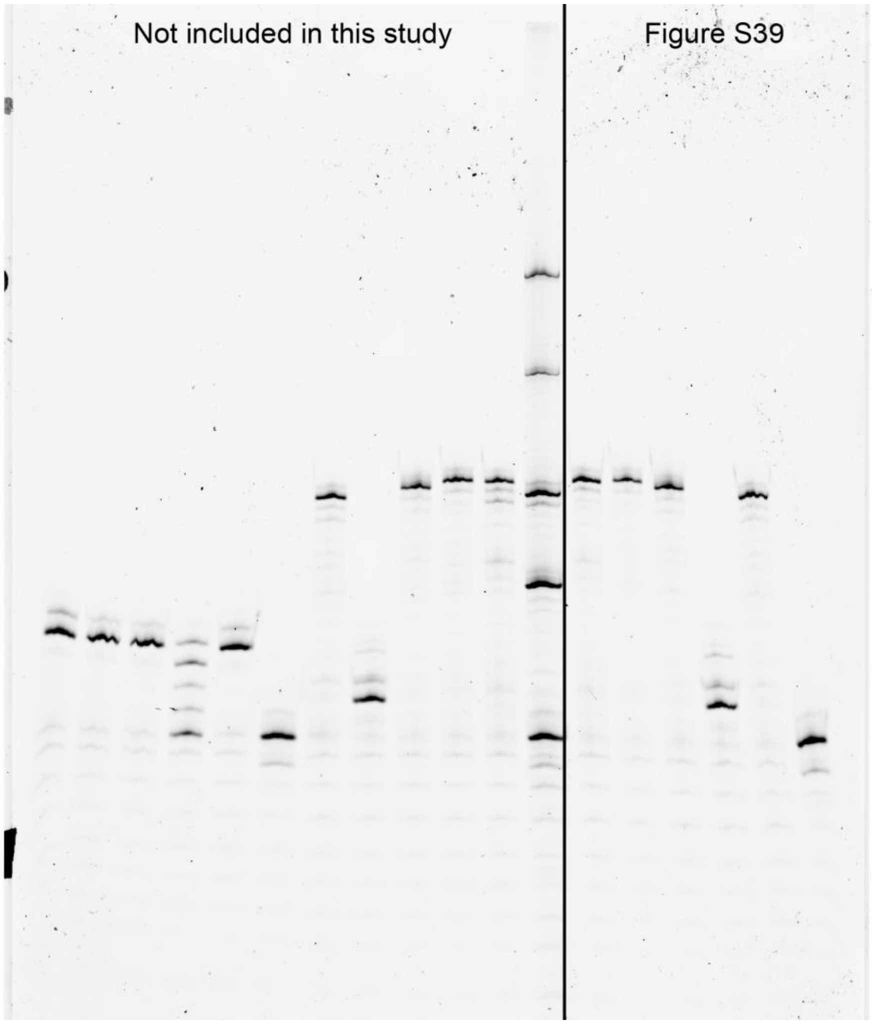
Not included in this study
Figure S39

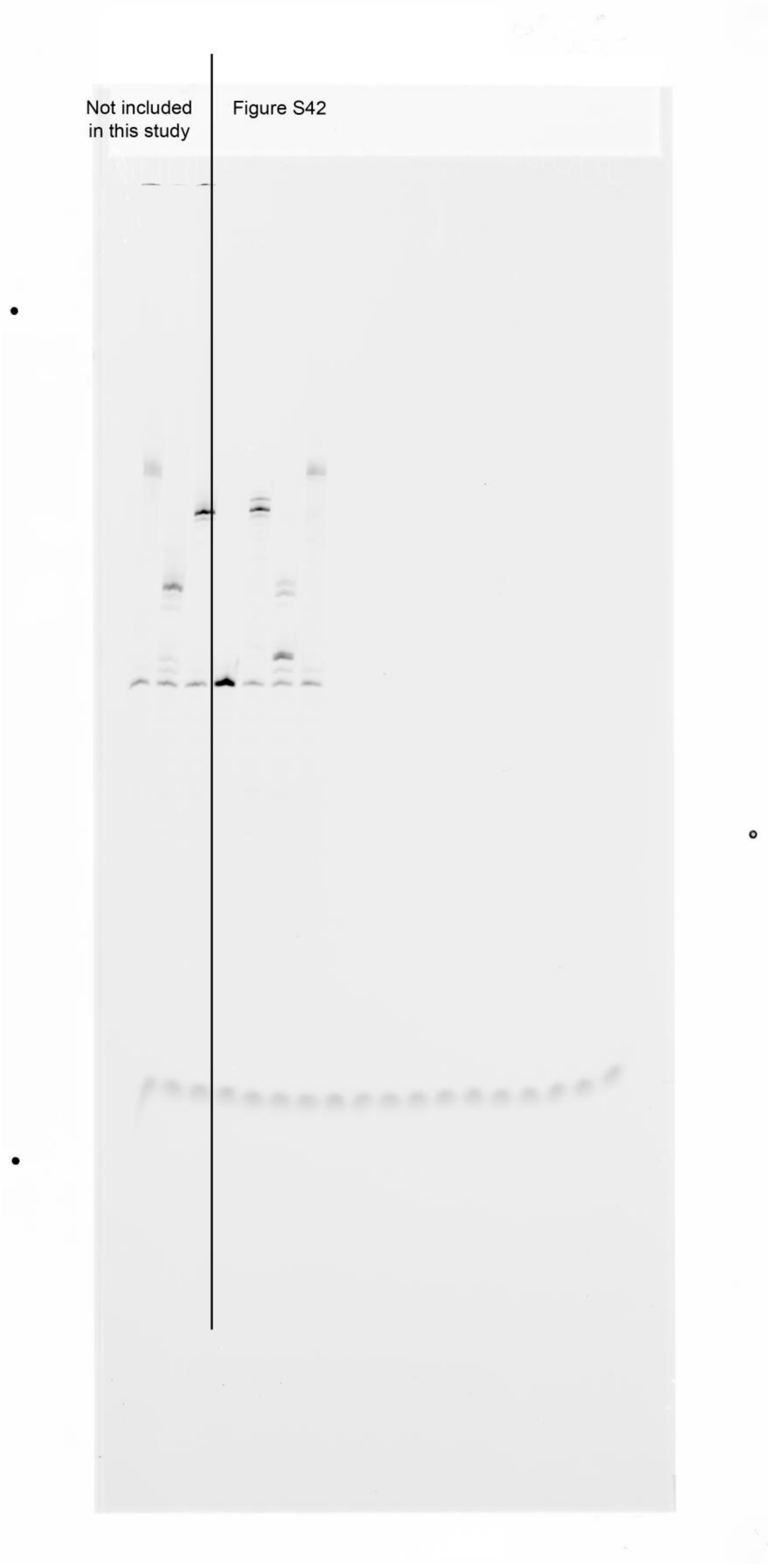
Not included in this study
Figure S42

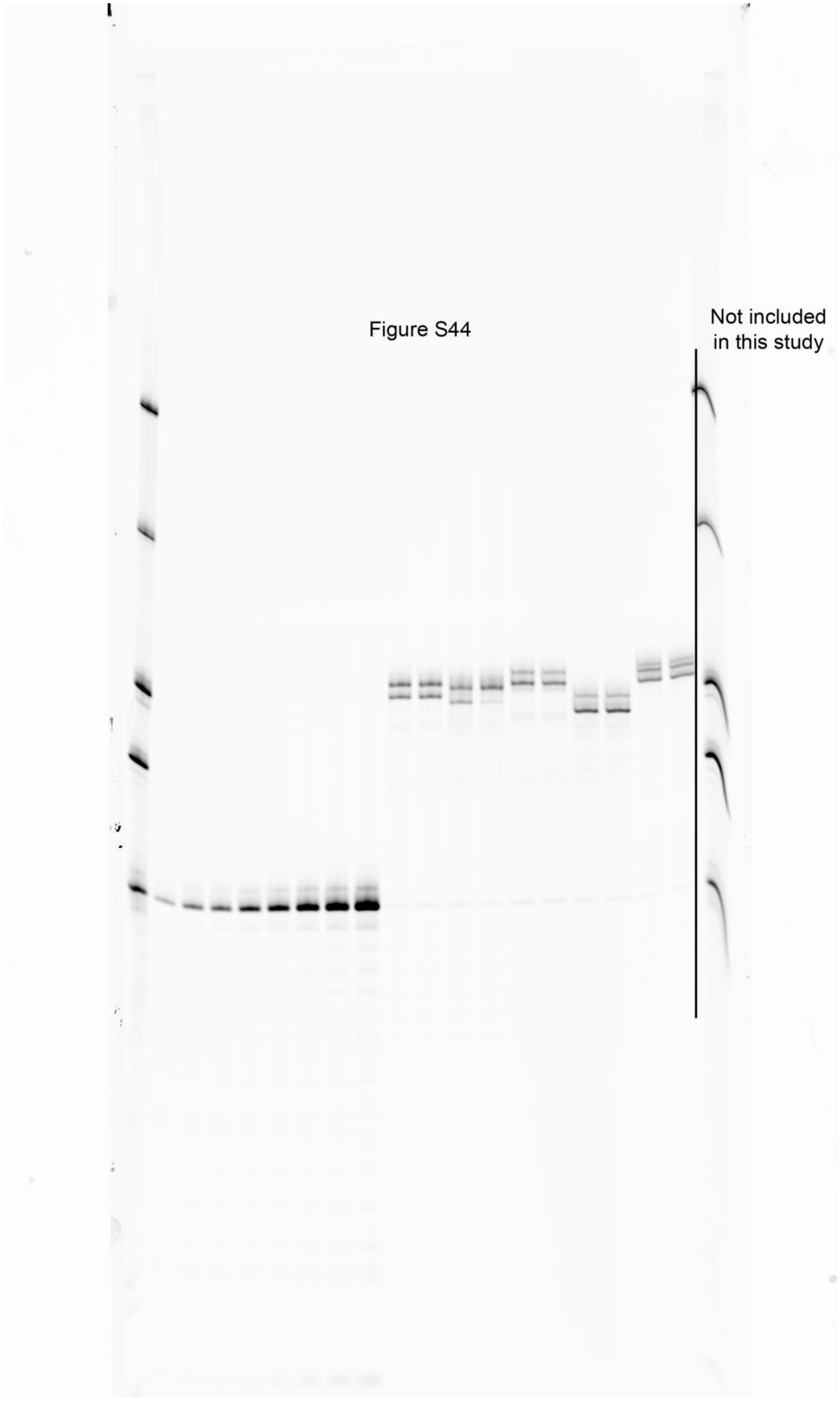

Figure S44
Not included in this study

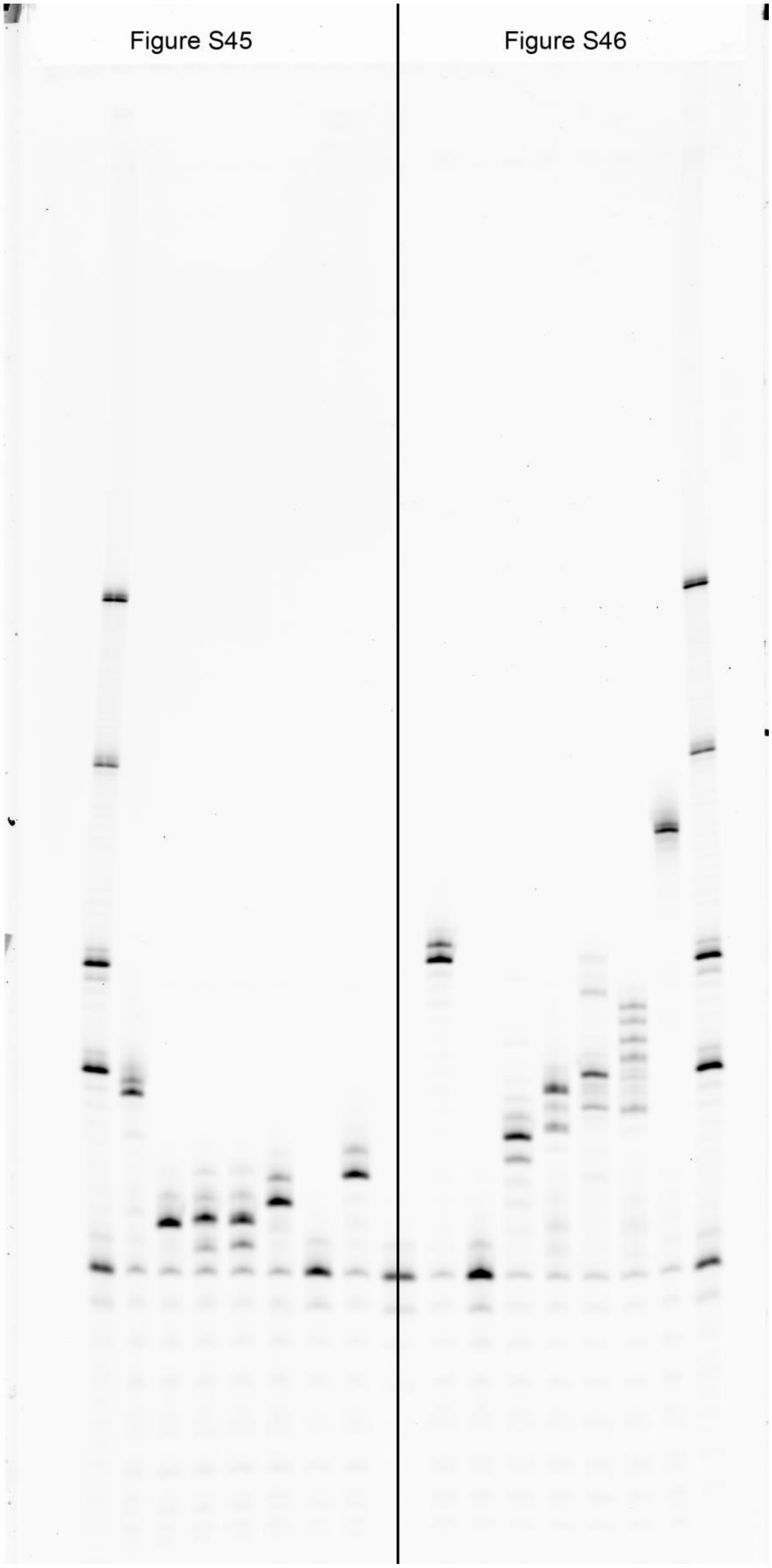

Figure S45
Figure S46

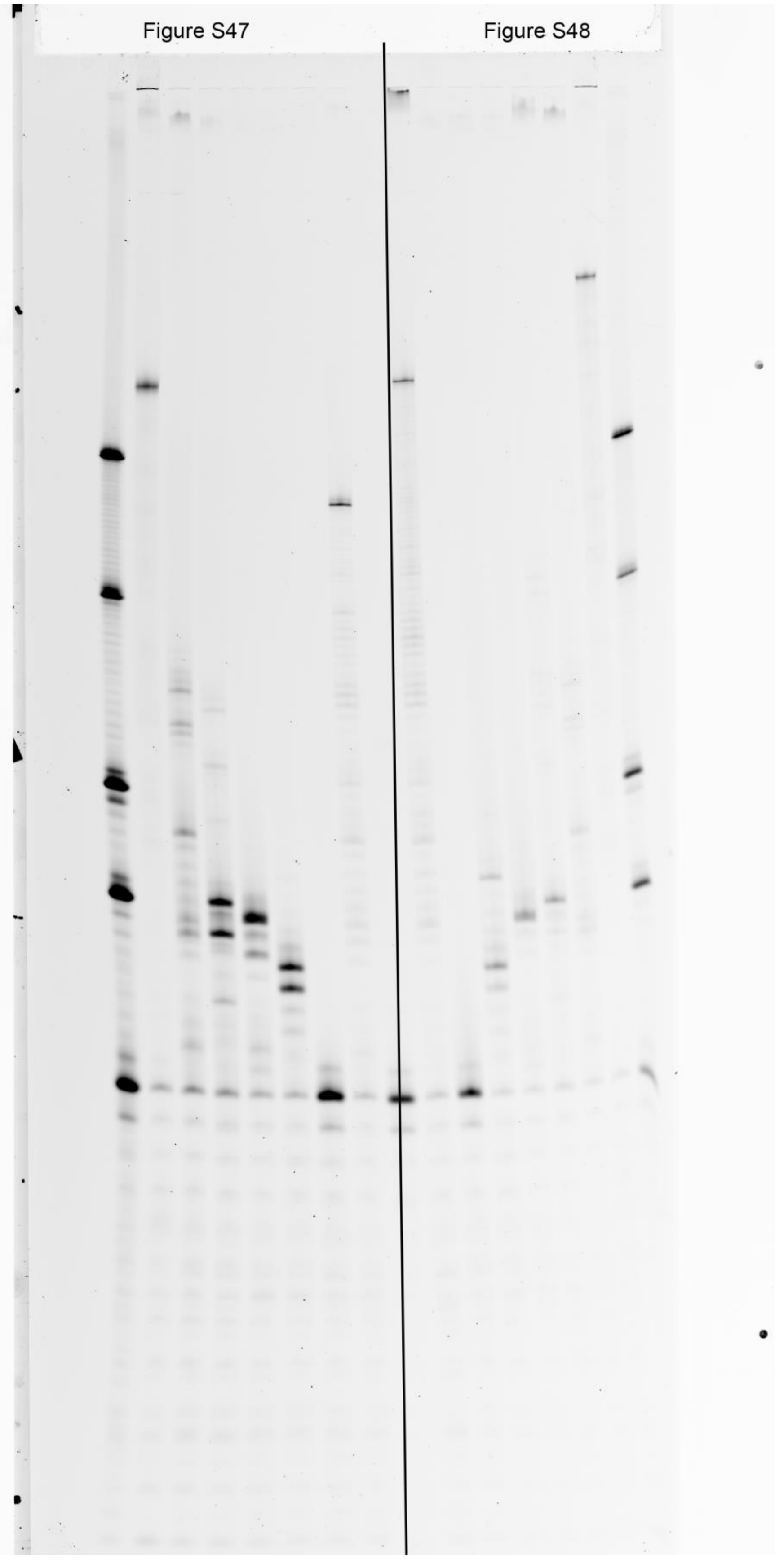
Figure S47
Figure S48

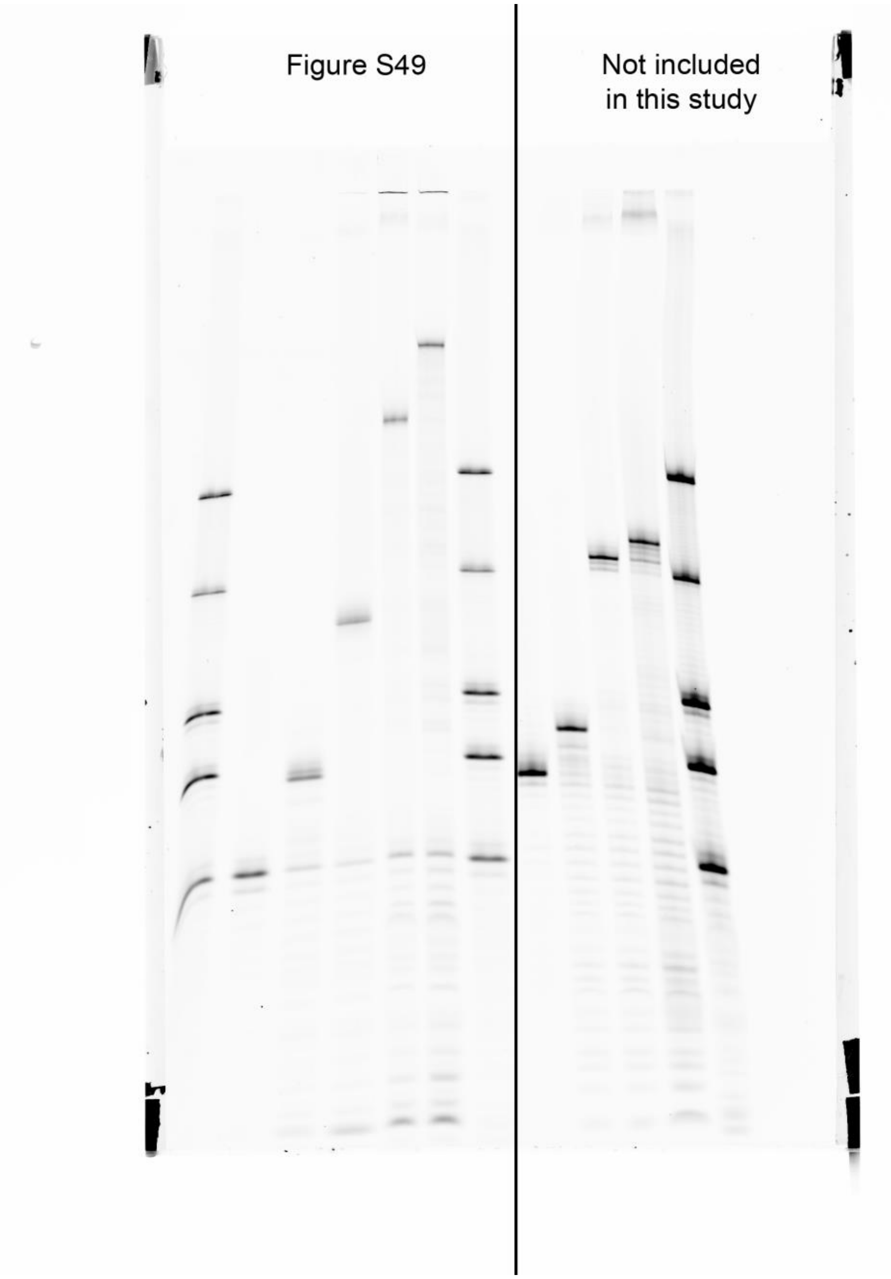
Figure S49
Not included
in this study

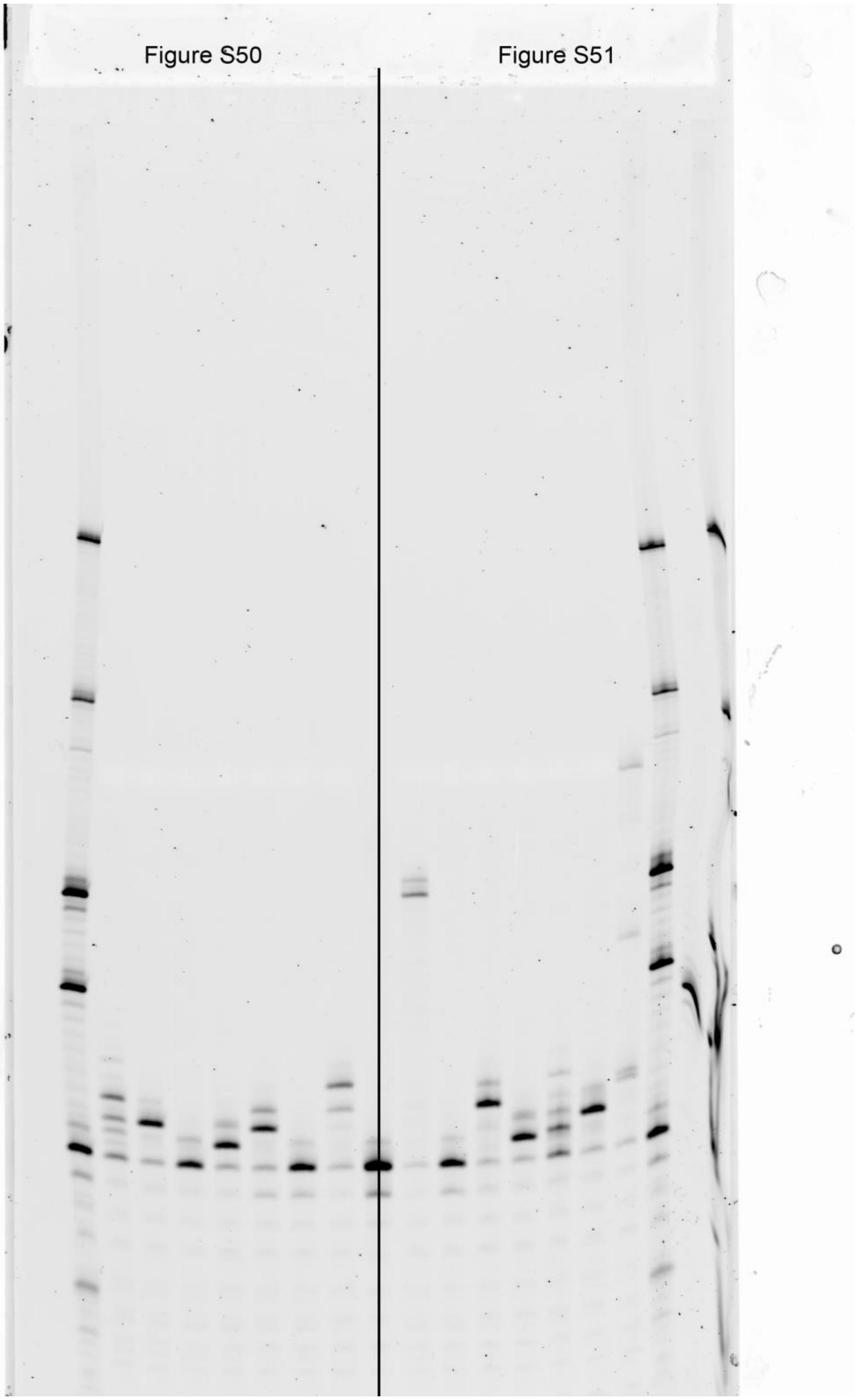

Figure S50
Figure S51

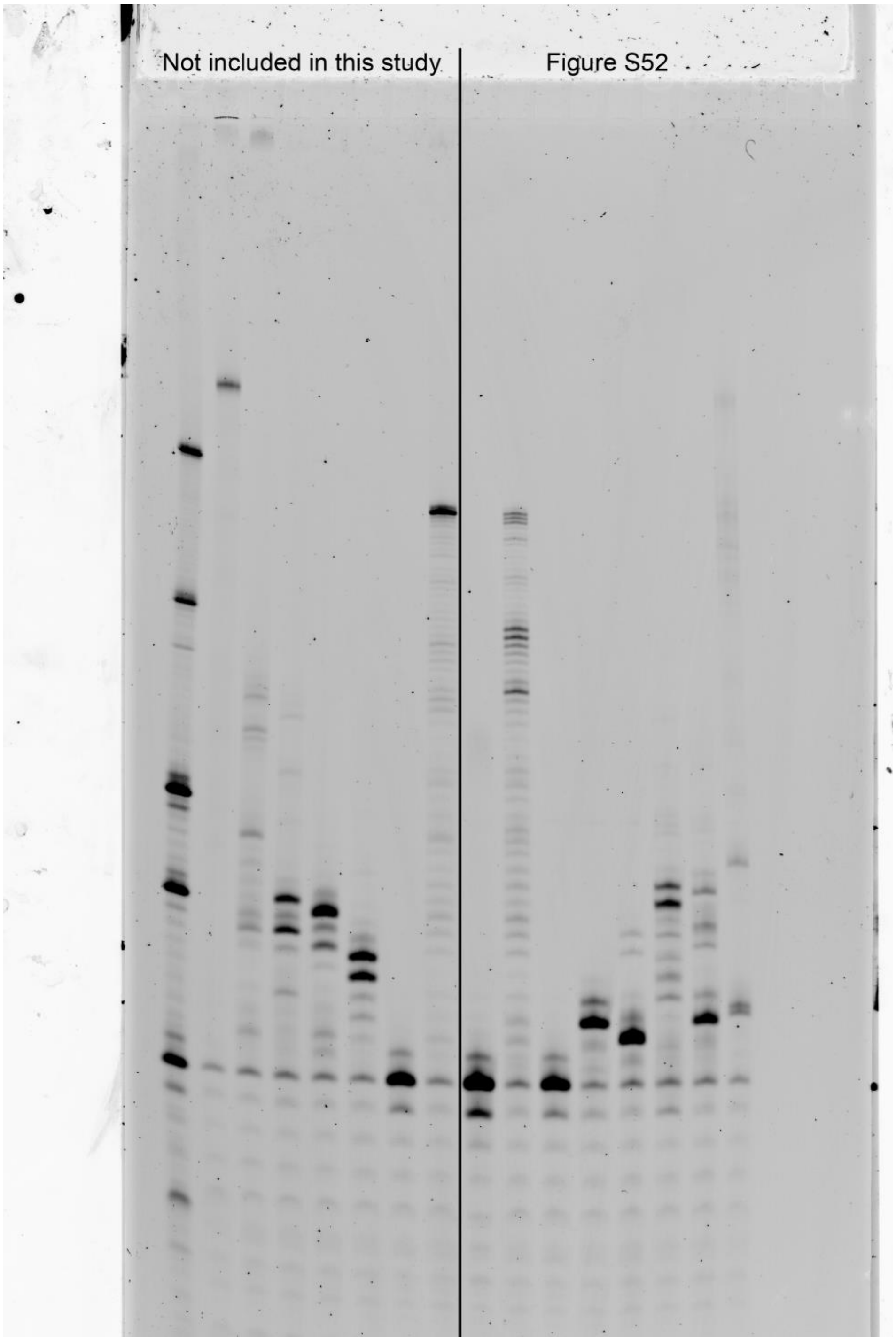
Not included in this study
Figure S52

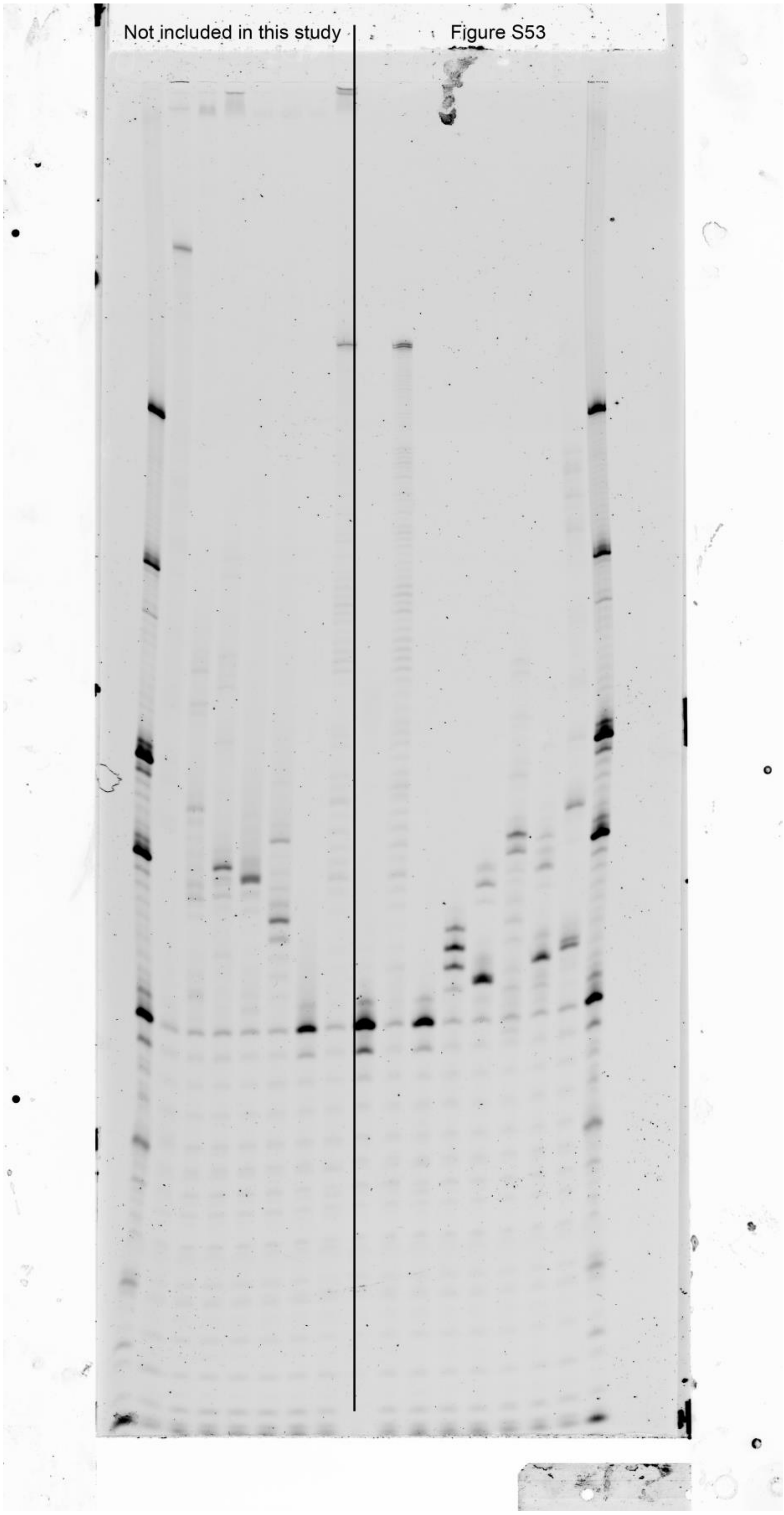
Not included in this study
Figure S53

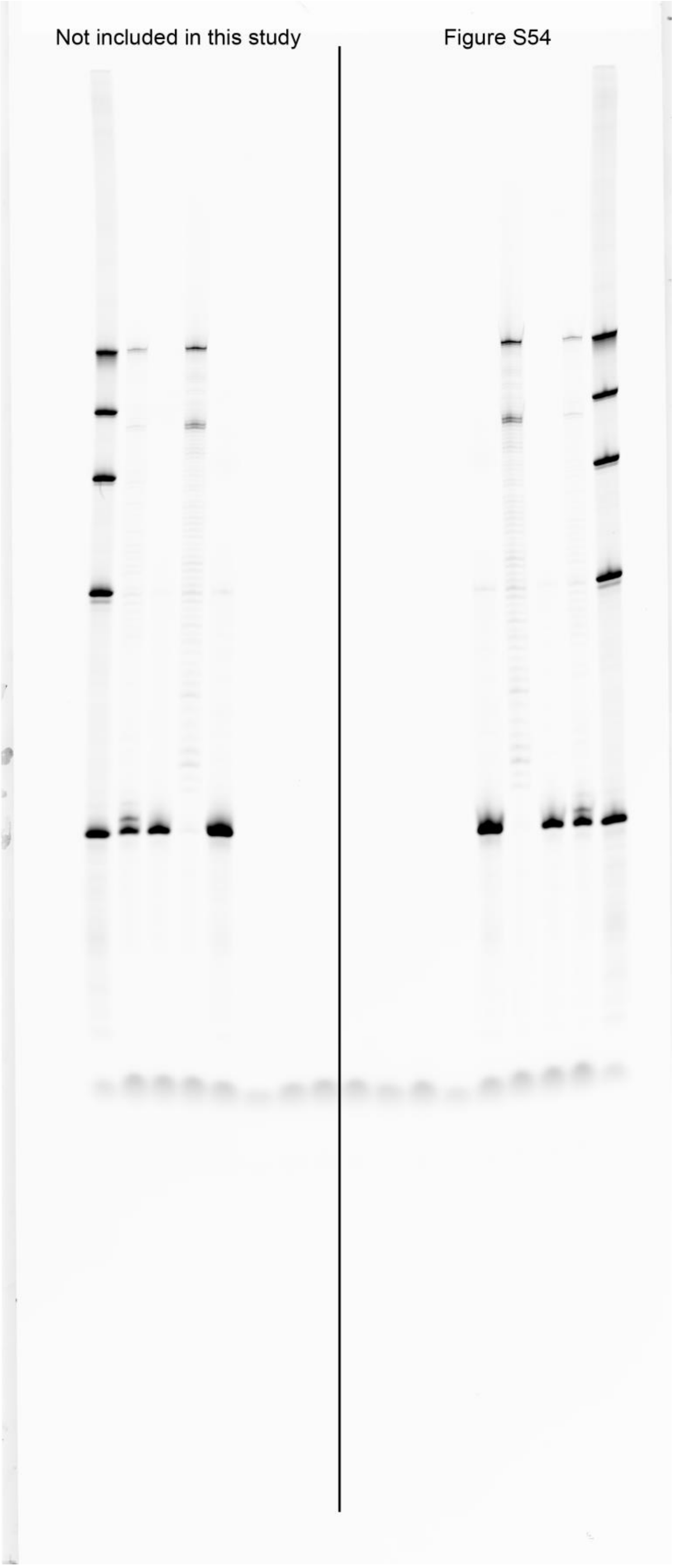
Not included in this study
Figure S54

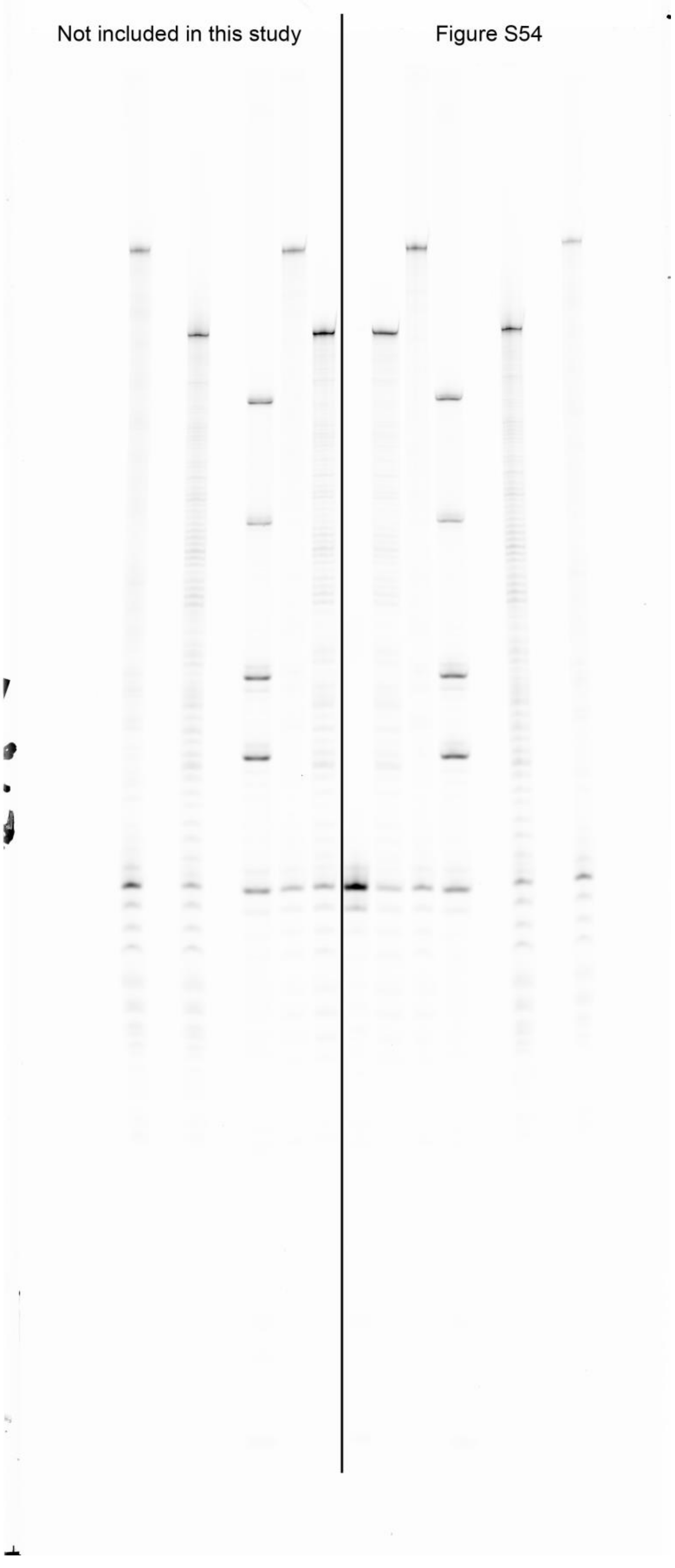
Not included in this study
Figure S54

Figure S55

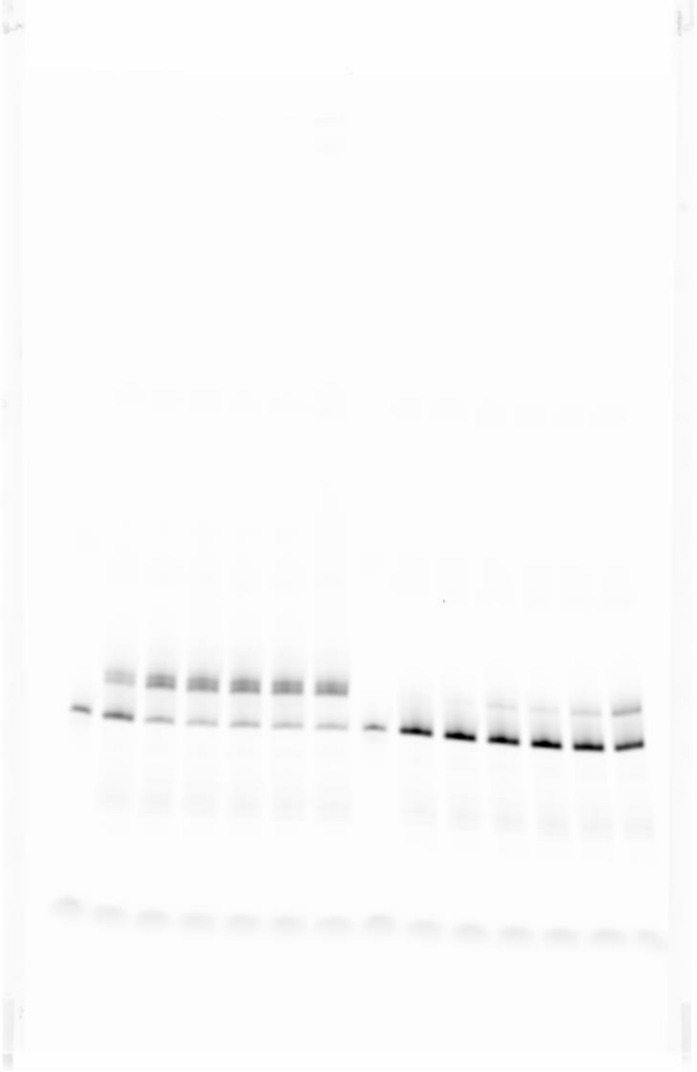

Figure S57

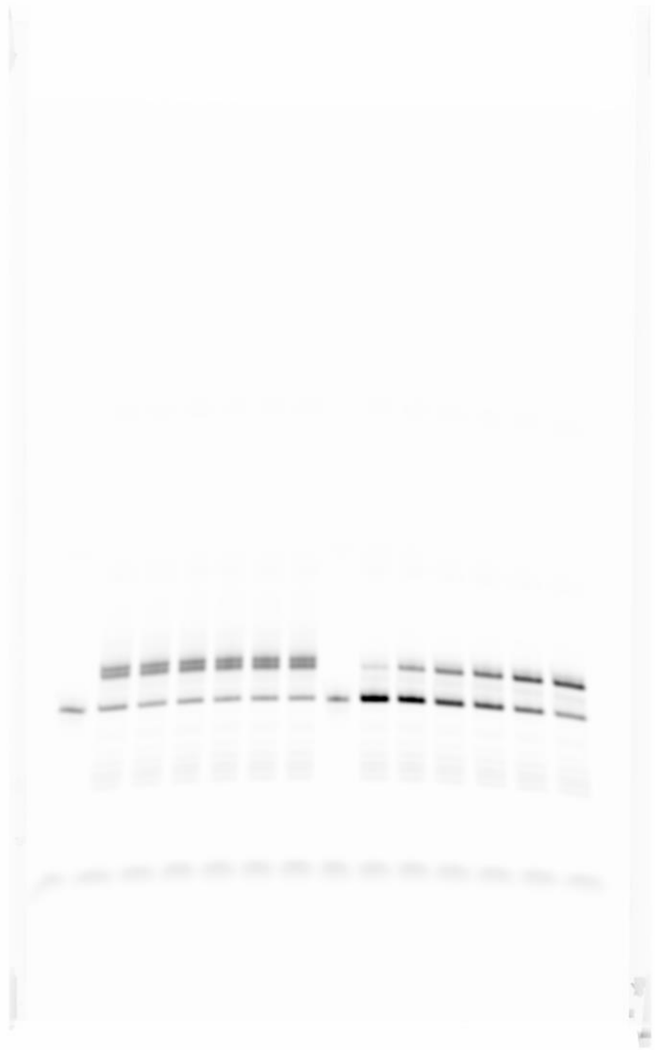

Figure S58

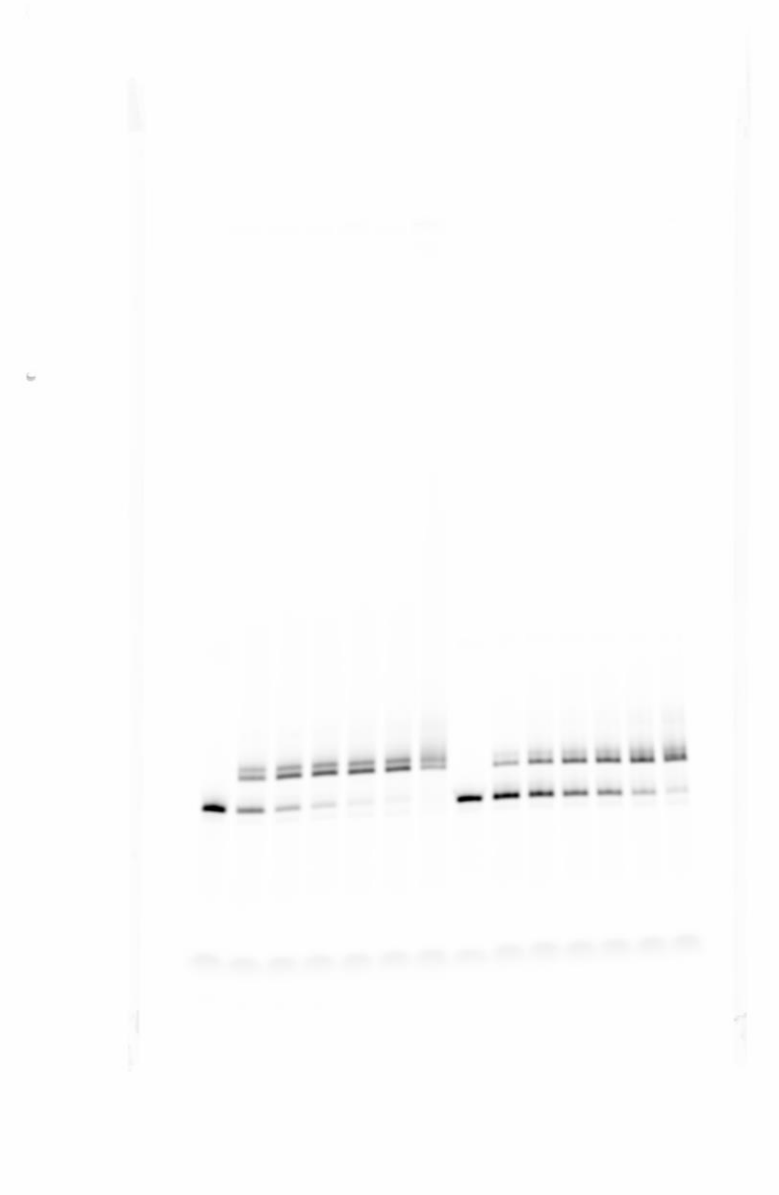

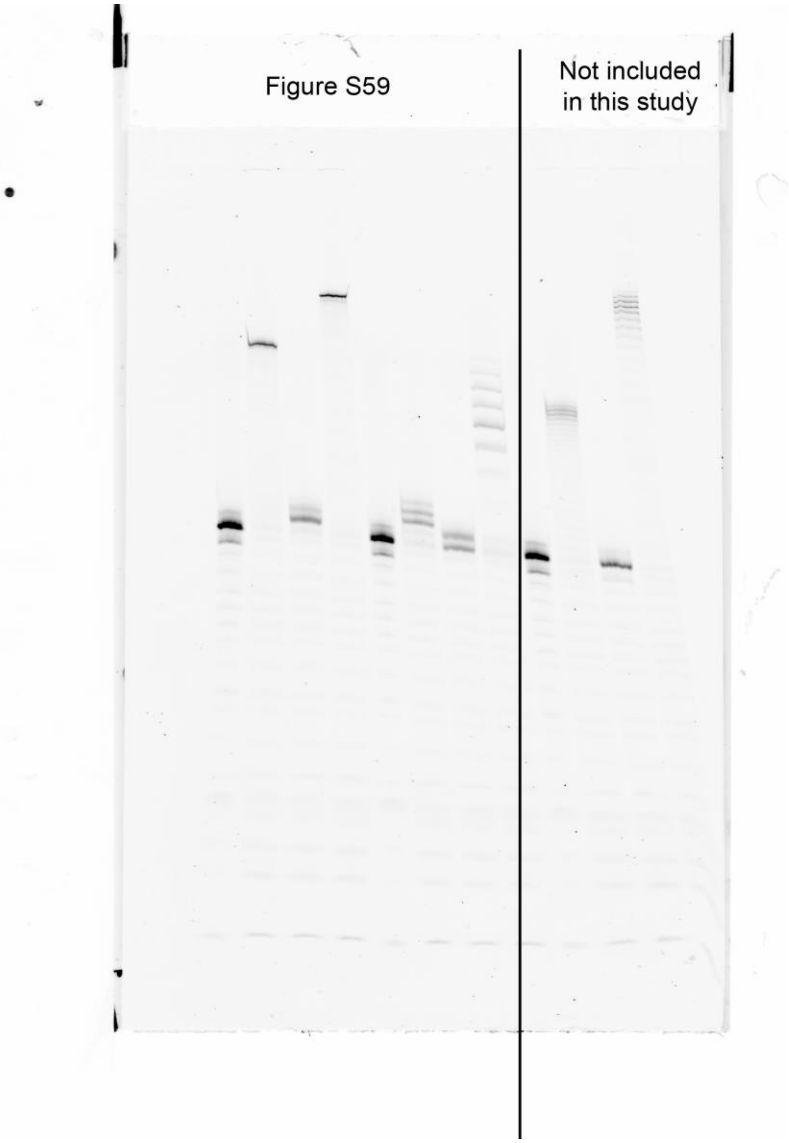
Figure S59
Not included
in this study

Figure S60

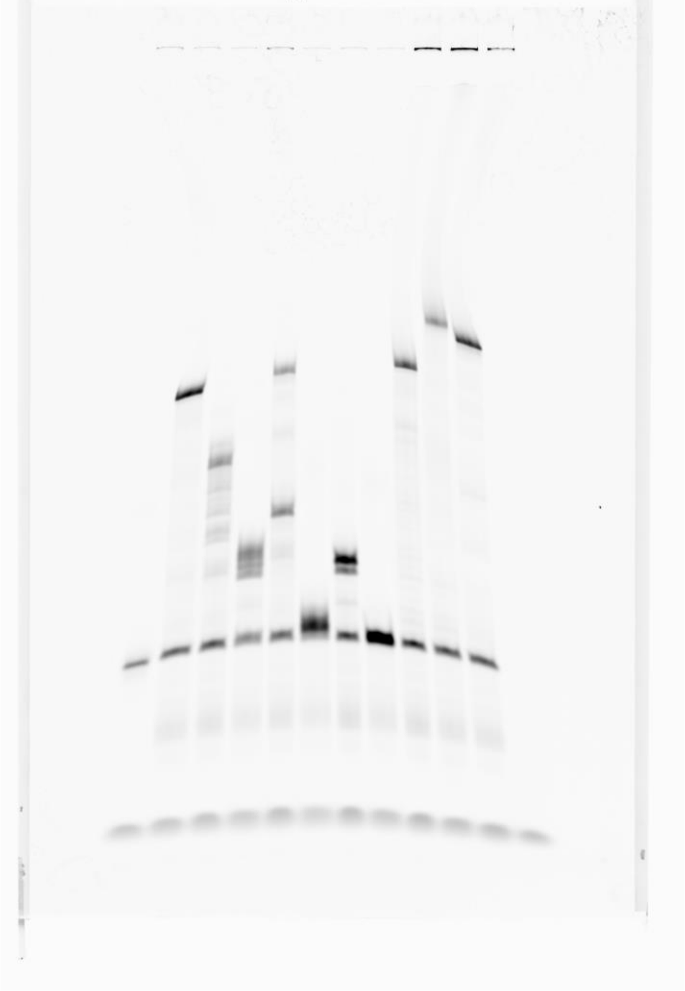

Figure S61

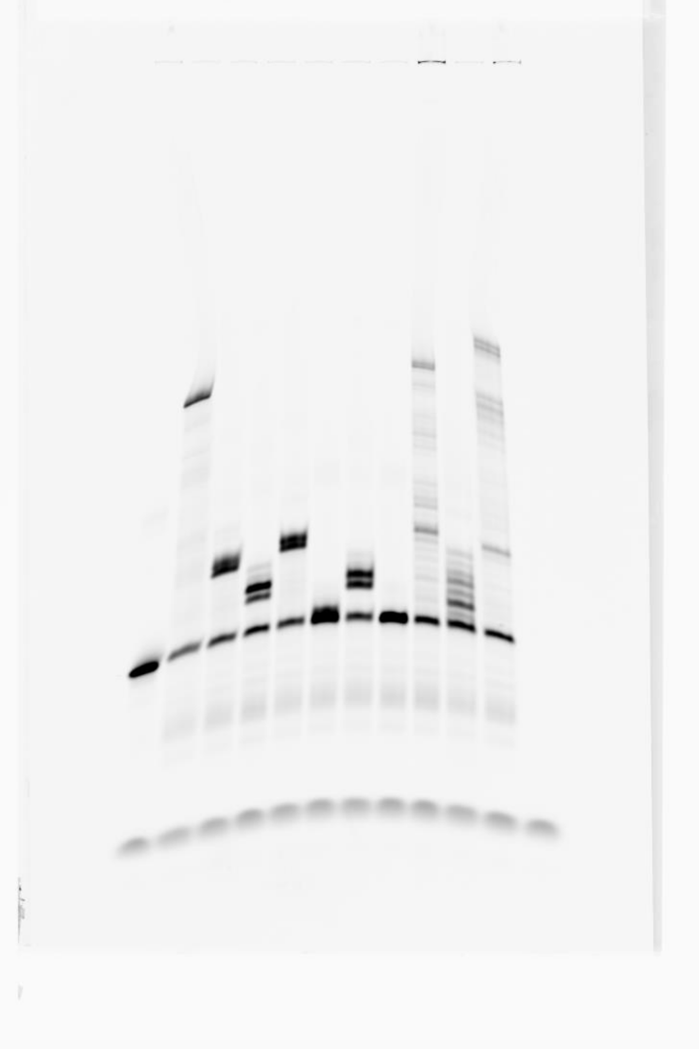

Figure S62

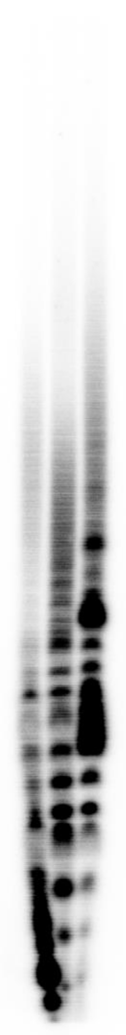

Figure S63

Not included in this study

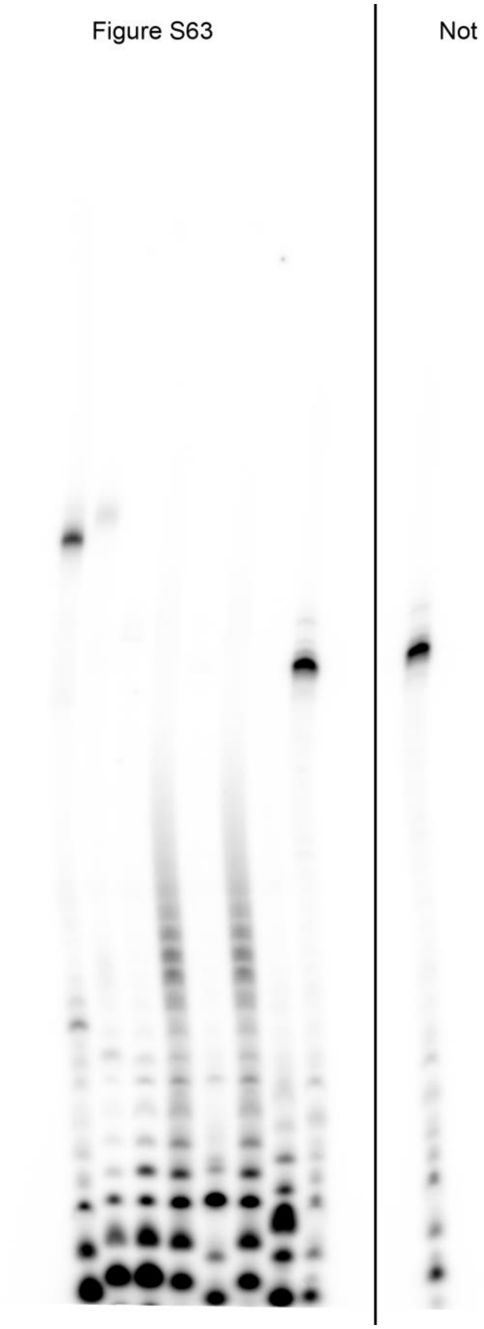

Figure S64

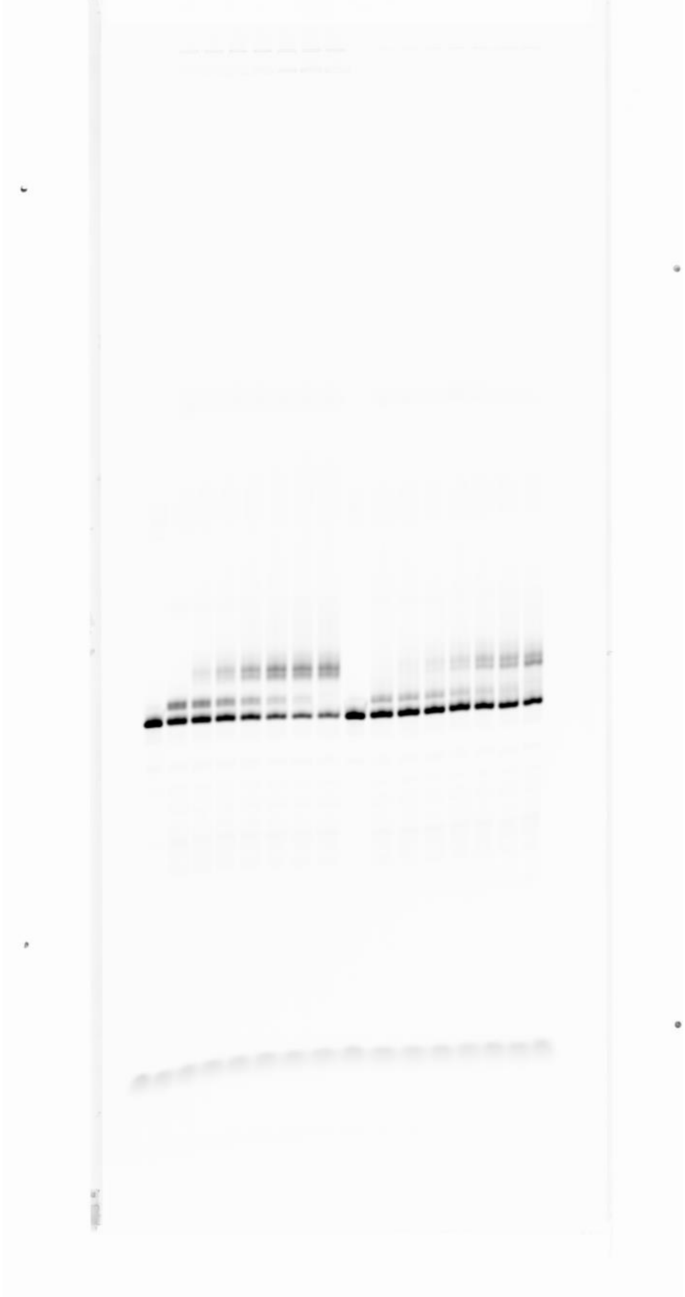

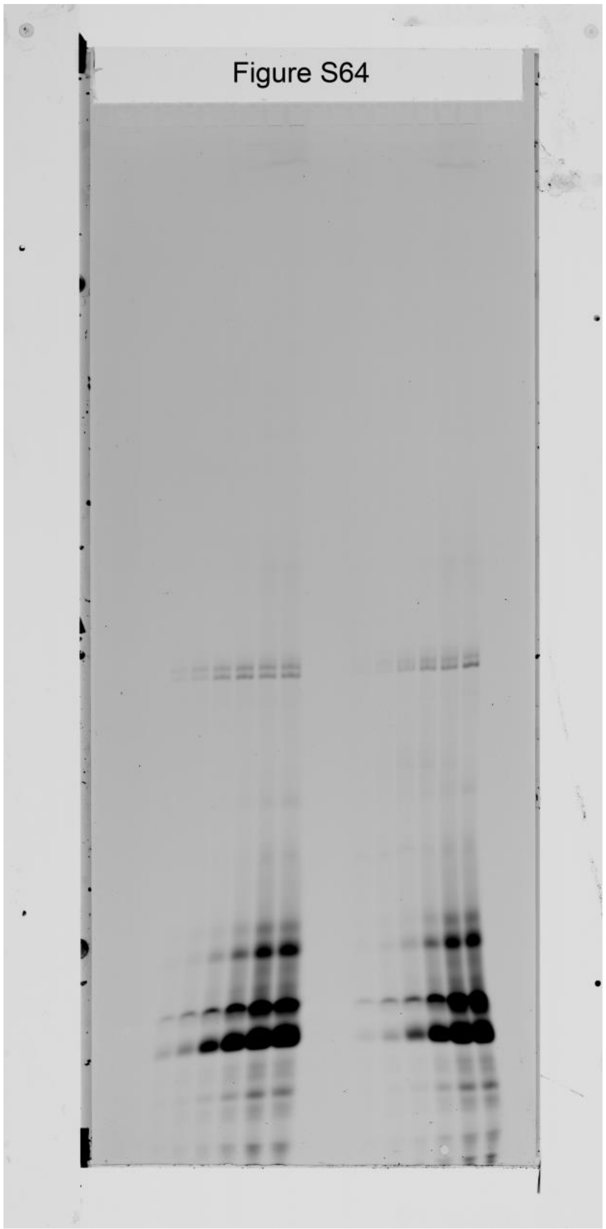
Figure S64

Figure S66

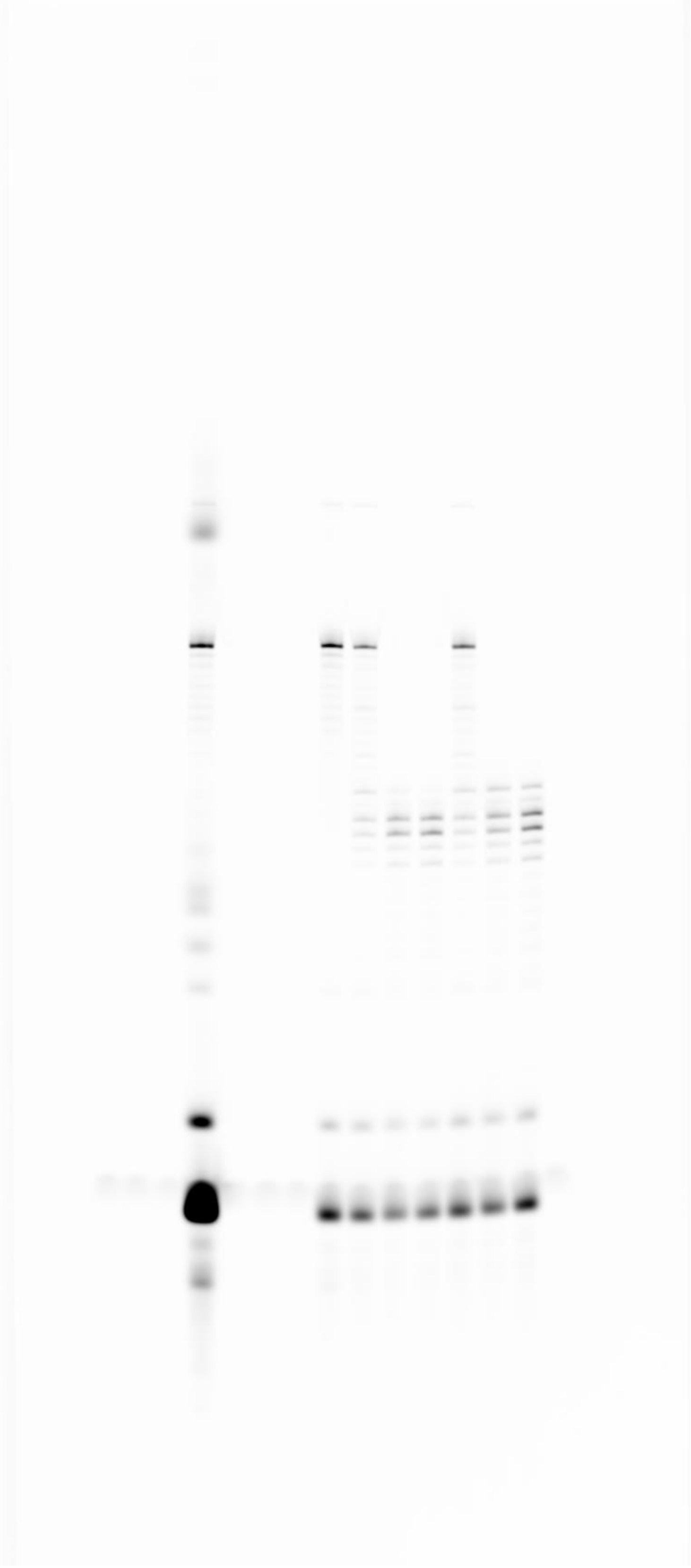

Figure S66

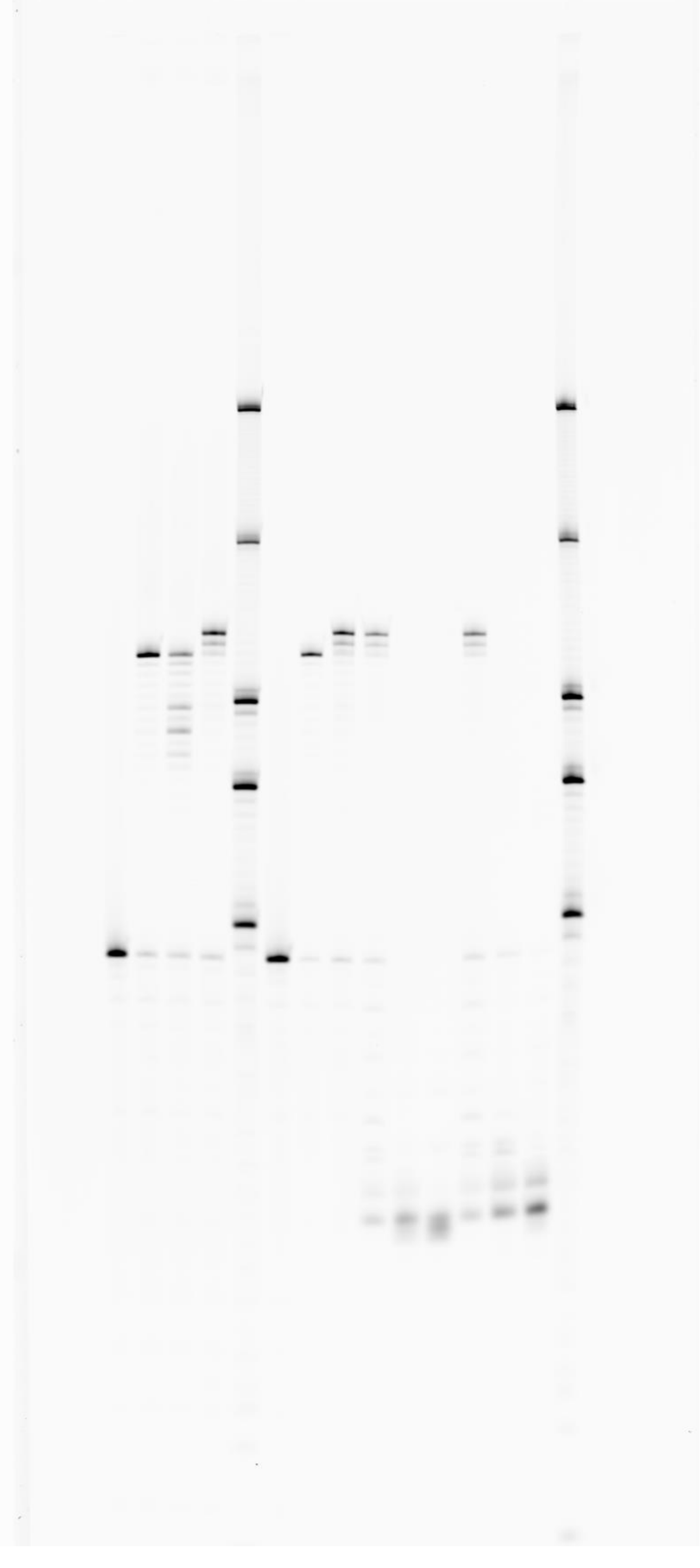

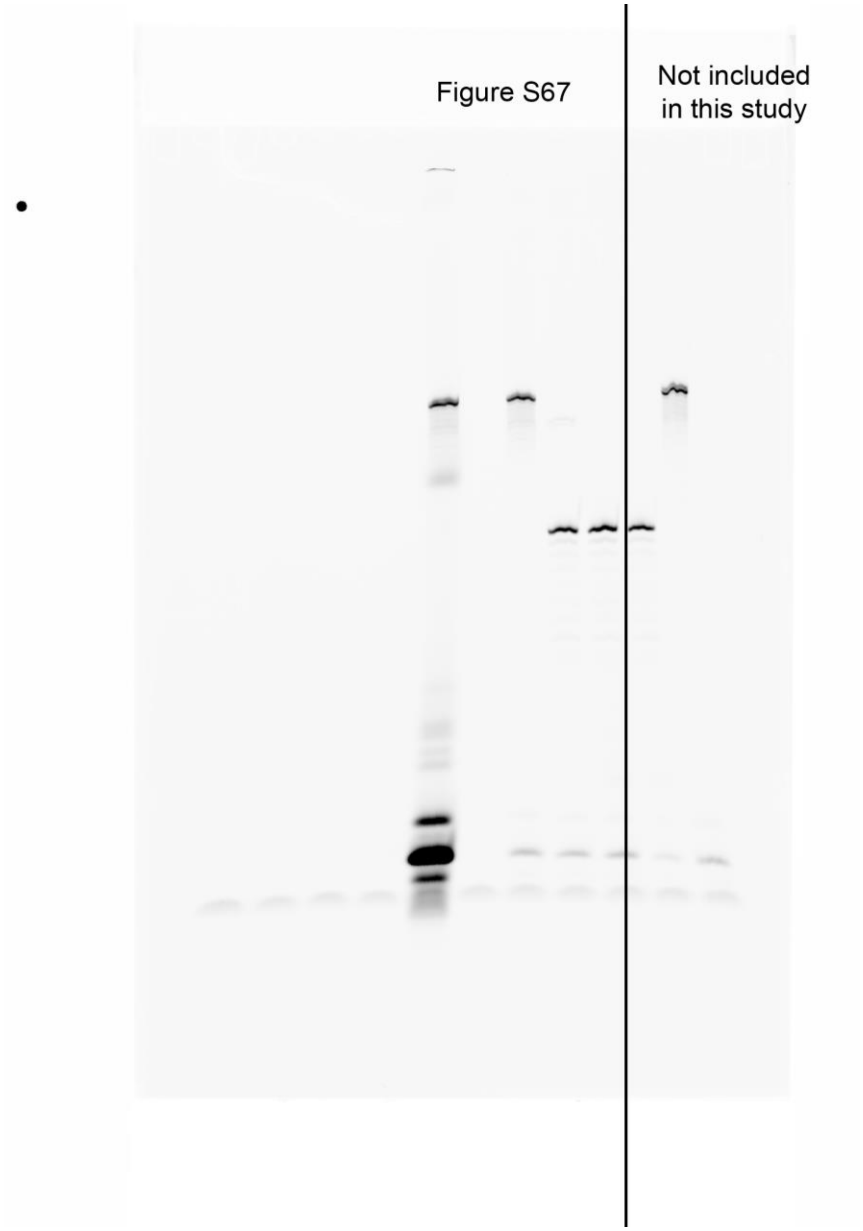
Figure S67
Not included
in this study

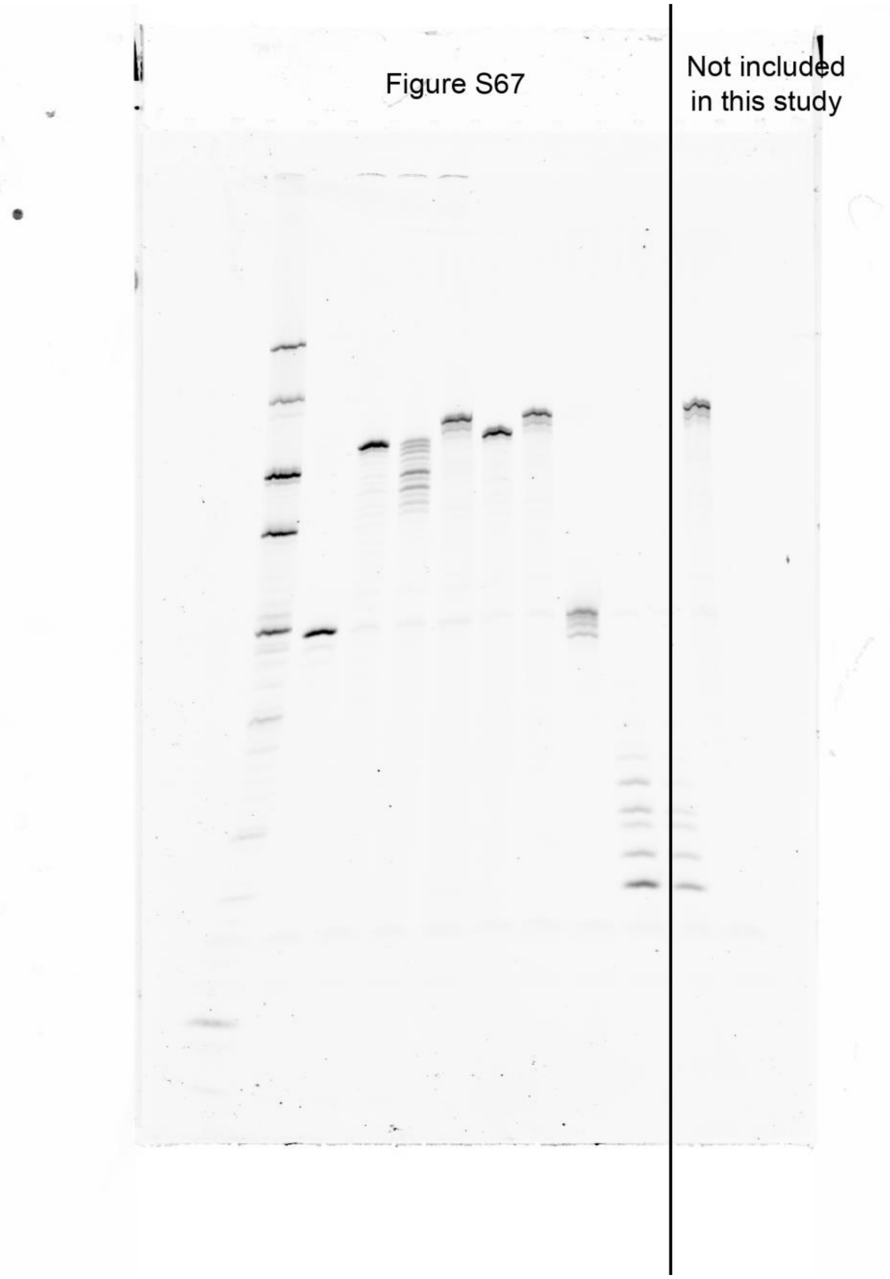
Figure S67
Not included
in this study

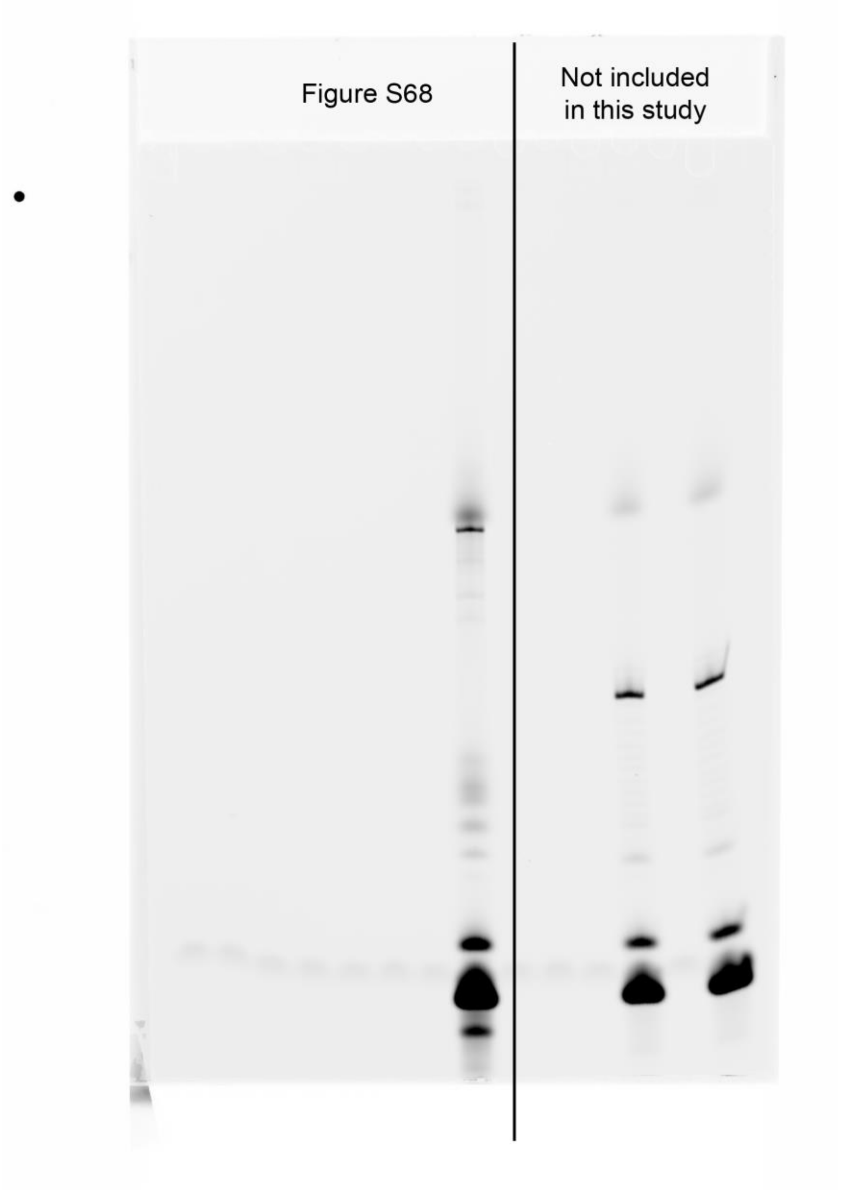
Figure S68
Not included
in this study

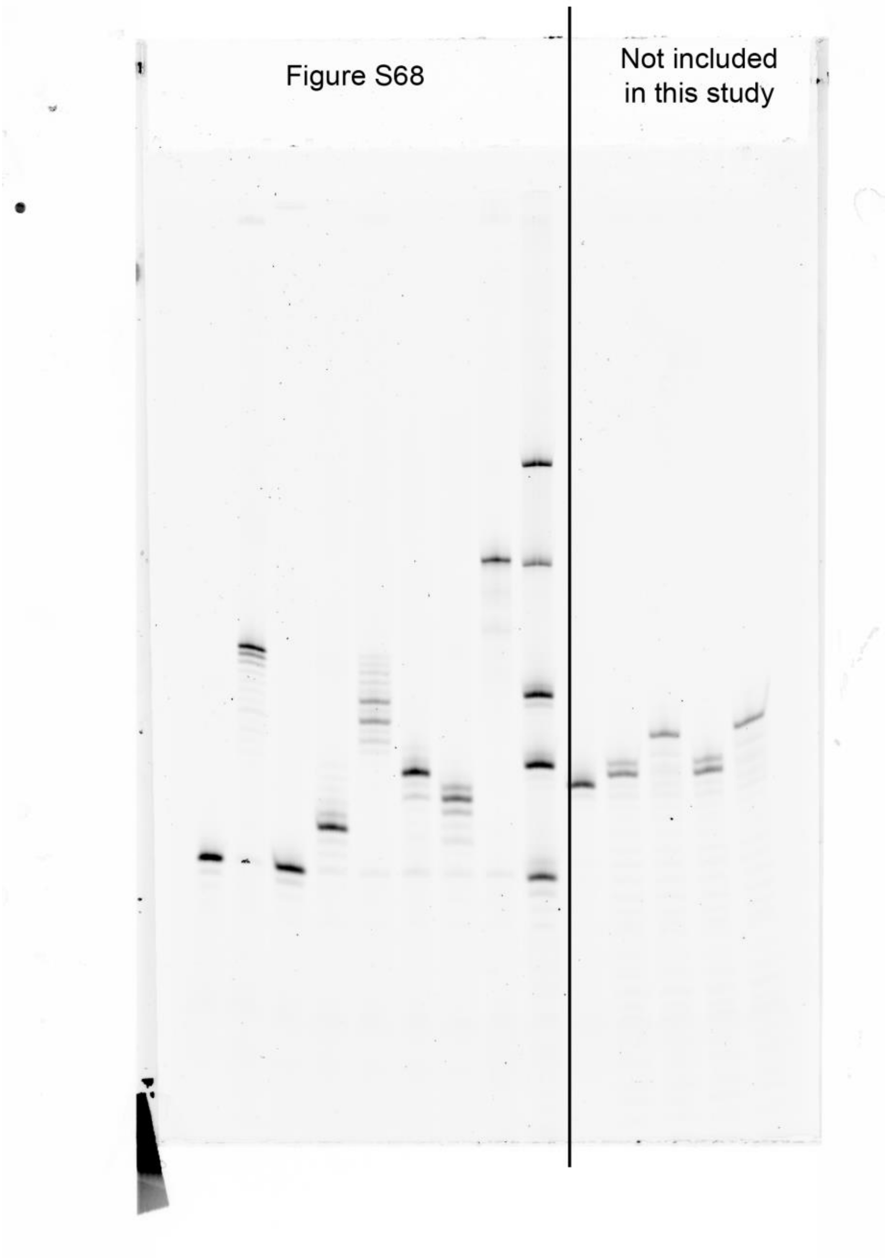
Figure S68
Not included in this study

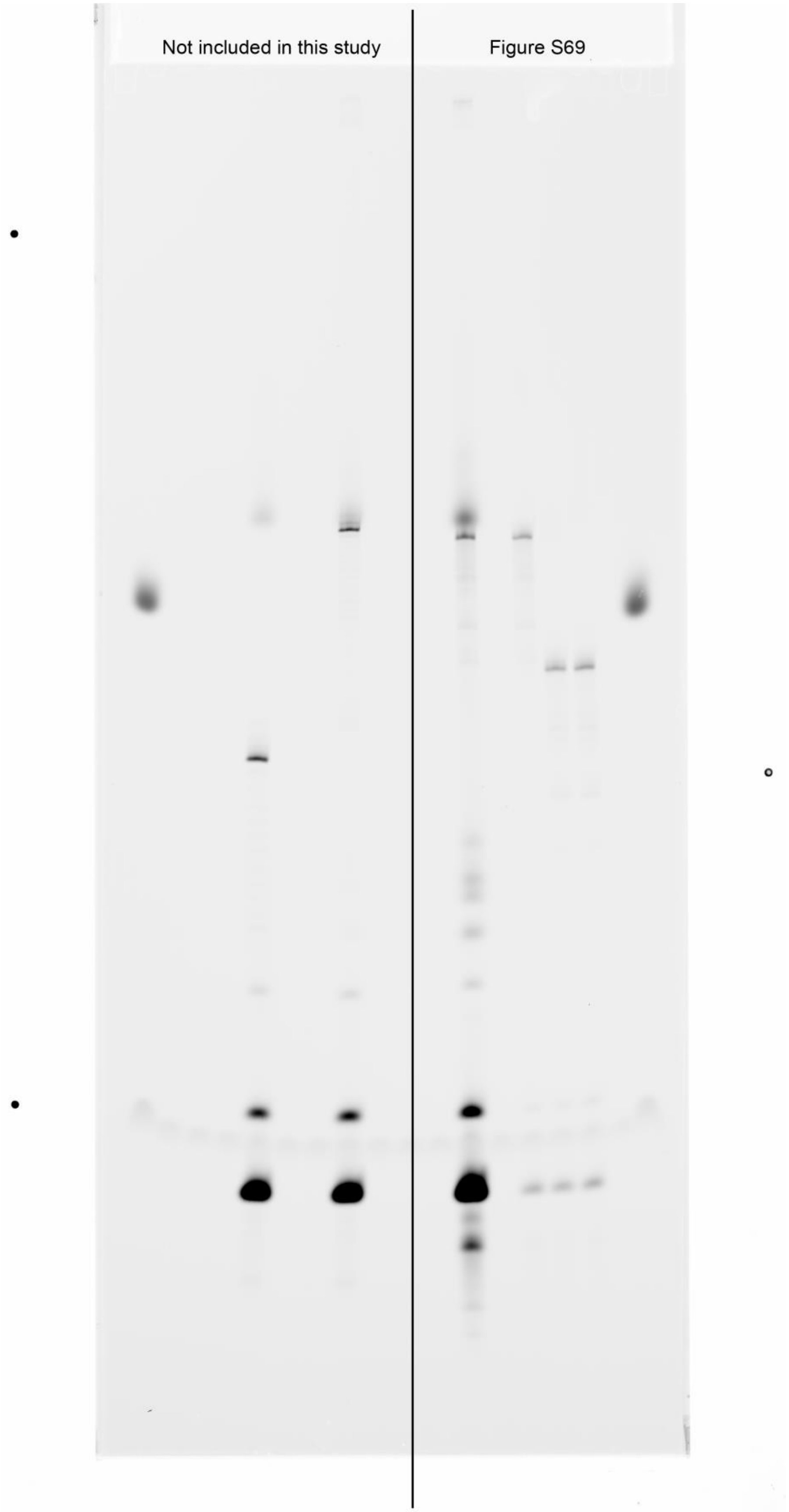
Not included in this study
Figure S69

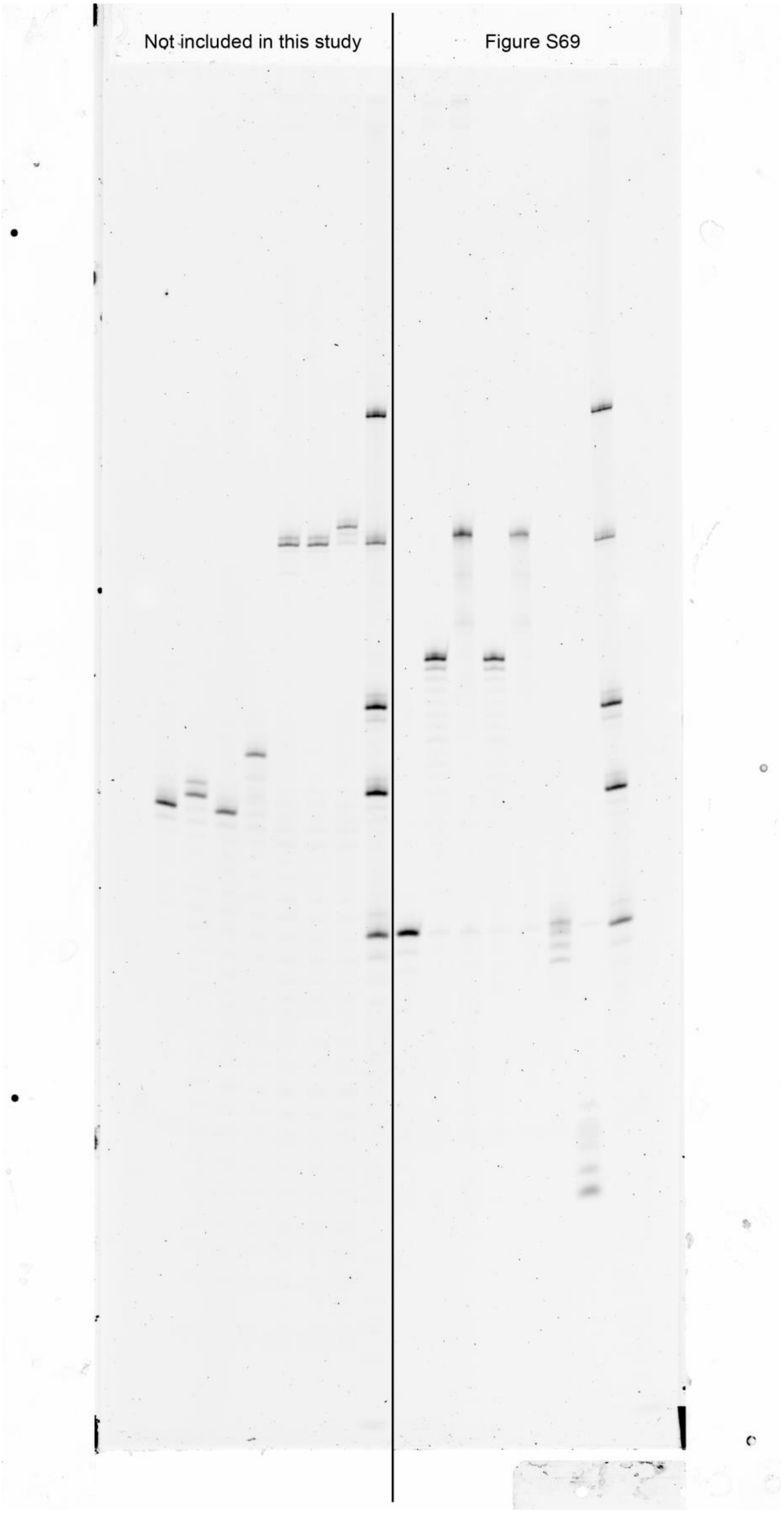
Not included in this study
Figure S69

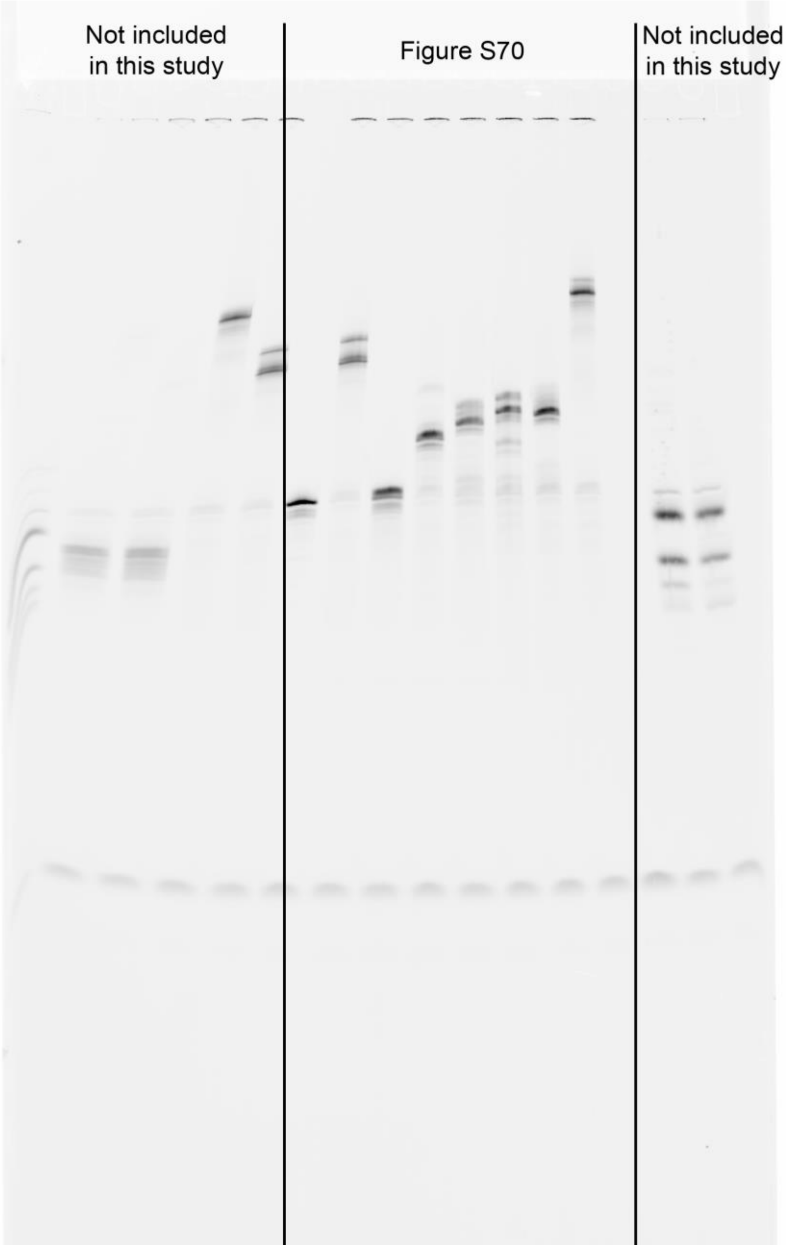
Not included
in this study
Figure S70
Not included
in this study

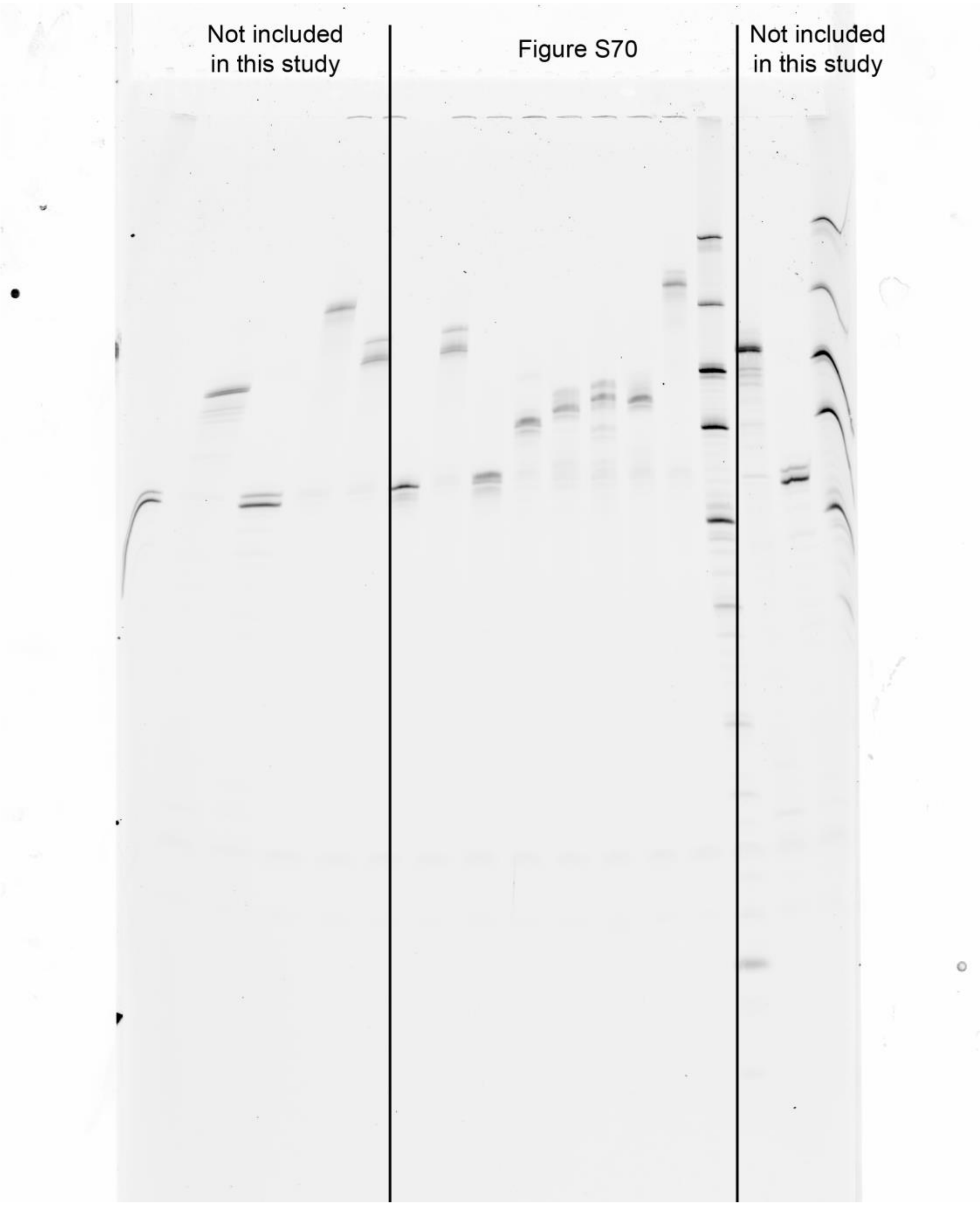
Not included in this study
Figure S70
Not included in this study

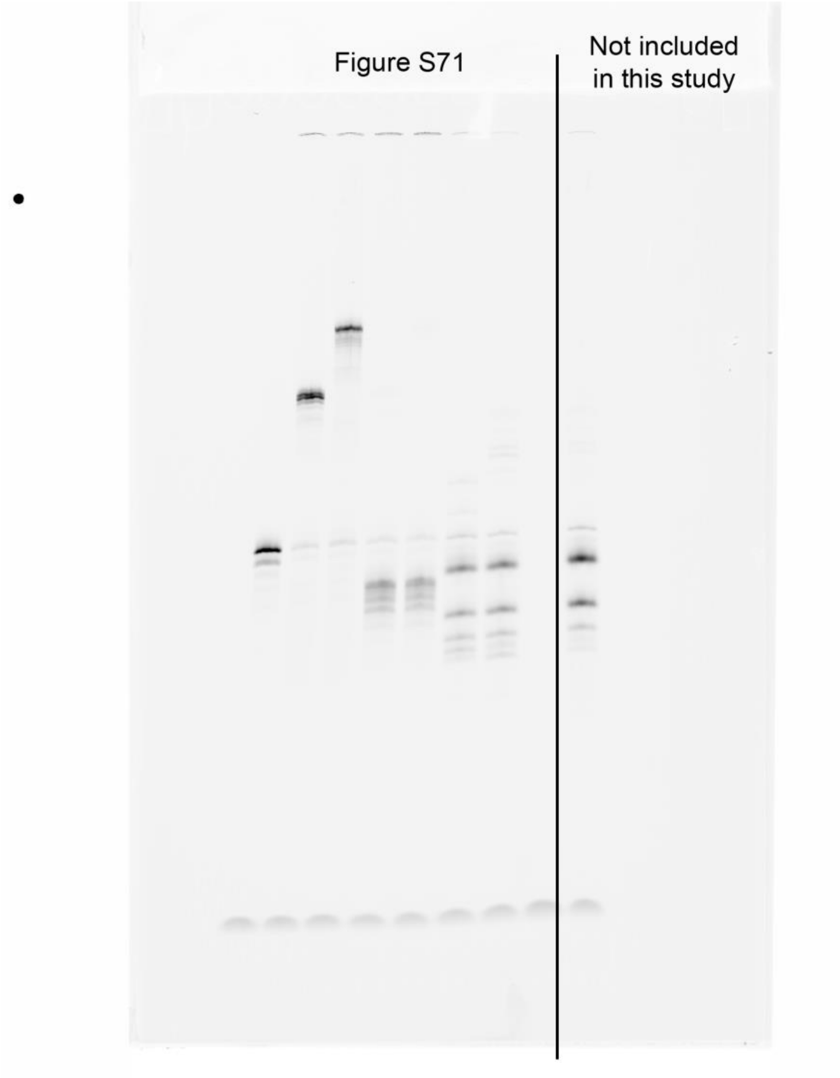
Figure S71
Not included in this study

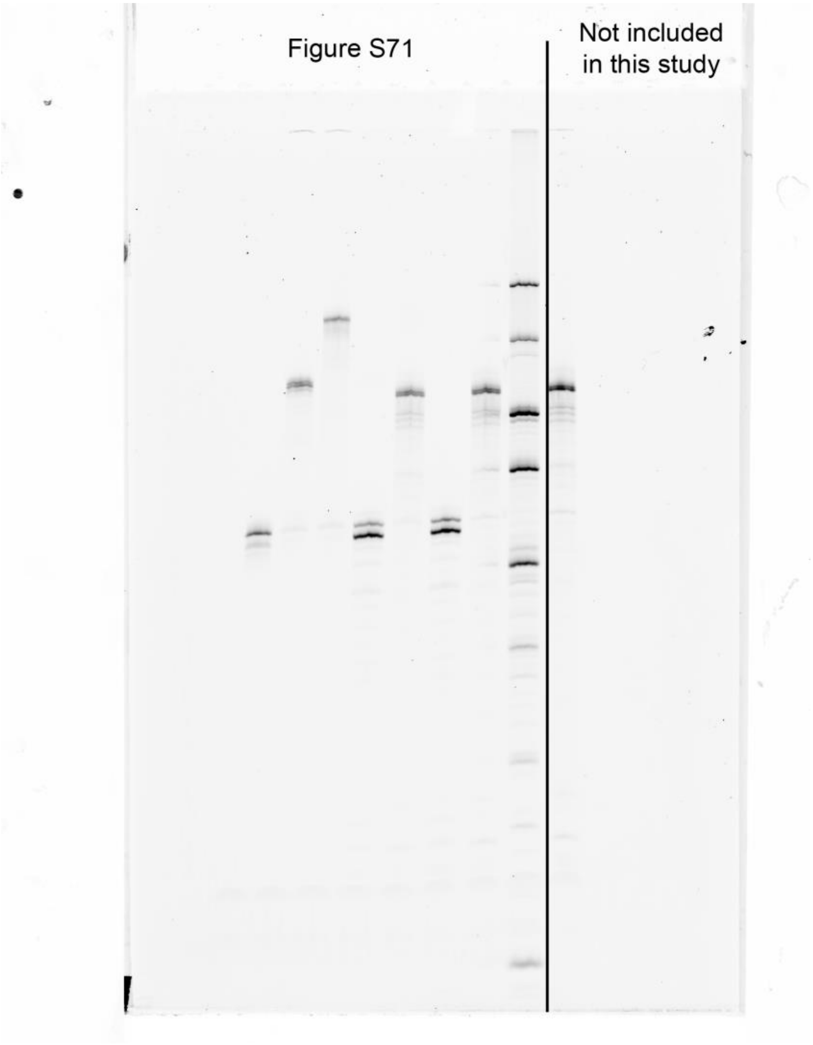
Figure S71
Not included
in this study

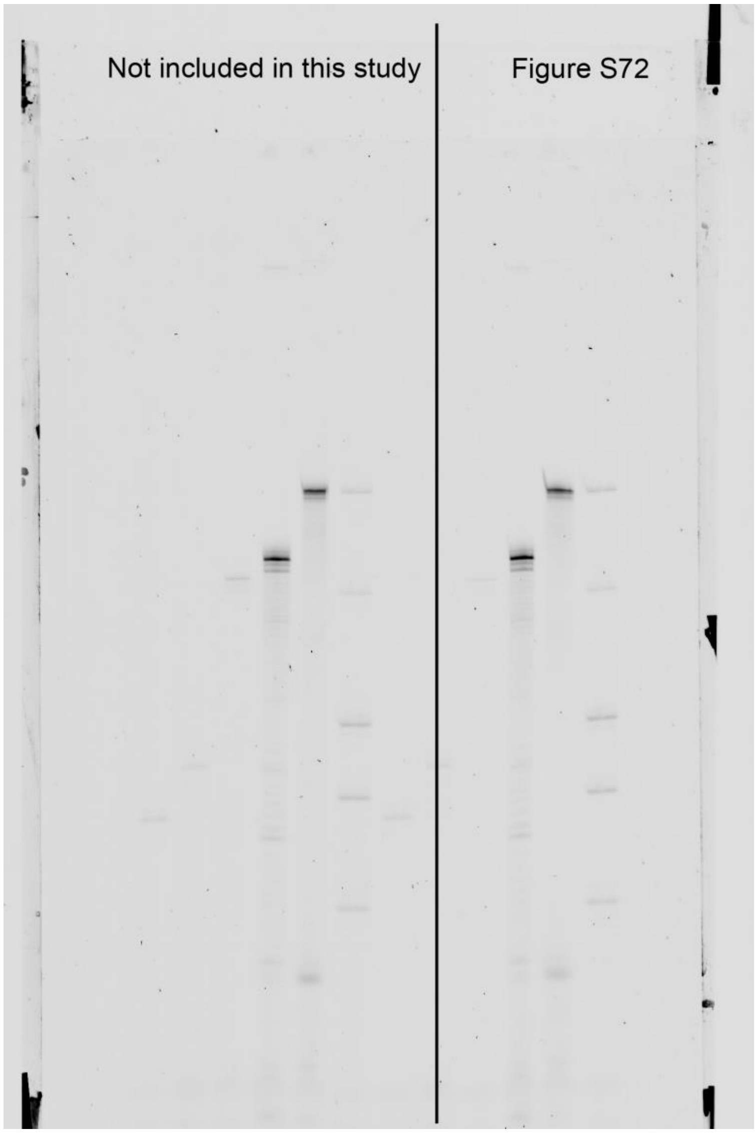
Not included in this study
Figure S72

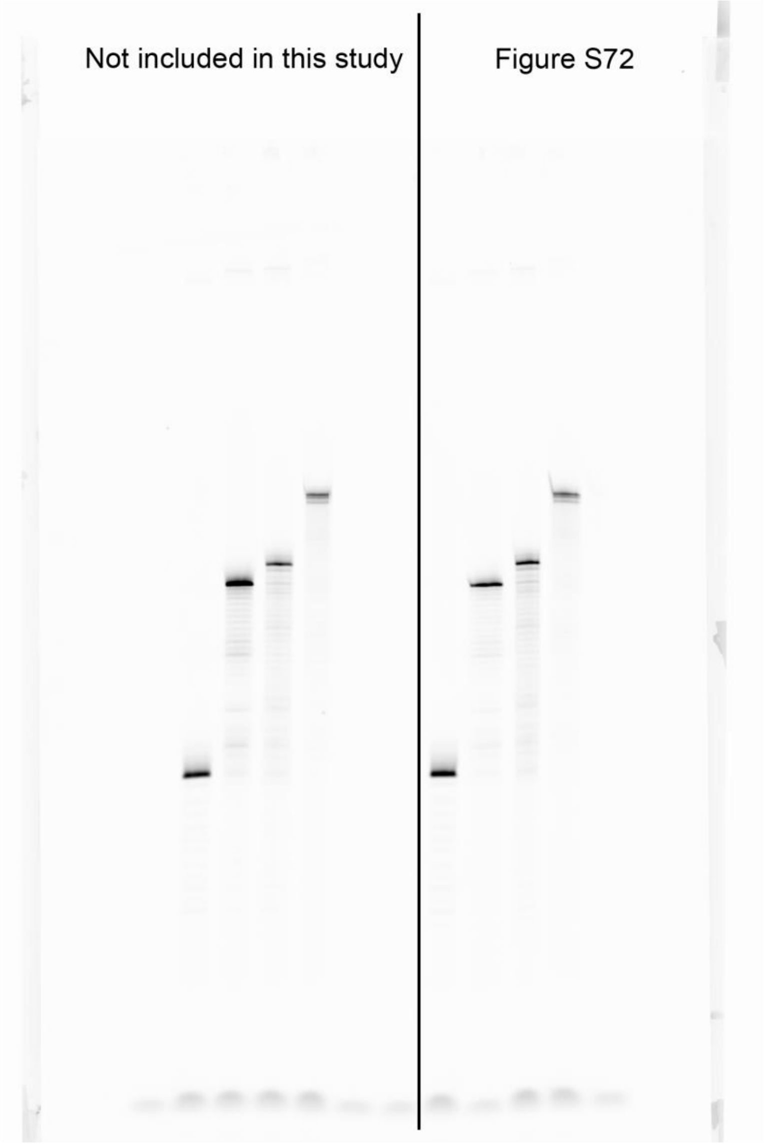
Not included in this study
Figure S72

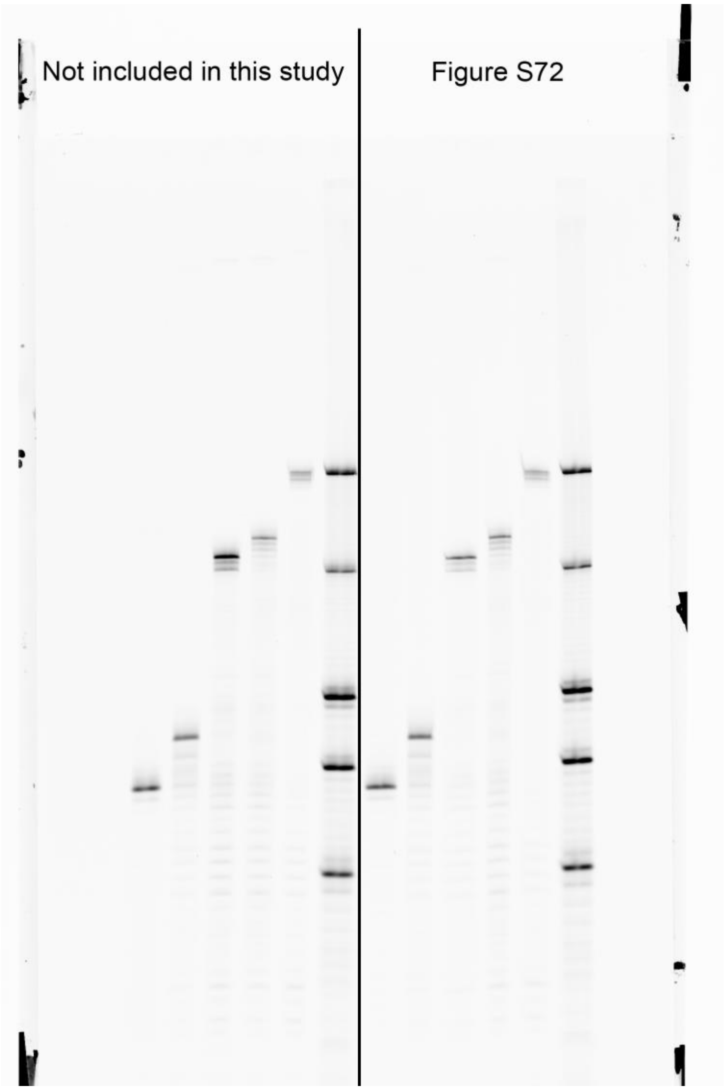
Not included in this study
Figure S72

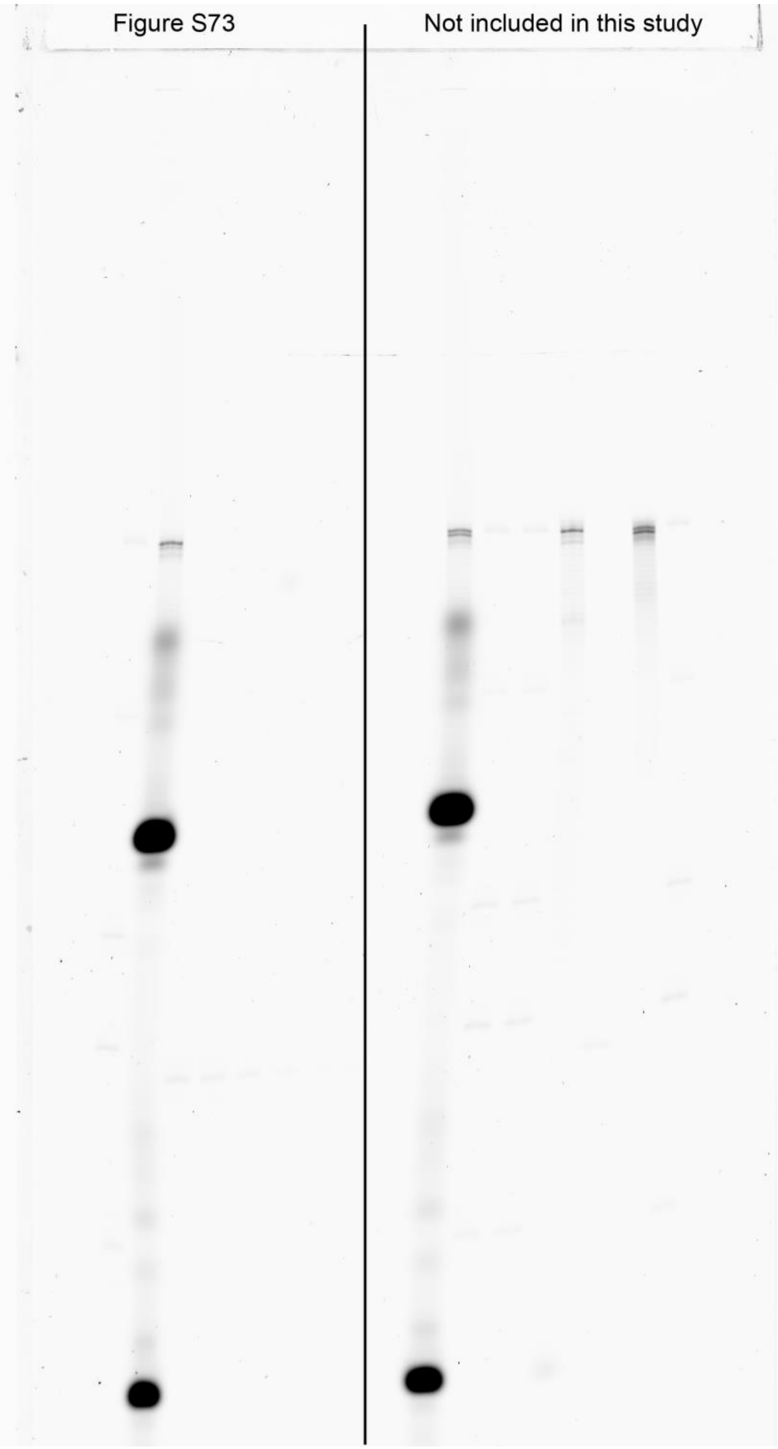
Figure S73
Not included in this study

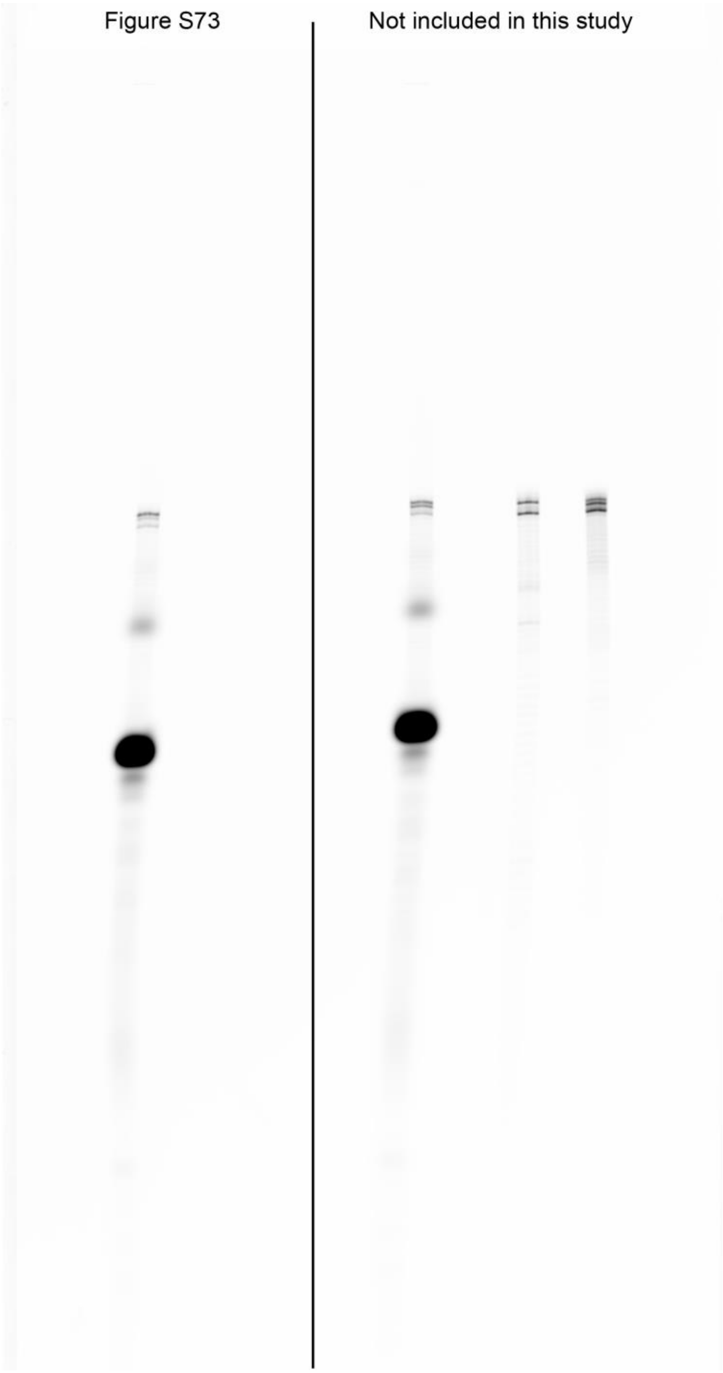
Figure S73
Not included in this study

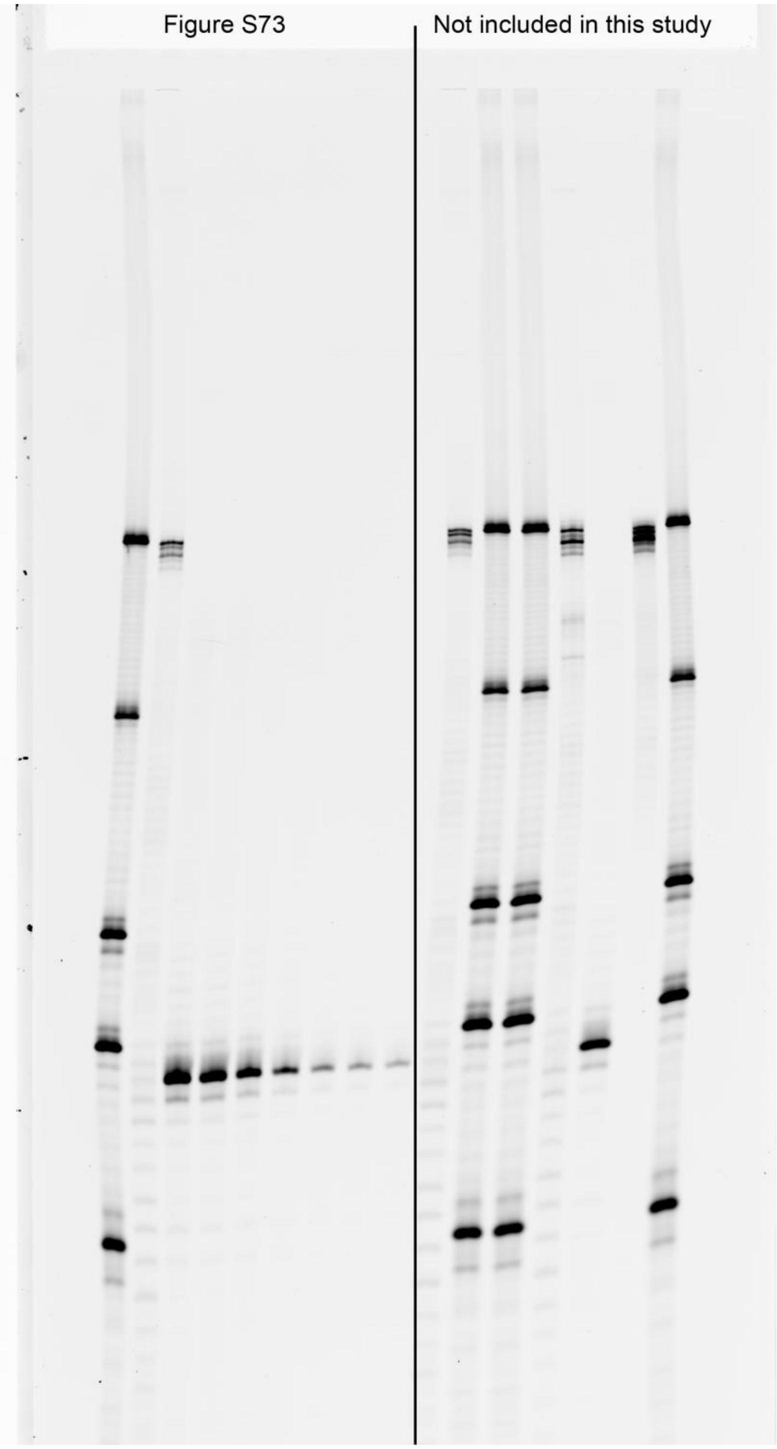

Figure S73
Not included in this study

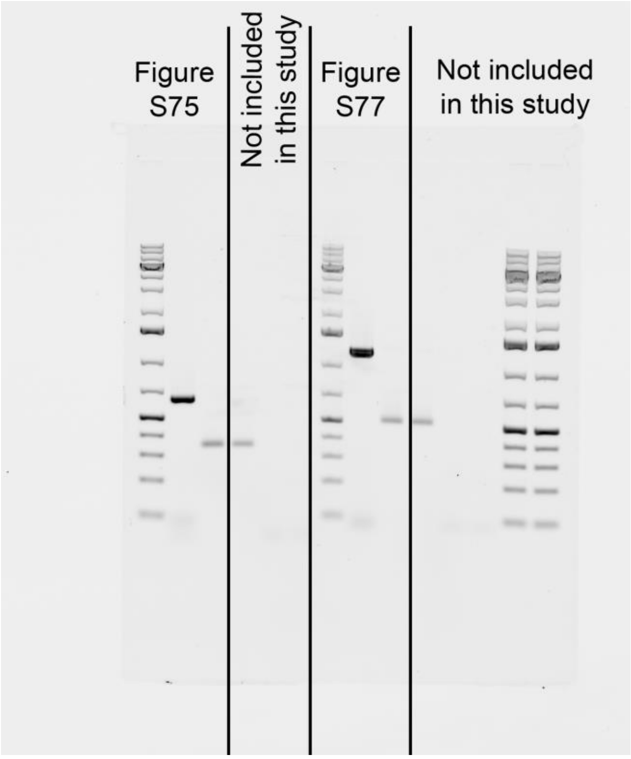

Figure S75
Not included in this study
Figure S77
Not included in this study

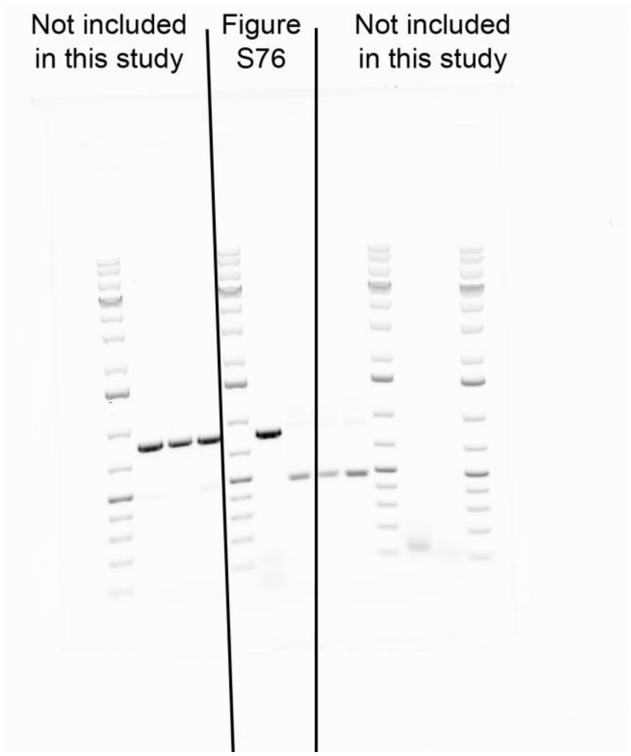

Not included in this study
Figure S76
Not included in this study

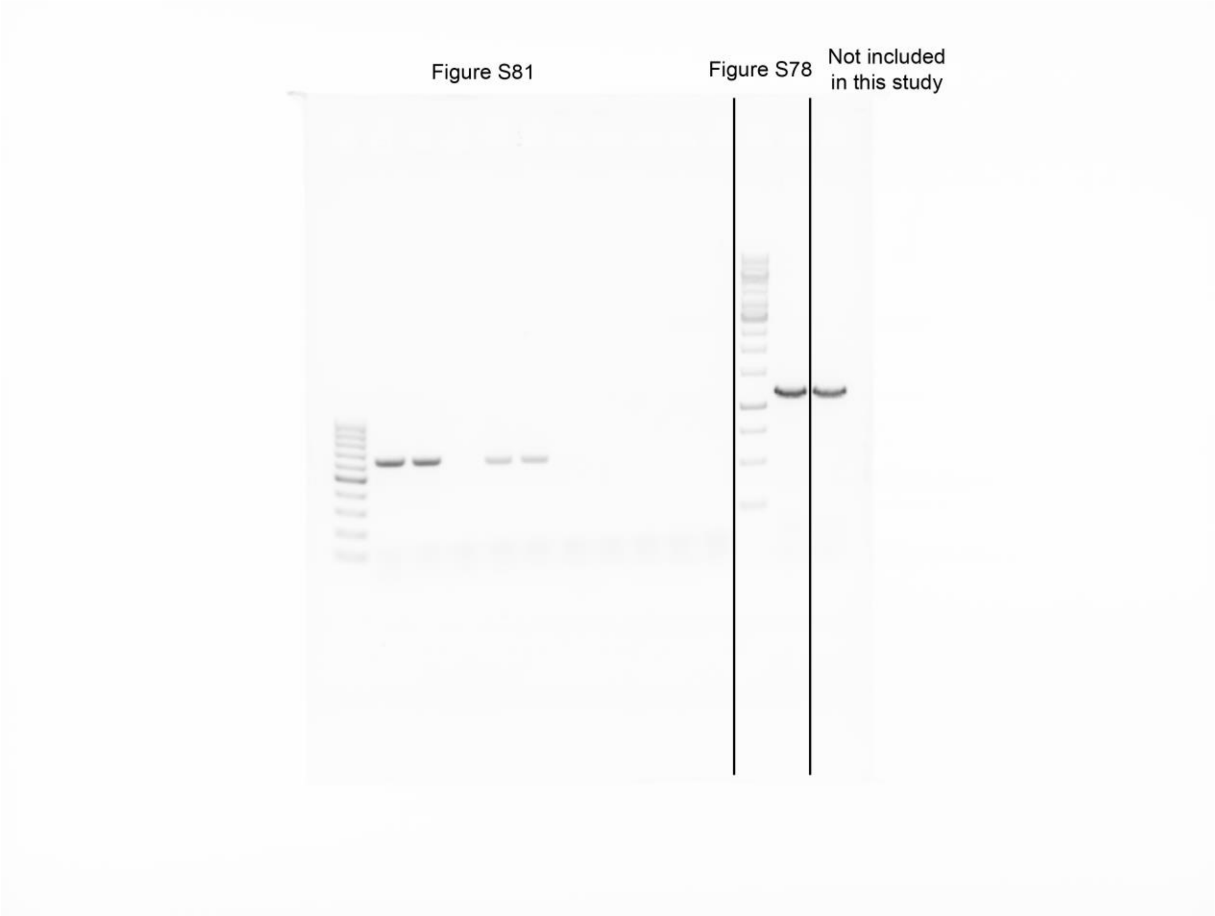
Figure S81
Figure S78
Not included in this study

Figure S79

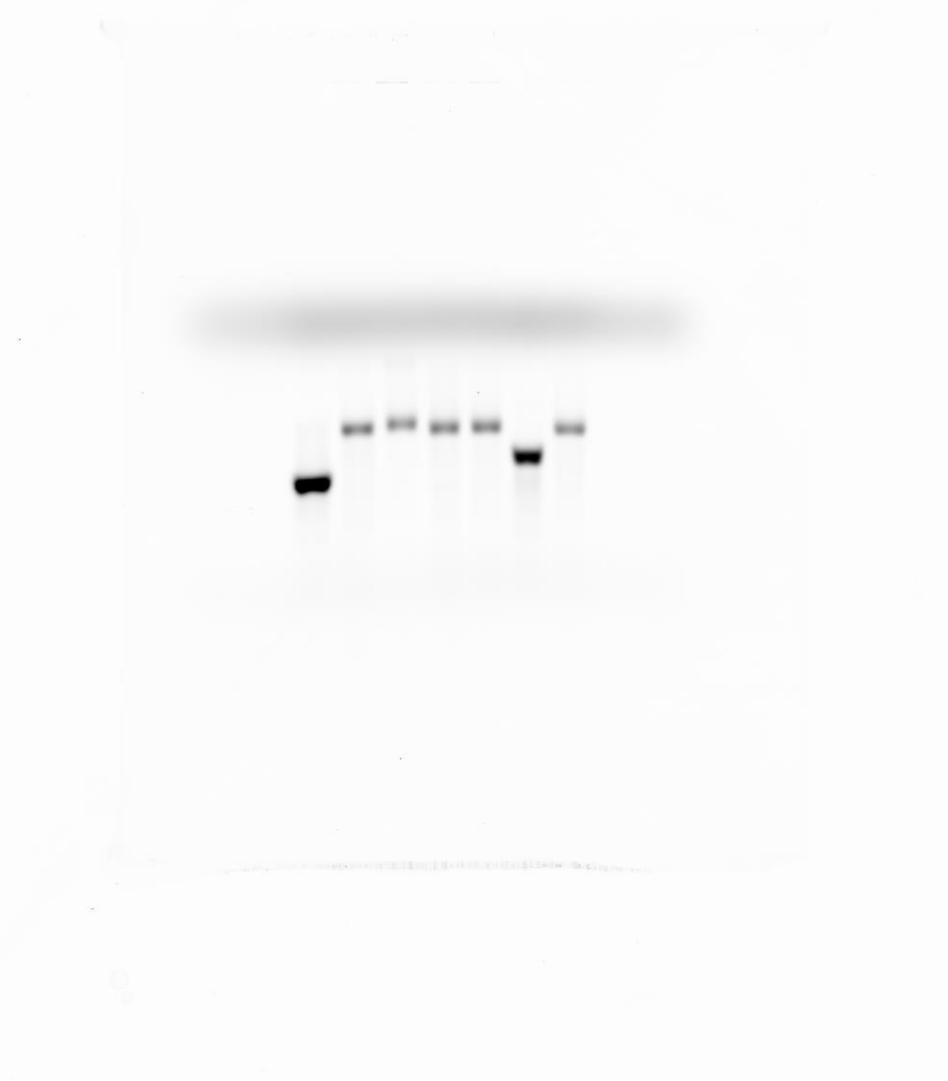

Figure S79

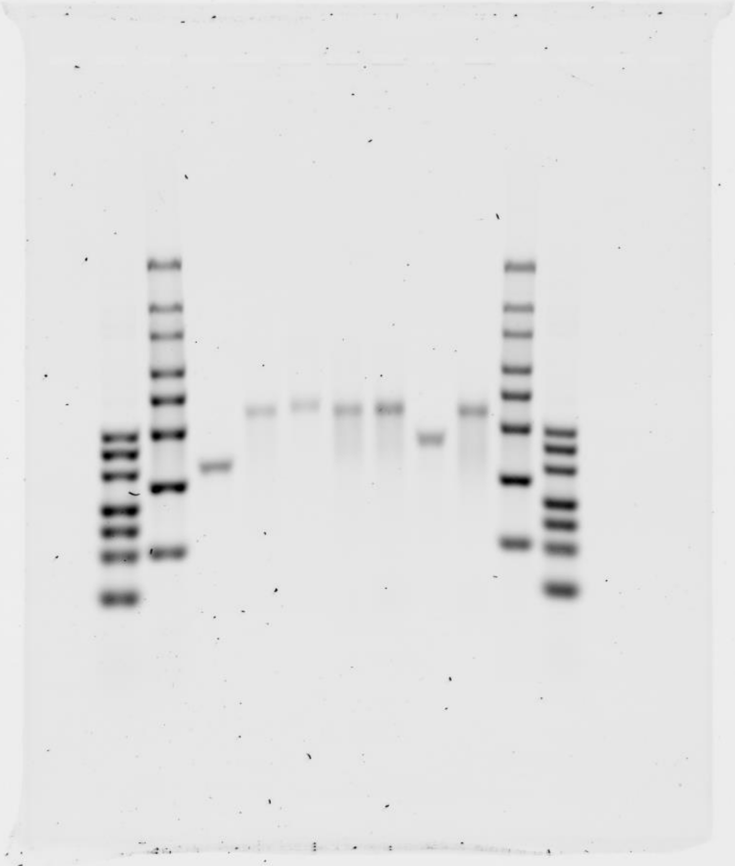

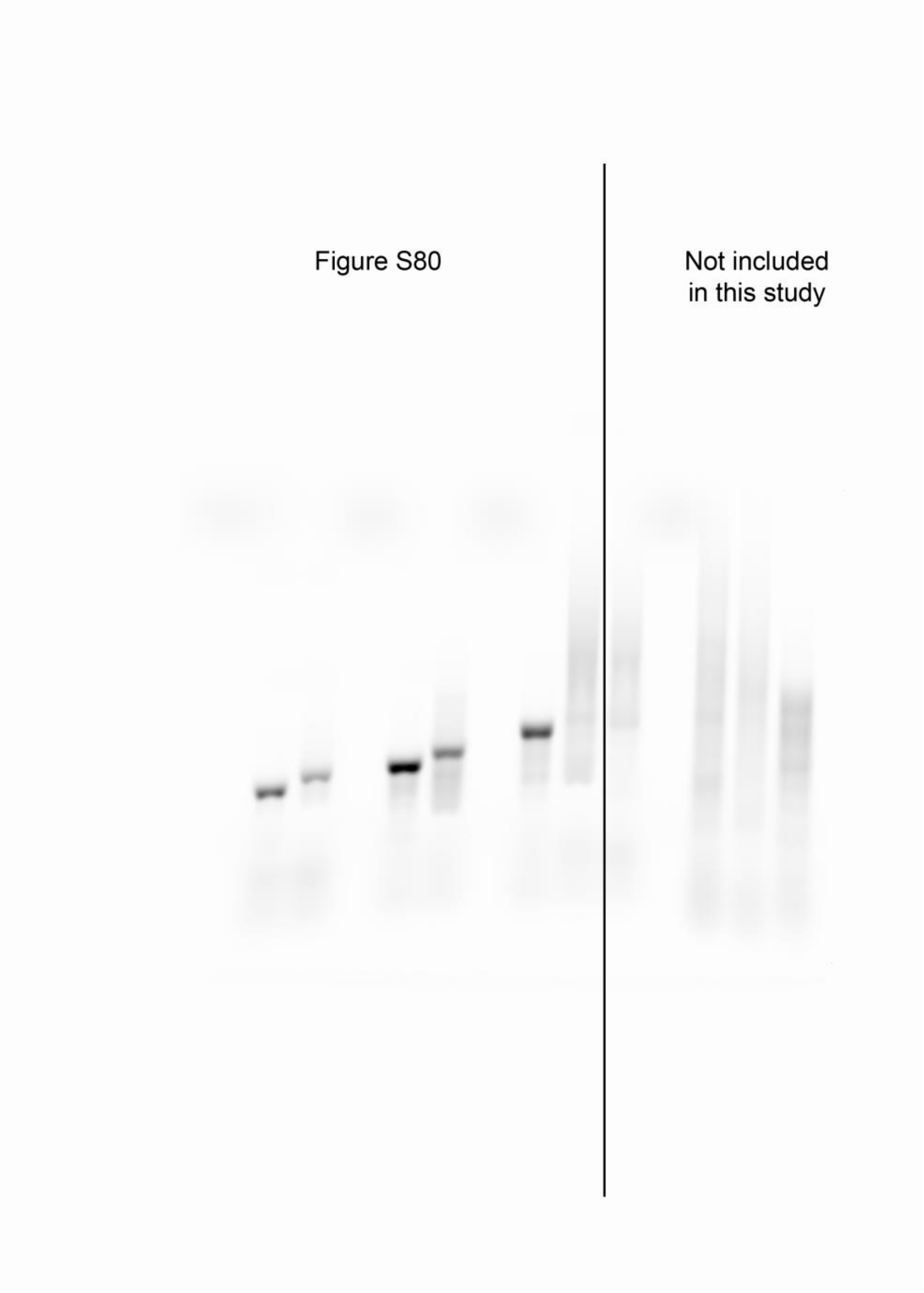
Figure S80
Not included
in this study

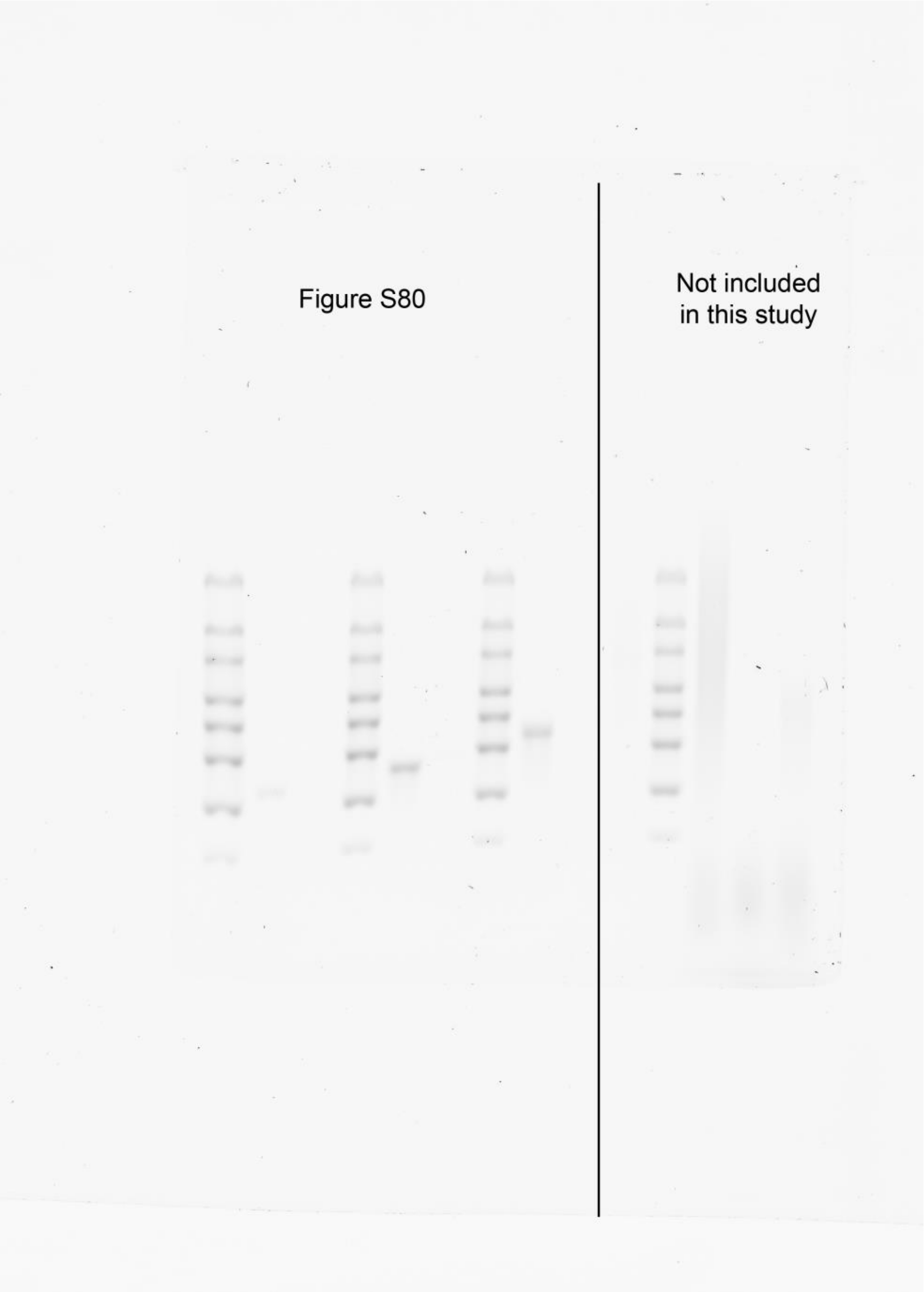
Figure S80
Not included in this study

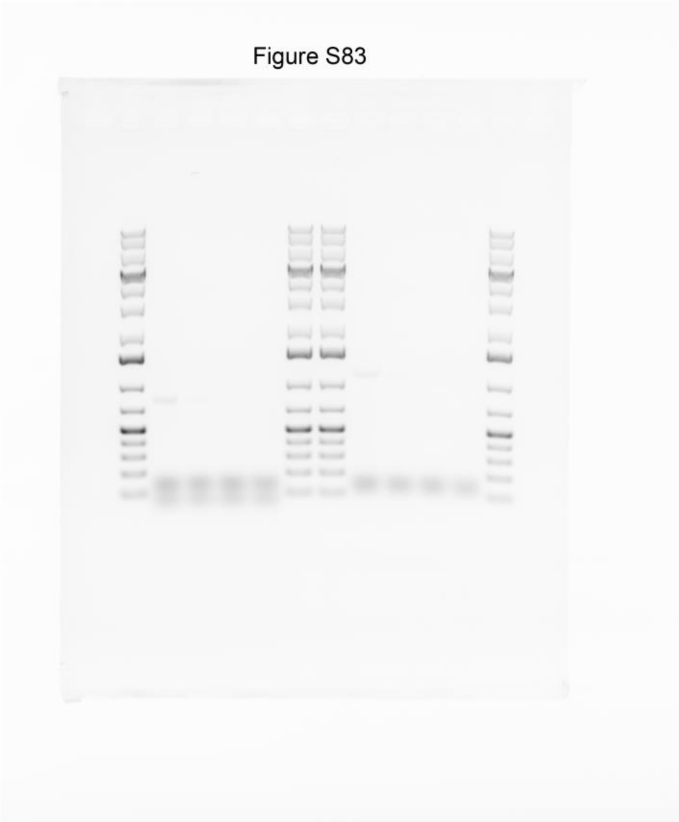
Figure S83

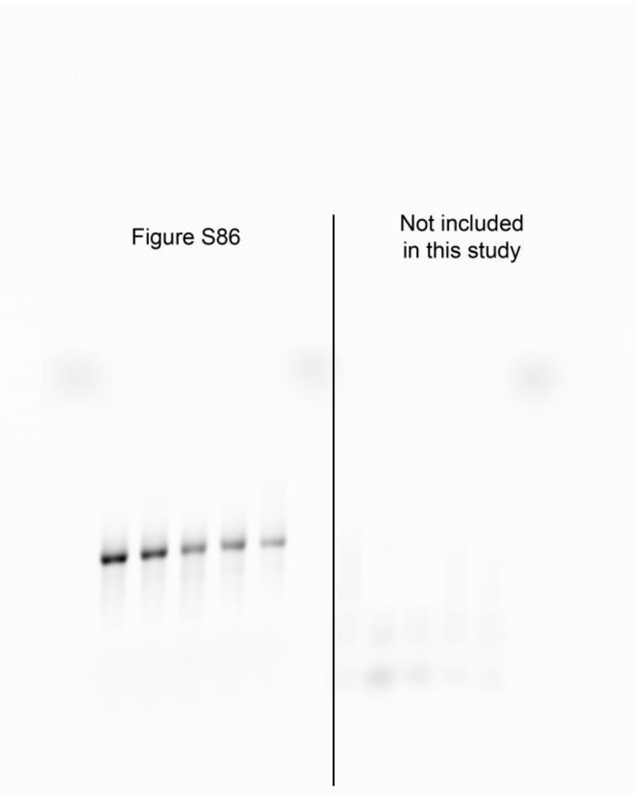
Figure S86
Not included
in this study

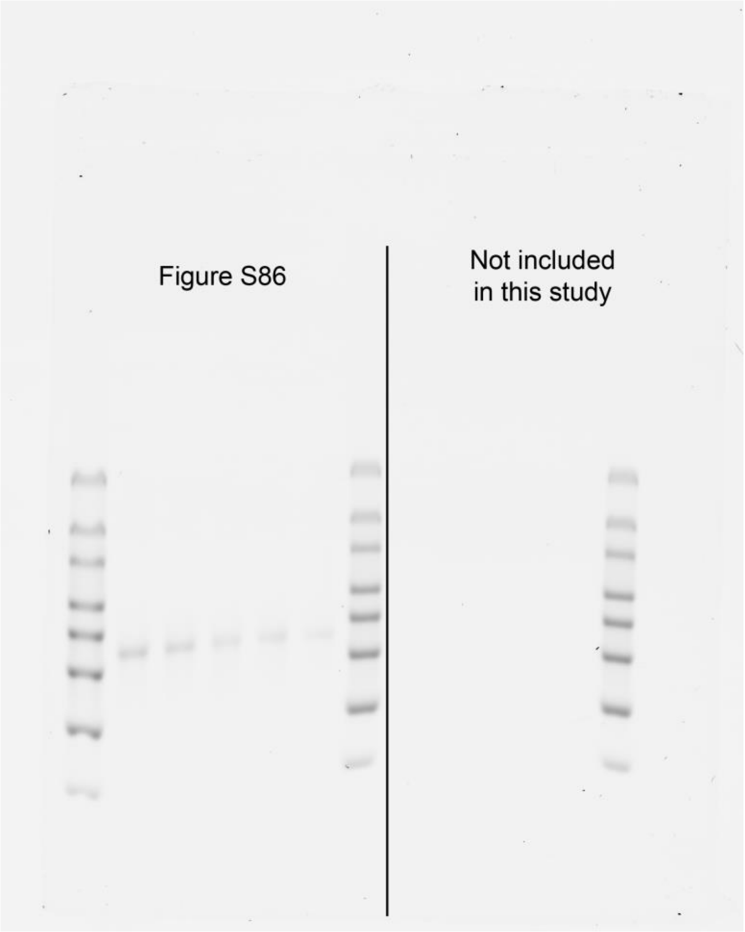
Figure S86
Not included in this study

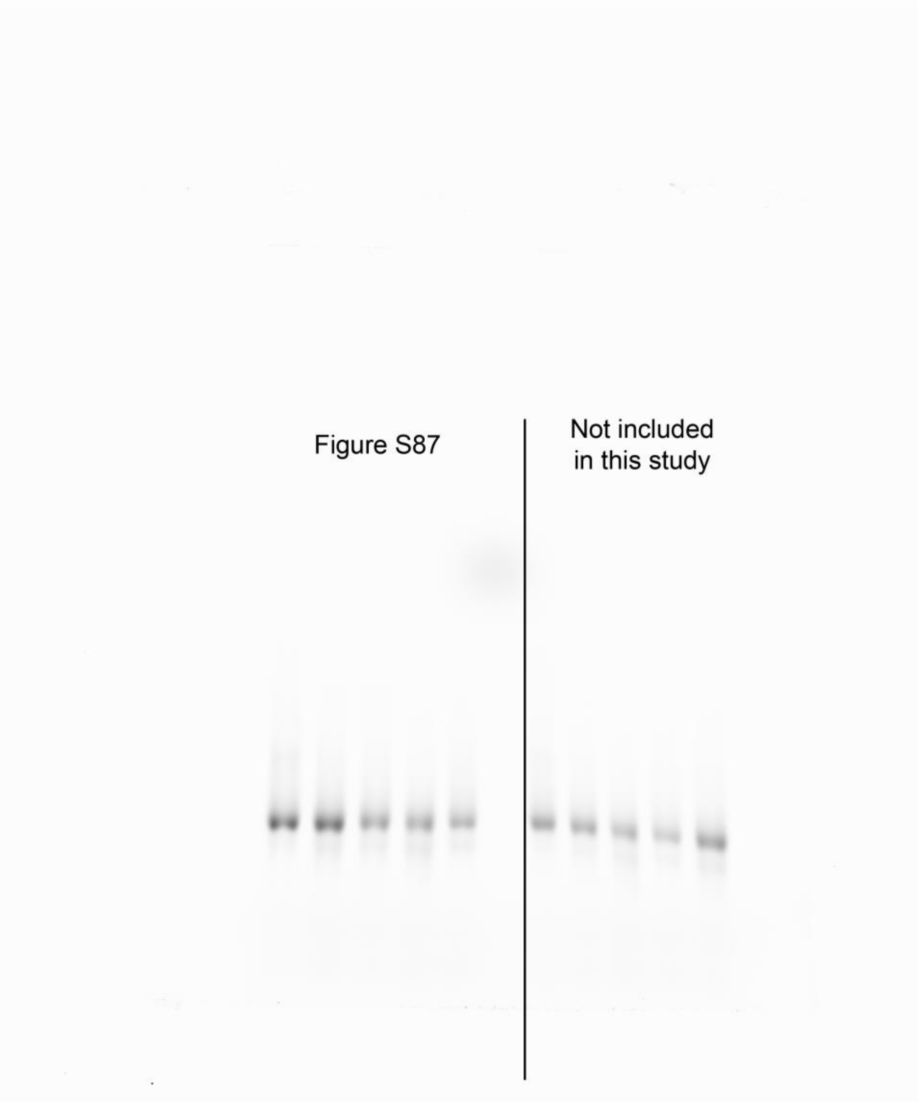
Figure S87
Not included in this study

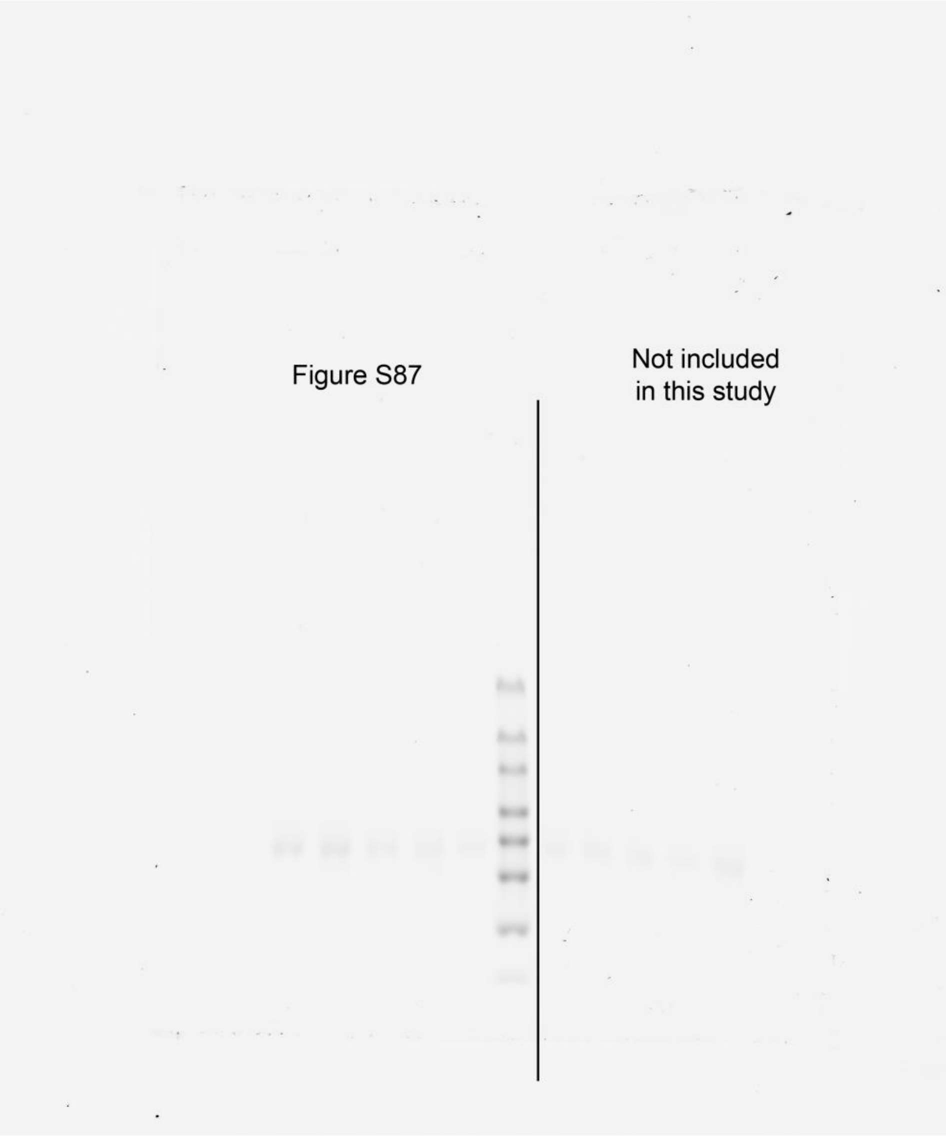
Figure S87
Not included
in this study

Figure S88

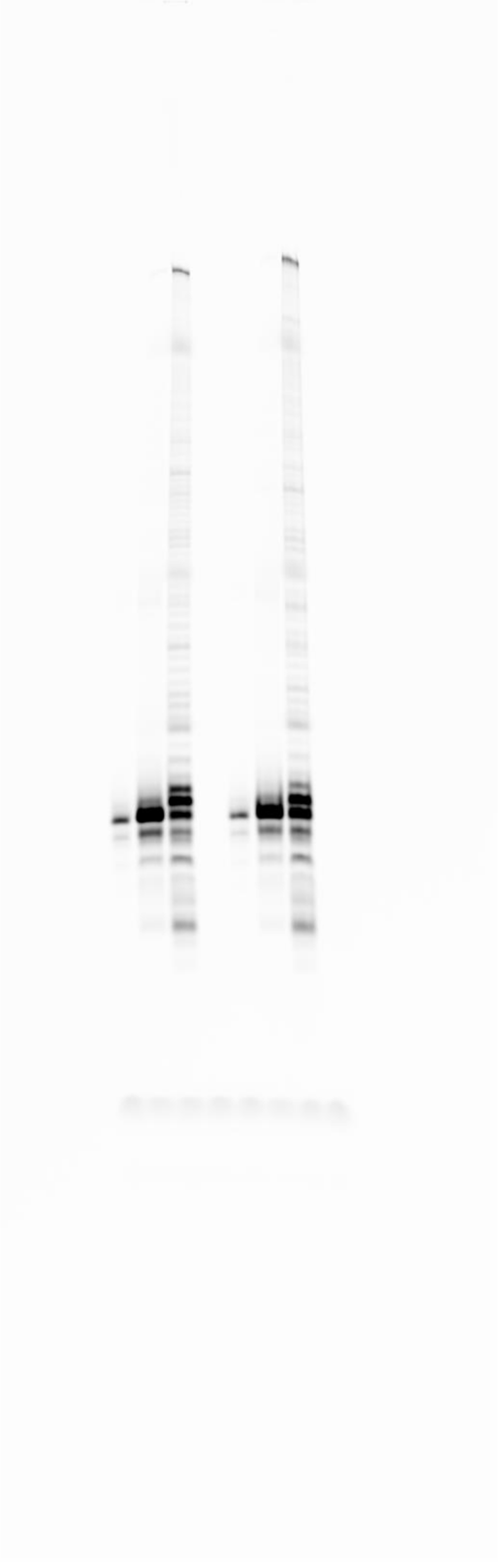

Figure S88

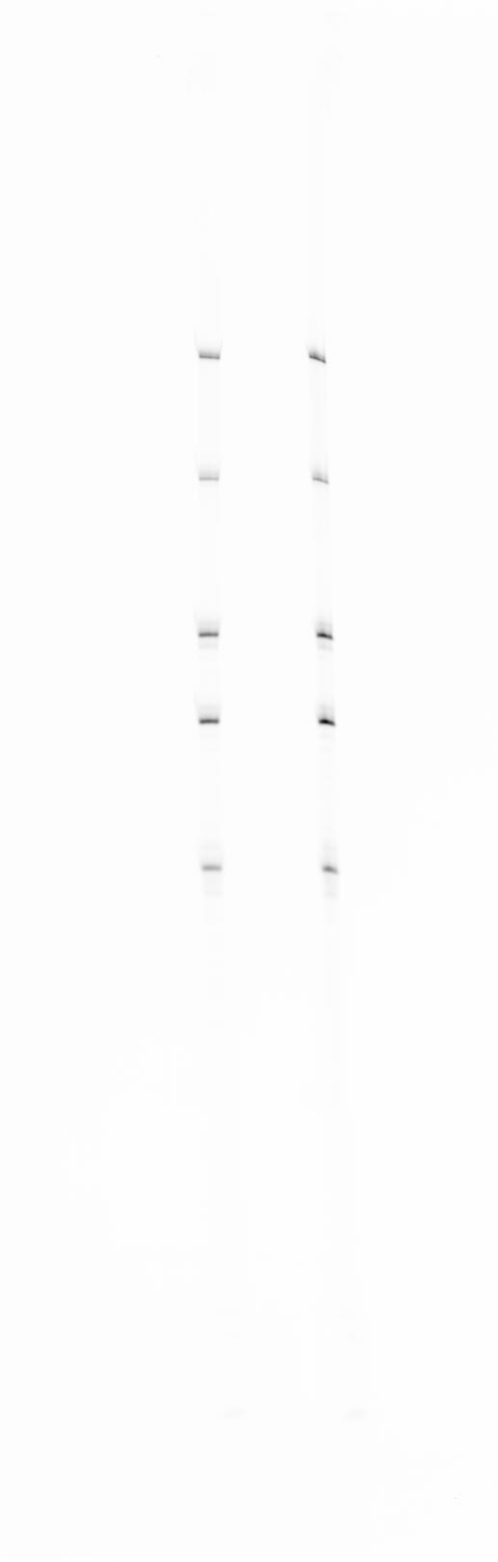

Supplement: Supplementary file 1 — Supplementary information [file 41467_2024_47444_MOESM1_ESM.pdf]
