# Supplementary material for: Expedient production of site specifically nucleobase-labelled or hypermodified RNA with engineered thermophilic DNA polymerases
Source: Nat Commun. 2024 Apr 9;15:3054. doi: 10.1038/s41467-024-47444-9 (PMC11004144; doi:10.1038/s41467-024-47444-9)
Supplement: Supplementary file 3 — Reporting Summary [file 41467_2024_47444_MOESM3_ESM.pdf]

nature portfolio

Corresponding author(s): Matouš Krömer, Michal Hocek

Last updated by author(s): Mar 16, 2024

Reporting Summary

Nature Portfolio wishes to improve the reproducibility of the work that we publish. This form provides structure for consistency and transparency in reporting. For further information on Nature Portfolio policies, see our Editorial Policies and the Editorial Policy Checklist.

Statistics

For all statistical analyses, confirm that the following items are present in the figure legend, table legend, main text, or Methods section.

n/a	Confirmed	
☐	☒	The exact sample size (*n*) for each experimental group/condition, given as a discrete number and unit of measurement
☐	☒	A statement on whether measurements were taken from distinct samples or whether the same sample was measured repeatedly
☐	☒	The statistical test(s) used AND whether they are one- or two-sided<br>*Only common tests should be described solely by name; describe more complex techniques in the Methods section.*
☒	☐	A description of all covariates tested
☐	☒	A description of any assumptions or corrections, such as tests of normality and adjustment for multiple comparisons
☐	☒	A full description of the statistical parameters including central tendency (e.g. means) or other basic estimates (e.g. regression coefficient) AND variation (e.g. standard deviation) or associated estimates of uncertainty (e.g. confidence intervals)
☐	☒	For null hypothesis testing, the test statistic (e.g. *F*, *t*, *r*) with confidence intervals, effect sizes, degrees of freedom and *P* value noted<br>*Give P values as exact values whenever suitable.*
☒	☐	For Bayesian analysis, information on the choice of priors and Markov chain Monte Carlo settings
☒	☐	For hierarchical and complex designs, identification of the appropriate level for tests and full reporting of outcomes
☒	☐	Estimates of effect sizes (e.g. Cohen's *d*, Pearson's *r*), indicating how they were calculated

Our web collection on statistics for biologists contains articles on many of the points above.

Software and code

Policy information about availability of computer code

Data collection	No software used beyond standard instrument operating software that was enclosed to following instruments: gel scans were acquired by Typhoon FLA 9500 (GE Healthcare), qPCR was carried out on C1000 Touch thermal cycler (Bio-Rad), plate assays were evaluated by Spark multimodal reader (Tecan), fluorescent spectra were measured on a Fluoromax 4 spectrofluorometer (HORIBA Scientific), LC-MS data were acquired at Agilent 1290 Infinity II Bio system.
Data analysis	GraphPad Prism (v. 9.5.1) was used to generate graphs and analysis data from FRET, in vitro and in cellulo translations.<br>NMR spectra were processed by MestReNova and/or Bruker TopSpin program.<br>ImageJ (v 1.54f) was employed for gel densitometric analyses.<br>UniDec (v. 6.0.3) was used for biomolecule MS raw spectra deconvolution.

For manuscripts utilizing custom algorithms or software that are central to the research but not yet described in published literature, software must be made available to editors and reviewers. We strongly encourage code deposition in a community repository (e.g. GitHub). See the Nature Portfolio guidelines for submitting code & software for further information.

Data

Policy information about availability of data

All manuscripts must include a data availability statement. This statement should provide the following information, where applicable:

- Accession codes, unique identifiers, or web links for publicly available datasets
- A description of any restrictions on data availability
- For clinical datasets or third party data, please ensure that the statement adheres to our policy

All data generated or analyzed during this study are included in this published article and its supplementary information files. Source data are provided with this paper in Source Data File.

Research involving human participants, their data, or biological material

Policy information about studies with human participants or human data. See also policy information about sex, gender (identity/presentation), and sexual orientation and race, ethnicity and racism.

Reporting on sex and gender	Not relevant, since no experiments on humans were perfomed in this study.
Reporting on race, ethnicity, or other socially relevant groupings	Not relevant, since no experiments on humans were perfomed in this study.
Population characteristics	Not relevant, since no experiments on humans were perfomed in this study.
Recruitment	Not relevant, since no experiments on humans were perfomed in this study.
Ethics oversight	Not relevant, since no experiments on humans were perfomed in this study.

Note that full information on the approval of the study protocol must also be provided in the manuscript.

Field-specific reporting

Please select the one below that is the best fit for your research. If you are not sure, read the appropriate sections before making your selection.

☒ Life sciences ☐ Behavioural & social sciences ☐ Ecological, evolutionary & environmental sciences

For a reference copy of the document with all sections, see nature.com/documents/nr-reporting-summary-flat.pdf

Life sciences study design

All studies must disclose on these points even when the disclosure is negative.

Sample size	No sample size calculations were performed. Since obtained data from all replicates, when applicable, were consistent, no further replications were carried out.
Data exclusions	No data was excluded.
Replication	In FRET measurements we carried out 5 replicates for riboswitch and 3 replicates for negative controls. Since acquired points were consistent, we concluded this number to be sufficient.<br>For both in vitro and in cellulo mRNA translation studies we carried out 3 replicates for all conditions and samples. Since acquired points were consistent, we concluded this number to be sufficient.<br>For gel quantification of large scale PEX we carried out two replicates that were assayed and reported separately. As the resulting values were consistent, no further replications were neccessary. This reflects the consensus in the field of nucleic acids research.<br>For enzyme kinetics two replicates were carried out with RNA primer and single experiment only for DNA primer since semi-quantitative conclusions were drawn from these experiments and this corresponds to the standard of the field. Single-site riboswitch labelling for FRET measurements were replicated three times in total with similar, consistent results.<br>DNA primer removal experiments were carried two times in total with consistent results.
Randomization	Randomization was not performed and samples were not allocated in experimental groups. Biochemical experiments were performed with purified and characterised (bio)molecules. In vitro translations were conducted with standard commercially available lysates with well-described variability. In cellulo translations were performed in standardized established cell lines with no reported unusual variability. Cells were seeded at the same time using a single batch of cells and were treated in parallel. Identical culture plate, incubator and batch of transfection reagents were used for each replicate.
Blinding	Blinding was not relevant because animal or human participants were not used in this study and no allocations to experimental groups were performed and all quantitative measurements were carried out unbiasedly, e.g., using ImageJ software.

Reporting for specific materials, systems and methods

We require information from authors about some types of materials, experimental systems and methods used in many studies. Here, indicate whether each material, system or method listed is relevant to your study. If you are not sure if a list item applies to your research, read the appropriate section before selecting a response.

Materials & experimental systems

n/a	Involved in the study
☒	☐ Antibodies
☐	☒ Eukaryotic cell lines
☒	☐ Palaeontology and archaeology
☒	☐ Animals and other organisms
☒	☐ Clinical data
☒	☐ Dual use research of concern
☒	☐ Plants

Methods

n/a	Involved in the study
☒	☐ ChIP-seq
☒	☐ Flow cytometry
☒	☐ MRI-based neuroimaging

Eukaryotic cell lines

Policy information about cell lines and Sex and Gender in Research

Cell line source(s)	HEK293T cells were acquired from ATCC (CRL-3216).
Authentication	Cell lines were purchased from trusted vendor and not further authenticated in our laboratory.
Mycoplasma contamination	Cell lines are tested on regular basis once per year and infected cell lines are discarded.
Commonly misidentified lines (See ICLAC register)	None were used.

Plants

Seed stocks	Not relevant, as no experiments on plants were performed in this study.
Novel plant genotypes	Not relevant, as no experiments on plants were performed in this study.
Authentication	Not relevant, as no experiments on plants were performed in this study.
